# Synthesis of Pyrroles via Consecutive 6π-Electrocyclization/Ring-Contraction of Sulfilimines

**DOI:** 10.1021/jacs.1c04835

**Published:** 2021-06-09

**Authors:** Franz-Lucas Haut, Niklas J. Feichtinger, Immanuel Plangger, Lukas A. Wein, Mira Müller, Tim-Niclas Streit, Klaus Wurst, Maren Podewitz, Thomas Magauer

**Affiliations:** † Institute of Organic Chemistry and Center for Molecular Biosciences, Leopold-Franzens-University Innsbruck, Innrain 80-82, 6020 Innsbruck, Austria; ‡ Institute of General, Inorganic and Theoretical Chemistry and Center for Molecular Biosciences, Leopold-Franzens-University Innsbruck, Innrain 80-82, 6020 Innsbruck, Austria

## Abstract

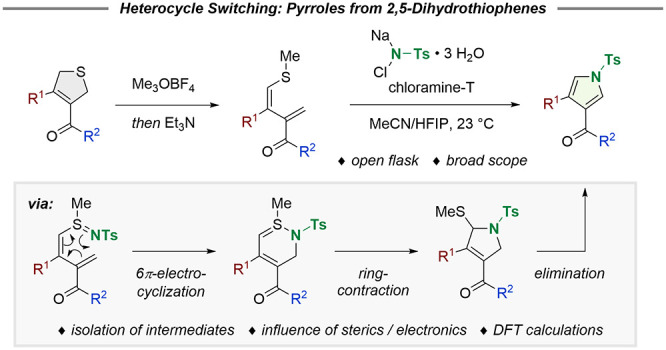

We present a modular, synthetic entry to polysubstituted pyrroles employing readily available 2,5-dihydrothiophenes. Ring-opening of the heterocycle provides access to a panel of 1,3-dienes which undergo pyrrole formation in the presence of inexpensive chloramine-T trihydrate. The transformation is conducted in an open flask and proceeds at ambient temperatures (23 °C) in nondry solvents. A careful adjustment of the electronics and sterics of the 1,3-diene precursor allows for the isolation of key intermediates. DFT studies identified a reaction mechanism that features a 6π-electrocyclization of a sulfilimine intermediate followed by spontaneous ring-contraction to reveal the pyrrole skeleton.

The efficient construction of structurally encumbered and highly functionalized heterocycles represents one of the major challenges for the development of novel pharmaceuticals and agrochemicals.^1^ In particular, tetrasubstituted pyrroles have served as valuable lead structures in medicinal chemistry to develop the anticancer agent sunitinib (**1**, Sutent),^2^ the cholesterin-lowering drug atorvastatin (**2**, Lipitor),^3^ and the Ca^2+^-channel activator FPL 64176 (**3**, Scheme 1A).^4^ For the assembly of these heterocycles, condensation chemistry has dominated the field for decades^5^ and powerful transition-metal based coupling strategies have only emerged later.^6^ Ring formation relying on pericyclic reactions represents a conceptionally different strategy which has found widespread application in all areas of heterocyclic chemistry. For instance, with the establishment of 1,3-dipoles by Huisgen, cycloaddition reactions became available as a robust method to synthesize a variety of five-membered heterocycles.^7^ This includes the [3 + 2]-cycloaddition reaction of azomethine, carbonyl, and thiocarbonyl ylide intermediates to allow for the rapid assembly of pyrroles, furans, and thiophenes.^8^ On the other hand, sigmatropic rearrangements have been extensively used to construct, for instance, indoles.^9^ For the synthesis of benzofuran derivatives, interrupted Pummerer reactions^10^ were reported to initiate charge-accelerated [3,3]-sigmatropic rearrangements.^11^ However, electrocyclization reactions have remained in a niche and have mainly been applied to the synthesis of six-membered heterocycles. For example, the 6π-electrocyclization of azatrienes was shown to provide a broad range of pyridines.^12^

During our studies to convert readily available 2,5-dihydrothiophenes **4**(13) into tetrasubstituted furans **6**, we found an unprecedented 6π-electrocyclic ring-opening as part of the reaction mechanism (Scheme 1B).^14^ While we were able to access a variety of furans, all efforts to prepare the corresponding pyrroles via exchange of the carbonyl function for an imine failed. However, we later found that the exposure of 1,3-diene **5a** to inexpensive chloramine-T effects selective sulfilimine formation. In contrast to a preliminary study relying on high temperatures (130 °C, two examples),^15^ subsequent 6π- electrocyclization/ring-contraction/elimination^16^ of **7** proceeded spontaneously at 23 °C in an open flask to give pyrrole **9** (Scheme 1C).

**Scheme 1:**
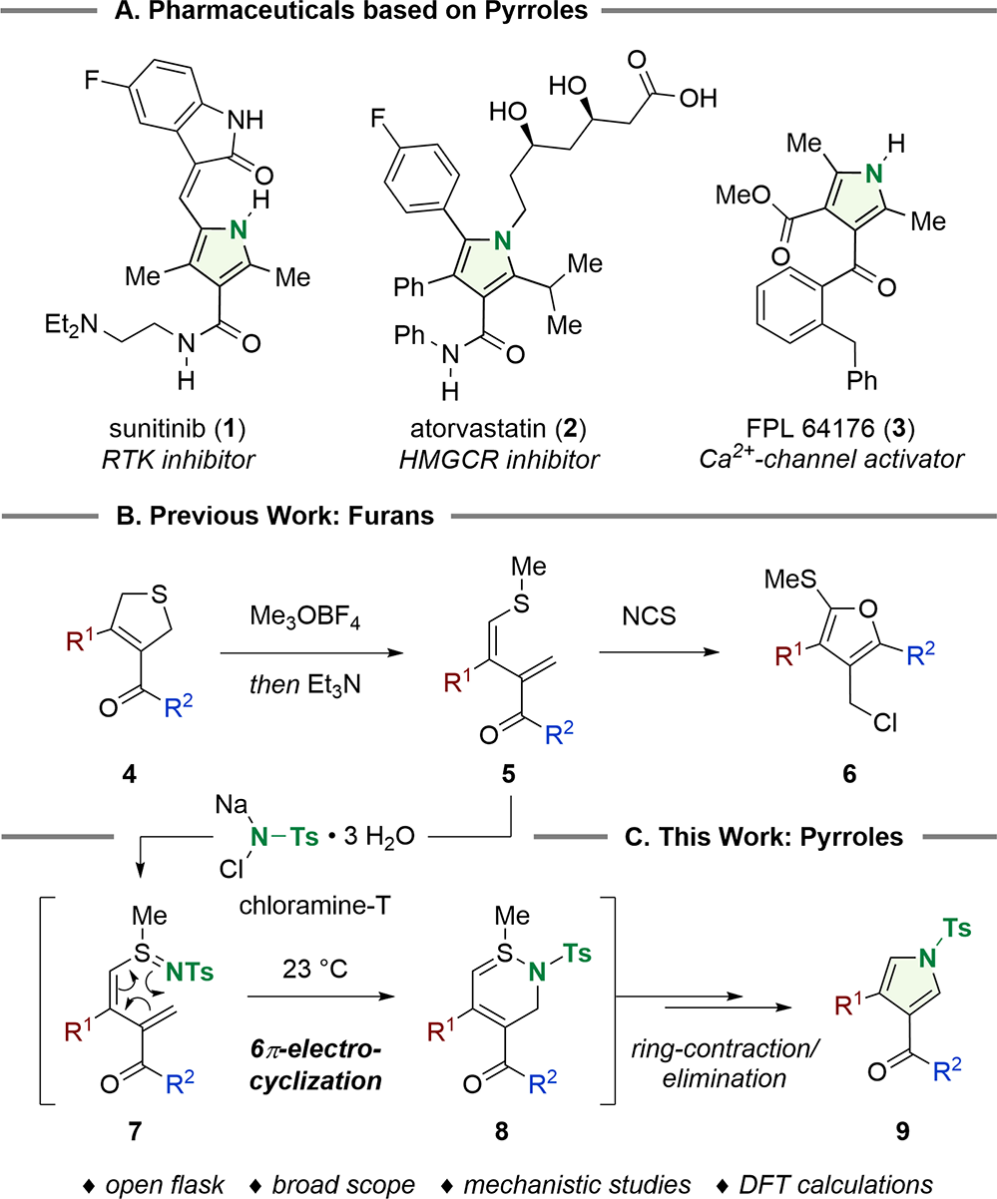
Pyrroles in Medicinal Chemistry and “Heterocycle Switches” of 2,5-Dihydrothiophenes into Furans and Pyrroles

Employing Sharpless’ conditions for the synthesis of *N*-tosyl sulfilimines (chloramine-T trihydrate, acetonitrile, 23 °C),^17^ we observed rapid conversion of 1,3-diene **5a** to pyrrole **9a** in 53% yield (Scheme 2, entry 1). The 2,5-dihydropyrrole **10** was isolated as the second product together with traces of trisubstituted pyrrole **11**, which might originate from **10** via a competing oxidation pathway. Further screening revealed slightly lower yields for the solvents *N*,*N*-dimethylformamide, methanol, and water (32–49%, entries 2–4). In the presence of 1 equiv of *p*-toluenesulfonic acid monohydrate (*p*-TsOH·H_2_O, entry 5), the yield was increased to 70%. The use of hexafluoroisopropyl alcohol (HFIP) as the cosolvent allowed for the removal of *p*-TsOH·H_2_O and further improved the yield of **9a** to 84% (entry 6). The use of 1.5 equiv of chloramine-T trihydrate or anhydrous chloramine-T (2 equiv) led to decreased yields (41–65%, entries 7 and 8). Dichloramine-T (TsNCl_2_) led to rapid consumption of the substrate, but pyrrole formation was accompanied by decomposition to give **9a** in only 23% yield. Variation of the vinyl sulfide revealed diene **5a** (R = Me) to be superior to **5b** (R = Et, 68%) and **5c** (R = Ph, 59%), delivering pyrrole **9a** in an 83% isolated yield. The addition of *m*-chloroperbenzoic acid (*m*-CPBA) after full conversion of the starting material allowed for selective sulfur oxidation of **11** and facilitated the isolation of pure **9a**.

**Scheme 2:**
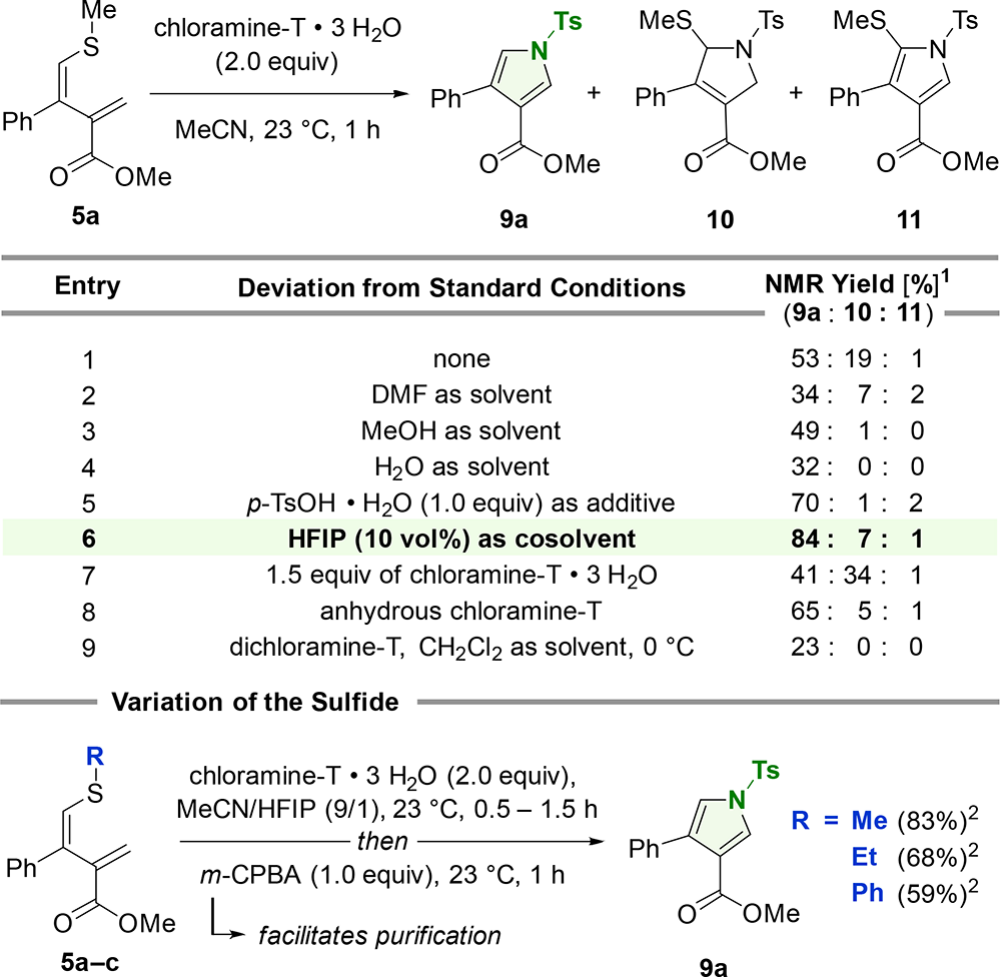
Optimization Studies Legend: (1) yield determined by ^1^H NMR analysis using nitromethane as internal standard; (2) isolated yield, 0.2 mmol scale of **5a**–**c**. Abbreviations: Ts = *p*-toluenesulfonyl, DMF = *N*,*N*-dimethylformamide, HFIP = hexafluoroisopropyl alcohol, *m*-CPBA = *m*-chloroperbenzoic acid.

With our optimized conditions in hand, we investigated the robustness and compatibility of the protocol for a panel of 1,3-dienes (Scheme 3). The scalability was demonstrated by the rapid synthesis of more than 1.5 g (78%) of pyrrole **9a** in a single run. Modifications of R^1^ (highlighted in red) allowed for the implementation of electronically enriched arenes and a thiophene to give **9b**–**d** in constantly good yields (72–79%). The presence of a strongly electron withdrawing substituent such as a nitro group (**9e**) or a trifluoromethyl group (**9f**) was well tolerated (63–64%). Different aryl halides were also shown to effectively undergo pyrrole formation to deliver chloride **9g**, fluoride **9h**, and bromide **9i** in high yields between 69 and 78%. In addition, tertiary amide **9j** and aldehyde **9k** were accessible from the reaction (59–65%). As shown for the synthesis of the alkyl (R^1^ = Me, *n*-Bu)- and allyl-substituted pyrroles **9l**–**n** (52–76%), an aryl residue was not required at the C3 position. Only alkyne **9o** and pivalate **9p** were obtained in lower yields (28–30%). Lactone **9q** (42%) was also accessible, thus expanding the synthetic utility to annelated ring systems. When the ester was changed to amides (R^2^, highlighted in blue), the primary and secondary amides **12a**,**b** were isolated in 56 and 81% yields, respectively. The latter bears the 3,4-substitution pattern as found in atorvastatin (**2**). Additionally, the Weinreb amide **12c** was synthesized in 33% yield. Ketones also participated in the transformation and gave the di- and trisubstituted pyrroles **13a**–**c** in good yields (55–77%). The presence of nitriles was also tolerated under the reaction conditions but required the absence of *m*-CPBA during the workup process. This allowed for the isolation of pyrrole **14a** in 51% yield (18% in the presence of *m*-CPBA). Consequently, we were able to prepare pyrrole **14b** (42%), which was quantitively converted to the fungicide fludioxonil (**15**, Pestanal)^1c,18^ through *N*-tosyl cleavage under basic conditions (NaOH, MeOH). Application of *O*-mesitylenesulfonyl hydroxylamine (MSH) and sodium carbonate^19^ allowed for the direct conversion of 1,3-diene **5a** to the unprotected pyrrole **16** (30%), which was produced in higher yields via deprotection of **9a** (Cs_2_CO_3_, MeOH, 84%). To conclude the synthetic scope, we explored the productivity of other chloramines to trigger the pyrrole formation of **5a**. Commercially available chloramine-B monohydrate allowed for the construction of pyrrole **17a** in 88% yield. When its *p*-nitrophenyl (chloramine-N), *p*-methoxyphenyl (chloramine-P) and methyl (chloramine-M) derivatives were applied, pyrroles **17b**–**d** were also accessible in yields up to 75%.

**Scheme 3:**
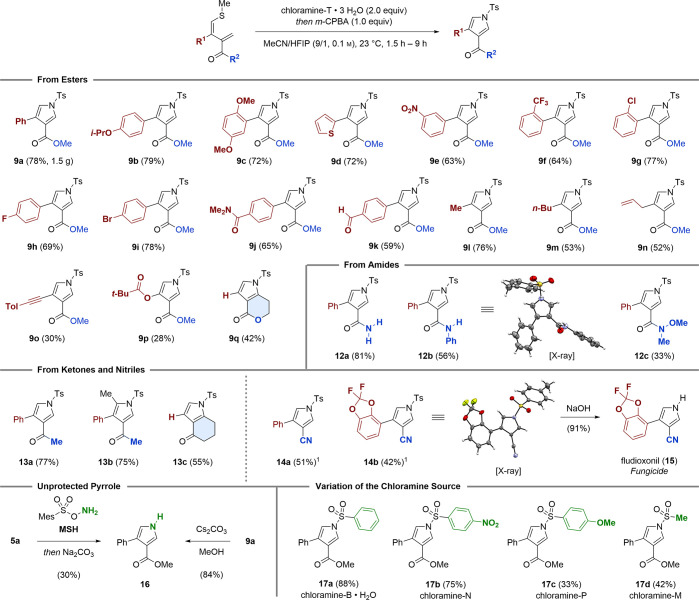
Synthetic Scope Standard conditions: substrate (0.2 mmol), chloramine-T trihydrate (2.0 equiv), MeCN/HFIP (9/1, 0.1 M), 0.5–8 h and then *m*-CPBA (1.0 equiv), 23 °C, 1 h. See Section 4.1 in the Supporting Information for experimental and substrate specific details. Legend: (1) no addition of *m*-CPBA.

By changing to sterically encumbered 1,3-dienes such as **18**, we were able to isolate the reactive sulfilimine **19** (61% yield, step A) under the standard reaction conditions (Scheme 4A). To our delight, thermal activation (toluene, reflux) allowed for the smooth initiation of the subsequent cascade to deliver pyrrole **20** in decent yield (76%, step B). When this two-step protocol was applied, trisubstituted pyrrole **21** (78% and 49%) and tetrasubstituted pyrrole **22** (61% and 99%) were formed. In addition, trisubstituted pyrrole **23** was obtained in good yields (62%), provided that benzonitrile was employed as the solvent.^20^ As exemplified by **24**, we found that the absence of an electron-withdrawing group (EWG) also allows for the isolation of its corresponding sulfilimines (99% yield, step A) under the standard reaction conditions. After this, thermal activation resulted in the formation of pyrrole **24** in quantitative yield. It is worth noting that, when sulfilimine **25** was subjected to thermal conditions (111 °C), a complete reaction was observed within 20 min. However, the main product was identified as the 2,5-dihydropyrrole **26** (44%) accompanied by small quantities of its *cis*-fused diastereomer (not shown, 10%) and pyrrole **27** (10%). Resubjecting **26** to refluxing toluene led to full conversion (28 h) to **27** in quantitative yield through the thermal release of methanethiol. Finally, sulfilimine **25** was directly converted into pyrrole **27** in 92% yield after an extended reaction time (44 h, step B).

**Scheme 4:**
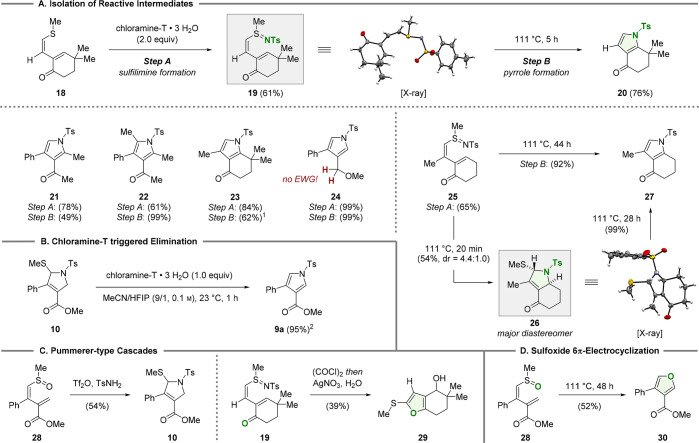
Mechanistic Investigations See Section 4.3 in the Supporting Information for experimental details. Legend: (1) benzonitrile as the solvent, 191 °C, 1 h (step B); (2) yield determined by ^**1**^H NMR analysis using methyl phenyl sulfone as an internal standard.

Having investigated the synthetic scope, we conducted further experiments to gain insights into the mechanism of the pyrrole formation. Thereby, chloramine-T was shown to effectively trigger the elimination of methyl sulfide from 2,5-dihydropyrrole **10** at ambient temperatures (23 °C, Scheme 4B). This revealed that 2 equiv of chloramine-T is required for full conversion and to avoid formation of a mixture of pyrrole and 2,5-dihydropyrrole (compare Scheme 2, entry 7). In addition, **10** was obtained through a Pummerer-type activation of sulfoxide **28** in the presence of triflic anhydride (Tf_2_O) and *p*-toluenesulfonamide (TsNH_2_, Scheme 4C).^21^ The lack of chloramine-T under these reaction conditions allowed for the selective formation of the 2,5-dihydropyrrole core without further elimination.

A second Pummerer-type reaction was demonstrated by the activation of sulfilimine **19** with oxalyl chloride (COCl)_2_.^22^ On the basis of our previous work,^14^**19** was rapidly converted into a trisubstituted furan bearing an unstable benzylic chloride. By telescoping the reaction in a one-pot fashion, the chloride was hydrolyzed (silver nitrate, acetone/water) to deliver furan **29** (39%). Finally, we adapted the 6π-electrocyclization/ring-contraction sequence for sulfoxide **28**, resulting in the smooth formation of the 3,4-substituted furan **30** (52%, Scheme 4D).

In a continuation of our mechanistic studies, DFT calculations (B3LYP-D3/6-311++G(2d,2p)) in implicit acetonitrile shed light on the rapid conversion of 1,3-diene **5a** to pyrrole **9a** at ambient temperature (Scheme 5, highlighted in black). Sulfilimine **A** is initially generated from the reaction of **5a** with chloramine-T, which is supported by the isolation of sulfilimines such as **19**.^23^ A thermal 6π-electrocyclization via **TS-A** with a barrier of Δ*G*^⧧^ = 13.5 kcal/mol results in the formation of 2,3-dihydrothiazine **B**. Facile ring-contraction through a 1,2-aza shift with a low activation energy (Δ*G*^⧧^ = 6.0 kcal/mol, **TS-B**) delivers the thermodynamically favored 2,5-dihydropyrrole **10** (Δ*G* = −39.5 kcal/mol), which could be isolated in the absence of chloramine-T (compare Scheme 2). Since a second equivalent of chloramine-T was shown to rapidly promote the final aromatization step (compare Scheme 4B), we assume an exergonic sulfilimine formation with ΔΔ*G* = −25.3 kcal/mol to yield **C**, which undergoes spontaneous elimination to give pyrrole **9a** and sulfonamide **31**.^24^

**Scheme 5:**
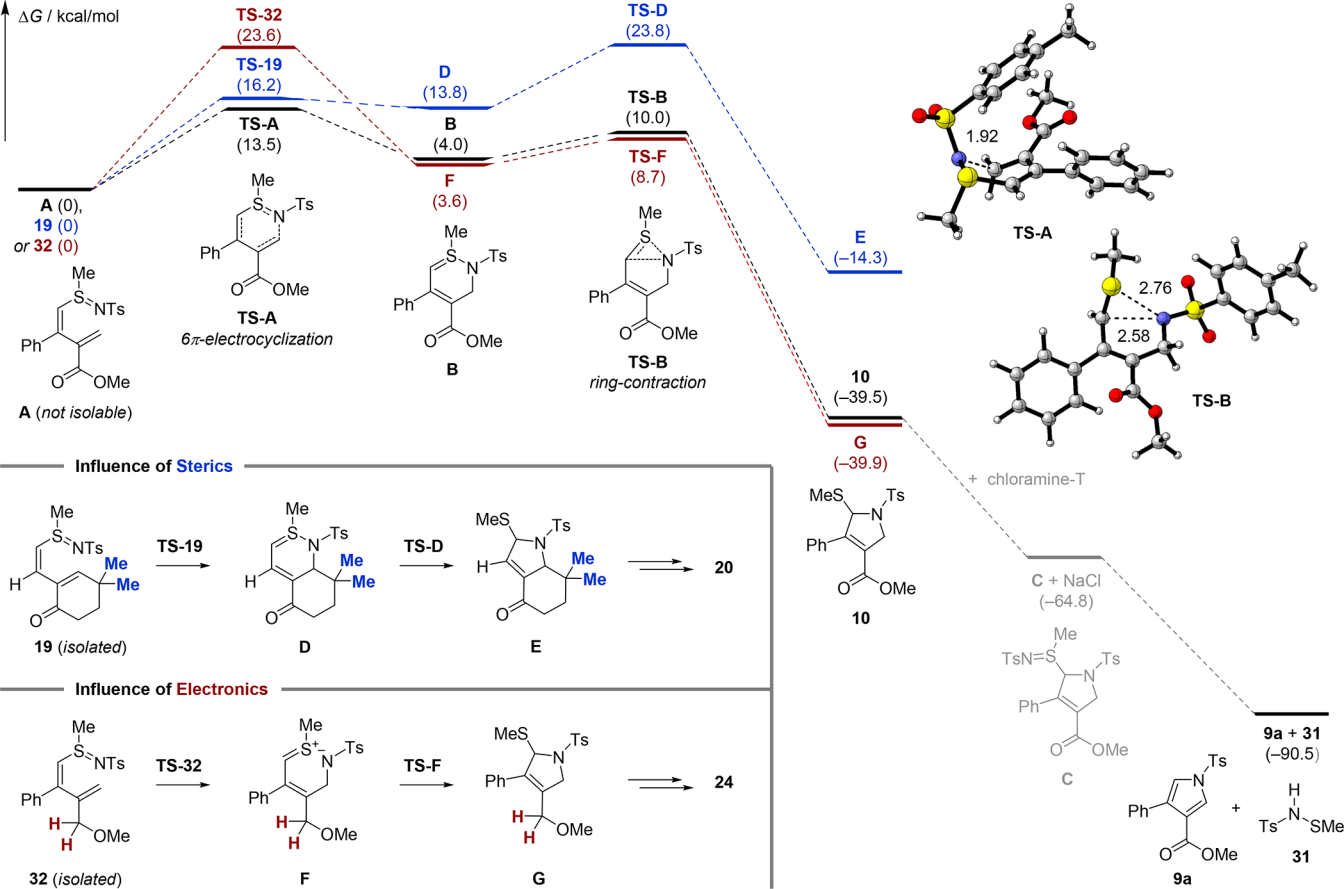
Computational Studies Proposed reaction mechanism as calculated with B3LYP-D3/6-311++G(2d,2p) in acetonitrile treated as the implicit solvent (see Section 6 in the Supporting Information for details). Relative Gibbs free energies at 298 K are given in kcal/mol, whereas the energies of the respective sulfilimines **A**, **19**, and **32** are arbitrarily set to zero. The energetically most favorable pathway for 1,3-diene **5a** to pyrrole **9a** is highlighted in black. For comparison, the influences of sterics (blue, **19** → **20**) and electronics (red, **32** → **24**) were investigated.

On the basis of the isolation of several reactive intermediates (Scheme 4A), additional calculations were carried out to explain the kinetic hindrance. For the sterically encumbered sulfilimine **19** (highlighted in blue), we found only a slightly increased barrier for the 6π-electrocyclization (**TS-19**) in comparison to **TS-A** with ΔΔ*G*^⧧^ = 2.7 kcal/mol. However, the formation of 2,3-dihydrothiazine **D** as well as the ring-contraction product **TS-D** is energetically increased (ΔΔ*G* = 9.8 kcal/mol and ΔΔ*G*^⧧^ = 13.8 kcal/mol) due to the rigidity of the annelated cyclohexene bearing the *gem*-dimethyl substitution pattern.^25^ Intermediate **D** was found to kinetically favor the back reaction, a 6π-electrocyclic ring-opening, to regenerate **19** instead of undergoing ring-contraction via **TS-D** to 2,5-dihydropyrrole **E** (ΔΔ*G*^⧧^ = 7.6 kcal/mol). Consequently, the product formation is kinetically suppressed at ambient temperature (23 °C), thus allowing for the isolation of **19**. This is fully consistent with the thermal activation of **19** (111 °C, Scheme 4A) resulting in the formation of pyrrole **20** via intermediate **E**.

The lack of an EWG (highlighted in red) significantly increases the activation energy for the 6π-electrocyclization of sulfilimine **32** (ΔΔ*G*^⧧^ = 10.1 kcal/mol, **TS-32** vs **TS-A**).^26^ In contrast to 2,3-dihydrothiazines **B** and **D**, the charge-separated intermediate **F** is preferentially formed, in which heterolytic cleavage of the S–N bond is observed. However, the ring-contraction barrier for **TS-F** is comparable to that of **TS-B** (ΔΔ*G*^⧧^ = 1.3 kcal/mol), and the thermodynamics of 2,5-dihydropyrrole **G** are equal to those of **10**. The similarity of the thermodynamic profiles (**B** → **10** and **F** → **G**) stands in sharp contrast to the sterically deactivated pathway of intermediate **D** to **E**. Alternative pathways for the formation of the 2,5-dihydropyrroles **10**, **E**, and **G** have been investigated in detail (See Section 6 in the Supporting Information) but are energetically less favorable.

In summary, we have demonstrated the synthetic potential of 2,5-dihydrothiophene-derived sulfilimines to access a variety of polysubstituted pyrroles under mild reaction conditions. Both the experimental results and DFT calculations are fully consistent with a mechanism that involves a 6π-electrocyclization/ring-contraction sequence. Despite the omnipresence of pericyclic reactions in heterocyclic chemistry, electrocyclic reactions have been largely limited to the formation of six-membered heterocycles. The developed methodology fills that gap and expands the unique chemical space of electrocyclic reactions. Further studies toward related N-heterocycles are currently ongoing in our laboratories and will be reported in due course.

## Supporting Information Available

Experimental details, spectroscopic data, and details of the calculations (PDF)

## Accession Codes

CCDC 2081881–2081884 contain the supplementary crystallographic data for this paper. These data can be obtained free of charge via www.ccdc.cam.ac.uk/data_request/cif, or by emailing data_request@ccdc.cam.ac.uk, or by contacting The Cambridge Crystallographic Data Centre, 12 Union Road, Cambridge CB2 1EZ, UK; fax: +44 1223 336033.
