# Supplementary material for: Synthesis of Pyrroles via Consecutive 6π-Electrocyclization/Ring-Contraction of Sulfilimines
Source: J Am Chem Soc. 2021 Jun 9;143(24):9002–8. doi: 10.1021/jacs.1c04835 (PMC8227482; doi:10.1021/jacs.1c04835)
Supplement: Supplementary file 1 — ja1c04835_si_001.pdf [file ja1c04835_si_001.pdf]

# – Supporting Information –

Synthesis of Pyrroles via Consecutive 6π-Electrocyclization/Ring-Contraction of Sulfilimines

Franz-Lucas Haut[a], Niklas Johann Feichtinger[a], Immanuel Plangger[a], Lukas Anton Wein[a], Mira Müller[a], Tim-Niclas Streit[a], Klaus Wurst[b], Maren Podewitz[b,*] & Thomas Magauer[a,*]

[a] Institute of Organic Chemistry and Center for Molecular Biosciences, Leopold-Franzens-University Innsbruck, Innrain 80−82, 6020 Innsbruck, Austria.

[b] Institute of General, Inorganic and Theoretical Chemistry and Center for Molecular Biosciences, Leopold-Franzens-University Innsbruck, Innrain 80−82, 6020 Innsbruck, Austria.

* Corresponding authors:

maren.podewitz@uibk.ac.at

thomas.magauer@uibk.ac.at

Table of Contents

1 General Experimental Details **3**

2 Optimization Studies and General Procedures (GP) **5**

2.1 Pyrrole Formation (GP1) 5

2.2 Suzuki Cross-Coupling (GP2) 5

2.3 1,3-Diene Formation (GP3) 6

3 Optimization Studies **6**

4 Pyrroles and Mechanistic Studies **9**

4.1 Substrate Scope 9

4.2 Additional Substrates and Limitations 29

4.3 Mechanistic Investigations 32

4.4 Additional Mechanistic Experiments 44

5 Synthesis of 1,3-Dienes and Reagents **49**

5.1 Literature-known Compounds 49

5.2 1,3-Dienes 49

5.3 Reagents 79

6 Computational Studies **82**

6.1 Computational Methodology 82

6.2 Standard Reaction Pathway 83

6.3 Influence of Sterics 84

6.4 Influence of Electronics 85

6.5 Comparison of Transition States 86

6.6 Cartesian Coordinates 88

7 References **127**

8 NMR Spectra **130**

9 Crystallographic Data **243**

1 General Experimental Details

All reactions were performed in flame-dried glassware fitted with rubber septa under a positive pressure of argon, unless otherwise noted. Air- and moisture-sensitive liquids were transferred via syringe or stainless-steel cannula through rubber septa. Solids were added under inert gas counter flow or were dissolved in appropriate solvents. Reactions at low temperatures were carried out in a Dewar vessel filled with a cooling agent: acetone/dry ice (−78 °C), acetonitrile/dry ice (−40 °C), sodium chloride/ice (–18 °C) or distilled water/ice (0 °C). Reaction temperatures above 23 °C were conducted in a heated oil bath or a metal block. The reactions were magnetically stirred and monitored by NMR spectroscopy or analytical thin-layer chromatography (TLC), using aluminum plates precoated with silica gel (0.25 mm, 60 Å pore size, Merck, impregnated with a fluorescent indicator (254 nm). TLC plates were visualized by exposure to ultraviolet light (UV), were stained by submersion in aqueous potassium permanganate solution ($KMnO_4$), ceric ammonium molybdate solution (CAM) or bromocresol green (BCG) and were developed by heating with a heat gun.

Flash-Column Chromatography (FCC) was performed as described by Still et al.,[1] employing silica gel (60 Å, 40–63 µm, Merck). The yields refer to chromatographically and spectroscopically (^{1}H and ^{13}C NMR) pure material.

High Performance Liquid Chromatography (HPLC) was carried out on normal-phase Varian Dynamax columns. For semipreparative separations a 250 × 21.4 mm Microsorb 60–8 Si-column was used.

Solvents and Reagents as acetone, acetonitrile (MeCN), chloroform ($CHCl_3$), dichloromethane (CH_2Cl_2), diethyl ether (Et_2O), dimethyl sulfoxide (DMSO), ethanol (EtOH), ethyl acetate (EtOAc), methanol (MeOH), *N*,*N*-dimethylformamide (DMF), tetrahydrofuran (THF) and toluene (PhMe) were purchased from Acros Organics or Sigma Aldrich as 'extra dry' reagents and used as received. Meerwein’s salt (Me_3OBF_4, Sigma Aldrich), triethylamine (Et_3N, Sigma Aldrich) and chloramine-T trihydrate (Sigma Aldrich) were used without further purification. Other reagents or solvents were purchased from chemical suppliers (Sigma-Aldrich, TCI, Fisher Scientific or others) and were used as received. Solvents for extraction, crystallization and flash column chromatography as petroleum ether (40–60 °C) were purchased in technical grade and distilled under reduced pressure prior to use. The molarity of *n*-butyllithium (*n*-BuLi), *tert*-butyllithium (*t*-BuLi) and ethylmagnesium bromide (EtMgBr) solutions was determined by titration against diphenylacetic acid or iodine as an indicator (average of three determinations).[2,3]

NMR Spectra were measured on a Bruker Avance Neo 400 MHz spectrometer at *Leopold–Franzens University Innsbruck* (*LFU Innsbruck*). Proton chemical shifts are expressed in parts per million (ppm, δ scale) and are referenced to residual proton in the NMR solvent ($CHCl_3$: δ = 7.26). Carbon chemical shifts are expressed in parts per million (δ scale) and are referenced to the carbon resonance of the NMR solvent ($CDCl_3$: δ = 77.16). ^{1}H NMR spectroscopic data are reported as follows: Chemical shift in ppm (multiplicity, coupling constants *J* (Hz), integration intensity). The multiplicities are abbreviated with s (singlet), d (doublet), t (triplet), q (quartet) and m (multiplet). In case of combined multiplicities, the multiplicity with the larger coupling constant is stated first. Except for multiplets, the chemical shift of all signals, as well for centrosymmetric multiplets, is reported as the center of the resonance range. In addition, to ^{1}H and ^{13}C NMR measurements, 2D NMR techniques such as homonuclear correlation spectroscopy (COSY), total correlation spectroscopy (TOCSY), heteronuclear single quantum coherence (HSQC) and heteronuclear multiple bond coherence (HMBC) were used to assist signal assignment. For further elucidation of 3D structures of the products, nuclear Overhauser enhancement spectroscopy (NOESY) was conducted. Coupling constants *J* are reported in Hz. All raw fid files were processed and the spectra analyzed using the software MestReNova 12.0.2 from Mestrelab Research S. L.

Infrared Spectra (IR) were recorded on a PerkinElmer Spectrum BX II FT-IR system. If required, substances were dissolved in CH_2Cl_2 or $CDCl_3$ prior to direct application on the ATR unit. Data are represented as follows: frequency of absorption (cm^{-1}) and intensity of absorption (*vs* = very strong, *s* = strong, *m* = medium, *w* = weak, *br* = broad).

High-Resolution Mass Spectra (HRMS) as ESI-HRMS were recorded on a Thermo Scientific™ QExactive™ Orbitrap Mass Spectrometer at *LFU Innsbruck*. DESI-HRMS as well as LTP-HRMS were recorded on a Thermo Scientific™ LTQ Orbitrap XL™ Hybrid Ion Trap-Orbitrap Mass Spectrometer equipped with a 3-in-1 ambient ionization interface.[4] The ionization and detection of 2,5-dihydrothiophenes could be signifiCAMtly improved by the addition of silver(I) nitrate to give the corresponding silver adducts (only the mono-isotopic Ag-adduct is reported).

Melting Points (MP) were measured with an SRS-MPA120 EZ-Melt Melting Point Apparatus in open glass capillaries and are uncorrected.

2 Optimization Studies and General Procedures (GP)

2.1 Pyrrole Formation (GP1)

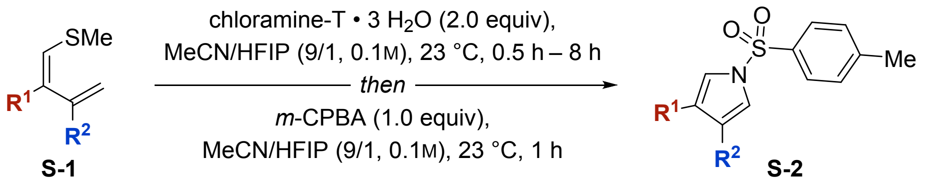

1,3-Diene **S-1** (0.20 mmol, 1 equiv) was placed in a vial (max. 20 mL) and dissolved in a mixture of acetonitrile and hexafluoroisopropanol (9:1, 2.0 mL). Chloramine-T trihydrate (115 mg, 400 µmol, 2.00 equiv) was added portionwise over ten minutes at 23 °C. Upon full conversion of the starting material, indicated by TLC analysis (0.5–8 h, see substrate specific details), 3-chloroperbenzoic acid (77%, 44.8 mg, 0.20 mmol, 1.0 equiv) was added in one portion at 23 °C. After one hour, saturated aqueous sodium thiosulfate solution (20 mL) and ethyl acetate (20 mL) were added, the layers were separated and the aqueous phase was extracted with ethyl acetate (2 × 20 mL). The combined organic layers were washed with saturated aqueous sodium bicarbonate solution (20 mL) and dried over sodium sulfate. The dried solution was filtered, the filtrate was concentrated under reduced pressure and the crude product was purified by flash-column chromatography on silica gel to furnish pyrrole **S-2**.

Note: The reaction was carried out without flame-drying the glassware and under air employing non-dried solvents (acetonitrile and hexafluoroisopropanol, HPLC-grade).

2.2 Suzuki Cross-Coupling (GP2)

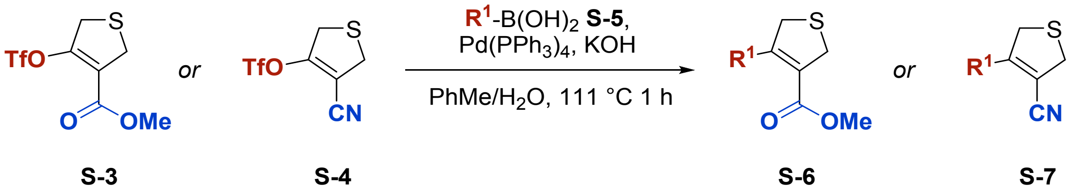

Triflate **S-3** or **S-4** (0.50 mmol, 1 equiv) was placed in a vial (max. 20 mL) under argon atmosphere and dissolved in toluene (0.1 M) under argon atmosphere. The solution was degassed by bubbling with argon for 20 minutes. Boronic acid **S-5** (0.60 mmol, 1.2 equiv), tetrakis-(triphenylphosphine)palladium (29 mg, 25 µmol, 5.0 mol%) and aqueous potassium hydroxide solution (4.0 M, 0.15 mL, 0.60 mmol, 1.2 equiv) were added at 23 °C. Upon complete addition the mixture was heated to 111 °C. After one hour, the reaction mixture was allowed to cool to 23 °C, filtered over a plug of Celite® and rinsed with diethyl ether (50 mL). Water (50 mL) was added, the layers were separated and the aqueous layer was extracted

with diethyl ether (2 × 50 mL). The combined organic layers were dried over sodium sulfate, the dried solution was filtered and the filtrate was concentrated under reduced pressure. The crude product was purified by flash-column chromatography on silica gel to furnish 2,5-dihydrothiophene **S-6** or **S-7**, respectively.

2.3 1,3-Diene Formation (GP3)

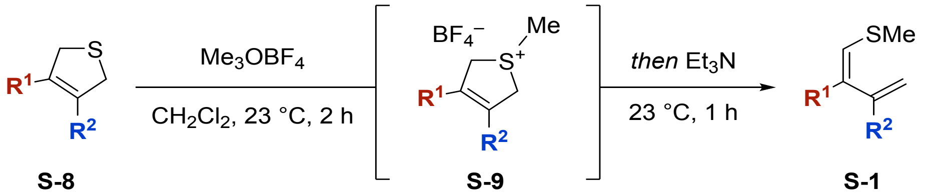

S-8 **S-9** **S-1**

2,5-Dihydrothiophene **S-8** (1 equiv) was placed in a vial (max. 20 mL) or a round-bottom flask under argon, was dissolved in dichloromethane (0.2 M) and trimethyloxonium tetrafluoroborate (1.5 equiv) was added in one portion at 23 °C. After two hours, triethylamine (3.0 equiv) was added dropwise at 23 °C. After one hour, the solvent was removed under reduced pressure and the crude product was purified by flash-column chromatography on silica gel to furnish diene **S-1**.

Note: The conversion of 2,5-dihydrothiophene **S-8** to sulfonium salt **S-9** was monitored either by ^{1}H NMR or TLC analysis of the reaction mixture.

3 Optimization Studies

Solvent Screening

1,3-Diene **5a** (12 mg, 50 µmol, 1 equiv) was dissolved in different solvents (0.50 mL) and chloramine-T trihydrate (1.0 – 4.0 equiv) was added in one portion at 23 °C (Table 1). After full conversion of the starting material (indicated by TLC analysis) or after one hour, saturated aqueous sodium thiosulfate solution (10 mL) and dichloromethane (10 mL) were added. The layers were separated and the aqueous layer was extracted with dichloromethane (2 × 10 mL). The combined organic layers were dried over sodium sulfate, the dried solution was filtered and the filtrate was concentrated under reduced pressure. Nitromethane (50 µmol) was added as an internal standard to the crude reaction mixture to determine the yield of pyrroles **9a**, **10** and **11** as well as sulfoxide **28** through ^{1}H NMR analysis.

Table 1: Solvent Screening.

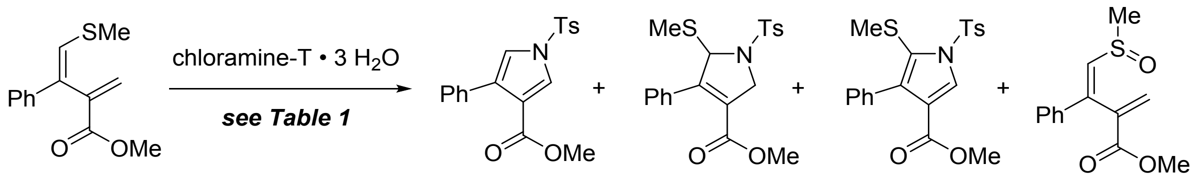

5a **9a** **10** **11** **28**

Entry	Chloramine-T [equiv]	Solvent	^{1}H NMR yield [%]				
			**9a**	**10**	**11**	**28**	**5a**
1[1]	1.0	MeCN	14	47	0	9	18
2	1.0	MeCN	18	34	0	7	23
3	1.2	MeCN	16	17	0	6	14
4	1.5	MeCN	41	34	1	13	7
5	2.0	MeCN	53	19	1	9	0
6[1]	2.0	MeCN	47	3	1	4	0
7	3.0	MeCN	52	0	1	7	0
8	4.0	MeCN	53	0	1	4	0
9	2.0	H_2O	32	0	0	23	12
10	2.0	DMF	34	7	2	13	1
11	2.0	MeOH	49	1	0	4	0
12	2.0	CH_2Cl_2	25	0	1	45	0
13[2]	2.0	CH_2Cl_2	23	0	0	0	0
14	2.0	EtOAc	29	0	1	47	0
15	2.0	THF	44	0	3	40	3
16	2.0	Acetone	40	16	1	21	4
17	2.0	*i*-PrOH	52	11	1	12	7
18	2.0	*i*-PrOH/MeCN	37	21	1	5	1
19	2.0	MeCN/HFIP (1/1)	61	8	1	6	0
**20**	**2.0**	**MeCN/HFIP (9/1)**	**84**	**7**	**1**	**4**	**0**
21[3]	2.0	MeCN/HFIP (9/1)	61	0	1	12	0
22[4]	2.0	MeCN/HFIP (9/1)	65	5	1	3	0

[1]The reaction was conducted at 0 °C. [2]The reaction was conducted with dichloramine-T at 0 °C. [3]The reaction was conducted at 40 °C. [4]Anhydrous chloramine-T was used.

Additive Screening

To a solution of 1,3-diene **5a** (12 mg, 50 µmol, 1 equiv) in acetonitrile (0.5 mL) were added the additive (10 mol%) and chloramine-T (29 mg, 0.10 mmol, 2.0 equiv) at 23 °C. After the indicated reaction time (see Table 2), saturated aqueous sodium thiosulfate solution (10 mL) and dichloromethane (10 mL) were added. The layers were separated and the aqueous layer was extracted with dichloromethane (2 × 10 mL). The combined organic layers were dried over sodium sulfate, the dried solution was filtered and the filtrate was concentrated under reduced pressure. Nitromethane (50 µmol) was added as an internal standard to the crude reaction mixture to determine the yield of pyrroles **9a**, **10** and **11** as well as sulfoxide **28** through ^{1}H NMR analysis.

Table 2: Additive Screening.

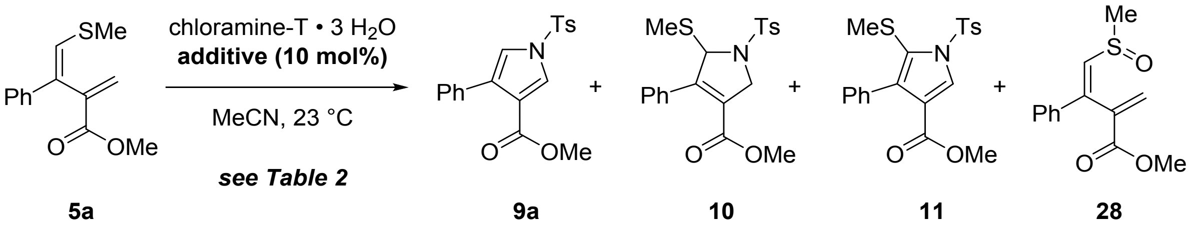

Entry	Additive	Time [h]	^{1}H NMR yield [%] 9	10	11	28	5a
1	MS 3Å	1	54	12	2	12	6
2[1]	$MgSO_4$	1	54	14	1	12	2
3	$Zn(OAc)_2$	19	36	14	1	15	0
4	ZnI_2	22	0	0	0	0	0
5	*p*-TsOH • H_2O	1	70	1	2	11	1
6[2]	*p*-TsOH • H_2O	1	65	5	0	9	0
7[1,2]	*p*-TsOH • H_2O	0.5	70	0	2	6	5
8	Amberlyst 15	0.5	50	8	1	14	0
9	TCA	1	44	0	2	41	0
10	Oxalic acid • 2 H_2O	1	67	0	2	18	0
11	(*R*,*R*)-CSA	0.5	57	7	2	13	0
12	$TsNH_2$	2	66	5	1	12	0
13	DIPEA	5	20	7	10	4	46
14	Piperidine	5	4	3	0	0	75

[1]1.0 equiv of additive was added. [2]The additive was added five minutes after chloramine-T addition.

4 Pyrroles and Mechanistic Studies

4.1 Substrate Scope

Pyrrole 9a

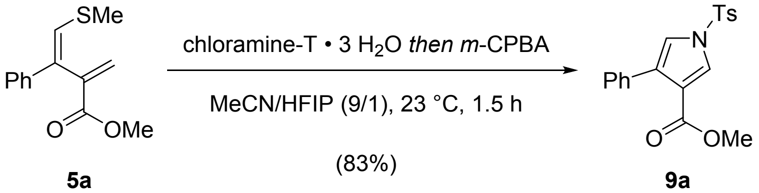

5a **9a**

Following GP1 (20 minutes for pyrrole formation), 1,3-diene **5a** (47 mg, 0.20 mmol) was converted into pyrrole **9a**. Purification by flash-column chromatography on silica gel (20% ethyl acetate in cyclohexane) furnished pyrrole **9a** (59 mg, 83%) as a pale-yellow oil.

TLC (50% diethyl ether in petroleum ether): R_f = 0.41 (UV, $KMnO_4$).

^{1}H NMR ($CDCl_3$, 400 MHz): δ = 7.85 – 7.81 (m, 3H), 7.43 – 7.38 (m, 2H), 7.38 – 7.30 (m, 5H), 7.13 (d, J = 2.6 Hz, 1H), 3.73 (s, 3H), 2.43 (s, 3H).

^{13}C NMR ($CDCl_3$, 101 MHz): δ = 163.8, 146.1, 135.2, 132.7, 130.5, 130.2, 129.3, 128.0, 127.7, 127.5, 126.8, 119.5, 118.2, 51.5, 21.9.

IR (Diamond-ATR, CH_2Cl_2): $\tilde{\upsilon}_{max}$ = 1727 (*m*), 1377 (*m*), 1269 (*m*), 1189 (*w*), 1174 (*s*), 1124 (*m*), 1066 (*m*), 761 (*w*), 672 (*s*), 597 (*w*).

HRMS (ESI) calc. for $C_{19}H_{17}NNaO_4S$ $[M+Na]^+$: 378.0770; found: 378.0761.

Upscale Experiment:

Following GP1 (one hour for pyrrole formation), 1,3-diene **5a** (1.30 g, 5.55 mmol) was converted into pyrrole **9a**. Purification by flash-column chromatography on silica gel (20% ethyl acetate in cyclohexane) furnished pyrrole **9a** (1.54 g, 78%) as a pale-yellow oil. The obtained analytical data was in full agreement with those obtained from previous experiments.

Pyrrole 9a

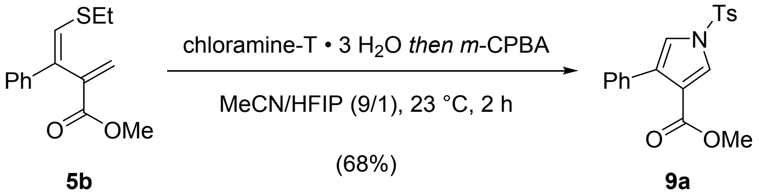

5b **9a**

Following GP1 (one hour for pyrrole formation), 1,3-diene **5b** (50 mg, 0.20 mmol) was converted into pyrrole **9a**. Purification by flash-column chromatography on silica gel (20% to 50% diethyl ether in petroleum ether) furnished pyrrole **9a** (49 mg, 68%) as a pale-yellow oil.

The obtained analytical data was in full agreement with those obtained from previous experiments.

Pyrrole 9a

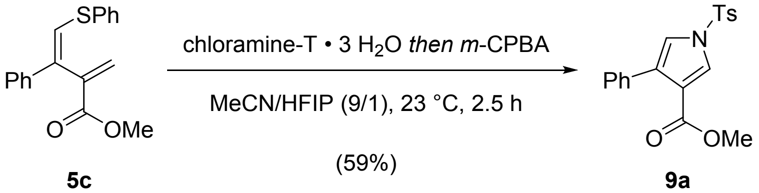

5c **9a**

Following GP1 (1.5 hours for pyrrole formation), 1,3-diene **5c** (59 mg, 0.20 mmol) was converted into pyrrole **9a**. Purification by flash-column chromatography on silica gel (20% to 50% diethyl ether in petroleum ether) furnished pyrrole **9a** (42 mg, 59%) as a pale-yellow oil. The obtained analytical data was in full agreement with those obtained from previous experiments.

Pyrrole 9b

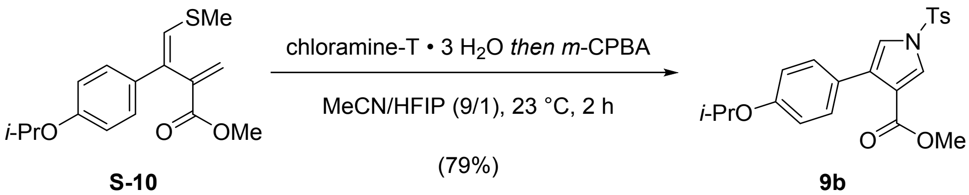

S-10 **9b**

Following GP1 (one hour for pyrrole formation), 1,3-diene **S-10** (59 mg, 0.20 mmol) was converted into pyrrole **9b**. Purification by flash-column chromatography on silica gel (10% to 20% ethyl acetate in cyclohexane) furnished pyrrole **9b** (65 mg, 79%) as a yellow oil.

TLC (20% ethyl acetate in cyclohexane): R_f = 0.35 (UV, $KMnO_4$).

^{1}H NMR ($CDCl_3$, 400 MHz): δ = 7.84 – 7.79 (m, 3H), 7.36 – 7.30 (m, 4H), 7.08 (d, *J* = 2.6 Hz, 1H), 6.89 – 6.83 (m, 2H), 4.55 (dt, *J* = 12.1, 6.1 Hz, 1H), 3.74 (s, 3H), 2.43 (s, 3H), 1.34 (d, *J* = 6.0 Hz, 6H).

^{13}C NMR ($CDCl_3$, 101 MHz): δ = 164.0, 157.7, 146.0, 135.3, 130.5, 130.4, 130.0, 127.5, 126.8, 124.8, 119.0, 118.1, 115.4, 70.0, 51.5, 22.2, 21.9.

IR (Diamond-ATR, $CDCl_3$): $\tilde{\upsilon}_{max}$ = 1725 (*m*), 1519 (*w*), 1375 (*m*), 1243 (*m*), 1173 (*s*), 1121 (*s*), 1090 (*w*), 1064 (*s*), 675 (*s*), 595 (*m*).

HRMS (ESI) calc. for $C_{22}H_{23}NNaO_5S$ $[M+Na]^+$: 436.1189; found: 436.1182.

Pyrrole 9c

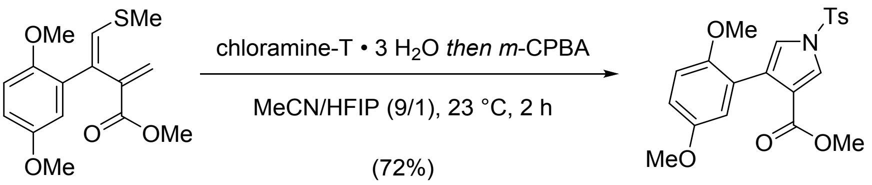

S-11 **9c**

Following GP1 (one hour for pyrrole formation), 1,3-diene **S-11** (59 mg, 0.20 mmol) was converted into pyrrole **9c**. Purification by flash-column chromatography on silica gel (50% diethyl ether in petroleum ether) furnished pyrrole **9c** (60 mg, 72%) as a yellow foam.

TLC (50% diethyl ether in petroleum ether): R_f = 0.13 (UV, $KMnO_4$).

^{1}H NMR ($CDCl_3$, 400 MHz): δ = 7.85 – 7.80 (m, 2H), 7.76 (d, *J* = 2.5 Hz, 1H), 7.36 – 7.31 (m, 2H), 7.14 (d, *J* = 2.5 Hz, 1H), 6.84 – 6.78 (m, 3H), 3.77 (s, 3H), 3.68 (s, 3H), 3.66 (s, 3H), 2.43 (s, 3H).

^{13}C NMR ($CDCl_3$, 101 MHz): δ = 164.1, 153.4, 151.5, 146.0, 135.4, 130.4, 127.5, 125.6, 125.4, 123.0, 120.1, 119.9, 116.8, 113.6, 111.8, 56.1, 55.9, 51.5, 21.8.

IR (Diamond-ATR, CH_2Cl_2): $\tilde{\upsilon}_{max}$ = 1730 (*w*), 1491 (*w*), 1377 (*w*), 1263 (*m*), 1218 (*w*), 1174 (*s*), 1114 (*m*), 1091 (*w*), 1065 (*m*), 672 (*m*).

HRMS (ESI) calc. for $C_{21}H_{21}NNaO_6S$ $[M+Na]^+$: 438.0982; found: 438.0968.

Pyrrole 9d

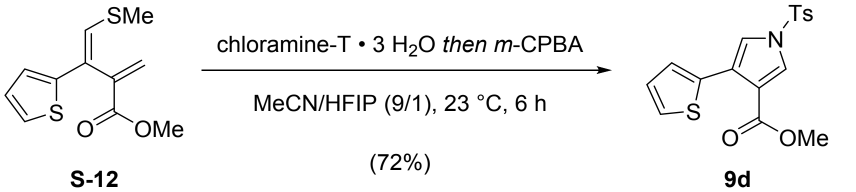

S-12 **9d**

Following GP1 (five hours for pyrrole formation), 1,3-diene **S-12** (48 mg, 0.20 mmol) was converted into pyrrole **9d**. Purification by flash-column chromatography on silica gel (10% to 20% ethyl acetate in cyclohexane) furnished pyrrole **9d** (52 mg, 72%) as a pale-yellow oil.

TLC (20% ethyl acetate in cyclohexane): R_f = 0.35 (UV, $KMnO_4$).

^{1}H NMR ($CDCl_3$, 400 MHz): δ = 7.83 – 7.81 (m, 3H), 7.38 – 7.33 (m, 3H), 7.28 – 7.24 (m, 2H), 7.03 (dd, *J* = 5.2, 3.6 Hz, 1H), 3.79 (s, 3H), 2.44 (s, 3H).

^{13}C NMR ($CDCl_3$, 101 MHz): δ = 163.6, 146.3, 135.1, 133.6, 130.5, 127.7, 127.5, 127.4, 127.1, 125.3, 122.8, 119.6, 117.9, 51.6, 21.9.

IR (Diamond-ATR, CH_2Cl_2): $\tilde{\upsilon}_{max}$ = 1726 (*m*), 1378 (*m*), 1255 (*w*), 1189 (*w*), 1174 (*s*), 1115 (*m*), 1089 (*w*), 1067 (*s*), 702 (*w*), 673 (*s*).

HRMS (ESI) calc. for $C_{17}H_{15}NNaO_4S_2$ $[M+Na]^+$: 384.0335; found: 384.0330.

Pyrrole 9e

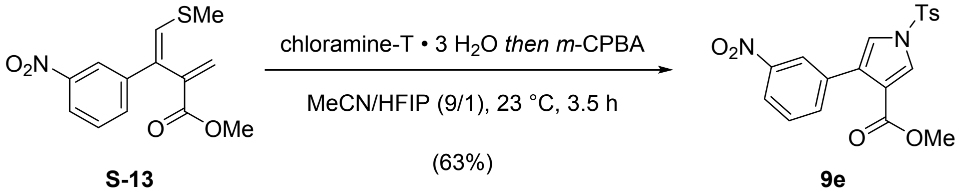

S-13 **9e**

Following GP1 (2.5 hours for pyrrole formation), 1,3-diene **S-13** (56 mg, 0.20 mmol) was converted into pyrrole **9e**. Purification by flash-column chromatography on silica gel (5% to 20% ethyl acetate in cyclohexane) furnished pyrrole **9e** (50 mg, 63%) as a colorless oil.

TLC (20% ethyl acetate in cyclohexane): R_f = 0.17 (UV, $KMnO_4$).

^{1}H NMR ($CDCl_3$, 400 MHz): δ = 8.27 (t, *J* = 2.0 Hz, 1H), 8.16 (ddd, *J* = 8.3, 2.3, 1.1 Hz, 1H), 7.89 – 7.86 (m, 2H), 7.85 (d, *J* = 1.9 Hz, 1H), 7.76 (dt, *J* = 7.8, 1.3 Hz, 1H), 7.51 (t, *J* = 8.0 Hz, 1H), 7.41 – 7.35 (m, 2H), 7.22 (d, *J* = 2.6 Hz, 1H), 3.75 (s, 3H), 2.45 (s, 3H).

^{13}C NMR ($CDCl_3$, 101 MHz): δ = 163.5, 148.0, 146.5, 135.6, 134.9, 134.5, 130.7, 128.9, 127.8, 127.6, 127.2, 124.2, 122.5, 120.1, 117.8, 51.7, 21.9.

IR (Diamond-ATR, $CDCl_3$): $\tilde{\upsilon}_{max}$ = 1722 (*m*), 1529 (*s*), 1380 (*m*), 1350 (*m*), 1266 (*w*), 1190 (*w*), 1175 (*s*), 1134 (*m*), 1068 (*m*), 675 (*s*).

HRMS (ESI) calc. for $C_{19}H_{16}N_2NaO_6S$ $[M+Na]^+$: 423.0621; found: 423.0618.

Pyrrole 9f

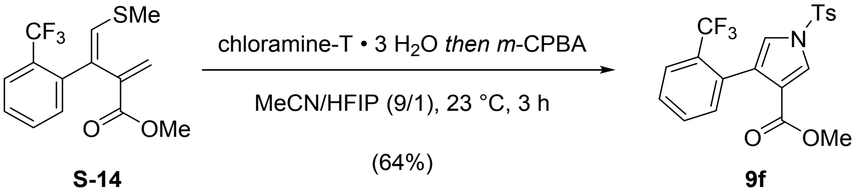

S-14 **9f**

Following GP1 (two hours for pyrrole formation), 1,3-diene **S-14** (61 mg, 0.20 mmol) was converted into pyrrole **9f**. Purification by flash-column chromatography on silica gel (20% diethyl ether in petroleum ether) furnished pyrrole **9f** (55 mg, 64%) as a colorless oil.

TLC (50% diethyl ether in petroleum ether): R_f = 0.37 (UV, $KMnO_4$).

^{1}H NMR ($CDCl_3$, 400 MHz): δ = 7.82 – 7.77 (m, 3H), 7.68 (dd, *J* = 7.8, 1.4 Hz, 1H), 7.54 – 7.48 (m, 1H), 7.44 (tdd, *J* = 7.5, 1.5, 0.8 Hz, 1H), 7.37 – 7.33 (m, 2H), 7.30 (ddd, *J* = 7.5, 1.5, 0.8 Hz, 1H), 7.12 (d, *J* = 2.5 Hz, 1H), 3.61 (s, 3H), 2.45 (s, 3H).

^{13}C NMR ($CDCl_3$, 101 MHz): δ = 163.4, 146.1, 135.3, 132.7, 132.1 (q, J = 2 Hz), 131.0, 130.4, 129.7 (q, J = 31 Hz), 128.0, 127.2, 125.9, 125.8 (q, J = 5 Hz), 125.5, 124.1(q, *J* = 273 Hz), 120.8 (q, J = 2 Hz), 119.9, 51.4, 21.8.

^{19}F NMR ($CDCl_3$, 376 MHz) δ = –58.5.

IR (Diamond-ATR, CH_2Cl_2): $\tilde{\upsilon}_{max}$ = 1729 (*m*), 1379 (*m*), 1315 (*s*), 1259 (*w*), 1190 (*w*), 1174 (*s*), 1129 (*s*), 1091 (*w*), 1063 (*m*), 674 (*m*).

HRMS (ESI) calc. for $C_{20}H_{16}F_3NNaO_4S$ $[M+Na]^+$: 446.0644; found: 446.0631

Pyrrole 9g

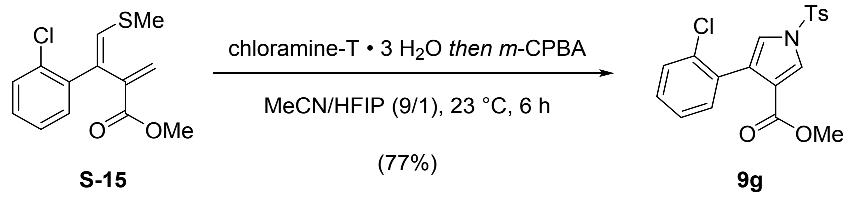

S-15 **9g**

Following GP1 (five hours for pyrrole formation, additional 0.20 equiv of chloramine-T trihydrate after three hours), 1,3-diene **S-15** (54 mg, 0.20 mmol) was converted into pyrrole **9g**. Purification by flash-column chromatography on silica gel (20% ethyl acetate in cyclohexane) furnished pyrrole **9g** (66 mg, 77%) as a colorless oil.

TLC (10% ethyl acetate in *n*-pentane): R_f = 0.20 (UV, $KMnO_4$).

^{1}H NMR (400 MHz, $CDCl_3$): δ = 7.86 – 7.81 (m, 3H), 7.41 (dt, *J* = 6.5, 1.6 Hz, 1H), 7.39 – 7.35 (m, 2H), 7.31 – 7.24 (m, 3H), 7.14 (d, *J* = 2.5 Hz, 1H), 3.68 (s, 3H), 2.45 (s, 3H).

^{13}C NMR (101 MHz, $CDCl_3$): δ = 163.5, 146.0, 135.2, 134.1, 132.2, 131.5, 130.4, 129.2, 129.1, 127.3, 126.5, 126.3, 125.6, 120.4, 119.6, 51.4, 21.7.

IR (Diamond-ATR, $CDCl_3$): $\tilde{\nu}_{max}$ = 1725 (*m*), 1736 (*m*), 1189 (*s*), 1174 (*s*), 1131 (*vs*), 1061 (*s*), 785 (*m*), 674 (*s*), 598 (*s*), 540 (*s*).

HRMS (ESI) calc. for $C_{19}H_{16}ClNNaO_4S$ $[M+Na]^+$: 412.0381; found: 412.0366.

Pyrrole 9h

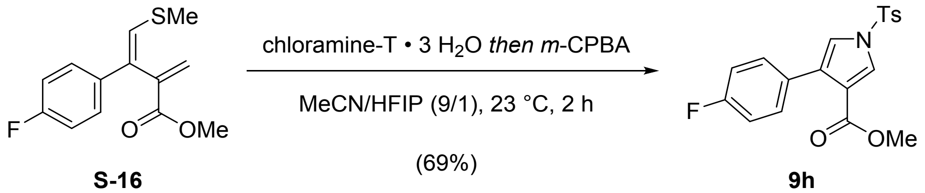

S-16 **9h**

Following GP1 (one hour for pyrrole formation), 1,3-diene **S-16** (51 mg, 0.20 mmol) was converted into pyrrole **9h**. Purification by flash-column chromatography on silica gel (10% to 20% ethyl acetate in cyclohexane) furnished pyrrole **9h** (51 mg, 69%) as a colorless oil.

TLC (40% ethyl acetate in cyclohexane): R_f = 0.53 (UV, $KMnO_4$).

^{1}H NMR ($CDCl_3$, 400 MHz): δ = 7.85 – 7.81 (m, 3H), 7.39 – 7.34 (m, 4H), 7.11 (d, *J* = 2.5 Hz, 1H), 7.06 – 6.99 (m, 2H), 3.74 (s, 3H), 2.44 (s, 3H).

^{13}C NMR ($CDCl_3$, 101 MHz): δ = 163.6, 162.4 (d, *J* = 246 Hz), 146.1, 135.0, 130.9 (d, *J* = 8 Hz), 130.4, 129.0, 128.6 (d, *J* = 4 Hz), 127.4, 126.7, 119.3, 117.9, 114.8 (d, *J* = 22 Hz), 51.4, 21.7.

^{19}F NMR ($CDCl_3$, 376 MHz) δ = –114.8.

IR (Diamond-ATR, $CDCl_3$): $\tilde{\nu}_{max}$ = 1724 (*m*), 1520 (*w*), 1376 (*m*), 1268 (*w*), 1189 (*w*), 1173 (*s*), 1123 (*s*), 1065 (*s*), 675 (*s*), 586 (*s*).

HRMS (ESI) calc. for $C_{19}H_{16}FNNaO_4S$ $[M+Na]^+$: 396.0676; found: 396.0669.

Pyrrole 9i

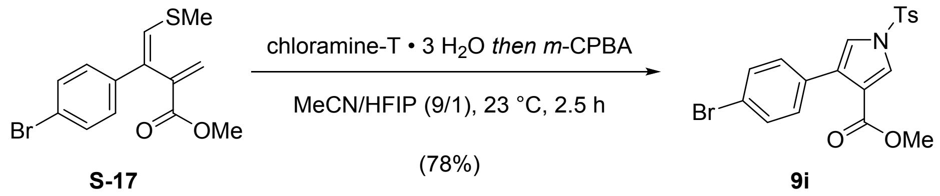

Following GP1 (1.5 hours for pyrrole formation), 1,3-diene **S-17** (63 mg, 0.20 mmol) was converted into pyrrole **9i**. Purification by flash-column chromatography on silica gel (10% to 20% ethyl acetate in *n*-pentane) furnished pyrrole **9i** (68 mg, 78%) as a colorless oil.

TLC (25% ethyl acetate in petroleum ether): R_f = 0.44 (UV, $KMnO_4$).

^{1}H NMR ($CDCl_3$, 400 MHz): δ = 7.84 – 7.82 (m, 3H), 7.47 – 7.45 (m, 2H), 7.36 – 7.34 (m, 2H), 7.29 – 7.27 (m, 2H), 7.14 (d, *J* = 2.5 Hz, 1H), 3.74 (s, 3H), 2.43 (s, 3H).

^{13}C NMR ($CDCl_3$, 101 MHz): δ = 163.7, 146.3, 135.0, 131.7, 131.1, 130.9, 130.5, 128.9, 127.5, 126.9, 121.9, 119.5, 117.9, 51.6, 21.8.

IR (Diamond-ATR, CH_2Cl_2): $\tilde{\upsilon}_{max}$ = 1724 (*m*), 1514 (*w*), 1377 (*m*), 1272 (*w*), 1189 (*w*), 1173 (*s*), 1124 (*m*), 1090 (*w*), 1066 (*s*), 673 (*s*).

HRMS (ESI) calc. for $C_{19}H_{16}BrNNaO_4S$ $[M+Na]^+$: 455.9876; found: 455.9872.

Pyrrole 9j

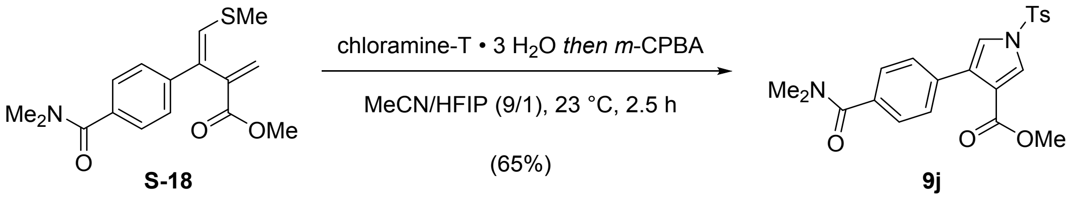

Following GP1 (1.5 hours for pyrrole formation), 1,3-diene **S-18** (61 mg, 0.20 mmol) was converted into pyrrole **9j**. Purification by flash-column chromatography on silica gel (40% diethyl ether in petroleum ether) furnished pyrrole **9j** (55 mg, 65%) as a pale-yellow oil.

TLC (50% methyl *tert*-butyl ether in dichloromethane): R_f = 0.30 (UV, $KMnO_4$, CAM).

^{1}H NMR ($CDCl_3$, 400 MHz): δ = 7.86 – 7.81 (m, 3H), 7.46 – 7.43 (m, 2H), 7.41 – 7.38 (m, 2H) 7.35 (d, *J* = 8.1 Hz, 2H), 7.15 (d, *J* = 2.5 Hz, 1H), 3.74 (s, 3H), 3.11 (s, 3H), 3.01 (s, 3H), 2.43 (s, 3H).

^{13}C NMR ($CDCl_3$, 101 MHz): δ = 171.6, 163.8, 146.3, 135.5, 135.1, 134.1, 130.6, 129.3, 129.2, 127.5, 127.1, 126.9, 119.8, 118.0, 51.6, 39.8, 35.5, 21.9.

IR (Diamond-ATR, $CDCl_3$): $\tilde{\upsilon}_{max}$ = 1724 (*m*), 1627 (*m*), 1378 (*m*), 1265 (*m*), 1189 (*w*), 1174 (*s*), 1124 (*m*), 1066 (*s*), 676 (*s*), 595 (*m*).

HRMS (ESI) calc. for $C_{22}H_{22}N_2NaO_5S$ $[M+Na]^+$: 449.1142; found: 449.1140.

Pyrrole 9k

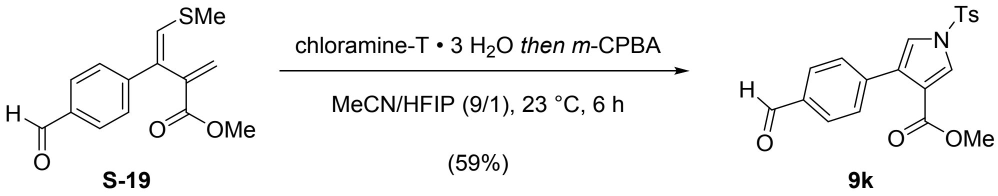

Following GP1 (five hours for pyrrole formation), 1,3-diene **S-19** (53 mg, 0.20 mmol) was converted into pyrrole **9k**. Purification by flash-column chromatography on silica gel (20% ethyl acetate in cyclohexane) furnished pyrrole **9k** (45 mg, 59%) as a colorless oil.

TLC (40% ethyl acetate in cyclohexane): R_f = 0.38 (UV, $KMnO_4$).

^{1}H NMR ($CDCl_3$, 400 MHz): δ = 10.01 (s, 1H), 7.87 – 7.83 (m, 5H), 7.59 – 7.55 (m, 2H), 7.39 – 7.34 (m, 2H), 7.22 (d, *J* = 2.6 Hz, 1H), 3.75 (s, 3H), 2.44 (s, 3H).

^{13}C NMR ($CDCl_3$, 101 MHz): δ = 192.0, 163.6, 146.4, 139.1, 135.5, 134.9, 130.6, 129.9, 129.5, 128.8, 127.6, 127.2, 120.1, 117.9, 51.7, 21.9.

IR (Diamond-ATR, CH_2Cl_2): $\tilde{\upsilon}_{max}$ = 1724 (*m*), 1700 (*m*), 1606 (*w*), 1379 (*m*), 1271 (*w*), 1190 (*w*), 1175 (*s*), 1126 (*m*), 1067 (*m*), 672 (*s*).

HRMS (ESI) calc. for $C_{20}H_{17}NNaO_5S$ $[M+Na]^+$: 406.0720; found: 406.0715.

Pyrrole 9l

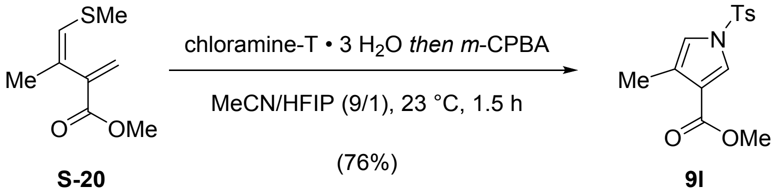

Following GP1 (30 minutes for pyrrole formation), 1,3-diene **S-20** (34 mg, 0.20 mmol) was converted into pyrrole **9l**. Purification by flash-column chromatography on silica gel (20% diethyl ether in petroleum ether) furnished pyrrole **9l** (44 mg, 76%) as a colorless solid.

TLC (20% diethyl ether in petroleum ether): R_f = 0.23 (UV, $KMnO_4$, CAM).

^{1}H NMR ($CDCl_3$, 400 MHz): δ = 7.79 – 7.74 (m, 2H), 7.70 (d, *J* = 2.5 Hz, 1H), 7.34 – 7.28 (m, 2H), 6.87 (dq, *J* = 2.4, 1.2 Hz, 1H), 3.78 (s, 3H), 2.41 (s, 3H), 2.19 (d, *J* = 1.2 Hz, 3H).

^{13}C NMR ($CDCl_3$, 101 MHz): δ = 164.5, 145.7, 135.5, 130.3, 127.3, 126.1, 125.2, 119.5, 118.9, 51.4, 21.8, 11.7.

IR (Diamond-ATR, $CDCl_3$): $\tilde{\upsilon}_{max}$ = 1715 (*m*), 1373 (*m*), 1229 (*m*), 1189 (*w*), 1170 (*m*), 1088 (*s*), 1059 (*s*), 774 (*w*), 666 (*s*), 582 (*s*).

HRMS (ESI) calc. for $C_{14}H_{16}NO_4S$ $[M+H]^+$: 294.0795; found: 294.0790.

MP: 79 – 80 °C.

Pyrrole 9m

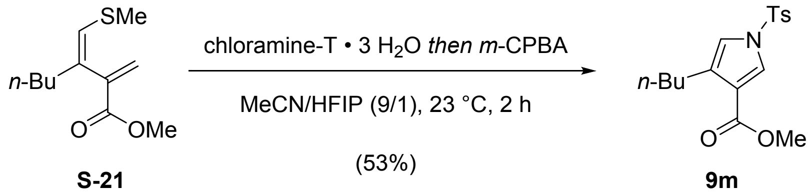

S-21 **9m**

Following GP1 (one hour for pyrrole formation), 1,3-diene **S-21** (43 mg, 0.20 mmol) was converted into pyrrole **9m**. Purification by flash-column chromatography on silica gel (20% diethyl ether in petroleum ether) furnished pyrrole **9m** (36 mg, 53%) as a colorless oil.

TLC (20% diethyl ether in petroleum ether): R_f = 0.31 (UV, $KMnO_4$, CAM).

^{1}H NMR ($CDCl_3$, 400 MHz): δ = 7.79 – 7.75 (m, 2H), 7.70 (d, *J* = 2.5 Hz, 1H), 7.34 – 7.29 (m, 2H), 6.88 (dt, *J* = 2.4, 1.1 Hz, 1H), 3.78 (s, 3H), 2.62 (ddd, *J* = 9.0, 6.6, 1.1 Hz, 2H), 2.42 (s, 3H), 1.55 – 1.46 (m, 2H), 1.33 (m, 2H), 0.90 (t, *J* = 7.3 Hz, 3H).

^{13}C NMR ($CDCl_3$, 101 MHz): δ = 164.4, 145.7, 135.6, 130.4, 130.3, 127.3, 126.3, 119.0, 118.3, 51.4, 31.7, 25.8, 22.6, 21.8, 14.1.

IR (Diamond-ATR, $CDCl_3$): $\tilde{\upsilon}_{max}$ = 1719 (*m*), 1375 (*m*), 1231 (*w*), 1211 (*w*), 1189 (*m*), 1172 (*s*), 1088 (*s*), 1061 (*s*), 670 (*s*), 587 (*s*).

HRMS (ESI) calc. for $C_{17}H_{22}NO_4S$ $[M+H]^+$: 336.1264; found: 336.1259.

Pyrrole 9n

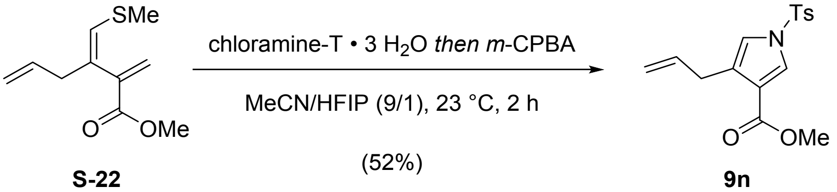

S-22 **9n**

Following GP1 (one hour for pyrrole formation), 1,3-diene **S-22** (40 mg, 0.20 mmol) was converted into pyrrole **9n**. Purification by flash-column chromatography on silica gel (10% diethyl ether in *n*-pentane) furnished pyrrole **9n** (33 mg, 52%) as a colorless oil.

TLC (20% diethyl ether in *n*-pentane): R_f = 0.48 (UV, $KMnO_4$, CAM).

^{1}H NMR ($CDCl_3$, 400 MHz): δ = 7.80 – 7.76 (m, 2H), 7.73 (d, *J* = 2.5 Hz, 1H), 7.35 – 7.30 (m, 2H), 6.88 (dt, *J* = 2.5, 1.1 Hz, 1H), 5.93 (ddt, *J* = 17.6, 9.6, 6.6 Hz, 1H), 5.08 – 5.04 (m, 1H), 5.03 (dt, *J* = 2.5, 1.1 Hz, 1H), 3.78 (s, 3H), 3.41 (dq, *J* = 6.6, 1.3 Hz, 2H), 2.41 (s, 3H).

^{13}C NMR ($CDCl_3$, 101 MHz): δ = 164.2, 145.8, 136.0, 135.5, 130.4, 128.0, 127.3, 126.4, 118.9, 118.8, 116.3, 51.4, 30.5, 21.8.

IR (Diamond-ATR, $CDCl_3$): $\tilde{\upsilon}_{max}$ = 1718 (*m*), 1509 (*w*), 1375 (*m*), 1212 (*w*), 1188 (*w*), 1173 (*s*), 1085 (*s*), 1060 (*s*), 672 (*s*), 576 (*s*).

HRMS (ESI) calc. for $C_{16}H_{17}NNaO_4S$ $[M+Na]^+$: 342.0770; found: 342.0766.

Pyrrole 9o

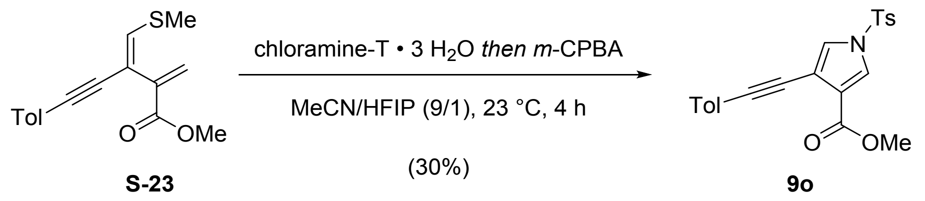

Following GP1 (three hours for pyrrole formation), 1,3-diene **S-23** (55 mg, 0.20 mmol) was converted into pyrrole **9o**. Purification by flash-column chromatography on silica gel (20% diethyl ether in petroleum ether) furnished pyrrole **9o** (24 mg, 30%) as a yellow oil.

TLC (20% methyl *tert*-butyl ether in *n*-pentane): R_f = 0.20 (UV, $KMnO_4$, CAM).

^{1}H NMR ($CDCl_3$, 400 MHz): δ = 7.83 – 7.78 (m, 2H), 7.73 (d, *J* = 2.5 Hz, 1H), 7.41 – 7.37 (m, 2H), 7.37 – 7.32 (m, 3H), 7.13 (d, *J* = 7.7 Hz, 2H), 3.85 (s, 3H), 2.43 (s, 3H), 2.35 (s, 3H).

^{13}C NMR ($CDCl_3$, 101 MHz): δ = 163.1, 146.4, 138.7, 134.9, 131.6, 130.6, 129.2, 127.5, 125.7, 124.6, 120.7, 120.2, 110.1, 92.9, 80.2, 51.8, 21.9, 21.7.

IR (Diamond-ATR, CH_2Cl_2): $\tilde{\upsilon}_{max}$ = 1729 (*w*), 1382 (*w*), 1190 (*m*), 1175 (*s*), 1085 (*w*), 1062 (*m*), 816 (*w*), 673 (*m*), 593 (*w*), 562 (*w*).

HRMS (ESI) calc. for $C_{22}H_{20}NO_4S$ $[M+H]^+$: 394.1108; found: 394.1106.

Pyrrole 9p

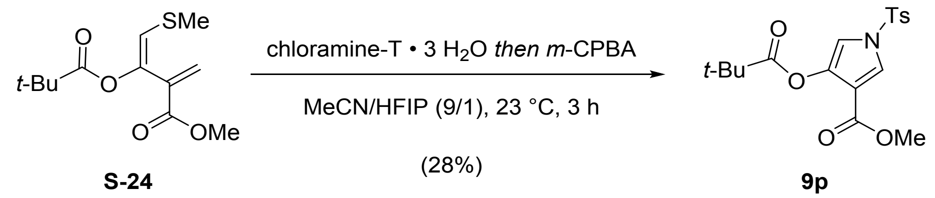

Following GP1 (two hours for pyrrole formation), 1,3-diene **S-24** (52 mg, 0.20 mmol) was converted into pyrrole **9p**. Purification by flash-column chromatography on silica gel (20% ethyl acetate in cyclohexane) followed by HPLC purification (5% grading to 15% ethyl acetate in *n*-hexane within 30 minutes) furnished pyrrole **9p** (21 mg, 28%) as a pale-yellow oil.

TLC (20% ethyl acetate in *n*-pentane): R_f = 0.28 (UV, $KMnO_4$).

^{1}H NMR (400 MHz, $CDCl_3$): δ = 7.82 – 7.73 (m, 2H), 7.68 (d, *J* = 2.7 Hz, 1H), 7.37 – 7.29 (m, 2H), 7.21 (d, *J* = 2.7 Hz, 1H), 3.78 (s, 3H), 2.42 (s, 3H), 1.32 (s, 9H).

^{13}C NMR (101 MHz, $CDCl_3$): δ = 175.7, 162.3, 146.1, 138.3, 134.9, 130.4, 127.4, 123.8, 113.1, 111.1, 51.4, 39.2, 27.1, 21.7.

IR (Diamond-ATR, $CDCl_3$): $\tilde{\upsilon}_{max}$ = 1758 (*m*), 1728 (*s*), 1381 (*m*), 1250 (*s*), 1190 (*s*), 1176 (*s*), 1097 (*vs*), 1058 (*s*), 671 (*vs*), 587 (*s*).

HRMS (ESI) calc. for $C_{18}H_{21}NNaO_6S$ $[M+Na]^+$: 402.0982; found: 402.0974.

Pyrrole 9q

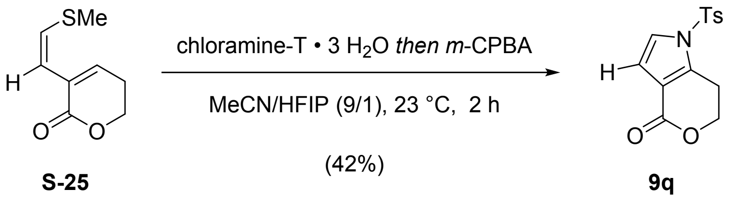

Following GP1 (one hours for pyrrole formation), 1,3-diene **S-25** (34 mg, 0.20 mmol) was converted into pyrrole **9q**. Purification by flash-column chromatography on silica gel (40% ethyl acetate in *n*-pentane) furnished pyrrole **9q** (24 mg, 42%) as a yellow solid.

TLC (40% ethyl acetate in *n*-pentane): R_f = 0.46 (UV, $KMnO_4$).

^{1}H NMR (400 MHz, $CDCl_3$): δ = 7.79 – 7.71 (m, 2H), 7.41 – 7.33 (m, 2H), 7.23 (d, *J* = 3.5 Hz, 1H), 6.67 (d, *J* = 3.5 Hz, 1H), 4.49 (t, *J* = 6.3 Hz, 2H), 3.14 (t, *J* = 6.2 Hz, 2H), 2.45 (s, 3H).

^{13}C NMR (101 MHz, $CDCl_3$): δ = 162.5, 146.4, 138.3, 135.0, 130.6, 127.1, 122.4, 115.4, 110.7, 66.8, 22.8, 21.8.

IR (Diamond-ATR, $CDCl_3$): $\tilde{\upsilon}_{max}$ = 1727(*vs*), 1447 (*w*), 1379 (*m*), 1190 (*m*), 1179 (*s*), 1128 (*s*) 1111 (*s*), 669 (*m*), 599 (*m*), 541 (*m*).

HRMS (ESI) calc. for $C_{14}H_{14}NO_4S$ $[M+H]^+$: 292.0638; found: 292.0634.

MP: 130 – 132 °C.

Pyrrole 12a

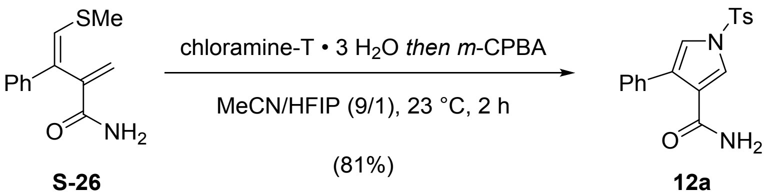

Following GP1 (one hour for pyrrole formation), 1,3-diene **S-26** (44 mg, 0.20 mmol) was converted into pyrrole **12a**. Purification by flash-column chromatography on silica gel (15% ethyl acetate in dichloromethane) furnished pyrrole **12a** (55 mg, 81%) as a colorless foam.

TLC (20% ethyl acetate in cyclohexane): R_f = 0.25 (UV, $KMnO_4$, CAM).

^{1}H NMR ($CDCl_3$, 400 MHz): δ = 7.84 – 7.78 (m, 3H), 7.42 – 7.29 (m, 7H), 7.11 (d, *J* = 2.6 Hz, 1H), 6.22 (br, 1H), 5.50 (br, 1H), 2.41 (s, 3H).

^{13}C NMR ($CDCl_3$, 101 MHz): δ = 165.4, 146.0, 135.2, 132.5, 130.4, 129.3, 129.0, 128.4, 127.6, 127.5, 125.3, 122.0, 119.3, 21.8.

IR (Diamond-ATR, CH_2Cl_2): $\tilde{\upsilon}_{max}$ = 1657 (*m*), 1596 (*w*), 1375 (*m*), 1307 (*w*), 1190 (*w*), 1173 (*s*), 1090 (*w*), 1066 (*m*), 701 (*w*), 672 (*s*).

HRMS (ESI) calc. for $C_{18}H_{17}N_2O_3S$ $[M+H]^+$: 341.0954; found: 341.0955.

Pyrrole 12b

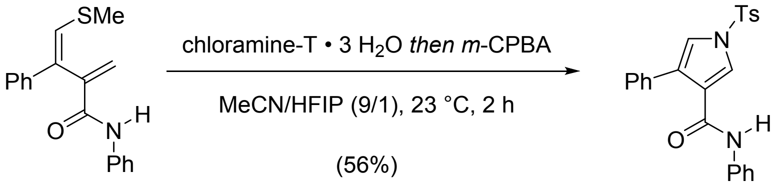

S-27 **12b**

Following GP1 (one hour for pyrrole formation), 1,3-diene **S-27** (45 mg, 0.20 mmol) was converted into pyrrole **12b**. Purification by flash-column chromatography on silica gel (20% ethyl acetate in cyclohexane) furnished pyrrole **12b** (47 mg, 56%) as an orange, crystalline solid. Recrystallization from dichloromethane and *n*-heptane gave crystals suitable for X-ray analysis.

TLC (20% ethyl acetate in cyclohexane): R_f = 0.26 (UV, $KMnO_4$, CAM).

^{1}H NMR ($CDCl_3$, 400 MHz): δ = 7.90 (d, *J* = 2.6 Hz, 1H), 7.88 – 7.83 (m, 2H), 7.48 – 7.43 (m, 5H), 7.35 (d, *J* = 8.1 Hz, 2H), 7.24 – 7.16 (m, 6H), 7.06 – 7.01 (m, 1H), 2.44 (s, 3H).

^{13}C NMR ($CDCl_3$, 101 MHz): δ = 161.1, 146.1, 137.8, 135.2, 132.4, 130.5, 129.8, 129.3, 129.1, 128.8, 127.6, 126.9, 125.4, 124.3, 123.1, 119.6, 119.4, 21.9.

IR (Diamond-ATR, CH_2Cl_2): $\tilde{\upsilon}_{max}$ = 1656 (*w*), 1597 (*w*), 1533 (*m*), 1440 (*w*), 1376 (*w*), 1174 (*s*), 1066 (*w*), 758 (*w*), 693 (*w*), 671 (*s*).

HRMS (ESI) calc. for $C_{24}H_{21}N_2O_3S$ $[M+H]^+$: 417.1267; found: 417.1268.

MP: 113 – 114 °C.

Pyrrole 12c

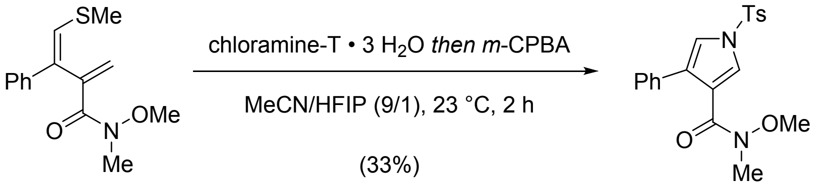

S-28 **12c**

Following GP1 (one hour for pyrrole formation), 1,3-diene **S-28** (53 mg, 0.20 mmol) was converted into pyrrole **12c**. Purification by flash-column chromatography on silica gel (20% to 40% ethyl acetate in cyclohexane) furnished pyrrole **12c** (25 mg, 33%) as a pale-yellow oil.

TLC (40% ethyl acetate in cyclohexane): R_f = 0.26 (UV, $KMnO_4$).

^{1}H NMR ($CDCl_3$, 400 MHz): δ = 7.83 – 7.77 (m, 3H), 7.53 (d, *J* = 2.5 Hz, 1H), 7.36 – 7.27 (m, 6H), 7.22 (d, *J* = 2.5 Hz, 1H), 3.47 (s, 3H), 3.13 (s, 3H), 2.42 (s, 3H).

^{13}C NMR ($CDCl_3$, 101 MHz): δ = 164.8, 145.9, 135.4, 133.2, 130.4, 129.8, 128.6, 127.6, 127.5, 127.4, 122.3, 120.8, 117.8, 61.0, 33.8, 21.8.

IR (Diamond-ATR, CH_2Cl_2): $\tilde{\upsilon}_{max}$ = 1646 (*m*), 1524 (*w*), 1375 (*m*), 1189 (*w*), 1173 (*s*), 1087 (*m*), 1063 (*m*), 701 (*w*), 672 (*m*), 597 (*m*).

HRMS (ESI) calc. for $C_{20}H_{20}N_2NaO_4S$ $[M+Na]^+$: 407.1036; found: 446.1029.

Pyrrole 13a

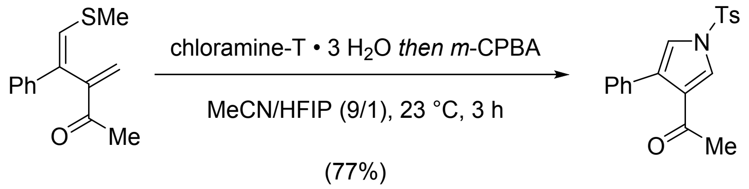

S-29 **13a**

Following GP1 (two hours for pyrrole formation), 1,3-diene **S-29** (44 mg, 0.20 mmol) was converted into pyrrole **13a**. Purification by flash-column chromatography on silica gel (20% ethyl acetate in cyclohexane) furnished pyrrole **13a** (52 mg, 77%) as a pale-yellow oil.

TLC (20% ethyl acetate in cyclohexane): R_f = 0.23 (UV, $KMnO_4$).

^{1}H NMR ($CDCl_3$, 400 MHz): δ = 7.86 – 7.82 (m, 2H), 7.77 (d, *J* = 2.4 Hz, 1H), 7.38 – 7.28 (m, 7H), 7.12 (d, *J* = 2.5 Hz, 1H), 2.44 (s, 3H), 2.33 (s, 3H).

^{13}C NMR ($CDCl_3$, 101 MHz): δ = 193.6, 146.2, 135.1, 133.1, 130.5, 129.6, 129.2, 128.2, 127.7, 127.5, 127.1, 126.2, 120.0, 28.9, 21.8.

IR (Diamond-ATR, CH_2Cl_2): $\tilde{\upsilon}_{max}$ = 1679 (*m*), 1507 (*w*), 1376 (*m*), 1189 (*w*), 1173 (*s*), 1105 (*m*), 1070 (*m*), 702 (*w*), 671 (*s*), 601 (*m*).

HRMS (ESI) calc. for $C_{19}H_{17}NNaO_3S$ $[M+Na]^+$: 362.0821; found: 362.0816.

Pyrrole 13b

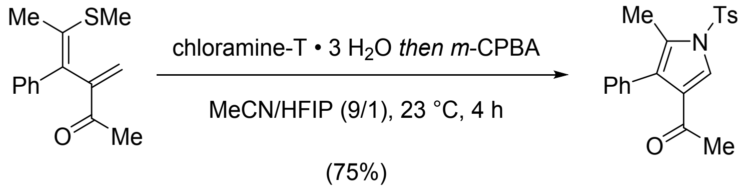

S-30 **13b**

Following GP1 (three hours for pyrrole formation), 1,3-diene **S-30** (43 mg, 0.20 mmol) was converted into pyrrole **13b**. Purification by flash-column chromatography on silica gel (10% ethyl acetate in cyclohexane) furnished pyrrole **13b** (49 mg, 75%) as a colorless oil.

TLC (20% ethyl acetate in cyclohexane): R_f = 0.23 (UV, $KMnO_4$, CAM).

^{1}H NMR ($CDCl_3$, 400 MHz): δ = 7.97 (s, 1H), 7.80 – 7.76 (m, 2H), 7.40 – 7.27 (m, 5H), 7.17 – 7.12 (m, 2H), 2.46 (s, 3H), 2.24 (s, 3H), 2.13 (s, 3H).

^{13}C NMR ($CDCl_3$, 101 MHz): δ = 193.6, 146.0, 135.4, 133.8, 130.5, 130.4, 129.1, 128.1, 127.7, 127.4, 126.4, 126.4, 126.0, 28.8, 21.9, 11.4.

IR (Diamond-ATR, CH_2Cl_2): $\tilde{\upsilon}_{max}$ = 1676 (*m*), 1516 (*w*), 1370 (*m*), 1191 (*w*), 1170 (*s*), 1092 (*m*), 781 (*w*), 701 (*m*), 669 (*s*), 595 (*s*).

HRMS (ESI) calc. for $C_{20}H_{20}NO_3S$ $[M+H]^+$: 354.1158; found: 354.1159.

Pyrrole 13c

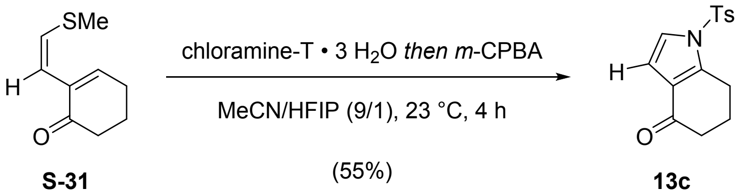

S-31 **13c**

Following GP1 (three hours for pyrrole formation), 1,3-diene **S-31** (34 mg, 0.20 mmol) was converted into pyrrole **13c**. Purification by flash-column chromatography on silica gel (5% to 10% ethyl acetate in dichloromethane) furnished pyrrole **13c** (32 mg, 55%) as an amorphous, colorless solid.

TLC (10% ethyl acetate in dichloromethane): R_f = 0.74 (UV, CAM).

^{1}H NMR ($CDCl_3$, 400 MHz): δ = 7.77 – 7.72 (m, 2H), 7.37 – 7.32 (m, 2H), 7.23 (d, *J* = 3.5 Hz, 1H), 6.61 (d, *J* = 3.5 Hz, 1H), 2.96 (t, *J* = 6.2 Hz, 2H), 2.43 (s, 3H), 2.41 (m, 2H), 2.09 (p, *J* = 6.3 Hz, 2H).

^{13}C NMR ($CDCl_3$, 101 MHz): δ = 194.4, 146.0, 143.8, 135.4, 130.5, 127.3, 124.9, 122.5, 108.3, 37.5, 23.4, 23.0, 21.8.

IR (Diamond-ATR, $CDCl_3$): $\tilde{\upsilon}_{max}$ = 1672 (*s*), 1442 (*m*), 1376 (*m*), 1189 (*m*), 1176 (*s*), 1119 (*s*), 1109 (*s*), 678 (*m*), 591 (*m*), 580 (*m*).

HRMS (ESI) calc. for $C_{15}H_{16}NO_3S$ $[M+H]^+$: 290.0845; found: 290.0837.

Pyrroles 14a and S-33

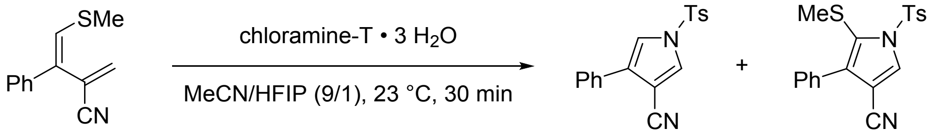

S-32 **14a** (51%) **S-33** (5%)

Following GP1 (30 minutes reaction time, without the addition of 3-chloroperbenzoic acid), 1,3-diene **S-32** (40 mg, 0.20 mmol) was converted into pyrroles **14a** and **S-33**. Purification by flash-column chromatography on silica gel (10% ethyl acetate in toluene) followed by HPLC purification (10% grading to 20% ethyl acetate in *n*-hexane within 30 minutes) furnished pyrroles **14a** (36 mg, 51%) and **S-33** (3.7 mg, 5%) as colorless oils.

TLC (25% ethyl acetate in *n*-hexane): R_f = 0.38 (UV, $KMnO_4$, CAM).

Major product **14a**:

^{1}H NMR (400 MHz, $CDCl_3$): δ = 7.86 – 7.81 (m, 2H), 7.72 (d, *J* = 2.4 Hz, 1H), 7.59 – 7.54 (m, 2H), 7.43 – 7.33 (m, 5H), 7.32 (d, *J* = 2.5 Hz, 1H), 2.45 (s, 3H).

^{13}C NMR (101 MHz, $CDCl_3$): δ = 146.8, 134.6, 130.8, 130.7, 130.1, 129.2, 128.7, 128.5, 127.6, 126.9, 117.7, 114.6, 97.6, 21.9.

IR (Diamond-ATR, $CDCl_3$): $\tilde{\upsilon}_{max}$ = 1515 (*w*), 1382 (*m*), 1191 (*m*), 1175 (*s*), 1068 (*s*), 707 (*w*), 671 (*s*), 605 (*w*), 571 (*s*), 540 (*w*).

HRMS (ESI) calc. for $C_{18}H_{14}N_2NaO_2S$ $[M+Na]^+$: 345.0668; found: 345.0663.

Minor product **S-33**:

^{1}H NMR (400 MHz, $CDCl_3$): δ = 8.09 (s, 1H), 7.97 – 7.92 (m, 2H), 7.50 – 7.46 (m, 2H), 7.45 – 7.35 (m, 5H), 2.46 (s, 3H), 2.10 (s, 3H).

^{13}C NMR (101 MHz, $CDCl_3$): δ = 146.6, 136.7, 134.4, 131.5, 130.7, 130.2, 129.3, 128.9, 128.7, 128.7, 123.6, 114.2, 97.7, 22.0, 21.3.

IR (Diamond-ATR, $CDCl_3$): $\tilde{\upsilon}_{max}$ = 1380 (*m*), 1303 (*w*), 1191 (*m*), 1178 (*s*), 1104 (*m*), 1090 (*m*), 699 (*w*), 676 (*m*), 666 (*m*), 576 (*vs*).

HRMS (ESI) calc. for $C_{19}H_{16}N_2NaO_2S_2$ $[M+Na]^+$: 391.0545; found: 391.0542.

Pyrroles 14b and S-35

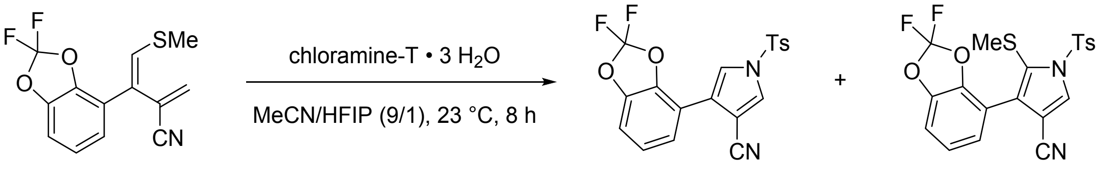

S-34 **14b** (42%) **S-35** (14%)

Following GP1 (eight hours reaction time, without the addition of 3-chloroperbenzoic acid), 1,3-diene **S-34** (56 mg, 0.20 mmol, 1 equiv) was converted into pyrroles **14b** and **S-35**. Purification by flash-column chromatography on silica gel (5% to 15% ethyl acetate in petroleum ether) followed by HPLC purification (10% grading to 20% ethyl acetate in *n*-hexane within 30 minutes) furnished pyrroles **14b** (34 mg, 42%) as a colorless solid and **S-35** (13 mg, 14%) as a colorless oil. Recrystallization of **14b** from dichloromethane, ethyl acetate and *n*-hexane gave crystals suitable for X-ray analysis.

TLC (10% ethyl acetate in *n*-hexane): R_f = 0.21 (UV, $KMnO_4$, CAM).

Major product **14b**:

^{1}H NMR (400 MHz, $CDCl_3$): δ = 7.89 – 7.83 (m, 2H), 7.75 (d, *J* = 2.4 Hz, 1H), 7.62 – 7.57 (m, 2H), 7.42 – 7.36 (m, 2H), 7.14 (dd, *J* = 8.1, 8.1 Hz, 1H), 7.03 (dd, *J* = 8.0, 1.1 Hz, 1H), 2.46 (s, 3H).

^{13}C NMR (101 MHz, $CDCl_3$): δ = 147.1, 144.0, 140.3, 134.3, 134.1, 131.5 (t, *J* = 256 Hz), 129.0, 127.7, 124.4, 121.7, 121.4, 120.7, 114.5, 114.3, 109.3, 97.1, 21.9.

^{19}F NMR (376 MHz, $CDCl_3$): δ = –49.4.

IR (Diamond-ATR, $CDCl_3$): $\tilde{\upsilon}_{max}$ = 1457 (*w*), 1387 (*w*), 1248 (*s*), 1191 (*m*), 1176 (*s*), 1072 (*s*), 1030 (*w*), 676 (*s*), 587 (*m*), 570 (*m*).

HRMS (ESI) calc. for $C_{19}H_{12}F_2N_2NaO_4S$ [M+Na]$^+$: 425.0378; found: 425.0371.

MP = 132 – 134 °C.

Minor product **S-35**:

^{1}H NMR (400 MHz, $CDCl_3$): δ = 8.12 (s, 1H), 7.99 – 7.93 (m, 2H), 7.38 (d, *J* = 8.1 Hz, 2H), 7.17 – 7.07 (m, 2H), 7.04 (dd, *J* = 7.5, 1.7 Hz, 1H), 2.47 (s, 3H), 2.27 (s, 3H).

^{13}C NMR (101 MHz, $CDCl_3$): δ = 146.9, 144.1, 141.6, 134.0, 131.9, 131.5 (t, *J* = 256 Hz), 130.3, 129.9, 128.9, 125.7, 125.3, 123.8, 114.1, 113.3, 110.0, 98.0, 22.0, 21.3.

^{19}F NMR (376 MHz, $CDCl_3$): δ = –49.8.

IR (Diamond-ATR, $CDCl_3$): $\tilde{\upsilon}_{max}$ = 1452 (*w*), 1384 (*w*), 1250 (*vs*), 1192 (*m*), 1177 (*s*), 1133 (*w*), 1090 (*w*), 1035 (*m*), 670 (*m*), 576 (*s*).

HRMS (ESI) calc. for $C_{20}H_{14}F_2N_2NaO_4S_2$ [M+Na]$^+$: 471.0255; found: 471.0254.

Fludioxonil 15

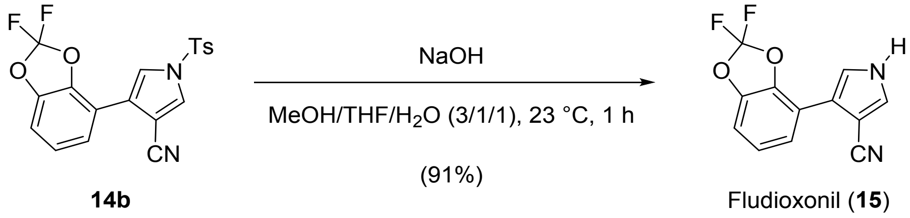

To a solution of pyrrole **14b** (8.0 mg, 0.20 mmol, 1 equiv) in methanol (0.3 mL) and tetrahydrofuran (0.1 mL) was added an aqueous solution of sodium hydroxide (6.0 M, 0.10 mL, 0.60 mmol, 30 equiv). After one hour, ethyl acetate (5 mL) and saturated aqueous ammonium chloride solution (5 mL) were added and the layers were separated. The aqueous layer was extracted with ethyl acetate (3 × 5 mL), the combined organic layers were dried over sodium sulfate, the dried solution was filtered and the filtrate was concentrated. The crude product was purified by flash-column chromatography on silica gel (15% to 25% ethyl acetate in petroleum ether) to furnish Fludioxonil (**15**, 4.5 mg, 91%) as a colorless solid. The obtained analytical data were in full agreement with those of commercial **15** purchased from *Sigma-Aldrich*.

TLC (25% ethyl acetate in petroleum ether): R_f = 0.32 (UV, $KMnO_4$).

Pyrrole 16

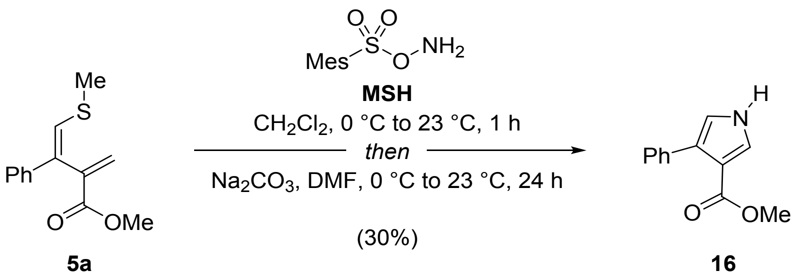

To a solution of 1,3-diene **5a** (47 mg, 0.20 mmol, 1 equiv) in dichloromethane (2.0 mL) was added dropwise a freshly prepared solution of *O*-mesitylenesulfonyl hydroxylamine (**MSH**, 0.08 M in dichloromethane, 2.5 mL, 0.20 mmol, 1.0 equiv) at 0 °C. Upon complete addition, the reaction mixture was allowed to warm to 23 °C. After 30 minutes, additional **MSH** solution (0.08 M in dichloromethane, 0.50 mL, 50 µmol, 0.20 equiv) was added. After 15 minutes, the solvent was removed under reduced pressure, the residue was redissolved in *N*,*N*-dimethylformamide (2.0 mL) and the solution was cooled to 0 °C. After 15 minutes, sodium carbonate (32 mg, 0.30 mmol, 1.5 equiv) was added and the reaction mixture was allowed to warm to 23 °C. After 24 hours, ethyl acetate (5 mL) and saturated aqueous ammonium chloride solution (5 mL) were added and the layers were separated. The aqueous layer was extracted with ethyl acetate (3 × 5 mL), the combined organic layers were dried over sodium sulfate, the dried solution was filtered and the filtrate was concentrated under reduced pressure. The crude product was purified by flash-column chromatography on silica gel (20% ethyl acetate in *n*-pentane) to furnish pyrrole **16** (15 mg, 30%) as a pale-yellow solid. The obtained analytical data were in full agreement with those reported in literature.[5]

TLC (20% ethyl acetate in cyclohexane): R_f = 0.15 (UV, $KMnO_4$, CAM).

Pyrrole 16

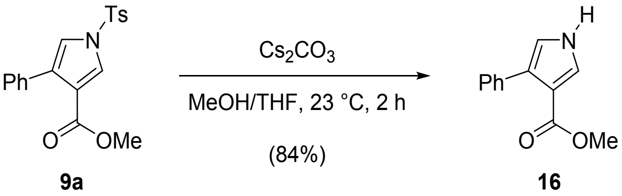

To a solution of pyrrole **9a** (50.0 mg, 140 µmol, 1 equiv) in tetrahydrofuran (2.4 mL) and methanol (1.2 mL) was added cesium carbonate (140 mg, 420 µmol, 3.00 equiv) in one portion at 23 °C. After two hours, aqueous hydrochloric acid solution (1 M, 20 mL) and dichloromethane (25 mL) were added. The layers were separated and the aqueous phase was extracted with dichloromethane (2 × 25 mL). The combined organic layers were dried over sodium sulfate, the dried solution was filtered and the filtrate was concentrated under reduced pressure. The crude product was purified by flash-column chromatography on silica gel (20%

to 40% diethyl ether in *n*-pentane) to yield pyrrole **16** (23.8 mg, 84%) as a colorless solid. The obtained analytical data were in full agreement with those reported in literature.[5]

Pyrrole 17a

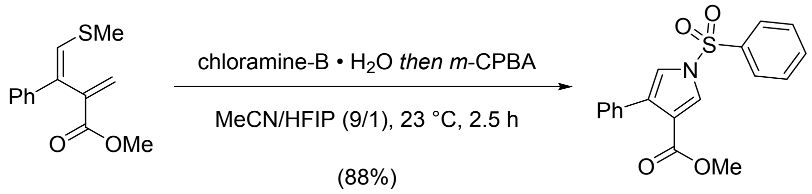

5a **17a**

To a solution of 1,3-diene **5a** (47 mg, 0.20 mmol, 1 equiv) in acetonitrile (1.8 mL) and hexafluoroisopropanol (0.2 mL) was added chloramine-B monohydrate (106 mg, 397 µmol, 2.00 equiv) at 23 °C portionwise over a period of ten minutes. After 30 minutes, 3-chloroperoxobenzoic acid (34.2 mg, 198 µmol, 1.00 equiv) was added. After two hours, the reaction mixture was poured into saturated aqueous thiosulfate solution (7 mL), ethyl acetate (7 mL) was added and the layers were separated. The aqueous layer was extracted with ethyl acetate (2 × 5 mL), the combined organic layers were washed with saturated aqueous sodium bicarbonate solution (10 mL). The organic layer was dried over sodium sulfate, the dried solution was filtered, and the filtrate concentrated under reduced pressure. The crude product was purified by flash column chromatography on silica gel (5% to 10% ethyl acetate in *n*-pentane) to furnish pyrrole **17a** (60 mg, 88%) as a colorless, amorphous solid.

TLC (10% ethyl acetate in *n*-pentane): R_f = 0.37 (UV, $KMnO_4$).

^{1}H NMR (400 MHz, $CDCl_3$): δ = 7.99 – 7.92 (m, 2H), 7.85 (d, *J* = 2.6 Hz, 1H), 7.73 – 7.64 (m, 1H), 7.59 – 7.54 (m, 2H), 7.44 – 7.39 (m, 2H), 7.39 – 7.30 (m, 3H), 7.15 (d, *J* = 2.6 Hz, 1H), 3.74 (s, 3H).

^{13}C NMR (101 MHz, $CDCl_3$): δ = 163.8, 138.3, 134.8, 132.6, 130.3, 129.9, 129.3, 128.1, 127.7, 127.4, 126.9, 119.6, 118.4, 51.6.

IR (Diamond-ATR, $CDCl_3$): $\tilde{\upsilon}_{max}$ = 1727 (*m*), 1378 (*m*), 1185 (*s*), 1176 (*s*), 1127 (*s*), 1091 (*m*), 1067 (*s*), 728 (*s*), 616 (*m*), 594 (*m*).

HRMS (ESI) calc. for $C_{18}H_{15}NNaO_4S$ $[M+Na]^+$: 364.0614; found: 364.0617.

Pyrrole 17b

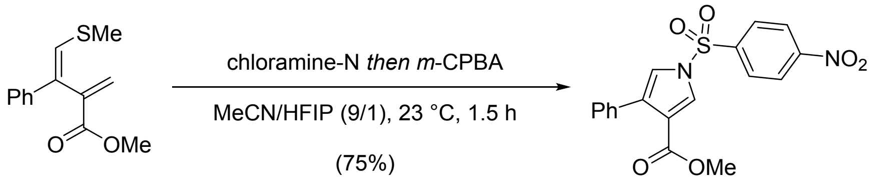

5a **17b**

To a solution of 1,3-diene **5a** (47 mg, 0.20 mmol, 1 equiv) in acetonitrile (1.8 mL) and hexafluoroisopropanol (0.2 mL) was added chloramine-N (131 mg, 405 μmol, 2.00 equiv) at 23°C portionwise over a period of ten minutes. After 30 minutes, 3-chloroperoxobenzoic acid (34.9 mg, 202 μmol, 1.00 equiv) was added. After one hour, saturated aqueous thiosulfate solution (5 mL) and ethyl acetate (5 mL) were added. The layers were separated and the aqueous phase was extracted with ethyl acetate (2 × 5 mL), the combined organic layers were washed with saturated aqueous sodium bicarbonate solution (10 mL) and dried over sodium sulfate. The dried solution was filtered, and the filtrate concentrated under reduced pressure. The crude product was purified by flash column chromatography on silica gel (5% to 10% ethyl acetate in *n*-pentane) to furnish pyrrole **17b** (59 mg, 75%) as a yellow oil.

TLC (5% ethyl acetate in *n*-pentane): R_f = 0.26 (UV, $KMnO_4$).

^{1}H NMR (400 MHz, $CDCl_3$): δ = 8.39 (d, *J* = 8.9 Hz, 2H), 8.14 (d, *J* = 8.9 Hz, 2H), 7.85 (d, *J* = 2.6 Hz, 1H), 7.43 – 7.31 (m, 5H), 7.16 (d, *J* = 2.6 Hz, 1H), 3.75 (s, 3H).

^{13}C NMR (101 MHz, $CDCl_3$): δ = 163.3, 151.2, 143.5, 132.1, 131.3, 129.2, 128.8, 128.1, 128.0, 126.8, 125.1, 119.6, 119.4, 51.7.

IR (Diamond-ATR, $CDCl_3$): $\tilde{\upsilon}_{max}$ = 1724 (*m*), 1532 (*s*), 1348 (*m*), 1269 (*m*), 1181 (*s*), 1125 (*s*), 1065 (*s*), 740 (*s*), 630 (*s*), 590 (*m*).

HRMS (ESI) calc. for $C_{18}H_{14}N_2NaO_6S$ $[M+Na]^+$: 409.0465; found: 409.0462.

Pyrrole 17c

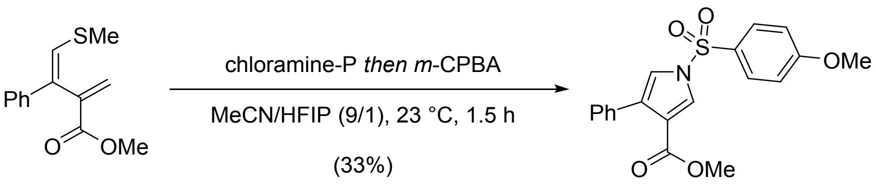

5a **17c**

To a solution of 1,3-diene **5a** (45 mg, 0.19 mmol, 1 equiv) in acetonitrile (1.8 mL) and hexafluoroisopropanol (0.2 mL) was added chloramine-P (105 mg, 388 μmol, 2.00 equiv) at 23 °C portionwise over a period of ten minutes. After 40 minutes, 3-chloroperoxobenzoic acid (33.1 mg, 192 μmol, 1.00 equiv) was added. After one hour, the solution was diluted with ethyl acetate (10 mL) and poured into saturated aqueous sodium thiosulfate solution (10 mL). The

layers were separated and the aqueous phase was extracted with ethyl acetate (2 × 5 mL). The combined organic layers were washed with saturated aqueous sodium bicarbonate solution (10 mL) and dried over sodium sulfate, the dried solution was filtered and the filtrate was concentrated under reduced pressure. The crude product was purified by flash-column chromatography on silica gel (15% to 20% ethyl acetate in *n*-pentane) to furnish pyrrole **17c** (24 mg, 33%) as a colorless, amorphous solid.

TLC (20% ethyl acetate in *n*-pentane): R_f = 0.37 (UV, $KMnO_4$).

^{1}H NMR (400 MHz, $CDCl_3$): δ = 7.91 – 7.87 (m, 2H), 7.83 (d, *J* = 2.5 Hz, 1H), 7.44 – 7.39 (m, 2H), 7.39 – 7.29 (m, 3H), 7.13 (d, *J* = 2.6 Hz, 1H), 7.03 – 6.98 (m, 2H), 3.87 (s, 3H), 3.73 (s, 3H).

^{13}C NMR (101 MHz, $CDCl_3$): δ = 164.6, 163.9, 132.8, 130.0, 129.9, 129.4, 129.3, 128.0, 127.6, 126.7, 119.4, 118.0, 115.1, 55.9, 51.5.

IR (Diamond-ATR, $CDCl_3$): $\tilde{\nu}_{max}$ = 1724 (*m*), 1375 (*m*), 1266 (*s*), 1165 (*s*), 1125 (*s*), 1092 (*m*), 1065 (*s*), 676 (*m*), 595 (*s*), 549 (*m*).

HRMS (ESI) calc. for $C_{19}H_{17}NNaO_5S$ $[M+Na]^+$: 394.0720; found: 394.0715.

Pyrrole 17d

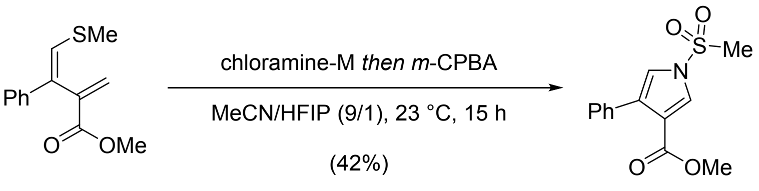

5a **17d**

To a solution of 1,3-diene **5a** (47 mg, 0.2 mmol, 1 equiv) in acetonitrile (1.8 mL) and hexafluoroisopropanol (0.2 mL) was added chloramine-M (84 mg, 0.50 mmol, 2.5 equiv) at 23 °C portionwise over a period of ten minutes. After 14 hours, 3-chloroperoxobenzoic acid (35 mg, 0.2 mmol, 1.0 equiv) was added. After stirring for one hour at 23 °C, the reaction mixture was poured into saturated aqueous sodium thiosulfate solution (10 mL) and ethyl acetate (10 mL) was added. The layers were separated, the aqueous layer was extracted with ethyl acetate (2 × 5 mL) and the combined organic layers were washed with saturated aqueous sodium bicarbonate solution (10 mL) and dried over sodium sulfate. The dried solution was filtrated and the filtrate was concentrated under reduced pressure. The crude product was purified by flash column chromatography on silica gel (25% ethyl acetate in *n*-pentane) to yield pyrrole **17d** (24 mg, 42%) as a colorless solid.

TLC (25% ethyl acetate in *n*-pentane): R_f = 0.30 (UV, $KMnO_4$).

^{1}H NMR (400 MHz, $CDCl_3$): δ = 7.82 (d, *J* = 2.6 Hz, 1H), 7.48 – 7.44 (m, 2H), 7.42 – 7.33 (m, 3H), 7.14 (d, *J* = 2.6 Hz, 1H), 3.77 (s, 3H), 3.27 (s, 3H).

^{13}C NMR (101 MHz, $CDCl_3$): δ = 163.7, 132.5, 130.3, 129.3, 128.1, 127.9, 126.7, 119.3, 118.5, 51.6, 43.4.

IR (Diamond-ATR, $CDCl_3$): $\tilde{\upsilon}_{max}$ = 1724 (*s*), 1370 (*s*), 1271 (*m*), 1174 (*s*), 1127 (*s*), 1069 (*m*), 983 (*m*), 763 (*m*), 571 (*m*), 511 (*m*).

HRMS (ESI) calc. for $C_{13}H_{14}NO_4S$ $[M+H]^+$: 260.0638; found: 260.0632.

MP = 132 – 134 °C.

4.2 Additional Substrates and Limitations

A. Additional Substrates

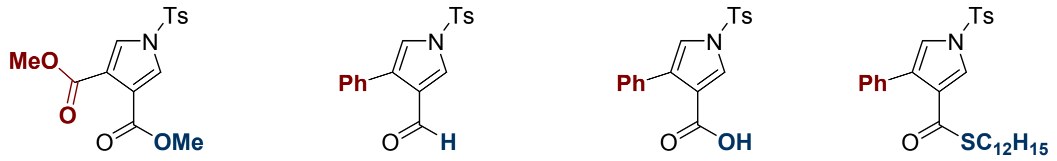

S-36 (27%) **S-37** (22%) **S-38** (16%) **S-39** (34%)

B. Limitations

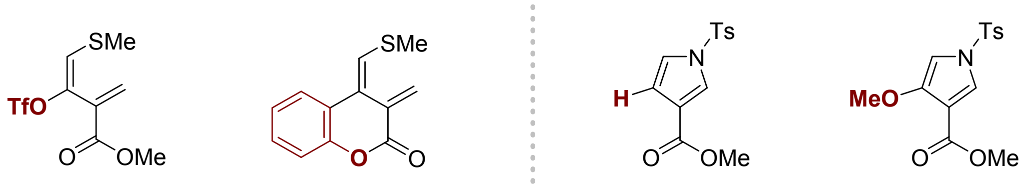

S-40 **S-41** **S-42** **S-43**

1,3-diene formation *pyrrole formation*

Pyrrole S-36

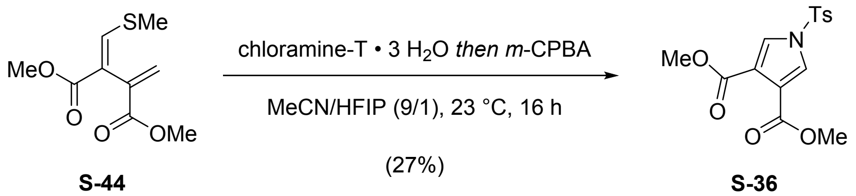

S-44 **S-36**

Following GP1 (15 hours for pyrrole formation), 1,3-diene **S-44** (48 mg, 0.20 mmol) was converted into pyrrole **S-36**. Purification by flash-column chromatography on silica gel (10% ethyl acetate in toluene) furnished pyrrole **S-36** (18 mg, 27%) as a colorless oil.

TLC (20% ethyl acetate in *n*-pentane): R_f = 0.28 (UV, $KMnO_4$).

^{1}H NMR (400 MHz, $CDCl_3$): δ = 7.85 – 7.78 (m, 2H), 7.65 (s, 2H), 7.41 – 7.32 (m, 2H), 3.82 (s, 6H), 2.44 (s, 3H).

^{13}C NMR (101 MHz, $CDCl_3$): δ = 162.7, 146.6, 134.4, 130.5, 127.6, 125.9, 119.4, 52.0, 21.8.

IR (Diamond-ATR, $CDCl_3$): $\tilde{\upsilon}_{max}$ = 1728 (*br*), 1516 (*w*), 1384 (*w*), 1273 (*m*), 1191 (*m*), 1176 (*s*), 1160 (*vs*), 1067 (*vs*), 674 (*vs*), 595 (*s*).

HRMS (ESI) calc. for $C_{15}H_{16}NO_6S$ $[M+H]^+$: 338,0693; found: 338.0688.

Pyrrole S-37

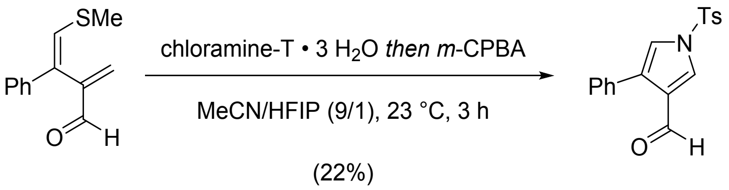

S-45 **S-37**

Following GP1 (two hours for pyrrole formation), 1,3-diene **S-45** (41 mg, 0.20 mmol) was converted into pyrrole **S-37**. Purification by flash-column chromatography on silica gel (5% to 40% ethyl acetate in cyclohexane) furnished pyrrole **S-37** (15 mg, 22%) as a yellow oil.

TLC (40% ethyl acetate in cyclohexane): R_f = 0.63 (UV, $KMnO_4$).

^{1}H NMR ($CDCl_3$, 400 MHz): δ = 9.89 (s, 1H), 7.85 (dd, *J* = 5.4, 2.9 Hz, 3H), 7.45 – 7.34 (m, 7H), 7.22 (d, *J* = 2.4 Hz, 1H), 2.44 (s, 3H).

^{13}C NMR ($CDCl_3$, 101 MHz): δ = 186.1, 146.5, 134.9, 131.8, 130.6, 129.7, 129.0, 128.7, 128.1, 127.6, 127.5, 126.5, 119.4, 21.9.

IR (Diamond-ATR, CH_2Cl_2): $\tilde{\upsilon}_{max}$ = 1687 (*m*), 1509 (*w*), 1378 (*m*), 1190 (*w*), 1174 (*s*), 1065 (*s*), 701 (*w*), 672 (*m*), 602 (*m*), 581 (*w*).

HRMS (ESI) calc. for $C_{18}H_{15}NNaO_3S$ $[M+Na]^+$: 348.0665; found: 348.0662.

Pyrrole S-38

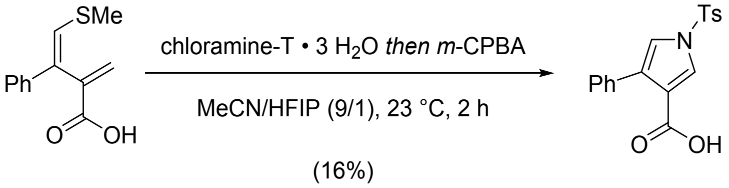

S-46 **S-38**

Following GP1 (one hour for pyrrole formation), 1,3-diene **S-46** (44 mg, 0.20 mmol) was converted into pyrrole **S-38**. Purification by flash-column chromatography on silica gel (20% ethyl acetate and 0.1% formic acid in cyclohexane) furnished pyrrole **S-38** (11 mg, 16%) as a colorless solid.

TLC (20% ethyl acetate in cyclohexane): R_f = 0.10 (UV, BCG, CAM).

^{1}H NMR (CD_2Cl_2, 400 MHz): δ = 7.92 (d, *J* = 2.5 Hz, 1H), 7.87 – 7.83 (m, 2H), 7.42 – 7.30 (m, 7H), 7.16 (d, *J* = 2.6 Hz, 1H), 2.43 (s, 3H).

^{13}C NMR (CD_2Cl_2, 101 MHz): δ = 167.7, 147.1, 135.3, 133.0, 131.0, 130.5, 129.7, 128.5, 128.5, 128.2, 128.0, 120.4, 117.5, 22.0.

IR (Diamond-ATR, $CDCl_3$): $\tilde{\upsilon}_{max}$ = 1692 (*m*), 1516 (*w*), 1379 (*w*), 1190 (*w*), 1174 (*s*), 1144 (*w*), 1065 (*m*), 671 (*m*), 593 (*m*), 539 (*w*).

HRMS (ESI) calc. for $C_{18}H_{14}NO_4S$ $[M–H]^–$: 340.0649; found: 340.0652.

Pyrrole S-39

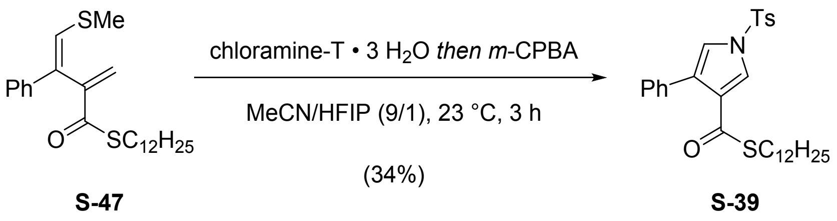

Following GP1 (two hours for pyrrole formation), 1,3-diene **S-47** (81 mg, 0.20 mmol) was converted into pyrrole **S-39**. Purification by flash-column chromatography on silica gel (2% to 8% ethyl acetate in cyclohexane) furnished pyrrole **S-39** (35 mg, 34%) as a colorless oil.

TLC (20% ethyl acetate in cyclohexane): R_f = 0.77 (UV, $KMnO_4$).

^{1}H NMR ($CDCl_3$, 400 MHz): δ = 7.85 (d, *J* = 2.4 Hz, 2H), 7.83 (d, *J* = 1.9 Hz, 1H), 7.40 – 7.29 (m, 7H), 7.14 (d, *J* = 2.5 Hz, 1H), 2.94 (t, *J* = 7.4 Hz, 2H), 2.44 (s, 3H), 1.64 – 1.56 (m, 2H), 1.35 – 1.21 (m, 18H), 0.93 – 0.79 (m, 3H).

^{13}C NMR ($CDCl_3$, 101 MHz): δ = 186.3, 146.2, 135.2, 132.5, 130.5, 129.1, 128.9, 128.2, 127.8, 127.5, 126.5, 125.1, 119.7, 32.1, 29.8, 29.7, 29.7, 29.5, 29.3, 29.1, 29.1, 22.8, 21.9, 14.3.

IR (Diamond-ATR, $CDCl_3$): $\tilde{\upsilon}_{max}$ = 2924 (*m*), 2853 (*w*), 1665 (*w*), 1382 (*w*), 1189 (*w*), 1175 (*m*), 1069 (*s*), 860 (*m*), 671 (*s*), 598 (*w*).

HRMS (ESI) calc. for $C_{30}H_{39}KNO_3S_2$ $[M+K]^+$: 564.2003; found: 564.2004

4.3 Mechanistic Investigations

Sulfilimine 19

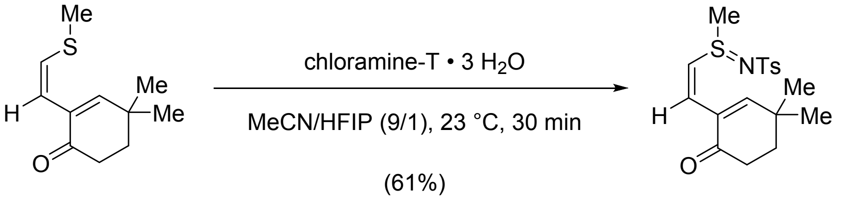

18 **19**

To a solution of 1,3-diene **18** (452 mg, 2.30 mmol, 1 equiv) in acetonitrile and hexafluoroisopropanol (9/1, 22.2 mL) was added chloramine-T trihydrate (1.32 g, 4.60 mmol, 2.00 equiv) portionwise over ten minutes at 23 °C. After 30 minutes saturated aqueous sodium thiosulfate solution (50 mL) and ethyl acetate (50 mL) were added and the layers were separated. The aqueous phase was extracted with ethyl acetate (2 × 50 mL), the combined organic layers were dried over sodium sulfate, the dried solution was filtered and the filtrate was concentrated under reduced pressure. The crude product was purified by flash-column chromatography on silica gel (50% ethyl acetate in dichloromethane) to yield sulfilimine **19** (515 mg, 61%) as a colorless solid. Recrystallization from chloroform gave crystals suitable for X-ray analysis.

TLC (50% ethyl acetate in dichloromethane): R_f = 0.23 (UV, $KMnO_4$, CAM).

^{1}H NMR ($CDCl_3$, 400 MHz): δ = 7.73 – 7.62 (m, 2H), 7.17 (d, *J* = 8.0 Hz, 2H), 6.81 (d, *J* = 1.1 Hz, 1H), 6.60 (dd, *J* = 10.1, 1.2 Hz, 1H), 6.25 (d, *J* = 10.1 Hz, 1H), 2.81 (s, 3H), 2.55 – 2.40 (m, 2H), 2.35 (s, 3H), 1.91 – 1.78 (m, 2H), 1.21 (s, 3H), 1.17 (s, 3H).

^{13}C NMR ($CDCl_3$, 101 MHz): δ = 197.0, 162.9, 141.8, 141.6, 135.6, 130.2, 129.2, 129.1, 126.3, 36.4, 35.3, 34.4, 34.0, 28.0, 27.1, 21.5.

IR (Diamond-ATR, CH_2Cl_2): $\tilde{\upsilon}_{max}$ = 2959 (*w*), 2924 (*w*), 1677 (*s*), 1279 (*m*), 1140 (*s*), 1090 (*m*), 1022 (*w*), 998 (*w*), 946 (*m*), 571 (*w*).

HRMS (ESI) calc. for $C_{18}H_{24}NO_3S_2$ $[M+H]^+$: 366.1192; found: 366.1186.

MP: 141 – 142 °C.

Pyrrole 20

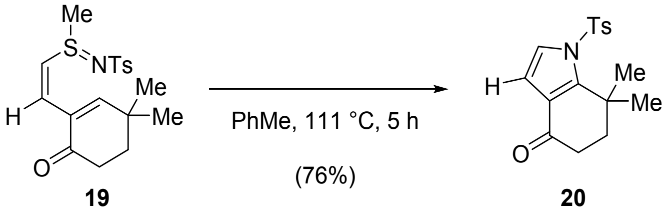

19 **20**

Sulfilimine **19** (37 mg, 0.10 mmol, 1 equiv) was dissolved in toluene (2.0 mL) and heated to 111 °C. After five hours, the solvent was removed under reduced pressure. Purification by

flash-column chromatography on silica gel (20% ethyl acetate in cyclohexane) furnished pyrrole **20** (24 mg, 76%) as a colorless oil.

TLC (20% ethyl acetate in cyclohexane): R_f = 0.18 (UV, $KMnO_4$, CAM).

^{1}H NMR (400 MHz, $CDCl_3$): δ = 7.62 – 7.57 (m, 2H), 7.34 – 7.28 (m, 3H), 6.65 (d, *J* = 3.6 Hz, 1H), 2.54 – 2.48 (m, 2H), 2.43 (s, 3H), 1.95 – 1.86 (m, 2H), 1.40 (s, 6H).

^{13}C NMR (101 MHz, $CDCl_3$): δ = 195.1, 151.1, 145.4, 137.1, 130.2, 126.8, 126.1, 125.0, 107.8, 41.8, 34.6, 34.4, 27.1, 21.8.

IR (Diamond-ATR, $CDCl_3$): $\tilde{\upsilon}_{max}$ = 1677 (*m*), 1365 (*m*), 1190 (*w*), 1174 (*s*), 1112 (*s*), 1083 (*m*), 671 (*s*), 594 (*m*), 564 (*m*), 542 (*m*).

HRMS (ESI) calc. for $C_{17}H_{20}NO_3S$ $[M+H]^+$: 318.1158; found: 318.1153.

Sulfilimine S-50

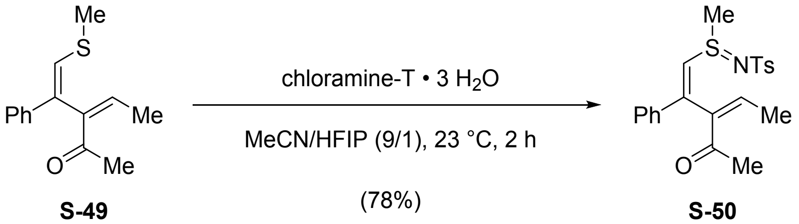

S-49 **S-50**

To a solution of 1,3-diene **S-49** (47 mg, 0.20 mmol, 1 equiv) in acetonitrile and hexafluoroisopropanol (9/1, 2.0 mL) was added chloramine-T trihydrate (115 mg, 400 μmol, 2.00 equiv) portionwise over ten minutes at 23 °C. After two hours, saturated aqueous sodium thiosulfate solution (20 mL) and ethyl acetate (20 mL) were added, the layers were separated and the aqueous phase was extracted with ethyl acetate (2 × 20 mL). The combined organic layers were washed once with saturated aqueous sodium bicarbonate solution and dried over sodium sulfate, the dried solution was filtered and the filtrate was concentrated under reduced pressure. The crude product was purified by flash-column chromatography on silica gel (50% ethyl acetate in dichloromethane) to yield sulfilimine **S-50** (63 mg, 78%) as a colorless oil.

TLC (50% ethyl acetate in dichloromethane): R_f = 0.33 (UV, $KMnO_4$, CAM).

^{1}H NMR ($CDCl_3$, 400 MHz): δ = 7.65 (d, *J* = 8.3 Hz, 2H), 7.29 – 7.26 (m, 1H), 7.25 – 7.19 (m, 2H), 7.18 – 7.13 (m, 3H), 7.09 (d, *J* = 2.0 Hz, 1H), 7.04 (q, *J* = 7.1 Hz, 1H), 6.67 (s, 1H), 2.69 (s, 3H), 2.32 (s, 3H), 2.26 (s, 3H), 1.51 (d, *J* = 7.0 Hz, 3H).

^{13}C NMR ($CDCl_3$, 101 MHz): δ = 197.5, 142.6, 141.7, 135.8, 130.7, 129.9, 129.3, 129.2, 128.8, 128.5, 127.7, 126.9, 126.4, 125.6, 36.3, 21.5, 16.6

IR (Diamond-ATR, CH_2Cl_2): $\tilde{\upsilon}_{max}$ = 1665 (*m*), 1279 (*m*), 1139 (*s*), 1089 (*w*), 1022 (*w*), 977 (*w*), 945 (*m*), 758 (*w*), 572 (*w*), 550 (*w*).

HRMS (ESI) calc. for $C_{21}H_{24}NO_3S_2$ $[M+H]^+$: 402.1192; found: 402.1192.

Pyrrole 21

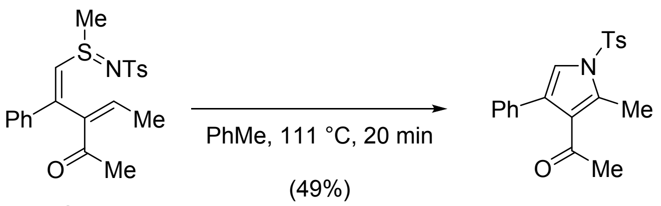

S-50 **21**

Sulfilimine **S-50** (40 mg, 0.10 mmol, 1 equiv) was dissolved in toluene (2.0 mL) and heated to 111 °C. After five hours, the solvent was removed under reduced pressure. Purification by flash-column chromatography on silica gel (20% ethyl acetate in cyclohexane) followed by HPLC purification (8% grading to 15% ethyl acetate in *n*-hexane within 30 minutes) furnished pyrrole **21** (17 mg, 49%) as a colorless oil.

TLC (40% ethyl acetate in cyclohexane): R_f = 0.61 (UV, $KMnO_4$, CAM).

^{1}H NMR (400 MHz, $CDCl_3$): δ = 7.80 – 7.74 (m, 2H), 7.43 – 7.32 (m, 5H), 7.32 – 7.27 (m, 3H), 2.50 (s, 3H), 2.44 (s, 3H), 1.99 (s, 3H).

^{13}C NMR (101 MHz, $CDCl_3$): δ = 198.7, 145.9, 135.5, 134.3, 134.2, 130.4, 128.9, 128.7, 127.7, 127.6, 126.9, 126.7, 119.2, 31.6, 21.8, 12.1.

IR (Diamond-ATR, $CDCl_3$): $\tilde{\upsilon}_{max}$ = 1672 (*m*), 1380 (*m*), 1370 (*m*), 1190 (*m*), 1175 (*s*), 1122 (*w*), 1094 (*s*), 671 (*s*), 587 (*m*), 541 (*m*).

HRMS (ESI) calc. for $C_{20}H_{20}NO_3S$ $[M+H]^+$: 354.1158; found: 354.1155.

Sulfilimine S-52

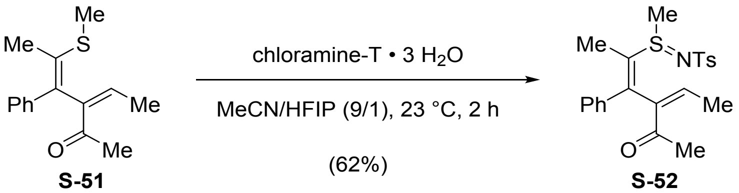

S-51 **S-52**

To a solution of 1,3-diene **S-51** (39 mg, 0.16 mmol, 1 equiv) in acetonitrile and hexafluoroisopropanol (9/1, 1.6 mL) was added chloramine-T trihydrate (89 mg, 0.32 mmol, 2.0 equiv) at 23 °C portionwise over a period of ten minutes. After two hours, the solution was diluted with ethyl acetate (10 mL) and washed with saturated aqueous sodium thiosulfate solution (10 mL). The organic layer was dried over sodium sulfate, the dried solution was filtrated and the filtrate was concentrated under reduced pressure. The crude product was purified by flash-column chromatography on silica gel (25% ethyl acetate in dichloromethane) to furnish sulfilimine **S-52** (41 mg, 62%) as a colorless foam.

TLC (25% ethyl acetate in dichloromethane): R_f = 0.30 (UV, $KMnO_4$).

^{1}H NMR (400 MHz, $CDCl_3$): δ = 7.75 (d, *J* = 8.2 Hz, 2H), 7.37 – 7.28 (m, 3H), 7.23 (d, *J* = 8.0 Hz, 2H), 7.10 – 7.05 (m, 2H), 6.99 (q, *J* = 7.1 Hz, 1H), 2.65 (s, 3H), 2.39 (s, 3H), 2.37 (s, 3H), 2.17 (s, 3H), 1.80 (d, *J* = 7.1 Hz, 3H).

^{13}C NMR (101 MHz, $CDCl_3$): δ = 197.9, 144.4, 143.6, 142.5, 141.9, 141.5, 137.5, 134.1, 129.1, 128.6, 128.5, 128.2, 126.3, 33.4, 26.0, 21.4, 16.5, 10.5.

IR (Diamond-ATR, $CDCl_3$): $\tilde{\upsilon}_{max}$ = 1666 (*m*), 1281 (*m*), 1141 (*s*), 1090 (*m*), 1022 (*w*), 998 (*m*), 947 (*m*), 702 (*w*), 573 (*m*), 551 (*w*).

HRMS (ESI) calc. for $C_{22}H_{25}NNaO_3S_2$ $[M+Na]^+$: 438.1168; found: 438.1162.

Pyrrole 22

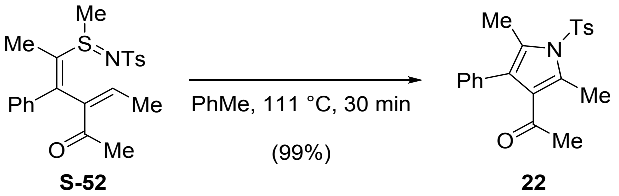

Sulfilimine **S-52** (35 mg, 85 µmol) was dissolved in toluene (1.6 mL) and the reaction mixture was heated to 111 °C. After 30 minutes, the reaction mixture was allowed to cool to 23 °C and the solvent was removed under reduced pressure to furnish pyrrole **22** (31 mg, 99%) as a colorless solid.

TLC (10% ethyl acetate in *n*-pentane): R_f = 0.33 (UV, $KMnO_4$).

^{1}H NMR (400 MHz, $CDCl_3$): δ = 7.72 – 7.65 (m, 2H), 7.41 – 7.31 (m, 5H), 7.21 – 7.14 (m, 2H), 2.63 (s, 3H), 2.44 (s, 3H), 2.29 (s, 3H), 1.80 (s, 3H).

^{13}C NMR (101 MHz, $CDCl_3$): δ = 198.9, 145.4, 136.8, 134.8, 130.2, 130.2, 128.5 (2C), 127.5, 126.8, 126.4, 124.9, 31.7, 21.8, 13.4, 13.3 (one *signal missing, not assignable through 2D NMR analysis*).

IR (Diamond-ATR, $CDCl_3$): $\tilde{\upsilon}_{max}$ = 1671 (*m*), 1371 (*s*), 1194 (*m*), 1166 (*s*), 1096 (*s*), 767 (*w*), 705 (*m*), 671 (*m*), 568 (*m*), 543 (*m*).

HRMS (ESI) calc. for $C_{21}H_{22}NO_3S$ $[M+H]^+$: 368.1315; found: 368.1311.

MP = 176 – 178 °C.

Sulfilimine S-54

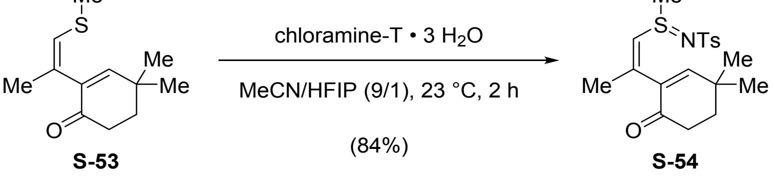

To a solution of 1,3-diene **S-53** (41.5 mg, 197 µmol, 1 equiv) in acetonitrile (1.8 mL) and hexafluoroisopropanol (0.2 mL) was added chloramine-T trihydrate (96.9 mg, 395 µmol,

2.00 equiv) at 23 °C portionwise over a period of ten minutes. After two hours, the reaction mixture was diluted with ethyl acetate (10 mL) and washed with saturated aqueous sodium thiosulfate solution (10 mL) The organic layer was dried over sodium sulfate, the dried solution was filtrated and the filtrate was concentrated under reduced pressure. The crude product was purified by flash-column chromatography on silica gel (50% ethyl acetate in dichloromethane) to furnish sulfilimine **S-54** (62 mg, 84%) as a colorless oil.

TLC (50% ethyl acetate in dichloromethane): R_f = 0.26 (UV, $KMnO_4$).

^{1}H NMR (400 MHz, $CDCl_3$): δ = 7.67 (d, *J* = 8.2 Hz, 2H), 7.17 (d, *J* = 8.3 Hz, 2H), 6.48 (s, 1H), 6.11 (s, 1H), 2.70 (s, 3H), 2.47 (dd, *J* = 8.0, 5.7 Hz, 2H), 2.35 (s, 3H), 1.91 (s, 3H), 1.88 - 1.82 (m, 2H), 1.19 (s, 3H), 1.14 (s, 3H).

^{13}C NMR (101 MHz, $CDCl_3$): δ = 196.6, 159.3, 148.3, 141.9, 141.4, 134.9, 129.1, 126.3, 125.1, 35.7, 35.4, 34.4, 33.4, 27.6, 27.3, 24.7, 21.3.

IR (Diamond-ATR, $CDCl_3$): $\tilde{\nu}_{max}$ = 1674 (*s*), 1278 (*s*), 1138 (*s*), 1098 (*s*), 994 (*m*), 944 (*s*), 747 (*w*), 653 (*w*), 572 (*m*), 551 (*m*).

HRMS (ESI) calc. for $C_{19}H_{25}NNaO_3S_2$ $[M+Na]^+$: 402.1168; found: 402.1161.

Pyrrole 23

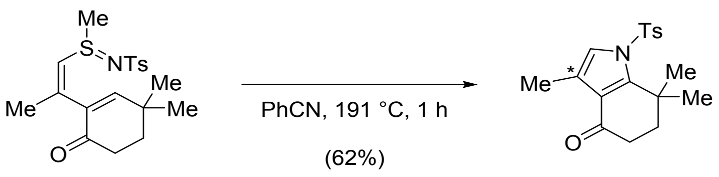

S-54 **23**

A solution of sulfilimine **S-54** (26 mg, 68 μmol) in benzonitrile (1.4 mL) was heated to 191 °C. After one hour, the reaction mixture was allowed to cool to 23 °C and the solvent was removed under reduced pressure. The crude product was purified by flash-column chromatography on silica gel (dichloromethane) to furnish pyrrole **23** (14 mg, 62%) as a pale yellow solid.

TLC (10% ethyl acetate in *n*-pentane): R_f = 0.29 (UV, $KMnO_4$).

^{1}H NMR (400 MHz, $CDCl_3$): δ = 7.59 (d, *J* = 8.5 Hz, 2H), 7.31 (d, *J* = 8.3 Hz, 2H), 7.07 (s, 1H), 2.51 – 2.46 (m, 2H), 2.43 (s, 3H), 2.25 (s, 3H), 1.92 – 1.85 (m, 2H), 1.40 (s, 6H).

^{13}C NMR (101 MHz, $CDCl_3$): δ = 196.5, 151.4, 145.2, 137.4, 130.1, 126.7, 123.3, 120.8, 41.9, 35.5, 34.4, 27.2, 21.8, 11.9. (* one *signal missing, not assignable through 2D NMR analysis*).

IR (Diamond-ATR, $CDCl_3$): $\tilde{\nu}_{max}$ = 2926 (*w*), 1677 (*s*), 1422 (*w*), 1365 (*m*), 1299 (*w*), 1190 (*w*), 1177 (*s*), 1105 (*m*), 665 (*m*), 583 (*s*).

HRMS (ESI) calc. for $C_{18}H_{21}NNaO_3S$ $[M+Na]^+$: 354.1134; found: 354.1134.

MP = 167 – 169 °C.

Sulfilimine 32

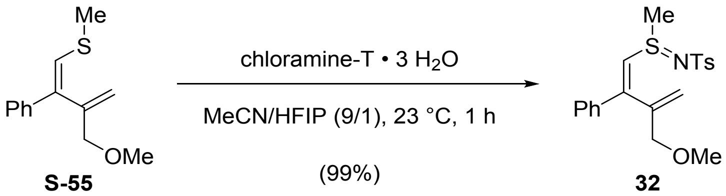

To a solution of 1,3-diene **S-55** (42 mg, 0.19 mmol, 1 equiv) in acetonitrile and hexafluoroisopropanol (9:1, 2.0 mL) was added chloramine-T trihydrate (107 mg, 381 µmol, 2.00 equiv) in one portion at 23 °C. After one hour, saturated aqueous sodium thiosulfate solution (10 mL) and ethyl acetate (10 mL) were added. The layers were separated, the aqueous phase was extracted with ethyl acetate (3 × 10 mL), the combined organic layers were dried over sodium sulfate, the dried solution was filtered and the filtrate was concentrated under reduced pressure. The crude product was purified by flash-column chromatography on silica gel (50% ethyl acetate in dichloromethane) to yield sulfilimine **32** (74 mg, 99%) as a colorless oil.

TLC (50% ethyl acetate in dichloromethane): R_f = 0.24 (UV, $KMnO_4$).

^{1}H NMR ($CDCl_3$, 400 MHz): δ = 7.77 (d, *J* = 8.2 Hz, 2H), 7.43 – 7.32 (m, 3H), 7.30 – 7.26 (m, 2H), 7.18 (d, *J* = 7.6 Hz, 2H), 6.46 (s, 1H), 5.58 (d, *J* = 1.4 Hz, 1H), 5.08 (d, *J* = 1.1 Hz, 1H), 3.85 – 3.77 (m, 1H), 3.76 – 3.69 (m, 1H), 3.23 (s, 3H), 2.83 (s, 3H), 2.34 (s, 3H).

^{13}C NMR ($CDCl_3$, 101 MHz): δ = 153.8, 142.0, 141.9, 141.6, 135.1, 130.6, 129.2, 129.0, 127.4, 126.5, 124.8, 120.4, 73.5, 58.6, 37.5, 21.4.

IR (Diamond-ATR, CH_2Cl_2): $\tilde{\upsilon}_{max}$ = 1279 (*m*), 1140 (*s*), 1090 (*m*), 1021 (*w*), 1001 (*w*), 940 (*m*), 760 (*w*), 655 (*w*), 573 (*w*), 551 (*w*).

HRMS (ESI) calc. for $C_{20}H_{24}NO_3S_2$ $[M+H]^+$: 390.1192; found: 390.1193.

Pyrrole 24

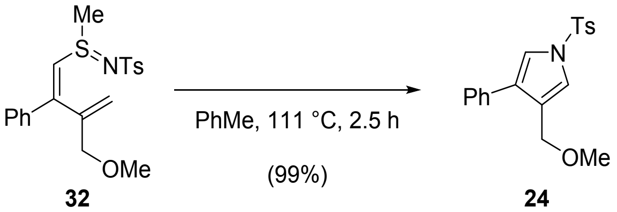

Sulfilimine **32** (40 mg, 0.10 mmol, 1 equiv) was dissolved in toluene (1.3 mL) and heated to 111 °C. After 2.5 hours, the solvent was removed under reduced pressure to furnish pyrrole **24** (35 mg, 99%) as a pale-orange oil.

TLC (20% ethyl acetate in petroleum ether): R_f = 0.54 (UV, $KMnO_4$).

^{1}H NMR (400 MHz, $CDCl_3$): δ = 7.79 (d, *J* = 8.4 Hz, 2H), 7.47 – 7.42 (m, 2H), 7.40 – 7.34 (m, 2H), 7.32 – 7.27 (m, 3H), 7.27 – 7.23 (m, 2H), 4.31 (s, 2H), 3.36 (s, 3H), 2.41 (s, 3H).

^{13}C NMR (101 MHz, $CDCl_3$): δ = 145.3, 136.0, 133.6, 130.2, 129.8, 128.8, 128.1, 127.3, 127.2, 123.9, 121.1, 118.2, 66.5, 57.9, 21.8.

IR (Diamond-ATR, $CDCl_3$): $\tilde{\upsilon}_{max}$ = 1370 (*m*), 1320 (*w*), 1171 (*s*), 1090 (*m*), 1067 (*s*), 812 (*w*), 702 (*w*), 672 (*s*), 599 (*m*), 589 (*m*).

HRMS (ESI) calc. for $C_{19}H_{19}NNaO_3S$ $[M+Na]^+$: 364.0978; found: 364.0975.

Sulfilimine 25

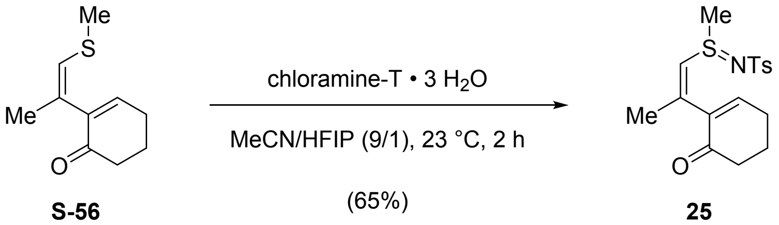

S-56 **25**

To a solution of 1,3-diene **S-56** (125 mg, 684 µmol, 1 equiv) in acetonitrile and hexafluoroisopropanol (9/1, 6.70 mL) was added chloramine-T trihydrate (295 mg, 1.03 mmol, 1.50 equiv) portionwise over ten minutes at 23 °C. After two hours, saturated aqueous sodium thiosulfate solution (20 mL) and ethyl acetate (20 mL) were added and the layers were separated. The aqueous phase was extracted with ethyl acetate (2 × 50 mL), the combined organic layers were dried over sodium sulfate, the dried solution was filtered and the filtrate was concentrated under reduced pressure. The crude product was purified by flash-column chromatography on silica gel (3% methanol in dichloromethane) to yield sulfilimine **25** (157 mg, 65%) as a colorless oil.

TLC (3% methanol in dichloromethane): R_f = 0.18 (UV, $KMnO_4$, CAM).

^{1}H NMR ($CDCl_3$, 400 MHz): δ = 7.71 (d, *J* = 8.1 Hz, 2H), 7.20 (d, *J* = 7.9 Hz, 2H), 6.80 (t, *J* = 4.2 Hz, 1H), 6.17 – 6.12 (m, 1H), 2.73 (s, 3H), 2.53 – 2.41 (m, 4H), 2.38 (s, 3H), 2.04 (p, *J* = 6.2 Hz, 2H), 1.96 (d, *J* = 1.4 Hz, 3H).

^{13}C NMR ($CDCl_3$, 101 MHz): δ = 196.9, 150.8, 148.6, 142.1, 141.6, 138.0, 129.2, 126.6, 125.1, 38.3, 35.7, 26.1, 24.8, 22.4, 21.6.

IR (Diamond-ATR, $CDCl_3$): $\tilde{\upsilon}_{max}$ = 1677 (*m*), 1349 (*w*), 1278 (*m*), 1161 (*s*), 1140 (*s*), 1090 (*s*), 942 (*m*), 662 (*w*), 584 (*m*), 550 (*m*).

HRMS (ESI) calc. for $C_{17}H_{22}NO_3S_2$ $[M+H]^+$: 352.1036; found: 352.1035.

Pyrrole 27

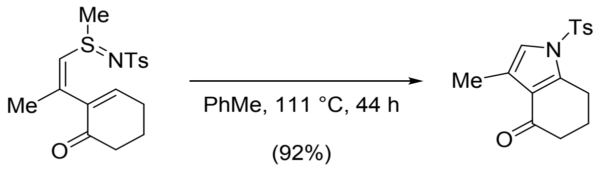

25 **27**

A solution of sulfilimine **25** (30 mg, 85 µmol, 1 equiv) in toluene was heated to 111 °C. After 44 hours, the solvent was removed under reduced pressure and the crude product was purified by flash-column chromatography on silica gel (10% to 25% *tert*-butyl methyl ether in petroleum ether) to furnish pyrrole **27** (24 mg, 92%) as a colorless oil.

TLC (20% ethyl acetate in cyclohexane): R_f = 0.21 (UV, $KMnO_4$, CAM).

^{1}H NMR (400 MHz, $CDCl_3$): δ = 7.75 – 7.71 (m, 2H), 7.34 (d, *J* = 8.1 Hz, 2H), 6.96 (d, *J* = 1.4 Hz, 1H), 2.95 (t, *J* = 6.2 Hz, 2H), 2.44 (s, 3H), 2.39 (dd, *J* = 7.3, 5.7 Hz, 2H), 2.23 (d, *J* = 1.3 Hz, 3H), 2.06 (p, *J* = 6.4 Hz, 2H).

^{13}C NMR (101 MHz, $CDCl_3$): δ = 195.5, 145.6, 143.9, 135.6, 130.3, 127.1, 123.4, 121.6, 119.2, 38.2, 23.3, 23.1, 21.7, 11.5.

IR (Diamond-ATR, $CDCl_3$): $\tilde{\nu}_{max}$ = 2924 (*w*), 1671 (*s*), 1428 (*w*), 1376 (*m*), 1189 (*m*), 1175 (*s*), 1124 (*m*), 1099 (*s*), 1024 (*w*), 670 (*s*).

HRMS (ESI) calc. for $C_{16}H_{18}NO_3S$ $[M+H]^+$: 304.1002; found: 304.1000.

Pyrroles 26.S-57 and 27

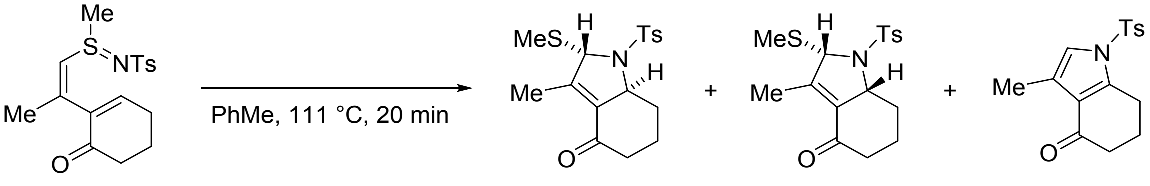

25 **26** (44%) **S-57** (10%) **27** (10%)

Sulfilimine **XX** (70 mg, 0.20 mmol, 1 equiv) was dissolved in toluene (4.0 mL) and heated to 111 °C. After 20 minutes the solvent was removed under reduced pressure. Purification by flash-column chromatography on silica gel (20% to 30% ethyl acetate in cyclohexane) followed by HPLC purification (15% grading to 30% ethyl acetate in *n*-hexane within 30 minutes) furnished pyrrole **27** (6.2 mg, 10%) as a colorless oil, 2,5-dihydropyrrole **26** (31 mg, 44%) as a colorless, crystalline solid and 2,5-dihydropyrrole **S-57** (8.9 mg, 10%) as a colorless oil. Recrystallization of **26** from dichloromethane gave crystals suitable for X-ray analysis.

TLC (20% ethyl acetate in cyclohexane): R_f = 0.21 (UV, $KMnO_4$, CAM).

Major diastereomer **26**:

^{1}H NMR (400 MHz, $CDCl_3$): δ = 7.97 – 7.86 (m, 2H), 7.33 – 7.27 (m, 2H), 5.56 – 5.50 (m, 1H), 4.30 – 4.21 (m, 1H), 2.97 – 2.88 (m, 1H), 2.55 – 2.47 (m, 1H), 2.43 (s, 3H), 2.29 – 2.17 (m, 1H), 2.10 – 2.03 (m, 1H), 2.01 (dd, J = 2.0, 0.8 Hz, 3H), 1.76 – 1.60 (m, 2H), 1.39 (s, 3H).

^{13}C NMR (101 MHz, $CDCl_3$): δ = 198.2, 143.7, 142.1, 137.0, 133.3, 129.4, 128.2, 75.6, 66.8, 40.6, 32.9, 21.7, 20.8, 12.7, 10.5.

IR (Diamond-ATR, CH_2Cl_2): $\tilde{\upsilon}_{max}$ = 1692 (*w*), 1639 (*w*), 1349 (*m*), 1161 (*s*), 1098 (*m*), 1065 (*w*), 712 (*w*), 663 (*w*), 584 (*m*), 547 (*w*).

HRMS (ESI) calc. for $C_{17}H_{21}KNO_3S_2$ $[M+K]^+$: 390.0594; found: 390.0592.

MP: 143 – 144 °C.

Minor diastereomer **S-57**:

^{1}H NMR (400 MHz, $CDCl_3$): δ = 7.80 (d, 2H), 7.31 (d, J = 8.0 Hz, 2H), 5.53 (dq, J = 2.4, 1.1 Hz, 1H), 4.53 (ddt, J = 11.6, 4.8, 2.4 Hz, 1H), 2.52 (ddd, J = 12.9, 5.6, 3.6 Hz, 3H), 2.44 (s, 3H), 2.20 (ddd, J = 17.5, 12.8, 6.7 Hz, 1H), 2.02 (dh, J = 13.4, 3.0 Hz, 1H), 1.95 (dd, J = 2.3, 1.1 Hz, 3H), 1.84 (s, 3H), 1.82 – 1.72 (m, 1H).

^{13}C NMR (101 MHz, $CDCl_3$): δ = 198.0, 144.2, 141.0, 135.7, 133.1, 129.9, 127.8, 75.7, 67.2, 41.3, 33.1, 21.7, 20.9, 12.5, 10.3.

IR (Diamond-ATR, CH_2Cl_2): $\tilde{\upsilon}_{max}$ = 1692 (*m*), 1645 (*w*), 1351 (*w*), 1159 (*s*), 1093 (*m*), 1045 (*w*), 710 (*m*), 661 (*w*), 581 (*s*), 549 (*m*).

HRMS (ESI) calc. for $C_{17}H_{21}KNO_3S_2$ $[M+K]^+$: 390.0594; found: 390.0592.

Pyrrole 27

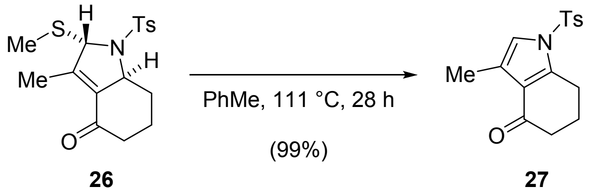

26 **27**

A solution of 2,5-dihydropyrrole **26** (23 mg, 65 µmol, 1 equiv) in toluene was heated to 111 °C. After 28 hours, the solvent was removed under reduced pressure to furnish pure pyrrole **27** (20 mg, 99%) as a colorless oil. The obtained analytical data was in full agreement with those obtained from previous experiments.

Chloramine-T triggered Elimination

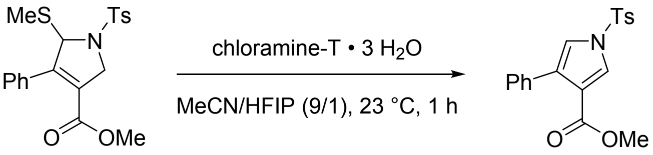

10 **9a** (95%)

To a solution of 2,5- dihydropyrrole **10** (5.1 mg, 13 µmol, 1 equiv) in acetonitrile (0.22 mL) and hexafluoroisopropanol (0.02 mL) was added chloramine-T (3.6 mg, 13 µmol, 1.0 equiv) at 23 °C. After one hour, saturated aqueous sodium thiosulfate solution (5 mL) and ethyl acetate (5 mL) were added. The layers were separated and the aqueous layer was extracted with dichloromethane (2 × 5 mL). The combined organic layers were dried over sodium sulfate, the dried solution was filtered and the filtrate was concentrated under reduced pressure. Methyl phenyl sulfone (3.9 mg, 25 µmol, 2.0 equiv) was added as an internal ^{1}H NMR standard to the crude reaction mixture to determine the yield of pyrrole **9a** (95%).

Sulfoxide 28

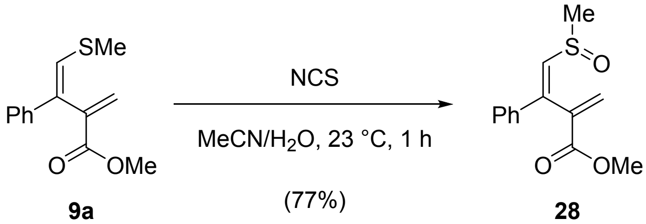

9a **28**

To a solution of 1,3-diene **9a** (100 mg, 427 µmol, 1 equiv) in acetonitrile (2.1 mL) and water (2.1 mL) was added *N*-chlorosuccinimide (64.0 mg, 469 µmol, 1.10 equiv) at 23 °C. After one hour, water (10 mL) and ethyl acetate (10 mL) were added. The layers were separated and the organic layer was washed with water (3 × 10 mL). The organic layer was dried over sodium sulfate, the dried solution was filtered and the filtrate was concentrated. The crude product was purified by flash-column chromatography on silica gel (3% methanol in dichloromethane) to yield sulfoxide **28** (82 mg, 77%) as a colorless solid.

TLC (5% methanol in dichloromethane): R_f = 0.37 (UV, $KMnO_4$).

^{1}H NMR ($CDCl_3$, 400 MHz): δ = 7.45 – 7.35 (m, 5H), 6.80 (s, 1H), 6.72 (d, *J* = 1.0 Hz, 1H), 5.87 (d, *J* = 1.0 Hz, 1H), 3.71 (s, 3H), 2.73 (s, 3H).

^{13}C NMR ($CDCl_3$, 101 MHz): δ = 165.6, 147.2, 137.1, 137.1, 135.2, 132.1, 130.0, 129.0, 127.2, 52.7, 40.4.

IR (Diamond-ATR, CH_2Cl_2): $\tilde{\upsilon}_{max}$ = 1723 (*s*), 1444 (*w*), 1340 (*w*), 1253 (*m*), 1141 (*m*), 1029 (*m*), 983 (*w*), 816 (*w*), 763 (*m*), 697 (*w*).

HRMS (ESI) calc. for $C_{13}H_{14}KO_3S$ $[M+K]^+$: 289.0295; found: 289.0285.

MP: 122 – 123 °C.

2,5-Dihydropyrrole 10

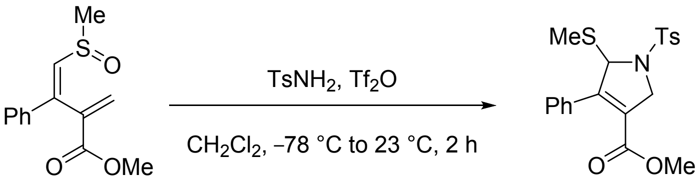

28 **10**

To a solution of sulfoxide **28** (52.3 mg, 209 µmol, 1 equiv) and *p*-toluenesulfonamide (125 mg, 731 µmol, 3.50 equiv) in dichloromethane (2.0 mL) under argon was added trifluoromethanesulfonic anhydride (42 µL, 0.25 mmol, 1.2 equiv) dropwise at –78 °C. After one hour, the reaction mixture was allowed to warm to 23 °C. After one hour, aqueous sodium hydroxide solution (1 M, 15 mL) and dichloromethane (15 mL) were added, the layers were separated and the aqueous phase extracted with dichloromethane (2 × 15 mL). The combined organic layer were dried over sodium sulfate, the dried solution was filtered and the solvent removed under reduced pressure. The crude product was purified by flash-column chromatography on silica gel (20% ethyl acetate in cyclohexane) to furnish 2,5-dihydropyrrole **10** (45.6 mg, 54%) as a colorless oil.

TLC (20% ethyl acetate in cyclohexane): R_f = 0.27 (UV, $KMnO_4$).

^{1}H NMR ($CDCl_3$, 400 MHz): δ = 7.89 – 7.84 (m, 2H), 7.39 – 7.30 (m, 5H), 7.26 – 7.20 (m, 2H), 6.02 (dd, J = 5.1, 1.4 Hz, 1H), 4.69 (dd, J = 15.3, 1.4 Hz, 1H), 4.38 (dd, J = 15.3, 5.1 Hz, 1H), 3.62 (s, 3H), 2.44 (s, 3H), 1.84 (s, 3H).

^{13}C NMR ($CDCl_3$, 101 MHz): δ = 163.6, 148.5, 144.1, 135.9, 131.7, 129.9, 129.5, 128.5, 128.2, 127.6, 125.4, 76.3, 55.6, 51.9, 21.7, 11.4.

IR (Diamond-ATR, CH_2Cl_2): $\tilde{\upsilon}_{max}$ = 1730 (*m*), 1706 (*m*), 1531 (*m*), 1274 (*m*), 1231 (*m*), 1163 (*s*), 1095 (*m*), 688 (*m*), 667 (*s*), 600 (*m*).

HRMS (ESI) calc. for $C_{20}H_{21}NNaO_4S_2$ $[M+Na]^+$: 426.0804; found: 426.0786.

Furan 29

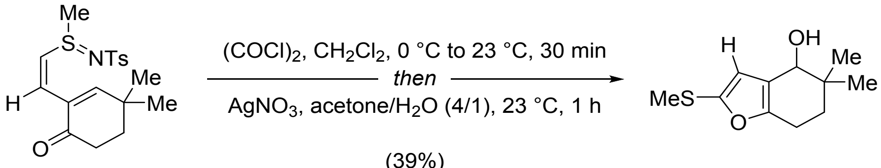

18 **29**

To a solution of sulfilimine **19** (25 mg, 68 µmol, 1 equiv) in dichloromethane (0.5 mL) was added a solution of oxalyl chloride (2.0 M in dichloromethane, 51 µL, 0.13 mmol, 1.5 equiv) at 0 °C. After 30 minutes, the solution was allowed to warm to 23 °C and the solvent was removed

under reduced pressure (23 °C bath temperature). The crude product was dissolved in acetone (0.5 mL) and an aqueous solution of silver nitrate (0.50 M, 0.14 mL, 68 µmol, 1.0 equiv) was added dropwise at 23 °C. After one hour, diethyl ether (5 mL) and water (5 mL) were added and the layers were separated. The aqueous layer was extracted with diethyl ether (2 × 5 mL), the combined organic layers were dried over sodium sulfate, the dried solution was filtered and the filtrate was concentrated under reduced pressure. The crude product was purified by flash-column chromatography on silica gel (20% to 30% diethyl ether in *n*-pentane) to furnish furan **29** (5.7 mg, 39%) as a colorless solid. The obtained analytical data were in full agreement with those reported in the literature.[6]

TLC (30% diethyl ether in petroleum ether): R_f = 0.26 (UV, $KMnO_4$, CAM).

Furan 30

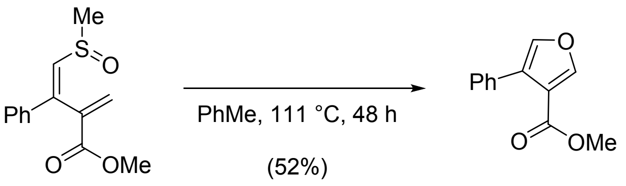

28 **30**

A solution of sulfoxide **28** (25 mg, 0.20 mmol, 1 equiv) in toluene (4.0 mL) was heated to 111 °C. After 48 hours, the solution was allowed to cool to 23 °C and the solvent was removed under reduced pressure. The crude product was purified by flash-column chromatography on silica gel (10% diethyl ether in *n*-pentane) to furnish furan **30** (11 mg, 52%) as a colorless oil.

TLC (10% diethyl ether in petroleum ether): R_f = 0.36 (UV, $KMnO_4$, CAM).

^{1}H NMR (400 MHz, $CDCl_3$): δ = 8.08 (d, *J* = 1.8 Hz, 1H), 7.48 (d, *J* = 1.8 Hz, 2H), 7.47 – 7.45 (m, 1H), 7.42 – 7.32 (m, 3H), 3.78 (s, 3H).

^{13}C NMR (101 MHz, $CDCl_3$): δ = 163.6, 149.7, 141.6, 130.8, 129.3, 128.2, 127.8, 126.9, 117.7, 51.6.

IR (Diamond-ATR, $CDCl_3$): $\tilde{\upsilon}_{max}$ = 1729 (*vs*), 1538 (*w*), 1436 (*w*), 1277 (*m*), 1148 (*m*), 1122 (*m*), 1059 (*w*), 878 (*w*), 761 (*m*), 697 (*w*).

HRMS (ESI) calc. for $C_{12}H_{10}NaO_3$ $[M+Na]^+$: 225.0522; found: 225.0518.

4.4 Additional Mechanistic Experiments

Iron(II)-catalyzed pyrrole formation (5a→9a)

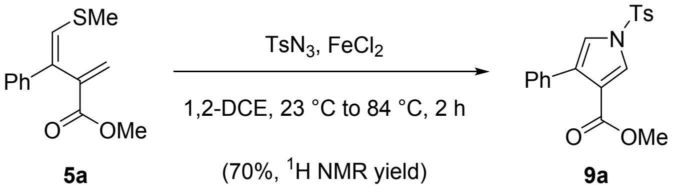

To a solution of 1,3-diene **5a** (12 mg, 50 µmol, 1 equiv) in 1,2-dichloroethane (0.80 mL) were added iron(II)-chloride (6.3 mg, 50 µmol, 1.0 equiv) and *p*-toluenesulfonyl azide solution (11% in toluene, 0.20 mL, 0.10 mmol, 2.0 equiv) at 23 °C. After one hour, the reaction mixture was heated to 84 °C. After one hour, the reaction mixture was allowed to cool to 23 °C and saturated aqueous sodium bicarbonate solution (10 mL) and dichloromethane (10 mL) were added. The layers were separated and the aqueous layer was extracted with dichloromethane (3 × 5 mL). The combined organic layers were dried over sodium sulfate, the dried solution was filtered and the filtrate was purified over a plug of silica. Nitromethane (2.7 µL, 50 µmol, 1.0 equiv) was added as an internal ^{1}H NMR standard to determine the yield of pyrrole **9a** (70%).

Iodine(III)-mediated pyrrole formation (5a→9a)

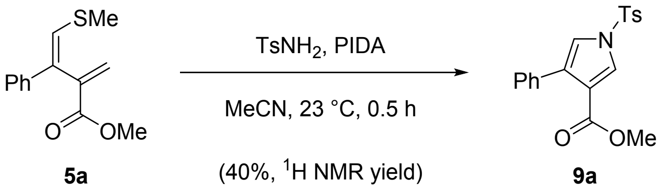

To a solution of 1,3-diene **5a** (12 mg, 50 µmol, 1 equiv) in acetonitrile (1.0 mL) were added *p*-toluenesulfonamide (25 mg, 0.15 mmol, 3.0 equiv) and (diacetoxyiodo)benzene (48 mg, 0.15 mmol, 3.0 equiv) at 23 °C. After 30 minutes, saturated aqueous sodium bicarbonate solution (5 mL), saturated aqueous sodium thiosulfate solution (5 mL) and ethyl acetate (10 mL) were added. The layers were separated and the aqueous layer was extracted with ethyl acetate (2 × 5 mL). The combined organic layers were dried over sodium sulfate, the dried solution was filtered and the filtrate concentrated. Nitromethane (2.7 µL, 50 µmol, 1.0 equiv) was added as an internal ^{1}H NMR standard to determine the yield of pyrrole **9a** (40%).

Competition Experiment

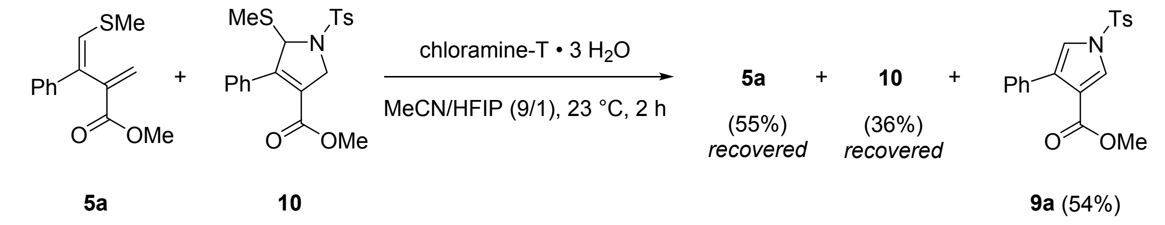

To a solution of 1,3-diene **5a** (4.9 mg, 21 μmol, 1 equiv) and 2,5-dihydropyrrole **10** (8.4 mg, 21 μmol, 1 equiv) in acetonitrile (0.36 mL) and hexafluoroisopropanol (0.04 mL) was added chloramine-T (6.0 mg, 21 μmol, 1.0 equiv) at 23 °C. After one hour, saturated aqueous sodium thiosulfate solution (5 mL) and ethyl acetate (5 mL) were added. The layers were separated and the aqueous layer was extracted with ethyl acetate (2 × 5 mL). The combined organic layers were dried over sodium sulfate, the dried solution was filtered and the filtrate was concentrated under reduced pressure. Methyl phenyl sulfone (4.9 mg, 31 μmol, 1.5 equiv) was added as an internal ^{1}H NMR standard to the crude reaction mixture to determine the yield of pyrrole **9a** (54%) as well as unreacted 2,5-dihydropyrrole **10** (36%) and 1,3-diene **5a** (55%).

Conclusion: Competing *S*-imidiation between 1,3-diene **5a** and 2,5-dihydropyrrole **10** indicating the requirement of two equivalents of chloramine-T to achieve full conversion of **5a** (Compare to chloramine-T triggered elimination).

Sulfilimine S-59

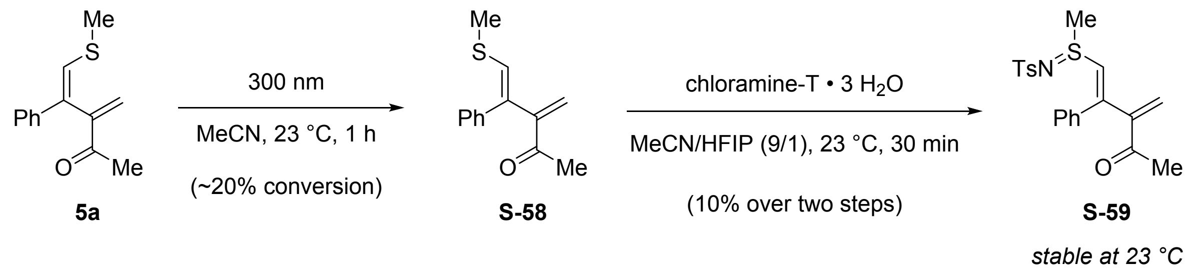

1,3-Diene **5a** (121 mg, 554 μmol, 1 equiv) was placed in a quartz-tube, dissolved in degassed acetonitrile (11 mL) and irradiated with UV light (300 nm) in a Rayonet photochemical reactor. After one hour, the solvent was removed under reduced pressure to give a mixture of 1,3-dienes **5a** and **S-58** (ratio = 4:1, 20% conversion based on ^{1}H NMR analysis).

To a solution of 1,3-dienes **5a** and **S-58** in acetonitrile (5.0 mL) and hexafluoroisopropanol (0.5 mL) was added chloramine-T trihydrate (319 mg, 1.11 mmol, 2.00 equiv) portionwise over ten minutes at 23 °C. After 30 minutes saturated aqueous sodium thiosulfate solution (25 mL) and ethyl acetate (25 mL) were added. The layers were separated and the aqueous phase was extracted with ethyl acetate (2 × 25 mL), the combined organic layers were dried over

sodium sulfate, the dried solution was filtered and the filtrate was concentrated under reduced pressure. The crude product was purified by flash-column chromatography on silica gel (10% to 100% ethyl acetate in cyclohexane) to yield sulfilimine **S-59** (21 mg, 10%) as a colorless oil and pyrrole **9a** (132 mg, 70%) as a pale-yellow oil.

TLC (50% ethyl acetate in dichloromethane): R_f = 0.12 (UV, $KMnO_4$, CAM).

^{1}H NMR ($CDCl_3$, 400 MHz): δ = 7.68 – 7.63 (m, 2H), 7.43 – 7.33 (m, 3H), 7.18 (d, *J* = 8.0 Hz, 2H), 7.11 – 7.04 (m, 2H), 6.61 (s, 1H), 6.23 (s, 1H), 5.76 (s, 1H), 2.77 (s, 3H), 2.39 (s, 3H), 2.30 (s, 3H).

^{13}C NMR ($CDCl_3$, 101 MHz): δ = 197.4, 151.5, 147.6, 141.7, 141.6, 135.1, 130.7, 129.9, 129.3, 128.9, 128.8, 127.8, 126.5, 36.7, 27.6, 21.5.

IR (Diamond-ATR, CH_2Cl_2): $\tilde{\upsilon}_{max}$ = 1684 (*w*), 1276 (*m*), 1137 (*s*), 1087 (*m*), 1021 (*w*), 989 (*w*), 938 (*s*), 815 (*w*), 728 (*m*), 699 (*m*).

HRMS (ESI) calc. for $C_{20}H_{22}NO_3S_2$ $[M+H]^+$: 388.1036; found: 388.1033.

Conclusion: Sulfilimine **S-59** could be isolated under standard reaction conditions (23 °C) since no 6π-electrocyclization/ring-contraction sequence is conceivable with respect to its *trans*-configuration.

Sulfilimine S-61

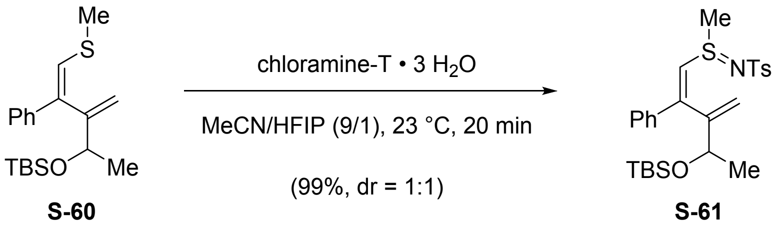

To a solution of 1,3-diene **S-60** (33.5 mg, 100 µmol, 1 equiv) in acetonitrile and hexafluoroisopropanol (9:1, 1.0 mL) was added chloramine-T trihydrate (57.5 mg, 200 µmol, 2.00 equiv) in one portion at 23 °C. After 20 minutes saturated aqueous sodium thiosulfate solution (5 mL) and ethyl acetate (5 mL) were added. The layers were separated and the aqueous phase was extracted with ethyl acetate (2 × 10 mL), the combined organic layers were dried over sodium sulfate, the dried solution was filtered and the filtrate was concentrated under reduced pressure. The crude product was purified by flash-column chromatography on silica gel (10% to 20% ethyl acetate and 1% toluene in dichloromethane) to yield sulfilimine **S-61** (dr = 1:1, 50 mg, 99%) as a colorless oil.

TLC (20% ethyl acetate and 1% toluene in dichloromethane): R_f = 0.27 (UV, $KMnO_4$, CAM).

^{1}H NMR ($CDCl_3$, 400 MHz): δ = 7 7.99 – 7.75 (m, 4H), 7.51 – 7.40 (m, 6H), 7.39 – 7.31 (m, 4H), 7.30 – 7.22 (m, 4H), 6.58 (s, 1H), 6.52 (s, 1H), 5.81 (t, *J* = 1.8 Hz, 1H), 5.68 (t, *J* = 1.7 Hz, 1H), 5.03 (q, *J* = 1.7 Hz, 2H), 4.31 – 4.22 (m, 1H), 4.14 – 4.06 (m, 1H), 2.91 (d, *J* = 6.8 Hz,

6H), 2.43 (d, J = 6.5 Hz, 6H), 1.04 (d, J = 6.4 Hz, 3H), 0.98 (d, J = 6.5 Hz, 3H), 0.94 (s, 9H), 0.92 (s, 9H), 0.08 (s, 3H), 0.07 (s, 3H), 0.01 (s, 3H), –0.02 (s, 3H).

^{13}C NMR ($CDCl_3$, 101 MHz): δ = 155.8, 155.1, 149.8, 148.1, 142.1, 142.1, 141.6, 141.6, 136.0, 135.5, 130.7, 130.7, 129.3, 129.1, 128.9, 127.7, 127.5, 126.6, 126.5, 124.9, 124.2, 118.1, 117.9, 69.2, 68.4, 38.7, 37.6, 25.9, 25.9, 23.3, 22.5, 21.5, 18.2, 18.2, –4.7, –4.8, –4.8, –4.9.

IR (Diamond-ATR, CH_2Cl_2): $\tilde{\upsilon}_{max}$ = 2927 (*w*), 1281 (*w*), 1259 (*w*), 1140 (*s*), 1111 (*m*), 1090 (*m*), 1022 (*w*), 940 (*m*), 829 (*m*), 572 (*w*).

HRMS (ESI) calc. for $C_{26}H_{38}NO_3S_2Si$ $[M+H]^+$: 504.2057; found: 504.2057.

Pyrrole S-62

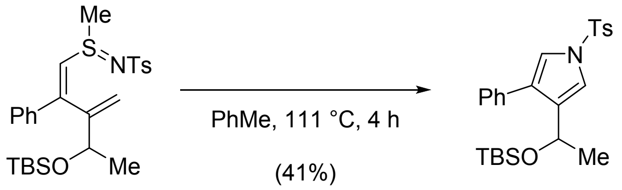

S-61 **S-62**

Sulfilimine **S-61** (38 mg, 75 µmol, 1 equiv) was dissolved in toluene (2.0 mL) and heated to 111 °C. After four hours, the solvent was removed under reduced pressure. Purification by flash-column chromatography on silica gel (20% ethyl acetate in cyclohexane) furnished pyrrole **S-62** (14 mg, 41%) as a colorless oil.

TLC (40% ethyl acetate in cyclohexane): R_f = 0.61 (UV, $KMnO_4$, CAM).

^{1}H NMR (400 MHz, $CDCl_3$): δ = 7.79 – 7.73 (m, 2H), 7.38 – 7.27 (m, 7H), 7.18 – 7.10 (m, 2H), 4.94 (q, *J* = 6.3 Hz, 1H), 2.41 (s, 3H), 1.22 (d, *J* = 6.3 Hz, 3H), 0.84 (s, 9H), –0.04 (s, 3H), – 0.10 (s, 3H).

^{13}C NMR (101 MHz, $CDCl_3$): δ = 145.0, 136.3, 134.2, 133.9, 130.1, 128.6, 128.5, 128.5, 127.2, 127.1, 118.7, 118.5, 64.9, 26.0, 25.5, 21.8, 18.3, –4.7, –4.7.

IR (Diamond-ATR, $CDCl_3$): $\tilde{\upsilon}_{max}$ = 1372 (*m*), 1172 (*s*), 1092 (*m*), 1068 (*s*), 945 (*w*), 832 (*m*), 811 (*w*), 775 (*m*), 671 (*s*), 594 (*m*).

HRMS (ESI) calc. for $C_{25}H_{33}NNaO_3SSi$ $[M+Na]^+$: 478.1843; found: 478.1840.

Side-Selective Thiomethylation (10→11)

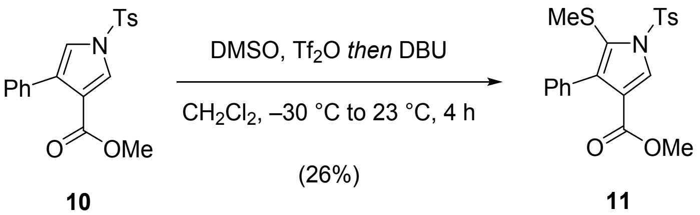

10 **11**

Pyrrole **11** was synthesized following a modified procedure reported by Procter:[7] To a solution of dimethyl sulfoxide (12 µL, 0.17 mmol, 1.2 equiv) in dichloromethane (0.5 mL) was added trifluoromethanesulfonic anhydride (29 µL, 0.17 mmol, 1.2 equiv) at –30 °C. Upon addition, a solution of pyrrole **10** (50 mg, 0.14 mmol, 1 equiv) in dichloromethane (0.5 mL) was added. Upon complete addition, the reaction mixture was allowed to warm to 23 °C. After one hour, DBU (45 µL, 0.30 mmol, 2.1 equiv) was added. After three hours, water (5 mL) and dichloromethane (5 mL) were added, the layers were separated and the aqueous phase extracted with dichloromethane (2 × 5 mL). The combined organic layer were dried over sodium sulfate, the dried solution was filtered and the solvent removed under reduced pressure. The crude product was purified by flash-column chromatography on silica gel (10% ethyl acetate in petroleum ether) to furnish **11** (15 mg, 26%) as a pale-yellow oil.

TLC (50% diethyl ether in petroleum ether): R_f = 0.45 (UV, $KMnO_4$).

^{1}H NMR ($CDCl_3$, 400 MHz): δ = 8.21 (s, 1H), 7.98 – 7.91 (m, 2H), 7.38 – 7.31 (m, 5H), 7.26 – 7.23 (m, 2H), 3.69 (s, 3H), 2.44 (s, 3H), 2.02 (s, 3H).

^{13}C NMR ($CDCl_3$, 101 MHz): δ = 163.4, 146.0, 137.2, 135.0, 132.8, 130.2, 130.0, 129.9, 128.7, 127.8, 127.7, 124.2, 117.0, 51.5, 21.9, 21.5.

IR (Diamond-ATR, CH_2Cl_2): $\tilde{\upsilon}_{max}$ = 1728 (*m*), 1375 (*w*), 1270 (*w*), 1190 (*m*), 1176 (*s*), 1138 (*s*), 1089 (*m*), 673 (*s*), 563 (*s*), 540 (*w*).

HRMS (ESI) calc. for $C_{20}H_{19}KNO_4S_2$ $[M+K]^+$: 440.0387; found: 440.0363.

5 Synthesis of 1,3-Dienes and Reagents

5.1 Literature-known Compounds

For procedures and analytical data, see: *J. Am. Chem. Soc.* **2021**, *143*, 1216–1223 (Ref 6).

A. 1,3-Dienes

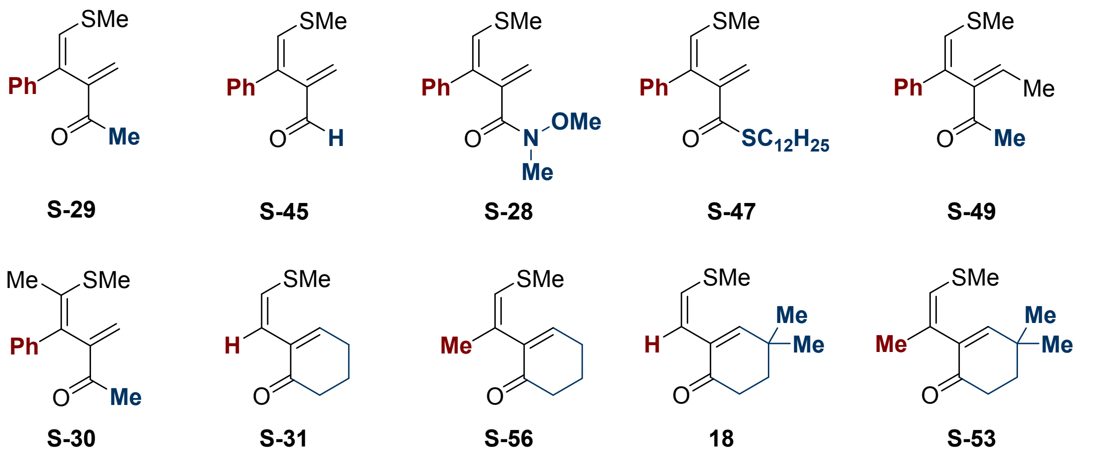

B. 2,5-Dihydrothiophenes

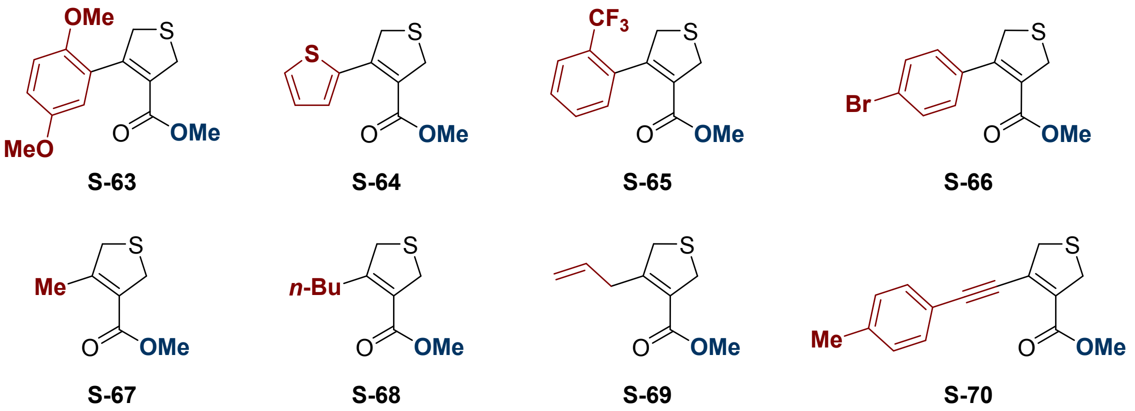

5.2 1,3-Dienes

Triflate S-3

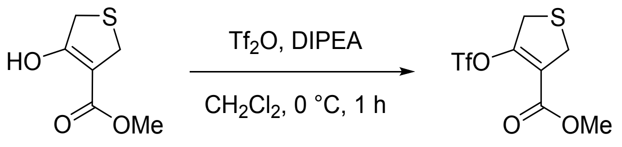

S-71 (92%) **S-3**

2,5-Dihydrothiophene **S-71** (7.27 g, 45.4 mmol, 1 equiv) was dissolved in dichloromethane (160 mL, HPLC grade) under argon. *N*,*N*-diisopropylethylamine (10.0 mL, 59.0 mmol, 1.30 equiv) was added and the solution was cooled to 0 °C. After 30 minutes, trifluoromethanesulfonic anhydride (9.20 mL, 54.5 mmol, 1.20 equiv) was added dropwise.

After complete addition, the reaction mixture was allowed to warm to 23 °C. After 30 minutes, the solvent was removed under reduced pressure and the crude product was purified by flash-column chromatography on silica gel (10% diethyl ether in *n*-pentane) to yield triflate **S-3** (12.1 g, 92%) as a pale-yellow oil. The obtained analytical data were in full agreement with those reported in the literature.[8]

TLC (10% diethyl ether in petroleum ether): R_f = 0.38 (UV, $KMnO_4$).

2,5-Dihydrothiophene S-70

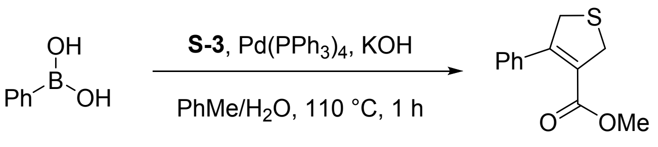

S-72 **S-73**

Following GP2, triflate **S-3** (8.50 g, 29.1 mmol, 1 equiv) and phenylboronic acid (**S-72**, 4.26 g, 34.9 mmol, 1.20 equiv) were converted into 2,5-dihydrothiophene **S-73**. Purification by flash-column chromatography on silica gel (10% to 20% diethyl ether in petroleum ether) furnished **S-73** (5.56 g, 87%) as a colorless solid. The obtained analytical data were in full agreement with those reported in literature.[6]

TLC (10% diethyl ether in petroleum ether): R_f = 0.42 (UV, $KMnO_4$).

1,3-Diene 5a

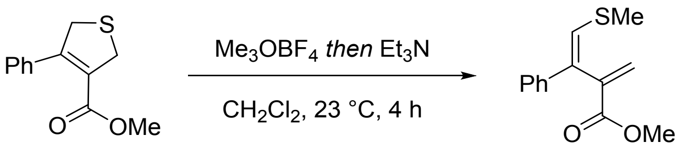

S-73 **5a**

Following GP3 (3.5 hours for *S*-methylation), dihydrothiophene **S-73** (2.11 g, 9.10 mmol, 1 equiv) was converted into 1,3-diene **5a**. Purification by flash-column chromatography on silica gel (20% diethyl ether in *n*-pentane) furnished **5a** (1.94 g, 91%) as a pale-yellow oil. The obtained analytical data were in full agreement with those reported in literature.[6]

TLC (10% diethyl ether in petroleum ether): R_f = 0.22 (UV, $KMnO_4$).

^{1}H NMR ($CDCl_3$, 400 MHz): δ = 7.33 – 7.27 (m, 4H), 7.26 – 7.19 (m, 1H), 6.64 (d, *J* = 1.5 Hz, 1H), 6.60 (s, 1H), 5.84 (d, *J* = 1.5 Hz, 1H), 3.70 (s, 3H), 2.39 (s, 3H).

^{13}C NMR ($CDCl_3$, 101 MHz): δ = 166.7, 139.8, 138.7, 134.5, 131.1, 129.7, 128.6, 127.2, 125.6, 52.3, 17.8.

IR (Diamond-ATR, $CDCl_3$): $\tilde{\upsilon}_{max}$ = 1718 (*s*), 1434 (*w*), 1250 (*s*), 1192 (*w*), 1134 (*s*), 989 (*w*), 956 (*w*), 820 (*m*), 756 (*vs*), 693 (*m*).

HRMS (ESI) calc. for $C_{13}H_{14}NaO_2S$ $[M+Na]^+$: 257.0607; found: 253.0580.

1,3-Diene 5b

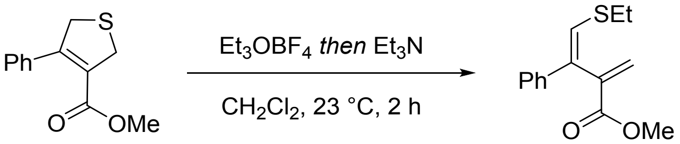

S-73 (98%) **5b**

To a solution of dihydrothiophene **S-73** (100 mg, 454 µmol, 1 equiv) in dichloromethane (2.0 mL) was added triethyloxonium tetrafluoroborate (111 mg, 567 µmol, 1.25 equiv) in one portion at 23 °C. After 1.5 hours, triethylamine (190 µL, 1.36 mmol, 3.00 equiv) was added dropwise. After 30 minutes, the solvent was removed under reduced pressure and the crude product was purified by flash-column chromatography on silica gel (toluene) to furnish 1,3-diene **5b** (110 mg, 98%) as a pale-yellow oil.

TLC (50% diethyl ether in petroleum ether): R_f = 0.55 (UV, $KMnO_4$).

^{1}H NMR ($CDCl_3$, 400 MHz): δ = 7.30 – 7.26 (m, 4H), 7.24 – 7.20 (m, 1H), 6.63 (s, 1H), 6.62 (d, *J* = 1.5 Hz, 1H), 5.82 (d, *J* = 1.5 Hz, 1H), 3.69 (s, 3H), 2.80 (q, *J* = 7.4 Hz, 2H), 1.35 (t, *J* = 7.4 Hz, 3H).

^{13}C NMR ($CDCl_3$, 101 MHz): δ = 166.8, 140.0, 138.9, 134.9, 131.0, 128.6, 128.0, 127.1, 125.7, 52.3, 28.7, 15.6.

IR (Diamond-ATR, CH_2Cl_2): $\tilde{\upsilon}_{max}$ = 1720 (*s*), 1436 (*w*), 1252 (*m*), 1193 (*w*), 1154 (*m*), 1136 (*m*), 977 (*w*), 823 (*w*), 759 (*m*), 696 (*w*).

HRMS (ESI) calc. for $C_{14}H_{16}NaO_2S$ $[M+Na]^+$: 271.0763; found: 271.0753.

1,3-Diene 5c

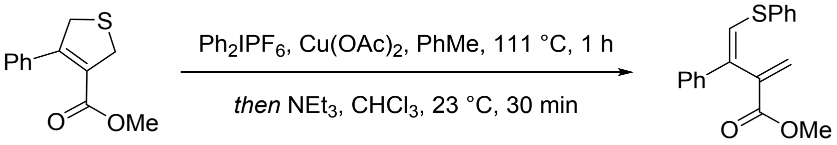

S-73 (94%) **5c**

To a solution of 2,5-dihydrothiophene **S-73** (100 mg, 454 µmol, 1 equiv) in toluene (1.0 mL) under argon were added copper (II) acetate (8.4 mg, 45 µmol, 10 mol%) and diphenyliodonium hexafluorophosphate (199 mg, 454 mmol, 1.00 equiv) at 23 °C. Upon complete addition, the reaction mixture was heated to 111 °C. After one hour, the reaction mixture was allowed to cool to 23 °C and the solvent was removed under reduced pressure. The residue was dissolved in chloroform (2.5 mL) and triethylamine (150 µL, 1.08 mmol, 2.37 equiv) was added dropwise at 23 °C. After 30 minutes, the solvent was removed under reduced pressure and

the crude product was purified by flash-column chromatography on silica gel (toluene) to furnish 1,3-diene **5c** (126 mg, 94%) as a pale-yellow oil.

TLC (toluene): R_f = 0.43 (UV, $KMnO_4$).

^{1}H NMR ($CDCl_3$, 400 MHz): δ = 7.41 – 7.38 (m, 2H), 7.33 – 7.149 (m, 8H), 6.81 (d, *J* = 0.9 Hz, 1H), 6.64 (m, 1H), 5.84 (d, *J* = 1.3 Hz, 1H), 3.66 (s, 3H).

^{13}C NMR ($CDCl_3$, 101 MHz): δ = 166.6, 139.6, 138.7, 137.6, 135.9, 131.2, 129.7, 129.2, 128.6, 127.7, 127.1, 126.1, 126.0, 52.3.

IR (Diamond-ATR, CH_2Cl_2): $\tilde{\upsilon}_{max}$ 1722 (*s*), 1581 (*w*), 1479 (*w*), 1439 (*m*), 1249 (*m*), 1192 (*m*), 1137 (*m*), 820 (*w*), 760 (*m*), 742 (*m*).

HRMS (ESI) calc. for $C_{18}H_{16}NaO_2S$ $[M+Na]^+$: 319.0763; found: 319.0751.

2,5-Dihydrothiophene S-75

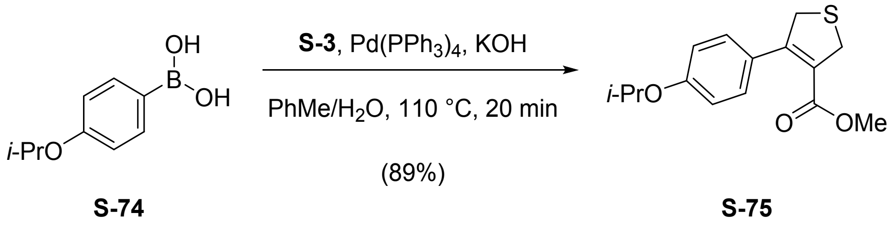

Following GP2 (20 minutes reaction time), triflate **S-3** (146 mg, 500 µmol, 1 equiv) and boronic acid **S-74** (108 mg, 600 µmol, 1.20 equiv) were converted into 2,5-dihydrothiophene **S-75**. Purification by flash-column chromatography on silica gel (10% to 20% diethyl ether in *n*-pentane) furnished **S-75** (123 mg, 89%) as a pale-yellow oil.

TLC (20% diethyl ether in petroleum ether): R_f = 0.40 (UV, $KMnO_4$).

^{1}H NMR ($CDCl_3$, 400 MHz): δ = 7.21 – 7.13 (m, 2H), 6.89 – 6.81 (m, 2H), 4.56 (hept, *J* = 6.1 Hz, 1H), 4.15 (q, *J* = 2.2 Hz, 4H), 3.62 (s, 3H), 1.34 (d, *J* = 6.1 Hz, 6H).

^{13}C NMR ($CDCl_3$, 101 MHz): δ = 165.4, 158.4, 151.6, 129.1, 127.6, 126.7, 115.2, 70.0, 51.7, 44.7, 40.3, 22.2.

IR (Diamond-ATR, CH_2Cl_2): $\tilde{\upsilon}_{max}$ = 1725 (*m*), 1606 (*m*), 1508 (*m*), 1335 (*w*), 1285 (*w*), 1245 (*s*), 1229 (*s*), 1185 (*m*), 1118 (*m*), 832 (*w*).

HRMS (ESI) calc. for $C_{15}H_{18}NaO_3S$ $[M+Na]^+$: 301.0869; found: 301.0852.

1,3-Diene S-10

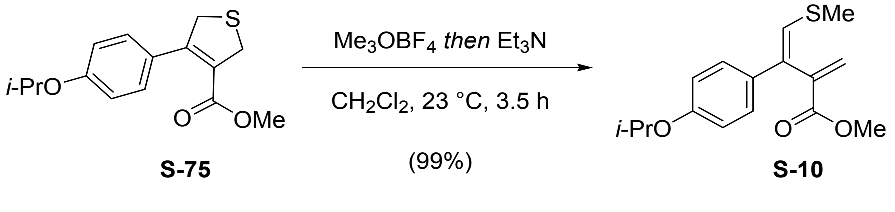

Following GP3 (three hours for *S*-methylation and 15 minutes for ring-opening), dihydrothiophene **S-75** (84 mg, 0.30 mmol) was converted into 1,3-diene **S-10**. Purification by flash-column chromatography on silica gel (dichloromethane) furnished **S-10** (87 mg, 99%) as a yellow oil.

TLC (dichloromethane): R_f = 0.92 (UV, $KMnO_4$).

^{1}H NMR ($CDCl_3$, 400 MHz): δ = 7.21 – 7.15 (m, 2H), 6.83 – 6.77 (m, 2H), 6.59 (d, *J* = 1.5 Hz, 1H), 6.45 (s, 1H), 5.79 (d, *J* = 1.5 Hz, 1H), 4.52 (hept, *J* = 6.1 Hz, 1H) 3.70 (s, 3H), 2.35 (s, 3H), 1.32 (d, *J* = 6.1 Hz, 6H).

^{13}C NMR ($CDCl_3$, 101 MHz): δ = 166.9, 157.3, 139.0, 134.4, 132.3, 130.8, 127.3, 126.8, 115.8, 70.0, 52.3, 22.2, 17.7.

IR (Diamond-ATR, CH_2Cl_2): $\tilde{\nu}_{max}$ = 1721 (*s*), 1606 (*w*), 1507 (*s*), 1435 (*w*), 1284 (*w*), 1247 (*s*), 1185 (*w*), 1155 (*w*), 1136 (*m*), 954 (*w*).

HRMS (ESI) calc. for $C_{16}H_{20}NaO_3S$ $[M+Na]^+$: 315.1025; found: 315.1018.

1,3-Diene S-11

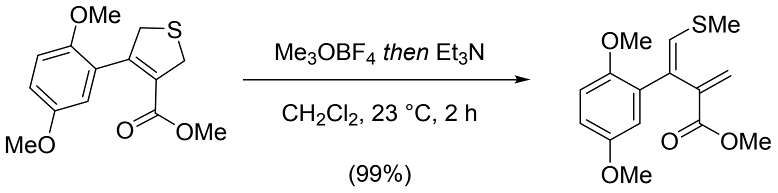

S-63 **S-11**

Following GP3 (one hour for *S*-methylation), 2,5-dihydrothiophene **S-63** (84 mg, 0.30 mmol) was converted into 1,3-diene **S-11**. Purification by flash-column chromatography on silica gel (dichloromethane) furnished **S-11** (87 mg, 99%) as a yellow oil.

TLC (dichloromethane): R_f = 0.57 (UV, $KMnO_4$).

^{1}H NMR ($CDCl_3$, 400 MHz): δ = 6.81 – 6.70 (m, 3H), 6.63 (s, 1H), 6.45 (d, *J* = 1.4 Hz, 1H), 5.71 (d, *J* = 1.4 Hz, 1H), 3.76 (s, 3H), 3.71 (s, 6H), 2.35 (s, 3H).

^{13}C NMR ($CDCl_3$, 101 MHz): δ = 166.7, 153.7, 151.1, 139.6, 132.8, 131.6, 131.0, 128.7, 115.7, 113.1, 112.7, 56.5, 55.8, 52.2, 17.9.

IR (Diamond-ATR, CH_2Cl_2): $\tilde{\nu}_{max}$ = 1721 (*s*), 1494 (*s*), 1464 (*w*), 1436 (*w*), 1278 (*m*), 1254 (*s*), 1225 (*m*), 1201 (*m*), 1137 (*m*), 1047 (*m*).

HRMS (ESI) calc. for $C_{15}H_{19}O_4S$ $[M+H]^+$: 295.0999; found: 295.0989.

1,3-Diene S-12

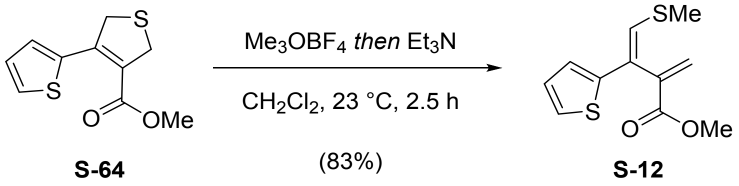

Following GP3 (1.5 hours for *S*-methylation), 2,5-dihydrothiophene **S-64** (68 mg, 0.30 mmol) was converted into 1,3-diene **S-12**. Purification by flash-column chromatography on silica gel (dichloromethane) furnished **S-12** (60 mg, 83%) as a yellow oil.

TLC (dichloromethane): R_f = 0.78 (UV, $KMnO_4$).

^{1}H NMR ($CDCl_3$, 400 MHz): δ = 7.11 (dd, *J* = 5.1, 1.2 Hz, 1H), 6.91 (dd, *J* = 5.1, 3.6 Hz, 1H), 6.75 (dd, *J* = 3.6, 1.2 Hz, 1H), 6.60 (d, *J* = 1.4 Hz, 1H), 6.58 (s, 1H), 5.86 (d, *J* = 1.4 Hz, 1H), 3.74 (s, 3H), 2.37 (s, 3H).

^{13}C NMR ($CDCl_3$, 101 MHz): δ = 166.3, 144.1, 138.1, 131.2, 128.8, 128.3, 127.6, 123.9, 123.2, 52.5, 17.6.

IR (Diamond-ATR, CH_2Cl_2): $\tilde{\upsilon}_{max}$ = 1721 (*s*), 1434 (*w*), 1318 (*w*), 1254 (*m*), 1232 (*w*), 1199 (*w*), 1152 (*w*), 1135 (*m*), 817 (*w*), 698 (*w*).

HRMS (ESI) calc. for $C_{11}H_{12}NaO_2S_2$ $[M+Na]^+$: 263.0171; found: 263.0168.

2,5-Dihydrothiophene S-77

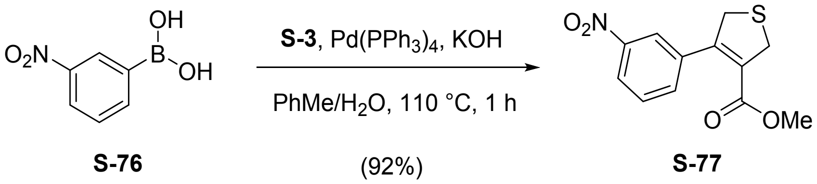

Following GP2, triflate **S-3** (146 mg, 500 µmol, 1 equiv) and boronic acid **S-76** (103 mg, 600 µmol, 1.20 equiv) were converted into 2,5-dihydrothiophene **S-77**. Purification by flash-column chromatography on silica gel (20% ethyl acetate in cyclohexane) furnished **S-77** (123 mg, 92%) as a yellow solid.

TLC (50% ethyl acetate in cyclohexane): R_f = 0.62 (UV, $KMnO_4$).

^{1}H NMR ($CDCl_3$, 400 MHz): δ = 8.21 (dt, *J* = 7.3, 2.1 Hz, 1H), 8.13 (ddd, *J* = 2.3, 1.5, 0.6 Hz, 1H), 7.58 – 7.53 (m, 2H), 4.22 – 4.15 (m, 4H), 3.62 (s, 3H).

^{13}C NMR ($CDCl_3$, 101 MHz): δ = 164.2, 149.8, 148.2, 137.8, 133.8, 130.2, 129.3, 123.4, 122.8, 52.0, 44.8, 40.0.

IR (Diamond-ATR, CH_2Cl_2): $\tilde{\upsilon}_{max}$ = 2918 (*w*), 1721 (*m*), 1528 (*s*), 1434 (*w*), 1350 (*m*), 1317 (*w*), 1281 (*w*), 1236 (*m*), 1209 (*m*), 1125 (*w*).

HRMS (ESI) calc. for $C_{12}H_{11}NNaO_4S$ $[M+Na]^+$: 288.0301; found: 288.0292.

MP = 93 – 95 °C.

1,3-Diene S-13

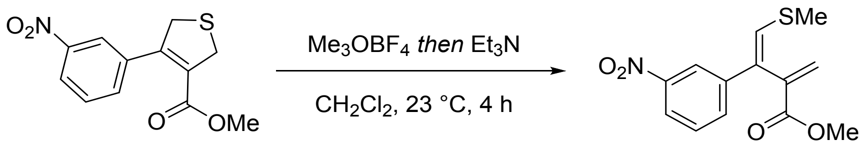

S-77 **S-13**

Following GP3 (three hours for *S*-methylation), 2,5-dihydrothiophene **S-77** (80 mg, 0.30 mmol) was converted into 1,3-diene **S-13**. Purification by flash-column chromatography on silica gel (dichloromethane) furnished **S-13** (67 mg, 80%) as a yellow solid.

TLC (20% ethyl acetate in cyclohexane): R_f = 0.31 (UV, $KMnO_4$).

^{1}H NMR ($CDCl_3$, 400 MHz): δ = 8.14 (t, *J* = 2.0 Hz, 1H), 8.06 (ddd, *J* = 8.0, 2.2, 1.1 Hz, 1H), 7.54 (ddd, *J* = 7.9, 1.9, 1.1 Hz, 1H), 7.45 (t, *J* = 8.0 Hz, 1H), 6.77 (s, 1H), 6.73 (d, *J* = 1.3 Hz, 1H), 5.93 (d, *J* = 1.3 Hz, 1H), 3.70 (s, 3H), 2.44 (s, 3H).

^{13}C NMR ($CDCl_3$, 101 MHz): δ = 166.1, 148.7, 141.4, 137.6, 133.6, 132.5, 131.7, 131.4, 129.5, 121.7, 120.2, 52.5, 17.8.

IR (Diamond-ATR, CH_2Cl_2): $\tilde{\upsilon}_{max}$ = 1717 (*m*), 1524 (*s*), 1435 (*w*), 1347 (*s*), 1319 (*w*), 1255 (*w*), 1139 (*m*), 822 (*w*), 803 (*w*), 740 (*w*).

HRMS (ESI) calc. for $C_{13}H_{13}NNaO_4S$ $[M+Na]^+$: 302.0457; found: 302.0454.

MP: 94 – 95 °C.

1,3-Diene S-14

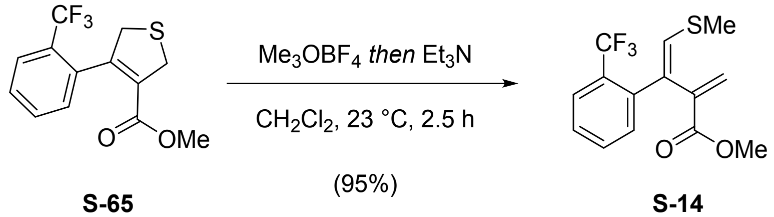

S-65 **S-14**

Following GP3 (15 minutes for ring-opening), 2,5-dihydrothiophene **S-65** (87 mg, 0.30 mmol) was converted into 1,3-diene **S-14**. Purification by flash-column chromatography on silica gel (dichloromethane) furnished **S-14** (86 mg, 95%) as a colorless oil.

TLC (dichloromethane): R_f = 0.75 (UV, $KMnO_4$).

^{1}H NMR ($CDCl_3$, 400 MHz): δ = 7.66 – 7.62 (m, 1H), 7.50 – 7.45 (m, 1H), 7.37 (m, 2H), 6.48 (d, *J* = 1.1 Hz, 1H), 6.30 (s, 1H), 5.76 (d, *J* = 1.0 Hz, 1H), 3.68 (s, 3H), 2.34 (s, 3H).

^{13}C NMR ($CDCl_3$, 101 MHz): δ = 166.6, 140.5, 138.8, 134.4 (q, *J* = 2 Hz), 131.9, 131.8, 131.5, 130.1, 128.3 (q, *J* = 30 Hz), 127.4, 126.4 (q, *J* = 5 Hz), 124.3 (q, *J* = 274 Hz), 52.2, 18.0.

^{19}F NMR ($CDCl_3$, 376 MHz) δ = –57.0.

IR (Diamond-ATR, CH_2Cl_2): $\tilde{\upsilon}_{max}$ = 1724 (*m*), 1437 (*w*), 1312 (*s*), 1264 (*w*), 1169 (*w*), 1129 (*m*), 1108 (*m*), 1063 (*w*), 1035 (*w*), 769 (*w*).

HRMS (ESI) calc. for $C_{14}H_{13}F_3NaO_2S$ $[M+Na]^+$: 325.0481; found: 325.0473.

2,5-Dihydrothiophene S-79

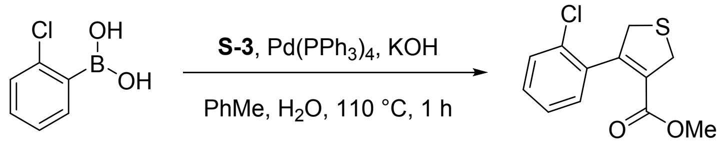

S-78 (88%) **S-79**

Following GP2, triflate **S-3** (500 mg, 1.71 mmol, 1 equiv) and (2-chlorophenyl)boronic acid (**S-78**, 321 mg, 2.05 mmol, 1.20 equiv) were converted into 2,5-dihydrothiophene **S-79**. Purification by flash-column chromatography on silica gel (10% to 20% diethyl ether in *n*-pentane) furnished **S-79** (426 mg, 88%) as a colorless oil.

TLC (20% diethyl ether in *n*-pentane): R_f = 0.45 (UV, $KMnO_4$).

^{1}H NMR (400 MHz, $CDCl_3$): δ = 7.45 – 7.37 (m, 1H), 7.32 – 7.24 (m, 2H), 7.19 – 7.11 (m, 1H), 4.22 – 4.15 (m, 2H), 4.11 (d, *J* = 5.5 Hz, 2H), 3.57 (s, 3H).

^{13}C NMR (101 MHz, $CDCl_3$): δ = 163.8, 150.3, 135.6, 131.6, 130.4, 129.5, 129.3, 128.7, 126.7, 51.7, 43.9, 39.2.

IR (Diamond-ATR, $CDCl_3$): $\tilde{\upsilon}_{max}$ = 1725 (*s*), 1712 (*s*), 1434 (*m*), 1336 (*m*), 1316 (*m*), 1232 (*vs*), 1202 (*s*), 1065 (*m*), 763 (*m*), 747 (*m*).

HRMS (ESI) calc. for $C_{12}H_{11}ClNaO_2S$ $[M+Na]^+$: 277.0060; found: 277.0052.

1,3-Diene S-15

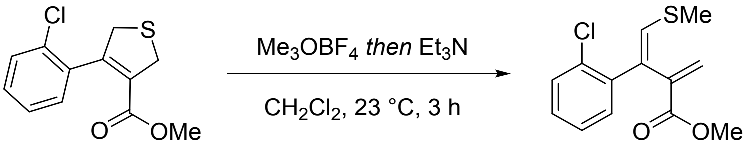

S-79 (75%) **S-15**

Following GP3, 2,5-dihydrothiophene **S-79** (330 mg, 1.30 mmol) was converted into 1,3-diene **S-15**. Purification by flash-column chromatography on silica gel (5% diethyl ether in *n*-pentane) furnished **S-15** (261 mg, 75%) as a colorless solid.

TLC (5% diethyl ether in *n*-pentane): R_f = 0.22 (UV, $KMnO_4$).

^{1}H NMR (400 MHz, $CDCl_3$): δ = 7.37 – 7.32 (m, 1H), 7.27 – 7.24 (m, 1H), 7.23 – 7.14 (m, 2H), 6.52 (d, *J* = 1.2 Hz, 1H), 6.41 (s, 1H), 5.80 (d, *J* = 1.2 Hz, 1H), 3.69 (s, 3H), 2.36 (s, 3H).

^{13}C NMR (101 MHz, $CDCl_3$): δ = 166.5, 139.7, 138.5, 134.6, 133.0, 132.2, 131.2, 130.1, 130.1, 128.6, 126.7, 52.2, 17.9.

IR (Diamond-ATR, $CDCl_3$): $\tilde{\upsilon}_{max}$ = 1721 (*vs*), 1434 (*m*), 1336 (*m*), 1318 (*m*), 1262 (v*s*), 1193 (*s*), 1138 (*m*), 1062 (*m*), 1036 (*m*), 755 (*s*).

HRMS (ESI) calc. for $C_{13}H_{13}ClNaO_2S$ $[M+Na]^+$: 291.0217; found: 291.0209.

MP: 54 – 56 °C.

2,5-Dihydrothiophene S-81

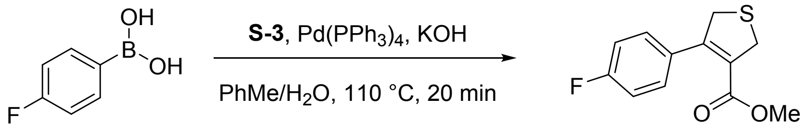

S-80 (87%) **S-81**

Following GP2 (20 minutes reaction time), triflate **S-3** (146 mg, 500 µmol, 1 equiv) and boronic acid **S-80** (86.5 mg, 600 µmol, 1.20 equiv) were converted into 2,5-dihydrothiophene **S-81**. Purification by flash-column chromatography on silica gel (10% to 20% diethyl ether in *n*-pentane) furnished 2,5-dihydrothiophene **S-81** (104 mg, 87%) as a yellow oil.

TLC (20% diethyl ether in petroleum ether): R_f = 0.47 (UV, $KMnO_4$).

^{1}H NMR ($CDCl_3$, 400 MHz): δ = 7.24 – 7.19 (m, 2H), 7.08 – 7.01 (m, 2H), 4.19 – 4.10 (m, 4H), 3.61 (s, 3H).

^{13}C NMR ($CDCl_3$, 101 MHz): δ = 164.8, 162.8 (d, *J* = 248 Hz), 151.1, 131.9 (d, *J* = 4 Hz), 129.4 (d, *J* = 8 Hz), 128.2, 115.3 (d, *J* = 22 Hz), 51.8, 45.0, 40.1.

^{19}F NMR (376 MHz, $CDCl_3$) δ = –112.8.

IR (Diamond-ATR, CH_2Cl_2): $\tilde{\upsilon}_{max}$ = 1722 (*m*), 1507 (*m*), 1434 (*w*), 1335 (*w*), 1315 (*w*), 1224 (*s*), 1204 (*s*), 1159 (*w*), 1044 (*w*), 835 (*w*).

HRMS (ESI) calc. for $C_{12}H_{11}FNaO_2S$ $[M+Na]^+$: 261.0356; found: 261.0341.

1,3-Diene S-16

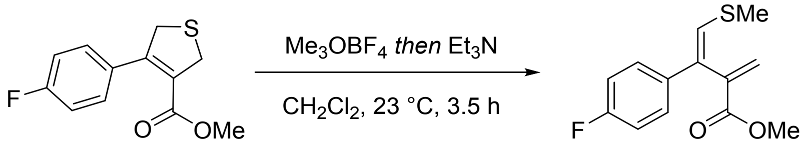

S-81 (96%) **S-16**

Following GP3 (three hours for *S*-methylation and 15 minutes for ring-opening), 2,5-dihydrothiophene **S-81** (71.5 mg, 300 µmol) was converted into 1,3-diene **S-16.** Purification by flash-column chromatography on silica gel (dichloromethane) furnished **S-16** (72.7 mg, 96%) as a yellow oil.

TLC (dichloromethane): R_f = 0.92 (UV, $KMnO_4$).

^{1}H NMR ($CDCl_3$, 400 MHz): δ = 7.25 – 7.20 (m, 2H), 7.01 – 6.93 (m, 2H), 6.62 (d, *J* = 1.4 Hz, 1H), 6.51 (s, 1H), 5.83 (d, *J* = 1.4 Hz, 1H), 3.69 (s, 3H), 2.37 (s, 3H).

^{13}C NMR ($CDCl_3$, 101 MHz): δ = 166.6, 162.2 (d, *J* = 247 Hz), 138.6, 136.0 (d, *J* = 3 Hz), 133.5, 131.2, 129.7 (d, *J* = 2 Hz), 127.3 (d, *J* = 8 Hz), 115.5 (d, *J* = 21 Hz), 52.4, 17.7.

^{19}F NMR ($CDCl_3$, 376 MHz) δ = –155.5.

IR (Diamond-ATR, CH_2Cl_2): $\tilde{\upsilon}_{max}$ = 1721 (*s*), 1508 (*m*), 1436 (*w*), 1281 (*w*), 1253 (*w*), 1227 (*m*), 1193 (*w*), 1157 (*w*), 1138 (*m*), 820 (*m*).

HRMS (ESI) calc. for $C_{13}H_{13}FNaO_2S$ $[M+Na]^+$: 275.0512; found: 275.0507.

1,3-Diene S-17

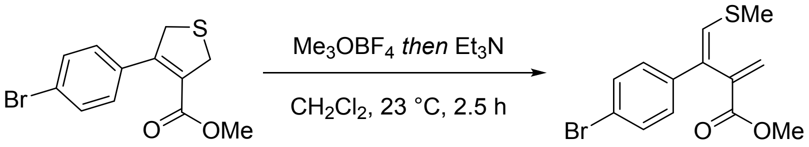

S-66 (93%) **S-17**

Following GP3 (1.5 hours for *S*-methylation), 2,5-dihydrothiophene **S-66** (89.8 mg, 300 µmol) was converted into 1,3-diene **S-17**. Purification by flash-column chromatography on silica gel (dichloromethane) furnished **S-17** (87.2 mg, 93%) as a colorless oil.

TLC (dichloromethane): R_f = 0.63 (UV, $KMnO_4$).

^{1}H NMR ($CDCl_3$, 400 MHz): δ = 7.42 – 7.37 (m, 2H), 7.15 – 7.11 (m, 2H), 6.64 (d, *J* = 1.4 Hz, 1H), 6.59 (s, 1H), 5.84 (d, *J* = 1.4 Hz, 1H), 3.69 (s, 3H), 2.38 (s, 3H)

^{13}C NMR ($CDCl_3$, 101 MHz): δ = 166.5, 138.7, 138.2, 133.2, 131.7, 131.6, 130.7, 127.2, 121.1, 52.4, 17.8.

IR (Diamond-ATR, CH_2Cl_2): $\tilde{\upsilon}_{max}$ = 1718 (*s*), 1486 (*w*), 1434 (*w*), 1250 (*m*), 1139 (*w*), 1154 (*m*), 1136 (*m*), 1073 (*w*), 1008 (*w*), 810 (*s*).

HRMS (ESI) calc. for $C_{13}H_{13}BrNaO_2S$ $[M+Na]^+$: 334.9712; found: 334.9707.

2,5-Dihydrothiophene S-83

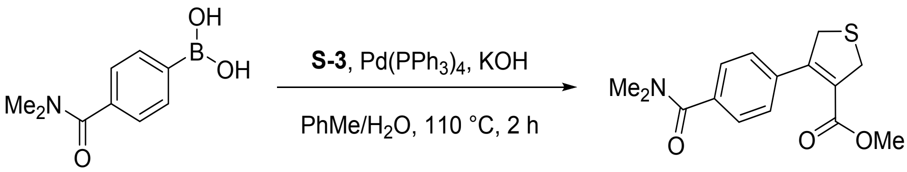

S-82 (81%) **S-83**

Following GP2 (two hours reaction time), triflate **S-3** (500 mg, 1.71 mmol, 1 equiv) and boronic acid **S-82** (495 mg, 2.57 mmol, 1.50 equiv) were converted into 2,5-dihydrothiophene **S-83**. Purification by flash-column chromatography on silica gel (10% to 20% diethyl ether in *n*-pentane) furnished **S-83** (403 mg, 81%) as a yellow oil.

TLC (70% ethyl acetate in cyclohexane): R_f = 0.30 (UV, $KMnO_4$).

^{1}H NMR ($CDCl_3$, 400 MHz): δ = 7.45 – 7.38 (m, 2H), 7.29 – 7.24 (m, 2H), 4.21 – 4.16 (m, 2H), 4.15 – 4.11 (m, 2H), 3.59 (s, 3H), 3.11 (s, 3H), 3.00 (s, 3H).

^{13}C NMR ($CDCl_3$, 101 MHz): δ = 171.2, 164.8, 151.0, 137.4, 136.4, 128.7, 127.6, 127.1, 51.8, 45.0, 40.3, 39.7, 35.5.

IR (Diamond-ATR, $CDCl_3$): $\tilde{\nu}_{max}$ = 2947 (*w*), 1722 (*m*), 1626 (*s*), 1393 (*m*), 1336 (*m*), 1230 (*m*), 1204 (*m*), 1081 (*m*), 846 (*w*), 756 (*w*).

HRMS (ESI) calc. for $C_{15}H_{17}NNaO_3S$ $[M+Na]^+$: 314.0821; found: 314.0805.

1,3-Diene S-18

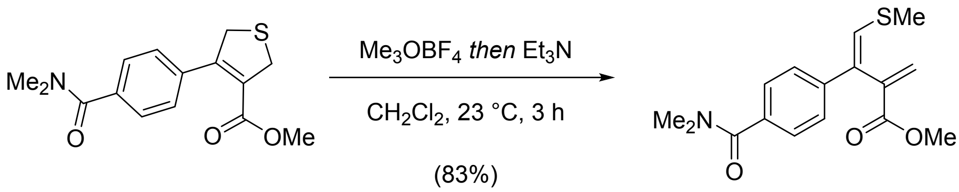

S-83 **S-18**

Following GP3 (2.5 hours for *S*-methylation and 15 minutes for ring-opening), 2,5-dihydrothiophene **S-83** (87.4 mg, 300 µmol) was converted into 1,3-diene **S-18**. Purification by flash-column chromatography on silica gel (10% to 40% methyl *tert*-butyl ether in dichloromethane) furnished **S-18** (76.4 mg, 83%) as a yellow oil.

TLC (50% methyl *tert*-butyl ether in dichloromethane): R_f = 0.50 (UV, $KMnO_4$, CAM).

^{1}H NMR ($CDCl_3$, 400 MHz): δ = 7.37 – 7.33 (m, 2H), 7.30 – 7.26 (m, 2H), 6.66 (s, 1H), 6.65 (d, *J* = 1.5 Hz, 1H), 5.84 (d, *J* = 1.4 Hz, 1H), 3.69 (s, 3H), 3.09 (s, 3H), 2.98 (s, 3H), 2.39 (s, 3H).

^{13}C NMR ($CDCl_3$, 101 MHz): δ = 171.5, 166.6, 140.9, 138.3, 134.7, 133.4, 131.6, 131.2, 127.6, 125.4, 52.4, 39.7, 35.6, 17.8.

IR (Diamond-ATR, CH_2Cl_2): $\tilde{\upsilon}_{max}$ = 1720 (*s*), 1628 (*s*), 1491 (*w*), 1438 (*w*), 1393 (*m*), 1259 (*m*), 1193 (*w*), 1137 (*w*), 1082 (*w*), 825 (*w*).

HRMS (ESI) calc. for $C_{16}H_{19}NNaO_3S$ $[M+Na]^+$: 328.0978; found: 328.0969.

2,5-Dihydrothiophene S-85

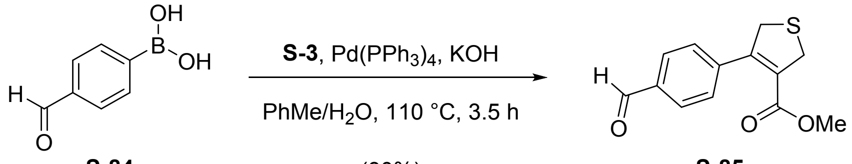

S-84 **S-85**

Following GP2 (3.5 hours reaction time), triflate **S-3** (146 mg, 500 µmol, 1 equiv) and boronic acid **S-84** (92.7 mg, 600 µmol, 1.20 equiv) were converted into 2,5-dihydrothiophene **S-85**. Purification by flash-column chromatography on silica gel (20% ethyl acetate in cyclohexane) furnished **S-85** (99 mg, 80%) as a yellow, amorphous solid.

TLC (40% ethyl acetate in cyclohexane): R_f = 0.64 (UV, $KMnO_4$).

^{1}H NMR ($CDCl_3$, 400 MHz): δ = 10.02 (s, 1H), 7.90 – 7.86 (m, 2H), 7.40 (d, *J* = 8.3 Hz, 2H), 4.22 – 4.12 (m, 4H), 3.59 (s, 3H).

^{13}C NMR ($CDCl_3$, 101 MHz): δ = 191.8, 164.4, 151.0, 142.5, 136.1, 129.7, 129.7, 128.2, 51.9, 44.9, 40.2.

IR (Diamond-ATR, $CDCl_3$): $\tilde{\nu}_{max}$ = 1720 (*s*), 1603 (*m*), 1434 (*w*), 1287 (*w*), 1232 (*m*), 1202 (*m*), 1170 (*w*), 1045 (*w*), 829 (*m*), 765 (*w*).

HRMS (ESI) calc. for $C_{13}H_{13}O_3S$ $[M+Na]^+$: 249.0580; found: 249.0577.

1,3-Diene S-19

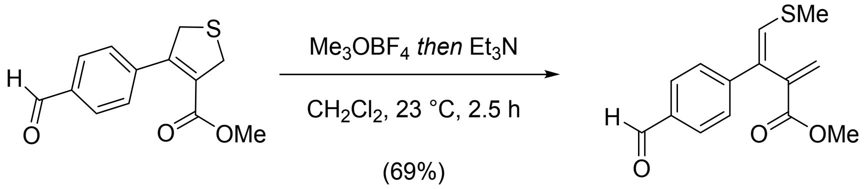

S-85 **S-19**

Following GP3 (1.5 hours for *S*-methylation), 2,5-dihydrothiophene **S-85** (75 mg, 0.30 mmol) was converted into 1,3-diene **S-19**. Purification by flash-column chromatography on silica gel (20% to 40% diethyl ether in *n*-pentane) furnished **S-19** (54 mg, 69%) as a yellow oil.

TLC (40% diethyl ether in petroleum ether): R_f = 0.19 (UV, $KMnO_4$).

^{1}H NMR ($CDCl_3$, 400 MHz): δ = 9.96 (s, 1H), 7.83 – 7.74 (m, 2H), 7.45 – 7.37 (m, 2H), 6.83 (s, 1H), 6.71 (d, *J* = 1.4 Hz, 1H), 5.89 (d, *J* = 1.4 Hz, 1H), 3.69 (s, 3H), 2.43 (s, 3H).

^{13}C NMR ($CDCl_3$, 101 MHz): δ = 191.7, 166.3, 145.3, 137.9, 134.8, 134.0, 132.7, 132.1, 130.2, 125.8, 52.4, 17.8.

IR (Diamond-ATR, CH_2Cl_2): $\tilde{\upsilon}_{max}$ = 1720 (*s*), 1697 (*s*), 1601 (*m*), 1572 (*w*), 1551 (*w*), 1257 (*w*), 1214 (*w*), 1171 (*w*), 1139 (*w*), 816 (*m*).

HRMS (ESI) calc. for $C_{14}H_{15}O_3S$ $[M+H]^+$: 263.0736; found: 263.0734.

1,3-Diene S-20

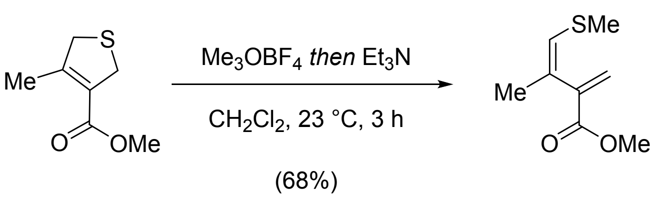

S-65 **S-20**

Following GP3 (2.5 hours for *S*-methylation and 30 minutes for ring-opening), 2,5-dihydrothiophene **S-67** (127 mg, 800 µmol) was converted into 1,3-diene **S-20**. Purification

by flash-column chromatography on silica gel (dichloromethane) furnished **S-20** (93.3 mg, 68%) as a pale-yellow oil.

TLC (dichloromethane): R_f = 0.69 (UV, $KMnO_4$).

^{1}H NMR ($CDCl_3$, 400 MHz): δ = 6.31 (d, *J* = 1.4 Hz, 1H), 5.93 (q, *J* = 1.4 Hz, 1H), 5.63 (d, *J* = 1.4 Hz, 1H), 3.78 (s, 3H), 2.23 (s, 3H), 1.95 (d, *J* = 1.4 Hz, 3H).

^{13}C NMR ($CDCl_3$, 101 MHz): δ = 166.7, 140.3, 131.9, 127.7, 126.4, 52.2, 23.7, 17.6.

IR (Diamond-ATR, CH_2Cl_2): $\tilde{\upsilon}_{max}$ = 1720 (*s*), 1684 (*w*), 1435 (*m*), 1315 (*w*), 1254 (*m*), 1199 (*w*), 1164 (*w*), 1111 (*w*), 1024 (*w*), 991 (*w*).

HRMS (ESI) calc. for $C_8H_{12}NaO_2S$ $[M+Na]^+$: 195.0450; found: 195.0449.

1,3-Diene S-21

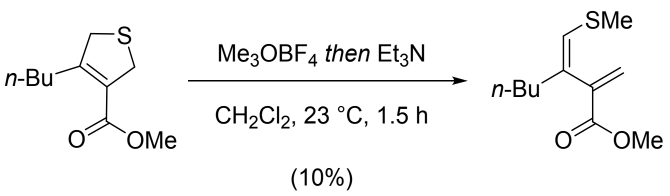

S-68 **S-21**

Following GP3 (one hour for *S*-methylation and 30 minutes for ring-opening), 2,5-dihydrothiophene **S-68** (253 mg, 1.26 mmol) was converted into 1,3-diene **S-21**. Purification by flash-column chromatography on silica gel (10% diethyl ether in petroleum ether) furnished **S-21** (27.0 mg, 10%) as a colorless oil.

TLC (20% diethyl ether in *n*-pentane): R_f = 0.60 (UV, $KMnO_4$).

^{1}H NMR ($CDCl_3$, 400 MHz): δ = 6.36 (d, *J* = 1.5 Hz, 1H), 5.90 (t, *J* = 1.2 Hz, 1H), 5.59 (d, *J* = 1.5 Hz, 1H), 3.75 (s, 3H), 2.27 – 2.22 (m, 2H), 2.22 (s, 3H), 1.30 (m, 4H), 0.86 (t, *J* = 7.1 Hz, 3H).

^{13}C NMR ($CDCl_3$, 101 MHz): δ = 166.7, 139.3, 136.5, 128.6, 125.9, 52.2, 36.9, 30.5, 22.2, 17.5, 14.0.

IR (Diamond-ATR, CH_2Cl_2): $\tilde{\upsilon}_{max}$ = 2954 (*w*), 2924 (*w*), 1722 (*s*), 1435 (*w*), 1317 (*m*), 1256 (*w*), 1195 (*w*), 1158 (*m*), 1119 (*w*), 815 (*w*).

HRMS (ESI) calc. for $C_{11}H_{18}NaO_2S$ $[M+Na]^+$: 237.0920; found: 237.0917.

1,3-Diene S-22

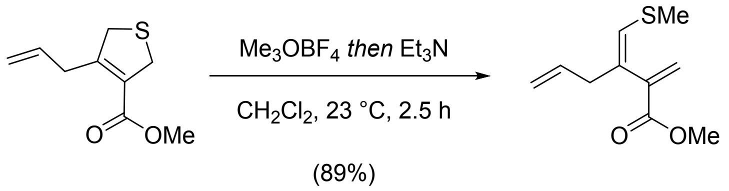

S-69 **S-22**

Following GP3 (30 minutes for ring-opening), 2,5-dihydrothiophene **S-69** (55 mg, 0.30 mmol) was converted into 1,3-diene **S-22**. Purification by flash-column chromatography on silica gel (dichloromethane) furnished **S-22** (53 mg, 89%) as a yellow oil.

TLC (dichloromethane): R_f = 0.73 (UV, $KMnO_4$).

^{1}H NMR ($CDCl_3$, 400 MHz): δ = 6.38 (d, *J* = 1.4 Hz, 1H), 5.97 (t, *J* = 1.3 Hz, 1H), 5.81 – 5.69 (m, 1H), 5.63 (d, *J* = 1.4 Hz, 1H), 5.07 – 5.01 (m, 2H), 3.77 (s, 3H), 3.00 (dq, *J* = 6.9, 1.3 Hz, 2H), 2.25 (s, 3H).

^{13}C NMR ($CDCl_3$, 101 MHz): δ = 166.6, 139.1, 135.5, 133.8, 129.0, 127.6, 117.0, 52.2, 41.4, 17.6.

IR (Diamond-ATR, CH_2Cl_2): $\tilde{\upsilon}_{max}$ = 1721 (*s*), 1435 (*w*), 1252 (*m*), 1195 (*w*), 1154 (*m*), 1112 (*w*), 994 (*w*), 952 (*w*), 917 (*w*), 813 (*w*).

HRMS (ESI) calc. for $C_{10}H_{14}NaO_2S$ $[M+Na]^+$: 221.0607; found: 221.0602.

1,3-Diene S-23

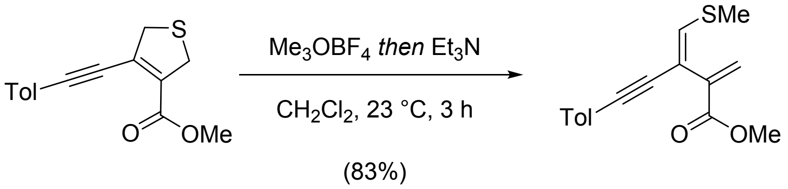

S-70 **S-23**

Following GP3 (2.5 hours for *S*-methylation and 15 minutes for ring-opening), 2,5-dihydrothiophene **S-70** (78 mg, 0.30 mmol) was converted into 1,3-diene **S-23**. Purification by flash-column chromatography on silica gel (20% diethyl ether in *n*-pentane) furnished **S-23** (68 mg, 83%) as a yellow oil.

TLC (20% diethyl ether in *n*-pentane): R_f = 0.40 (UV, $KMnO_4$).

^{1}H NMR ($CDCl_3$, 400 MHz): δ = 7.31 – 7.27 (m, 2H), 7.12 – 7.07 (m, 2H), 6.78 (s, 1H), 6.45 (d, *J* = 1.1 Hz, 1H), 5.89 (d, *J* = 1.0 Hz, 1H), 3.82 (s, 3H), 2.36 (s, 3H), 2.34 (s, 3H).

^{13}C NMR ($CDCl_3$, 101 MHz): δ = 166.1, 140.3, 138.3, 137.2, 131.4, 129.4, 129.2, 120.4, 115.9, 89.0, 88.1, 52.5, 21.6, 18.0.

IR (Diamond-ATR, CH_2Cl_2): $\tilde{\upsilon}_{max}$ = 1721 (*s*), 1435 (*w*), 1252 (*m*), 1195 (*w*), 1154 (*m*), 1112 (*w*), 994 (*w*), 952 (*w*), 917 (*w*), 813 (*w*).

HRMS (ESI) calc. for $C_{16}H_{17}O_2S$ $[M+H]^+$: 273.0944; found: 273.0940.

2,5-Dihydrothiophene S-86

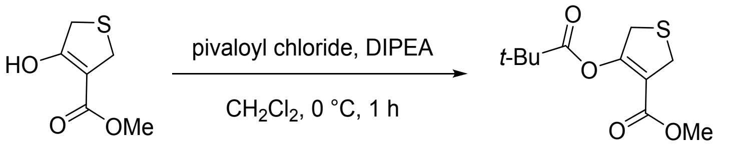

S-71 **S-86**

To a solution of 2,5-dihydrothiophene **S-71** (100 mg, 624 µmol, 1 equiv) in dichloromethane (2.5 mL) was added *N,N*-diisopropylethylamine (140 µL, 812 µmol, 1.30 equiv) at 23°C. The reaction mixture was cooled to 0 °C and pivaloyl chloride (75.0 mg, 624 µmol, 1.00 equiv) was added dropwise over 10 minutes. After one hour, the reaction mixture was allowed to warm to 23 °C. The solvent was removed under reduced pressure and the crude product was purified by flash-column chromatography on silica gel (5% diethyl ether in *n*-pentane) to furnish 2,5-dihydrothiophene **S-86** (137 mg, 90%) as a colorless solid.

TLC (5% diethyl ether in *n*-pentane): R_f = 0.50 (UV, $KMnO_4$).

^{1}H NMR (400 MHz, $CDCl_3$): δ = 3.90 (ddt, *J* = 7.0, 2.9, 1.6 Hz, 2H), 3.86 – 3.82 (m, 2H), 3.70 (s, 3H), 1.29 (s, 9H).

^{13}C NMR (101 MHz, $CDCl_3$): δ = 174.9, 162.8, 156.5, 117.8, 51.7, 39.2, 36.4, 33.5, 27.0.

IR (Diamond-ATR, $CDCl_3$): $\tilde{\upsilon}_{max}$ = 1757 (*m*), 1728 (*s*), 1343 (*s*), 1225 (*s*), 1168 (*s*), 1138 (*s*), 1068 (*vs*), 1045 (*m*), 1025 (*m*), 1005 (*m*).

HRMS (ESI) calc. for $C_{11}H_{16}NaO_4S$ $[M+Na]^+$: 267.0662; found: 267.0651.

MP: 54 – 59 °C.

1,3-Diene S-24

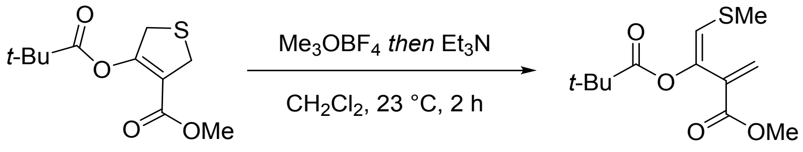

S-86 **S-24**

Following GP3 (one hours for *S*-methylation), 2,5-dihydrothiophene **S-86** (0.13 g, 0.52 mmol) was converted into 1,3-diene **S-24**. Purification by flash-column chromatography on silica gel (5% diethyl ether in *n*-pentane) furnished **S-24** (72 mg, 54%) as a colorless solid.

TLC (5% diethyl ether in *n*-pentane): R_f = 0.14 (UV, $KMnO_4$).

^{1}H NMR (400 MHz, $CDCl_3$): δ = 6.46 (s, 1H), 6.05 (s, 1H), 5.86 (s, 1H), 3.77 (s, 3H), 2.26 (s, 3H), 1.22 (s, 9H).

^{13}C NMR (101 MHz, $CDCl_3$): δ = 176.6, 165.2, 140.5, 134.2, 130.4, 120.7, 52.3, 38.9, 27.0, 18.0.

IR (Diamond-ATR, $CDCl_3$): $\tilde{\upsilon}_{max}$ = 1746 (*m*), 1726 (*s*), 1436 (*w*), 1251 (*s*), 1195 (*w*), 1150 (*m*), 1105 (*vs*), 1028 (*w*), 916 (*w*), 889 (*w*).

HRMS (ESI) calc. for $C_{12}H_{18}NaO_4S$ $[M+Na]^+$: 281.0818; found: 281.0812.

MP: 48 – 49 °C.

2,5-Dihydrothiophene S-90

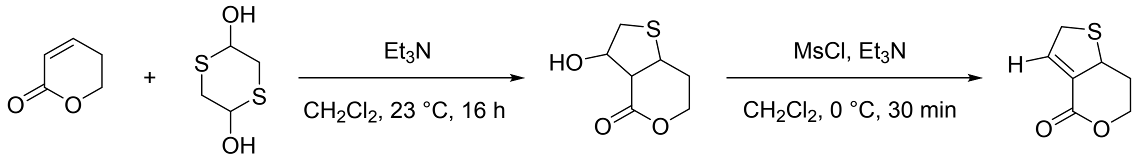

S-87 **S-88** **S-89** **S-90**

To a solution of lactone **S-87** (439 µL, 5.10 mmol, 2.00 equiv) and 1,4-dithiane-2,5-diol (**S-88**, 388 mg, 2.55 mmol, 1 equiv) in dichloromethane (25 mL) under argon was added triethylamine (710 µL, 5.10 mmol, 2.00 equiv) at 23 °C. After 16 hours, the solvent was removed under reduced pressure and the crude product was purified by flash-column chromatography on silica gel (50% ethyl acetate in *n*-pentane) to furnish tetrahydrothiophene **S-89** as a colorless oil (511 mg), which was used in the next step without any further purification.

TLC (50% ethyl acetate in *n*-pentane): R_f = 0.33 (UV, CAM).

To a solution of tetrahydrothiophene **S-89** (511 mg, 2.93 mmol, 1 equiv) and triethylamine (1.23 mL, 8.80 mmol, 3.00 equiv) in dichloromethane (15 mL) under argon was added methanesulfonyl chloride (341 µL, 4.40 mmol, 1.50 equiv) at 0 °C. After 30 minutes, saturated aqueous bicarbonate solution (15 mL) was added. The aqueous layer was extracted with dichloromethane (3 × 10 mL), the combined organic layers were dried over sodium sulfate, the dried solution was filtered and the filtrate was concentrated under reduced pressure. The crude product was purified by flash-column chromatography on silica gel (50% ethyl acetate in *n*-pentane) to furnish 2,5-dihydrothiophene **S-90** (354 mg, 77%, over two steps) as a colorless solid.

TLC (50% ethyl acetate in *n*-pentane): R_f = 0.75 (UV, CAM).

^{1}H NMR (400 MHz, $CDCl_3$): δ = 7.03 – 6.96 (m, 1H), 4.56 – 4.43 (m, 2H), 4.35 (ddd, *J* = 12.7, 11.8, 3.0 Hz, 1H), 3.91 (ddd, *J* = 17.0, 5.0, 2.3 Hz, 1H), 3.77 (ddd, *J* = 17.0, 5.8, 3.5 Hz, 1H), 2.33 – 2.23 (m, 1H), 2.11 (dtd, *J* = 13.6, 12.4, 4.9 Hz, 1H).

^{13}C NMR (101 MHz, $CDCl_3$): δ = 162.1, 142.3, 134.7, 69.3, 48.6, 37.3, 33.4.

IR (Diamond-ATR, $CDCl_3$): $\tilde{\upsilon}_{max}$ = 1709 (*vs*), 1643 (*m*), 1396 (*m*), 1296 (*m*), 1251 (*vs*), 1218 (*m*), 1202 (*m*), 1113 (*m*), 1086 (*m*), 727 (*m*).

HRMS (ESI) calc. for $C_7H_9O_2S$ $[M+H]^+$: 157.0318; found: 157.0317.

MP: 88 – 92 °C.

1,3-Diene S-25

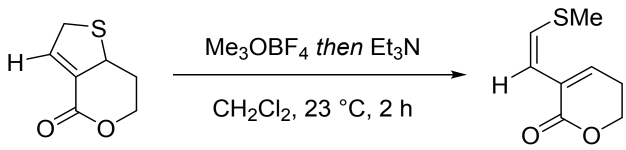

S-90 (79%) **S-25**

Following GP3 (one hours for *S*-methylation), 2,5-dihydrothiophene **S-90** (175 mg, 1.12 mmol) was converted into 1,3-diene **S-25**. Purification by flash-column chromatography on silica gel (40% ethyl acetate in *n*-pentane) furnished **S-25** (150 mg, 79%) as a colorless oil.

TLC (40% ethyl acetate in *n*-pentane): R_f = 0.61 (UV, $KMnO_4$).

^{1}H NMR (400 MHz, $CDCl_3$): δ = 6.98 (t, *J* = 4.7 Hz, 1H), 6.46 (d, *J* = 11.0 Hz, 1H), 6.33 (d, *J* = 10.9 Hz, 1H), 4.41 (t, *J* = 6.2 Hz, 2H), 2.61 (q, *J* = 5.9 Hz, 2H), 2.39 (s, 3H).

^{13}C NMR (101 MHz, $CDCl_3$): δ = 164.2, 140.4, 131.9, 128.4, 118.4, 66.2, 24.8, 18.9.

IR (Diamond-ATR, $CDCl_3$): $\tilde{\upsilon}_{max}$ = 1714 (*vs*), 1399 (*w*), 1334 (*w*), 1276 (*w*), 1187 (*m*), 1112 (*s*), 992 (*w*), 819 (*m*), 732 (*w*), 694 (*w*).

HRMS (ESI) calc. for $C_8H_{11}O_2S$ $[M+H]^+$: 171.0474; found: 171.0474.

Triflate S-4

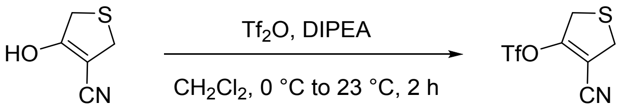

S-91 (81%) **S-4**

To a solution of 2,5-dihydrothiophene **S-91** (3.00 g, 23.6 mmol, 1 equiv) and *N*,*N*-diisopropylethylamine (5.40 mL, 30.7 mmol, 1.30 equiv) in dichloromethane (31 mL) under argon was added dropwise trifluoromethanesulfonic anhydride (4.78 mL, 28.3 mmol, 1.20 equiv) at 0 °C. Upon addition, the reaction mixture was allowed to warm to 23 °C. After two hours, the solvent was removed under reduced pressure and the crude product was purified by flash-column chromatography on silica gel (10% diethyl ether in *n*-pentane) to yield triflate **S-4** (4.95 g, 81%) as a pale-yellow oil.

TLC (10% diethyl ether in petroleum ether): R_f = 0.36 (UV, $KMnO_4$).

^{1}H NMR ($CDCl_3$, 400 MHz): δ = 4.05 (dd, *J* = 5.9, 4.4 Hz, 2H), 3.85 (dd, *J* = 5.9, 4.4 MHz, 2H).

^{13}C NMR ($CDCl_3$, 101 MHz): δ = 157.5, 118.4 (q, *J* = 321.1 Hz), 110.7, 104.4, 34.6, 32.1.

^{19}F NMR ($CDCl_3$, 376 MHz): δ = −72.7.

IR (Diamond-ATR, $CDCl_3$): $\tilde{\upsilon}_{max}$ = 2234 (*w*), 1667 (*w*), 1429 (*m*), 1216 (*s*), 1133 (*s*), 1021 (*m*), 836 (*m*), 751 (*w*), 594 (m), 501 (*m*).

HRMS (ESI) calc. for $C_6H_4F_3NNaO_3S$ $[M+Na]^+$: 281.9477; found: 281.9474.

2,5-Dihydrothiophene S-92

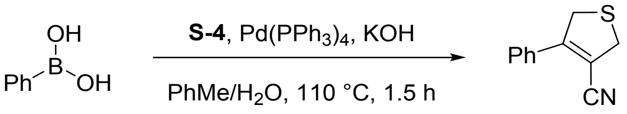

S-72 (85%) **S-92**

Following GP2 (1.5 hours reaction time), triflate **S-4** (698 mg, 2.69 mmol, 1 equiv) and phenylboronic acid (**S-69**, 394 mg, 3.23 mmol, 1.20 equiv) were converted into 2,5-dihydrothiophene **S-92**. Purification by flash-column chromatography on silica gel (10% to 20% diethyl ether in *n*-pentane) furnished **S-92** (427 mg, 85%) as a colorless oil. The obtained analytical data were in full agreement with those reported in the literature.[8]

TLC (20% diethyl ether in *n*-pentane): R_f = 0.45 (UV, $KMnO_4$).

2,5-Dihydrothiophene S-93

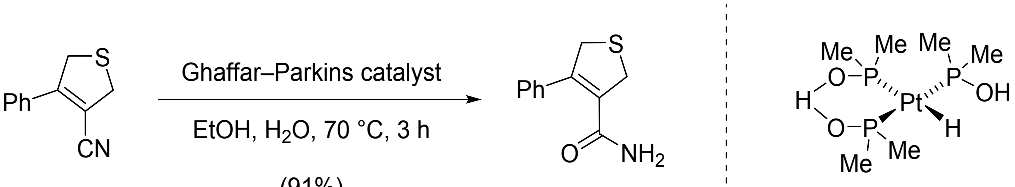

S-92 (91%) **S-93** Ghaffar–Parkins catalyst

To a solution of nitrile **S-92** (200 mg, 1.07 mmol, 1 equiv) in ethanol (8 mL) and water (2 mL) was added Ghaffar-Parkins catalyst (23 mg, 53 µmol, 5.0 mol%) at 23°C. Upon complete addition, the reaction mixture was heated to 70 °C. After three hours, the solution was allowed to cool down to 23 °C and ethyl acetate (5 mL) was added. The reaction mixture was dried over sodium sulfate, filtered through a plug of Celite® and the dried solution was concentrated under reduced pressure. The crude product was purified by flash-column chromatography on silica gel (50% ethyl acetate in *n*-pentane) to furnish primary amide **S-93** (200 mg, 91%) as a colorless solid.

TLC (20% ethyl acetate in *n*-pentane): R_f = 0.25 (UV, $KMnO_4$).

^{1}H NMR (400 MHz, $CDCl_3$) δ = 7.47 – 7.34 (m, 3H), 7.34 – 7.29 (m, 2H), 5.22 (s, 1H), 5.12 (s, 1H), 4.28 – 4.14 (m, 4H).

^{13}C NMR (101 MHz, $CDCl_3$): δ = 167.0, 145.0, 135.2, 132.2, 129.3, 129.1, 127.6, 45.3, 41.1.

IR (Diamond-ATR, $CDCl_3$): $\tilde{\upsilon}_{max}$ = 2905 (*w*), 2854 (*w*), 1619 (*vs*), 1493 (*m*), 1437 (*m*), 1308 (*m*), 1271 (*w*), 1242 (*m*), 1112 (*w*), 763 (*m*).

HRMS (ESI) calc. for $C_{11}H_{12}NOS$ $[M+H]^+$: 206.0634; found: 206.0635.

MP: 156 – 158 °C.

1,3-Dienes S-26 and S-94

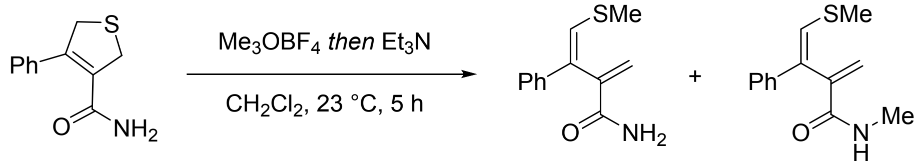

S-93 **S-26** (75%) **S-94** (15%)

Following GP3 (four hours for *S*-methylation), 2,5-dihydrothiophene **S-90** (174 mg, 848 μmol) was converted into 1,3-dienes **S-26** and **S-94**. Purification by flash-column chromatography on silica gel (50% ethyl acetate in *n*-pentane) furnished **S-26** (139 mg, 75%) as a yellow solid and **S-94** (30.0 mg, 15%) as a colorless oil.

*Major product **S-26**:*

TLC (50% ethyl acetate in *n*-pentane): R_f = 0.40 (UV, CAM).

^{1}H NMR (400 MHz, $CDCl_3$): δ = 7.30 – 7.13 (m, 5H), 6.69 (s, 1H), 6.58 (d, *J* = 1.7 Hz, 1H), 5.74 (s, 1H), 5.67 (s, 1H), 5.60 (d, *J* = 1.7 Hz, 1H), 2.34 (s, 3H).

^{13}C NMR (101 MHz, $CDCl_3$): δ = 167.0, 140.1, 138.4, 133.4, 131.2, 129.1, 128.8, 127.5, 125.3, 17.6.

IR (Diamond-ATR, $CDCl_3$): $\tilde{\upsilon}_{max}$ = 3178 (*w*), 1674 (*vs*), 1614 (*m*), 1595 (*m*), 1492 (*w*), 1442 (*w*), 1370 (*w*), 822 (*m*), 762 (*w*), 695 (*m*).

HRMS (ESI) calc. for $C_{12}H_{13}NNaOS$ $[M+Na]^+$: 242.0610; found: 242.0608.

MP: 97 – 104 °C.

*Minor product **S-94***:

TLC (40% ethyl acetate in *n*-pentane): R_f = 0.56 (UV, CAM).

^{1}H NMR (400 MHz, $CDCl_3$): δ = 7.39 – 7.14 (m, 5H), 6.70 (s, 1H), 6.24 (d, *J* = 1.5 Hz, 1H), 5.55 (d, *J* = 1.5 Hz, 1H), 3.79 (s, 3H), 2.36 (s, 3H).

^{13}C NMR (101 MHz, $CDCl_3$): δ = 166.2, 138.9, 133.9, 131.2, 128.7, 128.7, 127.3, 125.3, 124.6, 53.5, 17.6.

IR (Diamond-ATR, $CDCl_3$): $\tilde{\upsilon}_{max}$ = 1639 (*m*), 1600 (*m*), 1443 (*m*), 1366 (*m*), 1324 (*m*), 1190 (*m*), 1086 (*vs*), 840 (*m*), 757 (*vs*), 696 (*m*).

HRMS (ESI) calc. for $C_{13}H_{16}NOS$ $[M+H]^+$: 234.0947; found: 234.0945.

2,5-Dihydrothiophene S-95

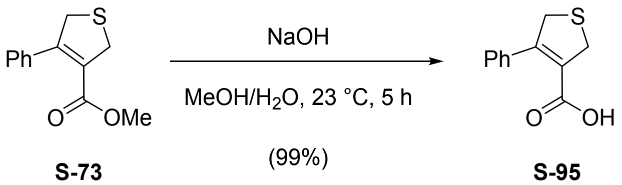

S-73 **S-95**

To a solution of 2,5-dihydrothiophene **S-73** (394 mg, 1.79 mmol, 1 equiv) in methanol (3.00 mL) was added aqueous sodium hydroxide solution (2.80 M, 143 mg, 1.28 mL, 2.00 equiv) at 23 °C. After 15 hours, aqueous hydrochloric acid solution (1 M, 20 mL) and dichloromethane (20 mL) were added. The layers were separated and the aqueous phase was extracted with dichloromethane (2 × 20 mL), the combined organic layers were dried over sodium sulfate, the dried solution was filtered and the filtrate was concentrated under reduced pressure. The crude product was purified by flash-column chromatography (20% ethyl acetate and 0.1% formic acid in cyclohexane) to yield 2,5-dihydrothiophene **S-95** (364 mg, 99%) as a colorless solid.

TLC (20% ethyl acetate in cyclohexane): R_f = 0.10 (UV, $KMnO_4$).

^{1}H NMR ($CDCl_3$, 400 MHz): δ = 7.35 (dd, *J* = 5.1, 1.9 Hz, 3H), 7.25 – 7.22 (m, 2H), 4.16 (s, 4H).

^{13}C NMR ($CDCl_3$, 101 MHz): δ = 168.4, 154.7, 135.7, 128.8, 128.4, 127.5, 127.2, 45.7, 40.2.

IR (Diamond-ATR, CH_2Cl_2): $\tilde{\upsilon}_{max}$ = 2898 (*w*), 1718 (*s*), 1683 (*m*), 1662 (*s*), 1637 (*s*), 1424 (*w*), 1368 (*m*), 1311 (*m*), 1255 (*m*), 950 (*w*).

HRMS (ESI) calc. for $C_{11}H_9O_2S$ $[M–H]^-$: 205.0329; found: 205.0322.

MP: 128 – 130 °C.

2,5-Dihydrothiophene S-96

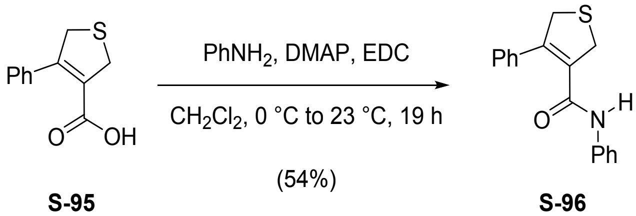

S-95 **S-96**

To a solution of acid **S-95** (155 mg, 751 µmol, 1 equiv) in dichloromethane (7.50 mL) were added DMAP (138 mg, 1.13 mmol, 1.50 equiv) and EDC (158 mg, 827 µmol, 1.10 equiv) at 0 °C. After 30 minutes aniline (82.3 µL, 902 µmol, 1.20 equiv) was added at 0 °C. Overnight the reaction mixture was allowed to warm to 23 °C. After 19 hours, aqueous hydrochloric acid solution (1 M, 20 mL) and dichloromethane (20 mL) were added. The layers were separated and the aqueous phase was extracted with dichloromethane (2 × 20 mL), the combined organic layers were dried over sodium sulfate, the dried solution was filtered and the filtrate was concentrated under reduced pressure. The crude product was purified by flash-column chromatography (10% to 20% ethyl acetate in cyclohexane) to yield 2,5-dihydrothiophene **S-96** (114 mg, 54%) as a yellow solid.

TLC (20% ethyl acetate in cyclohexane): R_f = 0.45 (UV, $KMnO_4$).

^{1}H NMR ($CDCl_3$, 400 MHz): δ = 7.51 – 7.43 (m, 3H), 7.37 (dd, *J* = 7.2, 2.4 Hz, 2H), 7.20 (dd, *J* = 8.7, 7.1 Hz, 2H), 7.03 (t, *J* = 7.6 Hz, 3H), 6.87 (s, 1H), 4.28 (m, 4H).

^{13}C NMR ($CDCl_3$, 101 MHz): δ = 163.1, 144.2, 137.4, 135.1, 133.9, 129.6, 129.5, 129.0, 128.0, 124.6, 119.7, 45.0, 41.2.

IR (Diamond-ATR, CH_2Cl_2): $\tilde{\upsilon}_{max}$ = 3726 (*w*), 1656 (*m*), 1597 (*m*), 1531 (*m*), 1497 (*w*), 1441 (*s*), 1332 (*w*), 1248 (*w*), 755 (*m*), 693 (*w*).

HRMS (ESI) calc. for $C_{17}H_{15}KNOS$ $[M+K]^+$: 320.0506; found: 320.0510.

MP: 146 – 147 °C.

1,3-Diene S-27

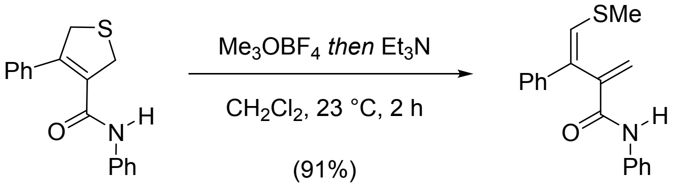

S-96 **S-27**

Following GP3 (one hour for *S*-methylation), 2,5-dihydrothiophene **S-96** (55 mg, 0.19 mmol) was converted into 1,3-diene **S-27**. Purification by flash-column chromatography on silica gel (30% ethyl acetate in cyclohexane) furnished **S-27** (52 mg, 91%) as a pale-yellow solid.

TLC (40% ethyl acetate in cyclohexane): R_f = 0.52 (UV, $KMnO_4$).

^{1}H NMR ($CDCl_3$, 400 MHz): δ = 7.45 (s, 1H), 7.33 (m, 4H), 7.26 (m, 2H), 7.19 (m, 3H), 7.00 (m, 1H), 6.80 (s, 1H), 6.69 (d, *J* = 1.7 Hz, 1H), 5.66 (d, *J* = 1.6 Hz, 1H), 2.36 (s, 3H).

^{13}C NMR ($CDCl_3$, 101 MHz): δ = 163.0, 141.2, 138.6, 137.9, 133.0, 132.3, 129.3, 129.1, 129.0, 127.9, 125.5, 124.5, 120.2, 17.8.

IR (Diamond-ATR, $CDCl_3$): $\tilde{\upsilon}_{max}$ = 1672 (*m*), 1598 (*m*), 1523 (*s*), 1498 (*m*), 1440 (*s*), 1315 (*w*), 1245 (*w*), 826 (*w*), 756 (*m*), 692 (*m*).

HRMS (ESI) calc. for $C_{18}H_{18}NOS$ $[M+H]^+$: 296.1104; found: 296.1105.

MP: 101 – 102 °C.

1,3-Diene S-32

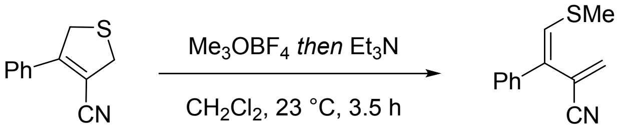

S-92 (85%) **S-32**

Following GP3 (three hours for *S*-methylation and 30 minutes for ring-opening), 2,5-dihydrothiophene **S-92** (100 mg, 534 µmol) was converted into 1,3-diene **S-32**. Purification

by flash-column chromatography on silica gel (10% diethyl ether in *n*-pentane) furnished **S-32** (90.9 mg, 85%) as a pale-yellow oil which solidified upon standing in the freezer.

TLC (20% diethyl ether in petroleum ether): R_f = 0.11 (UV, $KMnO_4$).

^{1}H NMR ($CDCl_3$, 400 MHz): δ = 7.38 – 7.27 (m, 5H), 6.60 (s, 1H), 6.37 (s, 1H), 6.02 (s, 1H), 2.45 (s, 3H).

^{13}C NMR ($CDCl_3$, 101 MHz): δ = 138.5, 135.8, 133.2, 131.2, 128.8, 128.1, 127.0, 120.6, 117.5, 18.5.

IR (Diamond-ATR, CH_2Cl_2): $\tilde{\upsilon}_{max}$ = 2921 (*w*), 2216 (*w*), 1597 (*w*), 1575 (*w*), 1491 (*w*), 1442 (*m*), 1047 (*w*), 826 (*w*), 756 (*m*), 701 (*s*).

HRMS (ESI) calc. for $C_{12}H_{11}NNaS$ $[M+Na]^+$: 224.0504; found: 224.0502.

MP: 73 – 74 °C.

2,5-Dihydrothiophene S-98

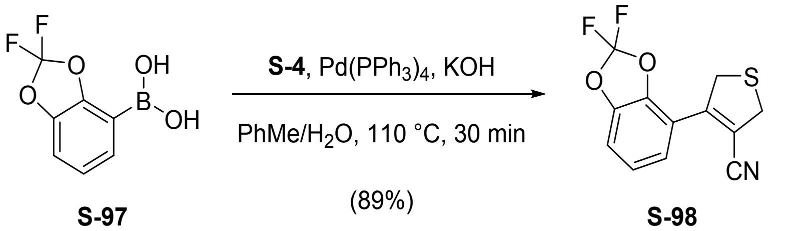

S-97 **S-98**

Following GP2 (30 minutes reaction time), triflate **S-4** (600 mg, 2.31 mmol, 1 equiv) and boronic acid **S-97** (561 mg, 2.78 mmol, 1.20 equiv) were converted into 2,5-dihydrothiophene **S-98**. Purification by flash-column chromatography on silica gel (5% to 10% ethyl acetate in petroleum ether) furnished **S-98** (553 mg, 89%) as a colorless solid.

TLC (10% diethyl ether in *n*-pentane): R_f = 0.38 (UV, $KMnO_4$).

^{1}H NMR (400 MHz, $CDCl_3$): δ = 7.39 (dd, *J* = 7.3, 2.0 Hz, 1H), 7.19 – 7.11 (m, 2H), 4.28 (dd, *J* = 5.5, 4.3 Hz, 2H), 4.07 (dd, *J* = 5.5, 4.3 Hz, 2H).

^{13}C NMR (101 MHz, $CDCl_3$): δ = 150.6, 144.1, 140.8, 131.2 (t, *J* = 257.3 Hz), 124.2, 122.9, 115.9, 114.9, 111.3, 110.4, 40.7, 39.5.

^{19}F NMR (376 MHz, $CDCl_3$) δ = –49.5.

IR (Diamond-ATR, $CDCl_3$): $\tilde{\upsilon}_{max}$ = 1451 (*s*), 1242 (*vs*), 1156 (*s*), 1113 (*m*), 1061 (*w*), 1039 (*w*), 904 (*w*), 843 (*m*), 779 (*m*), 772 (*m*).

HRMS (ESI) calc. for $C_{12}H_7F_2NNaO_2S$ $[M+Na]^+$: 290.0058; found: 290.0057.

MP: 97 – 99 °C.

1,3-Diene S-34

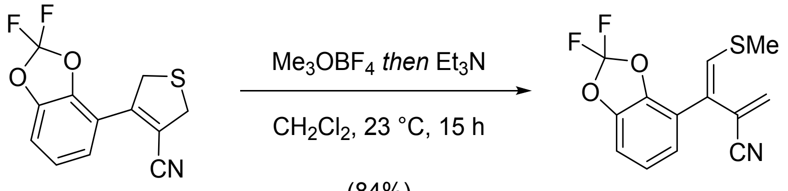

S-98 **S-34**

Following GP3 (14 hours for *S*-methylation), 2,5-dihydrothiophene **S-93** (150 mg, 561 μmol) was converted into 1,3-diene **S-34**. Purification by flash-column chromatography on silica gel (5% ethyl acetate in *n*-pentane) furnished **S-34** (133 mg, 84%) as a colorless solid.

TLC (10% ethyl acetate in *n*-pentane): R_f = 0.33 (UV, $KMnO_4$).

^{1}H NMR (400 MHz, CDCl3): δ = 7.35 (t, *J* = 8.0 Hz, 1H), 7.31 – 7.24 (m, 2H), 7.21 (dd, *J* = 8.0, 1.2 Hz, 1H), 6.69 (s, 1H), 6.34 (s, 1H), 2.76 (s, 3H).

^{13}C NMR (101 MHz, $CDCl_3$): δ = 144.1, 140.6, 138.7, 136.4, 131.5 (t, *J* = 256 Hz), 124.0, 123.5, 122.7, 121.3, 119.2, 117.0, 108.8, 18.5.

^{19}F NMR (376 MHz, $CDCl_3$): δ = –49.5.

IR (Diamond-ATR, $CDCl_3$): $\tilde{\upsilon}_{max}$ = 1556 (*m*), 1450 (*s*), 1225 (*vs*), 1145 (*s*), 1120 (*s*), 1025 (*w*), 963 (*w*), 772 (*s*), 718 (*w*).

HRMS (ESI) calc. for $C_{13}H_9F_2NNaO_2S$ $[M+Na]^+$: 304.0214; found: 305.0213.

MP: 91 – 98 °C.

1,3-Diene S-44

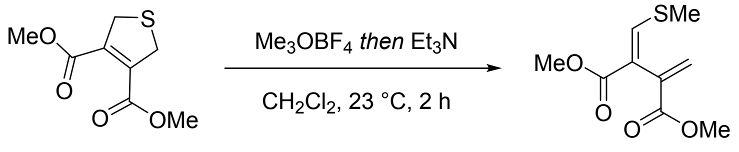

S-99 **S-44**

Following GP3 (one hour for *S*-methylation), 2,5-dihydrothiophene **S-99** (150 mg, 742 μmol) was converted into 1,3-diene **S-44**. Purification by flash-column chromatography on silica gel (20% diethyl ether in *n*-pentane to 100% ethyl acetate) furnished **S-44** (138 mg, 86%) as a colorless oil.

TLC (20% diethyl ether in *n*-pentane): R_f = 0.28 (UV, $KMnO_4$).

^{1}H NMR (400 MHz, $CDCl_3$): δ = 7.74 (s, 1H), 6.55 (d, *J* = 1.1 Hz, 1H), 5.78 (d, *J* = 1.1 Hz, 1H), 3.76 (s, 3H), 3.72 (s, 3H), 2.44 (s, 3H).

^{13}C NMR (101 MHz, $CDCl_3$): δ = 166.2, 164.8, 148.6, 135.1, 130.2, 123.7, 52.3, 52.0, 17.8.

IR (Diamond-ATR, $CDCl_3$): $\tilde{\upsilon}_{max}$ = 1703 (*s*), 1573 (*m*), 1434 (*w*), 1233 (v*s*), 1194 (*m*), 1144 (*w*), 1064 (*w*), 989 (*w*), 770 (*w*), 755 (*w*).

HRMS (ESI) calc. for $C_9H_{12}NaO_4S$ $[M+Na]^+$: 239.0349; found: 239.0349.

Note: For the synthesis of **S-99** through the [3+2]-cycloaddition of thiocarbonyl ylides to alkynes, see *J. Am. Chem. Soc.* **2019***, 141*, 13352−13357.[9]

1,3-Diene S-46

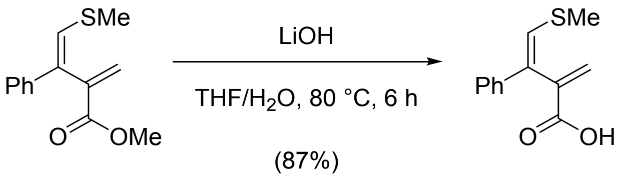

5a **S-46**

To a solution of 1,3-diene **5a** (300 mg, 1.28 mmol, 1 equiv) in tetrahydrofuran (6.4 mL) and water (6.4 mL) was added lithium hydroxide (153 mg, 6.40 mmol, 5.00 equiv) in one portion at 23 °C. The reaction mixture was heated to 80 °C with a pre-heated oil bath. After six hours, the reaction mixture was allowed to cool to 23 °C and aqueous hydrochloric acid solution (3 M, 15 mL) and ethyl acetate (15 mL) were added. The layers were separated and the aqueous phase was extracted with ethyl acetate (2 × 15 mL). The combined organic layers were dried over sodium sulfate, the dried solution was filtered and the filtrate was concentrated. The crude product was purified by flash-column chromatography on silica gel (0.1% formic acid and 20% ethyl acetate in cyclohexane) to yield acid **S-46** (245.5 mg, 87%) as a colorless solid.

TLC (0.1% formic acid and 20% ethyl acetate in cyclohexane): R_f = 0.23 (UV, $KMnO_4$, BCG).

^{1}H NMR ($CDCl_3$, 400 MHz): δ = 7.31 – 7.27 (m, 4H), 7.24 – 7.19 (m, 1H), 6.74 (d, *J* = 1.4 Hz, 1H), 6.61 (s, 1H), 5.94 (d, *J* = 1.4 Hz, 1H), 2.38 (s, 3H).

^{13}C NMR ($CDCl_3$, 101 MHz): δ = 170.9, 139.4, 138.1, 133.8, 133.5, 130.2, 128.7, 127.3, 125.5, 17.7

IR (Diamond-ATR, CH_2Cl_2): $\tilde{\upsilon}_{max}$ = 3026 (*w*), 2924 (*w*), 2857 (*w*), 1696 (*s*), 1617 (*w*), 1493 (*w*), 1443 (*w)*, 1318 (*m*), 1268 (*m*), 757 (*m*).

HRMS (ESI) calc. for $C_{12}H_{11}O_2S$ $[M-H]^-$: 219.0485; found: 219.0479.

MP: 109 – 111 °C.

2,5-Dihydrothiophene S-101

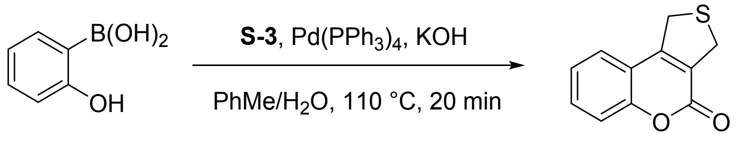

S-100 (77%) **S-101**

Following GP2 (20 minutes reaction time), triflate **S-3** (146 mg, 500 µmol, 1 equiv) and boronic acid **S-100** (82.8 mg, 600 µmol, 1.20 equiv) were converted into 2,5-dihydrothiophene **S-101**.

Purification by flash-column chromatography on silica gel (20% diethyl ether in *n*-pentane) furnished 2,5-dihydrothiophene **S-101** (78.7 mg, 77%) as a colorless solid.

TLC (20% diethyl ether in petroleum ether): R_f = 0.23 (UV, $KMnO_4$).

^{1}H NMR ($CDCl_3$, 400 MHz): δ = 7.56 (ddd, *J* = 8.6, 7.2, 1.7 Hz, 1H), 7.42 (td, *J* = 8.3, 1.4 Hz, 2H), 7.33 (td, *J* = 7.6, 1.2 Hz, 1H), 4.50 – 4.42 (m, 2H), 4.29 – 4.21 (m, 2H).

^{13}C NMR ($CDCl_3$, 101 MHz): δ = 159.2, 153.8, 152.2, 131.9, 126.5, 125.0, 124.8, 118.1, 117.1, 38.2, 37.2.

IR (Diamond-ATR, CH_2Cl_2): $\tilde{\upsilon}_{max}$ = 1719 (*s*), 1604 (*m*), 1454 (*w*), 1375 (*w*), 1176 (*w*), 1068 (*w*), 1038 (*m*), 1025 (*m*), 775 (*m*), 749 (*m*).

HRMS (ESI) calc. for $C_{11}H_8NaO_2S$ $[M+Na]^+$: 227.0137; found: 227.0125.

MP: 148 – 150 °C.

2,5-Dihydrothiophene S-102

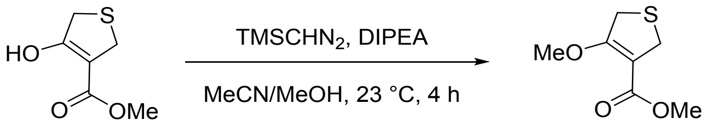

S-71 (67%) **S-102**

To a solution of 2,5-dihydrothiophene **S-71** (200 mg, 1.25 mmol, 1 equiv) in acetonitrile (1.8 mL) and methanol (0.2 mL) under argon was added *N,N*-diisopropylethylamine (0.22 mL, 1.3 mmol, 1.0 equiv) and a solution of (trimethylsilyl)diazomethane (2.0 M in diethyl ether, 0.62 mL, 1.3 mmol, 1.0 equiv) at 23 °C. After four hours, the solvent was removed under reduced pressure and the crude product was purified by flash-column chromatography on silica gel (50% ethyl acetate in *n*-pentane) to furnish 2,5-dihydrothiophene **S-102** (145 mg, 67%) as a colorless solid.

TLC (50% ethyl acetate in *n*-pentane): R_f = 0.29 (UV, $KMnO_4$).

^{1}H NMR (400 MHz, $CDCl_3$): δ = 3.97 (dd, *J* = 4.5, 3.4 Hz, 2H), 3.87 (s, 3H), 3.85 (dd, *J* = 4.5, 3.3 Hz, 2H), 3.72 (s, 3H).

^{13}C NMR (101 MHz, $CDCl_3$): δ = 165.5, 164.3, 104.3, 58.1, 51.2, 34.6, 33.3.

IR (Diamond-ATR, $CDCl_3$): $\tilde{\upsilon}_{max}$ = 1671 (*s*), 1623 (s), 1443 (*m*), 1374 (*m*), 1273 (*m*), 1212 (v*s*), 1192 (*s*), 1170 (*s*), 1146 (*m*), 1054 (*vs*).

HRMS (ESI) calc. for $C_7H_{10}NaO_3S$ $[M+Na]^+$: 197.0243; found: 197.0239.

1,3-Diene S-103

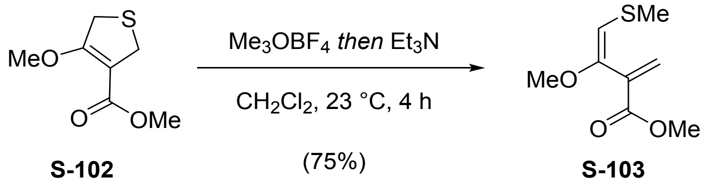

Following GP3 (three hours for *S*-methylation), 2,5-dihydrothiophene **S-102** (145 mg, 832 µmol) was converted into 1,3-diene **S-103**. Purification by flash-column chromatography on silica gel (50% diethyl ether in *n*-pentane) furnished **S-103** (118 mg, 75%) as a colorless solid.

TLC (50% ethyl acetate in *n*-pentane): R_f = 0.55 (UV, $KMnO_4$).

^{1}H NMR (400 MHz, $CDCl_3$): δ = 6.34 (d, *J* = 1.3 Hz, 1H), 5.85 (d, *J* = 1.2 Hz, 1H), 5.26 (s, 1H), 3.79 (d, *J* = 0.8 Hz, 3H), 3.66 (s, 3H), 2.14 (s, 3H).

^{13}C NMR (101 MHz, $CDCl_3$): δ = 166.2, 155.3, 136.7, 129.4, 97.4, 55.7, 52.3, 18.9.

IR (Diamond-ATR, $CDCl_3$): $\tilde{\upsilon}_{max}$ = 1721 (*vs*), 1436 (*m*), 1352 (*m*), 1255 (*m*), 1203 (*m*), 1170 (*m*), 1124 (*vs*), 958 (*w*), 910 (*w*), 777 (*w*).

HRMS (ESI) calc. for $C_8H_{12}NaO_3S$ $[M+Na]^+$: 211.0399; found: 211.0399.

2,5-Dihydrothiophene S-106

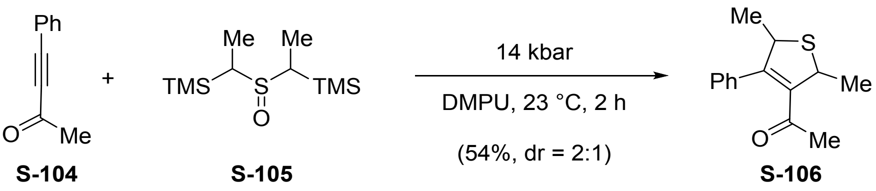

Alkyne **S-104** (225 mg, 1.50 mmol, 1 equiv) and sulfoxide **S-105** (752 mg, 3.00 mmol, 2.00 equiv) were placed in a PTFE-vial and *N,N'*-dimethylpropylenurea (3.2 mL) was added. (The reaction was conducted in three vials on 0.50 mmol scale. For detailed information about the experimental set-up and the preparation of **S-106**, see Ref 9). The vials were carefully lowered into the oil-filled compression chamber and high-pressure (14 kbar) was applied. After two hours, the reaction mixture was depressurized to atmospheric pressure and removed from the compression chamber. The reaction mixture was poured into water (5 mL), diethyl ether (5 mL) was added and the layers were separated. The aqueous layer was extracted with diethyl ether (2 × 7 mL), the combined organic layers were washed with water (3 × 10 mL). The combined organic layers were dried over sodium sulfate, the dried solution was filtered and the filtrate was concentrated. The crude product was purified by flash-column chromatography on silica gel (3% to 5% diethyl ether in *n*-pentane) to furnish 2,5-dihydrothiophene **S-106** (189 mg, 54%, dr = 2:1) as a pale-yellow oil.

TLC (5% diethyl ether in *n*-pentane): R_f = 0.35 (UV, $KMnO_4$).

^{1}H NMR (400 MHz, $CDCl_3$): δ = 7.42 – 7.35 (m, 6H), 7.23 – 7.19 (m, 2H), 7.17 (dd, *J* = 7.3, 2.2 Hz, 2H)*, 4.80 (qd, *J* = 6.7, 5.0 Hz, 1H)*, 4.67 (qd, *J* = 6.9, 5.0 Hz, 1H)*, 4.56 (qd, *J* = 6.7, 1.3 Hz, 1H), 4.41 (qd, *J* = 7.0, 1.3 Hz, 1H), 1.77 (s, 3H)*, 1.74 (s, 3H), 1.61 (d, *J* = 6.8 Hz, 3H), 1.44 (d, *J* = 6.7 Hz, 3H)*, 1.40 (d, *J* = 7.0 Hz, 3H),1.26 (d, *J* = 7.0 Hz, 3H)*.

^{13}C NMR (101 MHz, $CDCl_3$): δ = 201.1*, 199.4, 153.2, 151.3*, 143.2*, 142.8, 136.0, 135.5*, 133.1, 129.5, 128.9*, 128.8*, 128.6, 128.0*, 53.3, 52.5*, 49.7, 49.6*, 31.0*, 30.8, 26.6, 24.0, 22.9*, 22.6*.

** = NMR data of the major diastereomer.*

IR (Diamond-ATR, $CDCl_3$): $\tilde{\nu}_{max}$ = 2202 (*m*), 1669 (*s*), 1356 (*m*), 1315 (*m*), 1281 (*m*), 1238 (*m*), 1157 (*m*), 769 (*m*), 710 (*m*), 699 (*m*).

HRMS (ESI) calc. for $C_{14}H_{16}NaOS$ $[M+Na]^+$: 255.0814; found: 255.0811.

1,3-Diene S-51

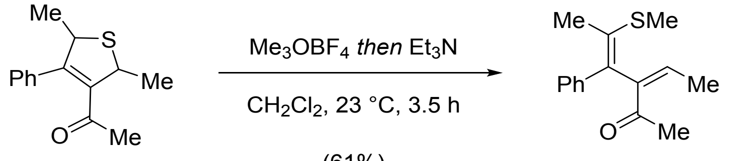

S-106 **S-51**

Following GP3 (2.5 hours for *S*-methylation), 2,5-dihydrothiophene **S-106** (111 mg, 476 µmol, dr = 2:1) was converted into 1,3-diene **S-51**. Purification by flash-column chromatography on silica gel (10% to 15% ethyl acetate in *n*-pentane) furnished 1,3-diene **S-51** (72.0 mg, 61%) as a colorless, amorphous solid.

TLC (5% ethyl acetate in *n*-pentane): R_f = 0.22 (UV, $KMnO_4$).

^{1}H NMR (400 MHz, $CDCl_3$): δ = 7.33 – 7.25 (m, 2H), 7.23 – 7.15 (m, 3H), 6.82 (q, *J* = 7.0 Hz, 1H), 2.27 (s, 6H), 2.15 (s, 3H), 1.80 (d, *J* = 7.0 Hz, 3H).

^{13}C NMR (101 MHz, $CDCl_3$): δ = 198.8, 144.5, 140.4, 139.7, 133.8, 132.1, 129.2, 128.2, 126.8, 27.1, 18.9, 15.7, 14.9.

IR (Diamond-ATR, $CDCl_3$): $\tilde{\nu}_{max}$ = 1688 (*m*), 1668 (*s*), 1615 (*m*), 1435 (*m*), 1352 (*s*), 1258 (*m*), 1220 (*m*), 1135 (*m*), 741 (*m*), 702 (*s*).

HRMS (ESI) calc. for $C_{15}H_{18}NaOS$ $[M+Na]^+$: 269.0971; found: 269.0965.

Alcohol S-107

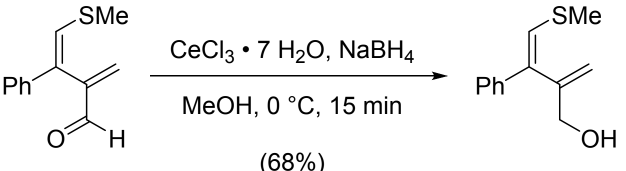

S-45 **S-107**

To a solution of aldehyde **S-45** (80 mg, 0.39 mmol, 1 equiv) in methanol (4.0 mL) were added cerium (III) chloride heptahydrate (0.16 g, 0.43 mmol, 1.1 equiv) and sodium borohydride (16 mg, 0.43 mmol, 1.1 equiv) at 0 °C. After 15 minutes, saturated aqueous sodium bicarbonate solution (10 mL) and ethyl acetate (10 mL) were added. The layers were separated and the aqueous phase was extracted with ethyl acetate (2 × 10 mL). The combined organic layers were dried over sodium sulfate, the dried solution was filtered and the filtrate was concentrated. The crude product was purified by flash-column chromatography on silica gel (10% to 20% ethyl acetate in petroleum ether) to yield alcohol **S-107** (55 mg, 68%) as a colorless oil.

TLC (25% ethyl acetate in petroleum ether): R_f = 0.50 (UV, CAM).

^{1}H NMR ($CDCl_3$, 400 MHz): δ = 7.34 – 7.20 (m, 5H), 6.44 (s, 1H), 5.64 (q, *J* = 1.6 Hz, 1H), 5.27 (q, *J* = 1.3 Hz, 1H), 4.16 (s, 2H), 2.37 (s, 3H), 1.74 (s, 1H).

^{13}C NMR ($CDCl_3$, 101 MHz): δ = 146.6, 139.2, 136.8, 128.6, 128.6, 127.2, 126.2, 116.9, 64.5, 17.8.

IR (Diamond-ATR, CH_2Cl_2): $\tilde{\upsilon}_{max}$ = 3356 (*br*), 1491 (*w*), 1441 (*w*), 1315 (*w*), 1048 (*m*), 998 (*w*), 909 (*m*), 816 (*m*), 751 (*s*), 694 (*s*).

HRMS (ESI) calc. for $C_{12}H_{15}OS$ $[M+H]^+$: 207.0838; found: 207.0837.

Methyl ether S-55

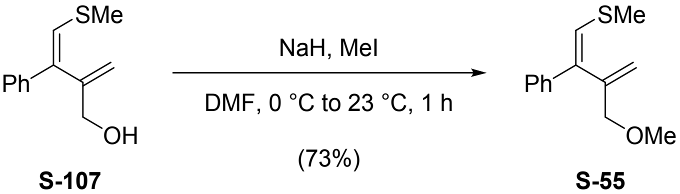

S-107 **S-55**

To a solution of alcohol **S-107** (54 mg, 0.26 mmol, 1 equiv) in *N*,*N*-dimethylformamide (1.5 mL) under argon was added sodium hydride (60% dispersion in mineral oil, 13 mg, 0.31 mmol, 1.2 equiv) at 0 °C. After 30 minutes, iodomethane (25 µL, 0.39 mL, 1.5 equiv) was added and the reaction mixture was allowed to warm to 23 °C. After 30 minutes, saturated aqueous ammonium chloride solution (10 mL) and diethyl ether (10 mL) were added, the layers were separated and the aqueous phase was extracted with diethyl ether (2 × 10 mL). The combined organic layers were dried over sodium sulfate, the dried solution was filtered and the filtrate

was concentrated under reduced pressure. The crude product was purified by flash-column chromatography on silica gel (10% diethyl ether in *n*-pentane) to yield methyl ether **S-55** (42 mg, 73%) as a colorless oil.

TLC (10% diethyl ether in petroleum ether): R_f = 0.36 (UV, CAM, $KMnO_4$).

^{1}H NMR ($CDCl_3$, 400 MHz): δ = 7.34 – 7.20 (m, 5H), 6.40 (s, 1H), 5.65 (q, *J* = 1.7 Hz, 1H), 5.33 – 5.30 (m, 1H), 3.92 (t, *J* = 1.4 Hz, 2H), 3.36 (s, 3H), 2.37 (s, 3H).

^{13}C NMR ($CDCl_3$, 101 MHz): δ = 143.7, 139.6, 137.1, 128.7, 128.5, 127.1, 126.3, 117.8, 73.5, 58.5, 17.9.

IR (Diamond-ATR, $CDCl_3$): $\tilde{\upsilon}_{max}$ = 3356 (*br*), 1491 (*w*), 1441 (*w*), 1315 (*w*), 1048 (*w*), 998 (*m*), 909 (*s*), 816 (*m*), 751 (*w*), 694 (*s*).

HRMS (ESI) calc. for $C_{13}H_{17}OS$ $[M+H]^+$: 221.0995; found: 221.0993.

Alcohol S-108

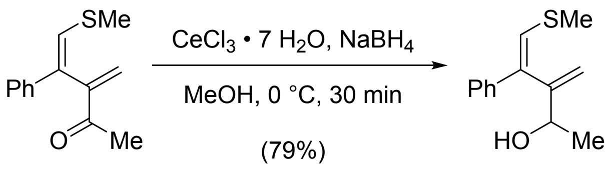

S-29 **S-108**

To a solution of 1,3-diene **S-29** (50 mg, 0.23 mmol, 1 equiv) in methanol (2.3 mL) were added cerium (III) chloride heptahydrate (87 mg, 0.23 mmol, 1.0 equiv) and sodium borohydride (9.0 mg, 0.23 mmol, 1.0 equiv) at 0 °C. After 30 minutes, saturated aqueous sodium bicarbonate solution (10 mL) and ethyl acetate (10 mL) were added. The layers were separated and the aqueous phase was extracted with ethyl acetate (2 × 10 mL). The combined organic layers were dried over sodium sulfate, the dried solution was filtered and the filtrate was concentrated. The crude product was purified by flash-column chromatography on silica gel (20% ethyl acetate in cyclohexane) to yield alcohol **S-108** (40.1 mg, 78%) as a colorless oil.

TLC (20% ethyl acetate in cyclohexane): R_f = 0.29 (UV, $KMnO_4$).

^{1}H NMR ($CDCl_3$, 400 MHz): δ = 7.36 – 7.20 (m, 5H), 6.42 (s, 1H), 5.69 (t, *J* = 1.5 Hz, 1H), 5.26 (dd, *J* = 1.5, 0.9 Hz, 1H), 4.32 (q, *J* = 6.5 Hz, 1H), 2.36 (s, 3H), 1.25 (d, *J* = 6.5 Hz, 3H).

^{13}C NMR ($CDCl_3$, 101 MHz): δ = 150.9, 139.7, 137.8, 128.7, 128.6, 127.2, 126.2, 115.6, 69.3, 22.4, 17.7.

IR (Diamond-ATR, CH_2Cl_2): $\tilde{\upsilon}_{max}$ = 3383 (*w*), 3342 (*w*), 2921 (*m*), 1444 (*w*), 1102 (*m*), 1073 (*m*), 1014 (*m*), 919 (*w*), 753 (*m*), 699 (*s*).

HRMS (ESI) calc. for $C_{13}H_{17}OS$ $[M+H]^+$: 221.0995; found: 211.0996.

Silyl ether S-60

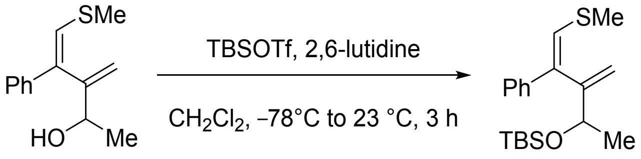

S-108 (77%) **S-60**

To a solution of 1,3-diene **S-108** (50 mg, 0.23 mmol, 1 equiv) in dichloromethane (2.1 mL) under argon were added 2,6-lutidine (47 µL, 0.41 mmol, 1.8 equiv) and *tert*-butyldimethylsilyl trifluoromethanesulfonate (63 µL, 0.27 mmol, 1.2 equiv) at –78 °C. Upon addition the reaction mixture was allowed to warm to 23 °C. After three hours, water (10 mL) and dichloromethane (10 mL) were added, the layers were separated and the aqueous phase was extracted with dichloromethane (2 × 5 mL). The combined organic layers were dried over sodium sulfate, the dried solution was filtered and the filtrate was concentrated under reduced pressure. The crude product was purified by flash-column chromatography on silica gel (1% diethyl ether in *n*-pentane) to yield silyl ether **S-60** (58 mg, 77%) as a colorless oil.

TLC (1% diethyl ether in petroleum ether): R_f = 0.33 (UV, $KMnO_4$).

^{1}H NMR ($CDCl_3$, 400 MHz): δ = 7.39 – 7.14 (m, 5H), 6.36 (s, 1H), 5.76 (t, *J* = 2.1 Hz, 1H), 5.21 (dd, *J* = 2.3, 1.4 Hz, 1H), 4.27 (qt, *J* = 6.4, 1.6 Hz, 1H), 2.36 (s, 3H), 1.17 (d, *J* = 6.4 Hz, 3H), 0.92 (s, 9H), 0.05 (s, 3H), 0.03 (s, 3H).

^{13}C NMR ($CDCl_3$, 101 MHz): δ = 150.9, 140.1, 138.0, 128.5, 128.5, 127.0, 126.3, 114.5, 69.0, 26.0, 23.5, 18.3, 17.8, –4.7, –4.7.

IR (Diamond-ATR, $CDCl_3$): $\tilde{\nu}_{max}$ = 2955 (*w*), 2927 (*w*), 1251 (*w*), 1108 (*s*), 974 (*m*), 867 (*w*), 831 (*s*), 813 (*m*), 774 (*m*), 753 (*m*).

HRMS (ESI) calc. for $C_{19}H_{30}NaOSSi$ $[M+Na]^+$: 357.1679; found: 357.1673.

5.3 Reagents

Boronic acid S-97

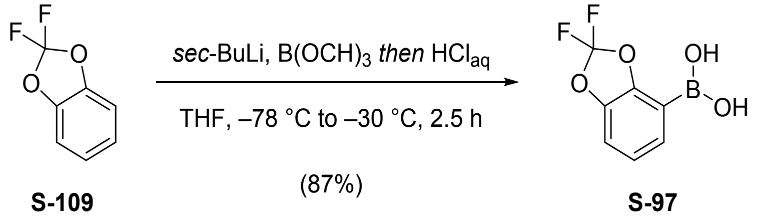

To a solution of 2,2-difluorobenzo-1,3-dioxole **S-109** (1.00 g, 6.33 mmol, 1 equiv) in tetrahydrofuran (20 mL) under argon was added *sec*-butyllithium (1.25 M, 5.06 mL, 6.33 mmol, 1.00 equiv) at –78 °C. After 1.5 hours, trimethyl borate (797 µL, 7.15 mmol, 1.13 equiv) was added and the reaction mixture was warmed to –30 °C. After one hour, the reaction mixture was warmed to 23 °C and diluted with aqueous hydrogen chloride solution (2 M, 10 mL) and water (10 mL). The aqueous layer was extracted with ethyl acetate (3 × 20 mL), the combined organic layers were dried over sodium sulfate, the dried solution was filtered and the filtrate was concentrated under reduced pressure. The crude product was purified by flash-column chromatography on silica gel (20% ethyl acetate in *n*-pentane) to furnish boronic acid **S-97** (1.12 g, 87%) as a colorless solid, which was used in the next step without further purification. The obtained analytical data were in full agreement with those reported in literature.[10]

TLC (20% ethyl acetate in *n*-pentane): R_f = 0.18 (UV, $KMnO_4$).

O-Mesitylenesulfonyl hydroxylamine (MSH)

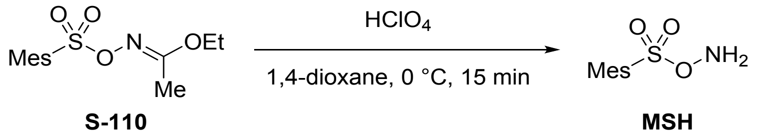

O-Mesitylenesulfonyl hydroxylamine (**MSH**) was prepared following a procedure of Bolm and coworkers.[11] To a suspension of ethyl *O*-(mesitylenesulfonyl)acetohydroxamate (**S-110**, 285 mg, 999 µmol, 1 equiv) in 1,4-dioxane (1.0 mL) was added dropwise perchloric acid (70%, 1.0 mL, 12 mmol, 12 equiv) at 0 °C. After 15 minutes, precooled water (10 mL) and dichloromethane (4 mL) were added and the layers were separated. The aqueous layer was extracted with dichloromethane (3 × 3 mL), the combined organic layers were washed with saturated aqueous sodium chloride solution (10 mL) and the washed solution was dried over magnesium sulfate. The dried solution was filtered to furnish a solution of **MSH** (0.08 M in dichloromethane) which was used in the next step without further purification.

Sodium chloro((4-nitrophenyl)sulfonyl)amide (chloramine-N)

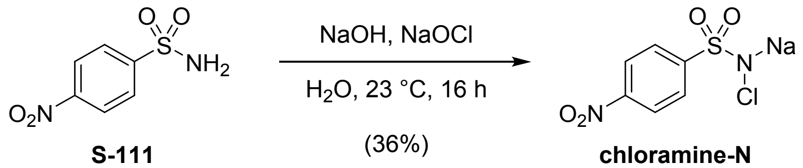

S-111 **chloramine-N**

To a solution of 4-nitrobenzenesulfonamid (**S-111**, 404 mg, 2.00 mmol, 1 equiv) in water (3 mL) were added sodium hydroxide (800 mg, 20.0 mmol, 10.0 equiv) and an aqueous sodium hypochlorite solution (5%, 2.47 mL, 2.00 mmol, 1.00 equiv) at 23 °C. After 16 hours, the reaction mixture was filtered, washed with water (2 × 3 mL) and dried *in vacuo* to yield **chloramine-N** (184 mg, 36%) as a pale-yellow solid, which was used in the next step without further purification. The obtained analytical data were in full agreement with those reported in literature.[12]

HRMS (ESI) calc. for $C_6H_4ClN_2O_4S$ $[M–Na]^-$: 234.9586; found: 234.9590.

HRMS (ESI) calc. for $C_6H_4ClN_2Na_2O_4S$ $[M+Na]^+$: 280.9370; found: 280.9368.

Sodium chloro((4-methoxyphenyl)sulfonyl)amide (chloramine-P)

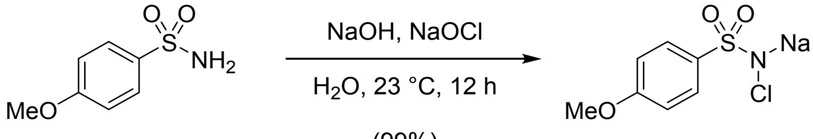

S-112 **chloramine-P**

To a solution of 4-methoxybenzenesulfonamid (**S-112**, 374 mg, 2.00 mmol, 1 equiv) in water (4 mL) were added sodium hydroxide (800 mg, 20.0 mmol, 10.0 equiv) and an aqueous sodium hypochlorite solution (5%, 2.47 mL, 2.00 mmol, 1.00 equiv) at 23 °C. After twelve hours, the reaction mixture was filtered, washed with water (2 × 5 mL) and dried *in vacuo* to yield **chloramine-P** (487 mg, 99%) as a yellow solid, which was used in the next step without further purification. The obtained analytical data were in full agreement with those reported in literature.[13]

HRMS (ESI) calc. for $C_7H_7ClNO_3S$ $[M–Na]^-$: 219.9841; found: 219.9837.

HRMS (ESI) calc. for $C_7H_8ClN_2NaO_3S$ $[M+H]^+$: 243.9806; found: 243.9803.

Sodium chloro(methylsulfonyl)amide (chloramine-M)

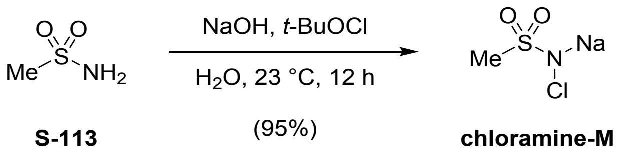

To a solution of methanesulfonamide (**S-113**, 501 mg, 5.27 mol, 1 equiv) in water (4 mL) were added sodium hydroxide (211 mg, 5.27 mol, 1.00 eqiuv) and *tert*-butyl hypochlorite (597 µL, 5.27 mol, 1.00 equiv) at 23 °C. After twelve hours, the solvents were removed under reduced pressure to furnish **chloramine-M** (756 mg, 95%) as a colorless solid, which was used in the next step without further purification. The obtained analytical data were in full agreement with those reported in literature.[14]

HRMS (ESI) calc. for CH_3ClNO_2S [M–Na]⁻: 127.9579, found: 127.9566.

HRMS (ESI) calc. for $CH_3ClNNa_2O_2S$ $[M+Na]^+$: 173.9363; found: 173.9357.

6 Computational Studies

6.1 Computational Methodology

All calculations were carried out with the Gaussian 16 package.[15] Investigated structures were fully optimized in implicit solvent with the B3LYP hybrid density functional[16–19] and the 6-311G++(2d,2p)[20] basis set on all atoms. Bulk solvent effects were implicitly modelled by Integral Equation Formalism Polarizable Continuum Model (IEFPCM) of Tomasi and Pascual-Ahuir[21–23] as implemented in Gaussian 16. The internally stored parameters for acetonitrile (ε = 35.688) were used. All species were calculated considering a closed-shell electronic configurations (singlet). Empirical dispersion correction of the D3-generation with Becke-Johnson damping[24] were added as implemented in Gaussian 16. Frequency analyses were carried out at the same level as the geometry optimizations and the nature of the stationary points was determined by analyses of the Hessian matrix. (Local) energy minima were confirmed to show only real eigenvalues, whereas transition states were confirmed to have one imaginary eigenvalue, while the corresponding eigenvector coincided with the change in the reaction coordinate. Zero-point energy and thermal corrections were calculated using the standard rigid-rotator/harmonic oscillator model to obtain Gibbs free energies at 298.15 K, no scaling of the frequencies was applied. The possibility of different conformations was considered for all structures. Structures were visualized by using CylView.[25]

6.2 Standard Reaction Pathway

In addition to the energetically favored pathway for the conversion of 1,3-diene **5a** to 2,5-dihydropyrrole **10** discussed in the main manuscript (highlighted in black), we found an alternative reaction pathway that is higher in energy (highlighted in red, Scheme S-1).

Scheme S-1. Predicted standard reaction pathways.

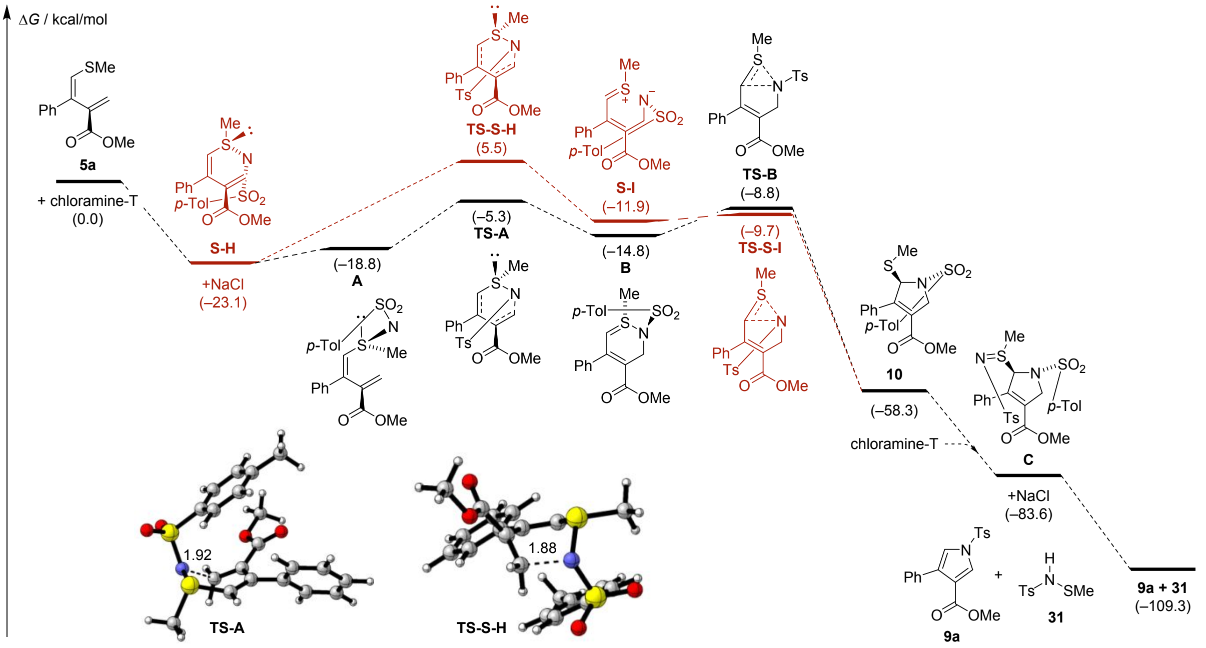

The favored reaction pathway is highlighted in black and the unfavored pathway is highlighted in red. Relative Gibbs free energies at 298 K are given in kcal/mol, whereas the energy of **5a** plus chloramine-T is arbitrarily set to zero. All calculations were carried out with B3LYP-D3/6-311++G(2d,2p) in acetonitrile treated as implicit solvent.

The initial reaction of 1,3-diene **5a** with chloramine-T generates a sulfilimine intermediate **S-H**, which was identified as thermodynamically preferred to its conformer **A** (ΔΔ*G* = –4.3 kcal/mol). However, the reaction barrier of Δ$G^‡$ = 28.6 kcal/mol for the 6π-electrocyclization (**TS-S-H**, "boat-like") is kinetically hindered at standard reaction conditions (23 °C, ambient pressure). Following **TS-S-H**, the charge-separated intermediate **S-I** is formed, in which the S–N bond is heterolytically cleaved in contrast to the energetically lower 2,3-dihydrothiazine **B** (ΔΔ*G* = +2.9 kcal/mol). Rapid ring-contraction (**TS-S-I**) with a low barrier of Δ$G^‡$ = 2.2 kcal/mol finally delivers 2,5-dihydropyrrole **10** following a comparable thermodynamic profile as the ring-contraction (**TS-B**) of intermediate **B**. Since no sulfilimine intermediate is experimentally observed at 23 °C, rapid conversion of **S-H** to **A** via C–S bond rotation with a low barrier can be assumed.

6.3 Influence of Sterics

In addition to the energetically favored pathway for the conversion of 1,3-diene **18** to 2,5-dihydropyrrole **E** discussed in the main manuscript (highlighted in black), we found alternative reaction pathways for the formation of **E** (highlighted in red) and its diastereomer **S-M** (highlighted in blue, Scheme 2).

Scheme S-2. Predicted reaction pathways of 18 to 2,5-dihydropyrroles E or S-M.

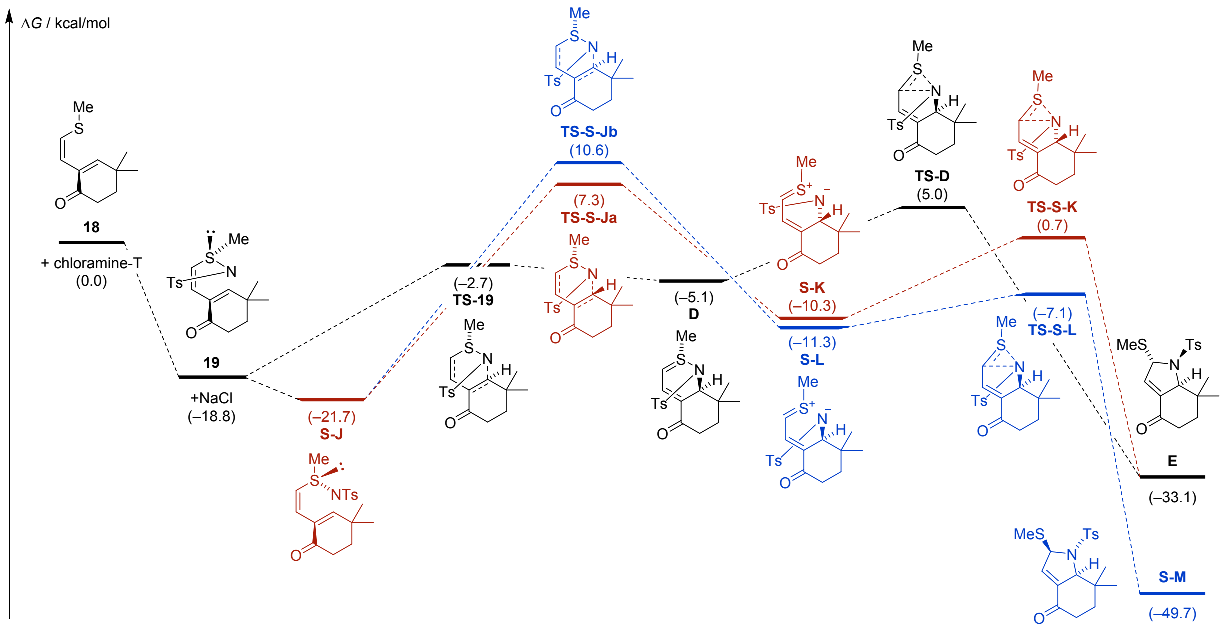

The favored reaction pathway is highlighted in black and the unfavored pathways are highlighted in red and blue. Relative Gibbs free energies at 298 K are given in kcal/mol, whereas the energy of **18** plus chloramine-T is arbitrarily set to zero. All calculations were carried out with B3LYP-D3/6-311++G(2d,2p) in acetonitrile treated as implicit solvent.

Both alternative pathways commence with the initial formation of sulfilimine **S-J**, which is ΔΔG = –2.9 kcal/mol lower in energy than its conformer **19**. Following the red pathway, the 6π-electrocyclization via **TS-S-Ja** is kinetically disfavored by $\Delta\Delta G^‡$ = 10.0 kcal/mol compared to **TS-19**. However, intermediate **S-K** is thermodynamically preferred over 2,3-dihydrothiazine **D** ($\Delta\Delta G$ = –5.2 kcal/mol). Final ring-contraction via **TS-S-K** ($\Delta G^‡$ = 11.0 kcal/mol) follows a similar energetic profile as shown for the favored pathway (**D**→**E**). Alternatively, sulfilimine **S-J** can undergo an alternative, less favorable 6π-electrocyclization through **TS-S-Jb** ($\Delta G^‡$ = 32.3 kcal/mol) entering the path to 2,5-dihydropyrrole **S-M** (highlighted in blue). After the endergonic formation of charge-separated intermediate **S-L** ($\Delta\Delta G$ = 10.4 kcal/mol), the low activation barrier of $\Delta G^‡$ = 4.2 kcal/mol for the ring-contraction step (**TS-S-L**) favors the highly exergonic formation of **S-M** (ΔG = –38.4 kcal/mol). In contrast to the favored pathway, the high activation barriers for the 6π-electrocyclization (**TS-S-Ja**, **TS-S-Jb**) make these pathways unlikely. However,

thermal activation (111 °C) of sulfilimine **25** lead to the isolation of both 2,5-dihydropyrroles (**26** and **S-57**, dr = 4:1, see Scheme 4A and Supporting Section 4.3).

6.4 Influence of Electronics

In addition to the energetically lowest pathway for the conversion of 1,3-diene **S-55** to 2,5-dihydropyrrole **G** discussed in the main manuscript (highlighted in black), we found an alternative reaction pathway that is higher in energy (highlighted in red, Scheme S-3).

Scheme S-3. Predicted reaction pathways of S-55 to 2,5-dihydropyrrole G.

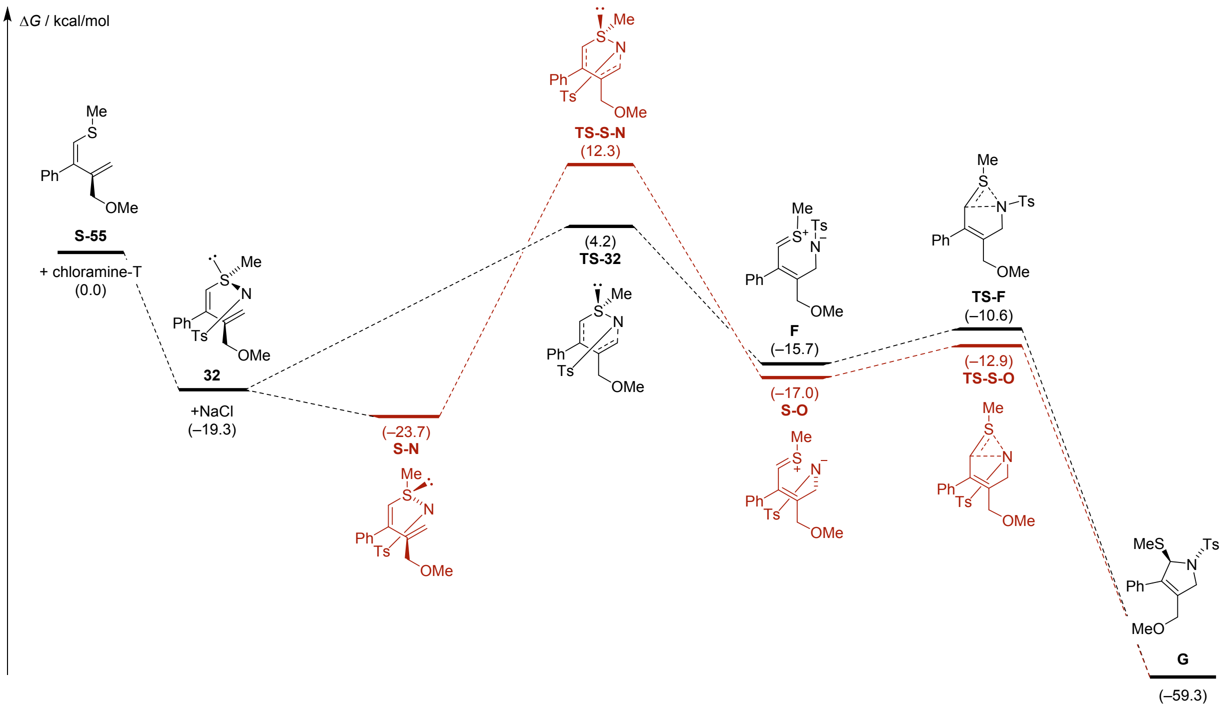

The favored reaction pathway is highlighted in black and the unfavored pathway is highlighted in red. Relative Gibbs free energies at 298 K are given in kcal/mol, whereas the energy of **S-55** plus chloramine-T is arbitrarily set to zero. All calculations were carried out with B3LYP-D3/6-311++G(2d,2p) in acetonitrile treated as implicit solvent.

Similar to the standard reaction pathway (**5a**→**9a**, Scheme S-1), the initial reaction of 1,3-diene **S-55** with chloramine-T generates sulfilimine intermediate **S-N**, which is thermodynamically preferred to its conformer **32** ($\Delta\Delta G$ = –4.4 kcal/mol). Again, the reaction barrier for its corresponding 6π-electrocyclization (**TS-S-N**) is $\Delta\Delta G^{‡}$ = 8.1 kcal/mol higher than **TS-32**. In respect to the Curtin–Hammett principle, **TS-32** represents the favored pathway since sulfilimines **32** and **S-N** equilibrate through C–S bond rotation. Following **TS-S-N**, charge-separated intermediate **S-O** is formed, which is marginally higher in energy than the 2,3-dihydrothiazine intermediate **F** ($\Delta\Delta G$ = 1.3 kcal/mol). Ring-contraction (**TS-S-O**) follows an equal thermodynamic profile as the ring-contraction (**TS-F**) of intermediate **F** cumulating in the exergonic formation (ΔG = –42.3 kcal/mol) of 2,5-dihydropyrrole **G**. The predicted reaction

barriers are in line with the experimental data obtained for sulfilimine **S-55**, which could be isolated at 23 °C. Thermal activation (111 °C) of **S-55** (or its conformer **S-N**) directly led to the quantitative conversion to pyrrole **24** through the release of methanethiol from intermediate **G** (See Supporting Section 4.3).

6.5 Comparison of Transition States

For a detailed analysis of the energetically favored reaction pathways (Scheme S-4), the transition state structures of the 6π-electrocyclization (**TS-A**, **TS-19** and **TS-32**) as well as the ring-contraction (**TS-B**, **TS-D** and **TS-F**) were studied in respect to steric as well as electronic influences.

Scheme S-4. Comparison of the favorable reaction pathways.

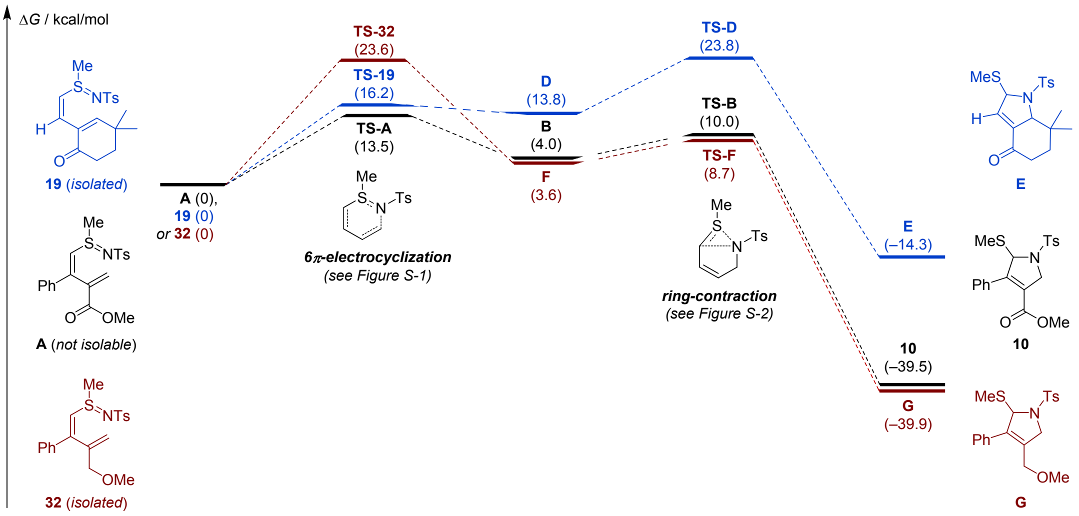

The standard reaction pathway (**A**→**10**) is highlighted in black, the sterically increased reaction pathway is highlighted in blue (**19**→**E**) and the reaction pathway lacking an electron-withdrawing group is highlighted in red (**32**→**G**). Relative Gibbs free energies at 298 K are given in kcal/mol, whereas the energy of the sulfilimines **A**, **19** or **32** is arbitrarily set to zero. All calculations were carried out with B3LYP-D3/6-311++G(2d,2p) in acetonitrile treated as implicit solvent.

All three favored transition states for the 6π-electrocyclization (**TS-A**, **TS-19** and **TS-32**) have a very similar conformation ("half-chair like", Figure S-1). Minor differences in the Gibbs energy between **TS-A** and **TS-19** ($\Delta\Delta G^{\ddagger}$ = 2.7 kcal/mol) might result from additional steric repulsion with the substituted cyclohexenone. However, the lack of an electron withdrawing group (**TS-32**) significantly increases the activation barrier ($\Delta\Delta G^{\ddagger}$ = 10.1 kcal/mol) compared to **TS-A**.

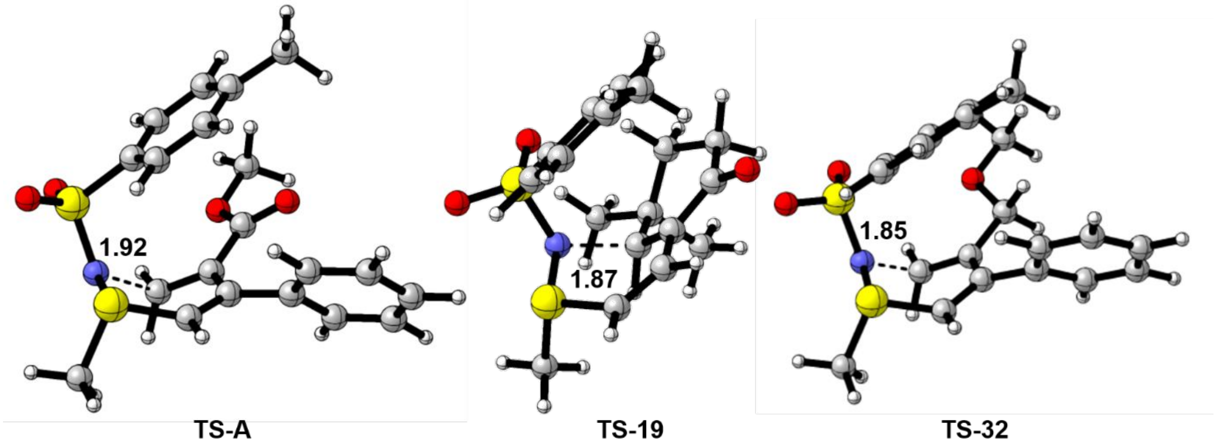

Figure S-1. Comparison of the transition states **TS-A**, **TS-19** and **TS-32** for the 6π-electrocyclization.

For the ring-contraction (Figure S-2), **TS-B** and **TS-F** adopt the same conformation with nearly identical distances between the participating atoms. In contrast to the 6π-electrocyclization step, the impact of the EWG or the lack thereof is negligible with regards to the similar free energy profiles (**B**→**10** and **F**→**G**). When comparing **TS-D** to **TS-B**, increased steric demand leads to elongated atom distances ($\Delta d_{N\text{–}S} = 0.78$ Å, $\Delta\delta_{C\text{–}N} = 0.53$ Å) resulting in an energetically higher reaction barrier of $\Delta\Delta G^{\ddagger} = 13.8$ kcal/mol.

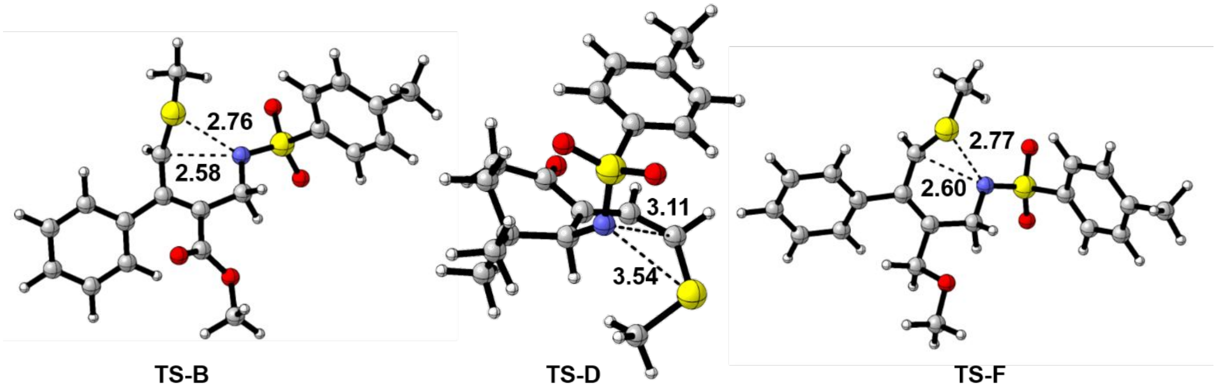

Figure S-2. Comparison of the transition states **TS-B**, **TS-D** and **TS-F** for the ring-contraction.

6.6 Cartesian Coordinates

1,3-Diene 5a

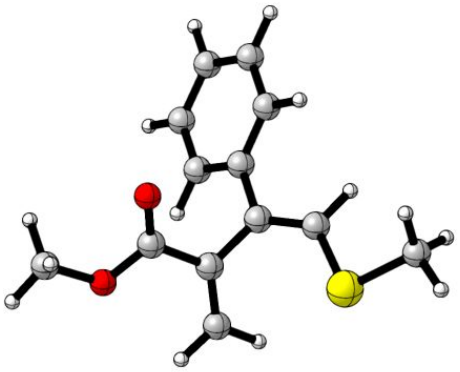

IEFPCM(MeCN)B3LYP-D3/6-311++G(2d,2p) Electronic Energy = –1052.728012

IEFPCM(MeCN)B3LYP-D3/6-311++G(2d,2p) Free Energy = –1052.536818

Number of imaginary frequencies = 0

C	0.452698	-0.373804	0.282036	C	-3.137308	-1.375875	0.905240
C	1.623004	-1.013902	0.118896	H	-1.816351	0.137570	1.644338
H	1.627457	-2.080556	-0.056931	C	-3.287293	-2.466975	0.055799
C	0.388068	1.077122	0.576162	H	-2.322416	-3.709336	-1.408366
C	-0.419574	1.897878	-0.377148	H	-3.964181	-1.049821	1.520825
O	-0.709088	1.530802	-1.496569	H	-4.231272	-2.990516	0.001102
O	-0.776026	3.089035	0.123068	S	3.226973	-0.299717	0.190509
C	-1.496402	3.959784	-0.772802	C	4.186215	-1.608610	-0.647085
H	-1.693411	4.860186	-0.202381	H	5.223794	-1.286973	-0.621076
H	-0.889978	4.184711	-1.646549	H	4.087478	-2.549741	-0.113254
H	-2.426945	3.491439	-1.082945	H	3.864317	-1.717259	-1.678550
C	-0.832574	-1.103525	0.190432	C	1.048713	1.654337	1.582080
C	-1.002009	-2.195644	-0.670500	H	1.003751	2.719949	1.745028
C	-1.923376	-0.701291	0.970974	H	1.648610	1.066937	2.261608
C	-2.212283	-2.872753	-0.732305				
H	-0.188774	-2.500269	-1.313667				

Pyrrole 9a

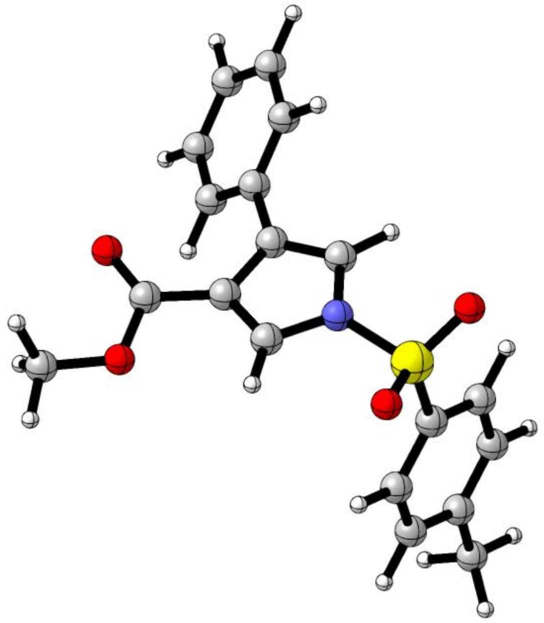

IEFPCM(MeCN)B3LYP-D3/6-311++G(2d,2p) Electronic Energy = –1488.532766

IEFPCM(MeCN)B3LYP-D3/6-311++G(2d,2p) Free Energy = –1488.265792

Number of imaginary frequencies = 0

C	-0.480281	-0.657032	-1.136581	H	3.152627	1.869107	0.285047
C	-1.590121	-0.262821	-0.439244	C	3.834708	-1.885547	0.89452
C	-1.404032	1.138595	-0.124053	H	2.785165	-2.223419	-0.950995
C	-0.191218	1.529656	-0.641267	C	4.293568	-0.947	1.820903
N	0.358084	0.443494	-1.276438	H	4.394401	1.149307	2.287802
H	-0.205072	-1.616026	-1.5327	H	4.032016	-2.935766	1.055849
H	0.297235	2.484772	-0.641523	C	-2.359498	2.056315	0.506574
C	-2.701879	-1.160706	-0.084999	O	-1.782751	3.233678	0.820562
C	-3.232602	-2.031924	-1.041648	C	-2.652897	4.229639	1.391499
C	-3.210114	-1.204027	1.217275	H	-2.023056	5.093696	1.570384
C	-4.244895	-2.924749	-0.705598	H	-3.451348	4.477141	0.696231
H	-2.857938	-1.998422	-2.055165	H	-3.079019	3.868816	2.324232
C	-4.220826	-2.094963	1.55242	O	-3.531125	1.817818	0.71472
H	-2.806998	-0.541662	1.968649	O	1.965529	-0.68145	-2.838859
C	-4.743432	-2.95875	0.59236	O	2.302125	1.73406	-2.187724
H	-4.646552	-3.587553	-1.459336	C	5.042639	-1.37435	3.051577
H	-4.599605	-2.11816	2.564791	H	5.272625	-2.437019	3.027716
H	-5.532019	-3.649944	0.854685	H	5.975831	-0.819822	3.151024
C	2.889995	-0.140195	-0.438337	H	4.452038	-1.174525	3.947209
C	3.339276	0.82155	0.463496	S	1.967367	0.370445	-1.849828
C	3.13152	-1.493598	-0.236104				
C	4.037586	0.409082	1.585345				

Dihydropyrrole 10

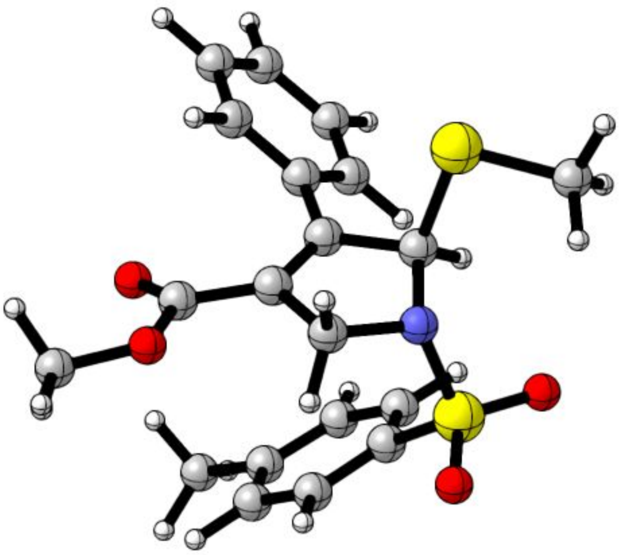

IEFPCM(MeCN)B3LYP-D3/6-311++G(2d,2p) Electronic Energy = –1927.298248

IEFPCM(MeCN)B3LYP-D3/6-311++G(2d,2p) Free Energy = –1926.984316

Number of imaginary frequencies = 0

C	0.777562	-0.676197	0.447138
C	0.219162	0.052408	1.429503
N	-1.472597	-1.389804	0.661643
C	0.740342	1.280680	2.052417
O	-0.015232	1.619169	3.114123
O	1.681719	1.940531	1.667923
C	0.350492	2.839814	3.788608
H	0.276648	3.680699	3.103712
H	1.364664	2.767280	4.172831
H	-0.359765	2.944236	4.600554
S	-2.625829	-0.777543	-0.403924
C	-1.957871	0.740469	-1.027184
C	-1.144485	0.713439	-2.155791
C	-2.138324	1.917410	-0.306003
C	-0.483192	1.869383	-2.541365
H	-1.039283	-0.195276	-2.727603
C	-1.467141	3.063291	-0.704478
H	-2.795493	1.938112	0.549093
C	-0.617398	3.054770	-1.814050
H	0.149745	1.849143	-3.417474
H	-1.604011	3.977573	-0.144132
O	-2.704708	-1.737710	-1.488289
O	-3.790391	-0.480079	0.406794
C	0.150293	4.286520	-2.203802
H	1.120705	4.299449	-1.702921
H	-0.380243	5.191977	-1.915026
H	0.334433	4.315570	-3.276200
C	-1.141868	-0.496319	1.796405
H	-1.903662	0.262700	1.930844
H	-1.104022	-1.083606	2.714307
C	-0.191862	-1.766493	0.042068
H	-0.303338	-1.881608	-1.030246
S	0.416263	-3.385741	0.683571
C	-0.841813	-4.466563	-0.070645
H	-1.833657	-4.153322	0.241909
H	-0.643303	-5.471246	0.292972
H	-0.762025	-4.447460	-1.154241
C	2.101274	-0.596571	-0.178336
C	2.232982	-0.766736	-1.561550
C	3.254528	-0.403403	0.589488
C	3.482052	-0.710377	-2.165571
H	1.357680	-0.925491	-2.172907
C	4.503734	-0.365040	-0.012704
H	3.168227	-0.295778	1.659388
C	4.621600	-0.510409	-1.392866
H	3.564678	-0.828055	-3.236623
H	5.386510	-0.223732	0.594628
H	5.595222	-0.475729	-1.860834

1,3-Diene 18

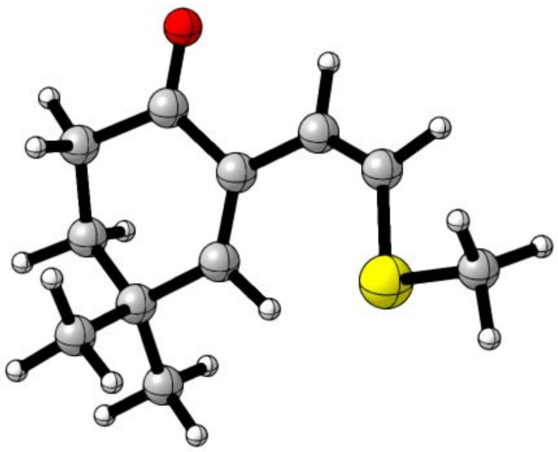

IEFPCM(MeCN)B3LYP-D3/6-311++G(2d,2p) Electronic Energy = –-902.452440

IEFPCM(MeCN)B3LYP-D3/6-311++G(2d,2p) Free Energy = –-902.250201

Number of imaginary frequencies = 0

C	-1.078267	1.486228	0.385905	H	2.665305	0.950602	-1.528441
C	-2.315412	0.97244	0.306577	H	3.475475	1.814709	-0.237505
H	-3.159863	1.629773	0.462765	C	2.905644	-0.226164	0.273615
S	-2.778013	-0.690418	-0.025581	C	1.849014	-2.35251	1.086311
C	-4.562603	-0.467724	-0.336965	C	1.762318	-1.805014	-1.357791
H	-4.966935	-1.459991	-0.516965	H	3.8376	-0.683556	-0.059967
H	-5.044681	-0.033298	0.534398	H	3.010952	-0.033362	1.343407
H	-4.719965	0.154447	-1.213025	H	1.791005	-1.973725	2.106894
C	0.214008	0.833592	0.200844	H	1.04834	-3.079063	0.944594
C	1.362925	1.741039	-0.081022	H	2.801771	-2.869987	0.968375
C	0.427119	-0.499758	0.260568	H	2.67642	-2.381994	-1.503227
O	1.238449	2.958128	-0.059265	H	0.911922	-2.470023	-1.507271
C	2.681017	1.100254	-0.444645	H	1.717375	-1.02942	-2.120623
H	-0.407752	-1.155222	0.470187	H	-1.018185	2.548281	0.578137
C	1.741207	-1.206801	0.066882				

Sulfilimine 19

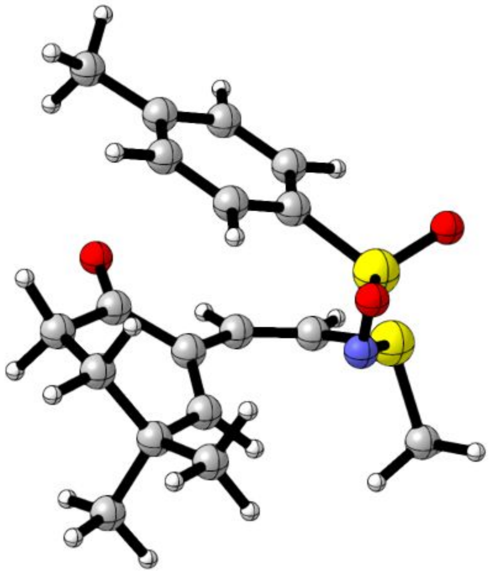

IEFPCM(MeCN)B3LYP-D3/6-311++G(2d,2p) Electronic Energy = –1776.959393

IEFPCM(MeCN)B3LYP-D3/6-311++G(2d,2p) Free Energy = –1776.634870

Number of imaginary frequencies = 0

C	0.265237	0.652845	2.305932	O	1.654481	-1.002350	-2.583342
C	1.481281	0.113473	2.408750	N	2.088725	-0.074605	-0.350188
H	1.826237	-0.265974	3.361773	H	-0.319911	0.634448	3.218077
S	2.659648	-0.369122	1.155195	C	-0.423460	1.257200	1.168105
C	3.902032	0.936887	1.325086	C	-1.899870	1.140014	1.159034
H	4.723232	0.664528	0.669413	C	0.197614	2.033401	0.258403
H	4.222864	0.951394	2.362852	O	-2.495233	0.543149	2.046279
H	3.460156	1.883910	1.034376	C	-2.652111	1.858392	0.062669
S	1.655035	-1.408056	-1.182929	C	-0.453875	2.729617	-0.898299
C	-0.034874	-1.751571	-0.731245	H	1.251545	2.235062	0.380638
C	-0.305663	-2.347673	0.497138	H	-3.551324	1.284406	-0.154964
C	-1.067999	-1.393201	-1.583558	H	-2.986736	2.805466	0.496205
C	-1.620967	-2.554416	0.877598	C	-1.824285	2.102657	-1.194522
H	0.500642	-2.646781	1.149874	C	-0.588102	4.225189	-0.533665
C	-2.383832	-1.610996	-1.190792	C	0.453606	2.593804	-2.133531
H	-0.842348	-0.950211	-2.540782	H	-1.662913	1.151175	-1.697319
C	-2.681513	-2.177773	0.047443	H	-2.374746	2.738948	-1.887861
H	-1.830434	-3.005619	1.837694	H	-0.992598	4.773167	-1.385121
H	-3.188622	-1.324330	-1.853835	H	0.383429	4.651978	-0.284604
C	-4.103946	-2.341388	0.503548	H	-1.249027	4.373550	0.319043
H	-4.240547	-3.272004	1.053099	H	-0.013093	3.087242	-2.986724
H	-4.795366	-2.329369	-0.336924	H	0.622307	1.547901	-2.382473
H	-4.374152	-1.521966	1.172774	H	1.422947	3.060716	-1.957485
O	2.440353	-2.586965	-0.810588				

Transition State TS-19

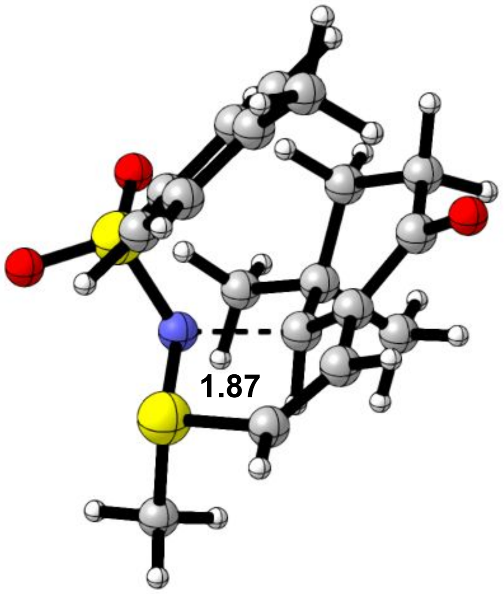

IEFPCM(MeCN)B3LYP-D3/6-311++G(2d,2p) Electronic Energy = –1776.935899

IEFPCM(MeCN)B3LYP-D3/6-311++G(2d,2p) Free Energy = –1776.609092

Number of imaginary frequencies = 1 (341.19i cm^{-1})

C	0.107477	0.286409	-2.138152	O	-0.593563	-0.566982	2.502111
C	-0.002543	-1.051600	-2.355641	N	-1.123083	-1.231495	0.153645
H	0.587502	-1.577661	-3.089982	H	0.824991	0.825288	-2.744509
S	-0.636317	-2.166476	-1.148547	C	-0.596942	1.023449	-1.156975
C	-2.204729	-2.815149	-1.782953	C	-0.175649	2.347754	-0.797663
H	-2.577037	-3.508621	-1.034405	C	-1.729720	0.392868	-0.558464
H	-1.959889	-3.336814	-2.705172	O	0.748468	2.945098	-1.375142
H	-2.901993	-2.006541	-1.968909	C	-0.932838	3.066250	0.308285
S	-0.031700	-1.376038	1.439758	C	-2.635674	1.171598	0.376474
C	1.505262	-0.655661	0.932201	H	-2.324258	-0.175687	-1.263116
C	2.416338	-1.425063	0.215797	H	-0.197289	3.603922	0.906658
C	1.768589	0.676384	1.226821	H	-1.538915	3.833809	-0.179764
C	3.592288	-0.837971	-0.226821	C	-1.804147	2.172017	1.190828
H	2.214803	-2.465970	0.016483	C	-3.623339	1.920719	-0.552932
C	2.951878	1.245071	0.783442	C	-3.461736	0.260016	1.290388
H	1.063041	1.254398	1.800698	H	-1.181242	1.613787	1.885547
C	3.874288	0.504252	0.040255	H	-2.473680	2.787450	1.793653
H	4.303255	-1.432406	-0.783549	H	-4.276841	2.550420	0.050820
H	3.158882	2.280752	1.012850	H	-4.246366	1.212296	-1.099019
C	5.132440	1.144873	-0.476249	H	-3.114658	2.550607	-1.279344
H	5.910438	0.404863	-0.653958	H	-4.202292	0.858917	1.821351
H	5.511822	1.889713	0.221584	H	-2.836706	-0.244788	2.018916
H	4.937164	1.653901	-1.422550	H	-3.992572	-0.494047	0.708244
O	0.191874	-2.794890	1.665624				

Tosylamide 31

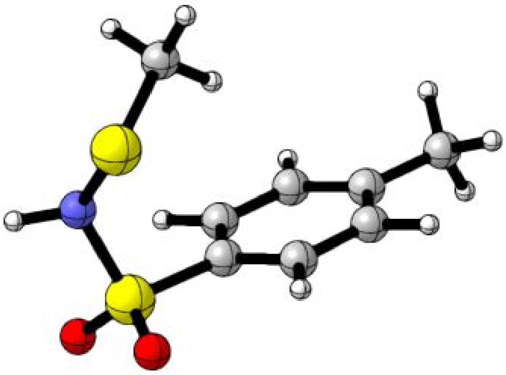

IEFPCM(MeCN)B3LYP-D3/6-311++G(2d,2p) Electronic Energy = –-1313.294233

IEFPCM(MeCN)B3LYP-D3/6-311++G(2d,2p) Free Energy = –-1313.154499

Number of imaginary frequencies = 0

C	3.001222	0.038178	0.042110	H	4.682502	1.067041	-0.817517
C	2.265120	0.051784	1.227534	O	-1.837334	-1.291366	1.417806
C	0.925064	-0.315008	1.244415	O	-1.681178	-2.150272	-0.927625
C	0.317565	-0.695358	0.056122	S	-1.402674	-1.104564	0.044478
C	1.025786	-0.718489	-1.142617	S	-2.060877	1.734273	0.288725
C	2.361182	-0.354326	-1.140175	C	-0.772577	2.670424	-0.587728
H	2.742139	0.355715	2.148691	H	-1.053234	2.819354	-1.625791
H	0.357147	-0.299709	2.160928	H	-0.719784	3.632303	-0.079835
H	0.536226	-1.011466	-2.058893	H	0.184854	2.164915	-0.509345
H	2.916873	-0.369031	-2.067527	N	-2.060616	0.284650	-0.652032
C	4.455289	0.419193	0.028544	H	-2.950784	0.065623	-1.086341
H	4.739485	0.931747	0.945012				
H	5.081885	-0.469662	-0.067097				

Sulfilimine 32

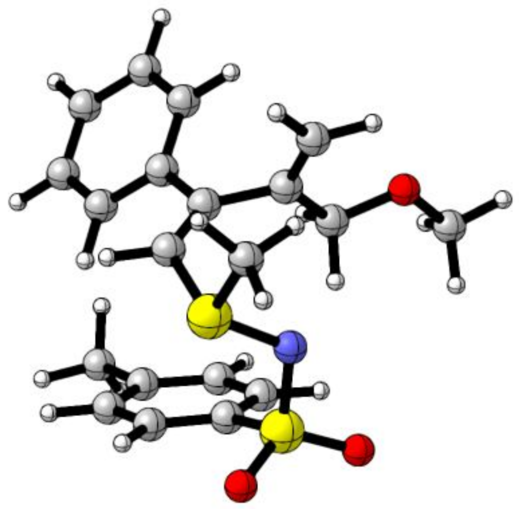

IEFPCM(MeCN)B3LYP-D3/6-311++G(2d,2p) Electronic Energy = –1853.163783

IEFPCM(MeCN)B3LYP-D3/6-311++G(2d,2p) Free Energy = –1852.832228

Number of imaginary frequencies = 0

C	-0.803891	-1.190579	-0.864485	H	1.130094	1.124176	2.407811
C	-0.347946	-2.187300	0.140661	C	-0.219061	3.216757	-0.536344
C	-0.010873	-0.677245	-1.815557	H	1.530441	2.565426	-1.600521
H	-0.399403	-0.018626	-2.577562	C	-0.952904	3.161468	0.649204
C	-2.223190	-0.770328	-0.786149	H	-0.990759	2.358297	2.641381
C	-3.197846	-1.654544	-0.308097	H	-0.585378	3.810274	-1.362744
C	-2.613861	0.519687	-1.167144	C	-2.266511	3.880053	0.786154
C	-4.529354	-1.266477	-0.236586	H	-2.447830	4.539867	-0.059707
H	-2.909767	-2.649721	-0.003403	H	-3.088412	3.163788	0.836026
C	-3.944345	0.906373	-1.092104	H	-2.297392	4.473429	1.699991
H	-1.869041	1.231519	-1.486359	O	3.474978	0.487609	1.473495
C	-4.907803	0.014033	-0.629413	O	3.726230	1.408119	-0.841054
H	-5.270962	-1.963347	0.127420	N	2.355157	-0.724498	-0.336734
H	-4.226758	1.908808	-1.380677	C	-0.142622	-3.455207	-0.202065
H	-5.943124	0.317731	-0.566084	H	0.126915	-4.190455	0.540691
S	1.773886	-0.706473	-1.884775	H	-0.271257	-3.784853	-1.222299
C	2.199943	-2.377637	-2.420077	C	-0.238885	-1.682092	1.549381
H	3.264113	-2.362894	-2.635022	O	-0.064597	-2.747834	2.460372
H	1.962372	-3.063896	-1.615880	C	0.179695	-2.276925	3.778339
H	1.624716	-2.592633	-3.315952	H	0.297837	-3.149954	4.414609
S	2.894317	0.733170	0.157455	H	1.091442	-1.673864	3.816761
C	1.444528	1.751643	0.382161	H	-0.657963	-1.674590	4.142957
C	0.744160	1.701980	1.582701	H	-1.134120	-1.101832	1.804271
C	0.973098	2.516484	-0.677424	H	0.614673	-1.001233	1.598376
C	-0.445774	2.402480	1.708119				

Transition State TS-32

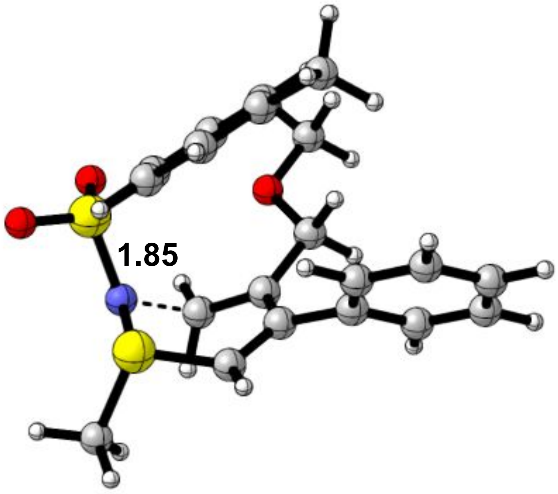

IEFPCM(MeCN)B3LYP-D3/6-311++G(2d,2p) Electronic Energy = –1853.129462

IEFPCM(MeCN)B3LYP-D3/6-311++G(2d,2p) Free Energy = –1852.794626

Number of imaginary frequencies = 1 (437.20i cm^{-1})

C	-0.586672	-1.498707	-0.502896
C	-0.011288	-1.657758	0.761205
C	0.151303	-1.508203	-1.687062
H	-0.339530	-1.345693	-2.635278
C	-2.045213	-1.230194	-0.635310
C	-2.986758	-2.077356	-0.044404
C	-2.497987	-0.114880	-1.345059
C	-4.347326	-1.815242	-0.158346
H	-2.648872	-2.950661	0.495444
C	-3.857663	0.153522	-1.453406
H	-1.776116	0.552852	-1.790717
C	-4.787125	-0.696128	-0.860376
H	-5.063366	-2.484816	0.297633
H	-4.190774	1.027112	-1.996335
H	-5.844771	-0.489089	-0.945048
S	1.813117	-0.968532	-1.800053
C	2.753120	-2.377489	-2.450354
H	3.786838	-2.056124	-2.547325
H	2.650200	-3.219600	-1.774416
H	2.325108	-2.606416	-3.423665
S	2.698479	0.682922	0.272929
C	1.202912	1.624456	0.108716
C	0.307762	1.669264	1.171578
C	0.925743	2.269137	-1.091522
C	-0.883308	2.360311	1.018680
H	0.539787	1.153369	2.089488
C	-0.275294	2.953358	-1.227361
H	1.637261	2.241938	-1.901775
C	-1.199066	3.002981	-0.182388
H	-1.583953	2.395625	1.841671
H	-0.497102	3.452886	-2.160111
C	-2.516145	3.708944	-0.342316
H	-2.564253	4.253219	-1.282991
H	-3.335993	2.988832	-0.325140
H	-2.686170	4.412047	0.473242
O	3.023877	0.538451	1.679106
O	3.684282	1.262497	-0.627818
N	2.373216	-0.881325	-0.234005
C	1.354312	-2.022428	0.799329
H	1.866583	-1.980884	1.747513
H	1.649188	-2.861412	0.180591
C	-0.748209	-1.285674	2.028306
O	0.178826	-0.913905	3.042725
H	-1.444752	-0.460690	1.846232
C	-0.473684	-0.585283	4.257245
H	-1.052619	-1.433073	4.636915
H	0.296454	-0.323741	4.978599
H	-1.148296	0.267036	4.124875
H	-1.345258	-2.125823	2.404967

1,3-Diene S-55

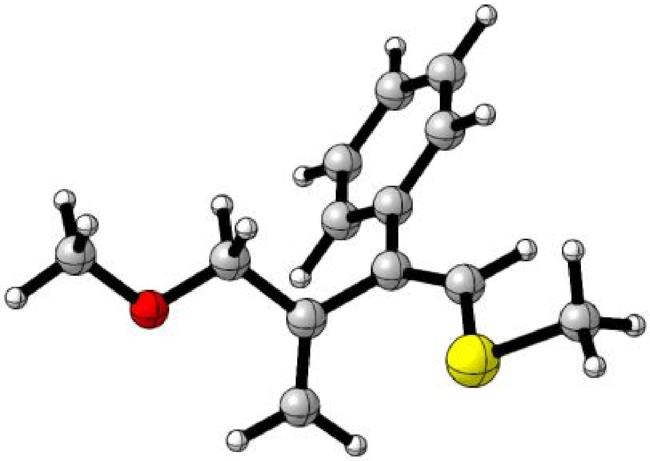

IEFPCM(MeCN)B3LYP-D3/6-311++G(2d,2p) Electronic Energy = –978.656845

IEFPCM(MeCN)B3LYP-D3/6-311++G(2d,2p) Free Energy = –978.446768

Number of imaginary frequencies = 0

C	0.207047	-0.420230	0.241412
C	1.093954	-1.414072	0.071842
H	0.753826	-2.420612	-0.128504
C	-1.248501	-0.666721	0.114621
C	-1.761879	-1.633559	-0.760933
C	-2.155366	0.078185	0.879951
C	-3.128495	-1.864079	-0.847314
H	-1.087737	-2.193148	-1.393587
C	-3.523459	-0.149198	0.789937
H	-1.780517	0.832527	1.557351
C	-4.017580	-1.124202	-0.071167
H	-3.501604	-2.612475	-1.532764
H	-4.203384	0.434719	1.394824
H	-5.081589	-1.299563	-0.144401
S	2.843293	-1.255794	0.180856
C	3.344203	-2.830265	-0.597447
H	4.429526	-2.859595	-0.551517
H	2.938415	-3.673320	-0.044776
H	3.024062	-2.864233	-1.634871
C	0.652115	0.964555	0.554830
C	1.290372	1.271508	1.680877
C	0.340621	1.974543	-0.514516
H	1.615946	2.281471	1.879723
H	1.492154	0.516241	2.426755
O	0.693068	3.282392	-0.111667
H	0.887261	1.699414	-1.427286
H	-0.726561	1.929250	-0.762653
C	0.426722	4.240877	-1.124602
H	-0.637442	4.259964	-1.379012
H	0.719897	5.211846	-0.734015
H	1.001974	4.027017	-2.030579

Sulfilimine A

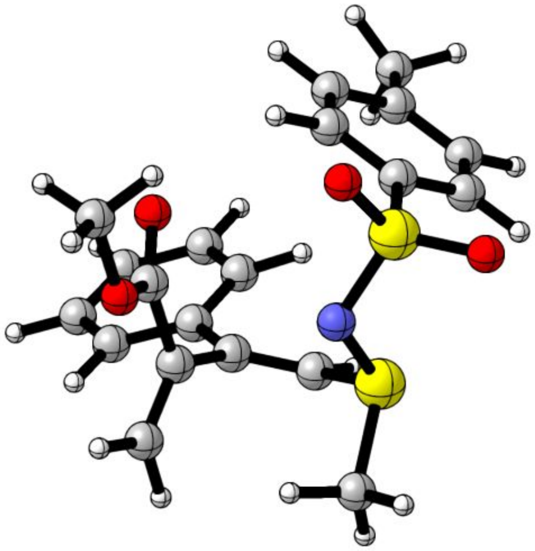

IEFPCM(MeCN)B3LYP-D3/6-311++G(2d,2p) Electronic Energy = –1927.233351

IEFPCM(MeCN)B3LYP-D3/6-311++G(2d,2p) Free Energy = –1926.921353

Number of imaginary frequencies = 0

C	-0.973042	-1.125217	-0.887847	H	1.712571	-2.733156	-2.233698
C	-0.364330	-2.281326	-0.174343	H	1.282819	-1.903019	-3.767958
C	-0.284351	-0.399286	-1.776874	S	2.773959	0.633791	0.219966
H	-0.746649	0.389340	-2.350630	C	1.444779	1.815142	0.383810
C	-2.384323	-0.798398	-0.594365	C	0.567494	1.719164	1.456221
C	-3.299326	-1.805456	-0.269650	C	1.242452	2.762567	-0.615404
C	-2.822474	0.532025	-0.618763	C	-0.524496	2.573224	1.519282
C	-4.626887	-1.492720	-0.006662	H	0.725606	0.975296	2.218968
H	-2.973056	-2.834018	-0.233005	C	0.140967	3.604313	-0.542476
C	-4.147550	0.842065	-0.349491	H	1.939139	2.839793	-1.435617
H	-2.113590	1.323687	-0.808388	C	-0.762864	3.518945	0.519630
C	-5.055815	-0.169481	-0.046985	H	-1.208315	2.496570	2.353470
H	-5.325147	-2.281905	0.233418	H	-0.016317	4.339947	-1.319567
H	-4.469466	1.873728	-0.359981	C	-1.977791	4.404592	0.567707
H	-6.086823	0.073591	0.167489	H	-1.762366	5.394286	0.167571
C	0.008013	-2.052030	1.250346	H	-2.785015	3.980209	-0.033253
O	0.888927	-2.946744	1.705023	H	-2.348862	4.513796	1.584982
O	-0.455084	-1.155529	1.921741	O	3.178491	0.197487	1.552380
C	1.332620	-2.753873	3.064604	O	3.790803	1.271022	-0.620416
H	0.489374	-2.804274	3.748458	N	2.127591	-0.697110	-0.474369
H	2.029381	-3.561800	3.255968	C	-0.253948	-3.476910	-0.748380
H	1.828129	-1.790430	3.152227	H	0.149461	-4.318884	-0.207014
S	1.491208	-0.371999	-1.965468	H	-0.583727	-3.639319	-1.764142
C	1.899290	-1.879692	-2.873852				
H	2.951450	-1.798105	-3.129547				

Transition State TS-A

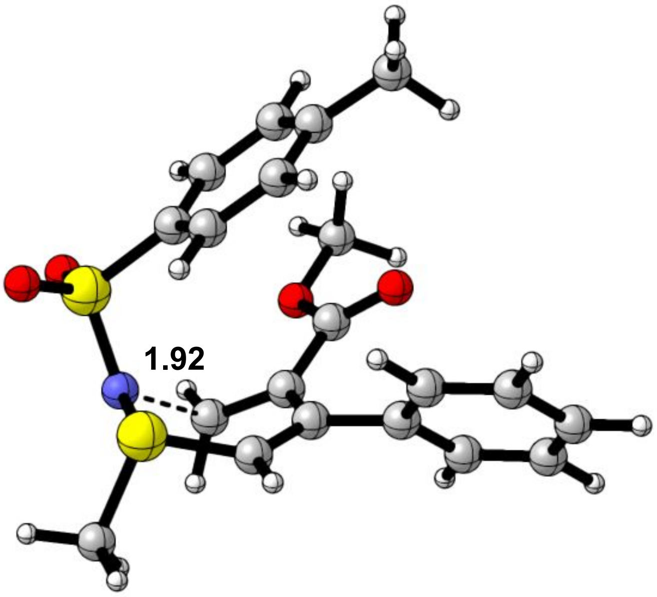

IEFPCM(MeCN)B3LYP-D3/6-311++G(2d,2p) Electronic Energy = –1927.215392

IEFPCM(MeCN)B3LYP-D3/6-311++G(2d,2p) Free Energy = –1926.899818

Number of imaginary frequencies = 1 (424.08i cm^{-1})

C	-0.900783	-1.374144	0.298046	H	1.772613	-4.053853	0.901866
C	-0.263502	-0.627162	1.344559	H	1.479082	-4.670979	-0.754520
C	-0.215277	-2.274890	-0.465062	S	2.946975	0.061219	-0.186863
H	-0.706048	-2.873509	-1.217822	C	1.676060	1.167213	-0.743887
C	-2.351802	-1.237701	0.008884	C	1.429794	2.338595	-0.041532
C	-3.286872	-1.353577	1.040101	C	0.942550	0.846976	-1.883315
C	-2.802578	-1.032614	-1.295666	C	0.426169	3.189887	-0.481598
C	-4.645534	-1.268517	0.771132	H	2.011726	2.573012	0.835103
H	-2.940119	-1.510392	2.051855	C	-0.071256	1.696444	-2.293718
C	-4.163963	-0.932894	-1.564712	H	1.160408	-0.046237	-2.448388
H	-2.082335	-0.924882	-2.094066	C	-0.350986	2.875475	-1.596363
C	-5.088877	-1.052729	-0.532838	H	0.234664	4.106410	0.058509
H	-5.360338	-1.367758	1.576189	H	-0.651310	1.443806	-3.170328
H	-4.500075	-0.758491	-2.577359	C	-1.485857	3.764439	-2.020290
H	-6.147154	-0.978406	-0.740572	H	-1.622911	3.745956	-3.100297
C	-0.730469	0.696961	1.724119	H	-2.418657	3.424133	-1.565278
O	0.045986	1.258850	2.696525	H	-1.322605	4.793672	-1.706166
O	-1.705608	1.282050	1.282229	O	3.588465	0.649610	0.974133
C	-0.358791	2.559093	3.143800	O	3.749913	-0.314381	-1.341647
H	-1.332226	2.511508	3.628033	N	2.206605	-1.310635	0.416319
H	0.399117	2.871314	3.854812	C	0.942660	-1.138582	1.851994
H	-0.407312	3.256476	2.311760	H	1.515328	-0.545248	2.544905
S	1.528073	-2.307556	-0.724772	H	1.002560	-2.204002	2.020392
C	2.023584	-3.951298	-0.148044				
H	3.092914	-4.031559	-0.320265				

Intermediate B

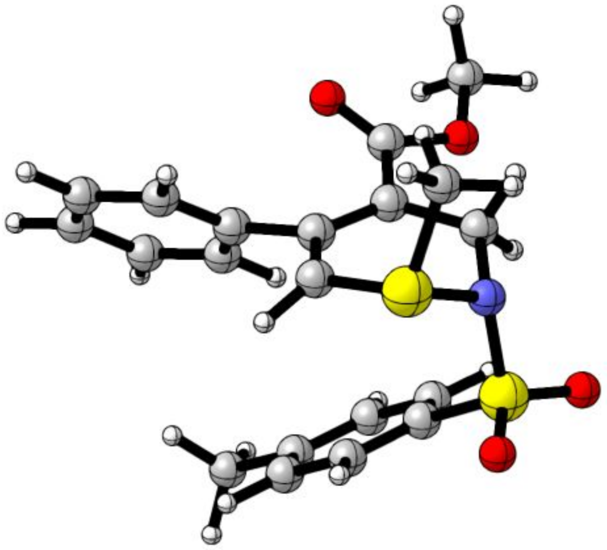

IEFPCM(MeCN)B3LYP-D3/6-311++G(2d,2p) Electronic Energy = –1927.231916

IEFPCM(MeCN)B3LYP-D3/6-311++G(2d,2p) Free Energy = –1926.915070

Number of imaginary frequencies = 0

C	-0.486045	-1.043013	-0.773003	H	1.416568	-3.264436	-1.213901
C	0.084361	-1.460641	0.417882	H	2.110958	-3.176739	-2.854738
C	0.263874	-0.742345	-1.928301	S	2.812942	0.909492	0.047624
H	-0.090399	-0.080825	-2.698929	C	1.310097	1.814708	0.273497
C	-1.940107	-0.751118	-0.900598	C	0.766012	1.933073	1.546835
C	-2.668232	-1.247702	-1.982612	C	0.684078	2.385077	-0.831690
C	-2.575085	0.096413	0.009394	C	-0.424289	2.628600	1.709264
C	-4.013357	-0.926121	-2.137374	H	1.268659	1.499725	2.397123
H	-2.180980	-1.898243	-2.695226	C	-0.503259	3.073248	-0.650477
C	-3.912700	0.428168	-0.149863	H	1.124735	2.298855	-1.811906
H	-2.010385	0.497897	0.836304	C	-1.078139	3.202518	0.617995
C	-4.639310	-0.086157	-1.222214	H	-0.851209	2.722058	2.697844
H	-4.569336	-1.329415	-2.972338	H	-0.994268	3.515773	-1.506064
H	-4.389738	1.089511	0.559997	C	-2.380197	3.932521	0.787617
H	-5.682362	0.170306	-1.344706	H	-2.302920	4.956876	0.421602
C	-0.718152	-2.036157	1.485049	H	-3.168029	3.443014	0.213595
O	-0.021871	-2.086703	2.659102	H	-2.686702	3.962635	1.830700
O	-1.852372	-2.484283	1.408214	O	3.513724	0.879537	1.315785
C	-0.692183	-2.715861	3.760903	O	3.476174	1.378790	-1.153037
H	-0.945898	-3.745379	3.518034	N	2.419115	-0.703029	-0.273831
H	0.011714	-2.684778	4.585969	C	1.577293	-1.399126	0.714238
H	-1.599061	-2.172887	4.017580	H	1.739356	-0.881890	1.656106
S	1.919725	-1.017756	-1.951799	H	1.998751	-2.394759	0.860373
C	2.184955	-2.817258	-1.833134				
H	3.181157	-2.985567	-1.434760				

Transition State TS-B

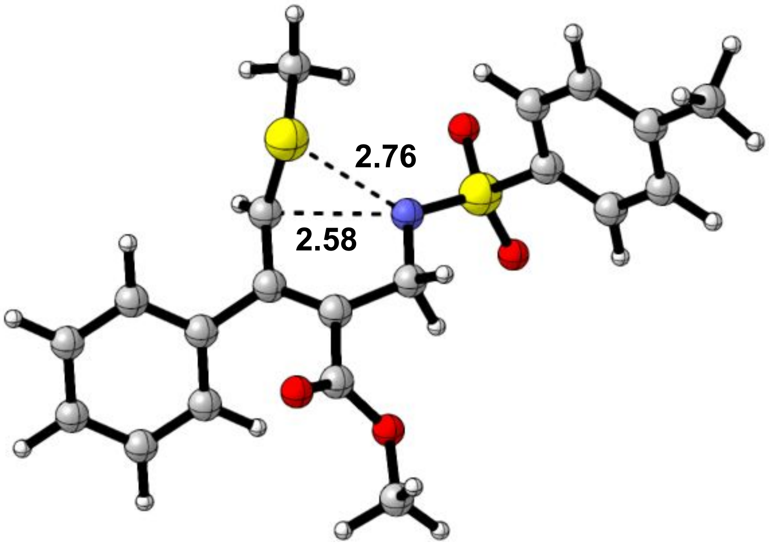

IEFPCM(MeCN)B3LYP-D3/6-311++G(2d,2p) Electronic Energy = –1927.214318

IEFPCM(MeCN)B3LYP-D3/6-311++G(2d,2p) Free Energy = –1926.905491

Number of imaginary frequencies = 1 (127.66i cm^{-1})

C	-2.091510	-0.423429	-0.134392	H	-0.669533	-4.419812	-0.073557
C	-1.242184	0.640089	-0.150690	H	0.844765	-3.514046	0.273248
C	-1.571925	-1.762801	-0.052053	S	1.959996	-0.743629	1.589037
H	-1.971421	-2.470752	0.662713	C	3.296007	-0.135559	0.560017
C	-3.569596	-0.298533	-0.100430	C	3.639076	-0.846488	-0.588635
C	-4.362490	-1.146938	-0.878950	C	3.944574	1.050497	0.868224
C	-4.190678	0.638320	0.732567	C	4.641195	-0.368328	-1.416145
C	-5.747944	-1.042462	-0.845922	H	3.121539	-1.763318	-0.829887
H	-3.893018	-1.873326	-1.527259	C	4.949898	1.522740	0.027384
C	-5.574277	0.741824	0.763701	H	3.666744	1.595370	1.757197
H	-3.589516	1.271384	1.369466	C	5.314269	0.824883	-1.122231
C	-6.356370	-0.096048	-0.027627	H	4.907180	-0.923983	-2.305639
H	-6.350045	-1.696760	-1.460058	H	5.454764	2.447481	0.272135
H	-6.042803	1.468658	1.411859	C	6.402301	1.329332	-2.031298
H	-7.433734	-0.015343	-0.000766	H	6.037746	1.440302	-3.053303
C	-1.755801	1.997237	-0.529253	H	7.238704	0.629181	-2.062811
O	-1.400678	2.938605	0.349247	H	6.781189	2.293376	-1.698171
O	-2.376126	2.215075	-1.545644	O	2.238581	-2.160840	1.858341
C	-1.800977	4.290361	0.028656	O	1.922185	0.138116	2.769232
H	-1.355506	4.595398	-0.914294	N	0.659183	-0.694645	0.679931
H	-1.431217	4.899287	0.844901	C	0.172710	0.609855	0.343236
H	-2.884038	4.349406	-0.037172	H	0.157957	1.286172	1.210645
S	-0.529770	-2.302670	-1.219553	H	0.813170	1.124792	-0.390137
C	0.136086	-3.824896	-0.492603				
H	0.633300	-4.365757	-1.291119				

Intermediate C

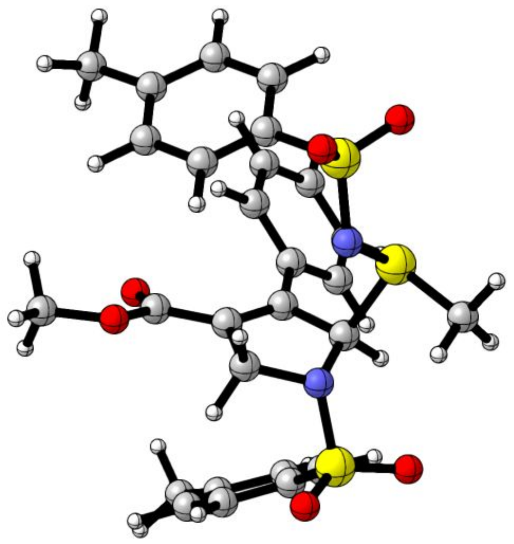

IEFPCM(MeCN)B3LYP-D3/6-311++G(2d,2p) Electronic Energy = –2801.816486

IEFPCM(MeCN)B3LYP-D3/6-311++G(2d,2p) Free Energy = –2801.379337

Number of imaginary frequencies = 0

C	-0.363337	0.095930	-0.437745	H	-4.693236	2.355196	1.767985
C	-0.427090	0.409738	0.867365	H	-4.546516	1.372376	-2.395877
N	-1.324246	-1.752602	0.680730	O	-3.320058	-2.447226	2.022259
C	0.079830	0.866015	-1.602813	O	-3.188741	-2.984778	-0.440281
C	1.194330	1.705814	-1.526643	C	-5.084328	3.434765	-0.700322
C	-0.582308	0.729612	-2.829680	H	-4.213276	4.083160	-0.816726
C	1.631111	2.399170	-2.644307	H	-5.637671	3.457103	-1.637284
H	1.725824	1.799309	-0.596032	H	-5.707088	3.859750	0.084713
C	-0.154070	1.438224	-3.944330	C	-0.870774	-0.769465	1.696962
H	-1.447298	0.089025	-2.914958	H	-0.052725	-1.197055	2.271239
C	0.955332	2.273514	-3.855482	H	-1.671948	-0.529752	2.388094
H	2.504123	3.031733	-2.571125	C	-0.778657	-1.342489	-0.596614
H	-0.683359	1.334096	-4.880555	H	-1.450284	-1.541158	-1.425321
H	1.295272	2.816774	-4.725769	C	-0.062306	-3.989591	-1.079828
C	-0.197928	1.727449	1.491189	H	-0.796369	-3.991657	-1.880849
O	-0.029986	1.588341	2.816741	H	0.727721	-4.707559	-1.275079
O	-0.198377	2.796469	0.922825	H	-0.526324	-4.159853	-0.113703
C	0.136782	2.810325	3.565793	S	0.735476	-2.370847	-1.045915
H	0.985318	3.372187	3.185147	N	1.558618	-2.394221	0.372101
H	-0.763589	3.414846	3.492877	S	3.166248	-2.175086	0.228236
H	0.309014	2.499337	4.589310	C	3.480186	-0.423106	0.366242
S	-2.987539	-2.070304	0.665652	O	3.736568	-2.789327	1.423537
C	-3.748024	-0.520642	0.280093	O	3.685662	-2.601300	-1.072587
C	-4.027035	0.381903	1.302758	C	3.024580	0.261971	1.490150
C	-3.943608	-0.173485	-1.054171	C	4.211967	0.232551	-0.611856
C	-4.474624	1.652734	0.976045	C	3.305079	1.611287	1.622381
H	-3.907139	0.092842	2.335055	H	2.461222	-0.253844	2.252838
C	-4.392171	1.101683	-1.360871	C	4.490661	1.587558	-0.463209
H	-3.765963	-0.894453	-1.837012	H	4.557724	-0.310556	-1.477369
C	-4.648209	2.038626	-0.356008	C	4.037879	2.298543	0.646558

H	2.954197	2.142430	2.495790	H	4.538621	4.039660	1.809429
H	5.058561	2.097870	-1.228780	H	5.100778	4.099825	0.133643
C	4.290052	3.774555	0.782424	H	3.396378	4.339362	0.508508

Intermediate D

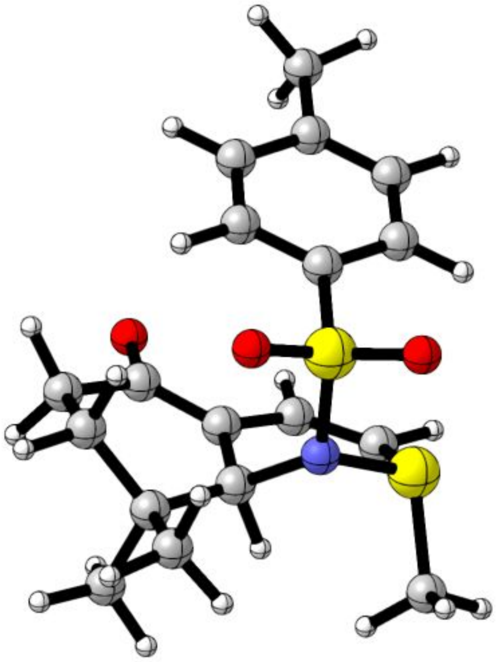

IEFPCM(MeCN)B3LYP-D3/6-311++G(2d,2p) Electronic Energy = –1776.941046

IEFPCM(MeCN)B3LYP-D3/6-311++G(2d,2p) Free Energy = –1776.612892

Number of imaginary frequencies = 0

C	0.181370	0.124873	-2.091905	O	-0.012793	-2.643795	1.697956
C	-0.018238	-1.221588	-2.281825	N	-1.218115	-1.066737	0.155890
H	0.591192	-1.826244	-2.933489	H	0.964811	0.585668	-2.680408
S	-0.821802	-2.187953	-1.071753	C	-0.486649	0.929665	-1.166742
C	-2.473752	-2.655846	-1.661899	C	-0.064977	2.266617	-0.916472
H	-2.953307	-3.195946	-0.850108	O	0.868101	2.825424	-1.532260
H	-3.037543	-1.780999	-1.965270	C	-0.804618	3.071382	0.143369
H	-2.297160	-3.312612	-2.510665	C	-2.540332	1.215447	0.367449
S	-0.101536	-1.215981	1.468873	H	-0.055183	3.635283	0.699477
C	1.475238	-0.625871	0.939277	H	-1.400244	3.815726	-0.391327
C	1.841304	0.686980	1.214392	C	-1.686010	2.262962	1.096480
C	2.320545	-1.479606	0.239044	C	-3.471100	1.923206	-0.642850
C	3.066327	1.150649	0.766609	C	-3.425768	0.435946	1.344339
H	1.180869	1.330065	1.772382	H	-1.070637	1.763737	1.838960
C	3.541259	-0.995487	-0.207144	H	-2.347727	2.936927	1.643690
H	2.036426	-2.503747	0.055372	H	-4.053304	2.690797	-0.133318
C	3.928266	0.323851	0.039446	H	-4.166210	1.208365	-1.084082
H	3.355507	2.170122	0.978896	H	-2.919593	2.397008	-1.452684
H	4.203837	-1.653536	-0.751481	H	-4.151767	1.115338	1.792260
C	5.235377	0.853433	-0.479761	H	-2.848077	-0.020948	2.140622
H	5.934600	0.047100	-0.691715	H	-3.978235	-0.348895	0.825917
H	5.076553	1.408679	-1.406624	C	-1.644382	0.288728	-0.482715
H	5.695012	1.536623	0.232962	H	-2.311788	-0.102155	-1.249571
O	-0.604180	-0.343917	2.504369				

Transition State TS-D

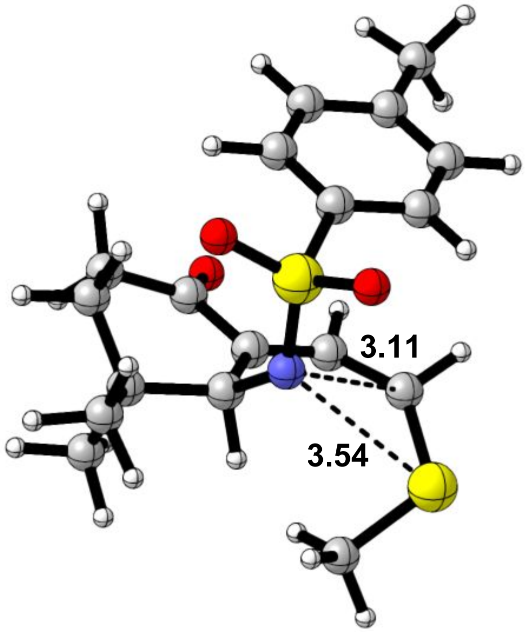

IEFPCM(MeCN)B3LYP-D3/6-311++G(2d,2p) Electronic Energy = –-1776.923873

IEFPCM(MeCN)B3LYP-D3/6-311++G(2d,2p) Free Energy = –-1776.596898

Number of imaginary frequencies = 1 (54.08 cm^{-1})

C	0.207772	1.247167	1.329047	O	0.132897	0.442559	-3.317331
C	0.223008	2.482215	0.626361	N	-1.145743	0.403592	-1.241000
H	1.211737	2.858489	0.383257	H	1.060283	1.133131	1.991083
S	-0.898209	3.616897	0.215260	C	-0.552927	0.143346	1.154186
C	-2.515760	3.091782	0.819149	C	-0.167005	-1.020306	2.054168
H	-3.035314	2.571513	0.018938	O	0.563540	-0.829690	3.008613
H	-2.403245	2.470828	1.701307	C	-0.727141	-2.390939	1.772567
H	-3.039630	4.012897	1.059431	C	-2.459371	-1.310127	0.256845
S	-0.093794	-0.367497	-2.106154	H	0.115522	-3.082977	1.786831
C	1.479307	-0.376283	-1.231929	H	-1.326952	-2.644685	2.649493
C	1.870492	-1.479460	-0.487354	C	-1.546670	-2.519806	0.491812
C	2.248451	0.783519	-1.204060	C	-3.385494	-1.098124	1.470814
C	3.006138	-1.409487	0.315919	C	-3.332365	-1.544278	-0.977613
H	1.293113	-2.389085	-0.534675	H	-0.897097	-2.635267	-0.370457
C	3.379372	0.844372	-0.404112	H	-2.154435	-3.423866	0.551437
H	1.954812	1.632021	-1.804482	H	-3.917093	-2.019544	1.709613
C	3.770531	-0.247516	0.382161	H	-4.124760	-0.326998	1.252018
H	3.293219	-2.270930	0.903592	H	-2.850882	-0.785443	2.369022
H	3.969144	1.751579	-0.383952	H	-4.011410	-2.378192	-0.791959
C	4.990100	-0.159835	1.258961	H	-2.724030	-1.772609	-1.847212
H	4.914041	0.680645	1.950028	H	-3.933727	-0.662008	-1.200505
H	5.123332	-1.068683	1.842185	C	-1.624968	0.000180	0.082296
H	5.890250	-0.005034	0.661946	H	-2.351887	0.774740	0.311194
O	-0.364381	-1.794691	-2.381550				

Intermediate E

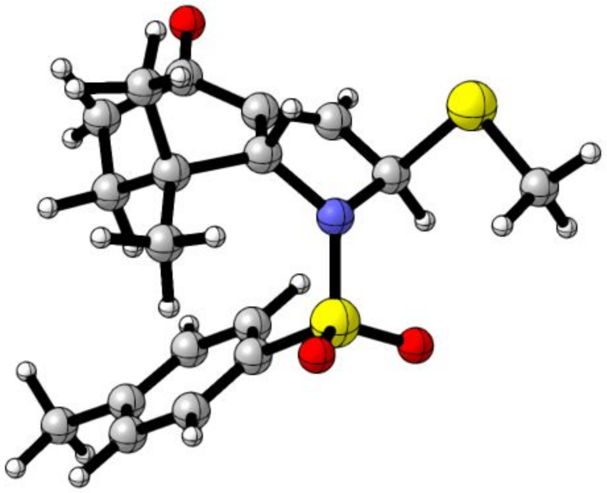

IEFPCM(MeCN)B3LYP-D3/6-311++G(2d,2p) Electronic Energy = –1776.984951

IEFPCM(MeCN)B3LYP-D3/6-311++G(2d,2p) Free Energy = –1776.657642

Number of imaginary frequencies = 0

C	1.499163	0.885961	-1.676922	H	3.642793	-2.140927	0.633590
C	0.941454	1.638481	-0.731359	H	5.252078	-2.007028	-0.105096
N	1.312818	-0.471722	0.226891	H	3.908358	-2.703176	-1.037443
S	0.386484	-1.827027	0.497109	H	1.551021	1.108116	-2.730204
C	-1.279625	-1.465927	-0.001532	C	0.111386	2.831724	-0.997793
C	-1.513740	-1.008791	-1.296885	O	0.331718	3.579888	-1.936086
C	-2.326407	-1.635703	0.892403	C	-1.080067	3.021869	-0.081709
C	-2.805205	-0.693332	-1.679799	H	-1.959173	3.103137	-0.722320
H	-0.701483	-0.888165	-1.996466	H	-0.965444	3.997327	0.393869
C	-3.619349	-1.320074	0.490663	C	-1.260557	1.911112	0.956700
H	-2.133332	-2.000798	1.888542	C	0.053355	1.525028	1.666567
C	-3.878317	-0.836049	-0.790948	H	-1.689647	1.032850	0.481837
H	-2.986103	-0.326791	-2.680792	H	-1.979168	2.239821	1.707302
H	-4.434884	-1.445352	1.188961	C	0.728447	2.801212	2.223013
O	0.920709	-2.831665	-0.410331	C	-0.238719	0.623952	2.866735
O	0.380585	-2.118654	1.914646	C	1.077705	0.971852	0.632442
C	-5.272661	-0.473594	-1.220070	H	0.010799	3.371808	2.811655
H	-5.327622	0.577529	-1.506275	H	1.562865	2.529758	2.868757
H	-5.990770	-0.648242	-0.422110	H	1.117237	3.456030	1.444062
H	-5.575596	-1.059781	-2.088525	H	-0.731466	1.220874	3.634613
C	2.001238	-0.390646	-1.082460	H	-0.893330	-0.201137	2.618185
H	1.797173	-1.270517	-1.677564	H	0.676478	0.213915	3.289309
S	3.839428	-0.278384	-0.897933	H	2.059003	1.245281	1.022789
C	4.182198	-1.962484	-0.291975				

Intermediate F

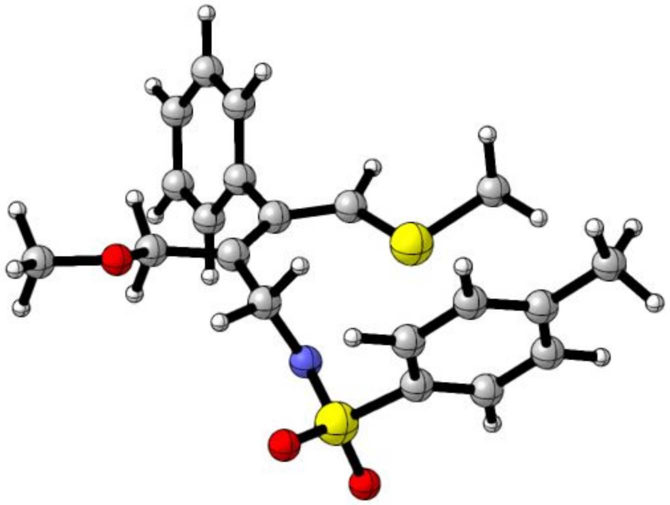

IEFPCM(MeCN)B3LYP-D3/6-311++G(2d,2p) Electronic Energy = –1853.155540

IEFPCM(MeCN)B3LYP-D3/6-311++G(2d,2p) Free Energy = –1852.826462

Number of imaginary frequencies = 0

C	-1.870032	-0.571123	-0.009211	H	3.394372	-0.325038	2.203686
C	-1.550190	0.722724	-0.366128	C	4.061241	-0.217426	-1.590900
C	-0.940103	-1.622688	0.122733	H	2.854473	1.561003	-1.593781
H	-1.365101	-2.613435	0.217324	C	4.618384	-1.249461	-0.837240
C	-3.300409	-0.979185	0.157650	H	4.808810	-2.049655	1.148594
C	-3.904493	-1.856552	-0.744616	H	4.241614	-0.179606	-2.656820
C	-4.038359	-0.494862	1.238395	C	5.461236	-2.319544	-1.477218
C	-5.231639	-2.233634	-0.574813	H	5.057818	-3.311202	-1.267509
H	-3.339231	-2.233457	-1.585917	H	6.480866	-2.298740	-1.089263
C	-5.365279	-0.874547	1.408821	H	5.508150	-2.193220	-2.556992
H	-3.571380	0.172360	1.949419	O	2.150466	1.891496	2.536756
C	-5.964750	-1.742457	0.501665	O	1.992813	3.109630	0.363984
H	-5.692374	-2.907474	-1.283085	N	0.409037	1.181861	0.941678
H	-5.927116	-0.495946	2.250808	C	-0.152587	1.253860	-0.400300
H	-6.996242	-2.036647	0.633658	H	-0.157001	2.260714	-0.808870
S	0.727724	-1.571328	0.110358	H	0.402001	0.631172	-1.133869
C	1.140599	-3.349629	0.056481	C	-2.654796	1.675961	-0.704015
H	2.212786	-3.398501	0.219867	O	-2.213621	2.655010	-1.615529
H	0.892670	-3.751925	-0.920249	H	-3.524866	1.143415	-1.097463
H	0.616637	-3.875782	0.847763	C	-3.213978	3.636139	-1.868402
S	1.832865	1.840923	1.102867	H	-3.485591	4.160767	-0.948549
C	3.029960	0.721871	0.370190	H	-2.791254	4.342051	-2.577247
C	3.588949	-0.294863	1.142360	H	-4.109930	3.180488	-2.298441
C	3.274650	0.765914	-0.996685	H	-2.975313	2.146365	0.240021
C	4.377470	-1.264387	0.541549				

Transition State TS-F

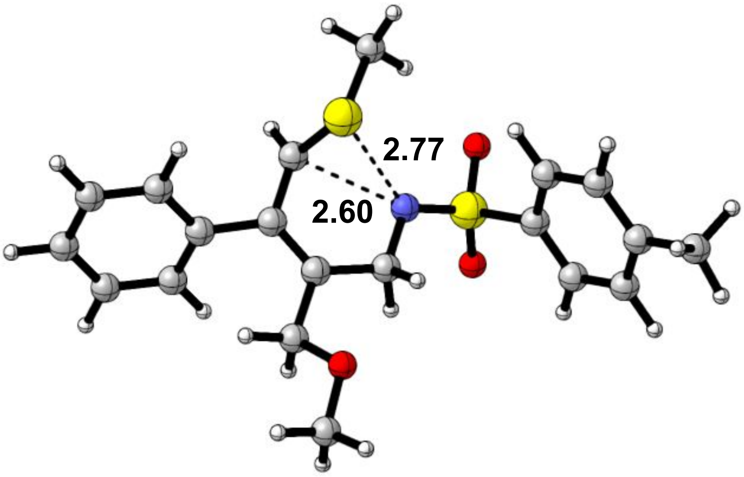

IEFPCM(MeCN)B3LYP-D3/6-311++G(2d,2p) Electronic Energy = –1853.146806

IEFPCM(MeCN)B3LYP-D3/6-311++G(2d,2p) Free Energy = –1852.818310

Number of imaginary frequencies = 1 (115.97 cm^{-1})

C	-2.209551	-0.232929	-0.104943	H	2.833003	-1.679667	-0.976958
C	-1.345534	0.809251	0.061292	C	4.928207	1.330286	0.213041
C	-1.735266	-1.577538	-0.256036	H	3.697908	1.279776	1.981586
H	-2.193570	-2.392089	0.291249	C	5.209578	0.757250	-1.025222
C	-3.692115	-0.095117	-0.076802	H	4.640386	-0.796640	-2.398638
C	-4.465501	-0.697985	-1.074013	H	5.507743	2.178603	0.551596
C	-4.336702	0.592864	0.956001	C	6.306889	1.293741	-1.904639
C	-5.851667	-0.595138	-1.052195	H	5.915405	1.597707	-2.876510
H	-3.978436	-1.233971	-1.876559	H	7.066946	0.532777	-2.088362
C	-5.721650	0.693728	0.978191	H	6.792930	2.154696	-1.450135
H	-3.753378	1.033897	1.751108	O	2.035274	-2.344665	1.724206
C	-6.482421	0.102642	-0.027294	O	1.849932	-0.130694	2.850399
H	-6.436615	-1.058522	-1.833919	N	0.506546	-0.704857	0.722798
H	-6.207089	1.227471	1.782882	C	0.076536	0.650865	0.508105
H	-7.560103	0.181813	-0.008422	H	0.134336	1.255964	1.426162
S	-0.662375	-1.945581	-1.466498	H	0.718045	1.185494	-0.204963
C	-0.073033	-3.593756	-0.989903	C	-1.830533	2.235240	0.032529
H	0.442575	-4.008335	-1.849983	O	-0.841328	3.040179	-0.593179
H	-0.913640	-4.222158	-0.711555	H	-2.778157	2.314043	-0.503845
H	0.610641	-3.448789	-0.155634	C	-1.209767	4.413008	-0.601772
S	1.811599	-0.896764	1.588979	H	-1.328295	4.792404	0.417356
C	3.165139	-0.257038	0.596368	H	-0.409502	4.954658	-1.098473
C	3.425427	-0.840899	-0.642085	H	-2.145039	4.565553	-1.148190
C	3.911107	0.830533	1.023912	H	-1.996952	2.593192	1.057109
C	4.440259	-0.338164	-1.439134				

Intermediate G

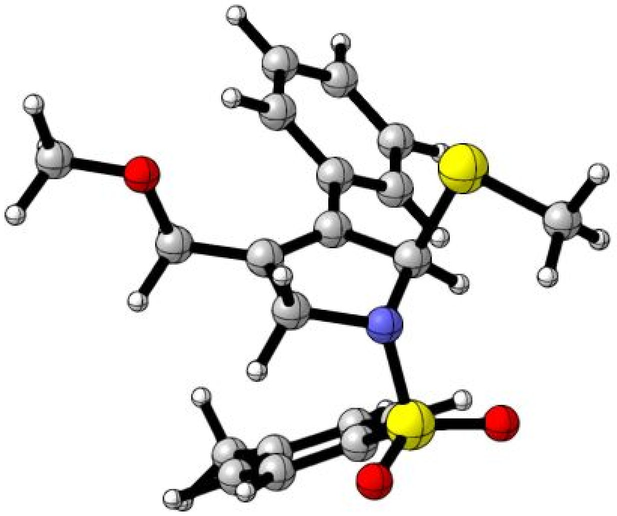

IEFPCM(MeCN)B3LYP-D3/6-311++G(2d,2p) Electronic Energy = –1853.229392

IEFPCM(MeCN)B3LYP-D3/6-311++G(2d,2p) Free Energy = –1852.895843

Number of imaginary frequencies = 0

C	0.779257	-0.687314	0.117150
C	0.547818	-0.362641	1.395788
N	-1.405772	-1.434028	0.629258
S	-2.747914	-0.542483	0.155873
C	-2.165522	1.100241	-0.168895
C	-1.703127	1.420135	-1.442036
C	-2.066824	2.014704	0.875525
C	-1.112721	2.655839	-1.657739
H	-1.815605	0.717570	-2.252956
C	-1.472843	3.246012	0.641965
H	-2.455910	1.771600	1.851851
C	-0.973738	3.580830	-0.619649
H	-0.754367	2.905010	-2.646820
H	-1.397190	3.958259	1.451758
O	-3.192356	-1.130611	-1.093752
O	-3.628883	-0.508328	1.307955
C	-0.287964	4.898341	-0.851312
H	0.787233	4.799330	-0.687135
H	-0.655613	5.661801	-0.168137
H	-0.432114	5.245124	-1.873088
C	-0.744562	-0.967764	1.874488
H	-1.378364	-0.269844	2.414489
H	-0.575319	-1.822625	2.531760
C	-0.349581	-1.552006	-0.394884
H	-0.731792	-1.268131	-1.368796
S	0.242015	-3.291778	-0.554831
C	-1.238541	-4.027418	-1.321042
H	-2.104841	-3.861236	-0.687404
H	-1.046562	-5.093586	-1.407768
H	-1.407825	-3.605409	-2.308147
C	1.929939	-0.340790	-0.724699
C	1.751868	-0.052294	-2.083878
C	3.225851	-0.291412	-0.194052
C	2.831114	0.301678	-2.881963
H	0.763842	-0.088157	-2.517995
C	4.305212	0.059817	-0.995565
H	3.384971	-0.553934	0.841399
C	4.112343	0.361680	-2.340184
H	2.672434	0.529830	-3.926526
H	5.298940	0.088513	-0.571017
H	4.953242	0.632454	-2.962914
C	1.388186	0.485554	2.287470
O	2.242870	-0.351125	3.069110
C	3.058896	0.409150	3.950735
H	3.676403	-0.293926	4.503246
H	3.702185	1.096637	3.393124
H	2.448956	0.985925	4.652284
H	0.750121	1.076590	2.953341
H	1.988548	1.181036	1.694144

Intermediate S-H

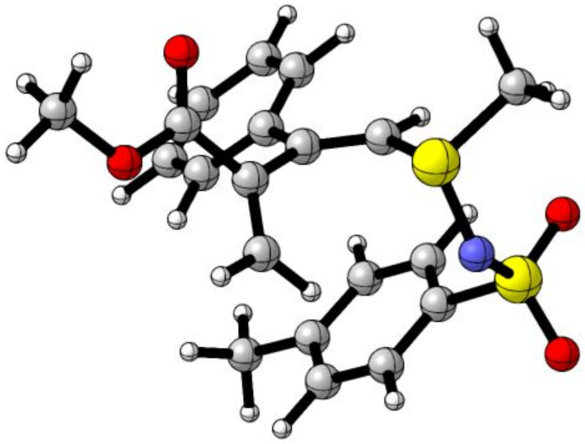

IEFPCM(MeCN)B3LYP-D3/6-311++G(2d,2p) Electronic Energy = –1927.241260

IEFPCM(MeCN)B3LYP-D3/6-311++G(2d,2p) Free Energy = –1926.928270

Number of imaginary frequencies = 0

C	-0.863548	-0.614279	0.492910	H	1.185454	-3.246136	2.679961
C	0.287521	-0.976072	1.074316	C	-0.751874	-1.447737	-1.837349
H	0.719208	-0.421211	1.891249	H	-1.186506	-1.969423	-2.675779
C	-1.417206	-1.323044	-0.688996	H	0.232852	-1.025107	-1.959055
C	-2.774516	-1.913501	-0.495146	S	1.274977	-2.333642	0.497057
O	-3.278609	-2.067118	0.597596	N	2.478100	-1.775933	-0.479219
O	-3.361260	-2.269052	-1.642033	S	3.289890	-0.417364	-0.083058
C	-4.652375	-2.903357	-1.522440	O	3.545884	-0.287061	1.353958
H	-4.960660	-3.119305	-2.538668	O	4.441645	-0.393197	-0.980007
H	-4.567089	-3.819407	-0.943766	C	2.235037	0.951483	-0.538066
H	-5.356337	-2.228567	-1.042641	C	1.783363	1.837303	0.428400
C	-1.601279	0.569061	0.982163	C	1.840339	1.091744	-1.865905
C	-1.549896	0.960161	2.324545	C	0.914307	2.859788	0.066414
C	-2.320487	1.355685	0.076908	H	2.093136	1.716467	1.454677
C	-2.184474	2.119984	2.744528	C	0.973050	2.114812	-2.212895
H	-1.028914	0.346173	3.044549	H	2.201091	0.403208	-2.615737
C	-2.953045	2.517577	0.498204	C	0.490297	3.010801	-1.252277
H	-2.355821	1.076117	-0.965595	H	0.545701	3.534823	0.825963
C	-2.884166	2.905875	1.831633	H	0.656652	2.215421	-3.242349
H	-2.140031	2.407901	3.785258	C	-0.468879	4.100775	-1.642333
H	-3.490741	3.123461	-0.216798	H	-0.805503	4.660361	-0.772711
H	-3.376344	3.810219	2.160142	H	-0.002525	4.798385	-2.339771
C	2.000562	-2.842930	2.084301	H	-1.346272	3.685018	-2.138618
H	2.722795	-3.619273	1.850587				
H	2.477632	-1.994854	2.560046				

Transition State TS-S-H

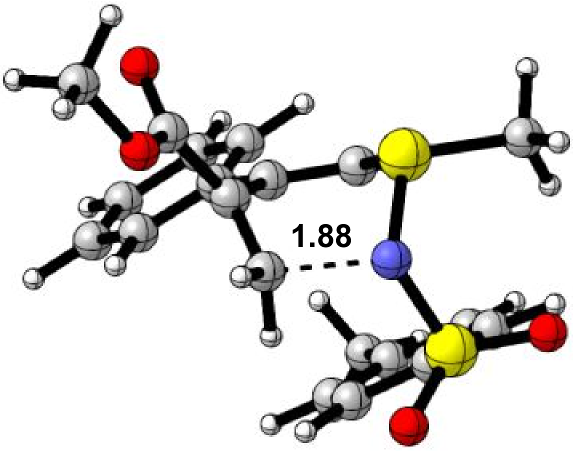

IEFPCM(MeCN)B3LYP-D3/6-311++G(2d,2p) Electronic Energy = –1927.197122

IEFPCM(MeCN)B3LYP-D3/6-311++G(2d,2p) Free Energy = –1926.882718

Number of imaginary frequencies = 1 (476.76i cm^{-1})

C	1.061289	0.281377	0.497845	H	-1.817858	1.066635	3.586843
C	0.020904	0.108257	1.345565	C	-0.125233	1.771889	-1.116424
H	-0.252657	-0.772079	1.901402	H	-0.184042	2.751862	-1.563489
C	1.011036	1.524628	-0.294259	H	-0.456274	0.988337	-1.773938
C	2.019433	2.515554	-0.061364	S	-1.000513	1.540112	1.455443
O	2.986712	2.382988	0.686542	N	-1.707431	1.753097	-0.098657
O	1.828327	3.672746	-0.766379	S	-2.876013	0.685937	-0.679557
C	2.781391	4.715783	-0.525501	O	-4.031690	0.722159	0.202519
H	2.461145	5.550263	-1.141298	O	-3.018030	1.082518	-2.070577
H	2.785052	5.003937	0.523910	C	-2.206009	-0.960119	-0.626076
H	3.781866	4.399846	-0.813805	C	-2.510812	-1.791370	0.444527
C	2.105777	-0.740153	0.329495	C	-1.294006	-1.367951	-1.597617
C	2.372999	-1.689150	1.324587	C	-1.839041	-2.998229	0.584403
C	2.808078	-0.813909	-0.877543	H	-3.264119	-1.501806	1.159575
C	3.305127	-2.693649	1.109295	C	-0.626990	-2.570443	-1.441243
H	1.860537	-1.630577	2.274132	H	-1.108339	-0.758378	-2.467149
C	3.741174	-1.819726	-1.093050	C	-0.862474	-3.389685	-0.332904
H	2.599128	-0.086035	-1.648095	H	-2.070811	-3.637843	1.424457
C	3.989845	-2.765108	-0.102382	H	0.096765	-2.873470	-2.184682
H	3.503515	-3.417952	1.886691	C	-0.066839	-4.648033	-0.134404
H	4.270593	-1.869101	-2.034169	H	0.932773	-4.401142	0.229165
H	4.714647	-3.549318	-0.269646	H	-0.536660	-5.306105	0.593412
C	-2.305974	1.077330	2.615836	H	0.053183	-5.191104	-1.071096
H	-3.053127	1.861869	2.573728				
H	-2.739483	0.116650	2.380920				

Intermediate S-I

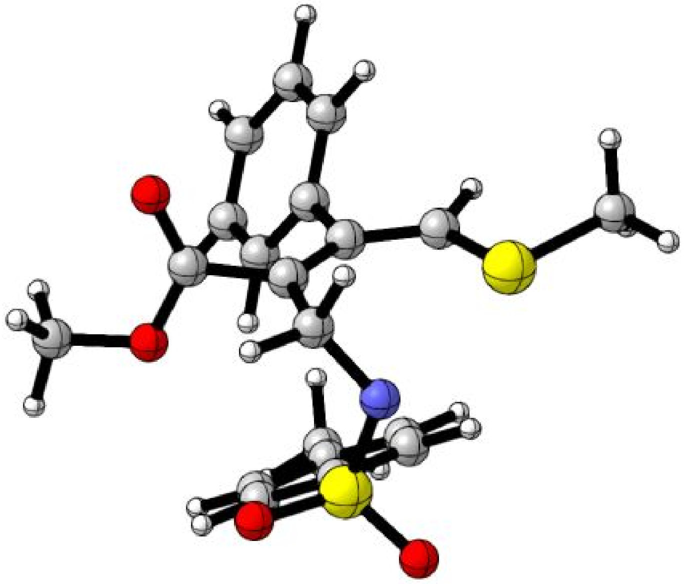

IEFPCM(MeCN)B3LYP-D3/6-311++G(2d,2p) Electronic Energy = –1927.223392

IEFPCM(MeCN)B3LYP-D3/6-311++G(2d,2p) Free Energy = –1926.910351

Number of imaginary frequencies = 0

C	-0.456070	1.423127	0.357488	H	0.836650	5.096442	-1.479971
C	0.076888	2.391987	-0.527673	C	1.711315	0.958148	1.679310
H	-0.644095	2.856829	-1.186587	H	1.896191	2.003562	1.959592
C	0.232092	0.837909	1.389429	H	1.946606	0.359537	2.561893
C	-0.542012	0.062317	2.411275	S	1.634940	2.942858	-0.717796
O	-1.225563	0.618614	3.241301	N	2.555578	0.651787	0.548059
O	-0.319969	-1.239657	2.338149	S	2.776862	-0.869519	0.182092
C	-0.993837	-2.069128	3.315370	O	3.905451	-0.919538	-0.760971
H	-0.695265	-3.082856	3.078009	O	2.878895	-1.763749	1.349090
H	-0.673727	-1.794531	4.316273	C	1.369270	-1.467493	-0.765675
H	-2.069338	-1.949433	3.218695	C	0.971380	-0.759118	-1.897605
C	-1.900486	1.104317	0.149112	C	0.695816	-2.622255	-0.397604
C	-2.861636	2.116814	0.114692	C	-0.099009	-1.210210	-2.652390
C	-2.298533	-0.221839	-0.022553	H	1.496372	0.140886	-2.181361
C	-4.201627	1.804246	-0.078454	C	-0.374425	-3.071817	-1.167582
H	-2.567325	3.147406	0.259288	H	1.003059	-3.158424	0.486153
C	-3.638974	-0.532606	-0.216788	C	-0.792000	-2.374265	-2.299474
H	-1.559957	-1.006656	-0.021486	H	-0.407891	-0.650842	-3.525442
C	-4.593748	0.478460	-0.244296	H	-0.897088	-3.972313	-0.873996
H	-4.938941	2.594192	-0.092926	C	-1.981918	-2.826698	-3.100585
H	-3.932842	-1.563356	-0.355429	H	-2.842970	-2.185168	-2.901330
H	-5.636214	0.237337	-0.396346	H	-1.781275	-2.775183	-4.170603
C	1.369497	4.273897	-1.946193	H	-2.262949	-3.848085	-2.851203
H	2.362610	4.593019	-2.246933				
H	0.828101	3.881580	-2.800819				

Transition State TS-S-I

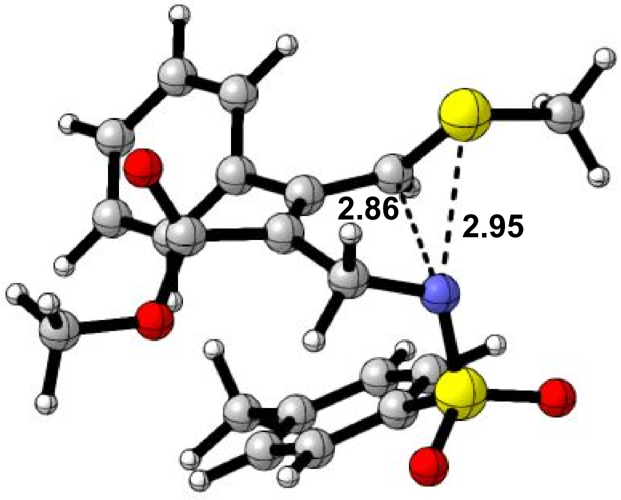

IEFPCM(MeCN)B3LYP-D3/6-311++G(2d,2p) Electronic Energy = –1927.218206

IEFPCM(MeCN)B3LYP-D3/6-311++G(2d,2p) Free Energy = –1926.906931

Number of imaginary frequencies = 1 (144.87i cm^{-1})

C	0.501944	1.078042	-0.453572	H	-1.386239	3.801451	2.114418
C	-0.323463	1.904063	0.384318	C	-1.389180	0.166987	-1.950053
H	-0.177348	1.849605	1.455596	H	-1.554451	0.756476	-2.862416
C	0.049776	0.417056	-1.547632	H	-1.429099	-0.872908	-2.292216
C	1.055284	-0.192234	-2.485223	S	-1.289990	3.106325	-0.193010
O	1.798287	0.470098	-3.172068	N	-2.402983	0.487721	-0.980189
O	0.978181	-1.521068	-2.507562	S	-2.991288	-0.735226	-0.183675
C	1.900297	-2.200273	-3.392739	O	-4.065916	-0.235778	0.685682
H	1.698612	-3.255982	-3.258284	O	-3.335821	-1.907932	-1.014021
H	1.717086	-1.898149	-4.419925	C	-1.682816	-1.315934	0.904728
H	2.922534	-1.962501	-3.110778	C	-1.428204	-0.636152	2.093073
C	1.907640	0.973519	0.030682	C	-0.848129	-2.350686	0.506560
C	2.598538	2.127181	0.413646	C	-0.328297	-0.981170	2.863604
C	2.539006	-0.268004	0.146074	H	-2.090395	0.156819	2.408795
C	3.902733	2.042062	0.886748	C	0.250827	-2.692462	1.290330
H	2.123618	3.093941	0.322122	H	-1.051933	-2.881739	-0.410294
C	3.842523	-0.350262	0.615693	C	0.538066	-2.007504	2.469082
H	2.000382	-1.167302	-0.105575	H	-0.135954	-0.447886	3.785283
C	4.528058	0.803505	0.986746	H	0.900158	-3.496345	0.970030
H	4.429029	2.941415	1.172837	C	1.762703	-2.331122	3.279667
H	4.318320	-1.316551	0.703609	H	2.550598	-1.600476	3.084347
H	5.541415	0.736429	1.356219	H	1.550450	-2.304375	4.348023
C	-2.103268	3.743309	1.302333	H	2.155271	-3.314980	3.029407
H	-2.476081	4.730568	1.049915				
H	-2.926717	3.078790	1.546529				

Intermediate S-J

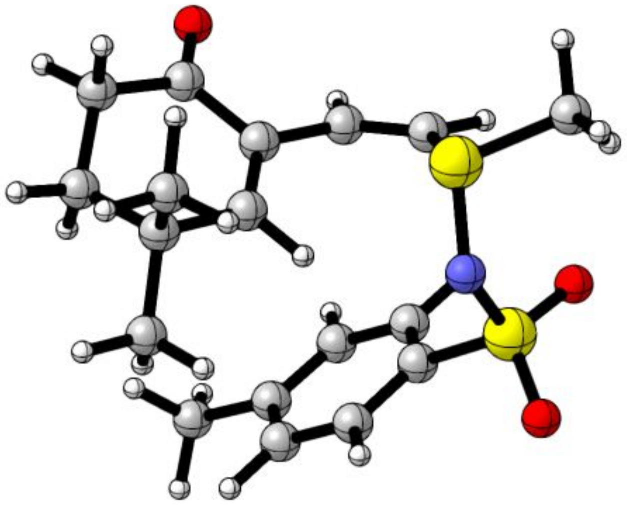

IEFPCM(MeCN)B3LYP-D3/6-311++G(2d,2p) Electronic Energy = –1776.962543

IEFPCM(MeCN)B3LYP-D3/6-311++G(2d,2p) Free Energy = –1776.639468

Number of imaginary frequencies = 0

C	0.453538	-1.092696	-1.718101
C	-0.757787	-1.593322	-1.474997
H	-1.520406	-1.637078	-2.236183
C	1.571485	-0.908123	-0.794342
C	2.926982	-1.129462	-1.364640
O	3.079484	-1.378906	-2.551836
C	-2.595107	-3.326162	-0.453799
H	-3.032662	-3.755074	0.442290
H	-3.318859	-2.734132	-1.001848
H	-2.147611	-4.102468	-1.069461
C	1.424176	-0.524808	0.489802
H	0.429164	-0.335280	0.871662
S	-1.244411	-2.251991	0.111128
N	-1.894407	-1.108822	1.097016
S	-2.849362	0.059764	0.475031
O	-3.549916	-0.350271	-0.744512
O	-3.652882	0.543858	1.593185
C	-1.734675	1.378662	0.025194
C	-1.470640	1.650768	-1.308493
C	-1.096061	2.095729	1.034416
C	-0.542899	2.634121	-1.633971
H	-1.978917	1.097386	-2.081639
C	-0.179679	3.076911	0.695827
H	-1.312648	1.883491	2.070509
C	0.122873	3.353322	-0.642222
H	-0.333821	2.840804	-2.674572
H	0.315553	3.633144	1.479813
C	1.156904	4.388294	-0.990919
H	1.136152	4.628382	-2.051930
H	1.000845	5.306839	-0.425407
H	2.157772	4.025709	-0.748106
H	0.651793	-0.800715	-2.741915
C	2.539689	-0.330396	1.476566
C	4.089812	-1.082233	-0.404999
H	4.211885	-2.098101	-0.017316
H	4.990305	-0.852270	-0.971219
C	3.867844	-0.096040	0.738262
H	4.694886	-0.149028	1.446315
H	3.861127	0.917660	0.332480
C	2.229338	0.879477	2.370420
H	2.150671	1.790795	1.780040
H	1.291325	0.740321	2.907717
H	3.025968	1.012430	3.102700
C	2.599580	-1.600631	2.357043
H	3.359963	-1.473563	3.127989
H	1.641777	-1.773330	2.847082
H	2.841223	-2.486453	1.772359

Transition State TS-S-Ja

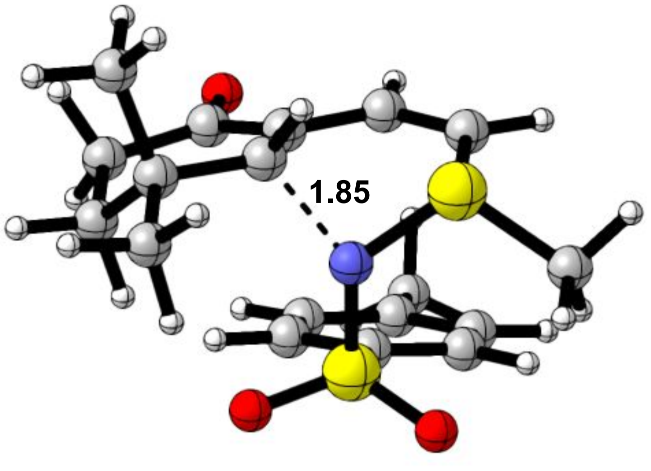

IEFPCM(MeCN)B3LYP-D3/6-311++G(2d,2p) Electronic Energy = –1776.921395

IEFPCM(MeCN)B3LYP-D3/6-311++G(2d,2p) Free Energy = –776.593239

Number of imaginary frequencies = 1 (368.91i cm^{-1})

C	0.194571	-0.264298	-2.046458	C	-0.419460	-2.314976	-0.825008
C	0.241186	1.073776	-2.249380	C	-1.814293	-0.230700	-0.635028
H	0.957769	1.584983	-2.870082	O	0.472540	-2.997926	-1.366570
S	-0.887794	2.131862	-1.379656	C	-1.287570	-2.974378	0.236109
S	-0.029998	1.357976	1.340381	C	-2.819914	-0.943691	0.255991
C	1.437120	0.463872	0.902336	H	-2.360900	0.340640	-1.381132
C	2.494036	1.121885	0.284302	H	-0.627391	-3.580341	0.857021
C	1.521648	-0.889785	1.203609	H	-1.939574	-3.681148	-0.283149
C	3.629100	0.405692	-0.062050	C	-2.109345	-2.014154	1.094070
H	2.446572	2.181667	0.096266	C	-3.822500	-1.605166	-0.722011
C	2.663306	-1.592833	0.853433	C	-3.605464	0.022927	1.147543
H	0.712524	-1.382532	1.715320	H	-1.469855	-1.514597	1.818066
C	3.726450	-0.963087	0.202759	H	-2.854088	-2.570621	1.665216
H	4.452832	0.918111	-0.539124	H	-4.534609	-2.203776	-0.154226
H	2.726212	-2.646305	1.085579	H	-4.379016	-0.844712	-1.269665
C	4.940918	-1.743847	-0.215529	H	-3.332618	-2.251709	-1.446850
H	5.821475	-1.106693	-0.274656	H	-4.412695	-0.520850	1.639164
H	5.144891	-2.558786	0.477027	H	-2.970170	0.466104	1.906927
H	4.785606	-2.184777	-1.202623	H	-4.048659	0.825146	0.556996
O	0.348532	2.746495	1.548004	C	0.063701	3.646250	-1.142447
O	-0.680897	0.636741	2.417165	H	1.056739	3.447833	-0.767774
N	-1.175906	1.344611	0.100543	H	-0.502306	4.278346	-0.468472
H	0.962185	-0.856560	-2.527879	H	0.093051	4.081808	-2.139554
C	-0.689048	-0.956387	-1.175005				

Transition State TS-S-Jb

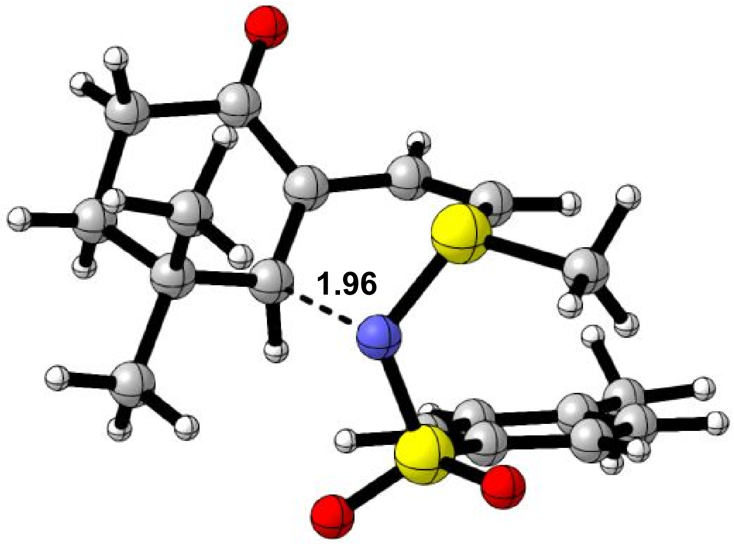

IEFPCM(MeCN)B3LYP-D3/6-311++G(2d,2p) Electronic Energy = –1776.912849

IEFPCM(MeCN)B3LYP-D3/6-311++G(2d,2p) Free Energy = –1776.587975

Number of imaginary frequencies = 1 (386.19i cm^{-1})

C	-0.258482	1.736516	1.021433	H	5.572155	2.592104	0.117082
C	0.480163	0.858221	1.724795	H	5.365981	2.723810	-1.630227
H	1.418068	1.027649	2.225290	H	0.016380	2.784663	1.029638
C	0.875884	-1.703440	2.826039	C	-1.465750	1.322121	0.341247
H	0.522405	-2.727485	2.790916	C	-2.594808	2.196218	0.458834
H	1.872534	-1.619235	2.417481	C	-1.517032	0.148902	-0.507143
H	0.827219	-1.315919	3.840429	O	-2.555791	3.241698	1.139464
S	-0.314834	-0.703694	1.901543	C	-3.870090	1.866028	-0.295923
N	-0.517411	-1.319004	0.326962	C	-2.881629	-0.423674	-0.852495
S	0.821757	-1.865238	-0.538650	H	-0.820458	0.115776	-1.330299
O	1.414757	-2.971597	0.197369	H	-4.237065	2.794580	-0.733407
O	0.310403	-2.091076	-1.878522	H	-4.613998	1.565916	0.445860
C	2.037280	-0.571340	-0.627447	C	-3.703196	0.787982	-1.357403
C	3.144527	-0.607642	0.208511	C	-3.560912	-1.057492	0.371501
C	1.849378	0.489430	-1.510115	C	-2.790148	-1.461239	-1.973008
C	4.048166	0.447066	0.189457	H	-3.206271	1.206089	-2.235527
H	3.308932	-1.454806	0.854864	H	-4.676830	0.424211	-1.689423
C	2.756231	1.534452	-1.516526	H	-4.589424	-1.320137	0.123353
H	1.011561	0.498046	-2.188783	H	-3.036071	-1.965075	0.662547
C	3.862914	1.538281	-0.659191	H	-3.576461	-0.388646	1.228917
H	4.908575	0.418169	0.843116	H	-3.791950	-1.795987	-2.243176
H	2.605716	2.360970	-2.197214	H	-2.319892	-1.041384	-2.862281
C	4.835240	2.684409	-0.677584	H	-2.207660	-2.324100	-1.661878
H	4.316301	3.635756	-0.558685				

Intermediate S-K

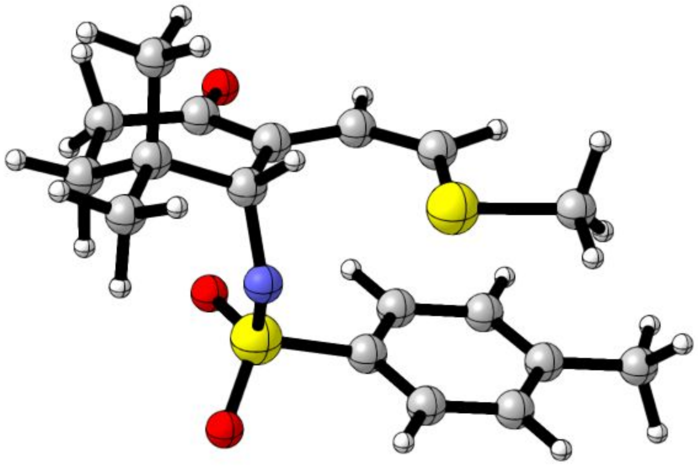

IEFPCM(MeCN)B3LYP-D3/6-311++G(2d,2p) Electronic Energy = –1776.946916

IEFPCM(MeCN)B3LYP-D3/6-311++G(2d,2p) Free Energy = –1776.621284

Number of imaginary frequencies = 0

C	-0.147972	0.999890	-1.616944	C	-2.193252	-0.295063	-1.922712
C	0.911568	1.719341	-1.040046	O	-1.861337	-0.528879	-3.070505
H	1.700528	2.044026	-1.704262	C	-3.521386	-0.768545	-1.387955
S	1.118662	2.106504	0.568211	C	-3.153576	0.648584	0.694488
S	-0.261137	-1.527850	0.852609	H	-3.658346	-1.790533	-1.737921
C	1.451678	-1.271153	0.387388	H	-4.270178	-0.169127	-1.913361
C	1.840401	-1.307940	-0.942021	C	-3.713721	-0.658048	0.122780
C	2.367035	-0.894039	1.369465	C	-3.799880	1.870873	0.022212
C	3.143489	-0.947484	-1.294048	C	-3.419543	0.709942	2.199425
H	1.131995	-1.606938	-1.698392	H	-3.219992	-1.494002	0.614293
C	3.656719	-0.540976	1.010579	H	-4.776977	-0.730309	0.355396
H	2.061935	-0.863917	2.404839	H	-4.885999	1.817978	0.098983
C	4.065985	-0.552580	-0.330769	H	-3.468458	2.789574	0.507461
H	3.435101	-0.971488	-2.335215	H	-3.549616	1.956752	-1.036092
H	4.359127	-0.243390	1.777824	H	-4.493847	0.745726	2.384785
C	5.466031	-0.147281	-0.704281	H	-3.007126	-0.160234	2.704561
H	5.682303	0.868786	-0.370070	H	-2.970188	1.601441	2.639974
H	5.615754	-0.189315	-1.781090	C	-1.625396	0.709359	0.427228
H	6.200731	-0.801615	-0.232839	H	-1.339065	1.741055	0.676664
O	-0.914354	-2.100051	-0.347739	C	2.687316	3.035627	0.524555
O	-0.206291	-2.410735	2.026462	H	2.987463	3.155157	1.560783
N	-0.816828	-0.127061	1.306053	H	2.522271	4.004995	0.065842
H	-0.029334	0.779264	-2.668131	H	3.430523	2.458704	-0.014759
C	-1.283056	0.523777	-1.026098				

Transition State TS-S-K

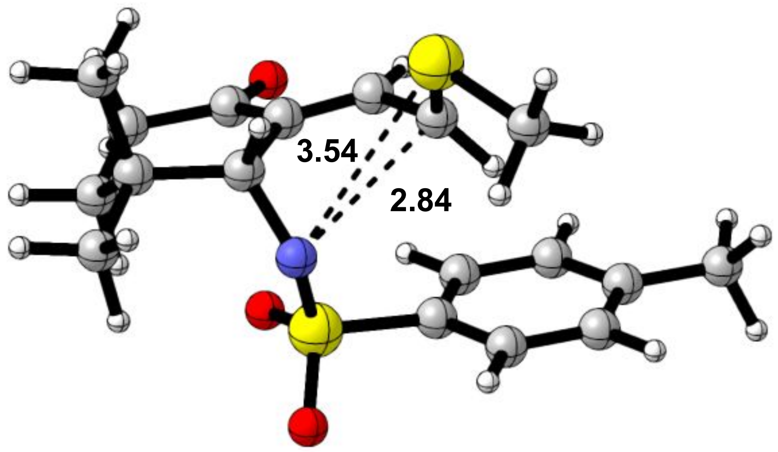

IEFPCM(MeCN)B3LYP-D3/6-311++G(2d,2p) Electronic Energy = –1776.927344

IEFPCM(MeCN)B3LYP-D3/6-311++G(2d,2p) Free Energy = –1776.603700

Number of imaginary frequencies = 1 (147.78i cm^{-1})

C	-0.035329	0.751785	-1.588079	C	-1.903210	-0.746799	-2.000527
C	0.824407	1.554400	-0.748027	O	-1.375556	-1.174751	-3.013151
H	1.780430	1.148409	-0.440213	C	-3.278855	-1.191563	-1.576654
S	0.497744	3.128083	-0.389622	C	-3.186374	0.304889	0.496717
S	-0.103081	-1.237050	1.283646	H	-3.420219	-2.210624	-1.932682
C	1.581410	-1.124137	0.668506	H	-3.974347	-0.569199	-2.148924
C	1.894041	-1.603064	-0.595433	C	-3.566660	-1.065523	-0.080938
C	2.547067	-0.458081	1.421546	C	-4.009381	1.411872	-0.188870
C	3.169349	-1.394479	-1.116493	C	-3.489100	0.323084	1.995697
H	1.141594	-2.119116	-1.171088	H	-3.023702	-1.840452	0.451333
C	3.812340	-0.255150	0.892718	H	-4.631829	-1.233510	0.086931
H	2.300930	-0.086051	2.404906	H	-5.074404	1.184130	-0.137844
C	4.144643	-0.715297	-0.388434	H	-3.843229	2.369688	0.305202
H	3.403047	-1.761428	-2.106740	H	-3.746028	1.538800	-1.239463
H	4.554073	0.272794	1.477142	H	-4.561204	0.201652	2.158091
C	5.524403	-0.493920	-0.946388	H	-2.966733	-0.479833	2.511290
H	5.846132	0.537442	-0.801555	H	-3.181104	1.266703	2.447211
H	5.561443	-0.719434	-2.010330	C	-1.679505	0.643202	0.251336
H	6.252661	-1.132706	-0.443012	H	-1.655120	1.731247	0.322870
O	-0.774146	-2.198547	0.385284	C	1.850652	3.594310	0.734823
O	0.050721	-1.667250	2.682305	H	1.841213	4.678182	0.784325
N	-0.697651	0.230079	1.248892	H	2.793438	3.236134	0.336007
H	0.385307	0.383351	-2.517227	H	1.643050	3.165417	1.710366
C	-1.210665	0.280420	-1.152957				

Intermediate S-L

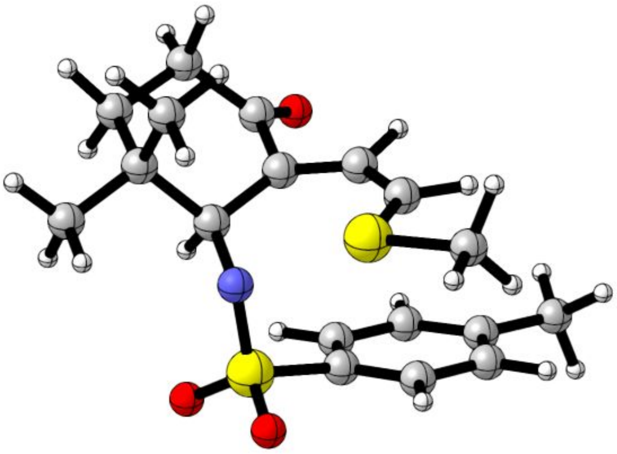

IEFPCM(MeCN)B3LYP-D3/6-311++G(2d,2p) Electronic Energy = –1776.944928

IEFPCM(MeCN)B3LYP-D3/6-311++G(2d,2p) Free Energy = –1776.622889

Number of imaginary frequencies = 0

C	0.054981	-0.646257	-1.618204	H	4.989147	-1.864374	-1.444188
C	0.653861	0.576684	-1.985959	H	5.519067	-2.347355	0.160676
H	1.471549	0.479724	-2.687953	H	4.296868	-3.297491	-0.674912
C	1.451022	3.034425	-2.704778	C	-0.915737	-0.962535	-0.714629
H	1.304564	4.089585	-2.494665	C	-1.493850	-2.345342	-0.841651
H	2.459586	2.738942	-2.432805	O	-0.789595	-3.334011	-0.879933
H	1.247992	2.830728	-3.751446	C	-2.995113	-2.361420	-0.956155
C	-1.524789	-0.144135	0.376927	H	-3.344336	-3.387033	-0.858534
S	0.237902	2.152236	-1.648261	H	-3.222410	-2.033636	-1.975510
N	-0.872616	1.107636	0.616199	C	-3.672962	-1.422531	0.051635
S	0.166044	1.142917	1.796974	H	-3.606556	-1.869183	1.045136
O	0.645269	2.523651	1.937714	H	-4.733136	-1.353630	-0.192212
O	-0.312170	0.491833	3.032397	C	-3.076838	-0.005148	0.112883
C	1.580855	0.163156	1.282921	C	-3.328308	0.782383	-1.177811
C	2.603715	0.752457	0.547627	C	-3.696950	0.738219	1.296855
C	1.565518	-1.216083	1.463848	H	-4.399733	0.896382	-1.343725
C	3.587330	-0.041050	-0.028111	H	-2.887489	1.774513	-1.103088
H	2.620889	1.823750	0.420200	H	-2.904672	0.292817	-2.054679
C	2.555010	-2.001249	0.884414	H	-4.776121	0.814791	1.158498
H	0.779615	-1.674487	2.044760	H	-3.507211	0.211996	2.232594
C	3.574265	-1.431184	0.120810	H	-3.282595	1.739516	1.380959
H	4.376258	0.425280	-0.603330	H	0.420394	-1.478321	-2.207027
H	2.527014	-3.074099	1.019797	H	-1.483098	-0.790720	1.268751
C	4.649914	-2.282952	-0.497542				

Transition State TS-S-L

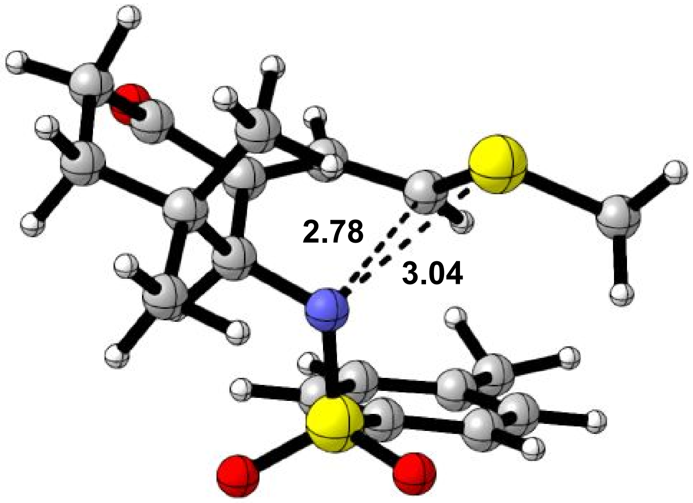

IEFPCM(MeCN)B3LYP-D3/6-311++G(2d,2p) Electronic Energy = –1776.938680

IEFPCM(MeCN)B3LYP-D3/6-311++G(2d,2p) Free Energy = –1776.616172

Number of imaginary frequencies = 1 (161.18i cm^{-1})

C	-0.265200	1.559446	0.924891	H	4.745698	3.046289	-0.420658
C	0.689011	0.712897	1.567991	H	5.655758	1.767265	0.391924
H	1.738908	0.948880	1.428036	H	5.760824	1.957843	-1.354571
C	-1.331109	1.247026	0.169360	H	-0.030126	2.616832	1.024719
C	1.946953	-1.020855	3.260385	C	-2.253406	2.365007	-0.209655
H	1.845904	-1.318254	4.299428	O	-1.840014	3.470233	-0.509211
H	2.175894	-1.884424	2.643888	C	-3.718332	2.018191	-0.138617
H	2.702751	-0.250528	3.155371	H	-4.283923	2.784943	-0.663798
S	0.322397	-0.372694	2.758873	H	-3.989975	2.085388	0.919502
N	-0.701052	-1.114591	-0.007081	C	-4.030638	0.612275	-0.663910
S	0.279234	-1.700630	-1.089180	H	-3.936717	0.612322	-1.751634
O	0.720879	-3.032948	-0.654386	H	-5.071505	0.377354	-0.439576
O	-0.209899	-1.599416	-2.478510	C	-3.129573	-0.498055	-0.095489
C	1.760450	-0.677198	-1.035619	C	-1.636761	-0.110016	-0.413776
C	2.849847	-1.064191	-0.265533	C	-3.324223	-0.676317	1.413017
C	1.755874	0.569710	-1.655720	C	-3.455636	-1.809834	-0.812230
C	3.918488	-0.191011	-0.089800	H	-1.621460	0.039940	-1.502499
H	2.852755	-2.038151	0.199241	H	-4.347545	-0.986472	1.626196
C	2.825153	1.434201	-1.471772	H	-2.648321	-1.442734	1.788111
H	0.914234	0.868458	-2.262535	H	-3.127798	0.239308	1.970682
C	3.921296	1.073544	-0.680347	H	-4.503225	-2.069849	-0.655123
H	4.758649	-0.496629	0.519340	H	-3.280075	-1.723348	-1.884663
H	2.808311	2.407114	-1.944622	H	-2.835301	-2.618605	-0.432542
C	5.082777	2.013417	-0.500326				

Intermediate S-M

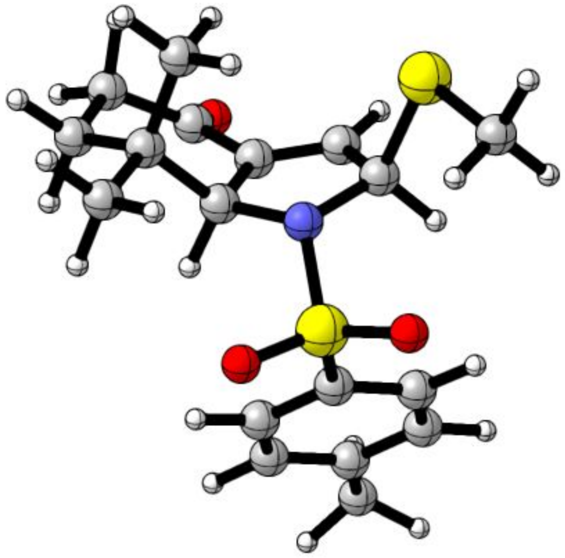

IEFPCM(MeCN)B3LYP-D3/6-311++G(2d,2p) Electronic Energy = –-1777.010981

IEFPCM(MeCN)B3LYP-D3/6-311++G(2d,2p) Free Energy = –-1776.684018

Number of imaginary frequencies = 0

C	0.636561	0.187572	-1.824698	H	1.624633	-3.784688	-0.082157
C	0.828711	1.179271	-0.958995	H	2.485231	-4.425696	-1.500340
N	0.745067	-0.789148	0.304447	H	0.742128	-4.100042	-1.596286
S	-0.506819	-1.412414	1.236348	H	0.555412	0.282272	-2.895414
C	-1.994349	-0.620382	0.682254	C	1.098676	2.595398	-1.287138
C	-2.713338	-1.169035	-0.376992	O	0.553320	3.171671	-2.212242
C	-2.400234	0.571937	1.275047	C	2.169155	3.243410	-0.433799
C	-3.831060	-0.502128	-0.854317	H	1.994174	4.317786	-0.416620
H	-2.417096	-2.113777	-0.805402	H	3.103639	3.090772	-0.982718
C	-3.520786	1.226067	0.783679	C	2.299047	2.671566	0.985649
H	-1.861676	0.969570	2.120977	H	1.466347	3.033508	1.592776
C	-4.246322	0.708410	-0.291855	H	3.207427	3.065355	1.441128
H	-4.394273	-0.931939	-1.670990	C	2.330008	1.126923	1.069795
H	-3.840175	2.148449	1.248353	C	0.984560	0.678410	0.450346
O	-0.587838	-2.821633	0.902327	C	3.531710	0.549691	0.316804
O	-0.250370	-0.993326	2.599010	C	2.409578	0.710331	2.539711
C	-5.439079	1.440158	-0.841245	H	0.202703	1.098559	1.082884
H	-5.141853	2.074655	-1.678931	H	4.455203	0.940680	0.744057
H	-5.892520	2.080881	-0.087400	H	3.546508	-0.535000	0.398967
H	-6.193022	0.746142	-1.209182	H	3.521724	0.794231	-0.743931
C	0.674684	-1.138464	-1.137729	H	3.336761	1.079577	2.978945
H	-0.195063	-1.753565	-1.347749	H	1.573851	1.112329	3.111634
S	2.118349	-2.104500	-1.759544	H	2.389965	-0.373129	2.637570
C	1.684000	-3.771794	-1.165338				

Intermediate S-N

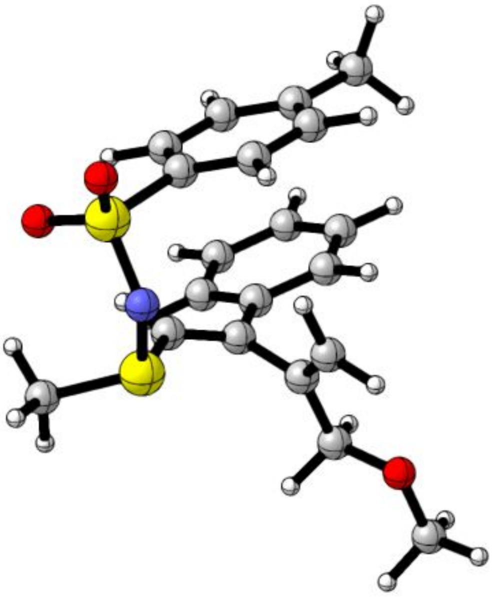

IEFPCM(MeCN)B3LYP-D3/6-311++G(2d,2p) Electronic Energy = –1853.170982

IEFPCM(MeCN)B3LYP-D3/6-311++G(2d,2p) Free Energy = –1852.839142

Number of imaginary frequencies = 0

C	-0.928028	-0.709933	0.512929	O	3.466656	-0.057075	1.358297
C	0.252218	-0.966177	1.094298	O	4.363800	-0.153053	-0.974510
H	0.651982	-0.356328	1.888033	C	2.072689	1.050253	-0.554625
C	-1.446285	-1.508242	-0.626750	C	1.567657	1.925822	0.394619
C	-1.744762	0.429482	0.988222	C	1.664463	1.135111	-1.882767
C	-1.728135	0.837636	2.326367	C	0.632077	2.881162	0.015297
C	-2.502435	1.161848	0.068159	H	1.888546	1.848037	1.421624
C	-2.434587	1.962827	2.728260	C	0.731897	2.092390	-2.247704
H	-1.175055	0.264779	3.056481	H	2.063623	0.452805	-2.618533
C	-3.205129	2.288808	0.470621	C	0.194766	2.975591	-1.304359
H	-2.511976	0.862533	-0.969679	H	0.222603	3.547224	0.761830
C	-3.170882	2.695360	1.800761	H	0.405210	2.148956	-3.277330
H	-2.416066	2.264583	3.765875	C	-0.835217	3.991880	-1.712716
H	-3.771930	2.854023	-0.255184	H	-1.210274	4.541159	-0.852263
H	-3.718648	3.572592	2.114583	H	-0.415938	4.708500	-2.420761
C	2.087294	-2.688403	2.142694	H	-1.681645	3.510020	-2.202940
H	2.861636	-3.418134	1.925995	C	-2.744416	-2.219364	-0.354993
H	2.504519	-1.799593	2.600023	O	-3.228151	-2.849450	-1.520664
H	1.301999	-3.133663	2.748333	C	-4.431587	-3.565296	-1.279301
C	-0.804086	-1.577143	-1.789253	H	-5.222115	-2.897199	-0.925052
H	-1.204511	-2.159391	-2.604935	H	-4.733412	-4.011108	-2.223167
H	0.116601	-1.039209	-1.949086	H	-4.277330	-4.355319	-0.538480
S	1.327997	-2.265802	0.544948	H	-2.580142	-2.961598	0.438553
N	2.499400	-1.653885	-0.439719	H	-3.483538	-1.507055	0.029612
S	3.216161	-0.236977	-0.074888				

Transition State TS-S-N

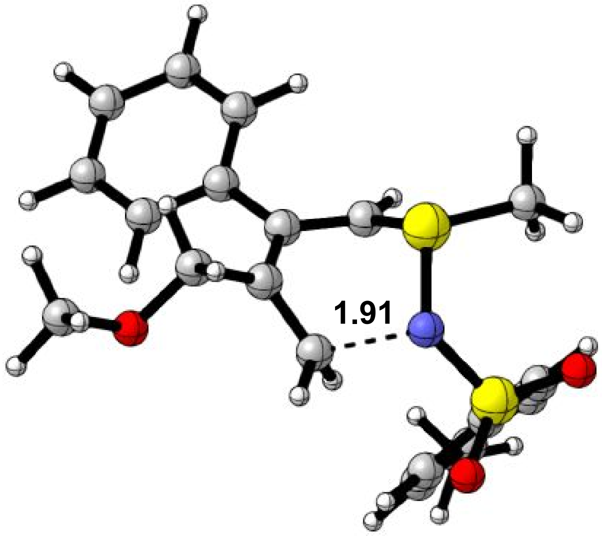

IEFPCM(MeCN)B3LYP-D3/6-311++G(2d,2p) Electronic Energy = –1853.112253

IEFPCM(MeCN)B3LYP-D3/6-311++G(2d,2p) Free Energy = –1852.781799

Number of imaginary frequencies = 1 (503.36 cm^{-1})

C	-1.640396	0.072852	0.508143	O	3.526941	-1.810580	1.290780
C	-0.550432	0.362710	1.320401	O	2.845646	-2.477609	-1.036150
H	-0.272065	1.309301	1.753040	C	2.900476	0.061783	-0.421604
C	-1.507314	-1.112364	-0.259109	C	3.426846	1.010445	0.446568
C	-2.841608	0.928201	0.493644	C	2.550683	0.413315	-1.723004
C	-3.200969	1.679464	1.620108	C	3.566663	2.323947	0.020153
C	-3.638304	1.018710	-0.655371	H	3.751806	0.720120	1.432885
C	-4.318749	2.503286	1.597001	C	2.695112	1.729565	-2.134799
H	-2.609013	1.597003	2.520495	H	2.193029	-0.336149	-2.411440
C	-4.760488	1.836932	-0.674981	C	3.191468	2.708178	-1.268890
H	-3.366829	0.446913	-1.530727	H	3.983485	3.057742	0.696063
C	-5.103132	2.584198	0.449094	H	2.426545	1.998998	-3.146951
H	-4.584703	3.073730	2.475988	C	3.310982	4.139106	-1.714537
H	-5.364723	1.897571	-1.569483	H	3.569218	4.203554	-2.770420
H	-5.975442	3.222472	0.431791	H	2.361223	4.660659	-1.578185
C	1.495719	-0.391183	3.033957	H	4.065162	4.671484	-1.138111
H	2.228503	-1.166003	3.220327	C	-2.645726	-2.034003	-0.552557
H	1.958561	0.494947	2.619666	O	-2.986500	-1.992024	-1.948822
H	0.940193	-0.154706	3.936449	H	-2.377989	-3.066394	-0.296201
C	-0.215867	-1.415155	-0.781005	C	-4.067025	-2.859801	-2.252425
H	-0.117997	-2.298613	-1.394441	H	-4.968875	-2.575976	-1.699888
H	0.348452	-0.563064	-1.123383	H	-4.261148	-2.777854	-3.319132
S	0.261664	-1.058444	1.887550	H	-3.821628	-3.899057	-2.009882
N	1.078323	-1.863277	0.553575	H	-3.524291	-1.763686	0.039431
S	2.669207	-1.618397	0.127193				

Intermediate S-O

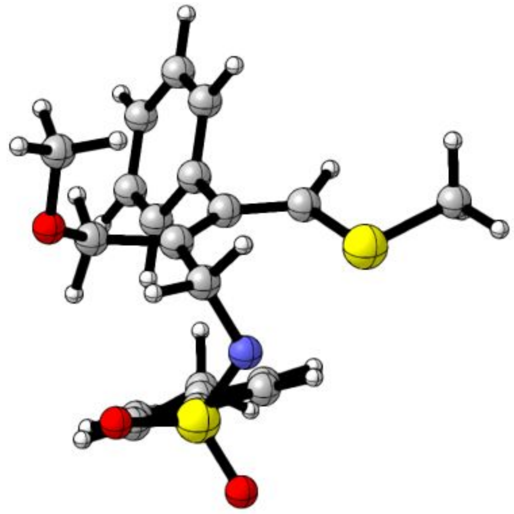

IEFPCM(MeCN)B3LYP-D3/6-311++G(2d,2p) Electronic Energy = –1853.162088

IEFPCM(MeCN)B3LYP-D3/6-311++G(2d,2p) Free Energy = –1852.828507

Number of imaginary frequencies = 0

C	-0.374822	-1.368331	0.290687
C	-0.117234	-1.653141	1.643068
H	-0.990352	-1.854606	2.249524
C	0.580158	-1.320395	-0.710468
C	-1.827109	-1.181030	-0.022611
C	-2.745663	-2.204947	0.216633
C	-2.280775	0.034112	-0.534621
C	-4.095344	-2.016629	-0.058553
H	-2.403291	-3.154332	0.605503
C	-3.630488	0.223299	-0.807231
H	-1.581031	0.834625	-0.711068
C	-4.541414	-0.801125	-0.570224
H	-4.796093	-2.819183	0.122938
H	-3.967469	1.172438	-1.199428
H	-5.591156	-0.654638	-0.781506
C	0.737385	-2.261396	4.129138
H	1.607020	-2.228048	4.778360
H	-0.027713	-1.586739	4.499590
H	0.367726	-3.278664	4.053334
C	2.056872	-1.423225	-0.466900
H	2.272399	-2.324722	0.114347
H	2.566893	-1.529680	-1.421813
S	1.324425	-1.689166	2.494805
N	2.535830	-0.285583	0.312308
S	2.550744	1.127719	-0.392987
O	3.542710	1.958795	0.308728
O	2.676516	1.079026	-1.862527
C	0.983098	1.957095	-0.079555
C	0.423820	1.899190	1.194564
C	0.352539	2.680689	-1.082464
C	-0.762398	2.565928	1.457153
H	0.911625	1.326460	1.968972
C	-0.837698	3.349537	-0.806842
H	0.788942	2.718505	-2.068805
C	-1.415677	3.300114	0.460770
H	-1.196080	2.509001	2.446700
H	-1.326181	3.908241	-1.593785
C	-2.729434	3.975527	0.744849
H	-3.538838	3.242661	0.769728
H	-2.715959	4.475644	1.712914
H	-2.970772	4.710812	-0.020415
C	0.166007	-1.118802	-2.145349
O	0.956537	-1.850065	-3.060556
C	0.723705	-3.255097	-2.987545
H	1.317884	-3.714519	-3.771940
H	1.031057	-3.662994	-2.020974
H	-0.333477	-3.481522	-3.148436
H	0.312234	-0.062931	-2.386966
H	-0.890171	-1.353851	-2.284175

Transition State TS-S-O

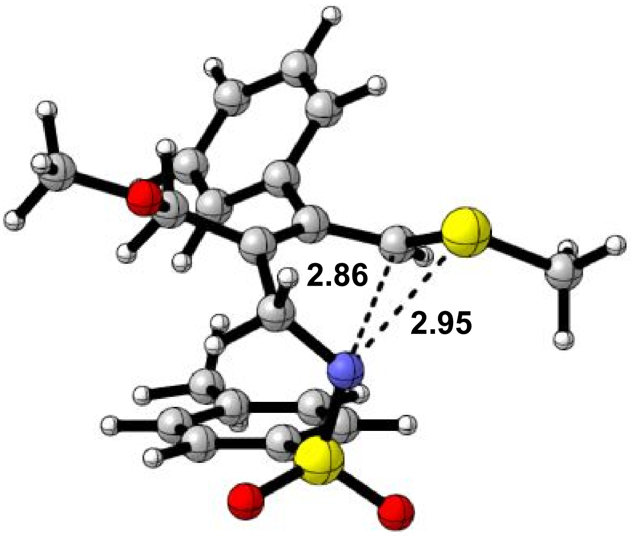

IEFPCM(MeCN)B3LYP-D3/6-311++G(2d,2p) Electronic Energy = –1853.151825

IEFPCM(MeCN)B3LYP-D3/6-311++G(2d,2p) Free Energy = –1852.822027

Number of imaginary frequencies = 1 (114.84i cm^{-1})

C	0.541502	-1.123366	-0.399425	O	-3.765900	1.164015	-0.755613
C	-0.078389	-1.069797	-1.683277	O	-3.390729	0.865132	1.689525
H	0.293156	-0.343345	-2.395622	C	-1.422043	1.763611	0.247794
C	-0.102891	-1.427813	0.764796	C	-0.878720	2.093322	-0.992295
C	1.994512	-0.779079	-0.447528	C	-0.797894	2.191061	1.410073
C	2.834144	-1.422194	-1.362074	C	0.283789	2.842532	-1.060797
C	2.525849	0.214676	0.377576	H	-1.370252	1.764710	-1.896703
C	4.181927	-1.088882	-1.437884	C	0.369951	2.948607	1.331285
H	2.435165	-2.195252	-2.004087	H	-1.224049	1.937720	2.368749
C	3.871745	0.547101	0.301739	C	0.931696	3.280747	0.101360
H	1.879715	0.744477	1.059293	H	0.699988	3.092771	-2.027831
C	4.703714	-0.104372	-0.605022	H	0.851575	3.276524	2.242889
H	4.821554	-1.597819	-2.144829	C	2.214067	4.061412	0.010740
H	4.268470	1.320135	0.944330	H	3.015480	3.437345	-0.389089
H	5.750757	0.156858	-0.664288	H	2.108542	4.918111	-0.655505
C	-1.580432	-1.549295	-3.900747	H	2.527154	4.423767	0.987922
H	-1.964619	-2.385800	-4.475512	C	0.683257	-1.677179	2.023343
H	-2.354492	-0.800833	-3.761228	O	0.007065	-2.646059	2.804623
H	-0.705303	-1.126737	-4.383840	H	1.700797	-2.005769	1.802730
C	-1.601250	-1.418328	0.960852	C	0.621087	-2.821939	4.075182
H	-1.908632	-2.468271	1.036849	H	0.603325	-1.890921	4.648779
H	-1.771514	-1.028638	1.969962	H	0.050711	-3.582358	4.601080
S	-1.121895	-2.214142	-2.271492	H	1.657453	-3.154550	3.968056
N	-2.399732	-0.756278	-0.044869	H	0.753559	-0.733719	2.582830
S	-2.871706	0.695760	0.315036				

Chloramine-T

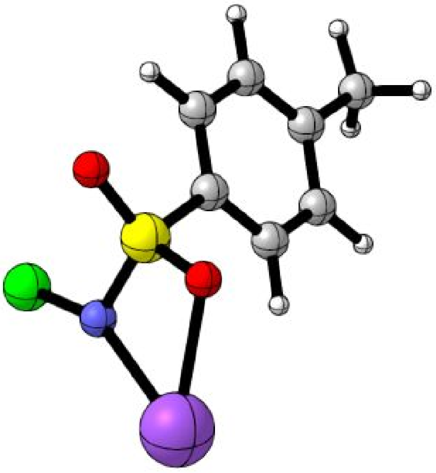

IEFPCM(MeCN)B3LYP-D3/6-311++G(2d,2p) Electronic Energy = –1497.137587

IEFPCM(MeCN)B3LYP-D3/6-311++G(2d,2p) Free Energy = –1497.047603

Number of imaginary frequencies = 0

C	-3.250370	0.223456	-0.188322	H	-5.277691	-0.498668	-0.107876
C	-2.766965	-0.813097	0.607778	H	-5.133682	1.259789	-0.125552
C	-1.413028	-0.911497	0.917144	H	-4.867221	0.347937	-1.601754
C	-0.530325	0.037456	0.425178	N	2.015417	-0.176170	-0.619398
C	-0.988857	1.078741	-0.379446	O	1.375015	-1.196005	1.701684
C	-2.338253	1.168435	-0.676207	O	1.658870	1.249929	1.263156
H	-3.453562	-1.555315	0.991512	S	1.219010	-0.075690	0.772768
H	-1.044044	-1.716161	1.533666	Cl	1.639928	-1.695057	-1.468478
H	-0.293676	1.807775	-0.768960	Na	3.000722	2.045249	-0.591892
H	-2.693366	1.979345	-1.298009				
C	-4.713308	0.334233	-0.522008				

Sodium Chloride

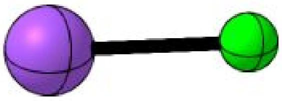

IEFPCM(MeCN)B3LYP-D3/6-311++G(2d,2p) Electronic Energy = –622.670413

IEFPCM(MeCN)B3LYP-D3/6-311++G(2d,2p) Free Energy = –622.692973

Number of imaginary frequencies = 0

Na	0.000000	0.000000	-1.607710
Cl	0.000000	0.000000	1.040283

7 References

(1) Still, W. C.; Kahn, M.; Mitra, A. Rapid chromatographic technique for preparative separations with moderate resolution. *J. Org. Chem.* **1978***, 43*, 2923–2925.

(2) Kofron, W. G.; Baclawski, L. M. A convenient method for estimation of alkyllithium concentrations. *J. Org. Chem.* **1976***, 41*, 1879–1880.

(3) Krasovskiy, A.; Knochel, P. Convenient Titration Method for Organometallic Zinc, Magnesium, and Lanthanide Reagents. *Synthesis* **2006***, 5*, 890–891.

(4) Meisenbichler, C., Kluibenschedl, F.; Müller, T. A 3-in-1 Hand-held Ambient Mass Spectrometry Interface for Identification and 2D Localization of Chemicals on Surfaces. *Anal. Chem.* **2020**, *92*, 14314–14318.

(5) Pavri, N. P.; Trudell, M. L. An Efficient Method for the Synthesis of 3-Arylpyrroles, *J. Org. Chem.* **1997**, *62*, 2649−2651.

(6) Haut, F.-L.; Habiger, C.; Wein, L. A.; Wurst, K.; Podewitz, M.; Magauer, T. Rapid Assembly of Tetrasubstituted Furans via Pummerer-Type Rearrangement. *J. Am. Chem. Soc.* **2021**, *143*, 1216–1223.

(7) Fernández-Salas, J. A.; Pulis, A. P.; Procter, D. J. Metal-free C–H thioarylation of arenes using sulfoxides: a direct, general diaryl sulfide synthesis. *Chem. Commun.* **2016**, *52*, 12364–12367.

(8) White, L. A.; Storr, R. C. Dihydrothiophenes as precursors to fused quinolines, quinolones and coumarins via o-quinodimethane intermediates. *Tetrahedron* **1996**, *52*, 3117–3134.

(9) Haut, F.-L.; Habiger, C.; Speck, K.; Wurst, K.; Mayer, P.; Korber, J. N.; Müller, T.; Magauer, T. Synthetic Entry to Polyfunctionalized Molecules through the [3+2]-Cycloaddition of Thiocarbonyl Ylides. *J. Am. Chem. Soc.* **2019***, 141*, 13352−13357.

(10) Alvaro, G.; Dambruoso, P.; Marasco, A.; Tommasi, S.; Decor, A.; Large, C. Preparation of hydantoin derivatives as Kv3 channel inhibitors. WO2012076877. June 14, 2012.

(11) Candy, M.; Guyon, C.; Mersmann, S.; Chen, J.-R.; Bolm, C. Synthesis of Sulfondiimines by *N*-Chlorosuccinimide-Mediated Oxidative Imination of Sulfiliminium Salts. *Angew. Chem. Int. Ed.* **2012**, *51*, 4440–4443.

(12) Wang, T.; Hong, T.; Tang, T.; Zhai, Q.; Xing, X.; Mao, W.; Zheng, X.; Xu, L.; Wu, J.; Weng, X.; Wang, S.; Tian, T.; Yuan, B.; Huang, B.; Zhuang, L.; Zhou, X. Application of *N*-Halogeno-*N*-sodiobenzenesulfonamide Reagents to the Selective Detection of 5-Methylcytosine in DNA Sequences. *J. Am. Chem. Soc.* **2013**, *135*, 1240−1243.

(13) Boberg, F.; Paetz, A.; Bruchmann, B.; Garming, A. Reaktionen von Thioxoheterocyclen mit *N*-Chloramiden I[1]. *N*-(2-Pyridylthio)amide. *Phosphorus Sulfur Relat. Elem.* **1987**, *33*, 99−107.

(14) Landais, Y.; Zekri, E. Desymmetrization of Cyclohexa-1,4-dienes − A Straightforward Route to Cyclic and Acyclic Polyhydroxylated Systems. *Eur. J. Org. Chem.* **2002**, 4037−4053.

(15) Gaussian 16, Revision C.01, Frisch, M. J.; Trucks, G. W.; Schlegel, H. B.; Scuseria, G. E.; Robb, M. A.; Cheeseman, J. R.; Scalmani, G.; Barone, V.; Petersson, G. A.; Nakatsuji, H.; Li, X.; Caricato, M.; Marenich, A. V.; Bloino, J.; Janesko, B. G.; Gomperts, R.; Mennucci, B.; Hratchian, H. P.; Ortiz, J. V.; Izmaylov, A. F.; Sonnenberg, J. L.; Williams-Young, D.; Ding, F.; Lipparini, F.; Egidi, F.; Goings, J.; Peng, B.; Petrone, A.; Henderson, T.; Ranasinghe, D.; Zakrzewski, V. G.; Gao, J.; Rega, N.; Zheng, G.; Liang, W.; Hada, M.; Ehara, M.; Toyota, K.; Fukuda, R.; Hasegawa, J.; Ishida, M.; Nakajima, T.; Honda, Y.; Kitao, O.; Nakai, H.; Vreven, T.; Throssell, K.; Montgomery, J. A., Jr.; Peralta, J. E.; Ogliaro, F.; Bearpark, M. J.; Heyd, J. J.; Brothers, E. N.; Kudin, K. N.; Staroverov, V. N.; Keith, T. A.; Kobayashi, R.; Normand, J.; Raghavachari, K.; Rendell, A. P.; Burant, J. C.; Iyengar, S. S.; Tomasi, J.; Cossi, M.; Millam, J. M.; Klene, M.; Adamo, C.; Cammi, R.; Ochterski, J. W.; Martin, R. L.; Morokuma, K.; Farkas, O.; Foresman, J. B.; Fox, D. J. Gaussian, Inc., Wallingford CT, 2016.

(16) Becke, A. D. Density-functional thermochemistry. III. The role of exact exchange. *J. Chem. Phys.* **1993**, *98*, 5648–5652.

(17) Lee, C., Yang, W.; Parr, R. G. Development of the Colle-Salvetti correlation-energy formula into a functional of the electron density. *Phys. Rev. B* **1988**, *37*, 785–789.

(18) Vosko, S. H., Wilk, L.; Nusair, M. Accurate spin-dependent electron liquid correlation energies for local spin density calculations: a critical analysis. *CAM. J. Phys.* **1980**, *58*, 1200–1211.

(19) Stephens, P. J., Devlin, F. J., Chabalowski, C. F.; Frisch, M. J. Ab Initio Calculation of Vibrational Absorption and Circular Dichroism Spectra Using Density Functional Force Fields. *J. Phys. Chem*. **1994**, *98*, 11623–11627.

(20) Ditchfield, R., Hehre W. J.; Pople, J. A. Self-Consistent Molecular-Orbital Methods. IX. An Extended Gaussian-Type Basis for Molecular-Orbital Studies of Organic Molecules. *J. Chem. Phys.* **1971**, *54*, 724–728.

(21) Miertuš, S.; Tomasi, J. Approximate evaluations of the electrostatic free energy and internal energy changes in solution processes. *Chem. Phys.* **1982**, *65*, 239–245.

(22) Miertuš, S., Scrocco, E.; Tomasi, J. Electrostatic interaction of a solute with a continuum. A direct utilization of AB initio molecular potentials for the prevision of solvent effects. *Chem. Phys.* **1981**, *55*, 117–129.

(23) Pascual-Ahuir, L., Silla, E.; Tuñón, I. GEPOL: An improved description of molecular surfaces. III. A new algorithm for the computation of a solvent-excluding surface. *J. Comp. Chem.* **1994**, *15*, 1127–1138.

(24) Grimme, S., Ehrlich, S.; Goerigk, L. Effect of the damping function in dispersion corrected density functional theory. *J. Comp. Chem.* **2011**, *32*, 1456–1465.

(25) Legault, C.: CylView. http://www.cylview.org.

8 NMR Spectra

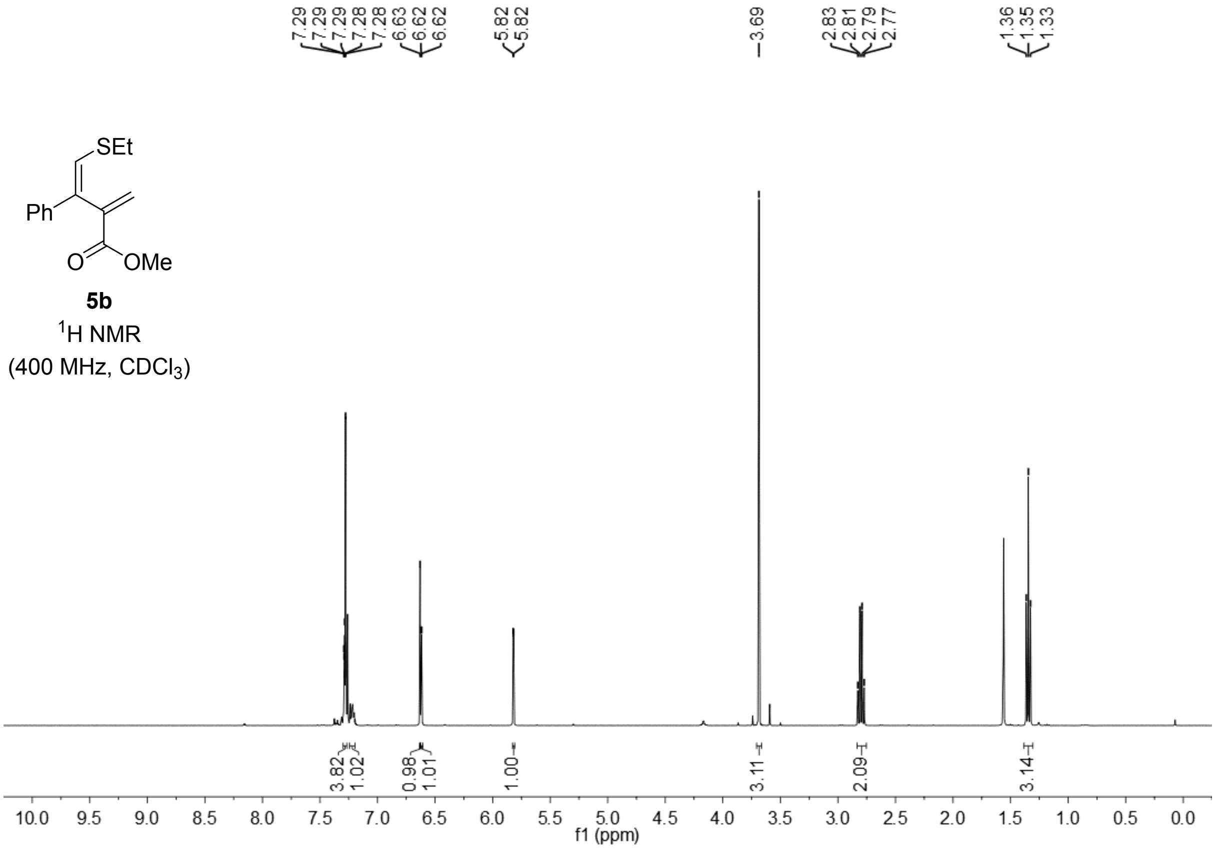

5b
^{1}H NMR
(400 MHz, $CDCl_3$)

5b
^{13}C NMR
(101 MHz, $CDCl_3$)

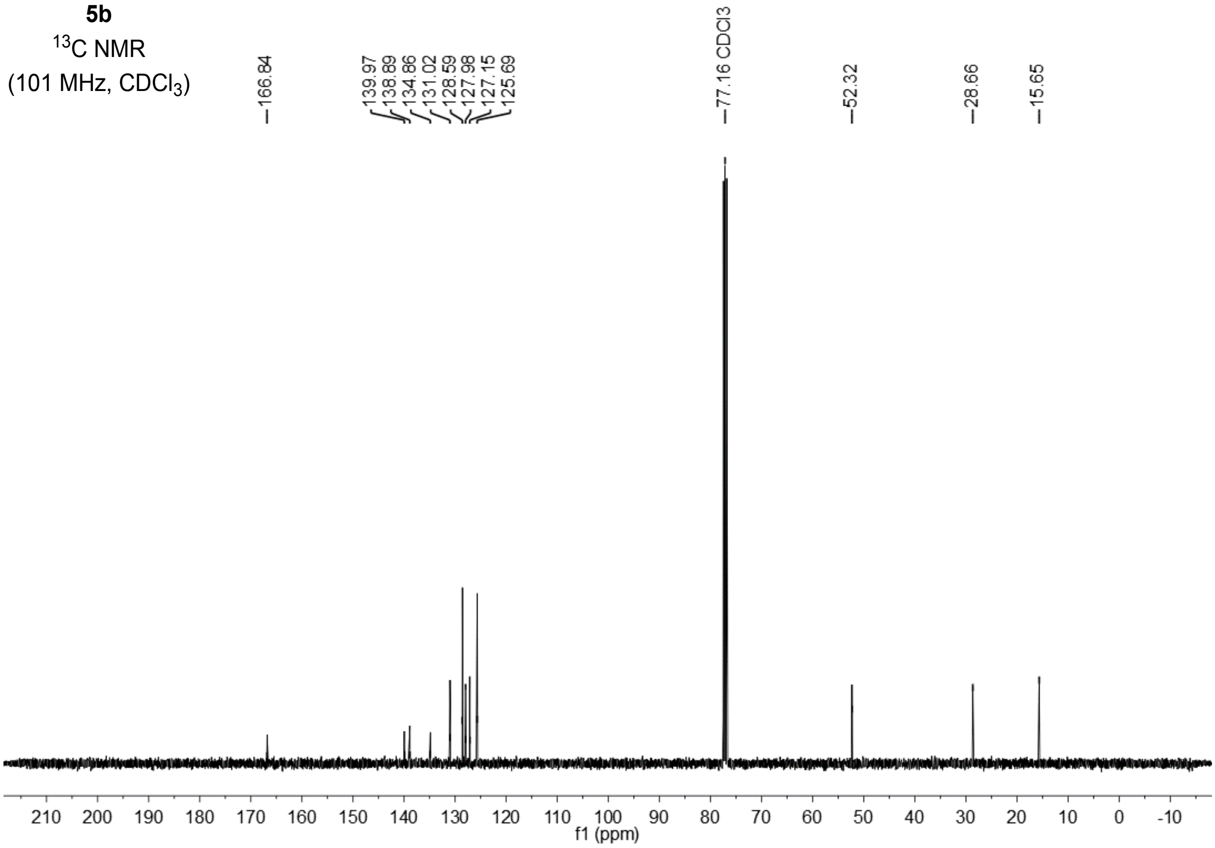

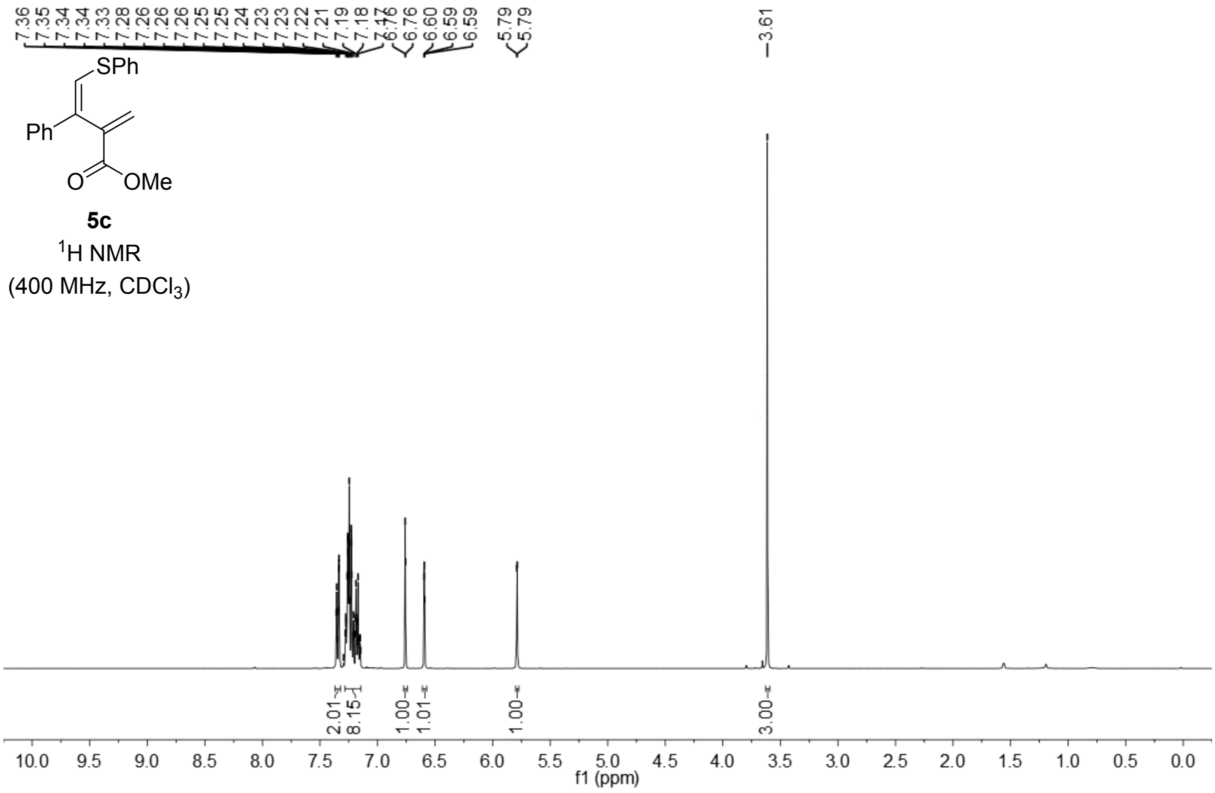

5c
^{1}H NMR
(400 MHz, $CDCl_3$)

5c
^{13}C NMR
(101 MHz, $CDCl_3$)

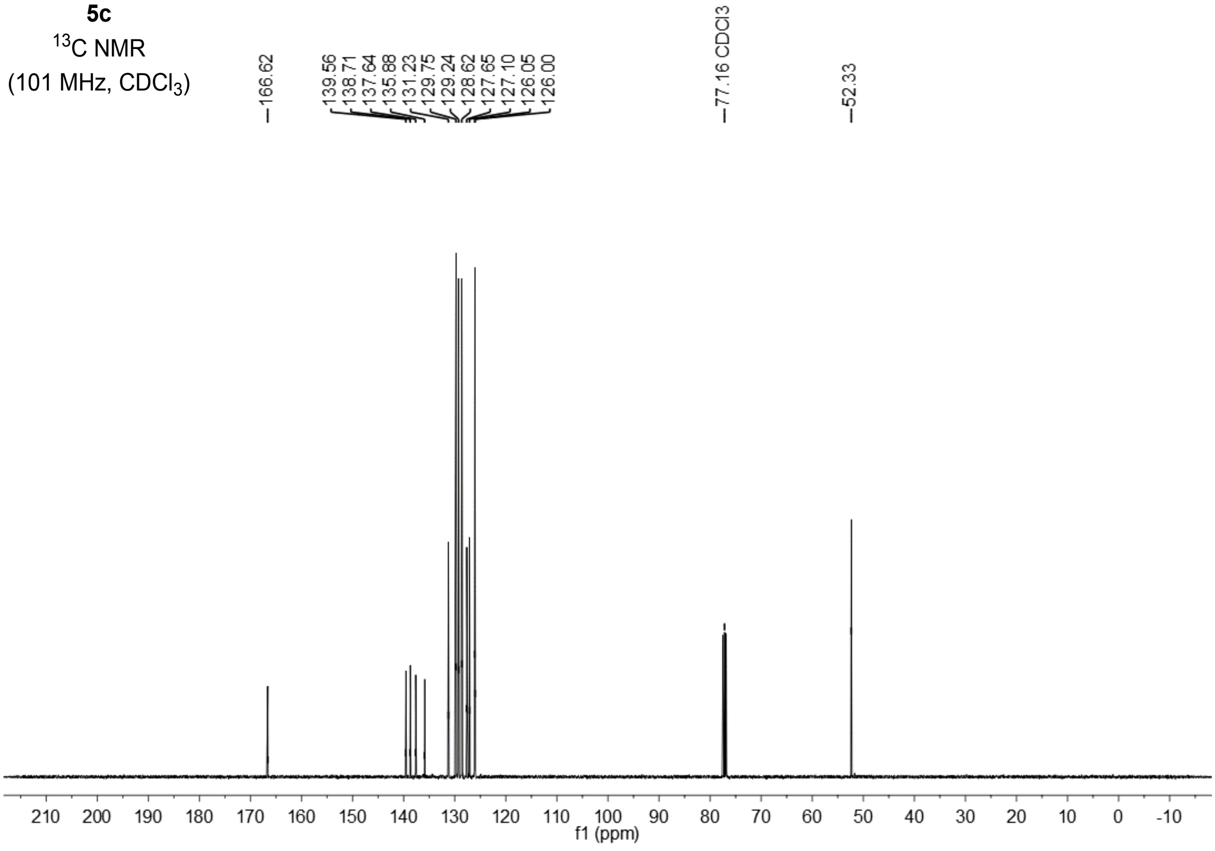

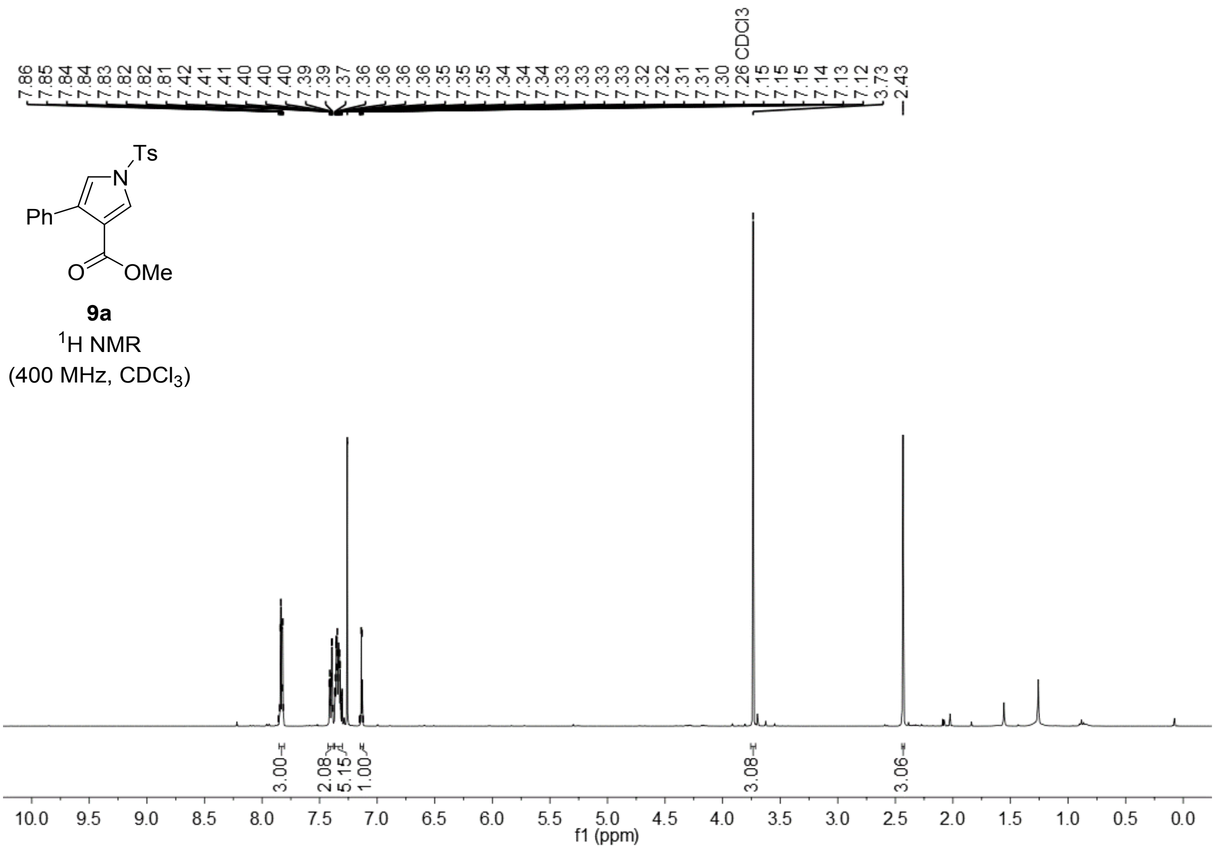
Ts
N
Ph
O
OMe
9a
^{1}H NMR
(400 MHz, $CDCl_3$)
7.26 CDCl3
3.73
2.43
3.00
2.08
5.15
1.00
3.08
3.06
f1 (ppm)

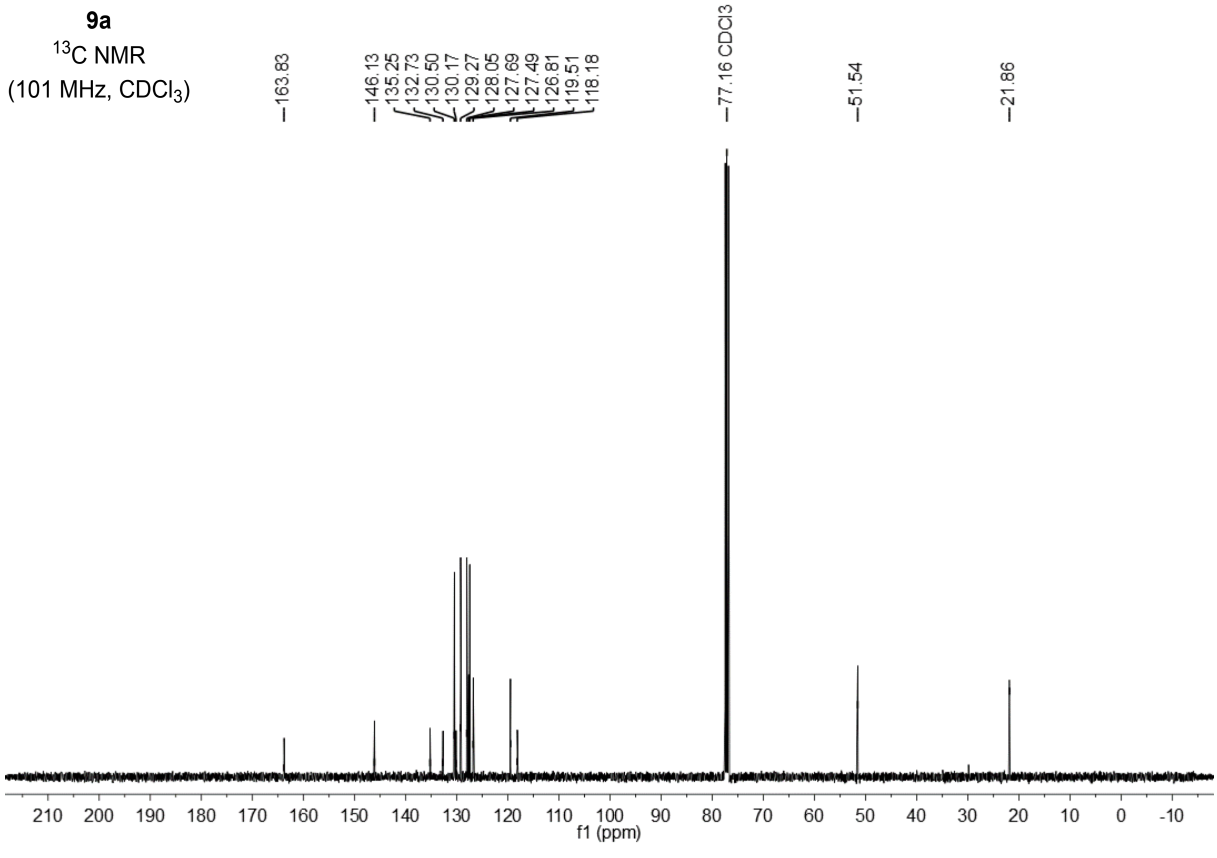
9a
^{13}C NMR
(101 MHz, $CDCl_3$)
163.83
146.13
135.25
132.73
130.50
130.17
129.27
128.05
127.69
127.49
126.81
119.51
118.18
77.16 CDCl3
51.54
21.86
f1 (ppm)

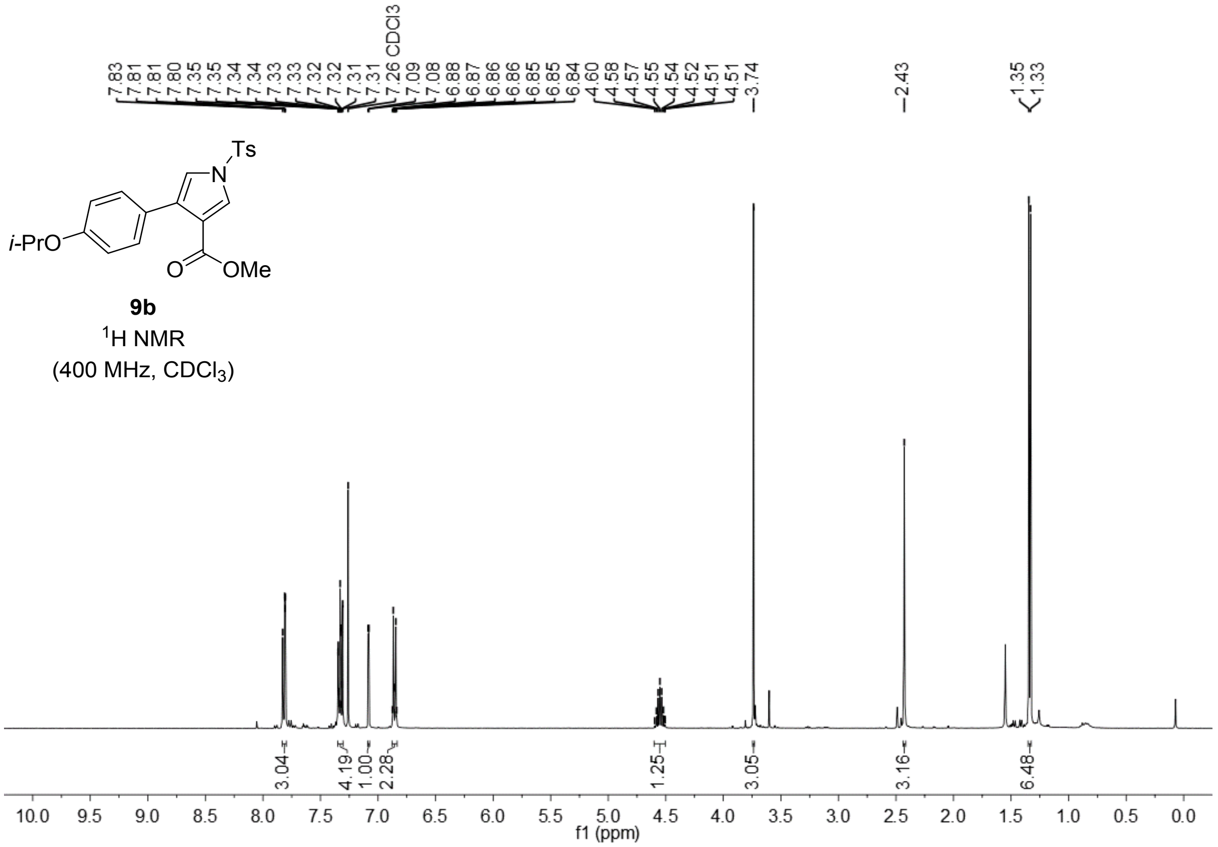

9b
^{1}H NMR
(400 MHz, $CDCl_3$)

9b
^{13}C NMR
(101 MHz, $CDCl_3$)

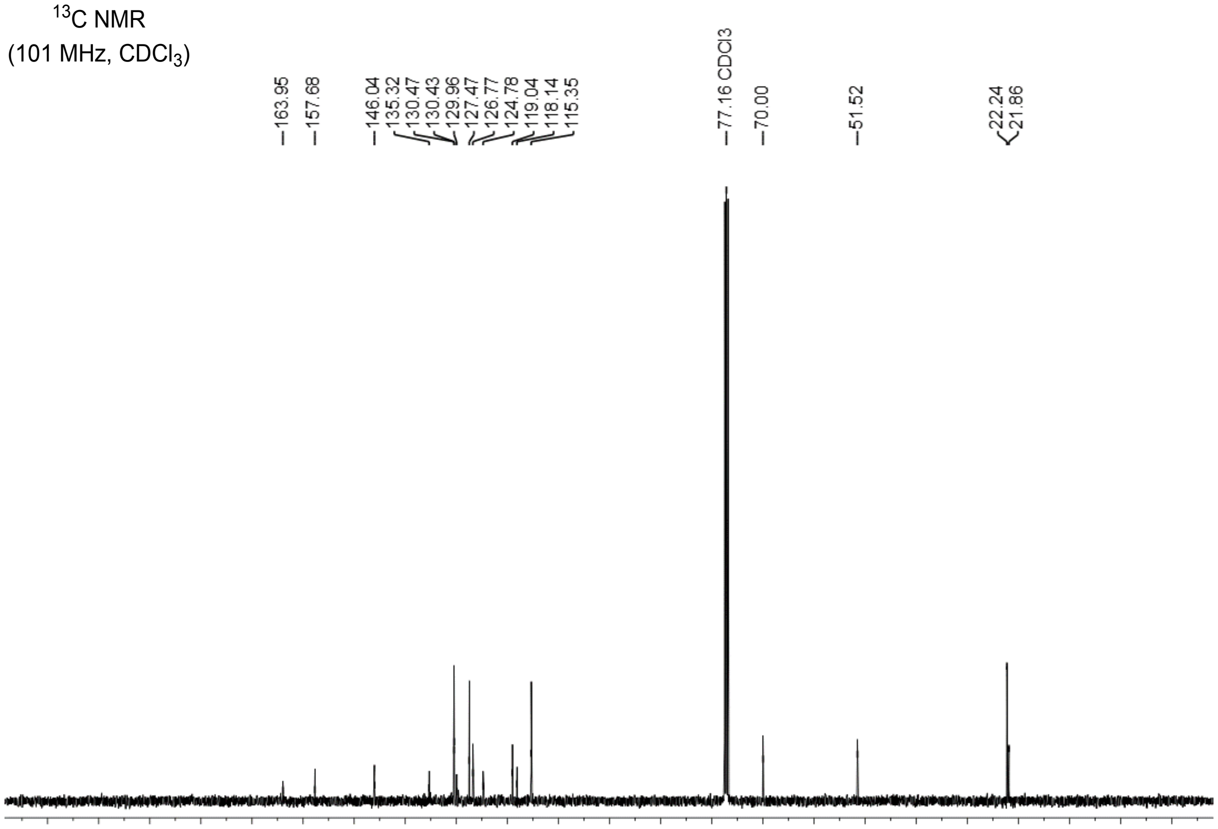

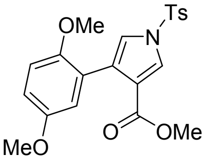

9c
^{1}H NMR
(400 MHz, $CDCl_3$)

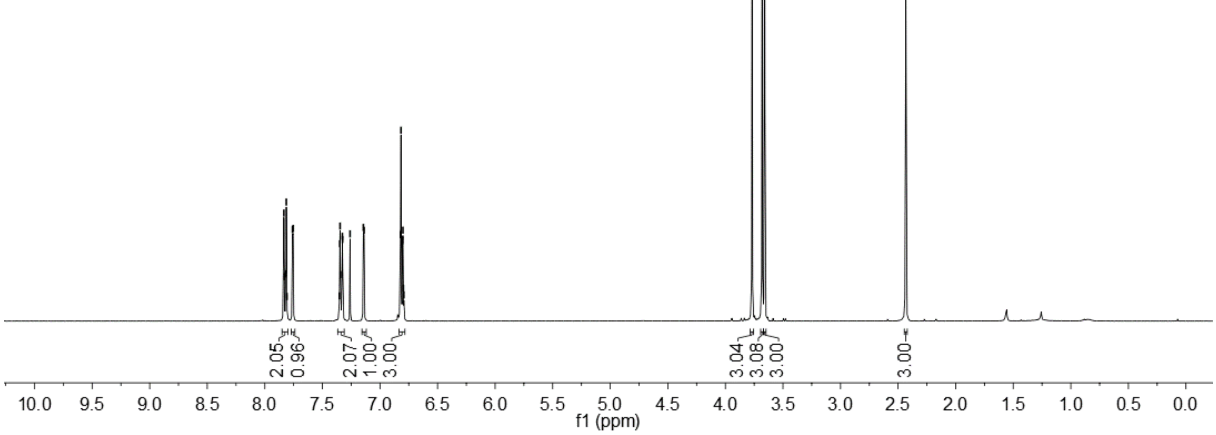

9c
^{13}C NMR
(101 MHz, $CDCl_3$)

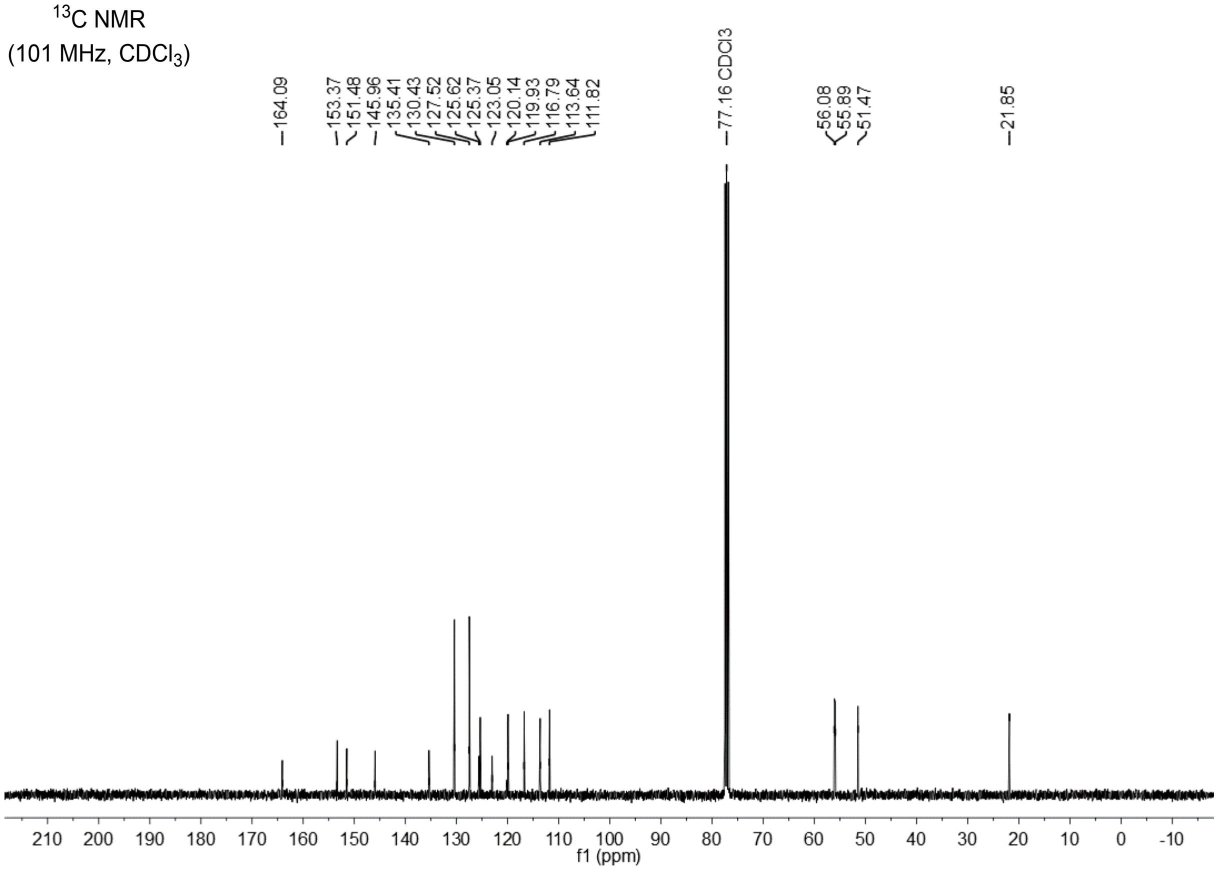

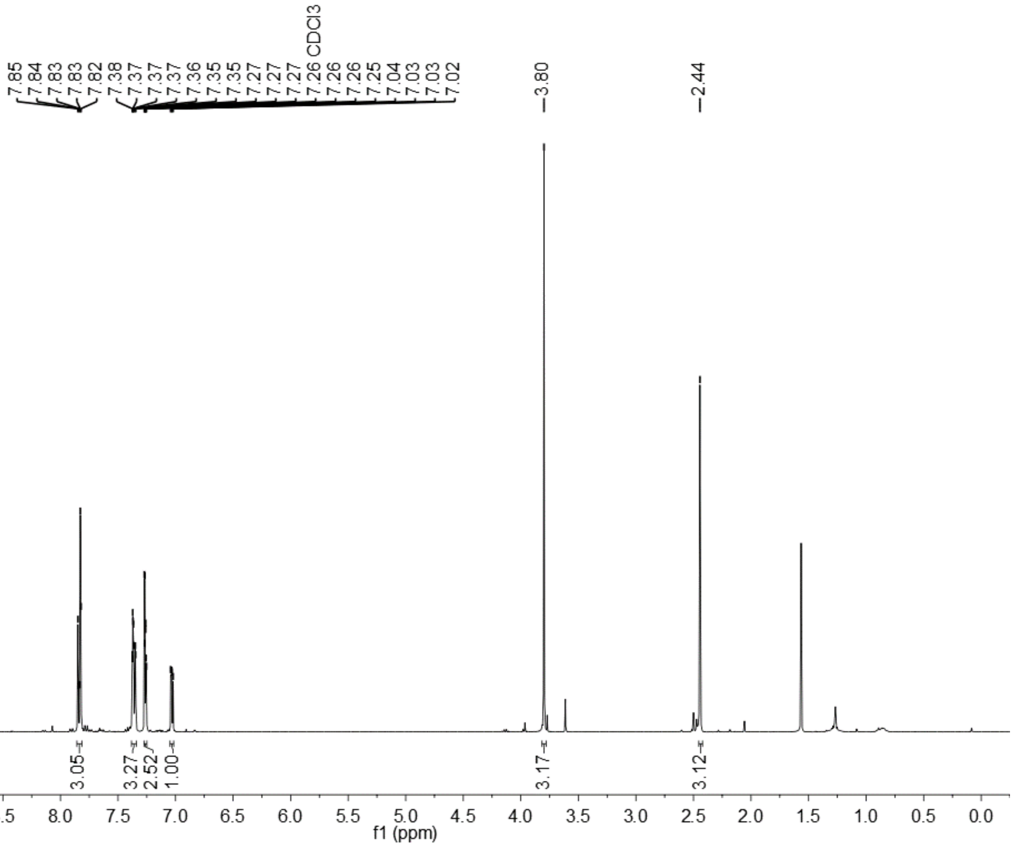

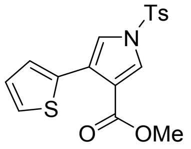

9d
^{1}H NMR
(400 MHz, $CDCl_3$)

9d
^{13}C NMR
(101 MHz, $CDCl_3$)

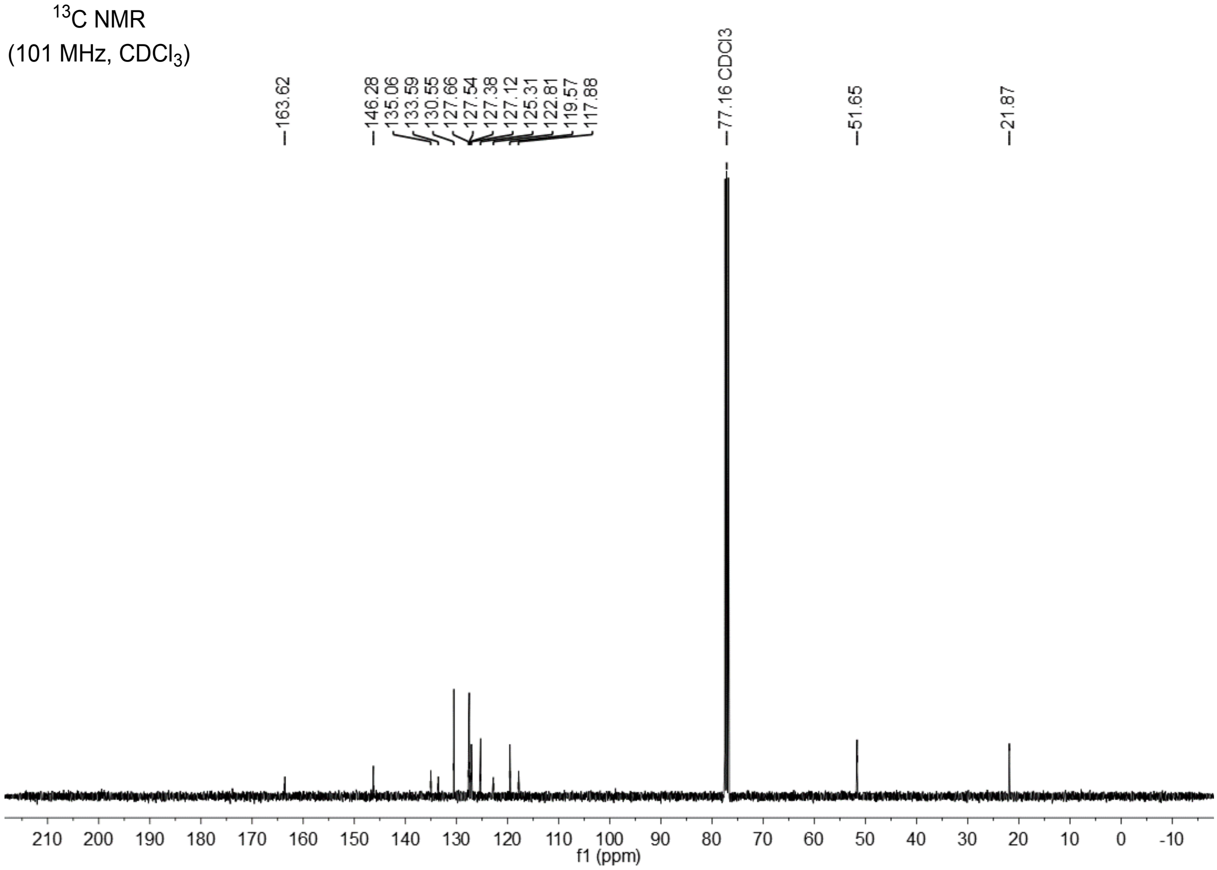

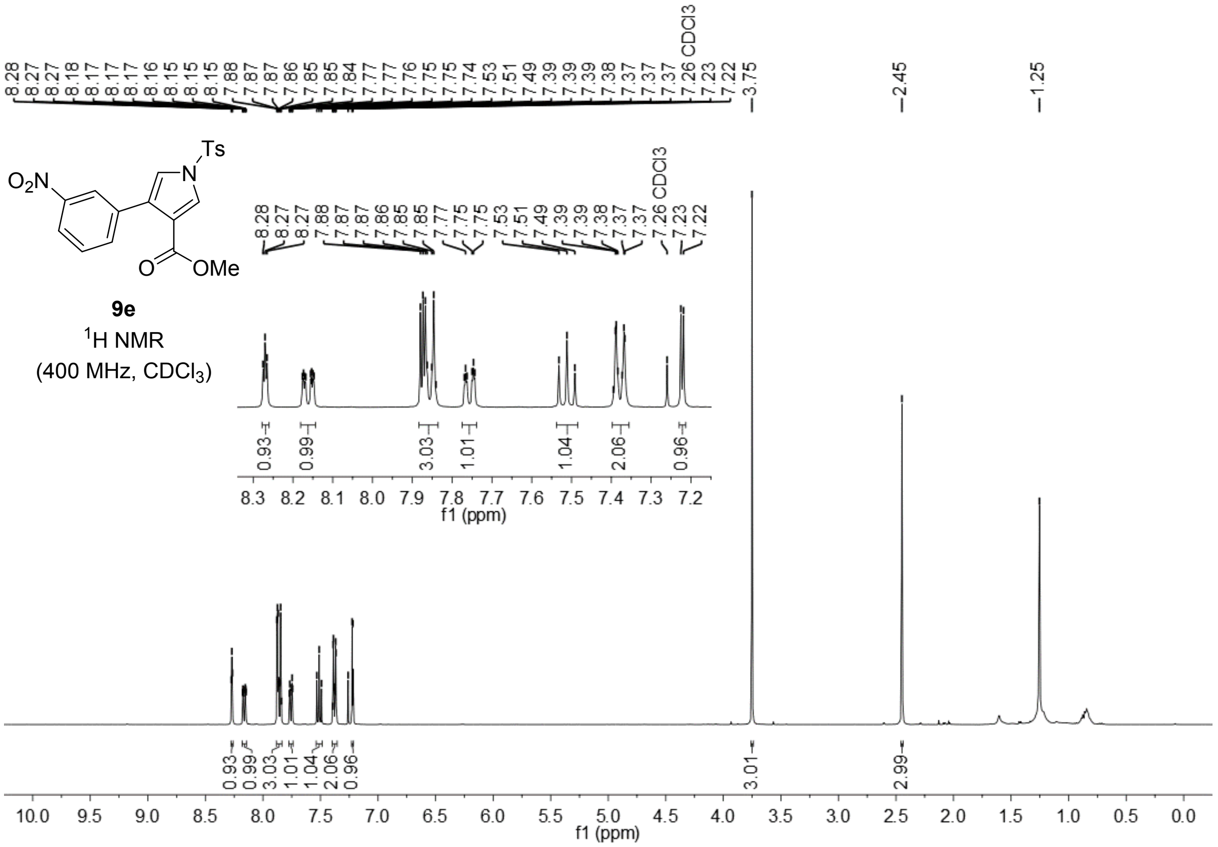

9e
^{1}H NMR
(400 MHz, $CDCl_3$)

9e
^{13}C NMR
(101 MHz, $CDCl_3$)

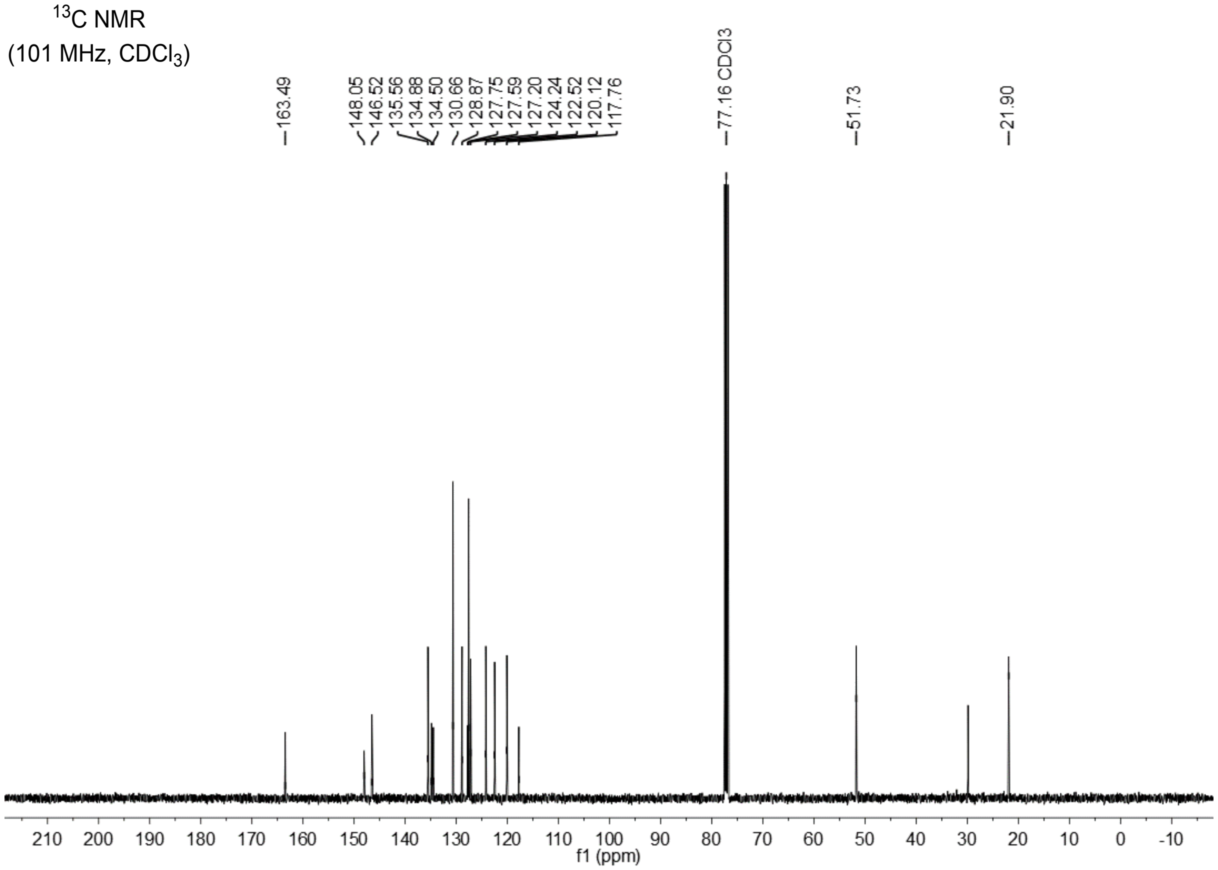

9f
^{1}H NMR
(400 MHz, $CDCl_3$)

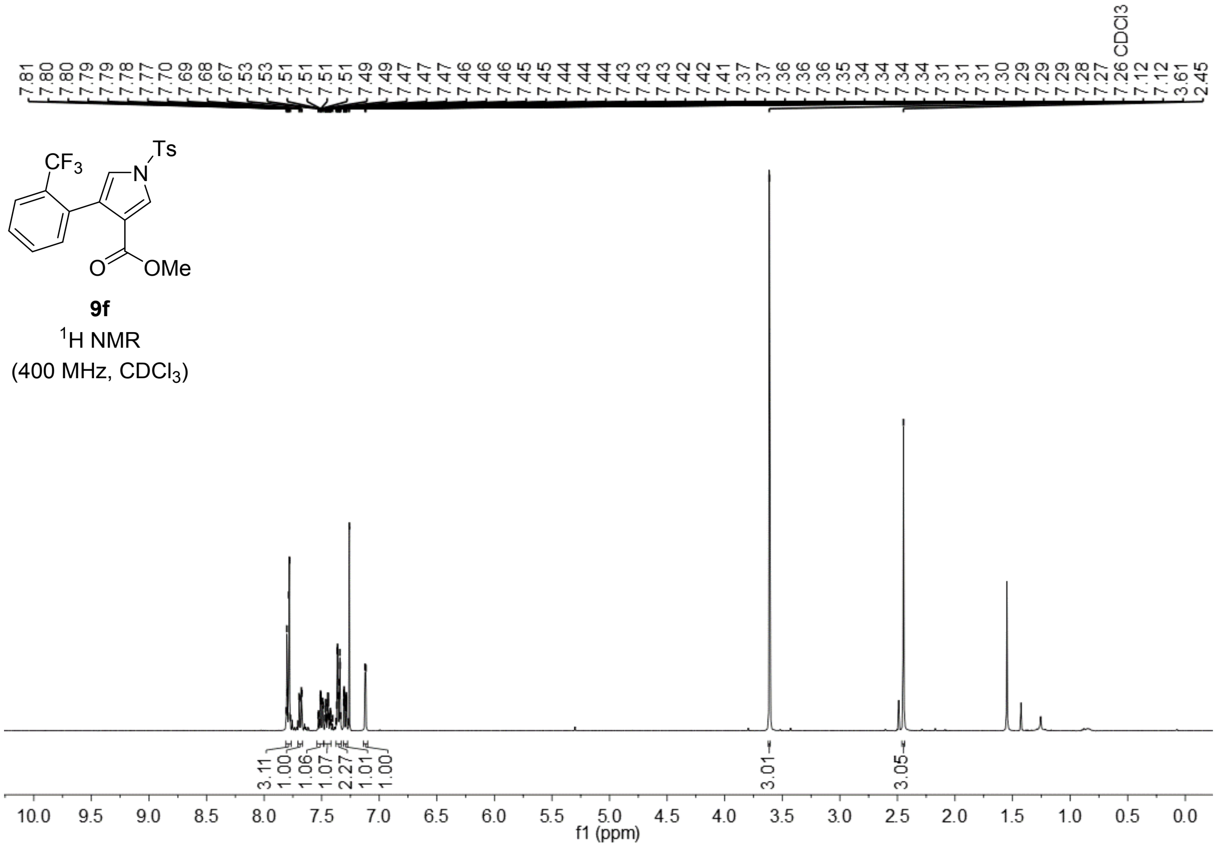

9f
^{13}C NMR
(101 MHz, $CDCl_3$)

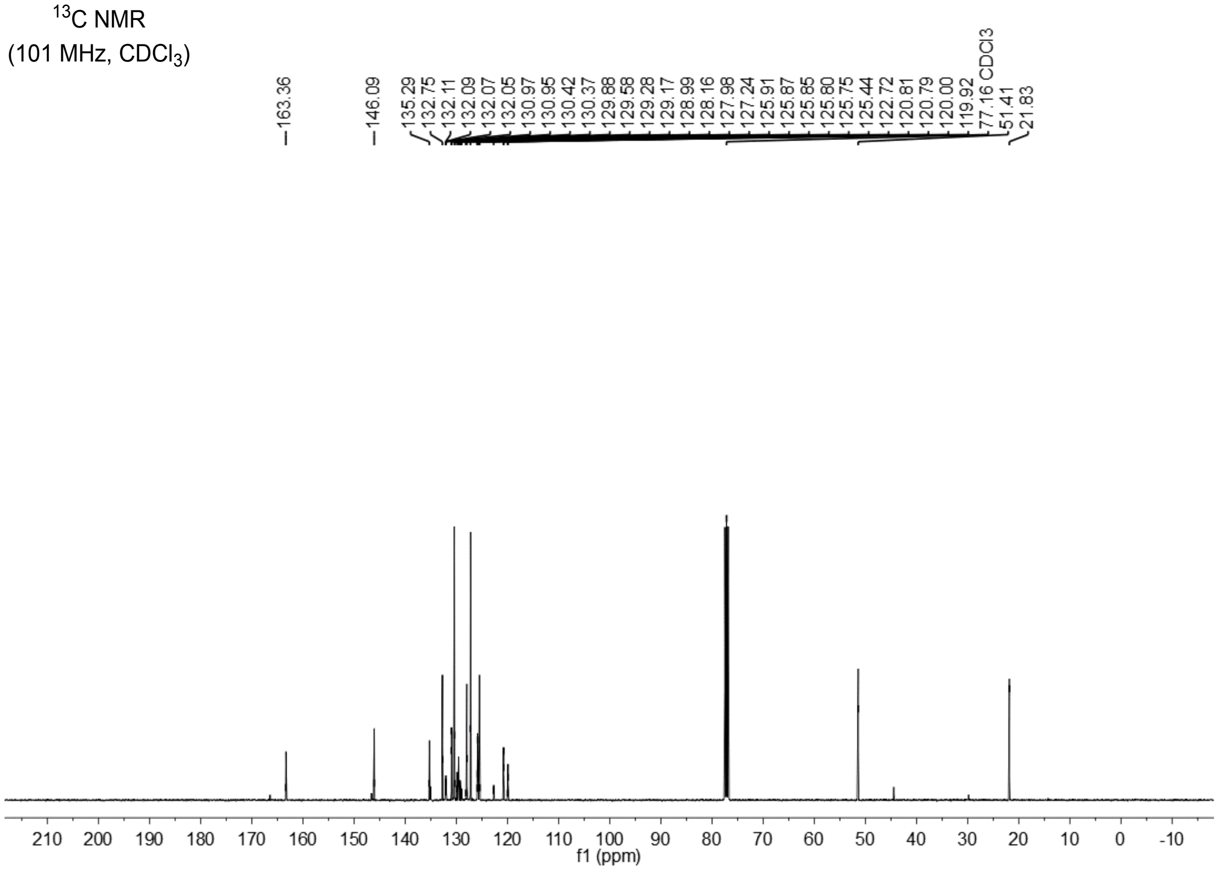

9f
^{19}F NMR
(376 MHz, $CDCl_3$)

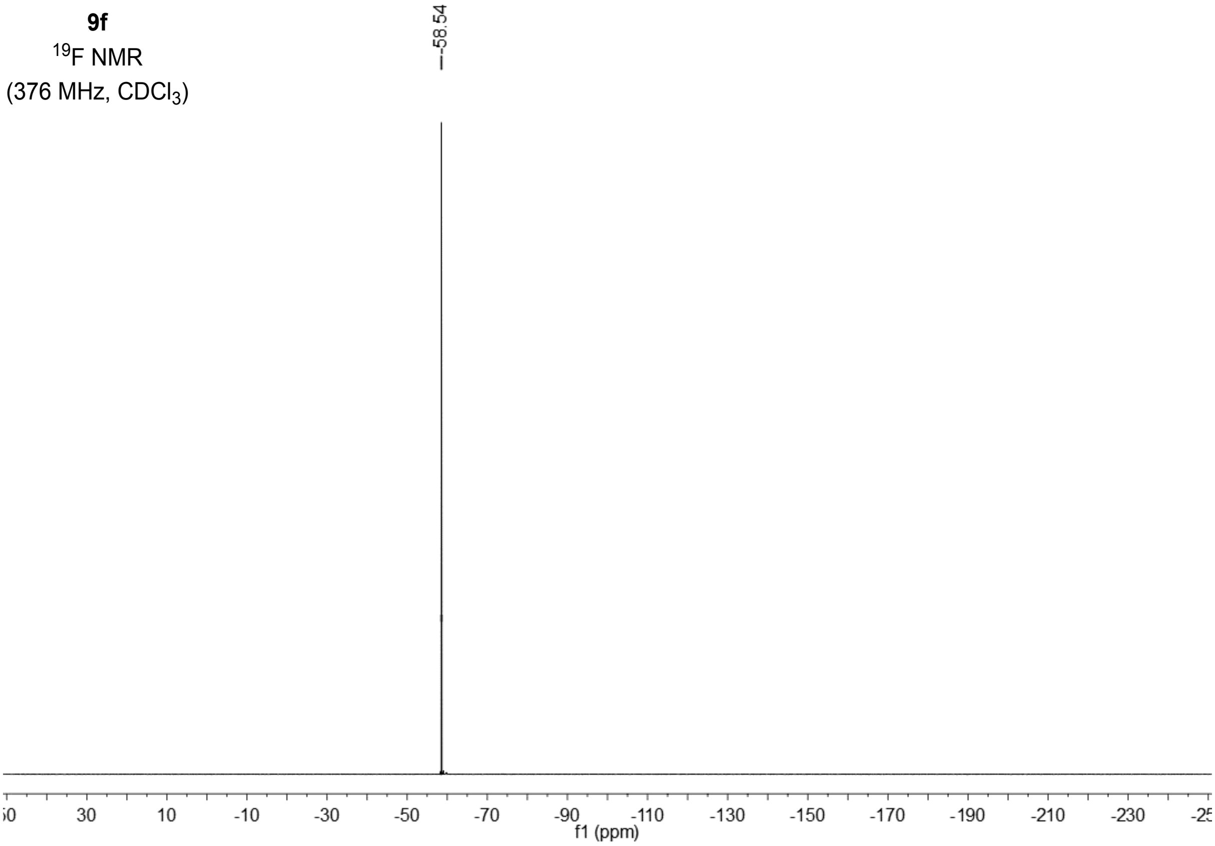

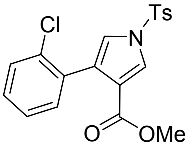

9g
^{1}H NMR
(400 MHz, $CDCl_3$)

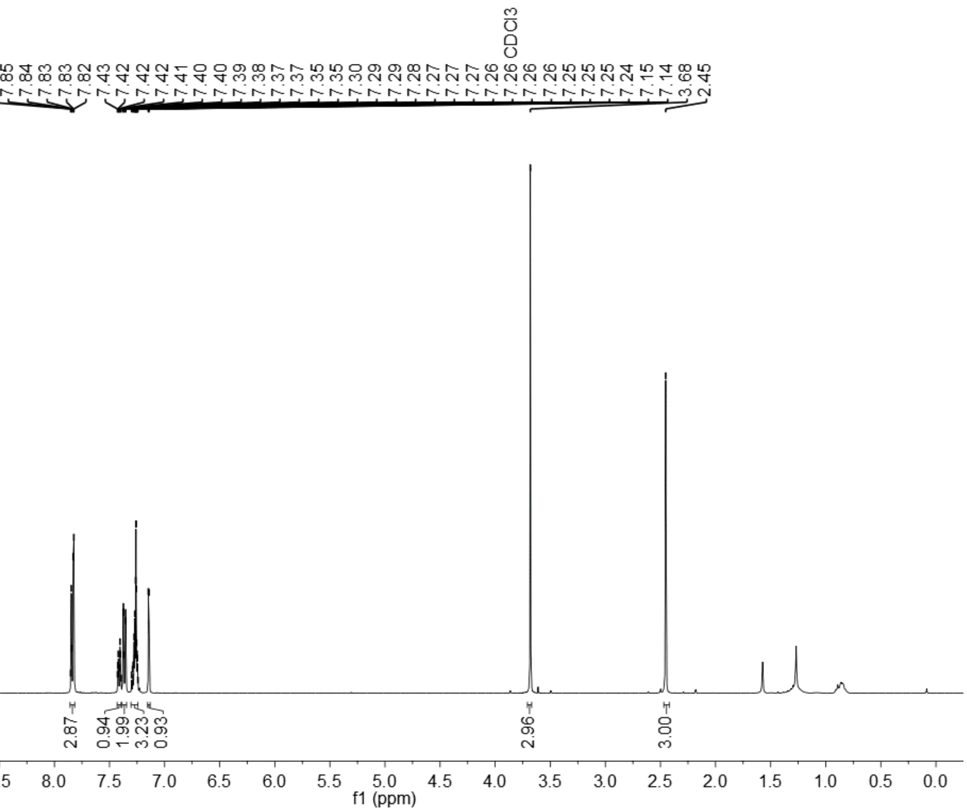

9g
^{13}C NMR
(101 MHz, $CDCl_3$)

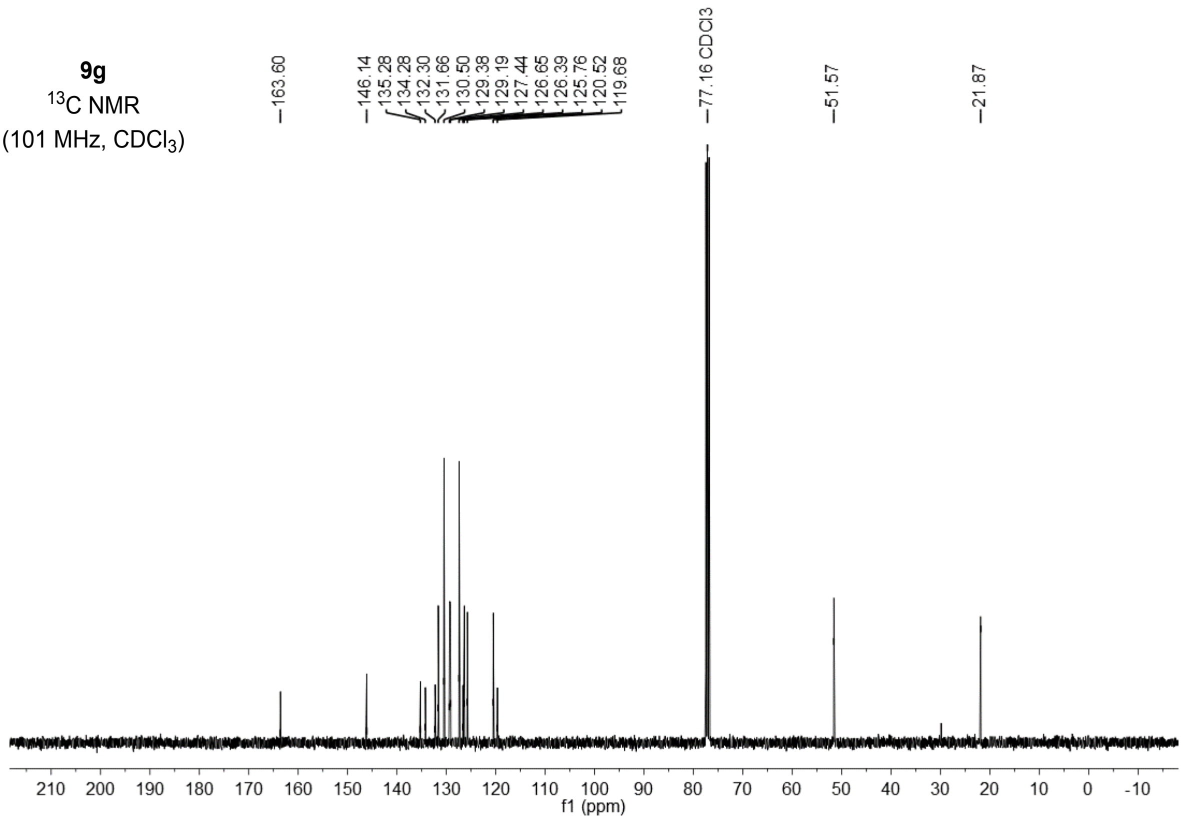

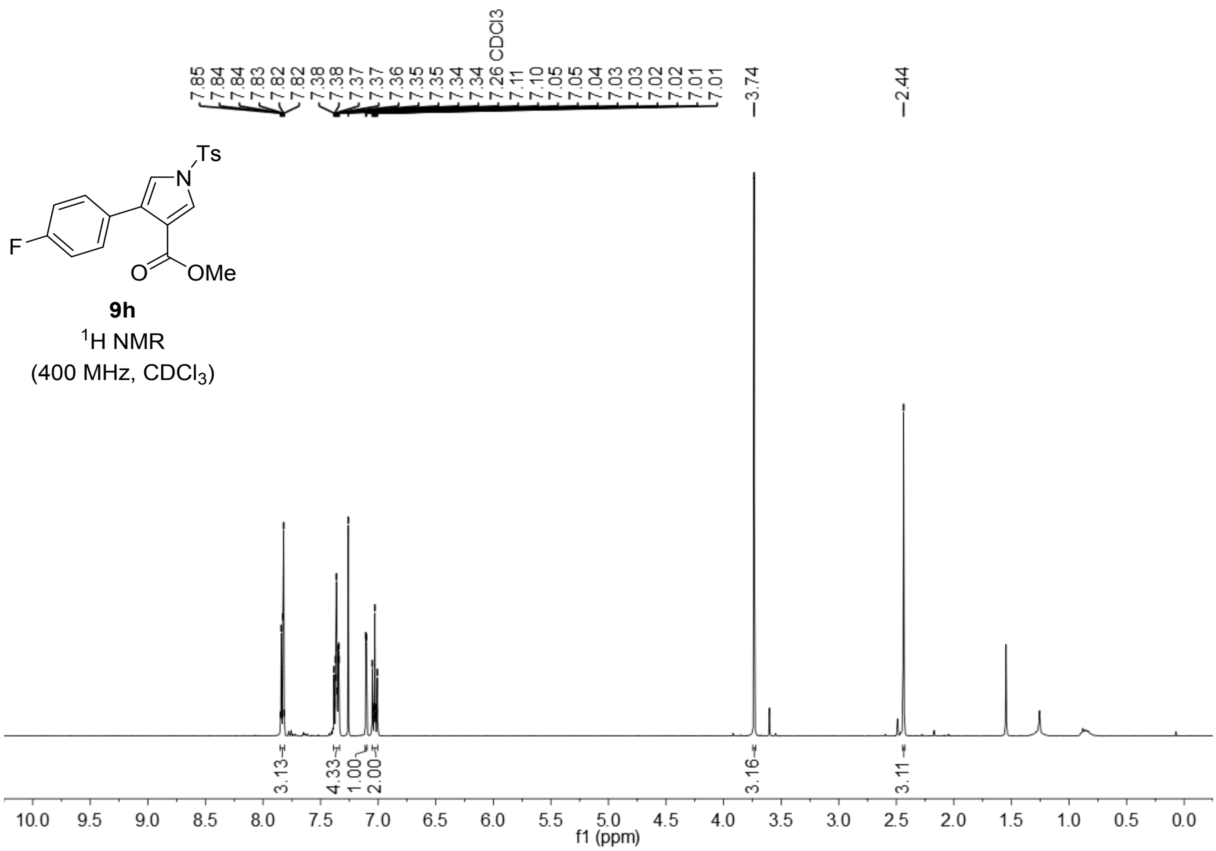

9h
^{1}H NMR
(400 MHz, $CDCl_3$)

9h
^{13}C NMR
(101 MHz, $CDCl_3$)

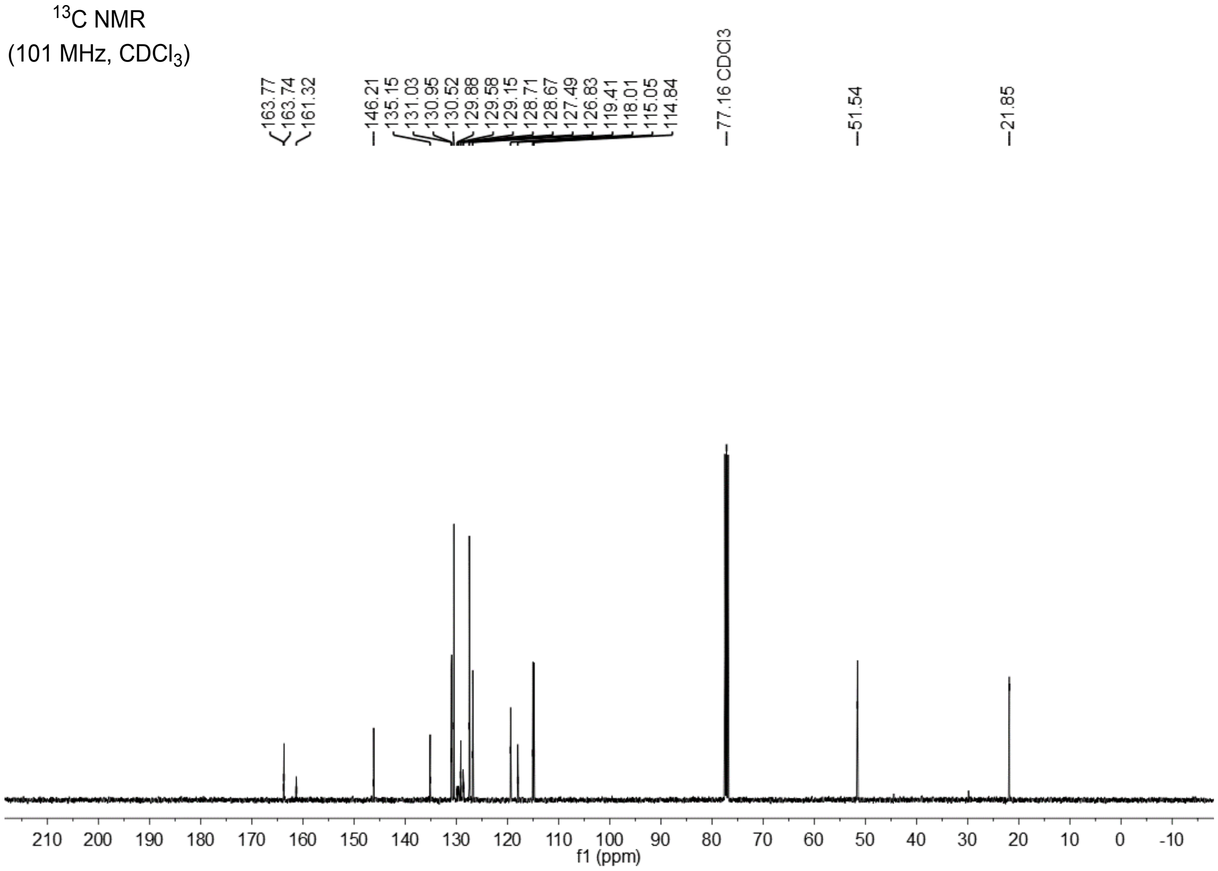

9h
^{19}F NMR
(376 MHz, $CDCl_3$)

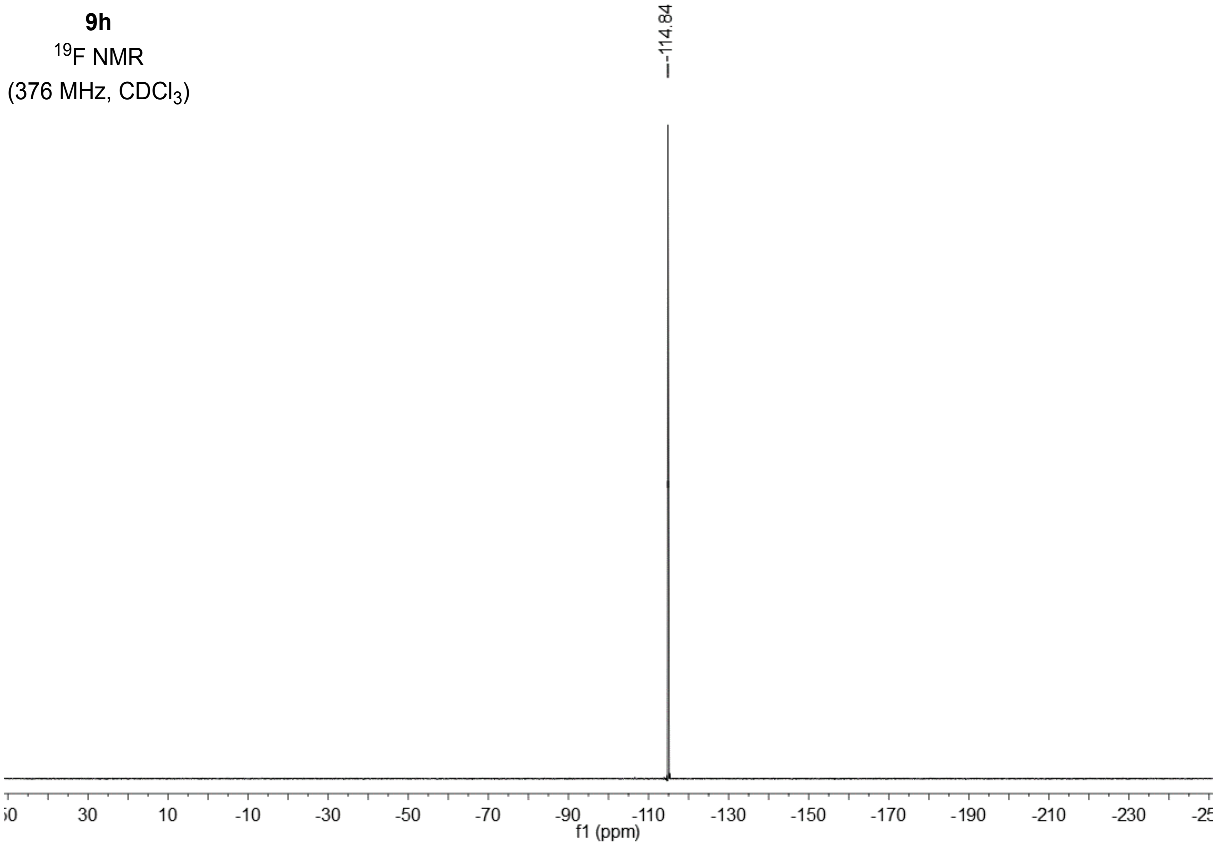

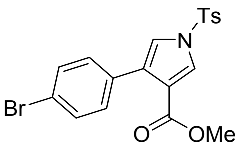

9i
^{1}H NMR
(400 MHz, $CDCl_3$)

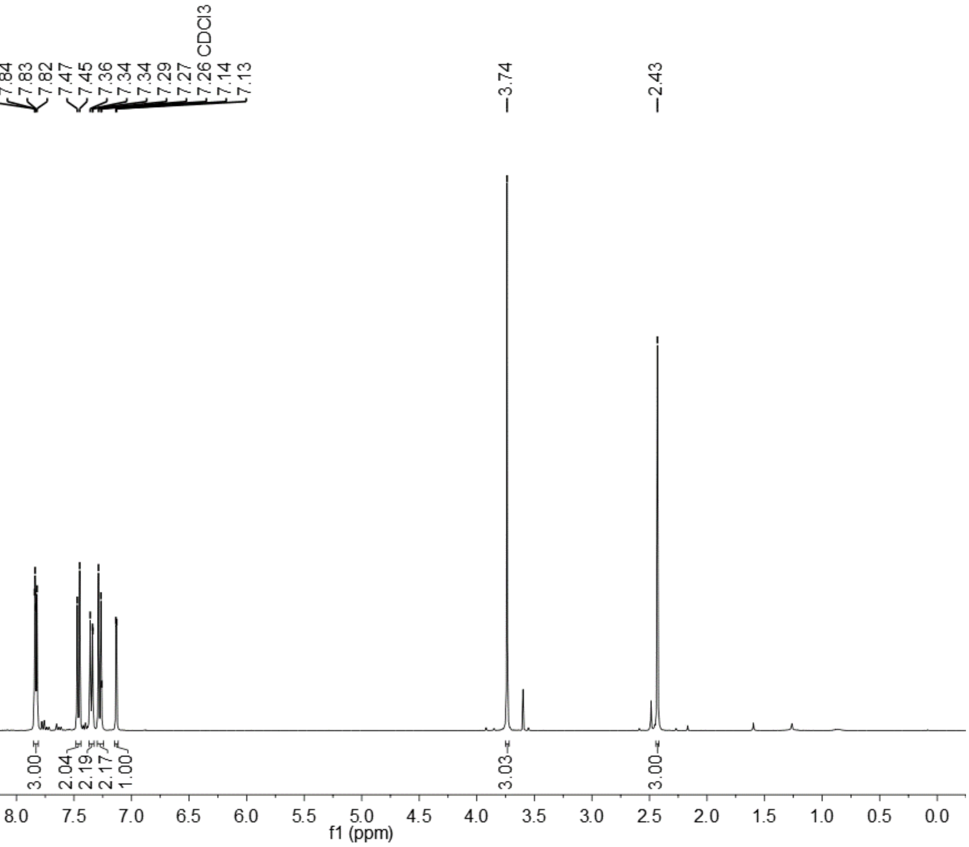

9i
^{13}C NMR
(101 MHz, $CDCl_3$)

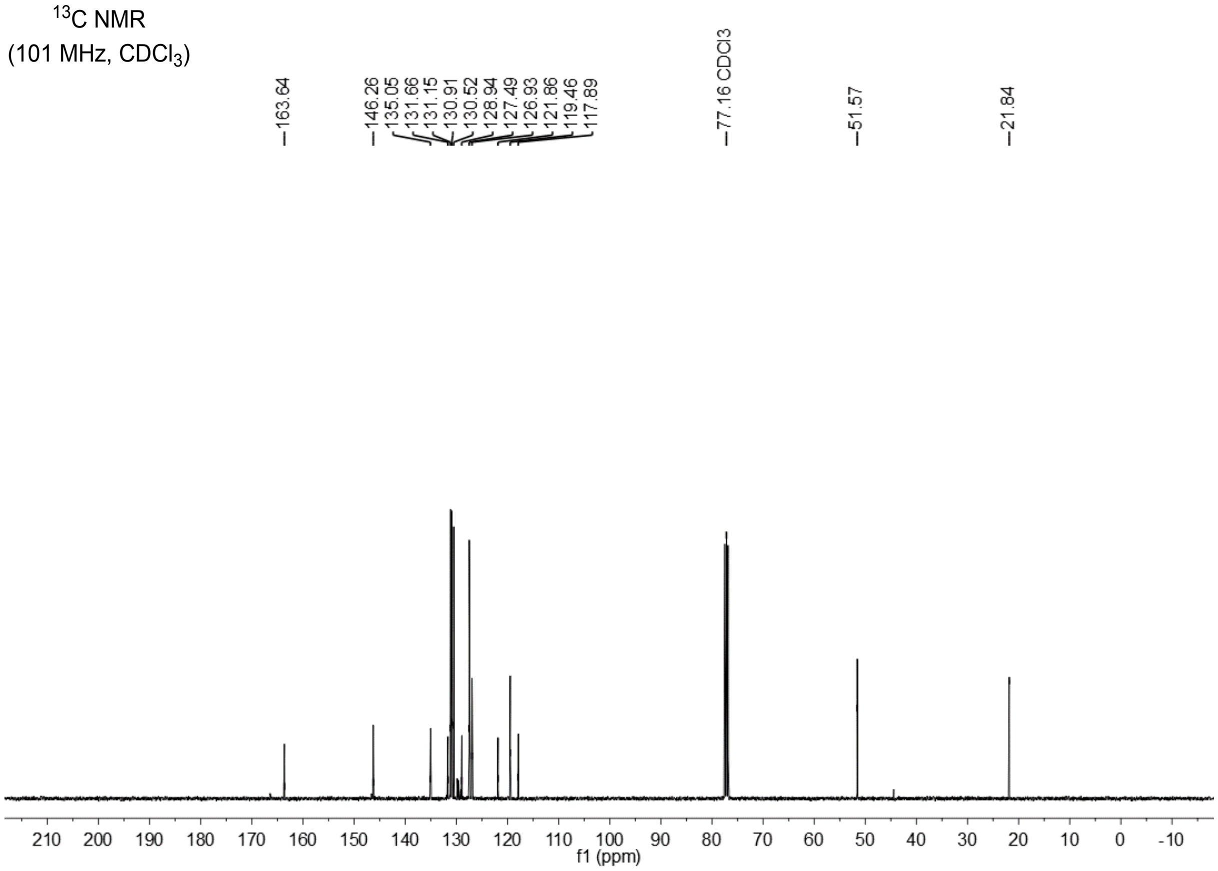

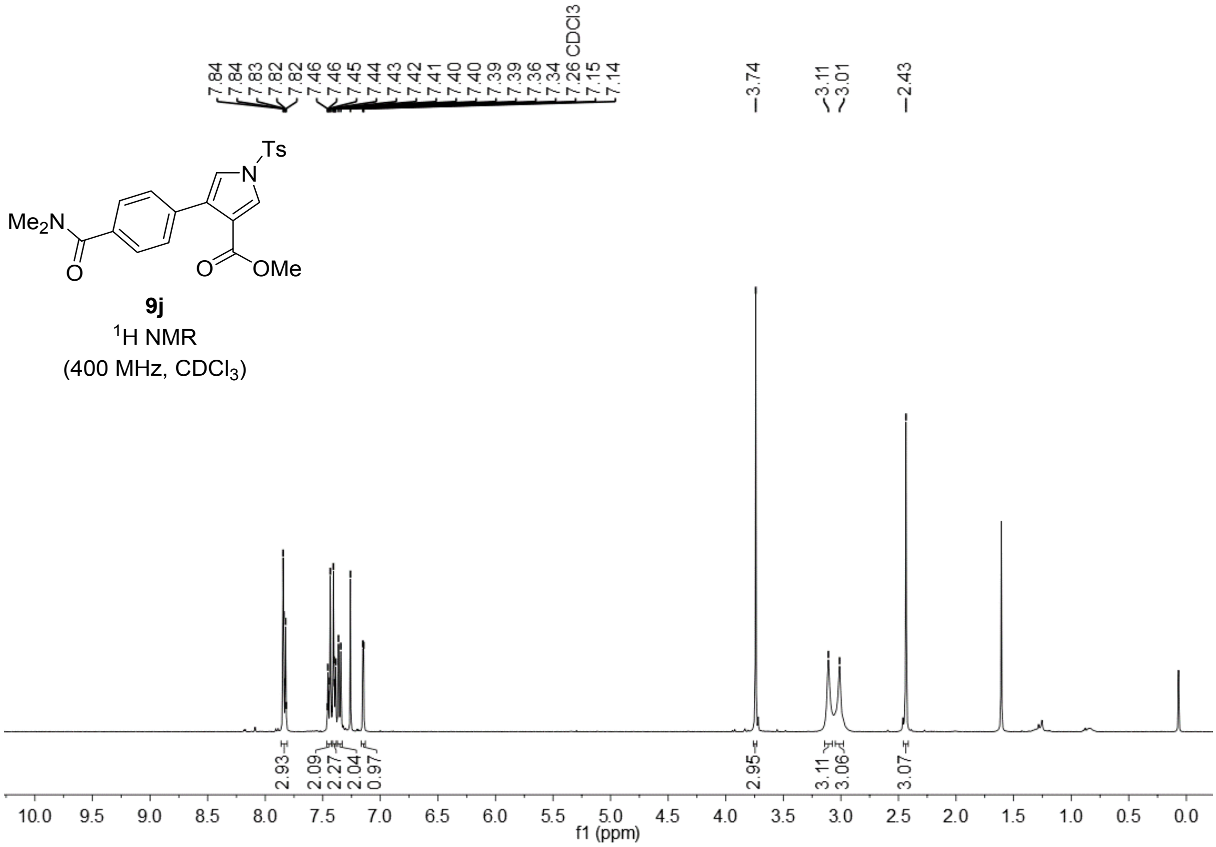

9j
^1H NMR
(400 MHz, $CDCl_3$)

9j
^{13}C NMR
(101 MHz, $CDCl_3$)

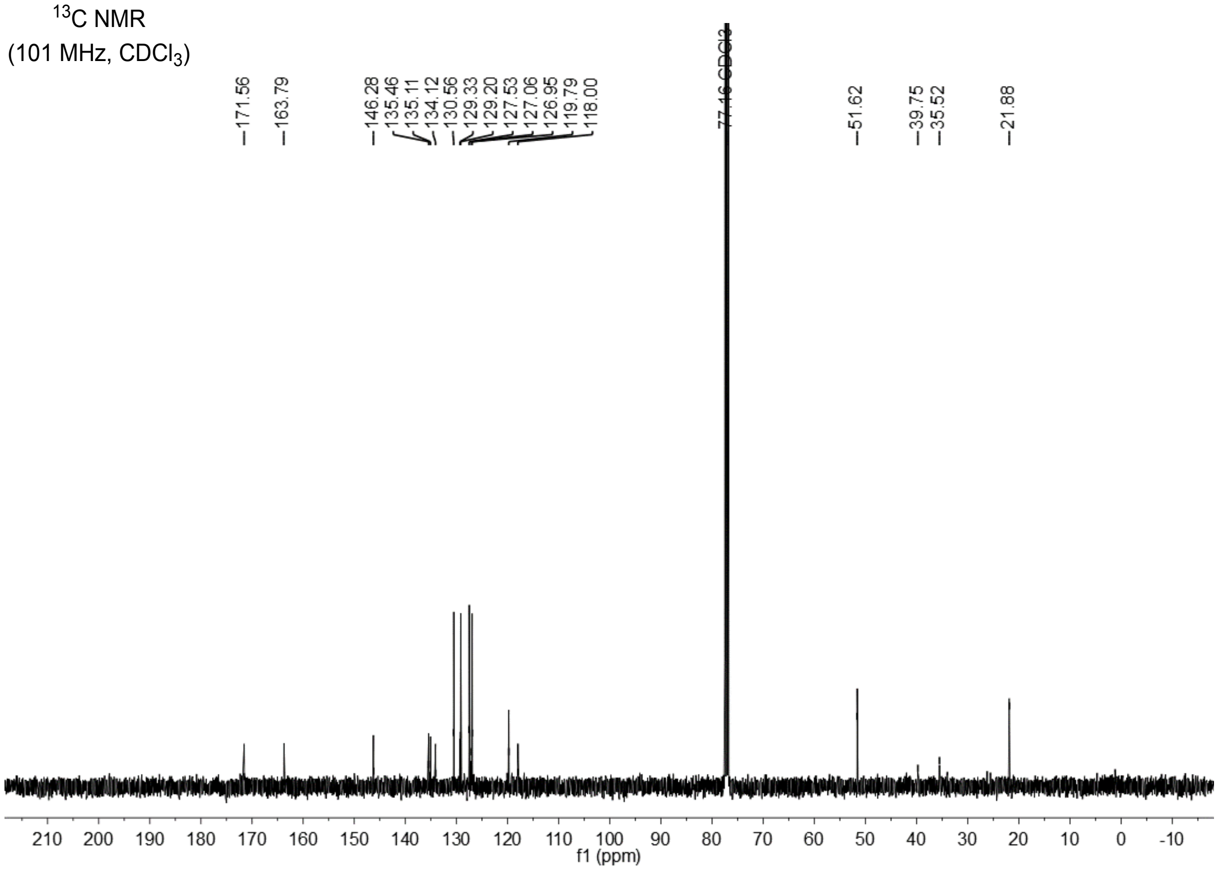

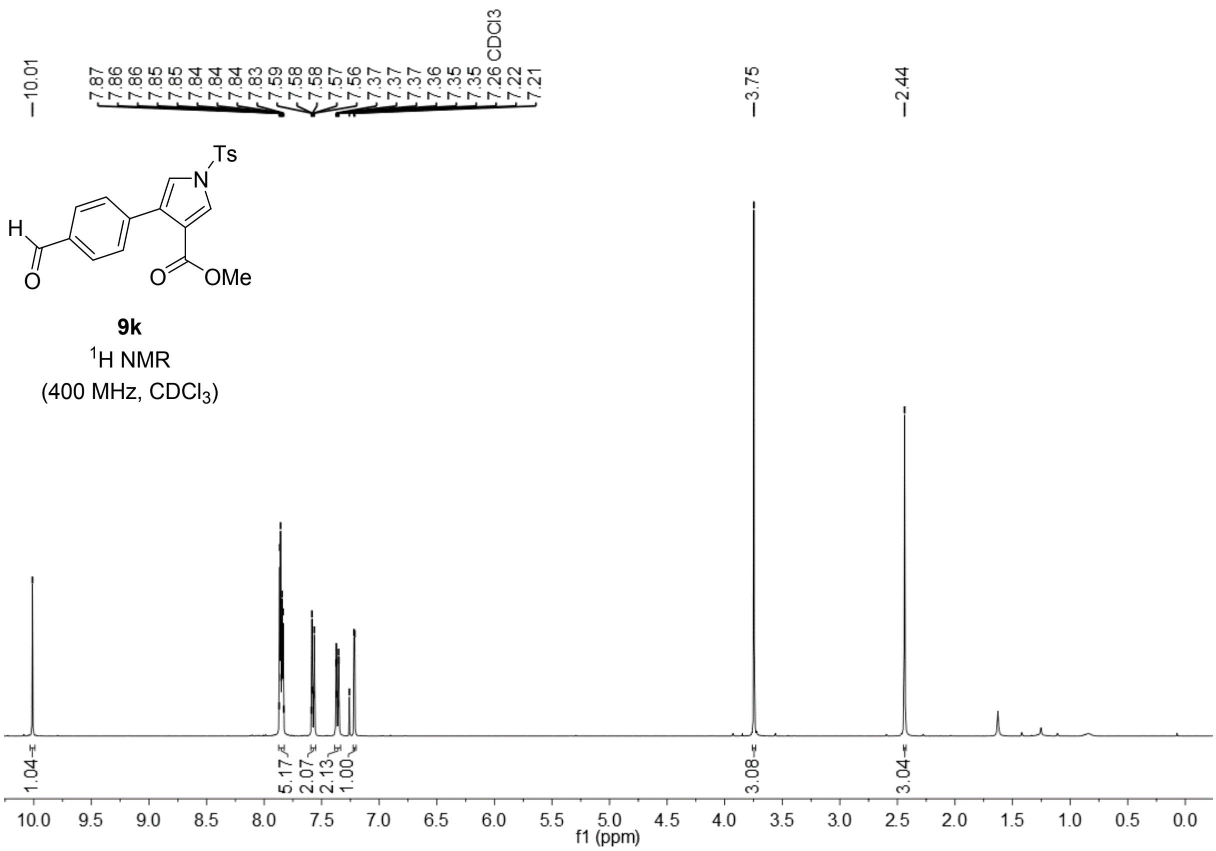

9k
^{1}H NMR
(400 MHz, $CDCl_3$)

9k
^{13}C NMR
(101 MHz, $CDCl_3$)

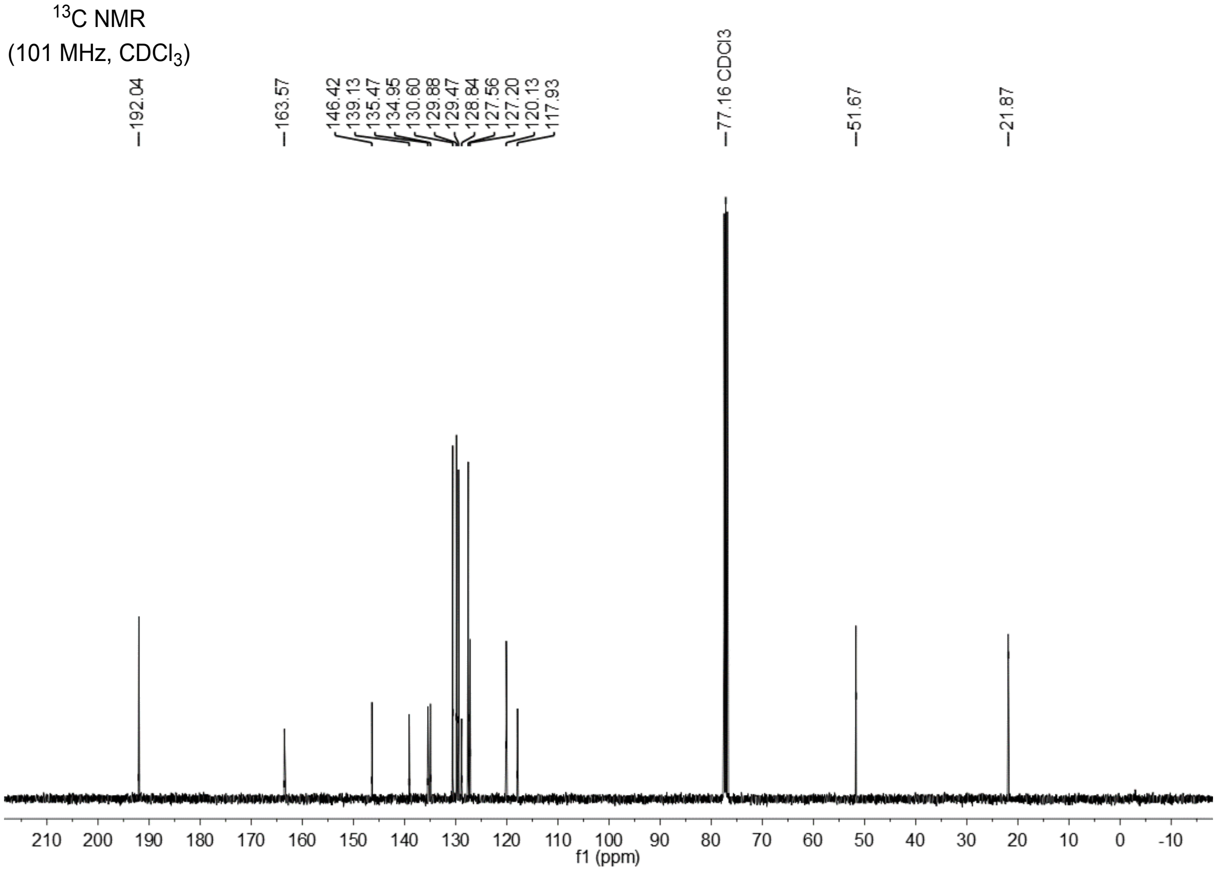

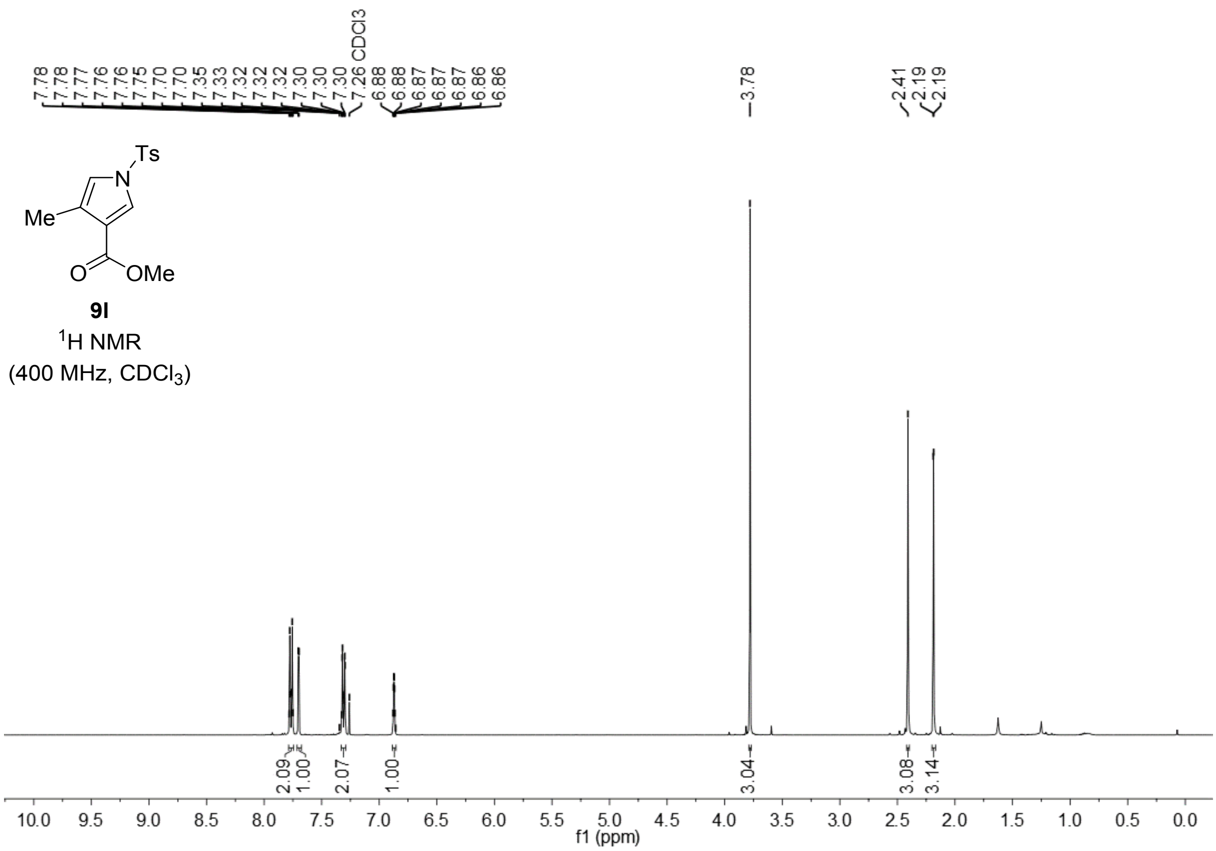

9l
^{1}H NMR
(400 MHz, $CDCl_3$)

9l
^{13}C NMR
(101 MHz, $CDCl_3$)

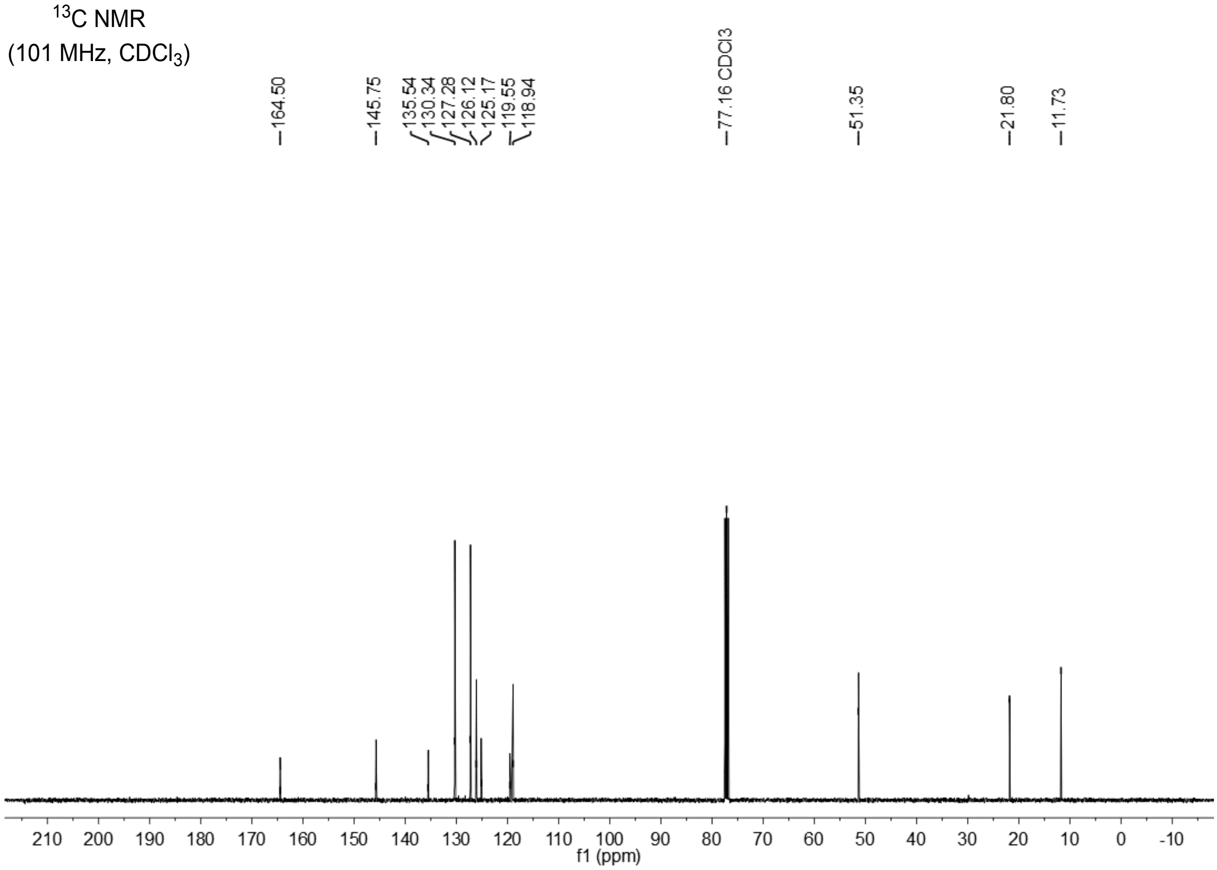

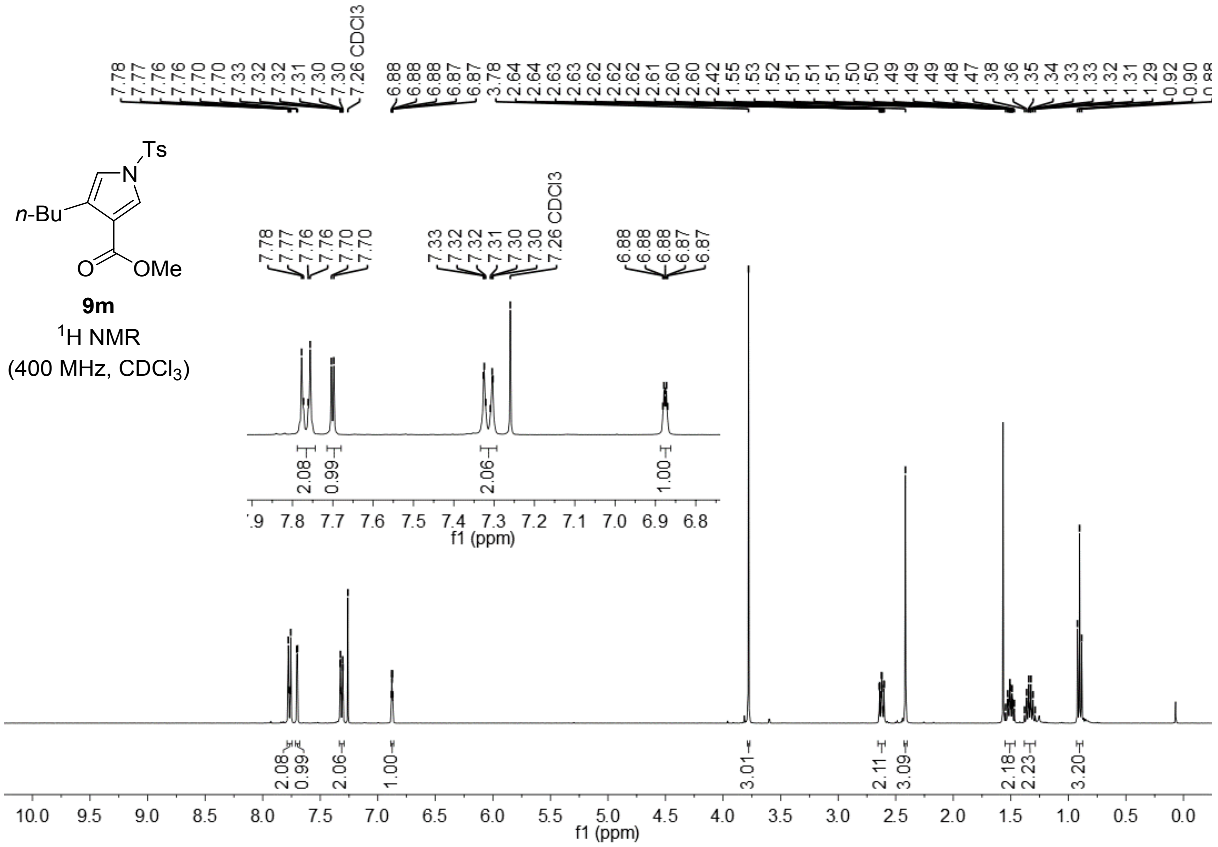

9m
^{1}H NMR
(400 MHz, $CDCl_3$)

9m
^{13}C NMR
(101 MHz, $CDCl_3$)

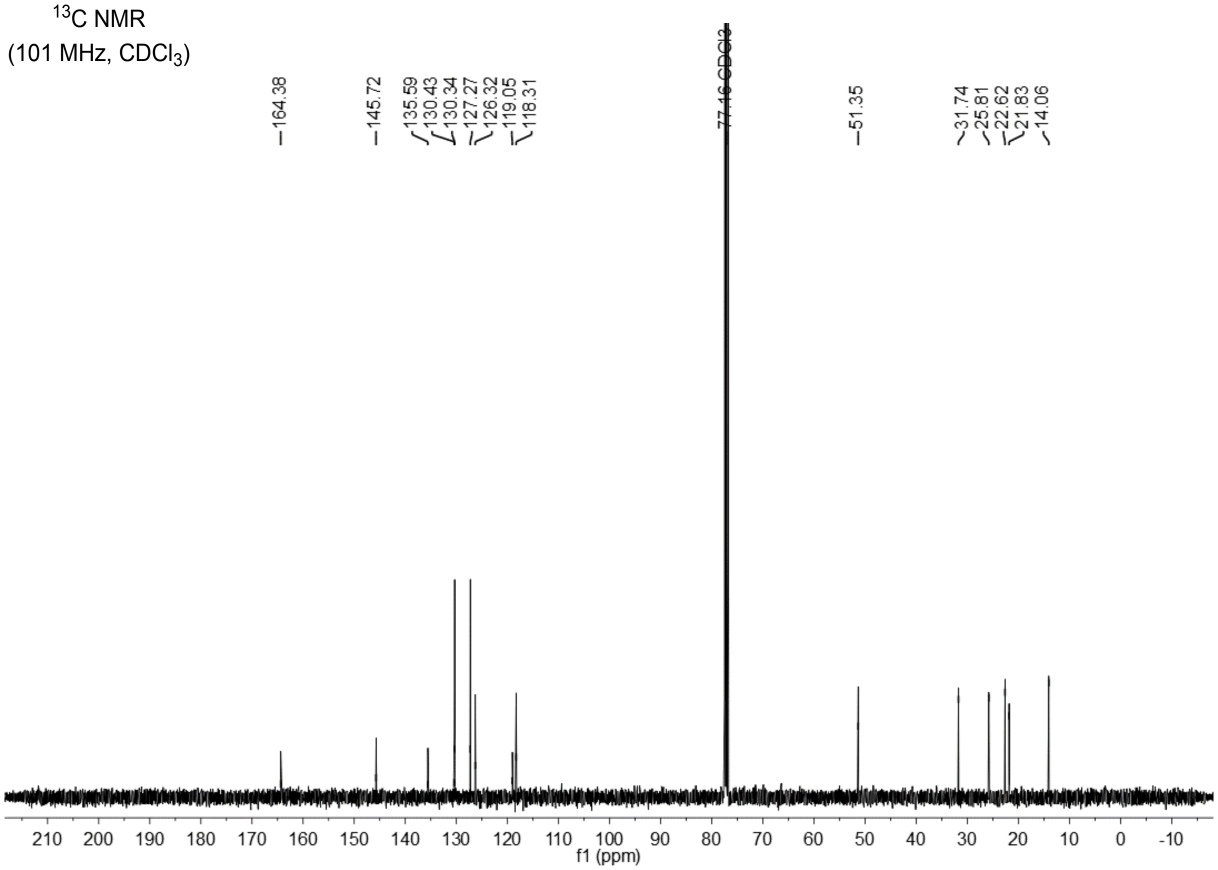

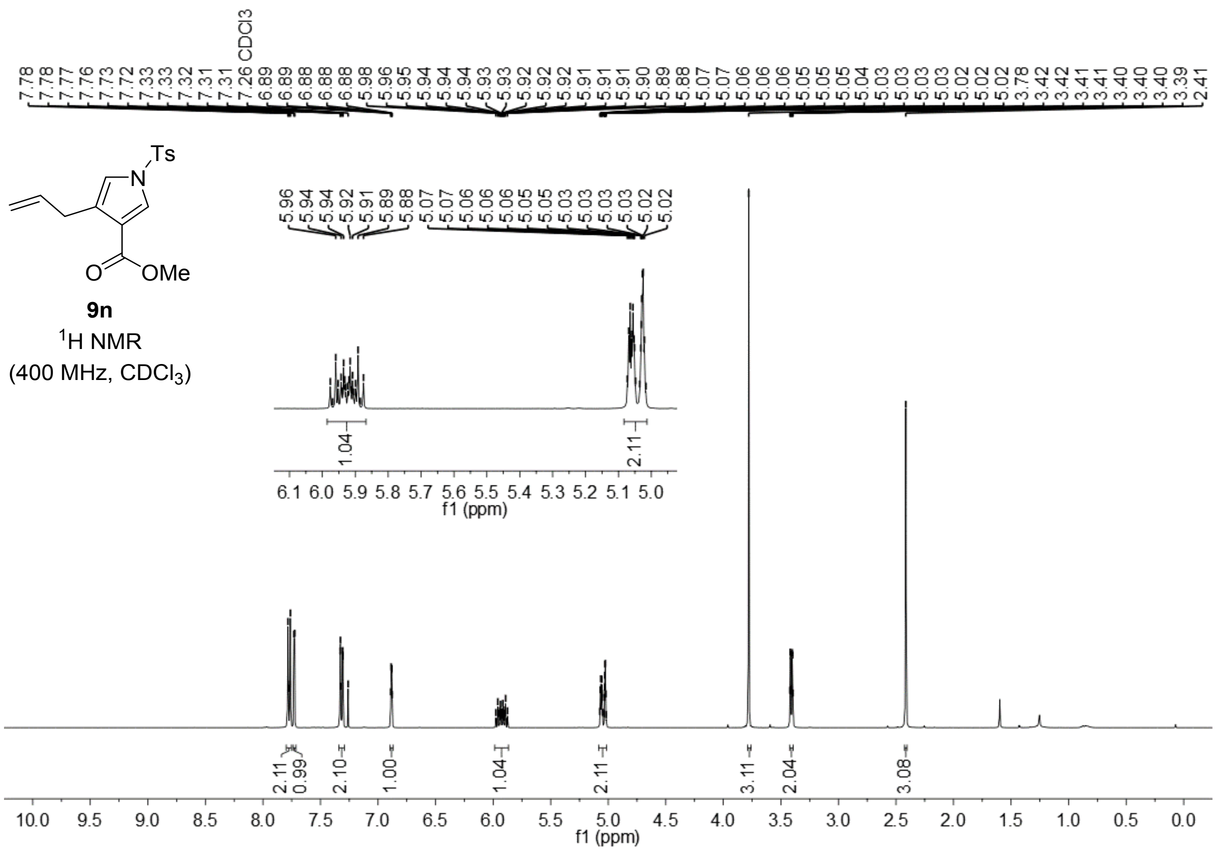

9n
^{1}H NMR
(400 MHz, $CDCl_3$)

9n
^{13}C NMR
(101 MHz, $CDCl_3$)

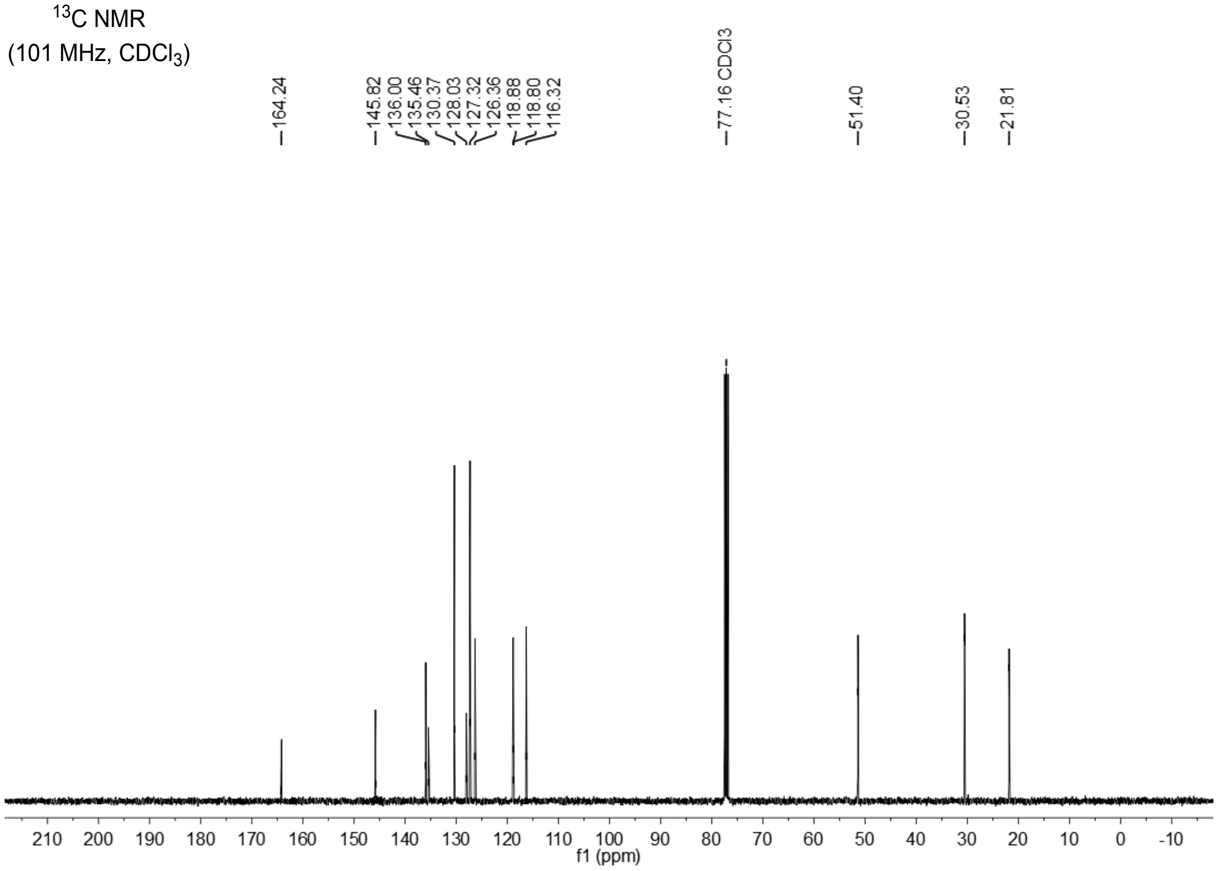

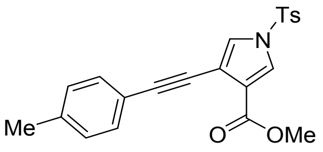

9o
^{1}H NMR
(400 MHz, $CDCl_3$)

9o
^{13}C NMR
(101 MHz, $CDCl_3$)

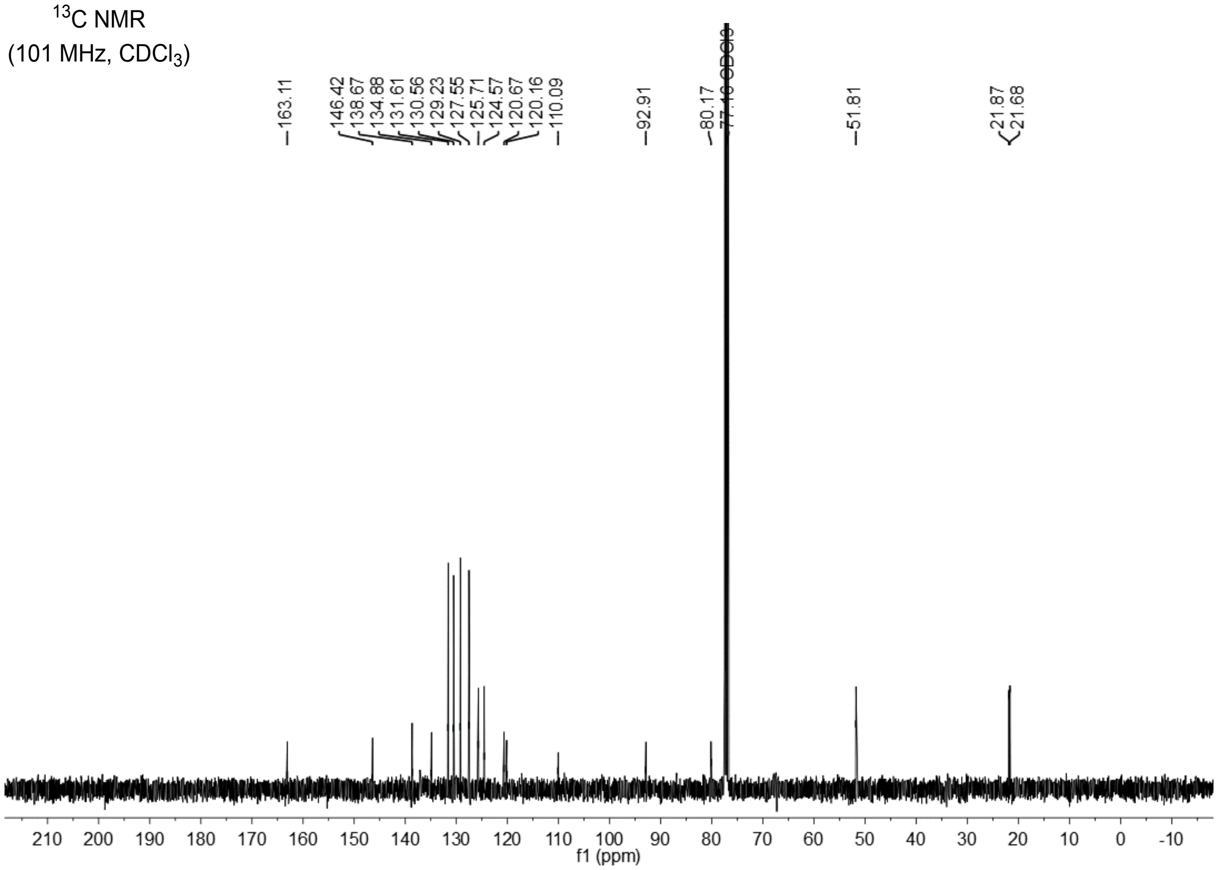

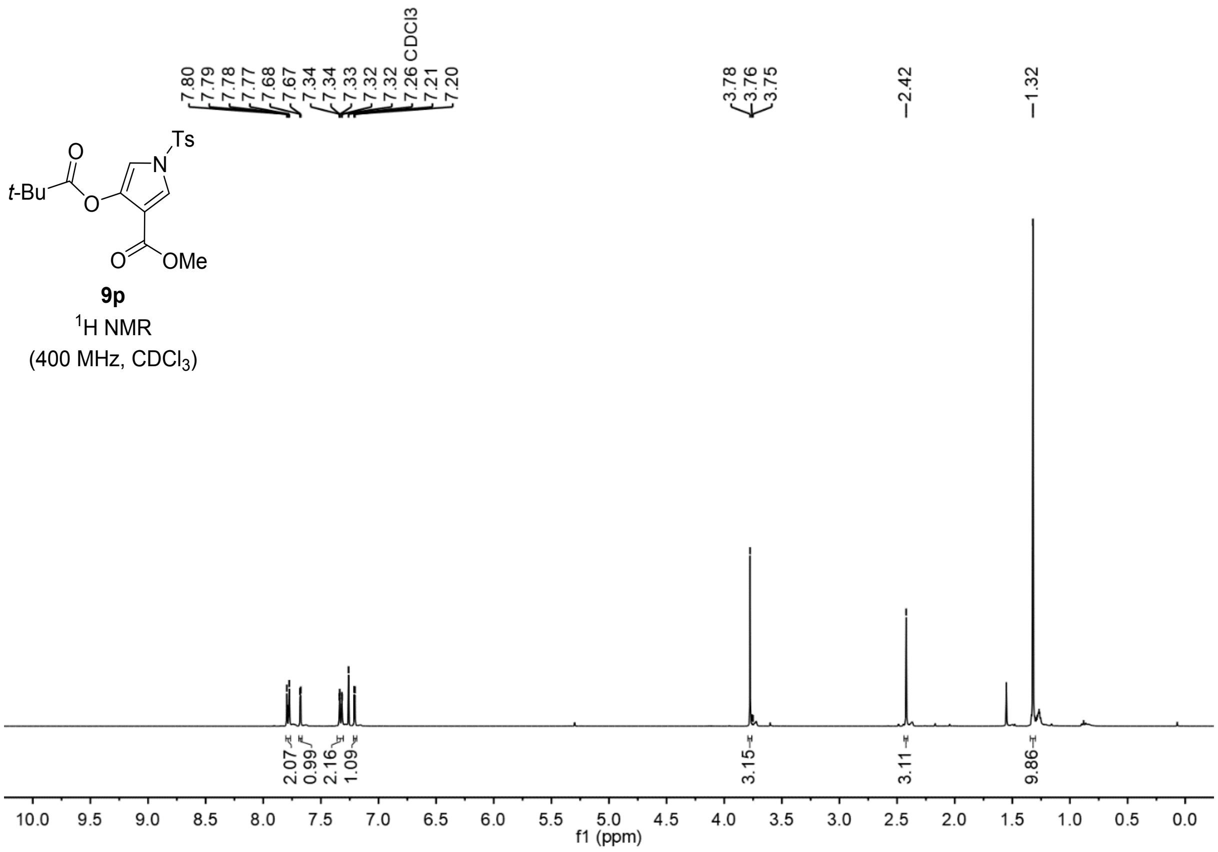
7.80
7.79
7.78
7.77
7.68
7.67
7.34
7.34
7.33
7.32
7.32
7.26 CDCl3
7.21
7.20
3.78
3.76
3.75
2.42
1.32
Ts
N
O
t-Bu
O
O
OMe
9p
1H NMR
(400 MHz, CDCl3)
2.07
0.99
2.16
1.09
3.15
3.11
9.86
f1 (ppm)

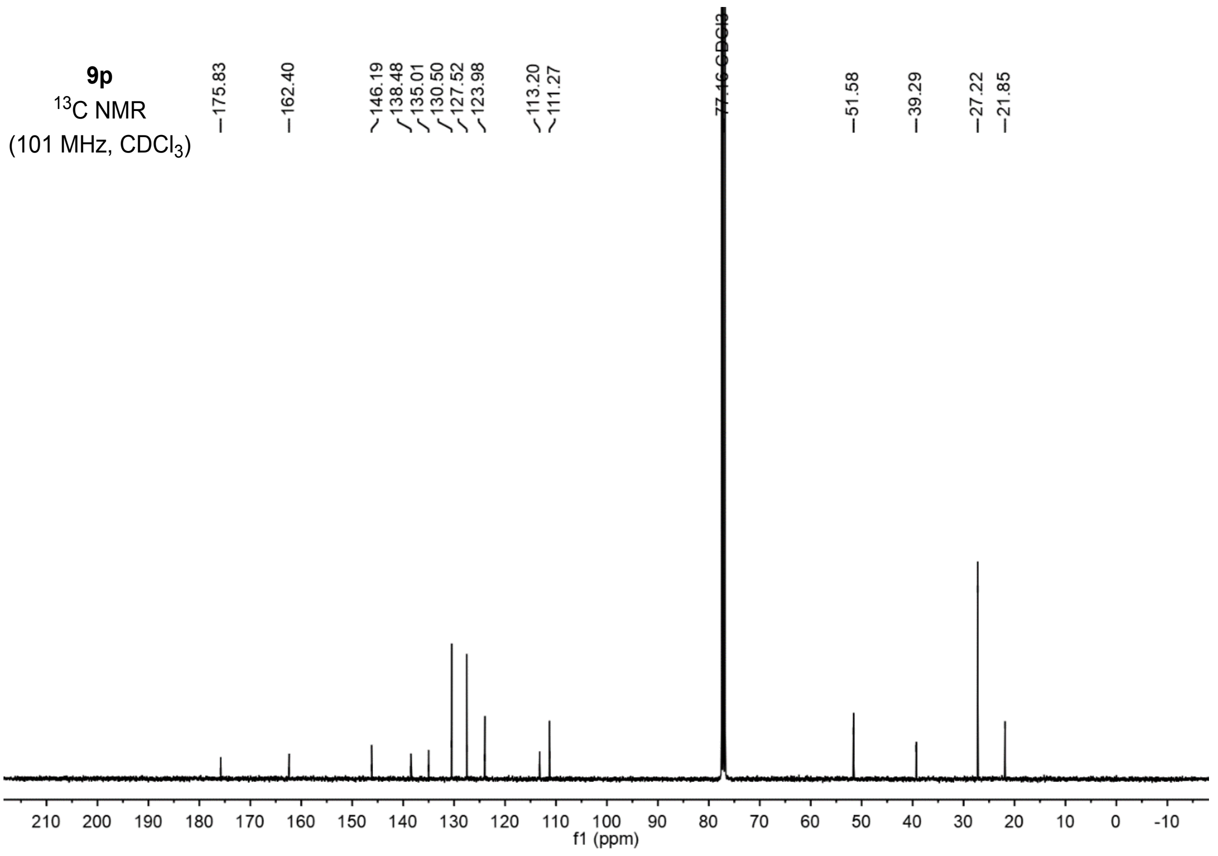
9p
13C NMR
(101 MHz, CDCl3)
175.83
162.40
146.19
138.48
135.01
130.50
127.52
123.98
113.20
111.27
77.16 CDCl3
51.58
39.29
27.22
21.85
f1 (ppm)

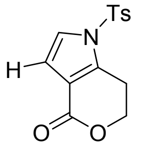

9q
^{1}H NMR
(400 MHz, $CDCl_3$)

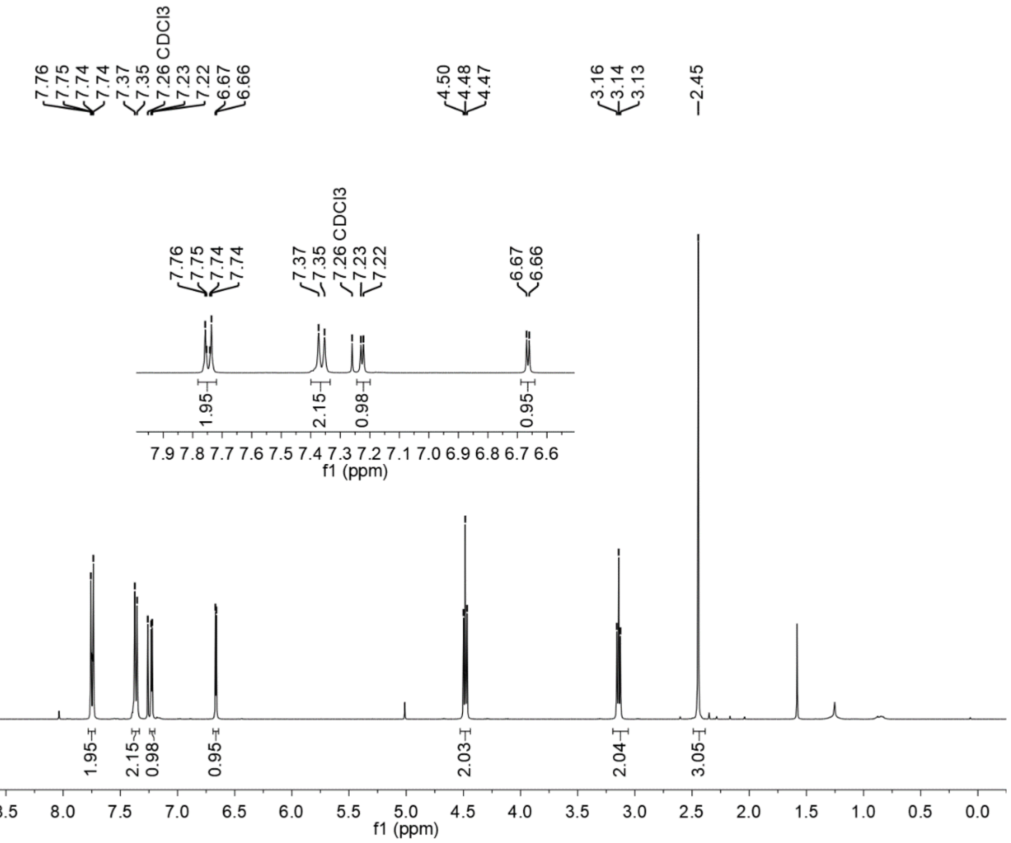

9q
^{13}C NMR
(101 MHz, $CDCl_3$)

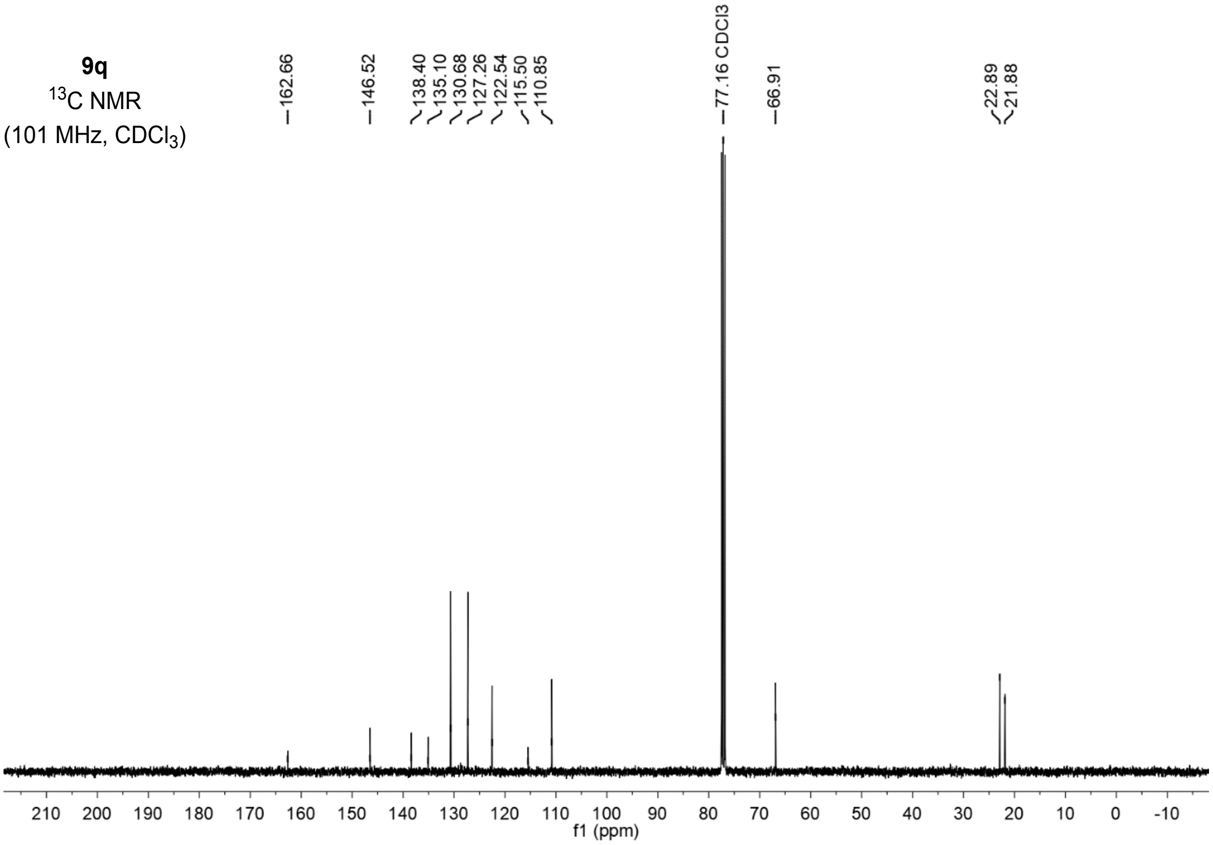

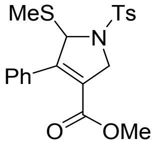

10
^{1}H NMR
(400 MHz, $CDCl_3$)

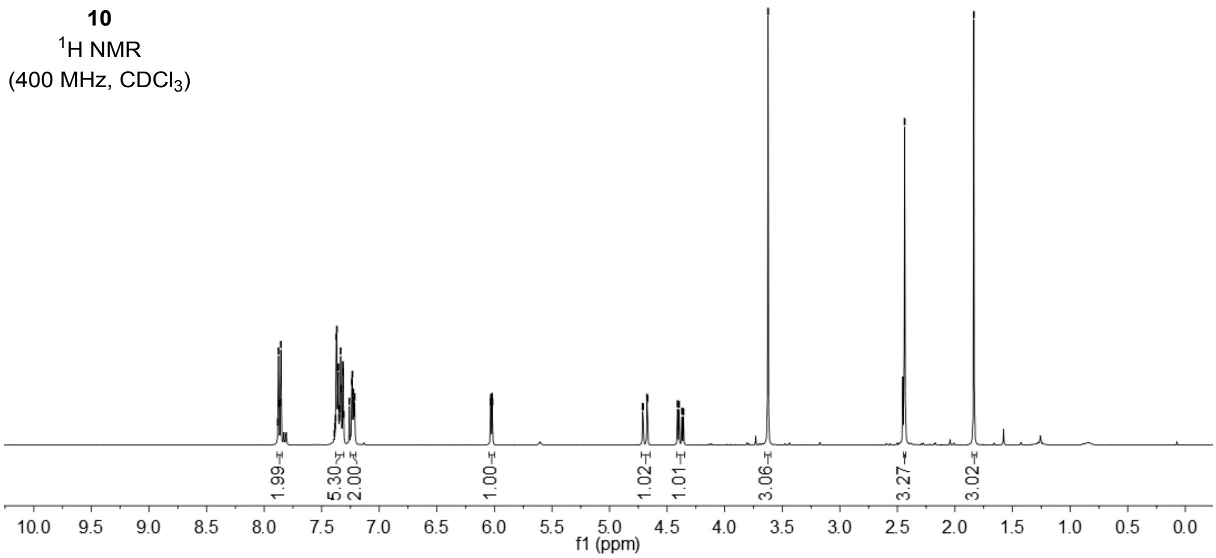

10
^{13}C NMR
(101 MHz, $CDCl_3$)

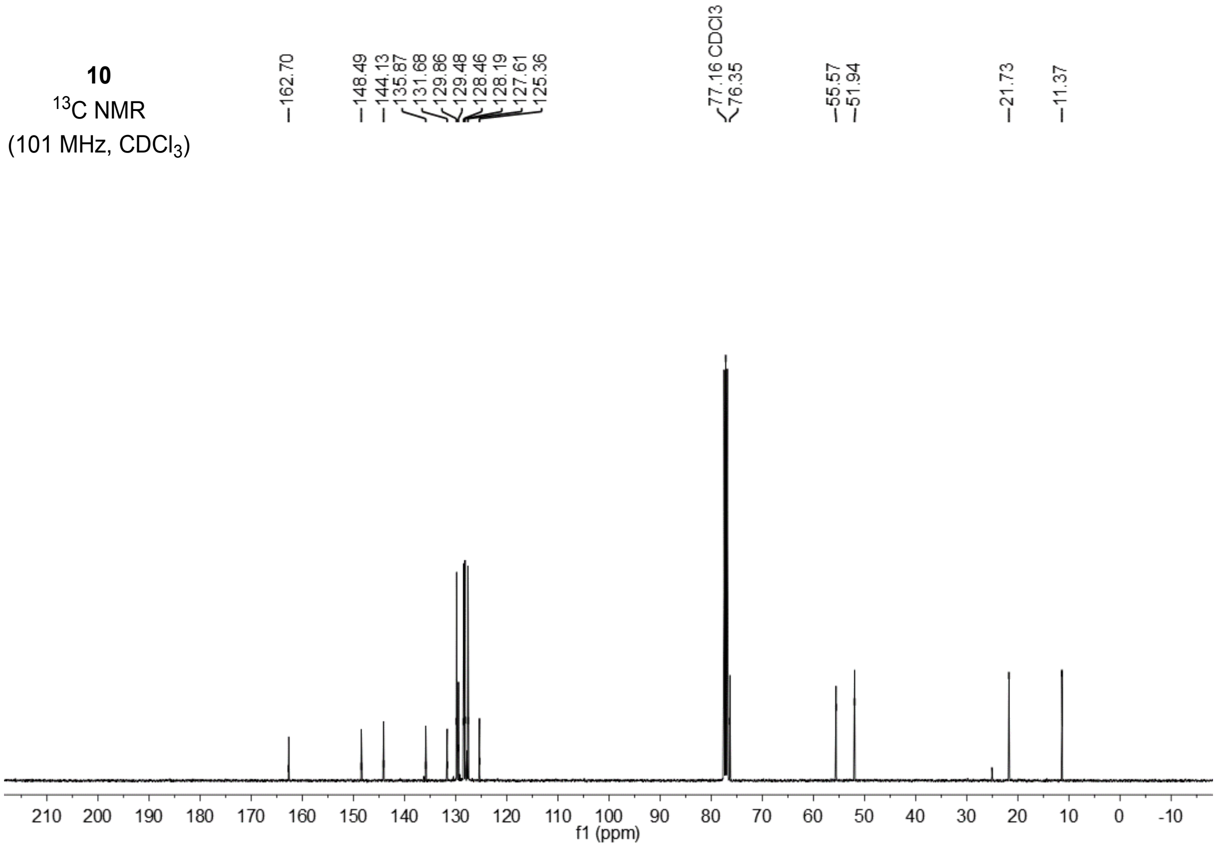

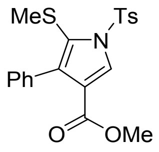

11
^{1}H NMR
(400 MHz, $CDCl_3$)

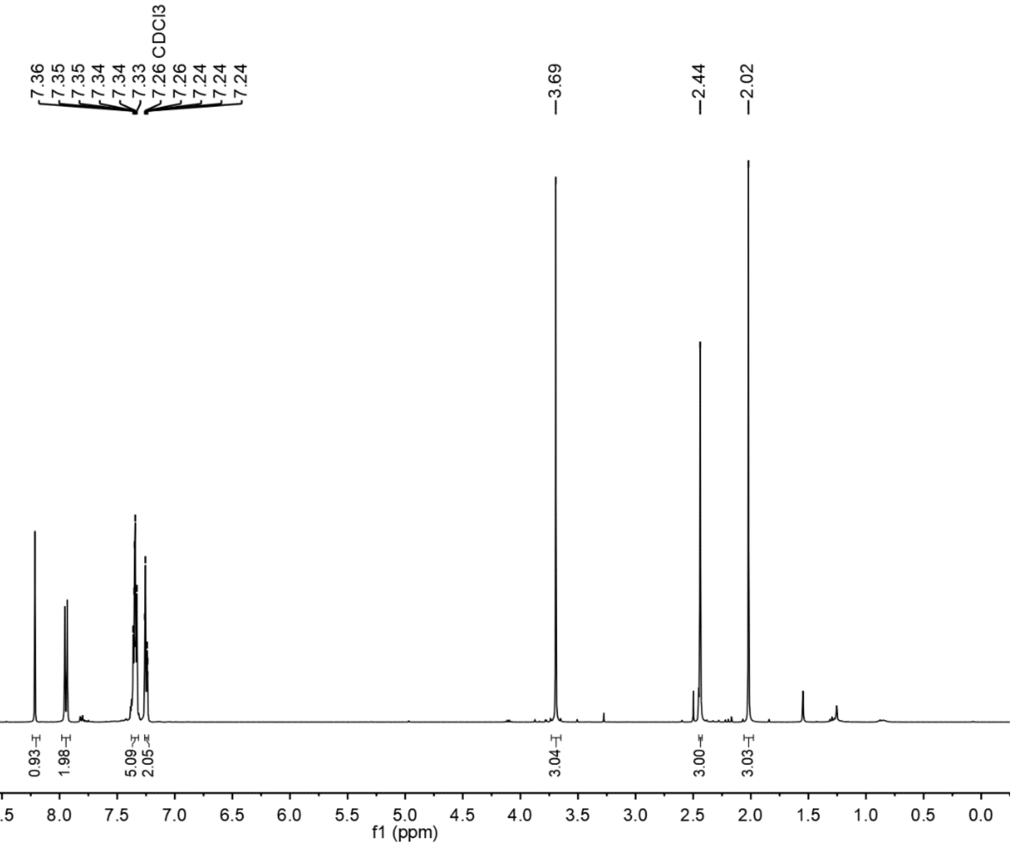

11
^{13}C NMR
(101 MHz, $CDCl_3$)

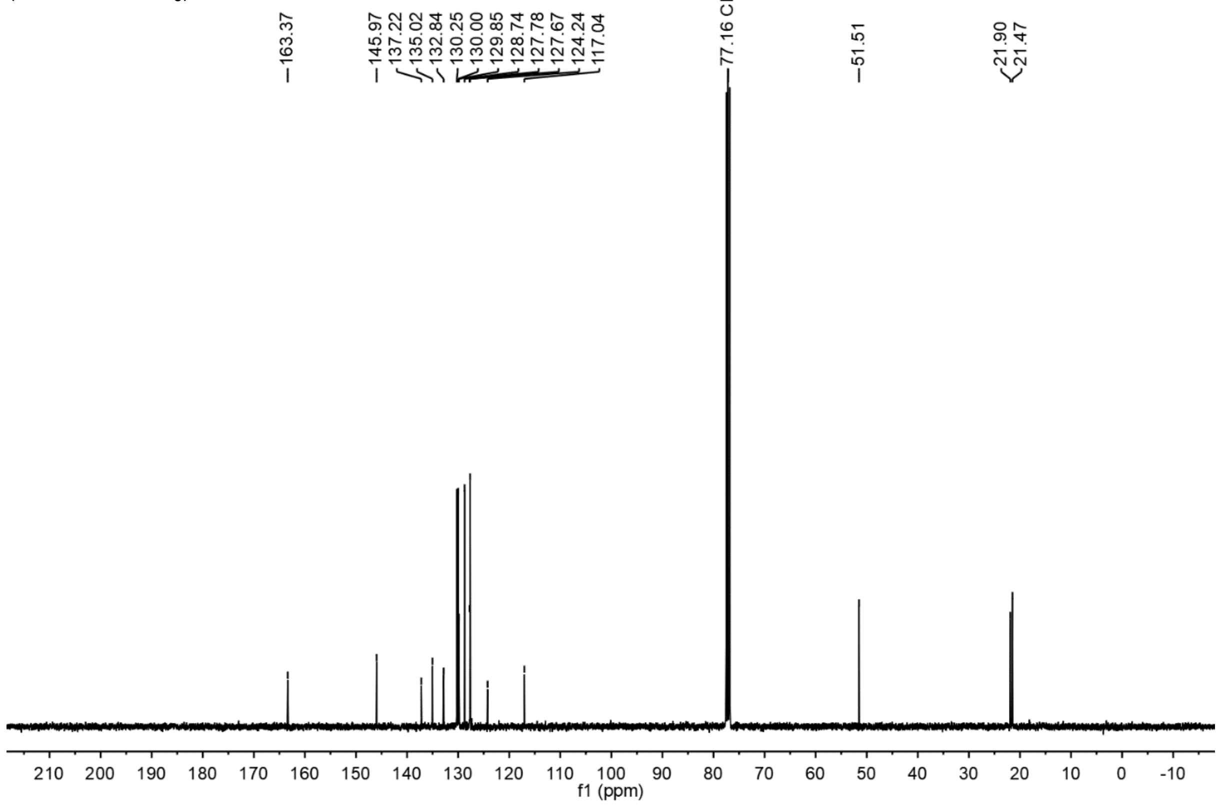

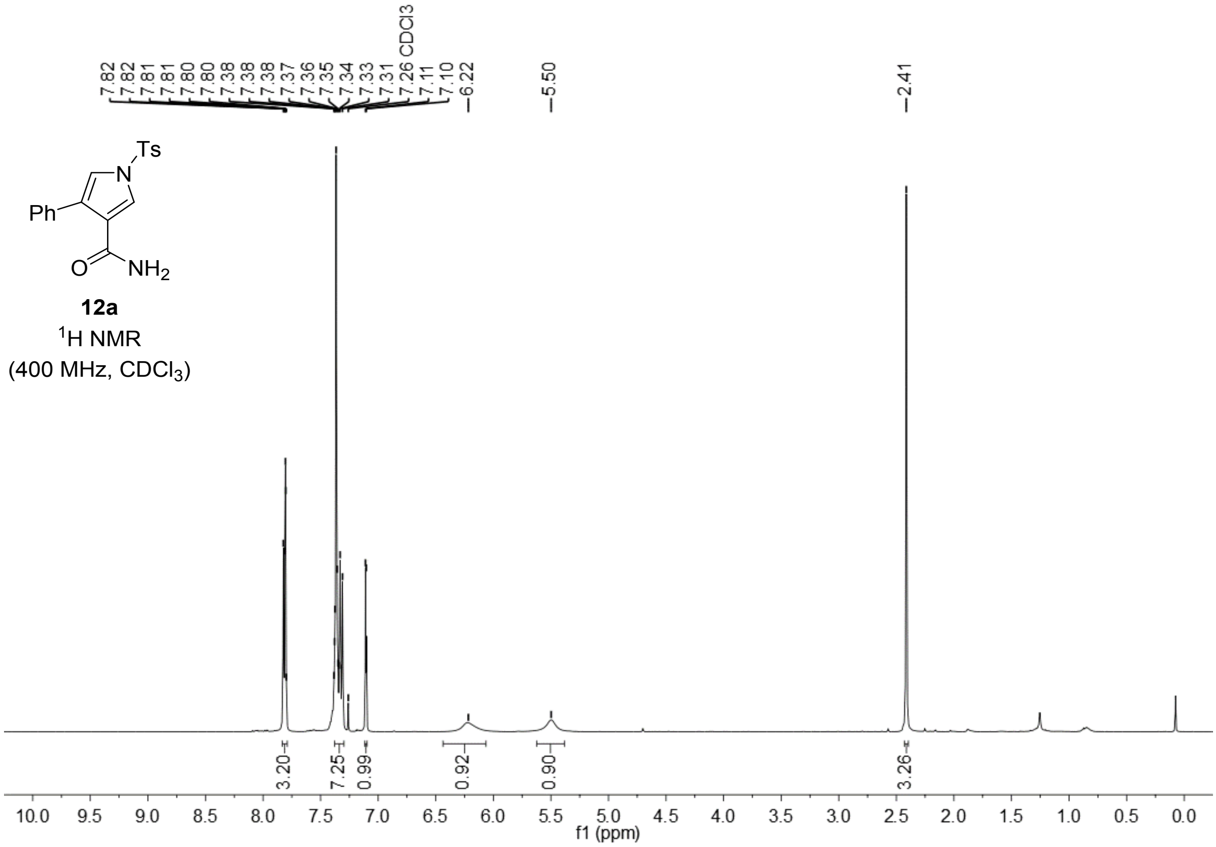

12a
^{1}H NMR
(400 MHz, $CDCl_3$)

12a
^{13}C NMR
(101 MHz, $CDCl_3$)

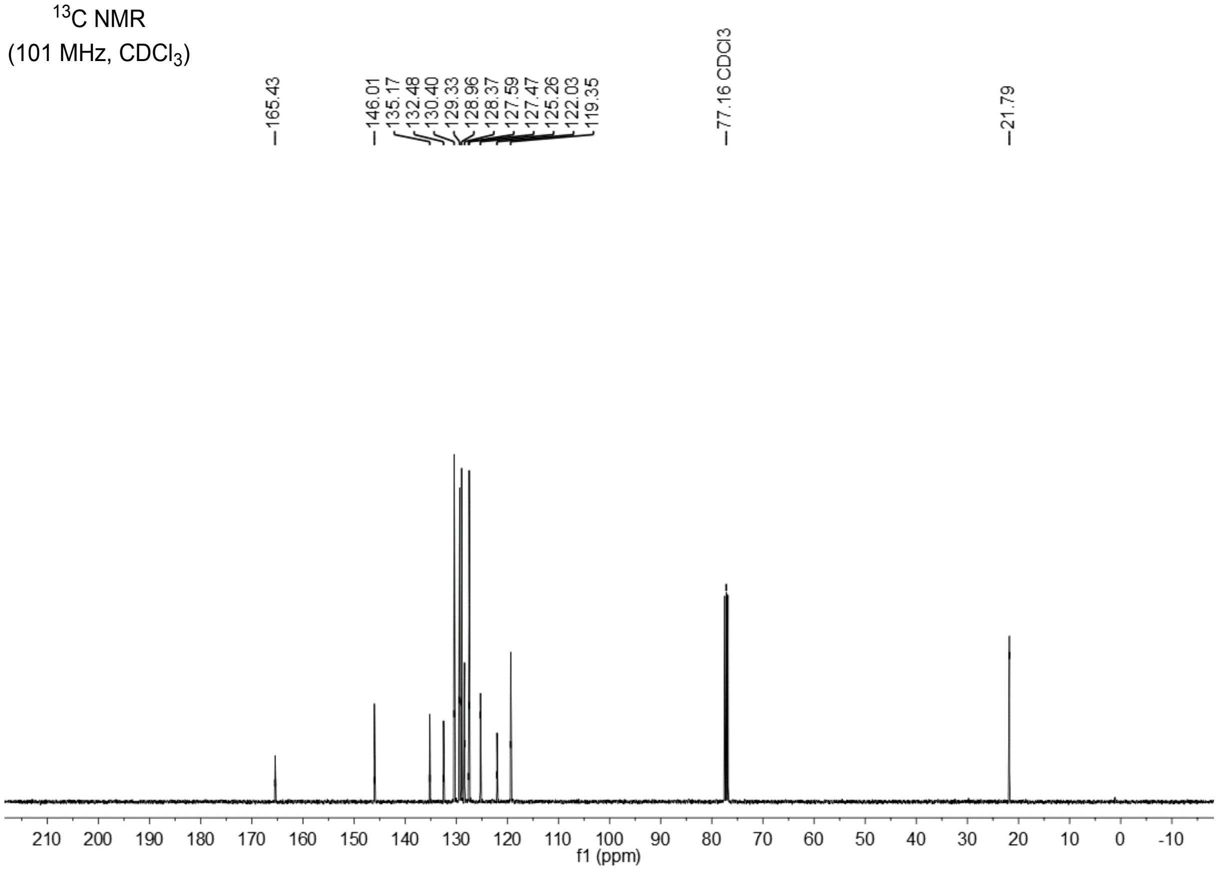

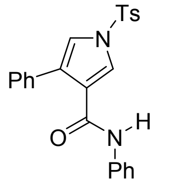

12b

^{1}H NMR

(400 MHz, $CDCl_3$)

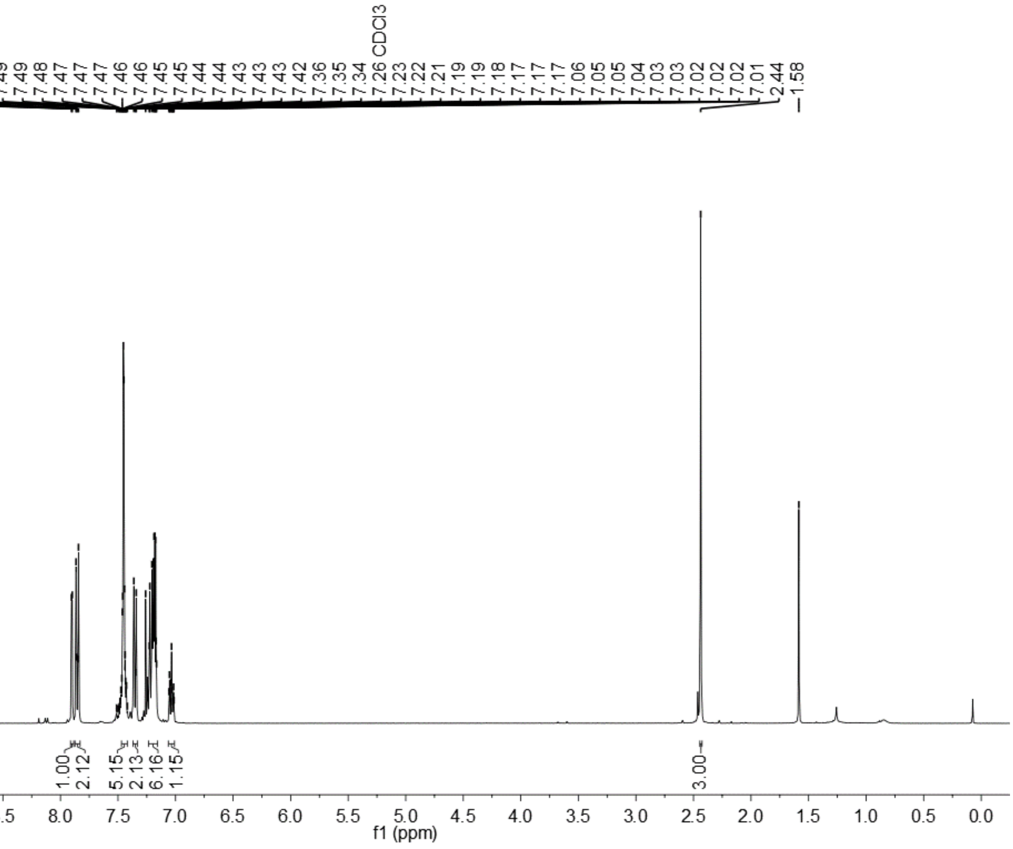

12b

^{13}C NMR

(101 MHz, $CDCl_3$)

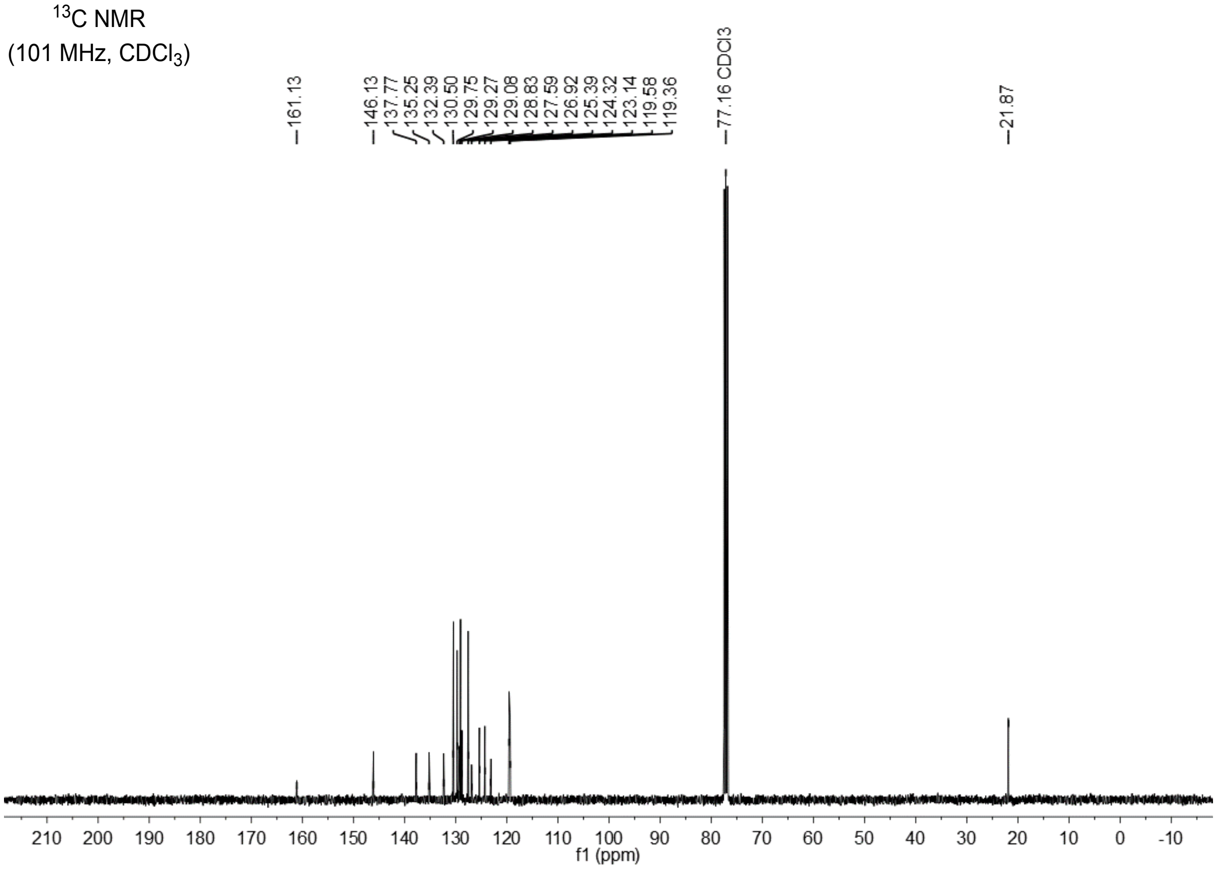

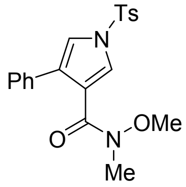

12c
^{1}H NMR
(400 MHz, $CDCl_3$)

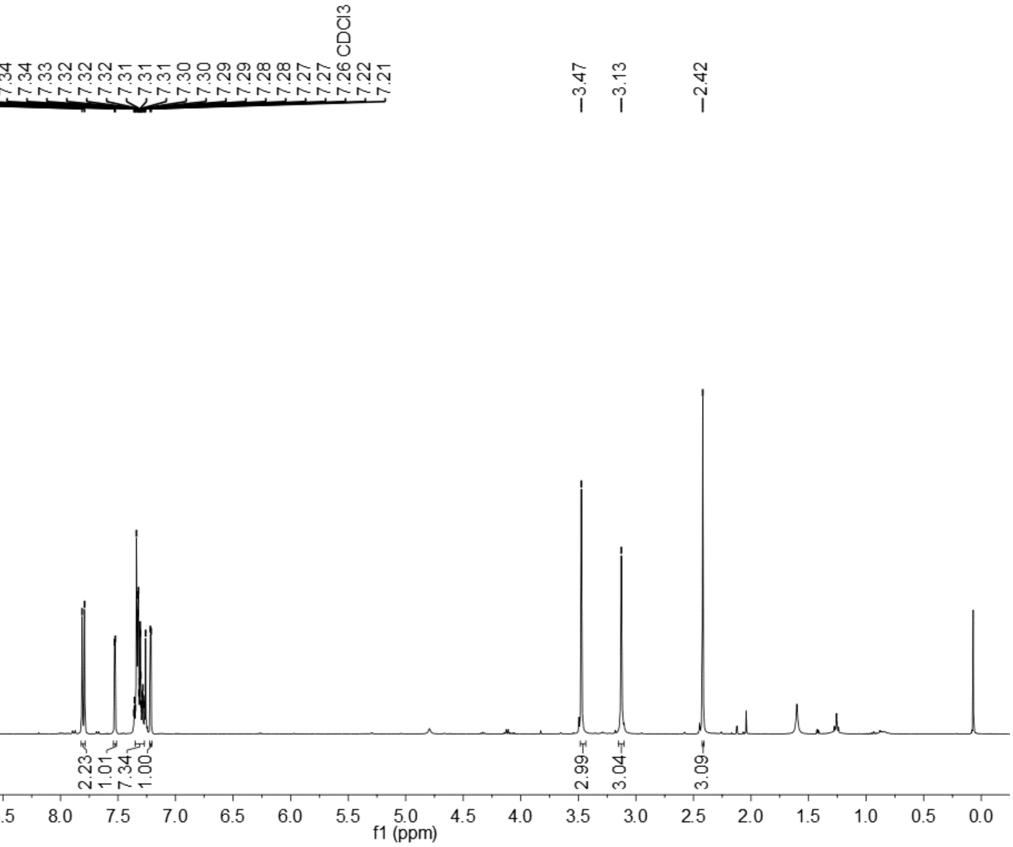

12c
^{13}C NMR
(101 MHz, $CDCl_3$)

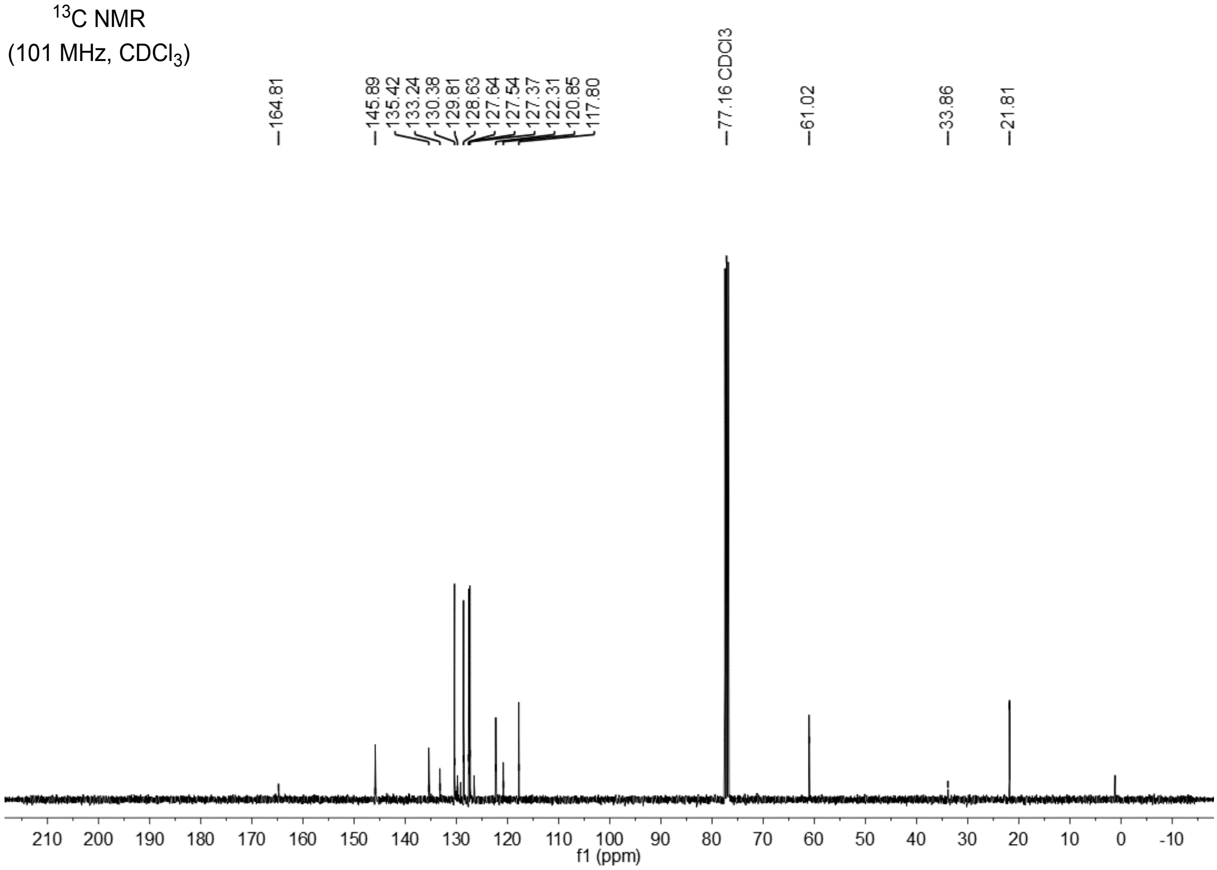

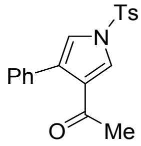

13a
^{1}H NMR
(400 MHz, $CDCl_3$)

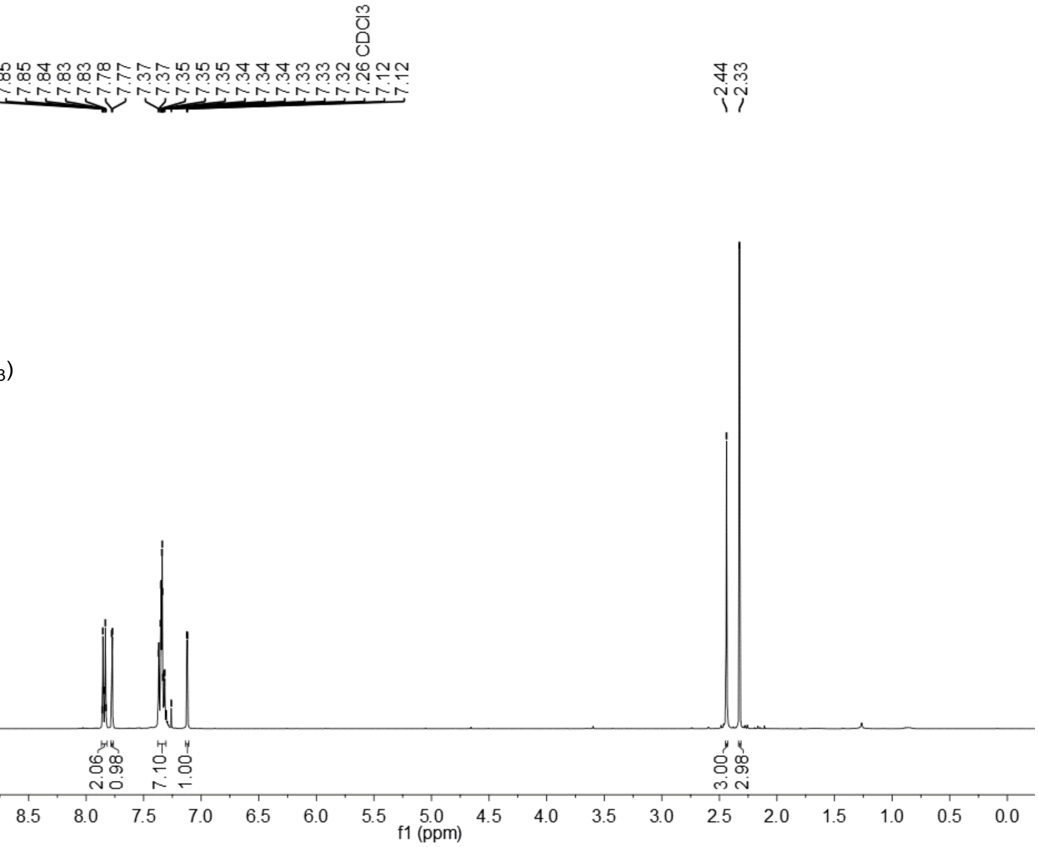

13a
^{13}C NMR
(101 MHz, $CDCl_3$)

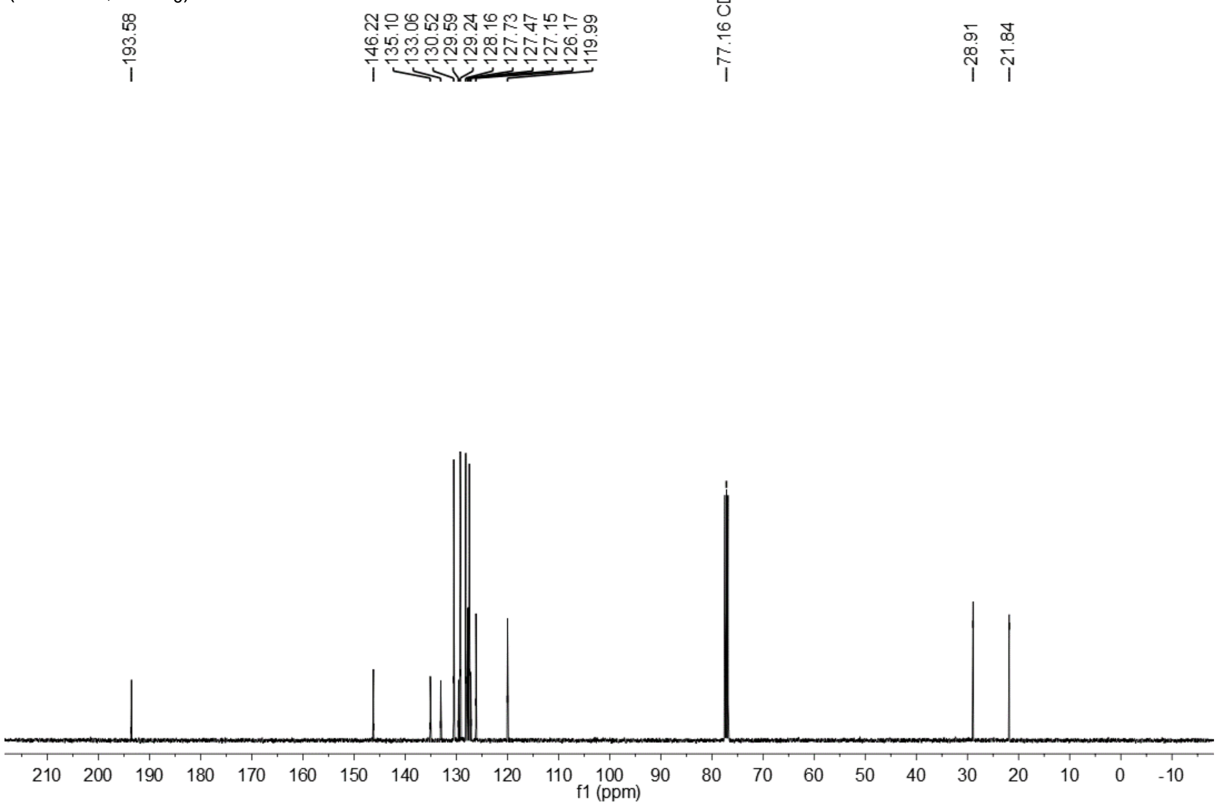

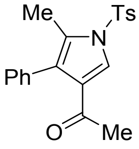

13b
^{1}H NMR
(400 MHz, $CDCl_3$)

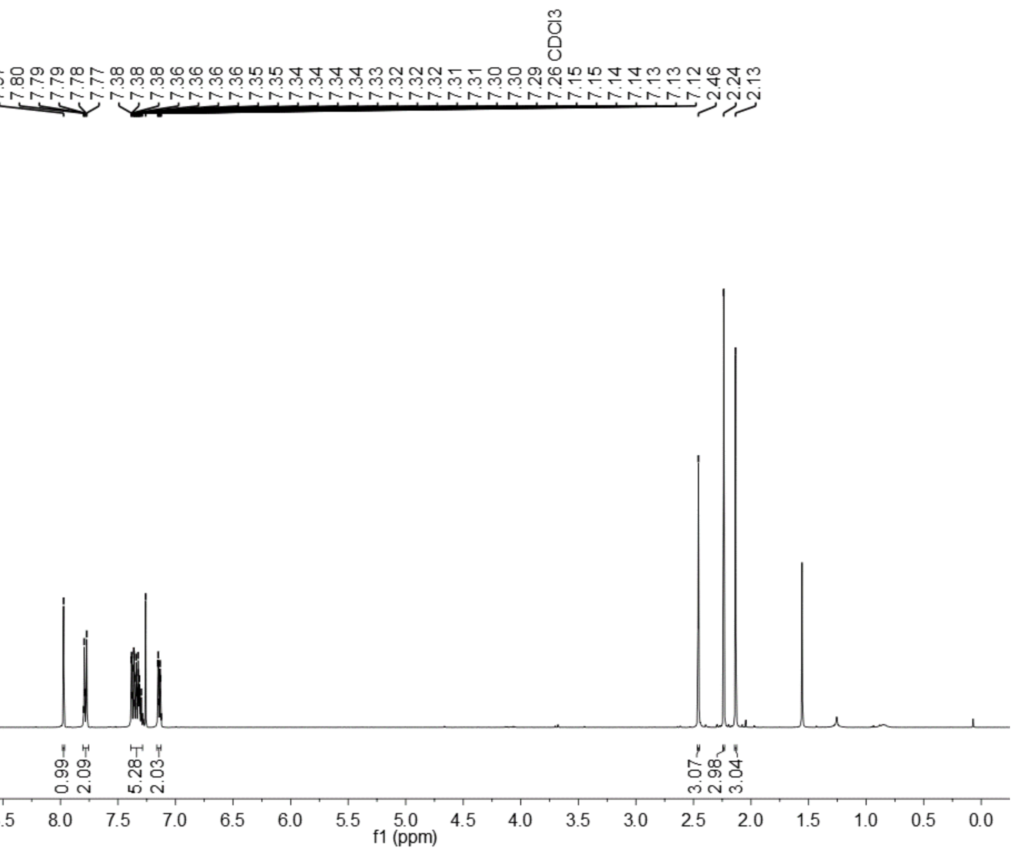

13b
^{13}C NMR
(101 MHz, $CDCl_3$)

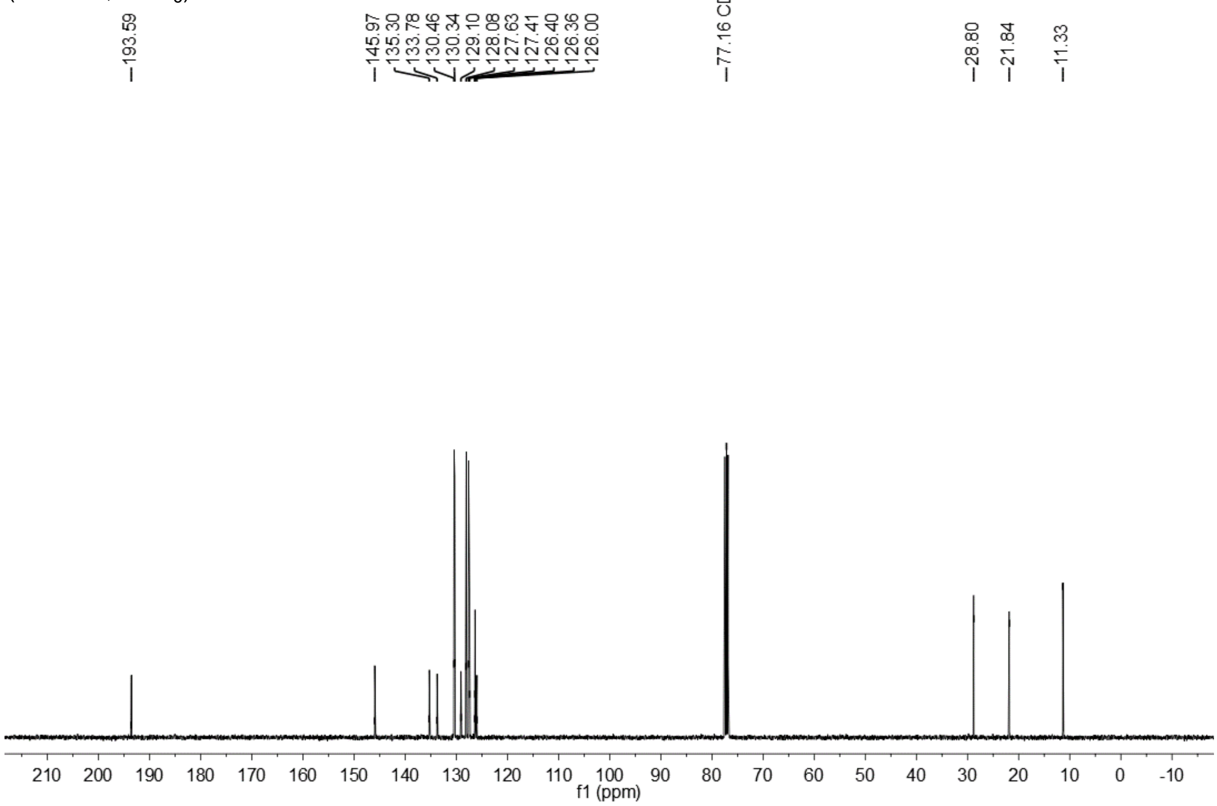

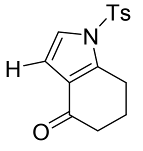

13c
^{1}H NMR
(400 MHz, $CDCl_3$)

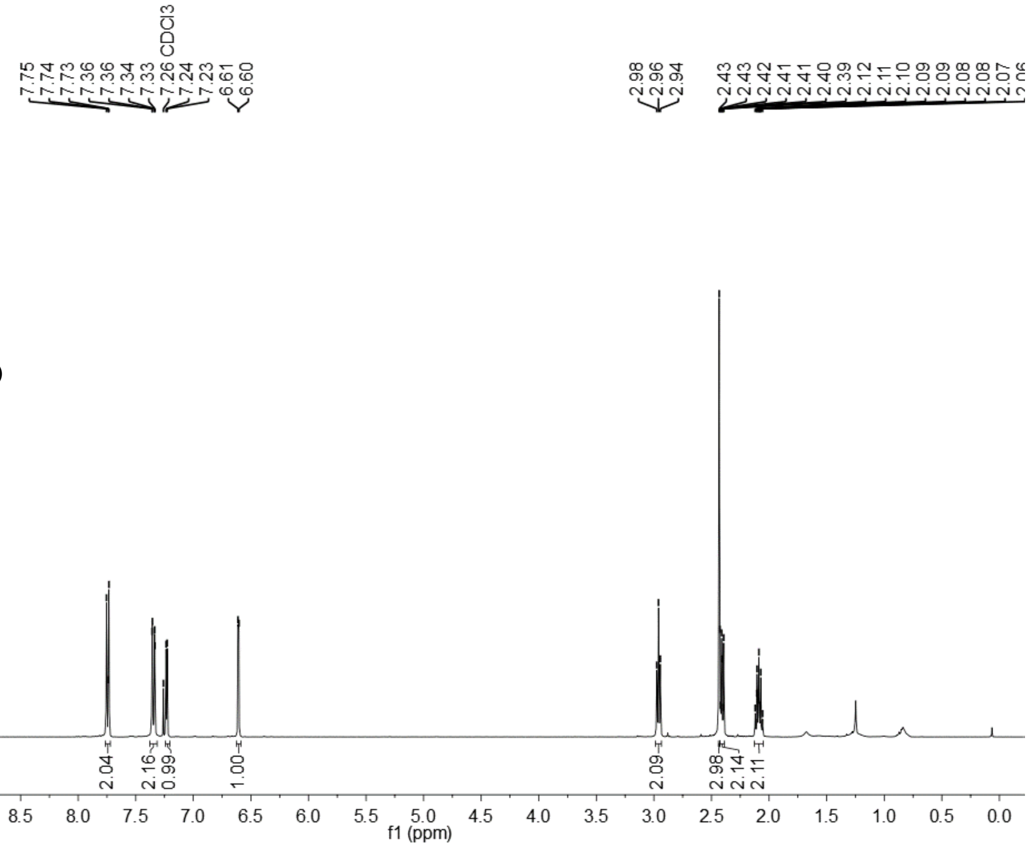

13c
^{13}C NMR
(101 MHz, $CDCl_3$)

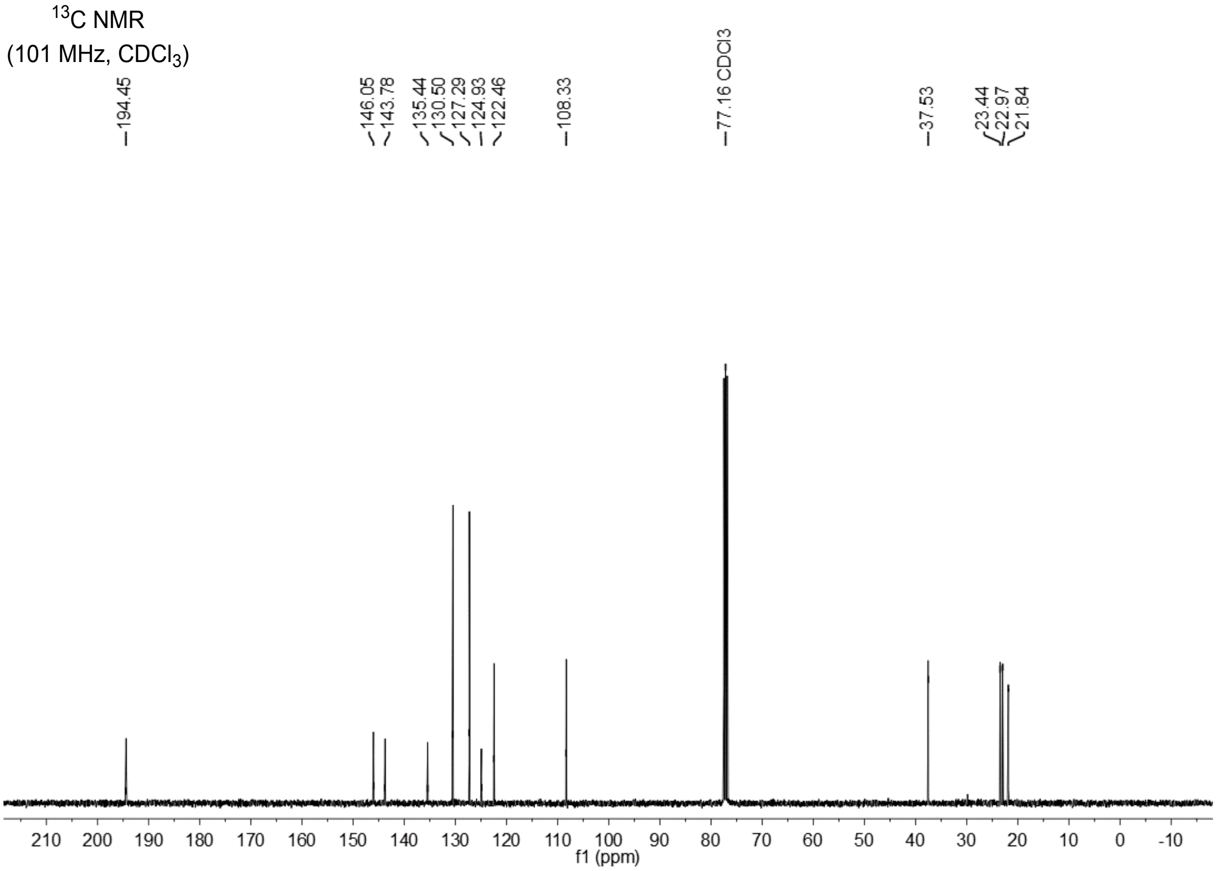

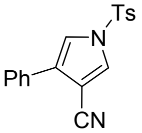

14a
^{1}H NMR
(400 MHz, $CDCl_3$)

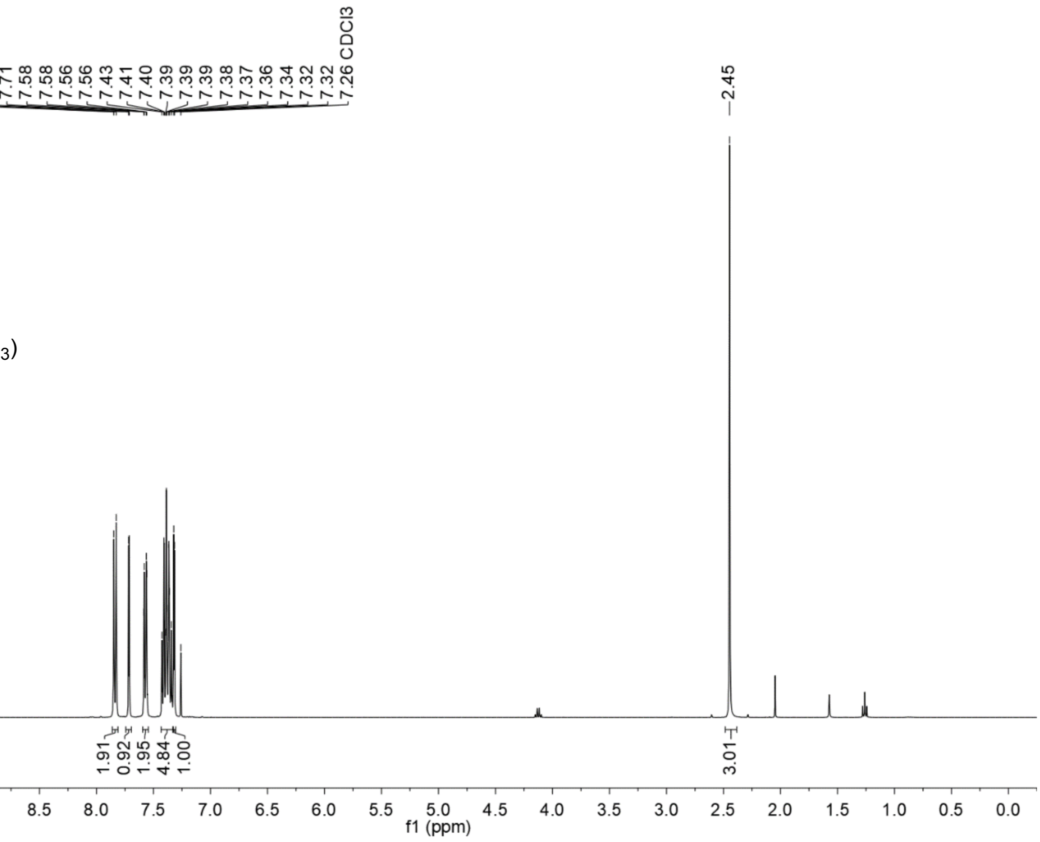

14a
^{13}C NMR
(101 MHz, $CDCl_3$)

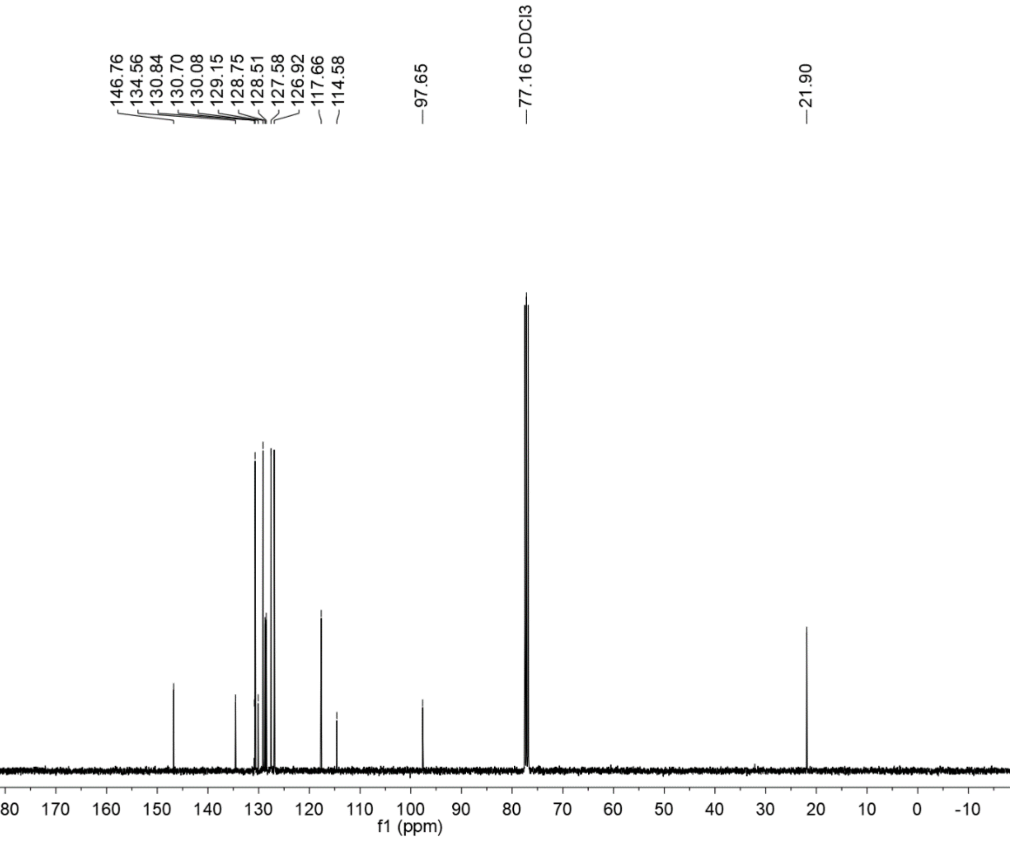

14b
^{1}H NMR
(400 MHz, $CDCl_3$)

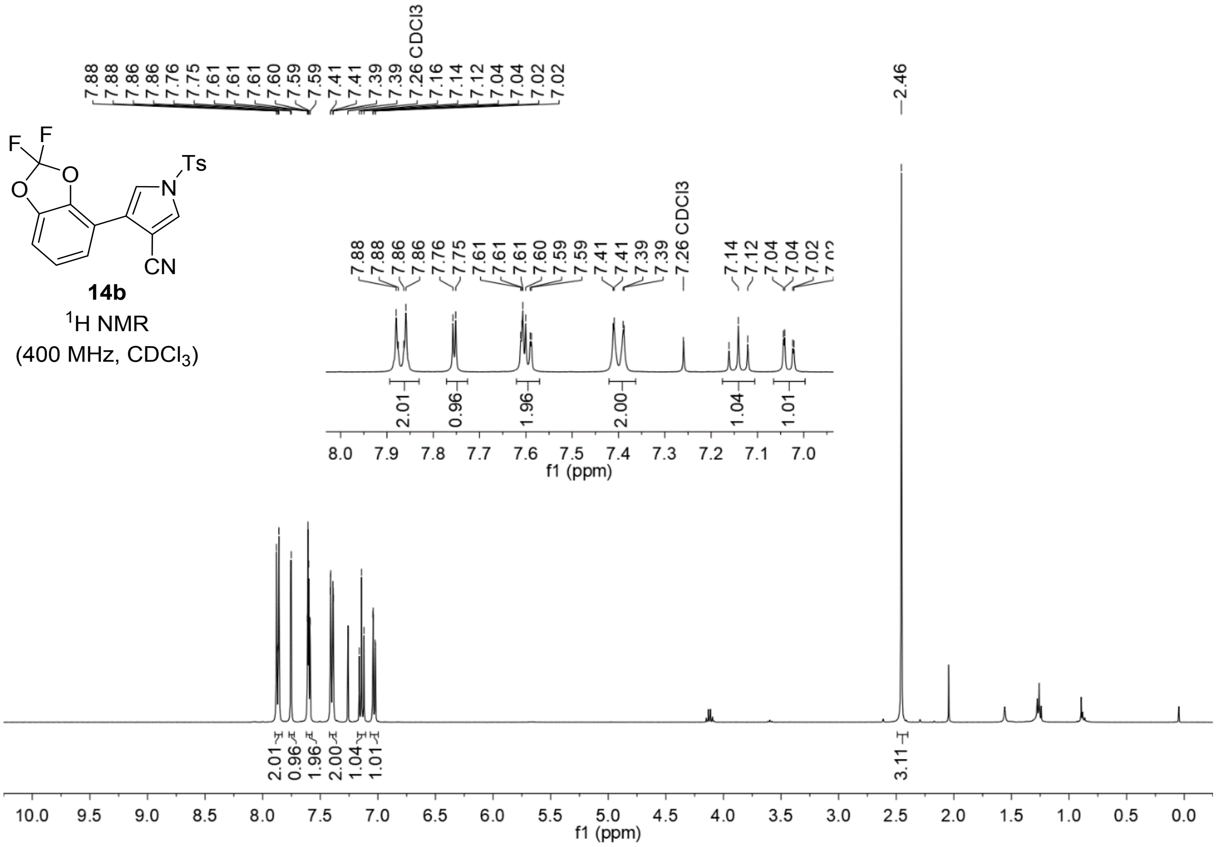

14b
^{13}C NMR
(101 MHz, $CDCl_3$)

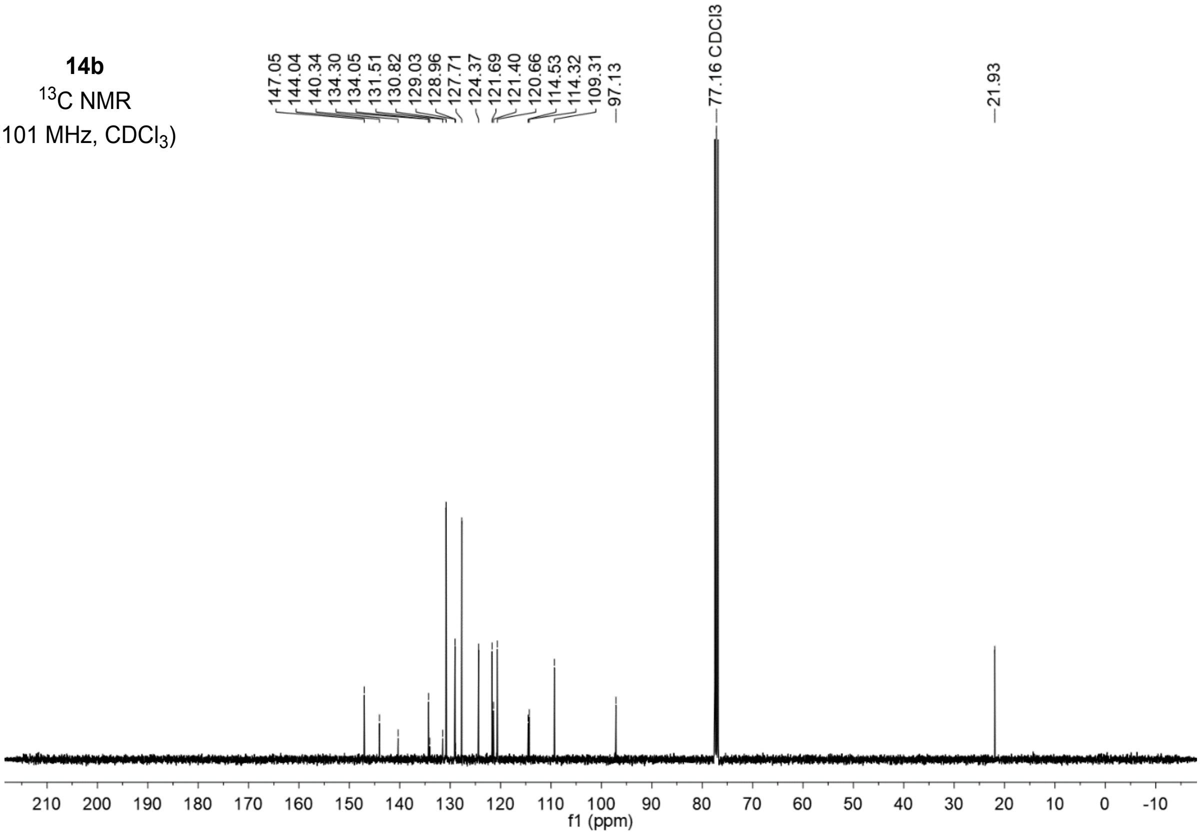

14b
^{19}F NMR
(376 MHz, $CDCl_3$)

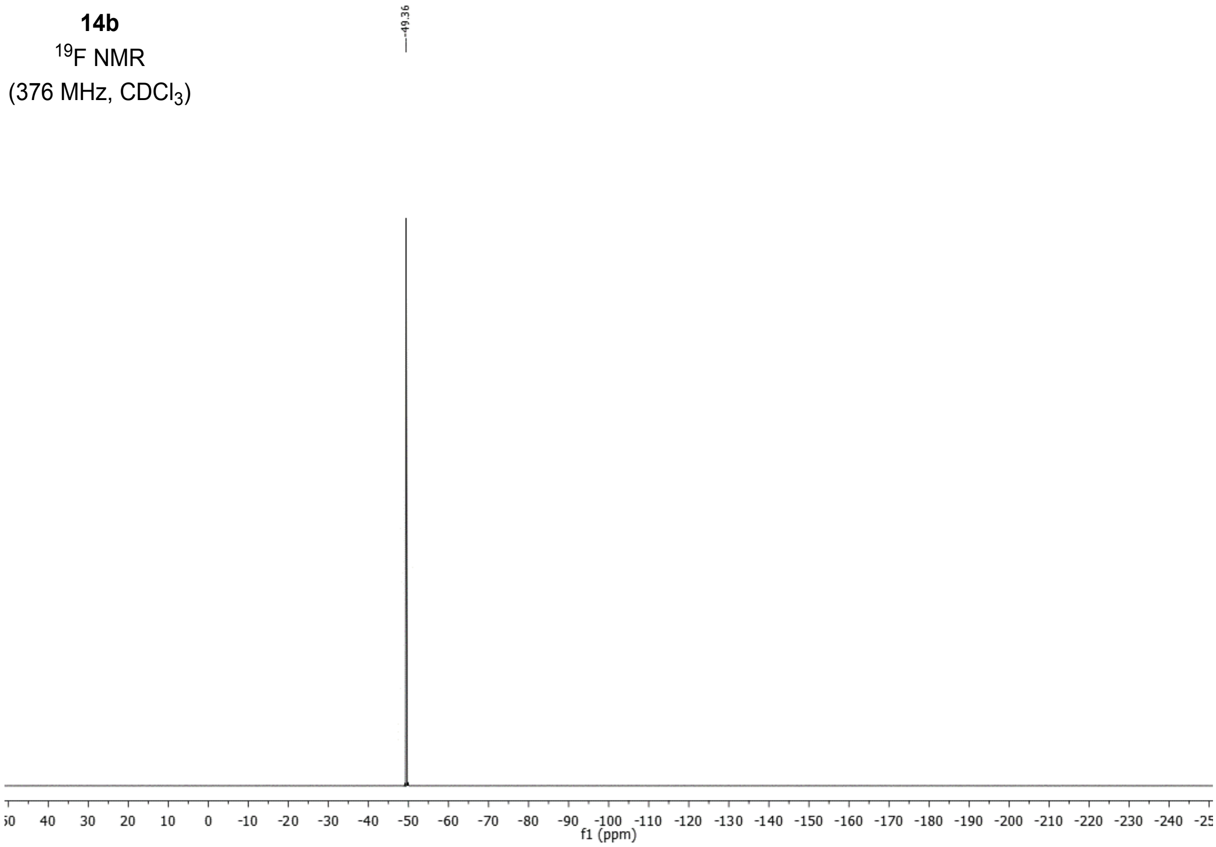

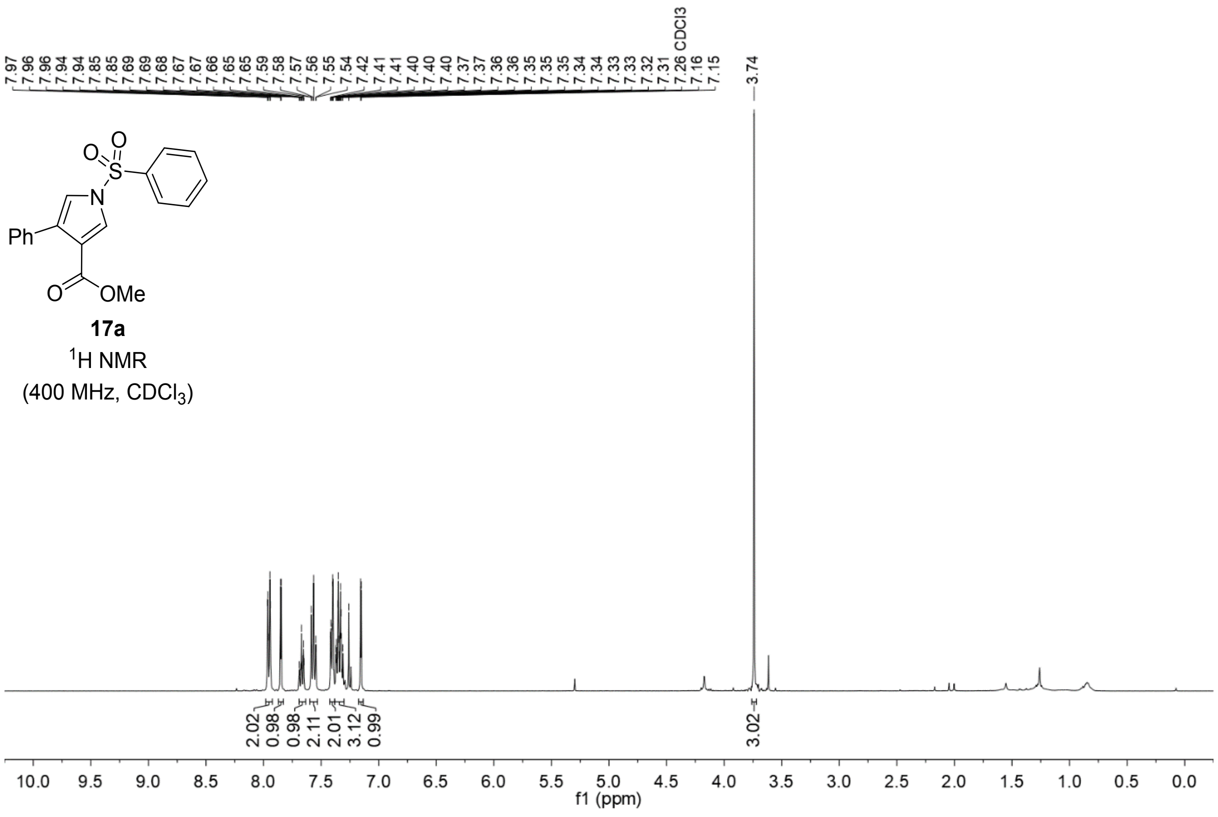

17a

^{1}H NMR

(400 MHz, $CDCl_3$)

17a

^{13}C NMR

(101 MHz, $CDCl_3$)

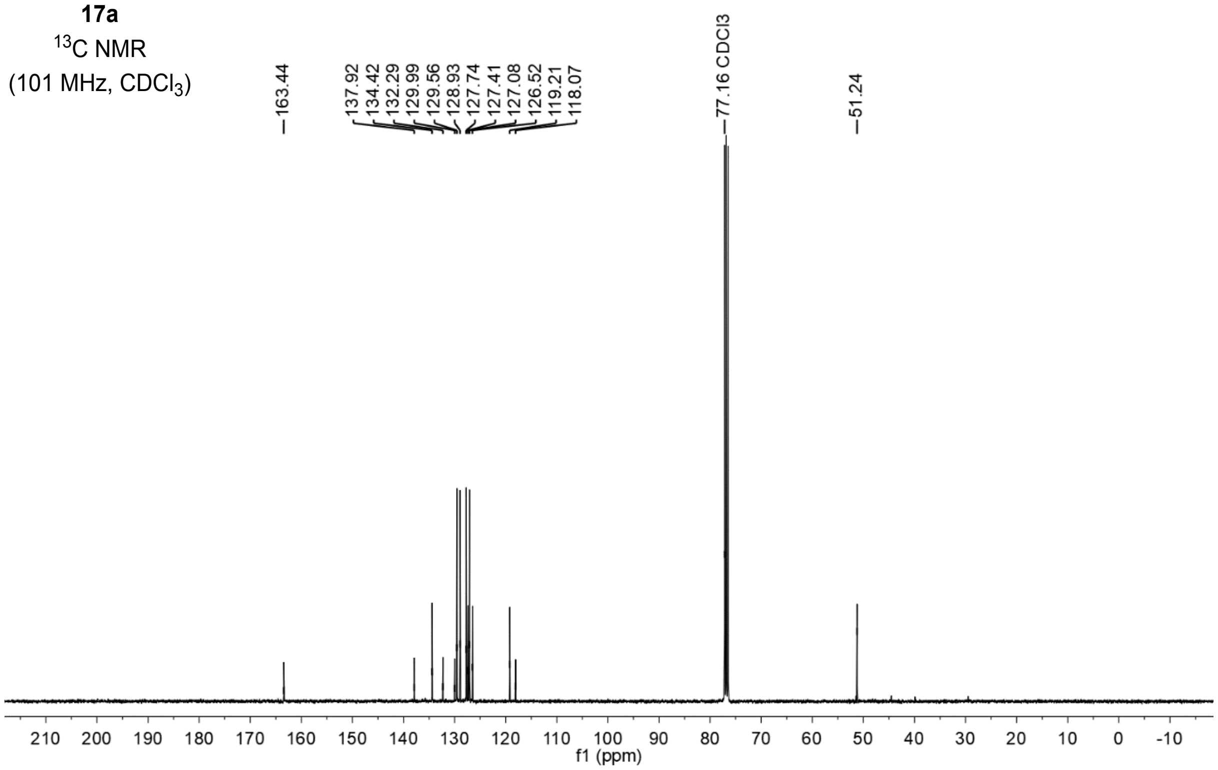

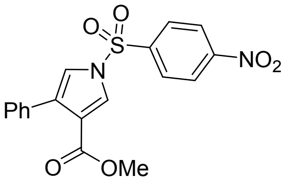

17b
^{1}H NMR
(400 MHz, $CDCl_3$)

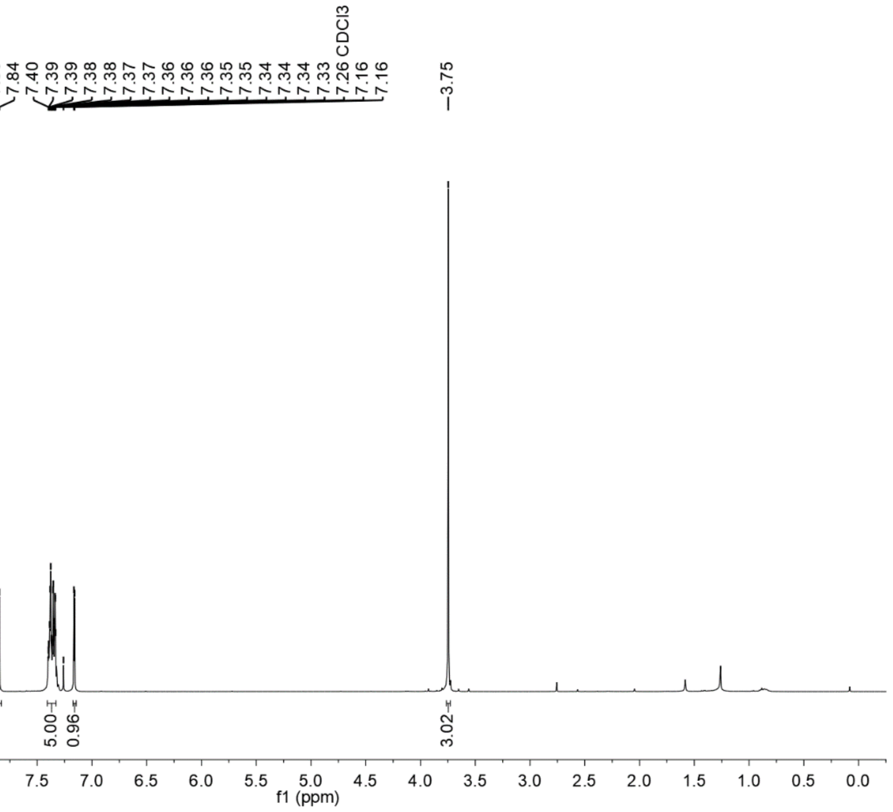

17b
^{13}C NMR
(101 MHz, $CDCl_3$)

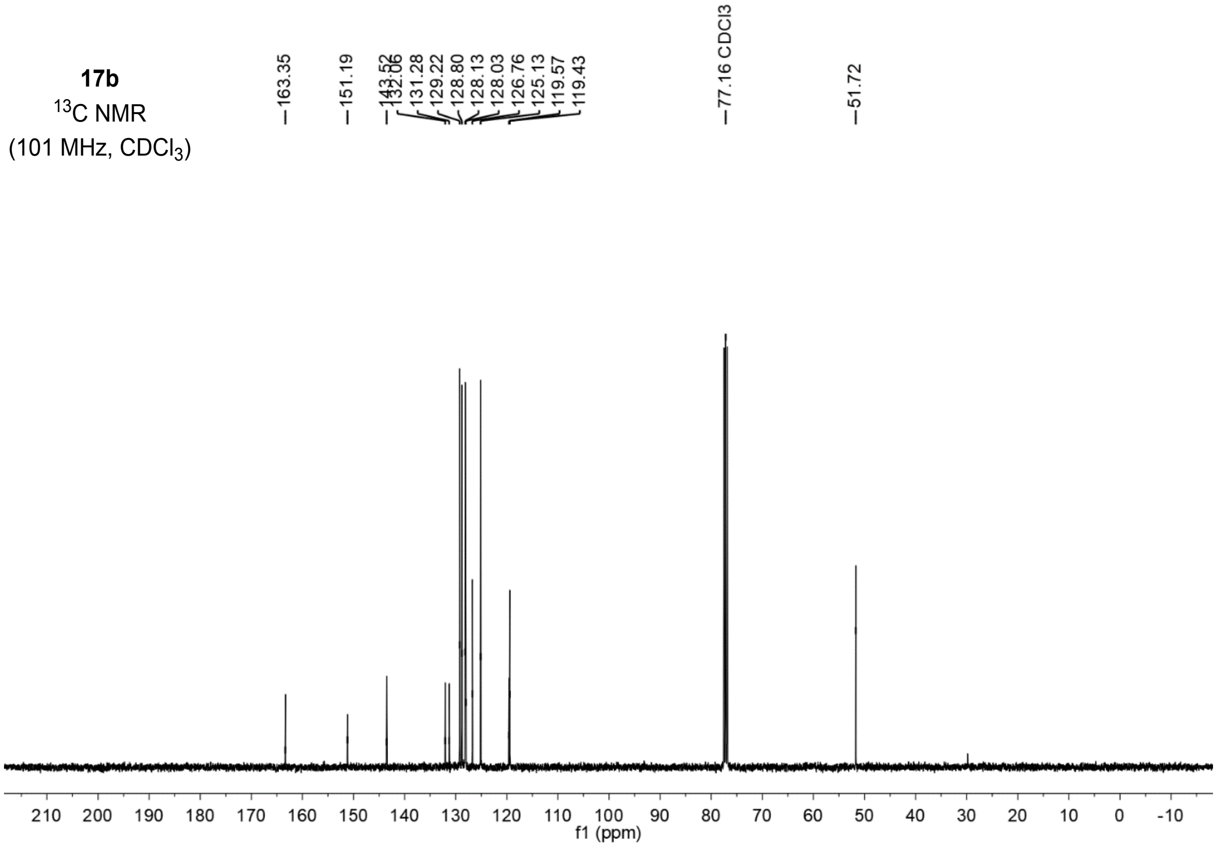

17c

^{1}H NMR

(400 MHz, $CDCl_3$)

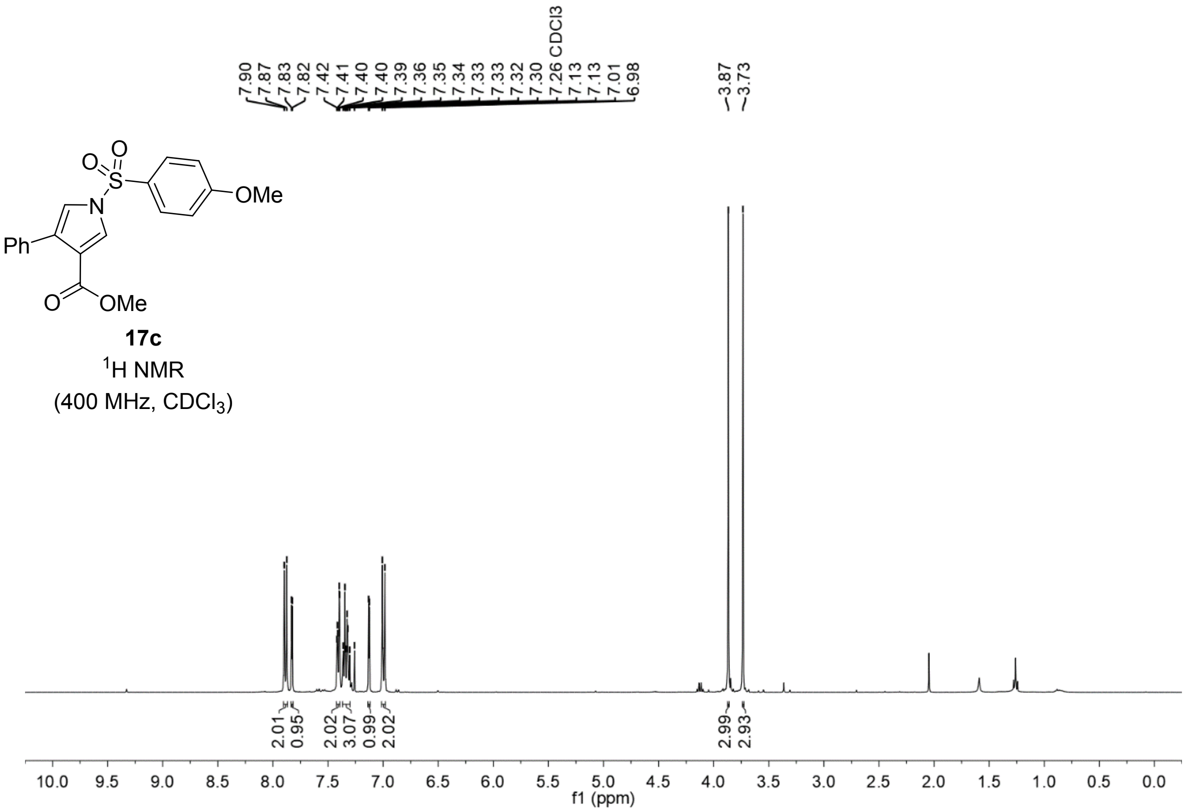

17c

^{13}C NMR

(101 MHz, $CDCl_3$)

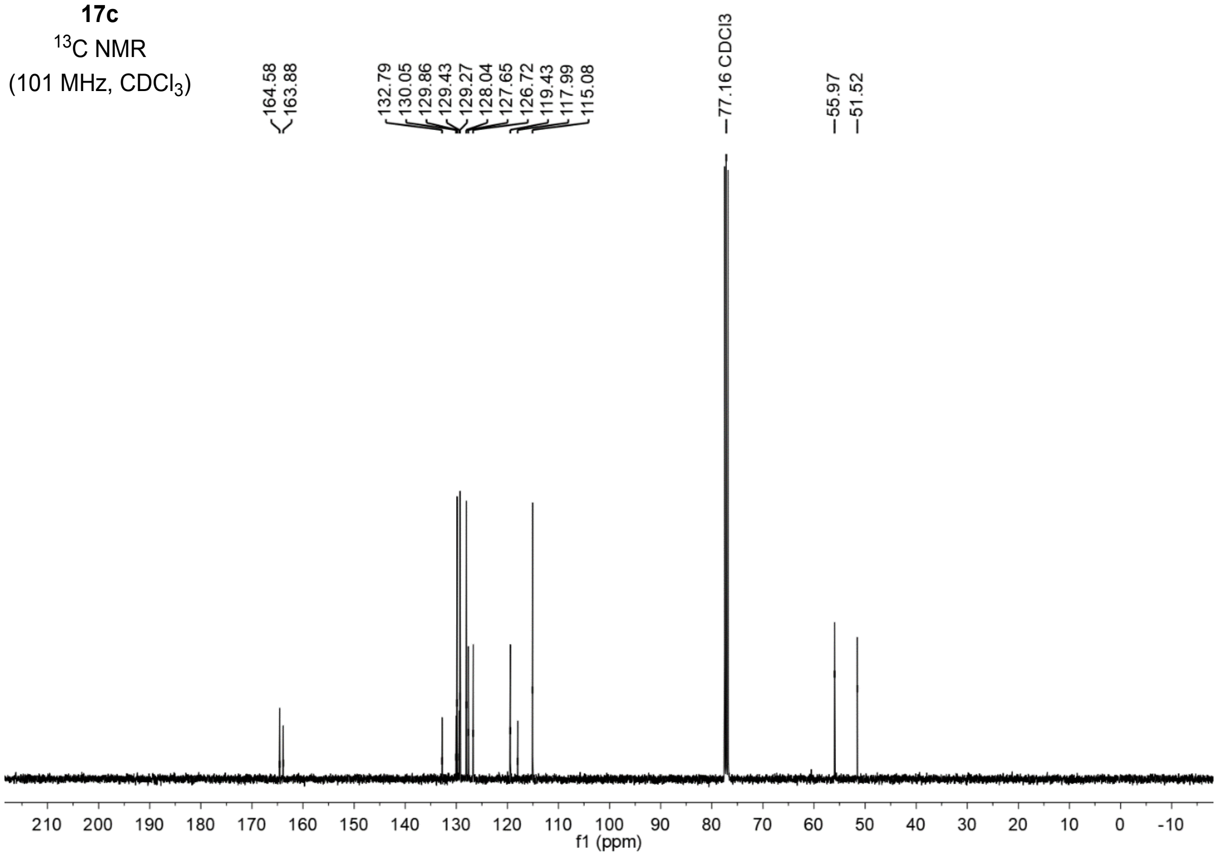

17d
^{1}H NMR
(400 MHz, $CDCl_3$)

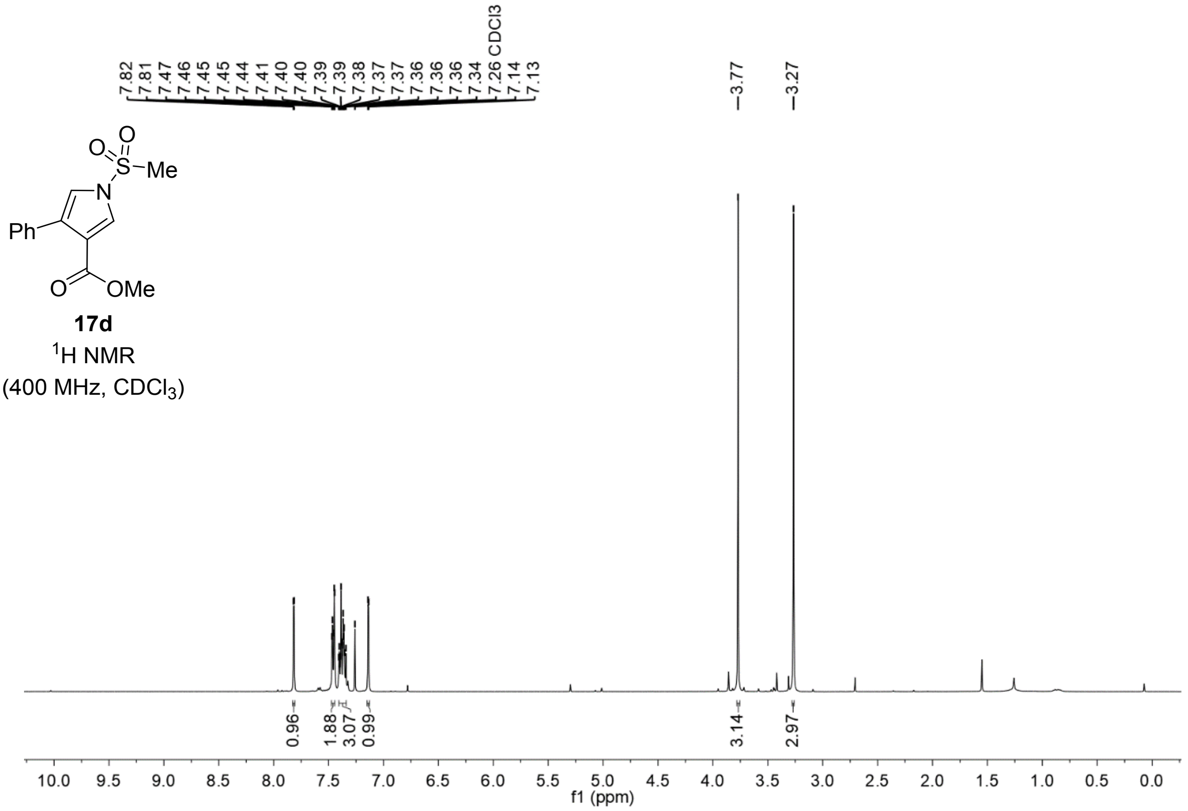

17d
^{13}C NMR
(101 MHz, $CDCl_3$)

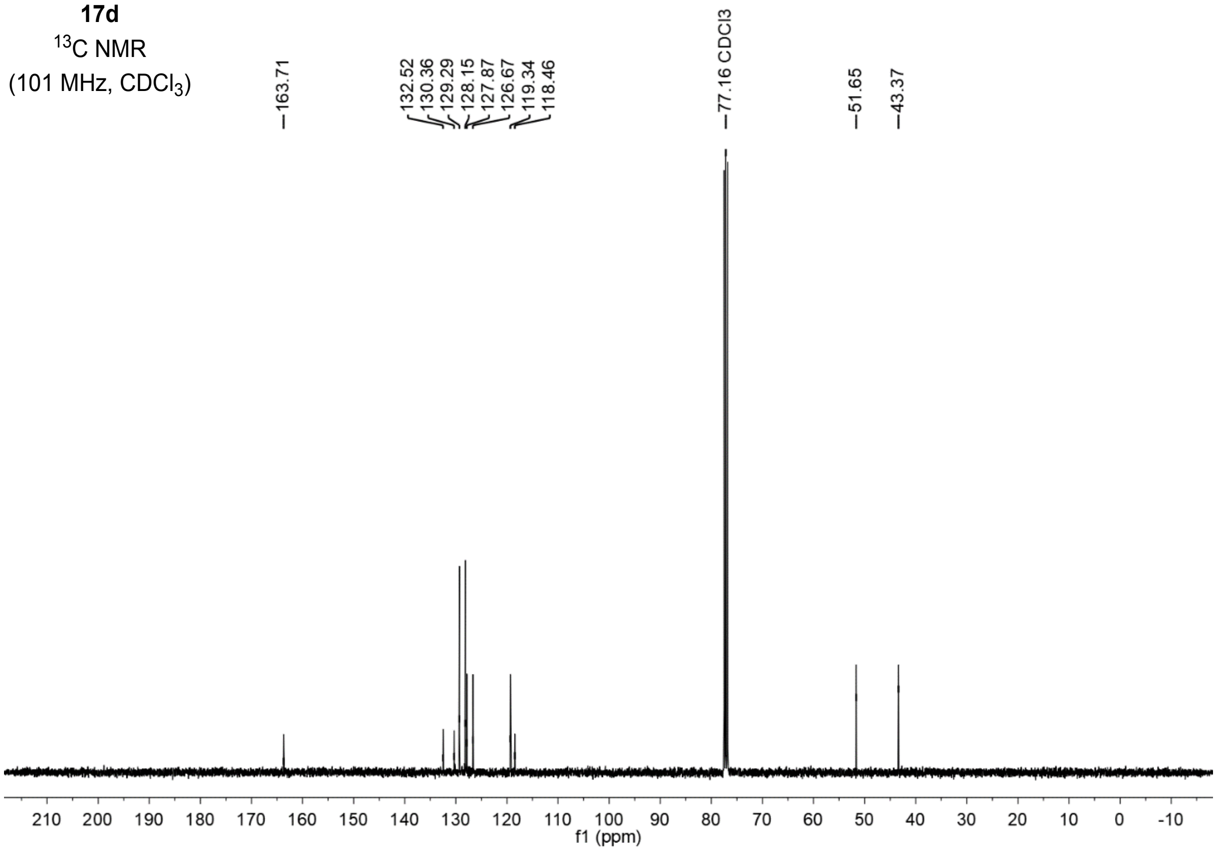

<

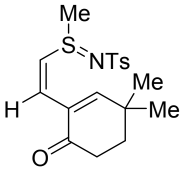

19

^{1}H NMR

(400 MHz, $CDCl_3$)

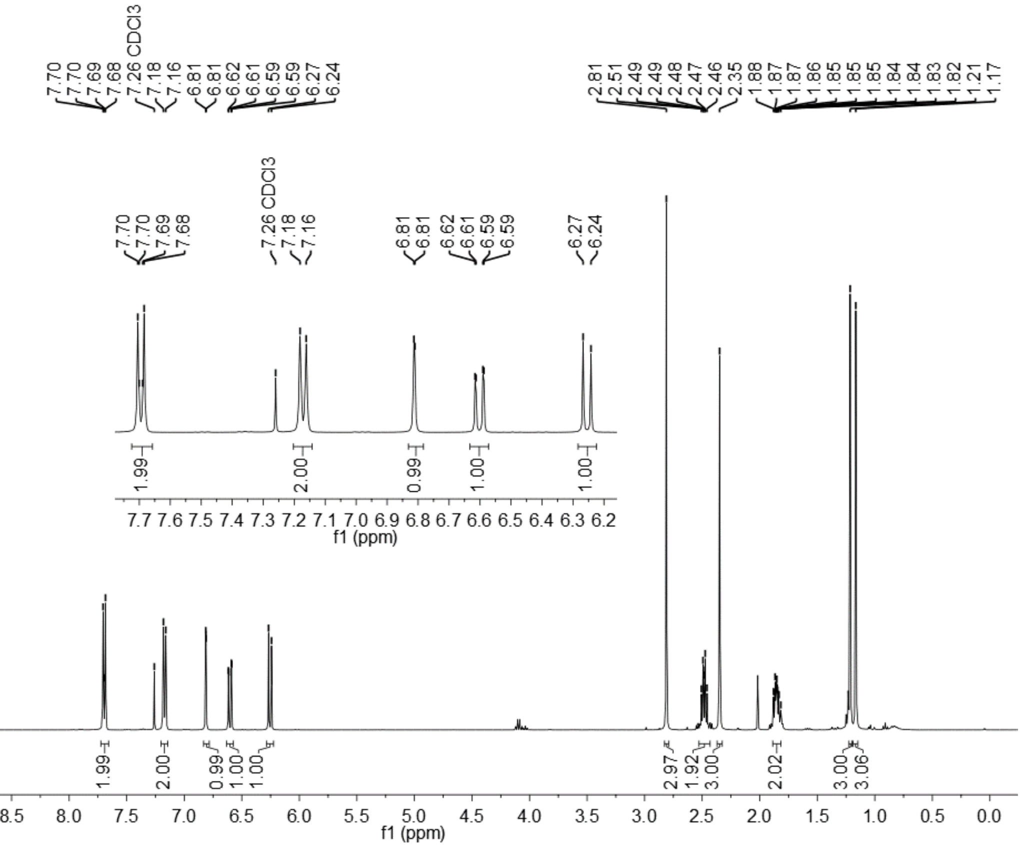

19

^{13}C NMR

(101 MHz, $CDCl_3$)

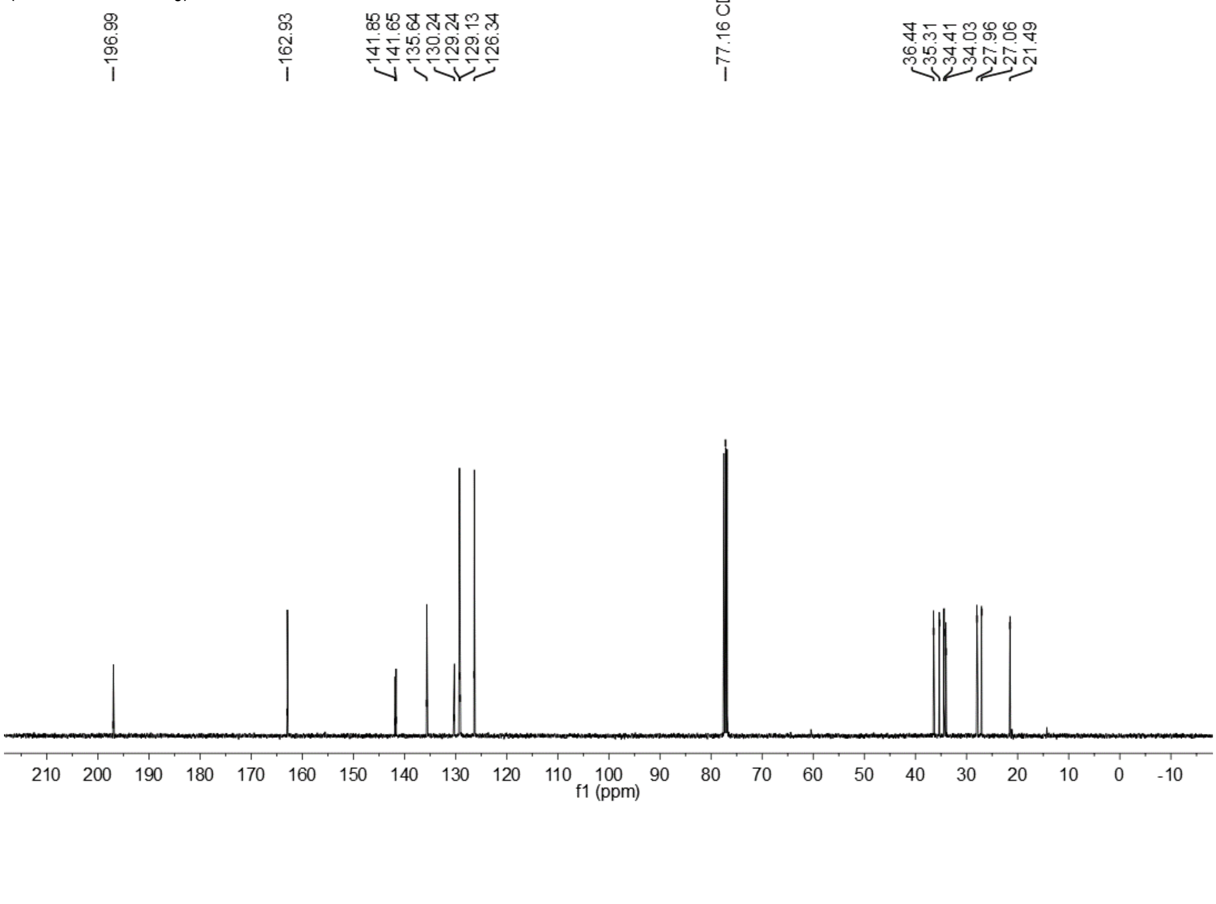

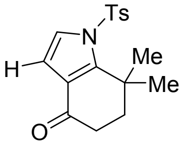

20
^{1}H NMR
(400 MHz, $CDCl_3$)

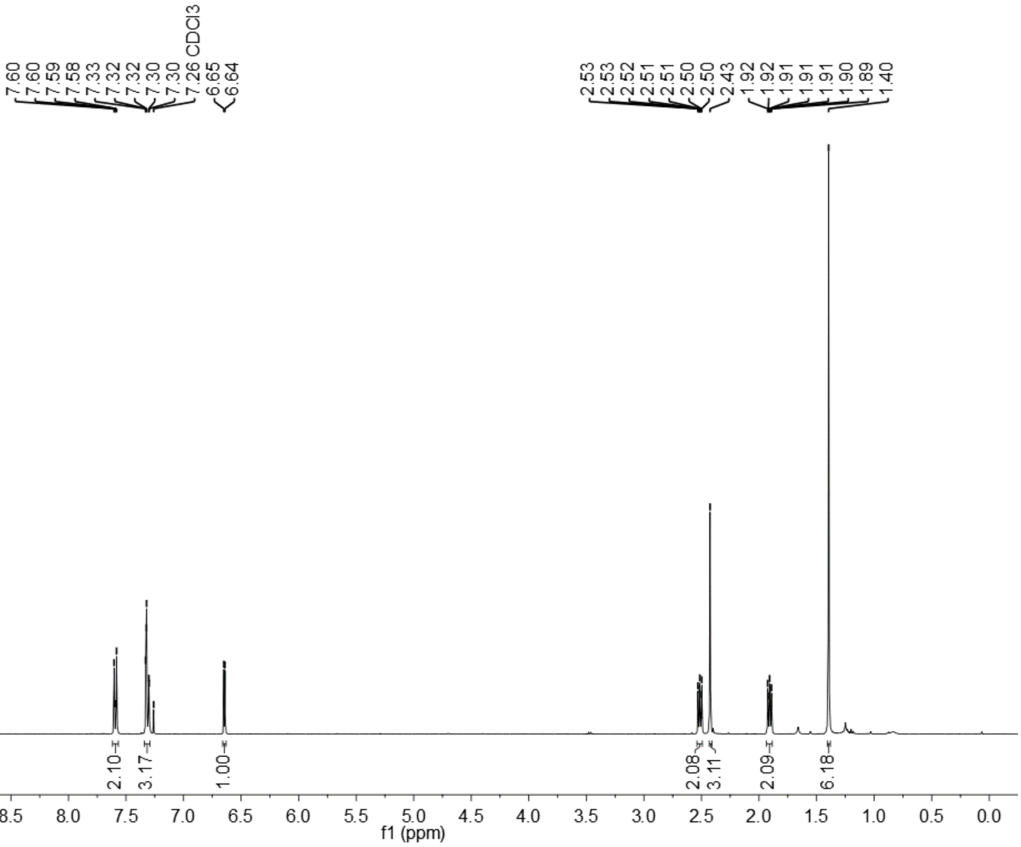

20
^{13}C NMR
(101 MHz, $CDCl_3$)

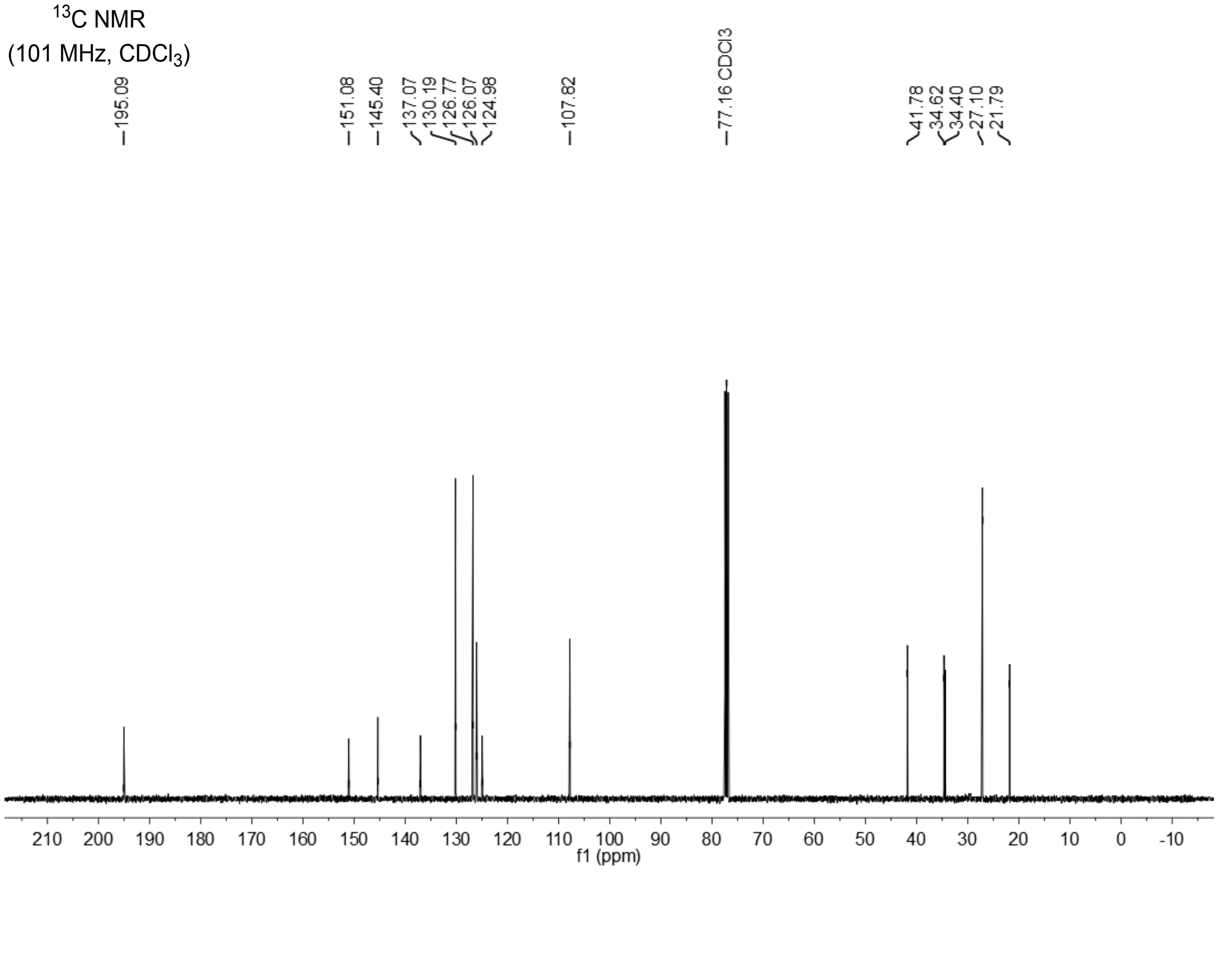

21
^{1}H NMR
(400 MHz, $CDCl_3$)

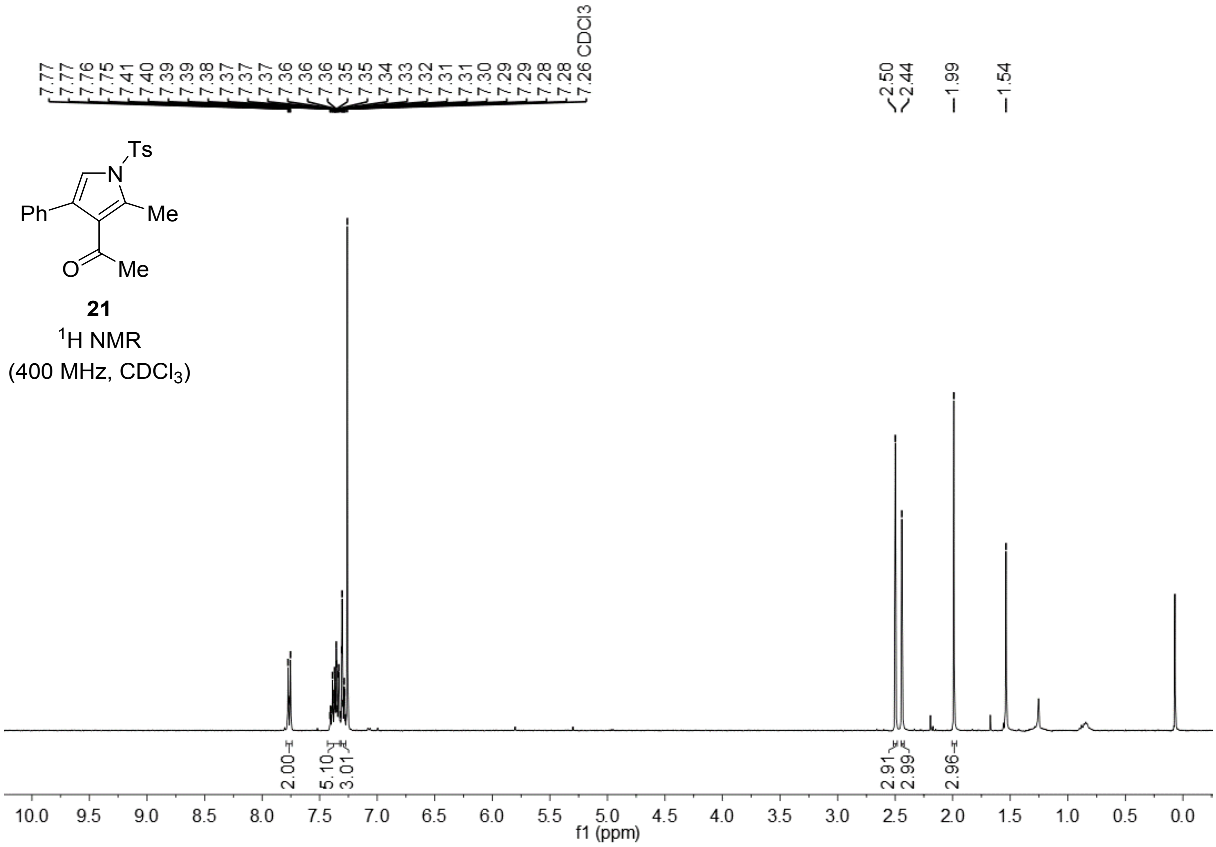

21
^{13}C NMR
(101 MHz, $CDCl_3$)

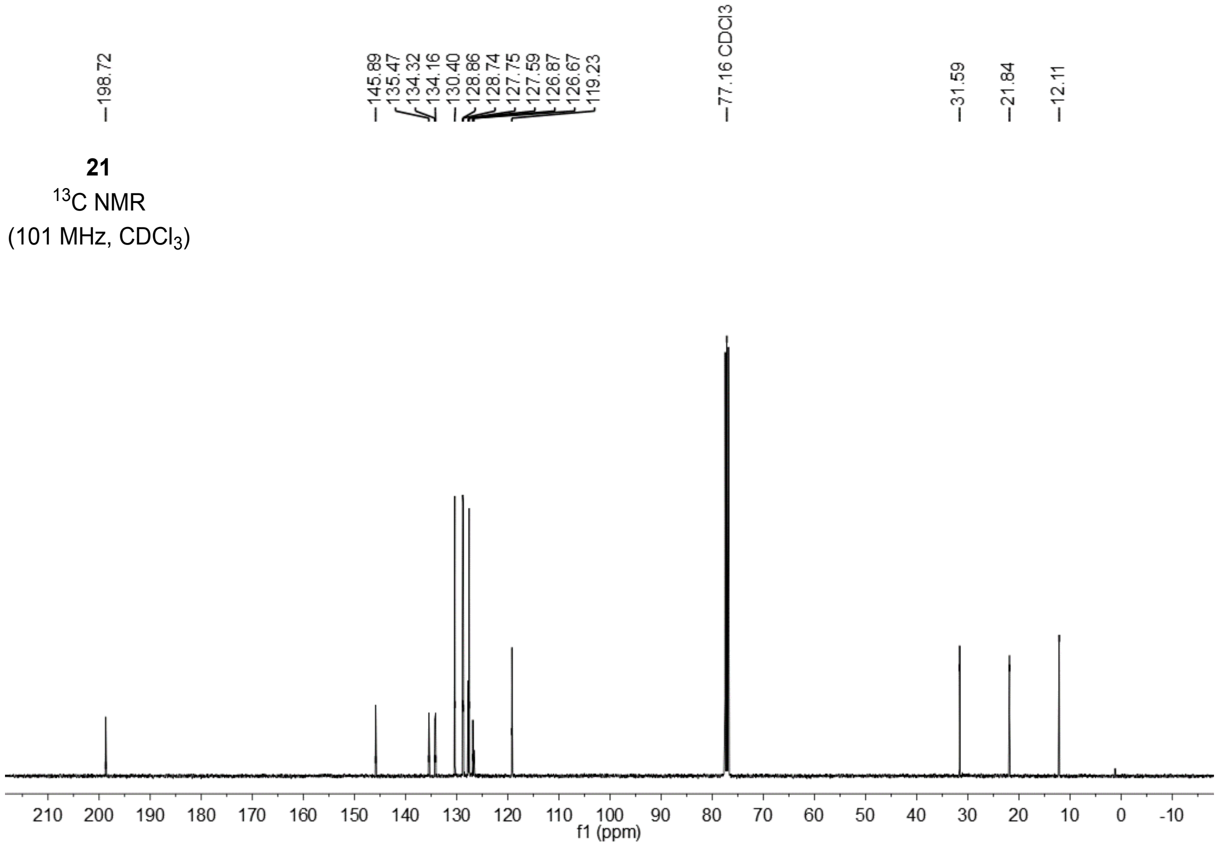

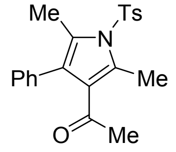

22

^{1}H NMR

(400 MHz, $CDCl_3$)

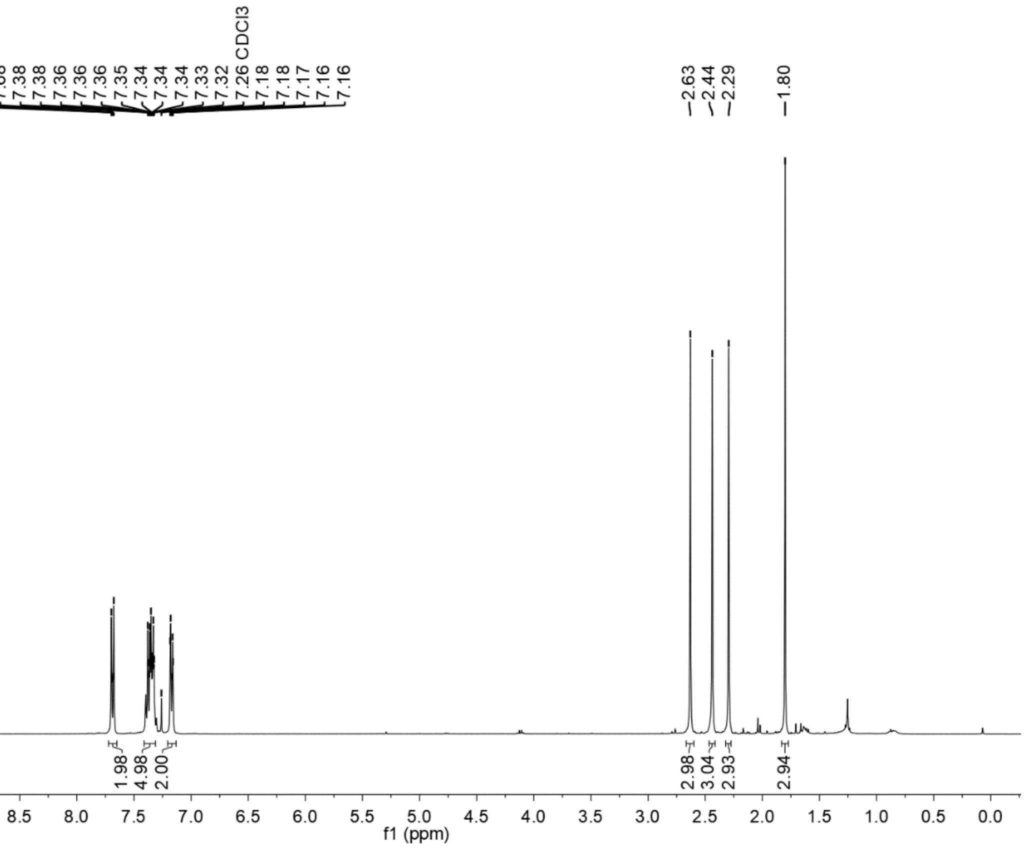

22

^{13}C NMR

(101 MHz, $CDCl_3$)

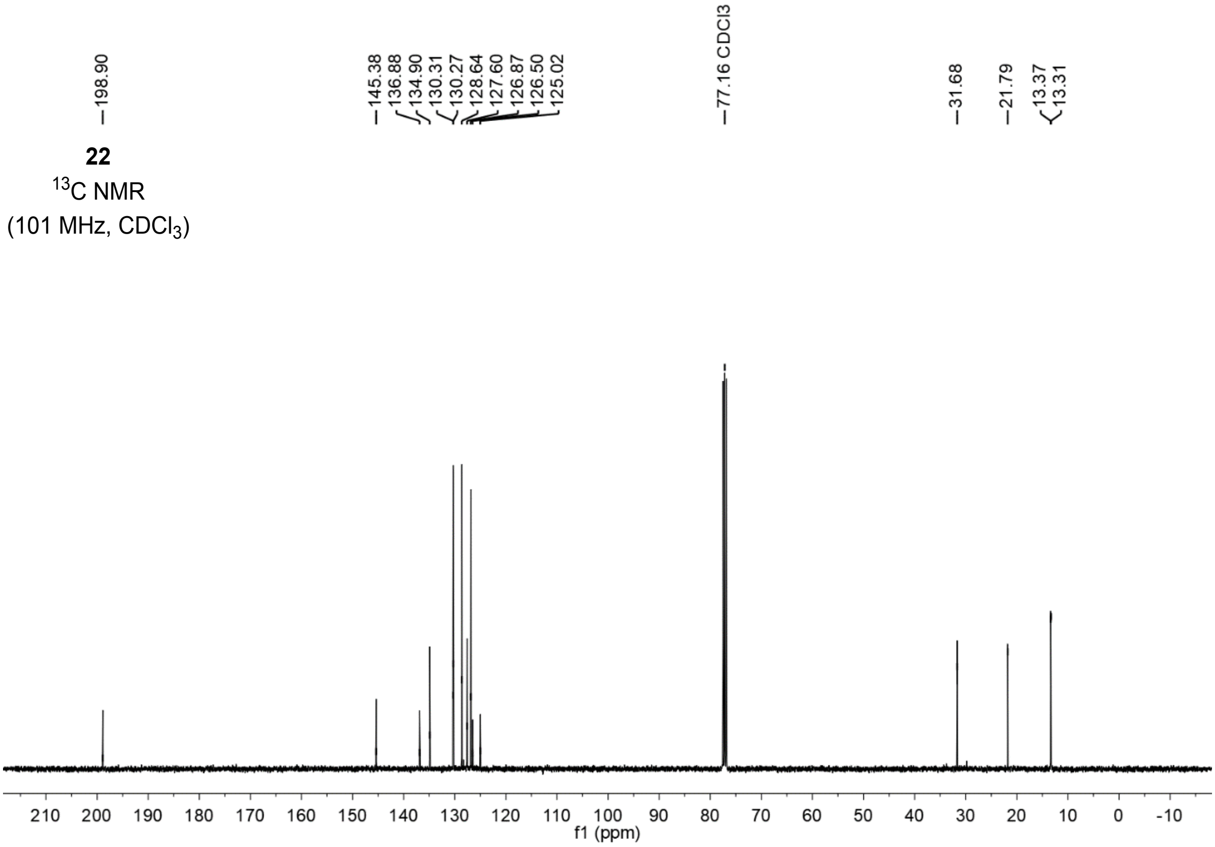

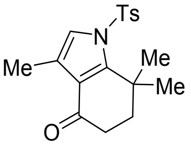

23
^{1}H NMR
(400 MHz, $CDCl_3$)

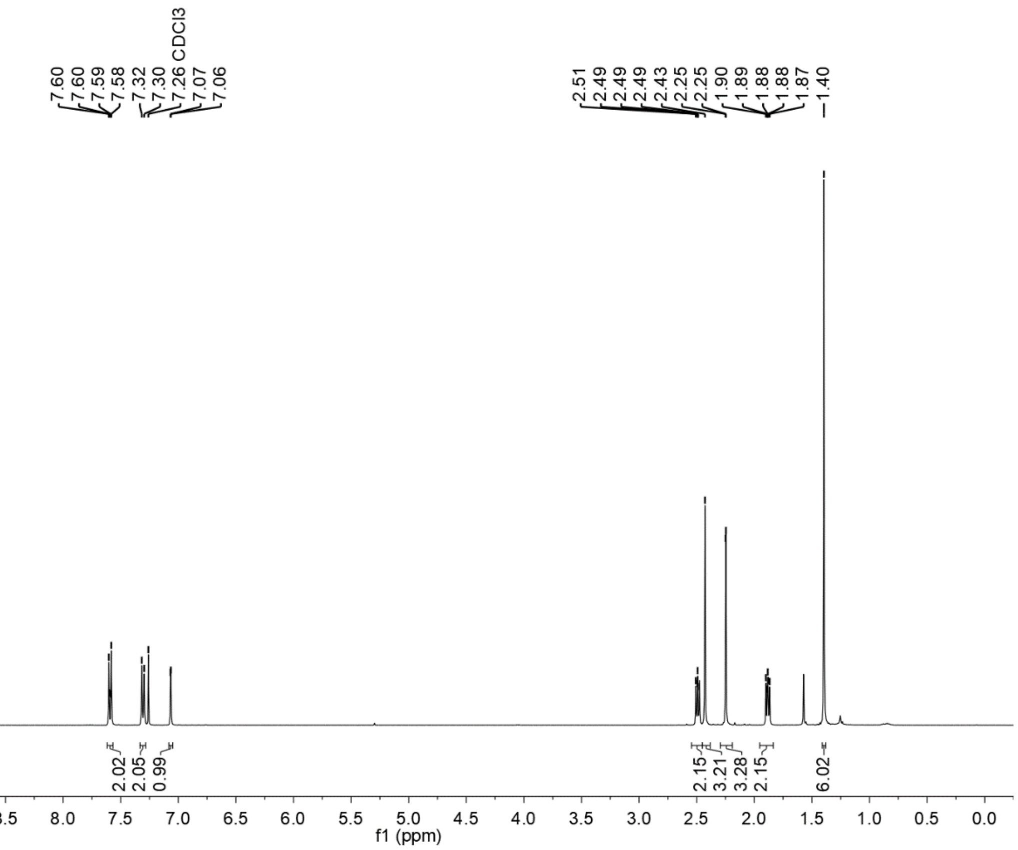

23
^{13}C NMR
(101 MHz, $CDCl_3$)

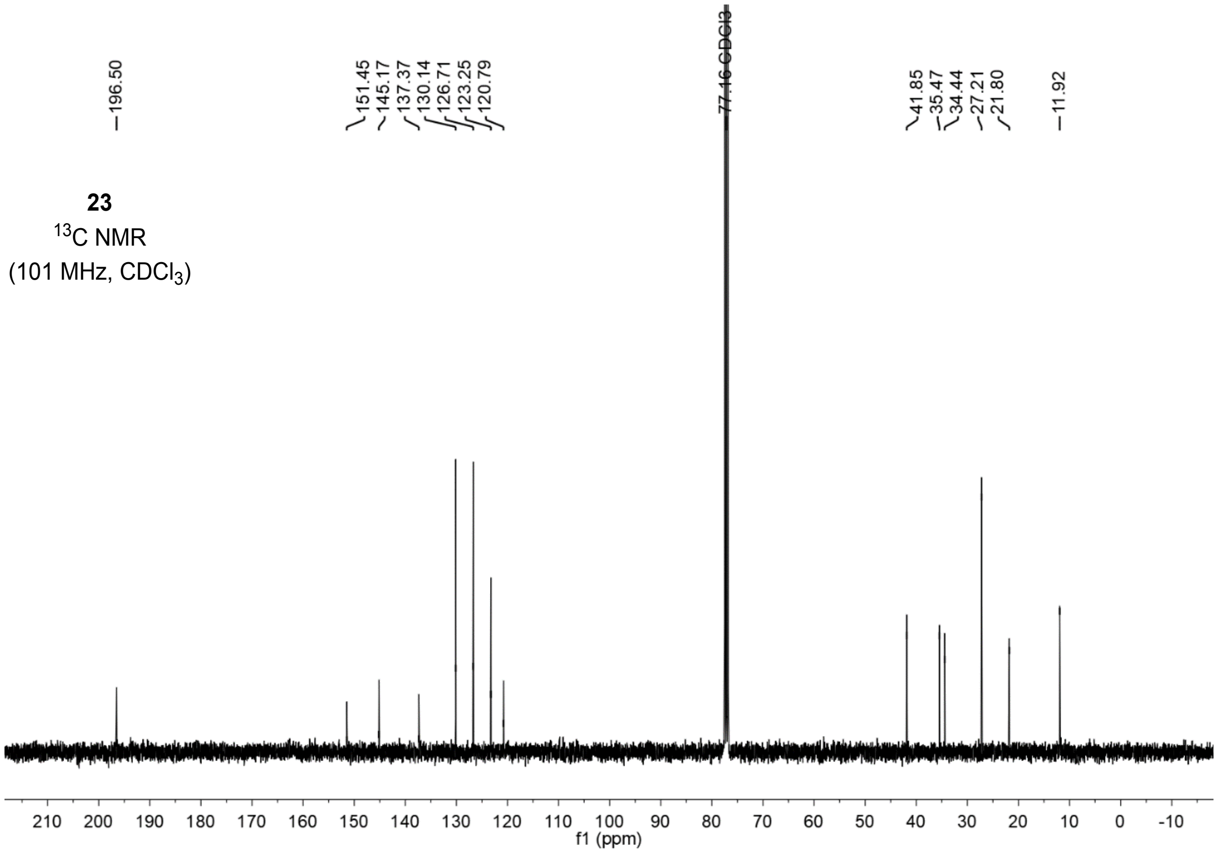

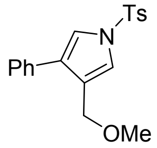

24
^{1}H NMR
(400 MHz, $CDCl_3$)

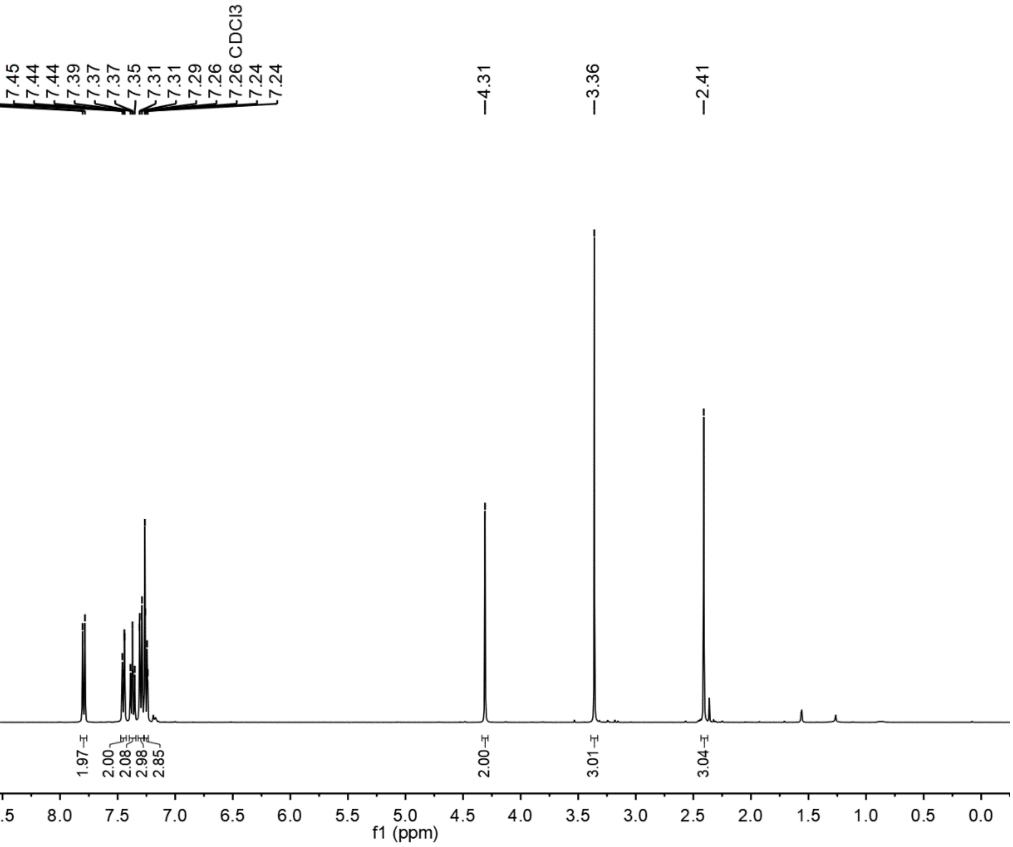

24
^{13}C NMR
(101 MHz, $CDCl_3$)

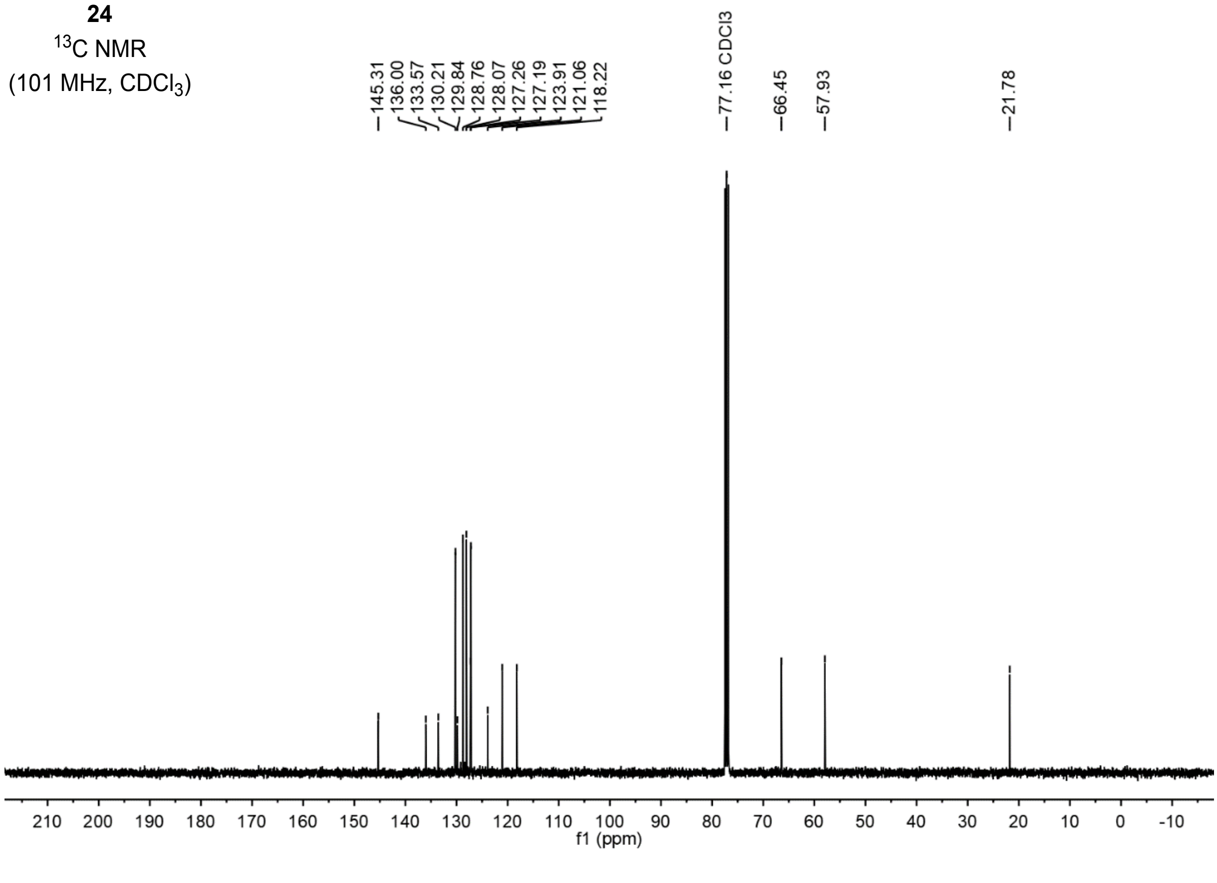

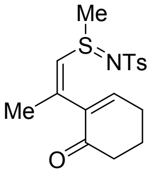

25

^{1}H NMR

(400 MHz, $CDCl_3$)

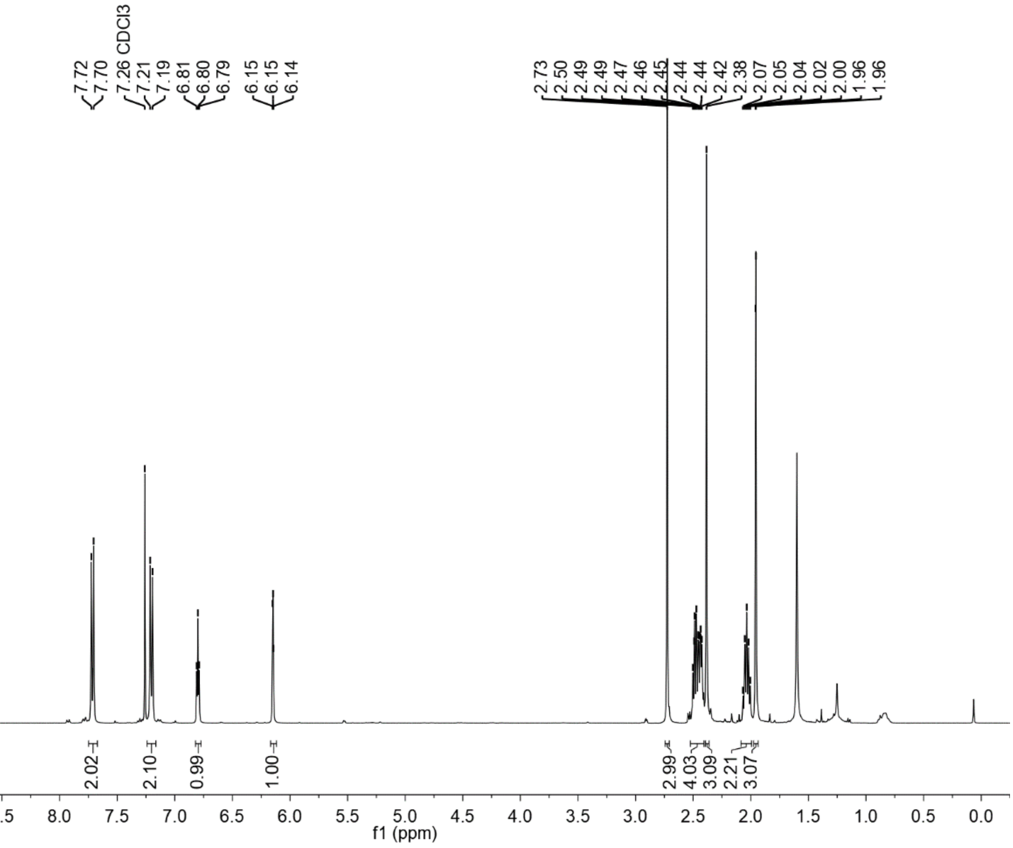

25

^{13}C NMR

(101 MHz, $CDCl_3$)

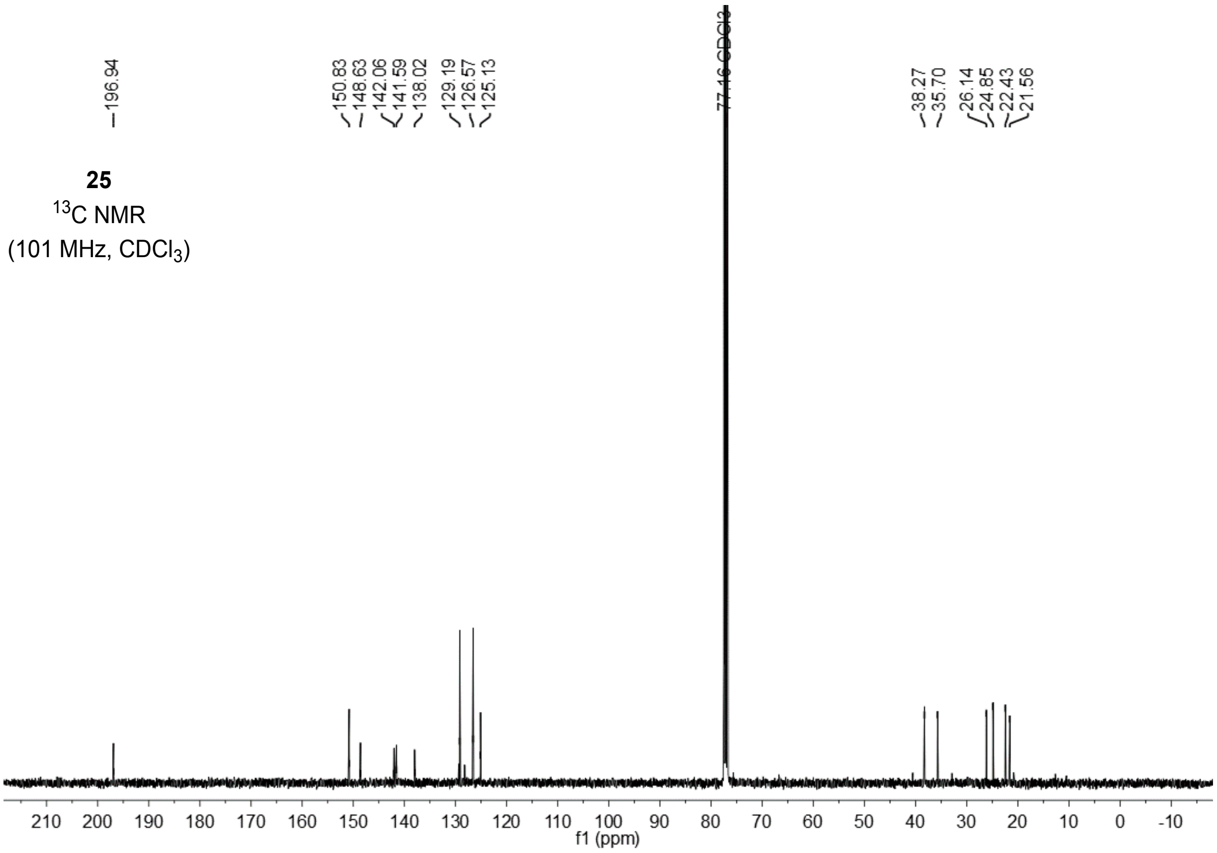

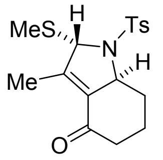

26
^{1}H NMR
(400 MHz, $CDCl_3$)

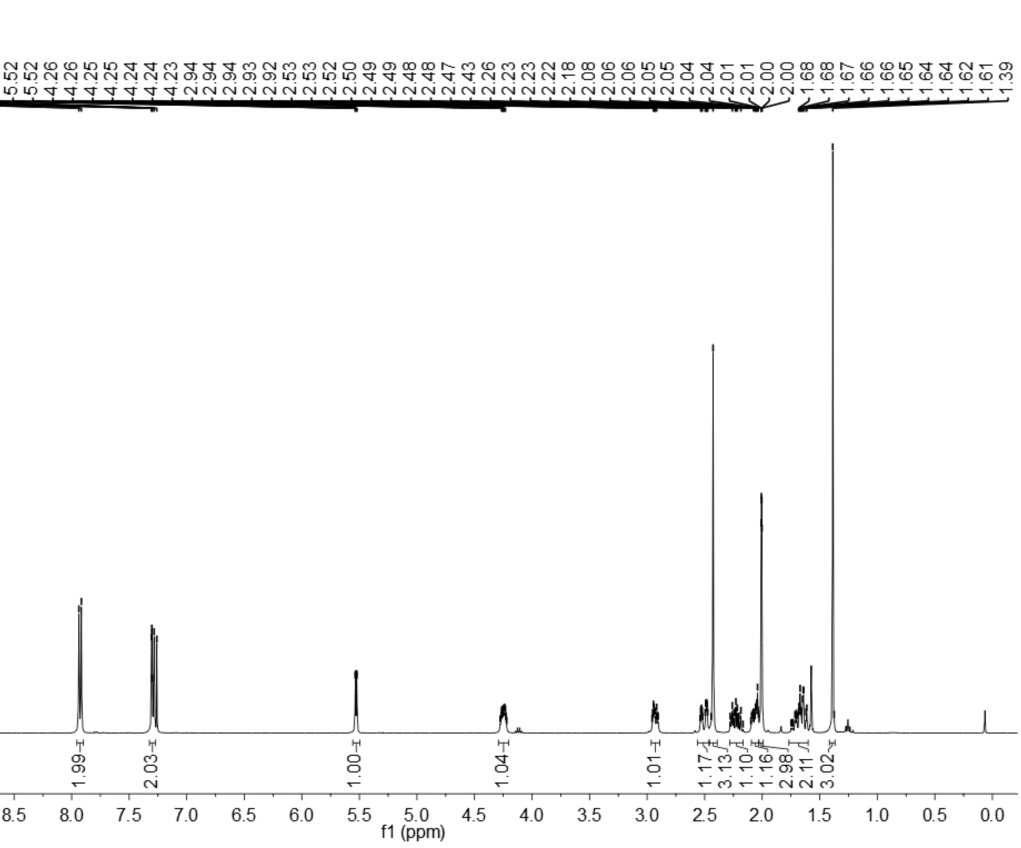

26
^{13}C NMR
(101 MHz, $CDCl_3$)

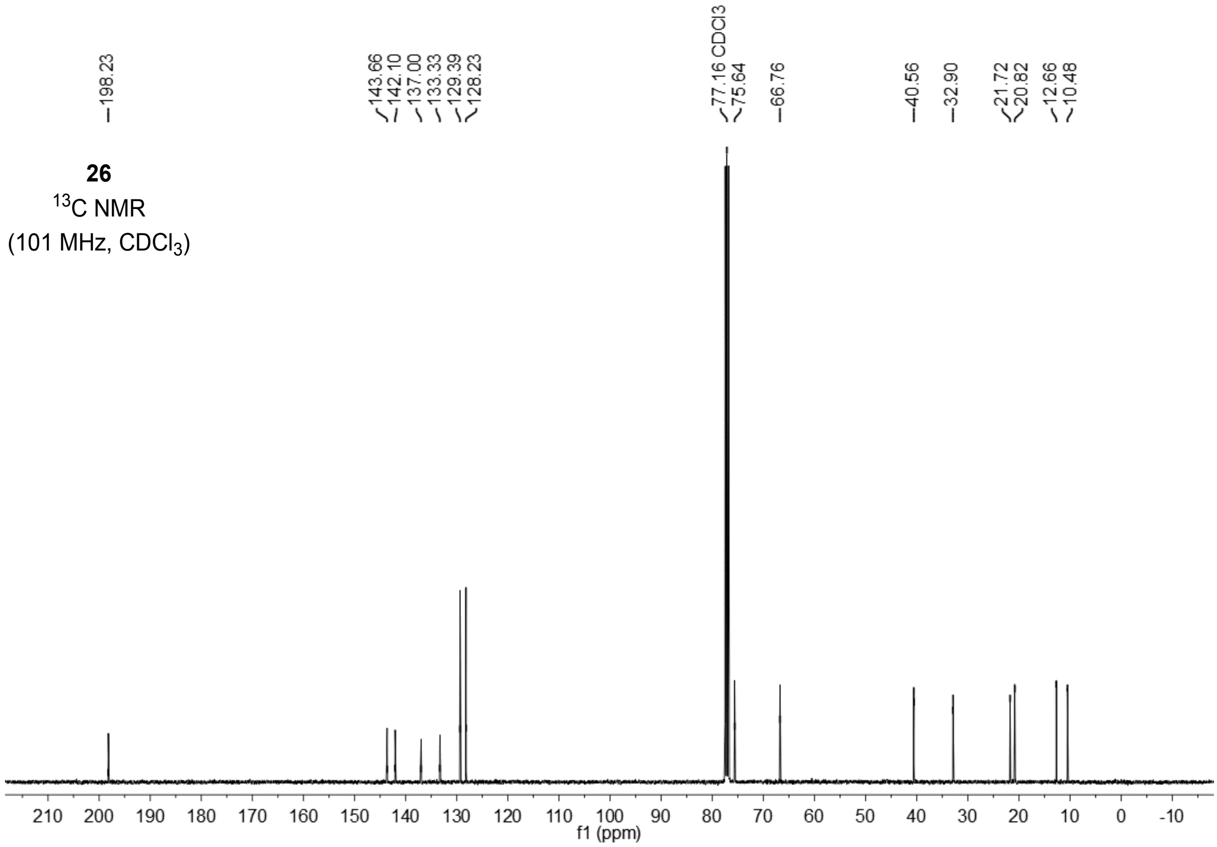

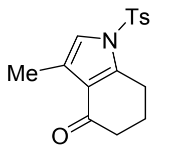

27
^{1}H NMR
(400 MHz, $CDCl_3$)

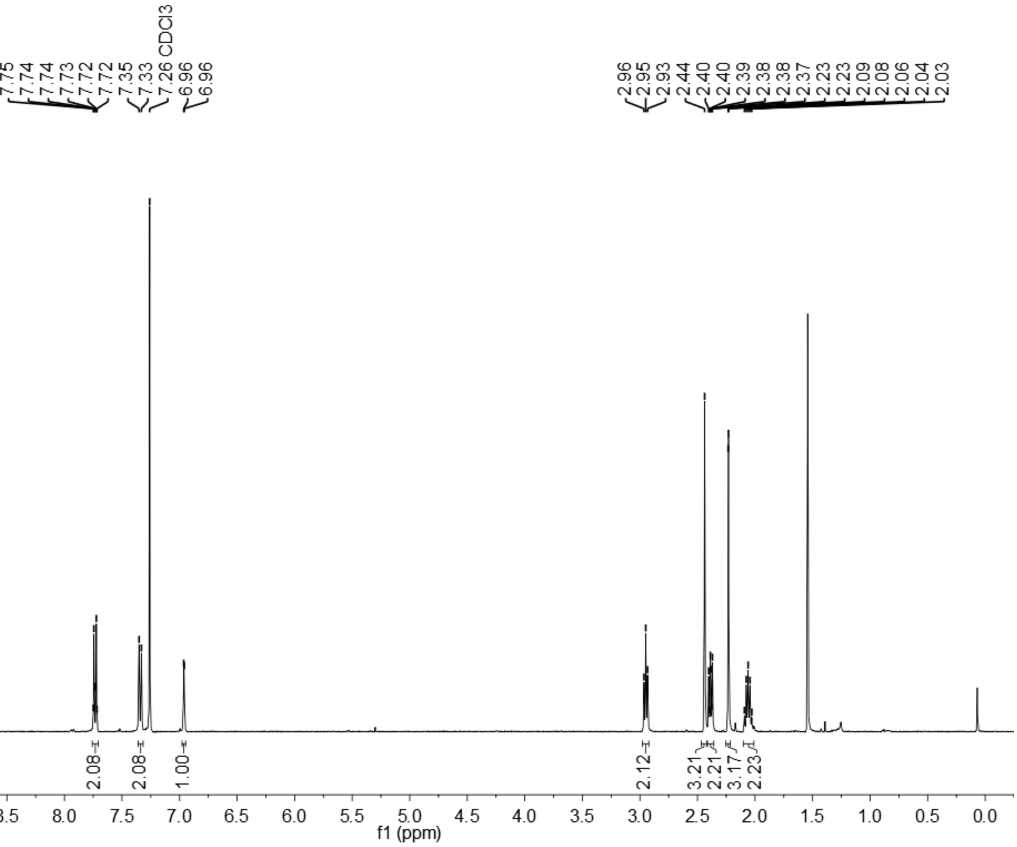

27
^{13}C NMR
(101 MHz, $CDCl_3$)

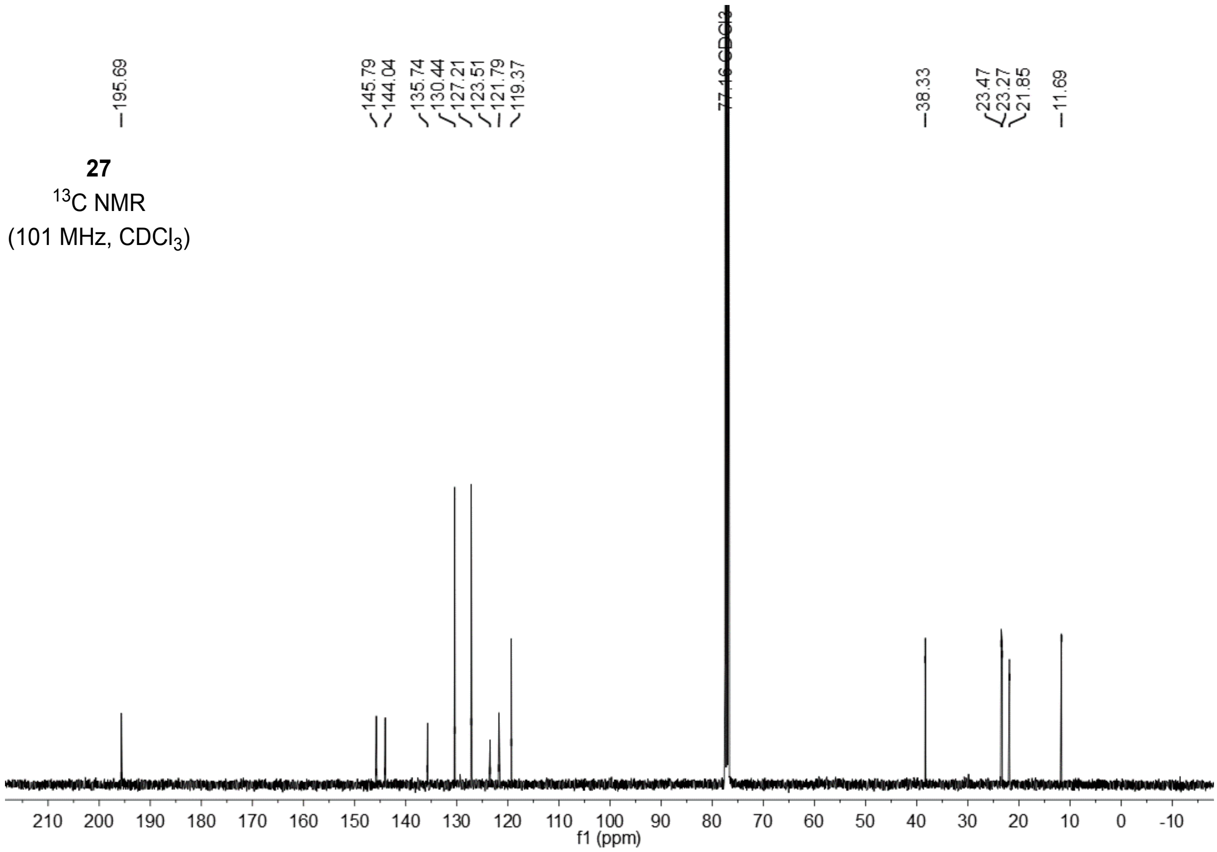

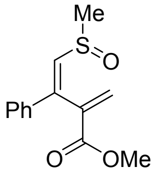

28

^{1}H NMR

(400 MHz, $CDCl_3$)

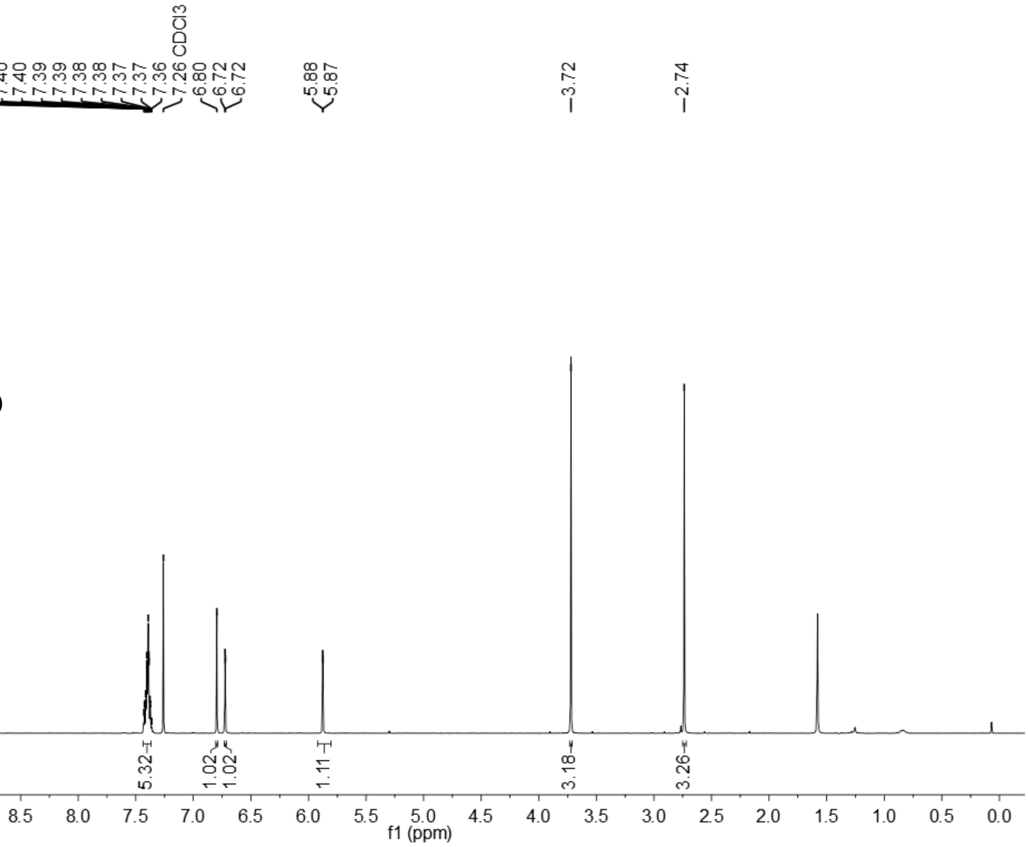

28

^{13}C NMR

(101 MHz, $CDCl_3$)

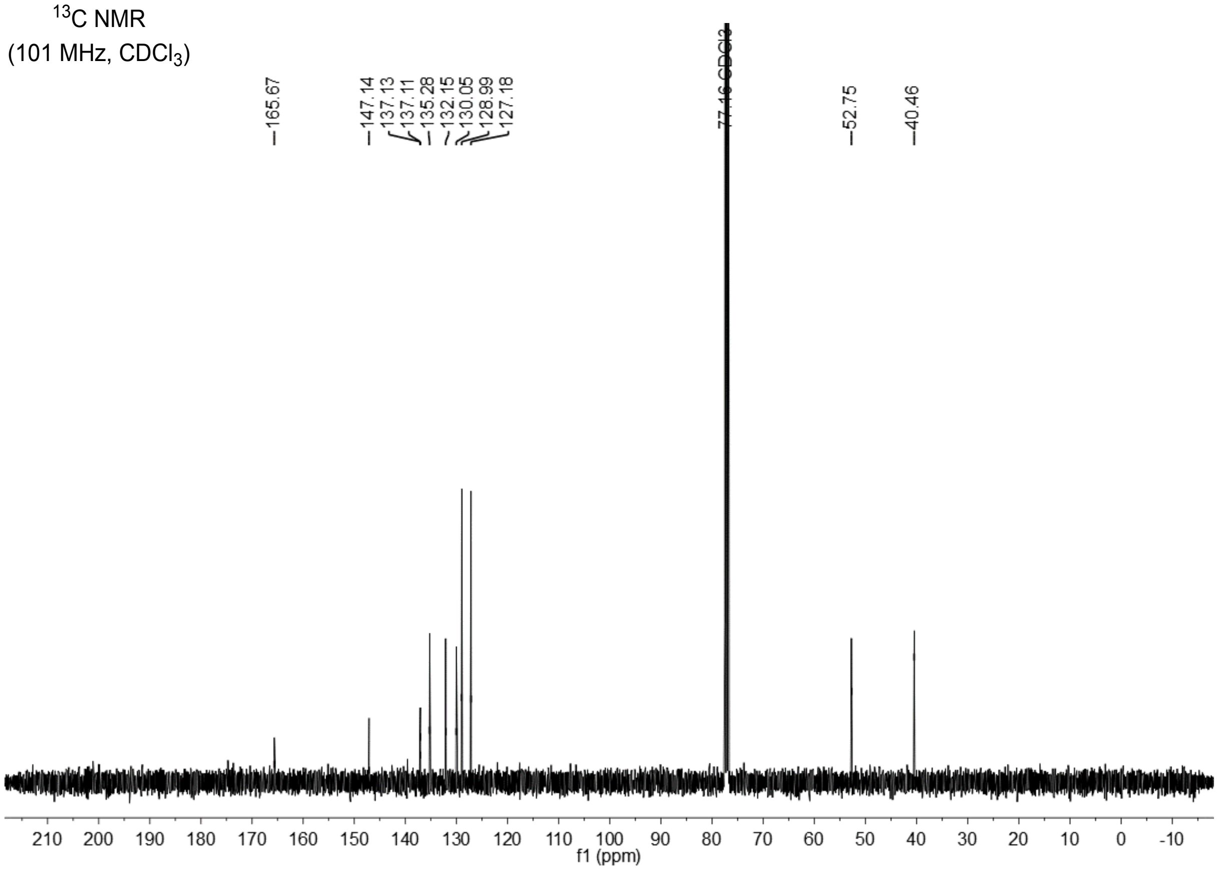

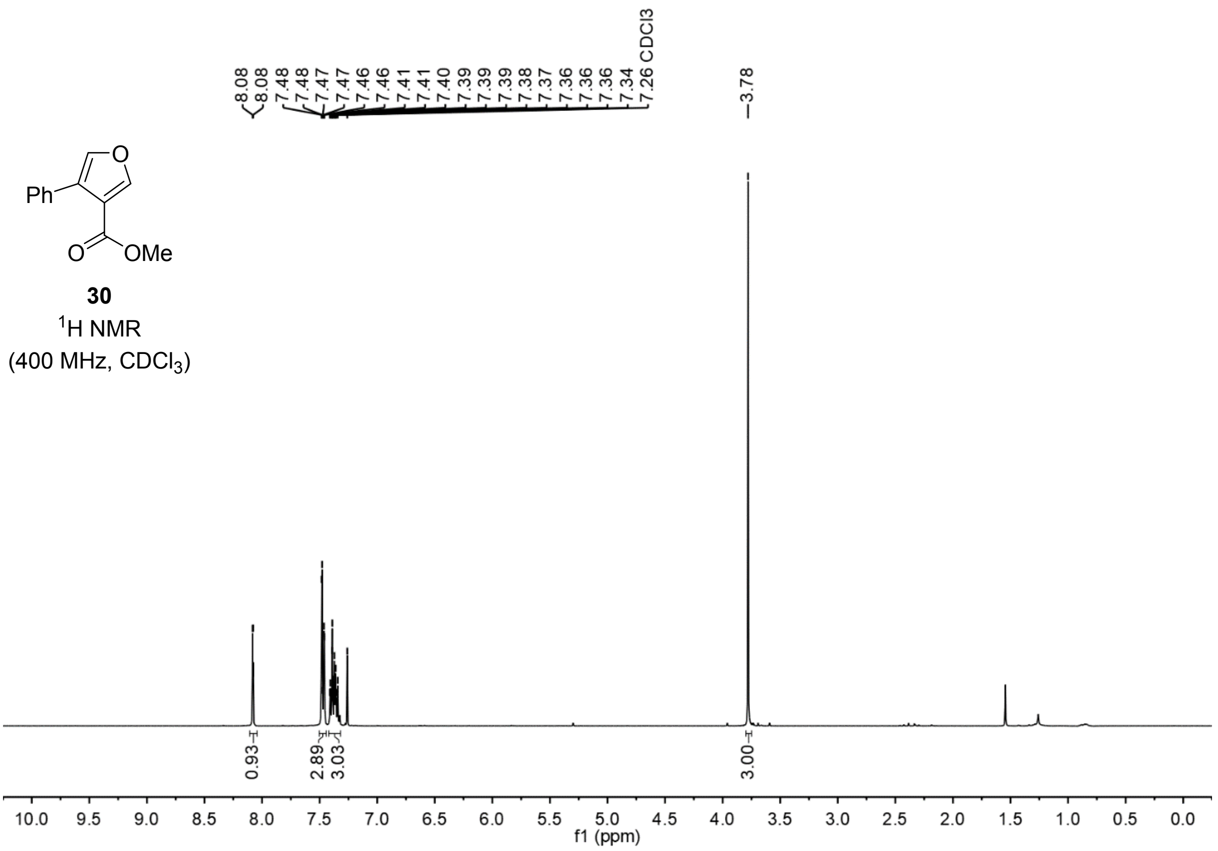

30
^{1}H NMR
(400 MHz, $CDCl_3$)

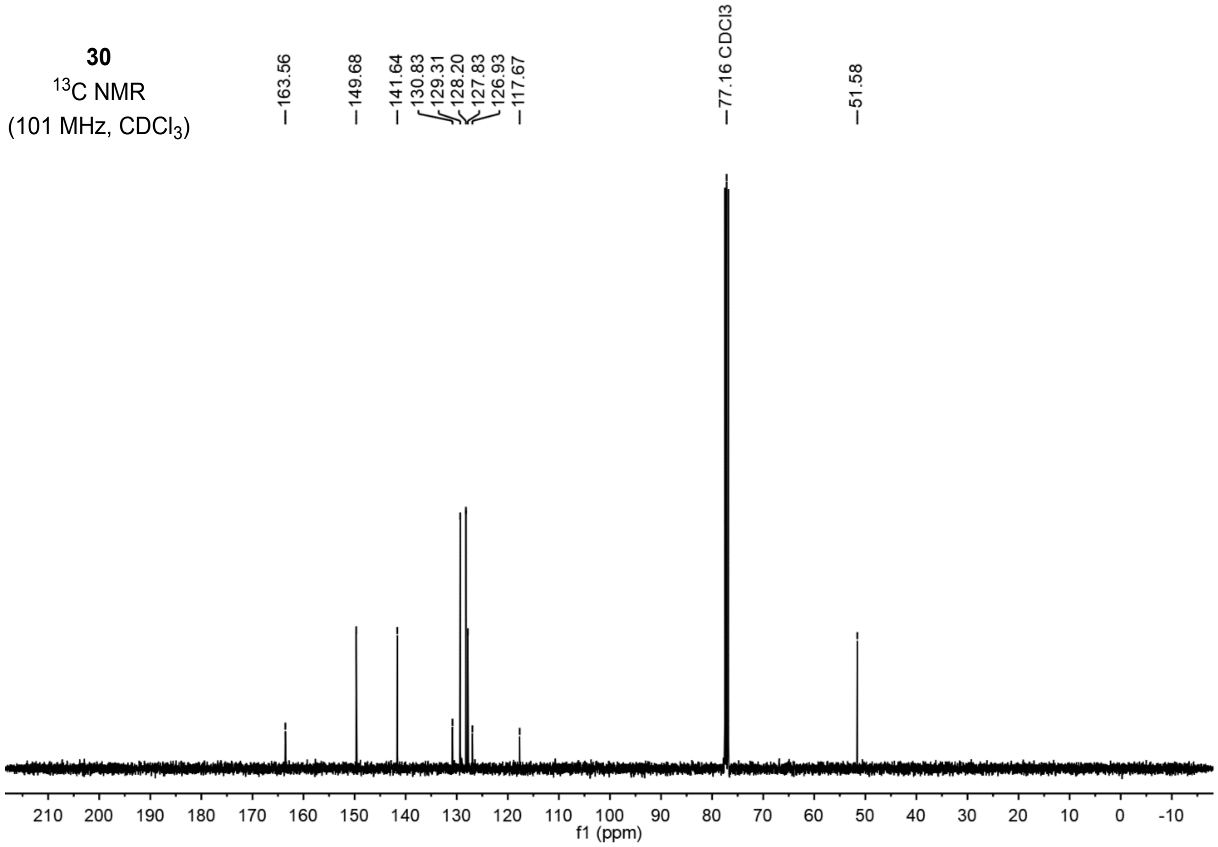

30
^{13}C NMR
(101 MHz, $CDCl_3$)

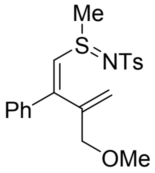

32
^{1}H NMR
(400 MHz, $CDCl_3$)

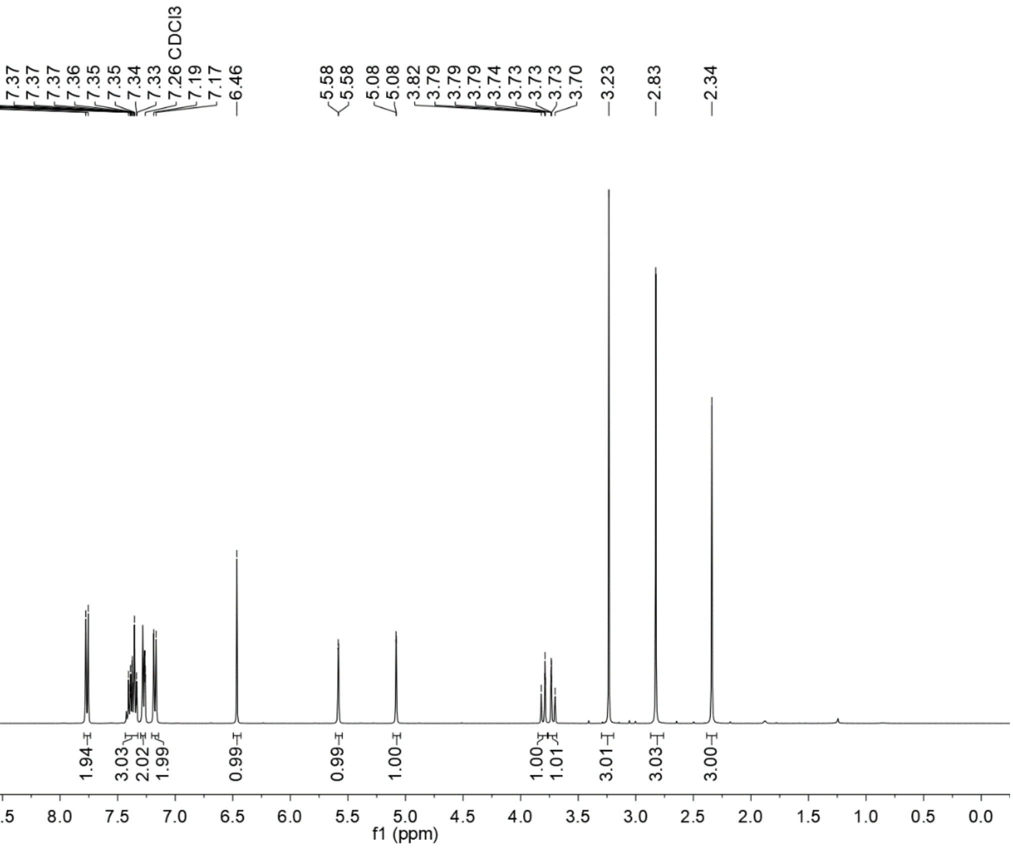

32
^{13}C NMR
(101 MHz, $CDCl_3$)

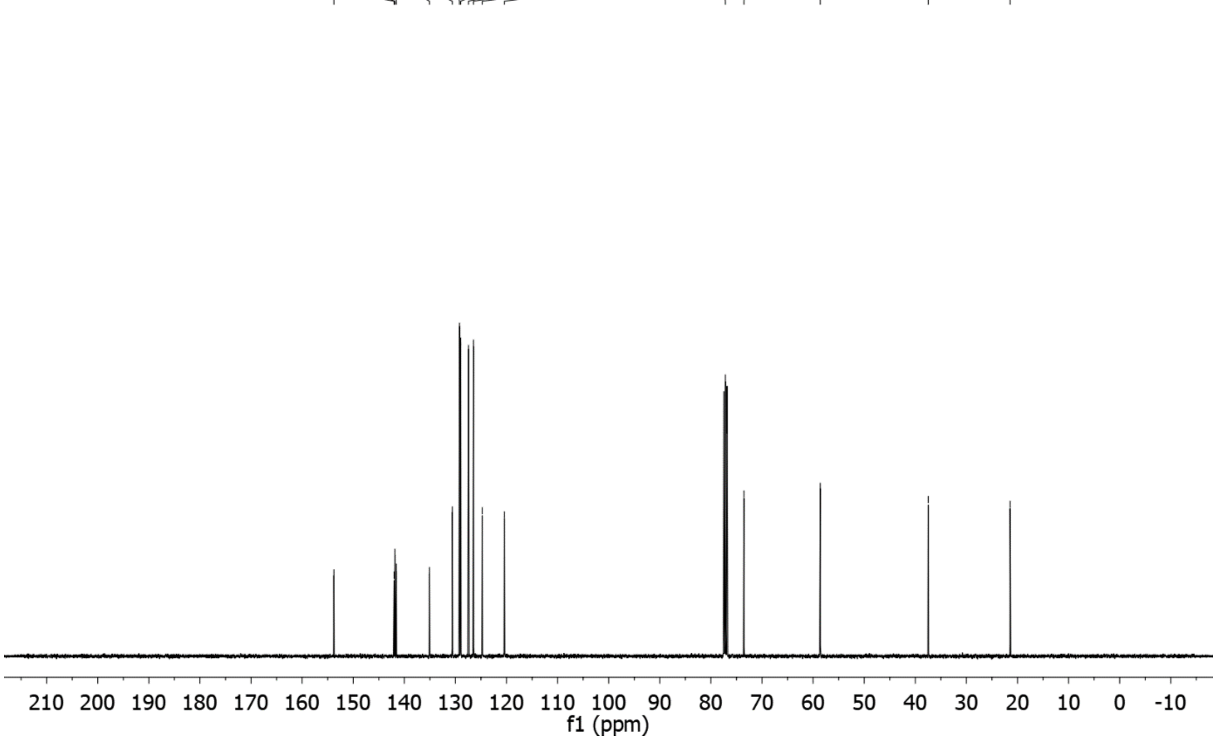

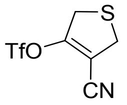

S-4
^{1}H NMR
(400 MHz, $CDCl_3$)

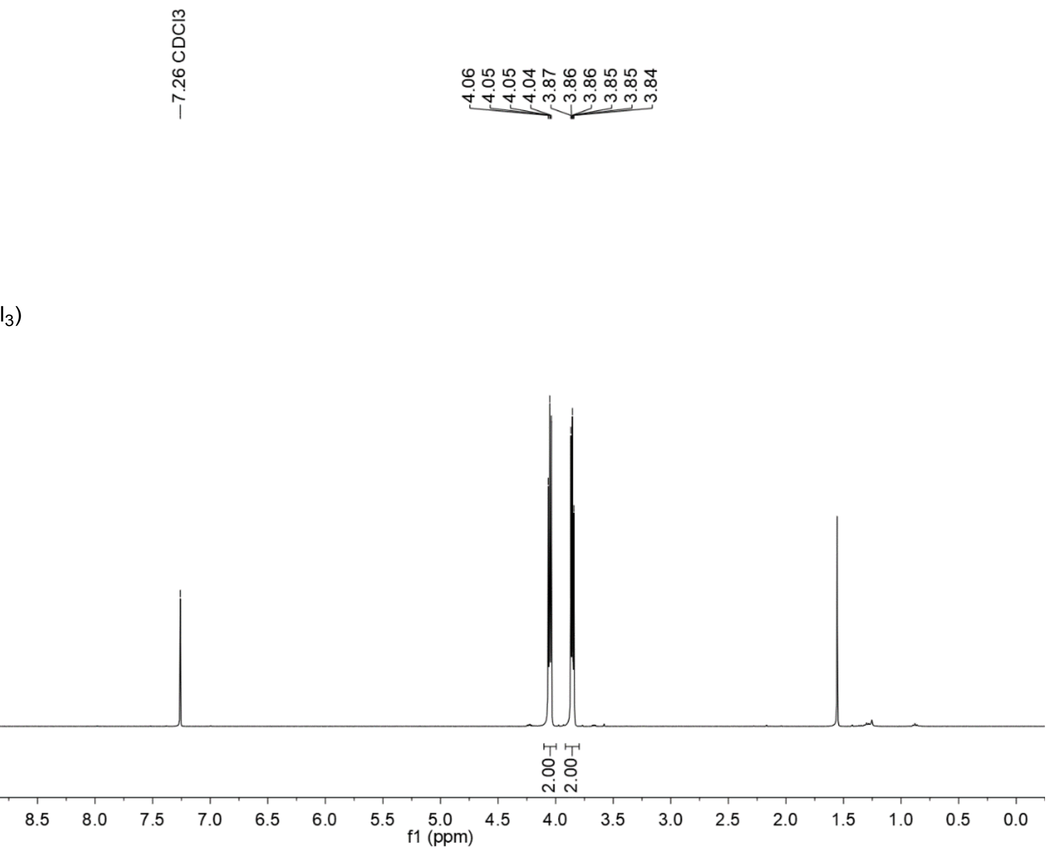

S-4
^{13}C NMR
(101 MHz, $CDCl_3$)

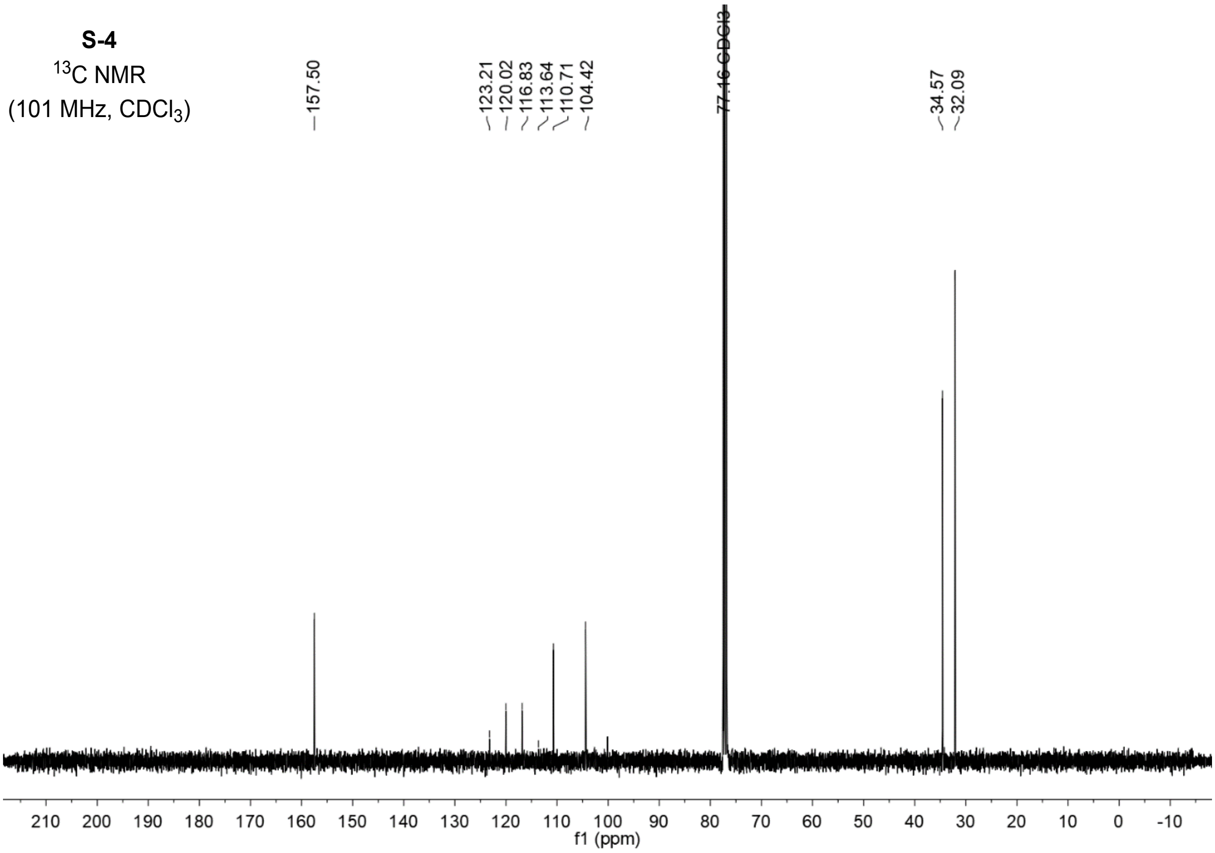

S-4
^{19}F NMR
(376 MHz, $CDCl_3$)

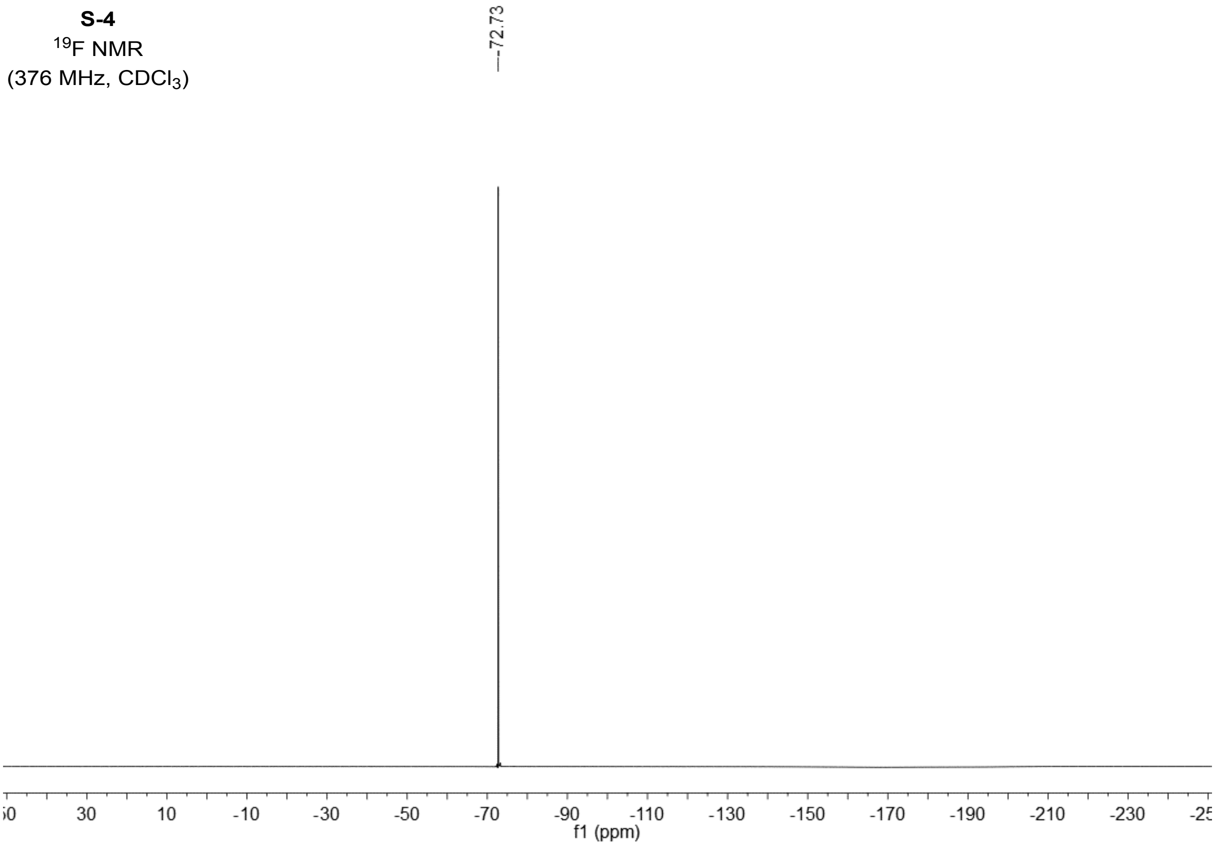

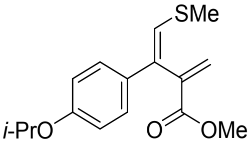

S-10
^{1}H NMR
(400 MHz, $CDCl_3$)

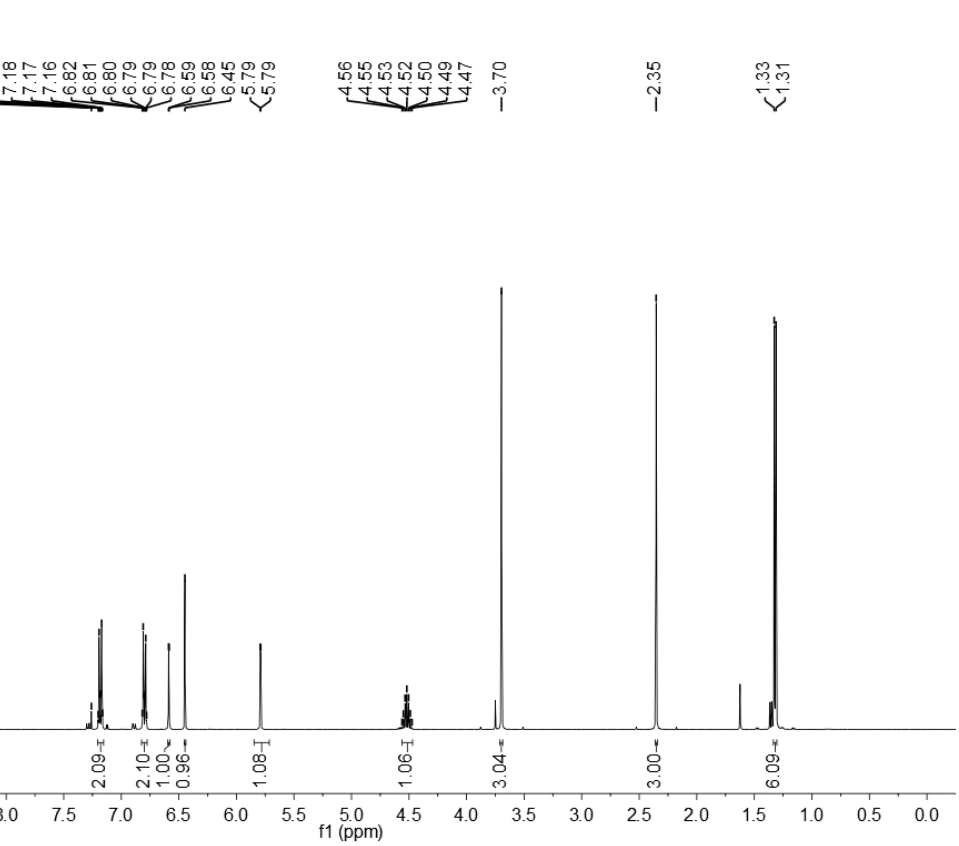

S-10
^{13}C NMR
(101 MHz, $CDCl_3$)

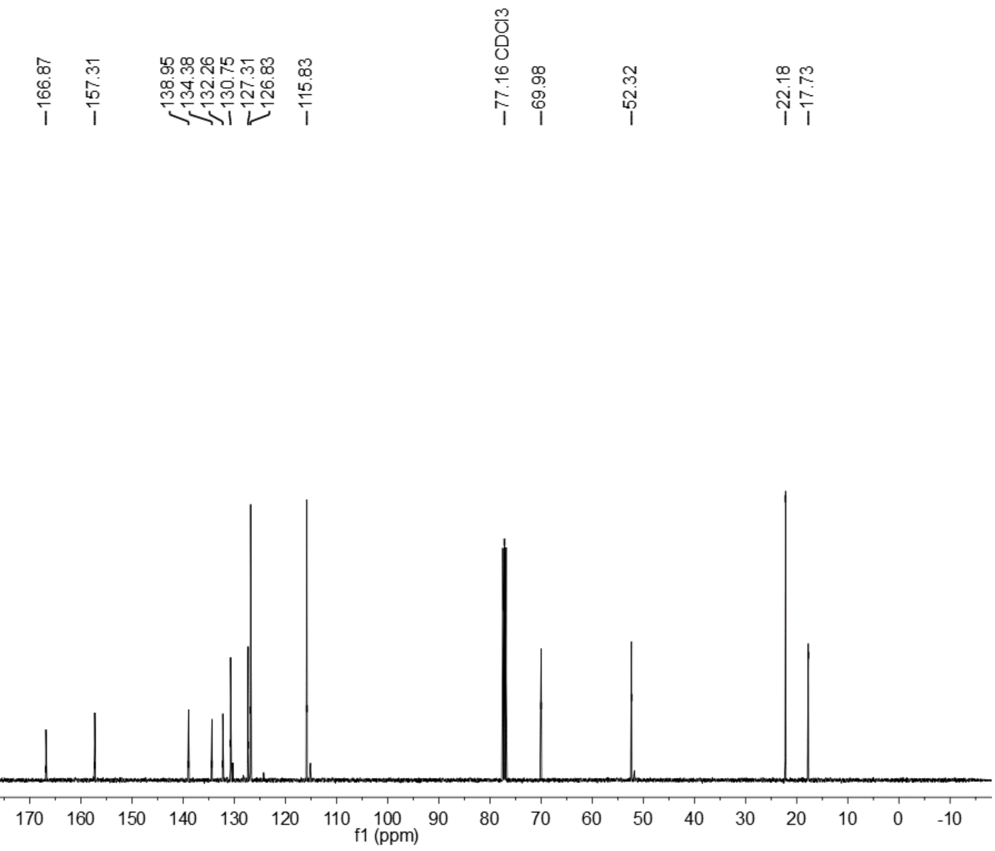

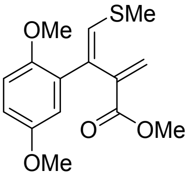

S-11
^{1}H NMR
(400 MHz, $CDCl_3$)

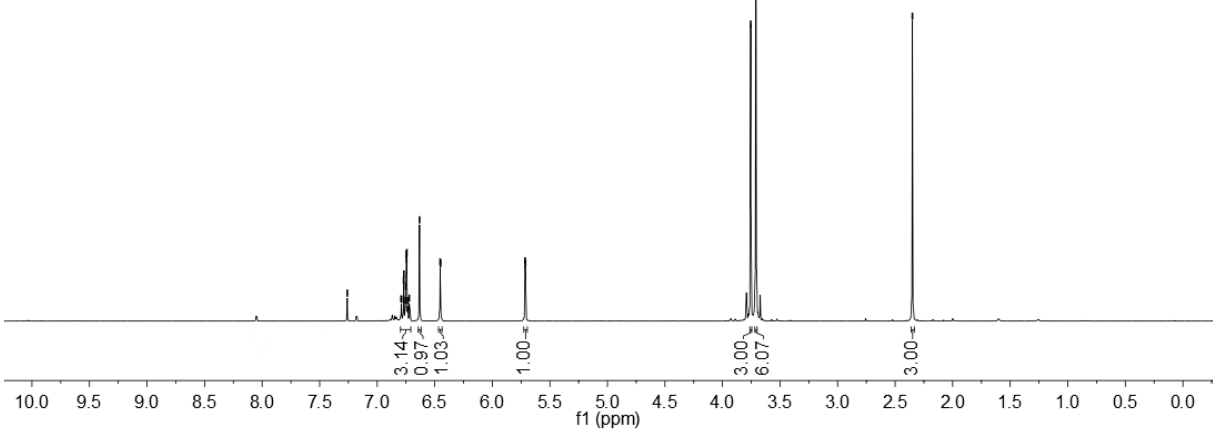

S-11
^{13}C NMR
(101 MHz, $CDCl_3$)

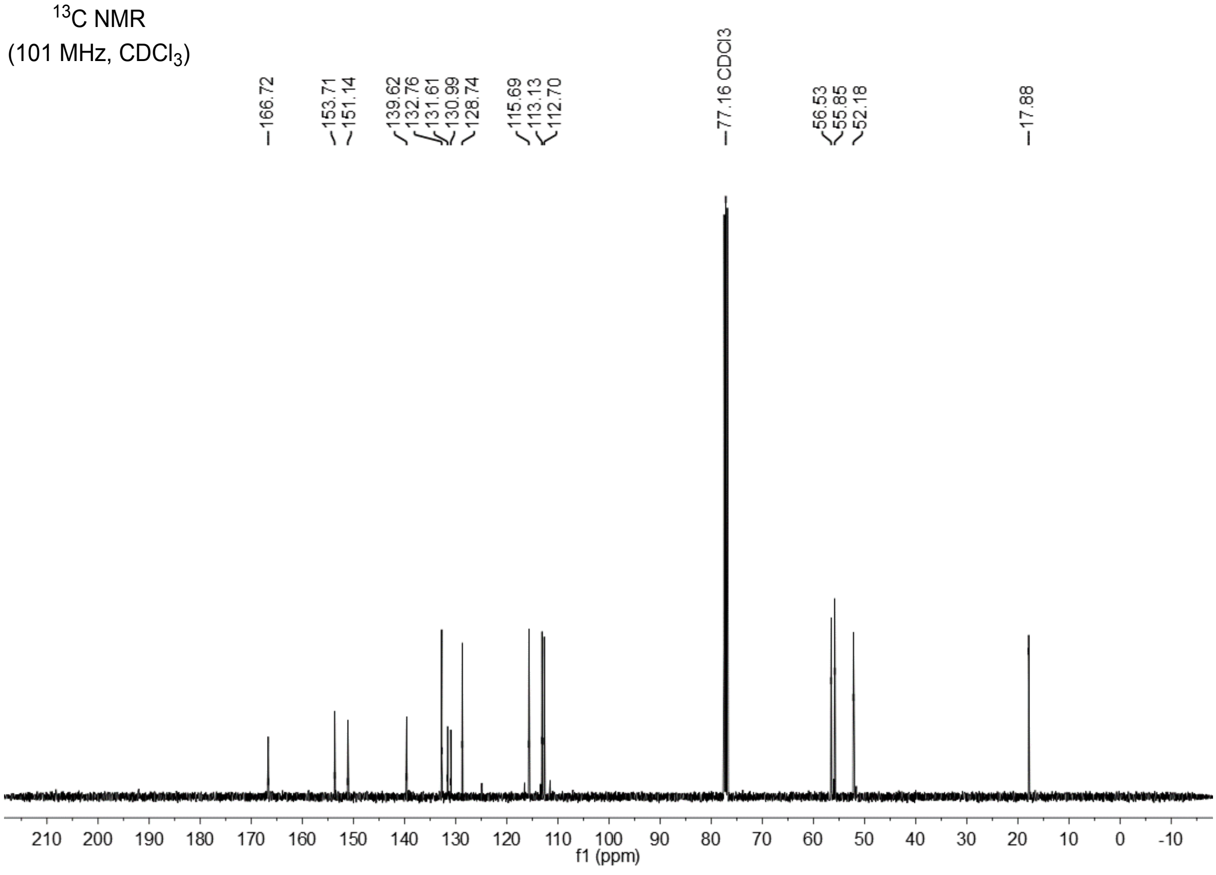

S-12
^{1}H NMR
(400 MHz, $CDCl_3$)

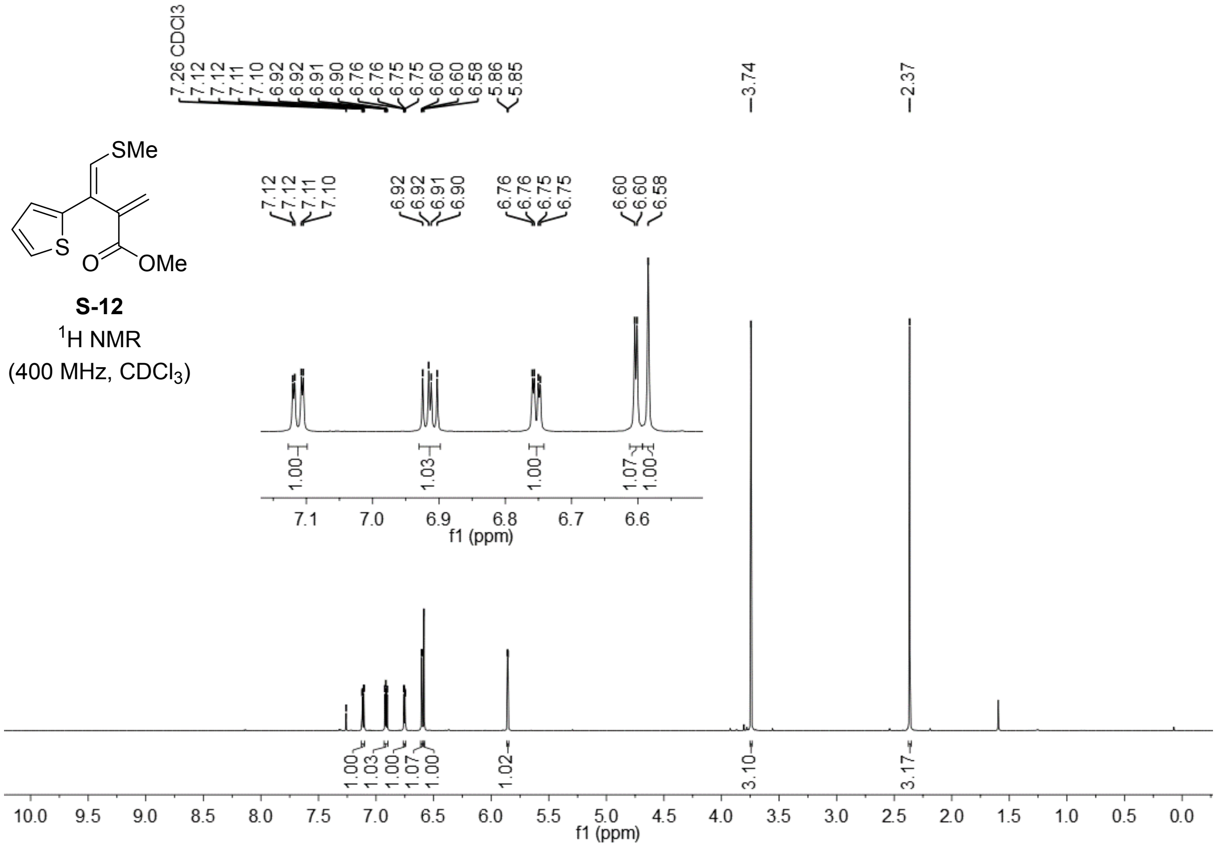

S-12
^{13}C NMR
(101 MHz, $CDCl_3$)

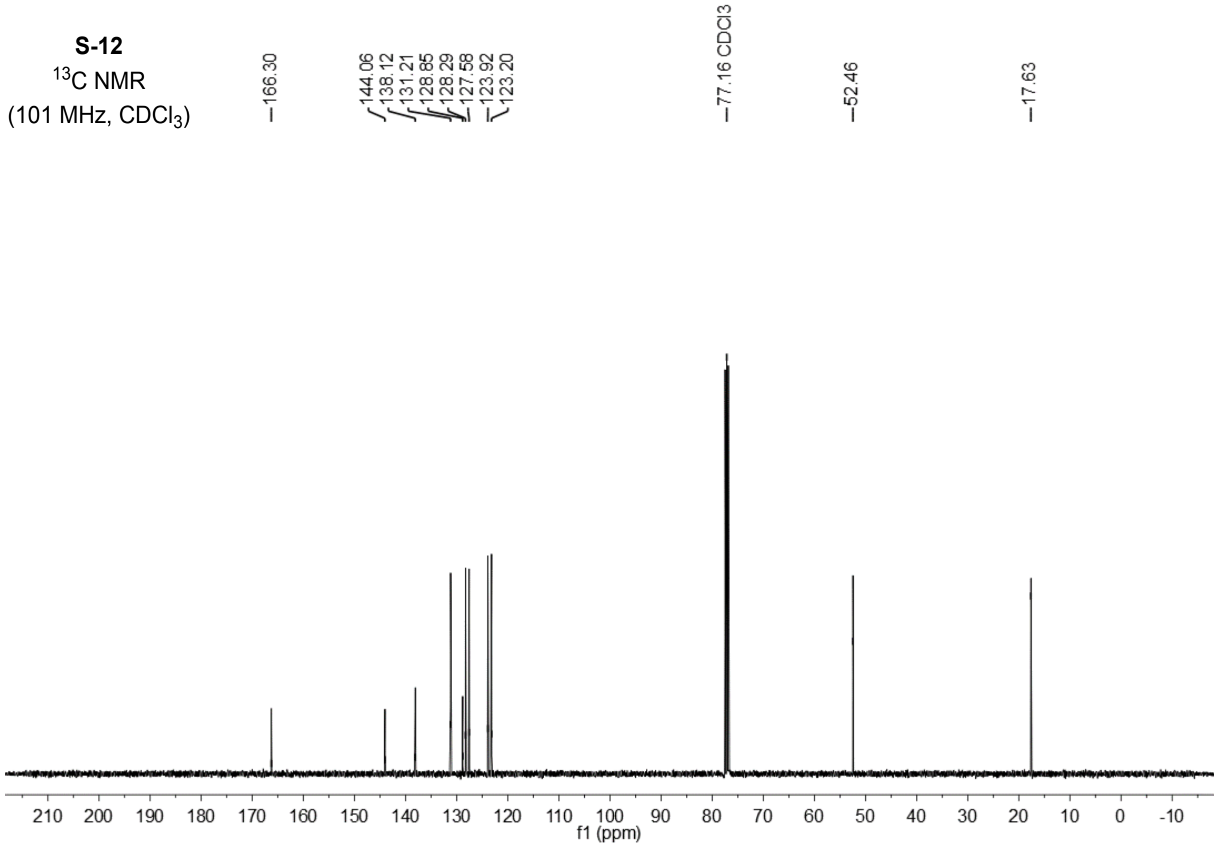

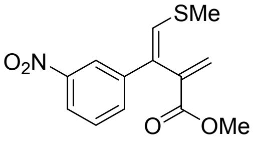

S-13
^{1}H NMR
(400 MHz, $CDCl_3$)

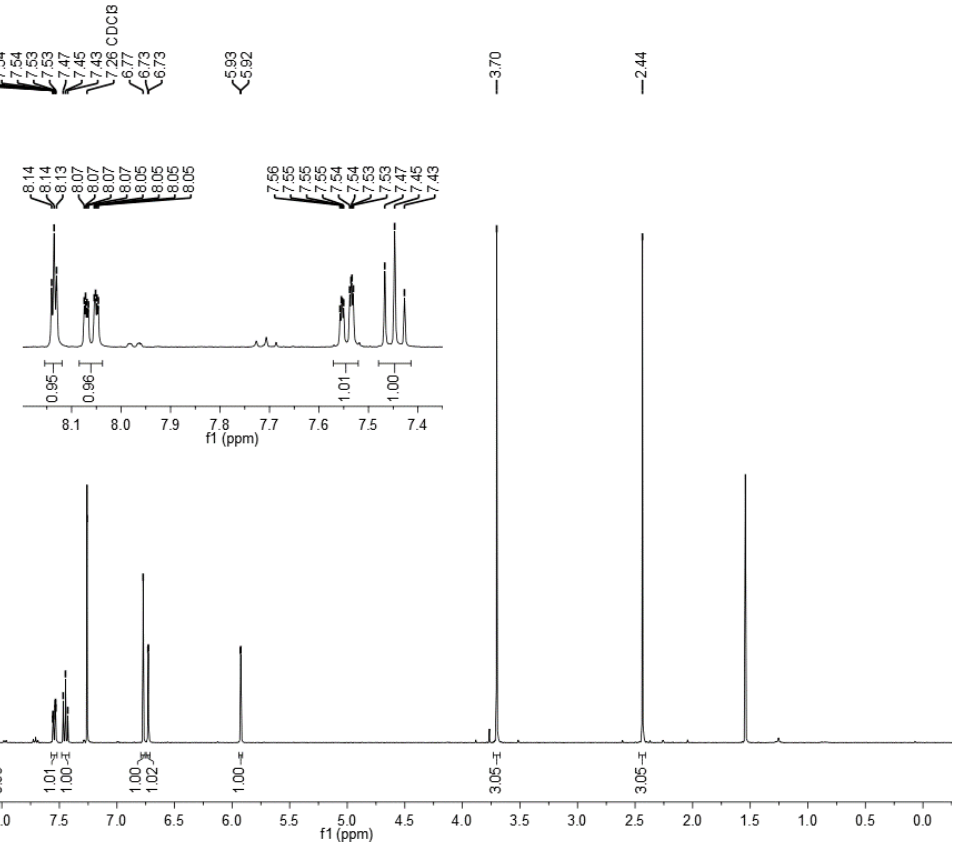

S-13
^{13}C NMR
(101 MHz, $CDCl_3$)

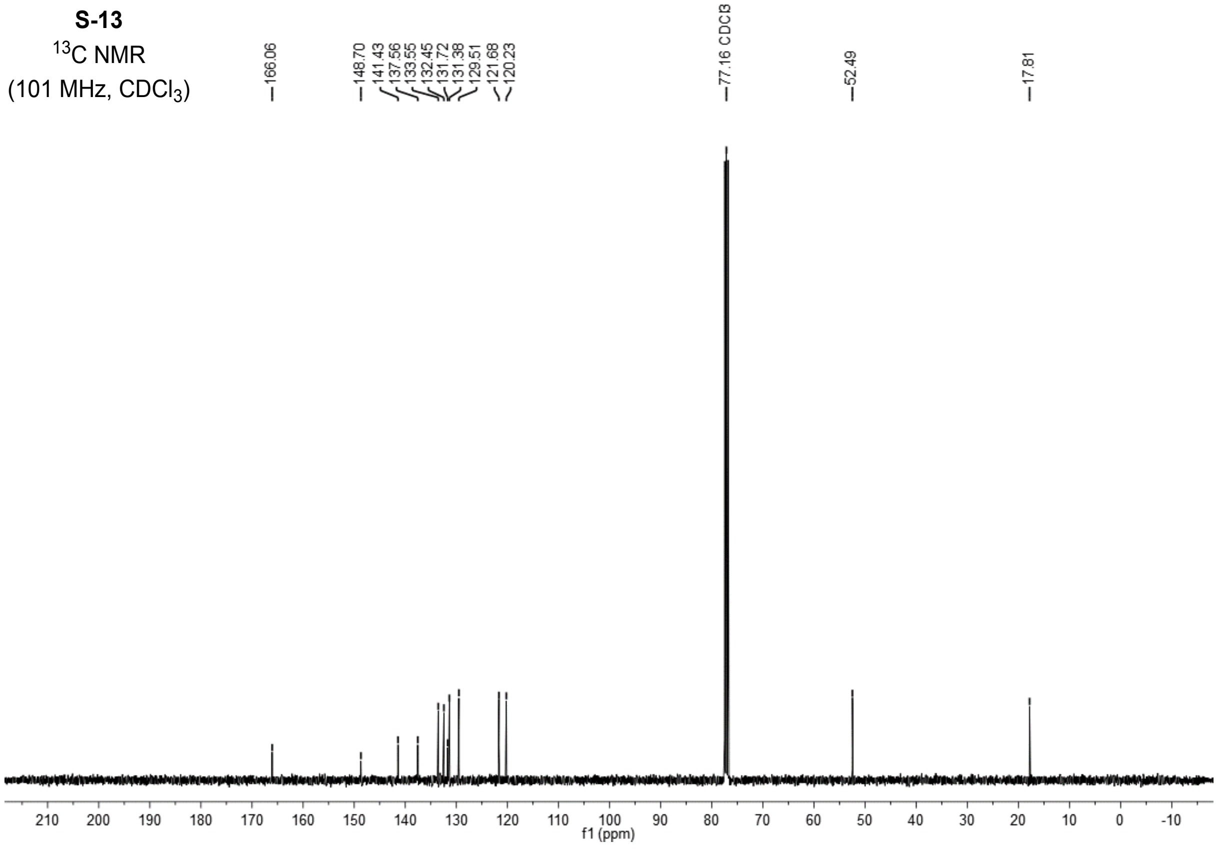

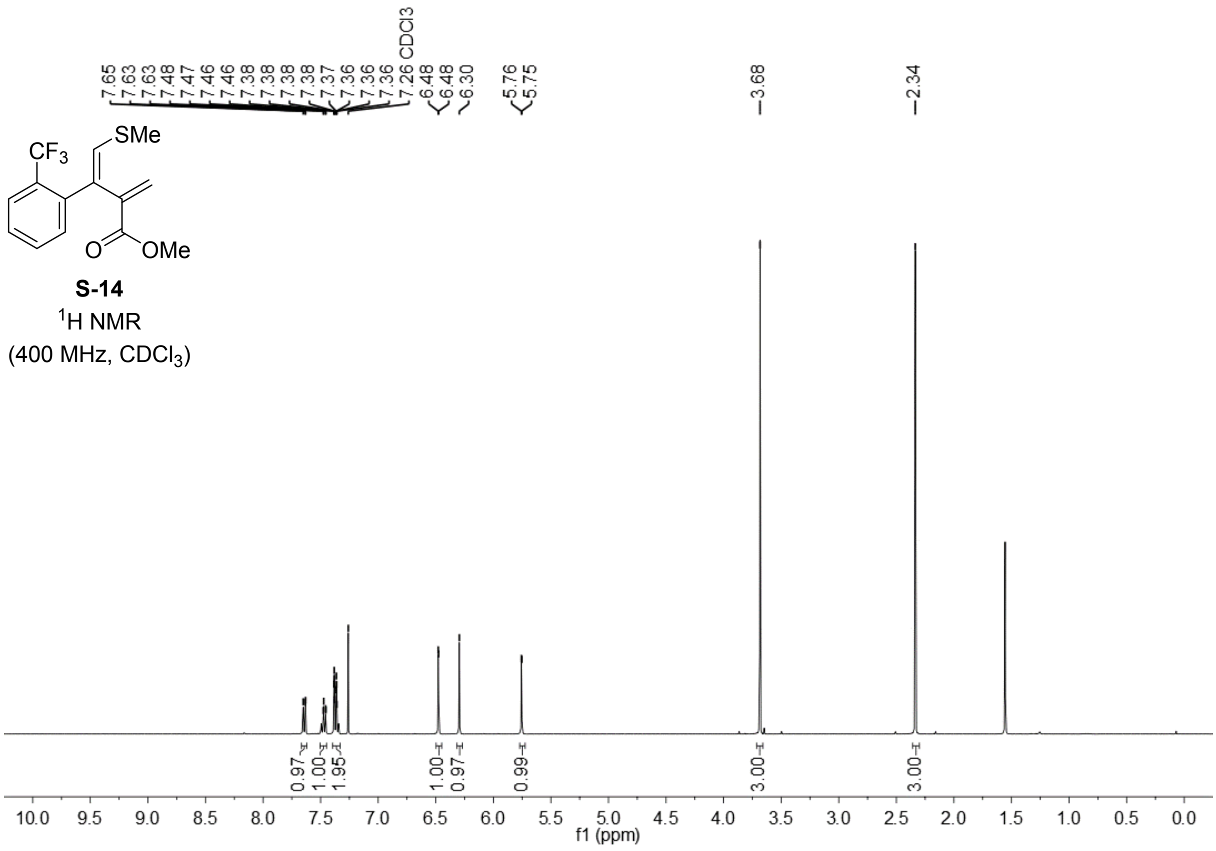

S-14
^{1}H NMR
(400 MHz, $CDCl_3$)

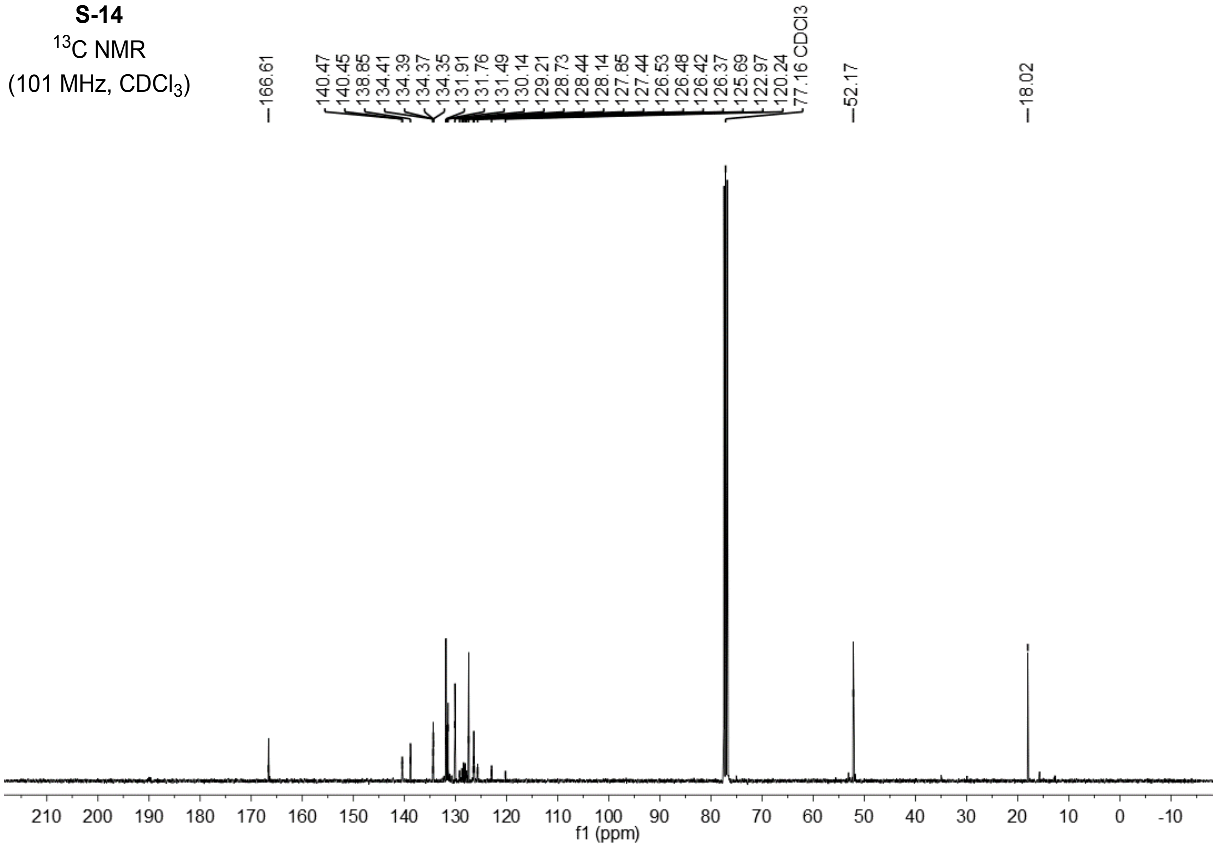

S-14
^{13}C NMR
(101 MHz, $CDCl_3$)

S-14
^{19}F NMR
(376 MHz, $CDCl_3$)

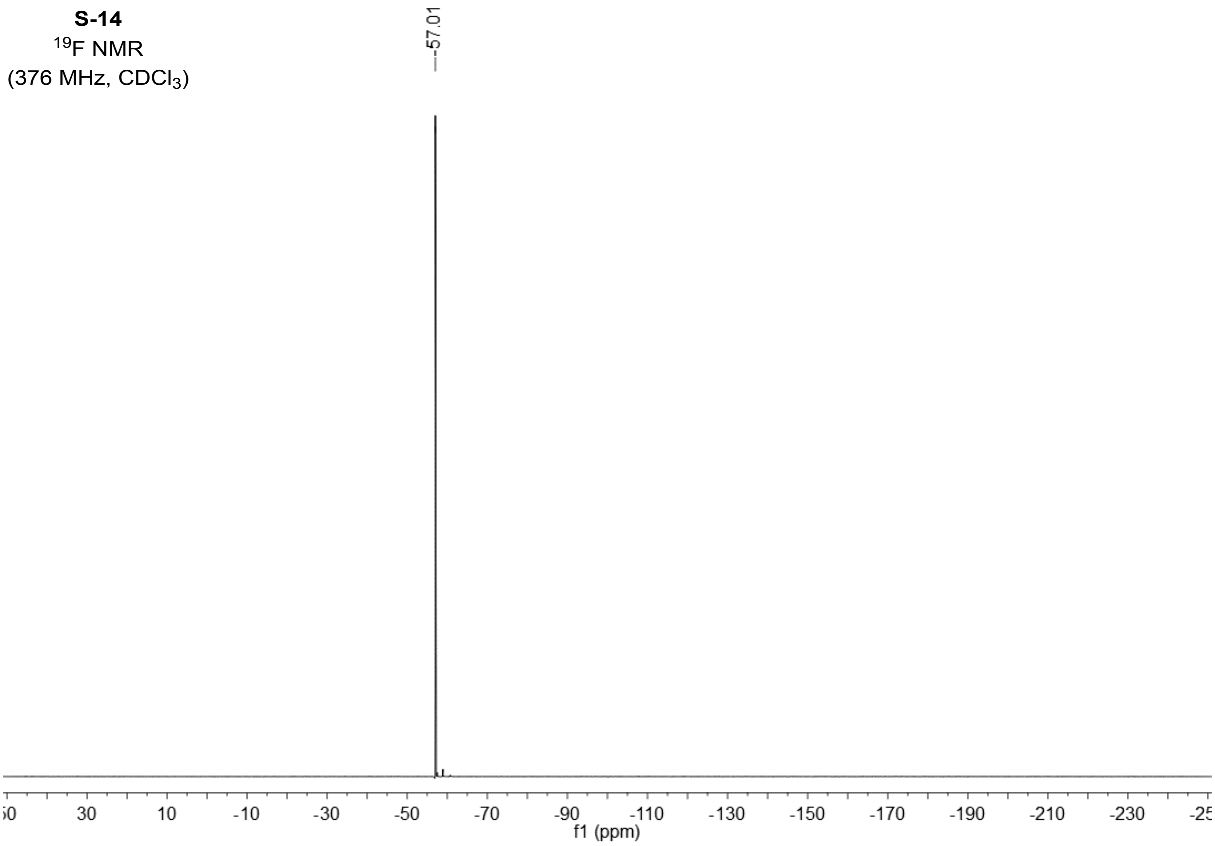

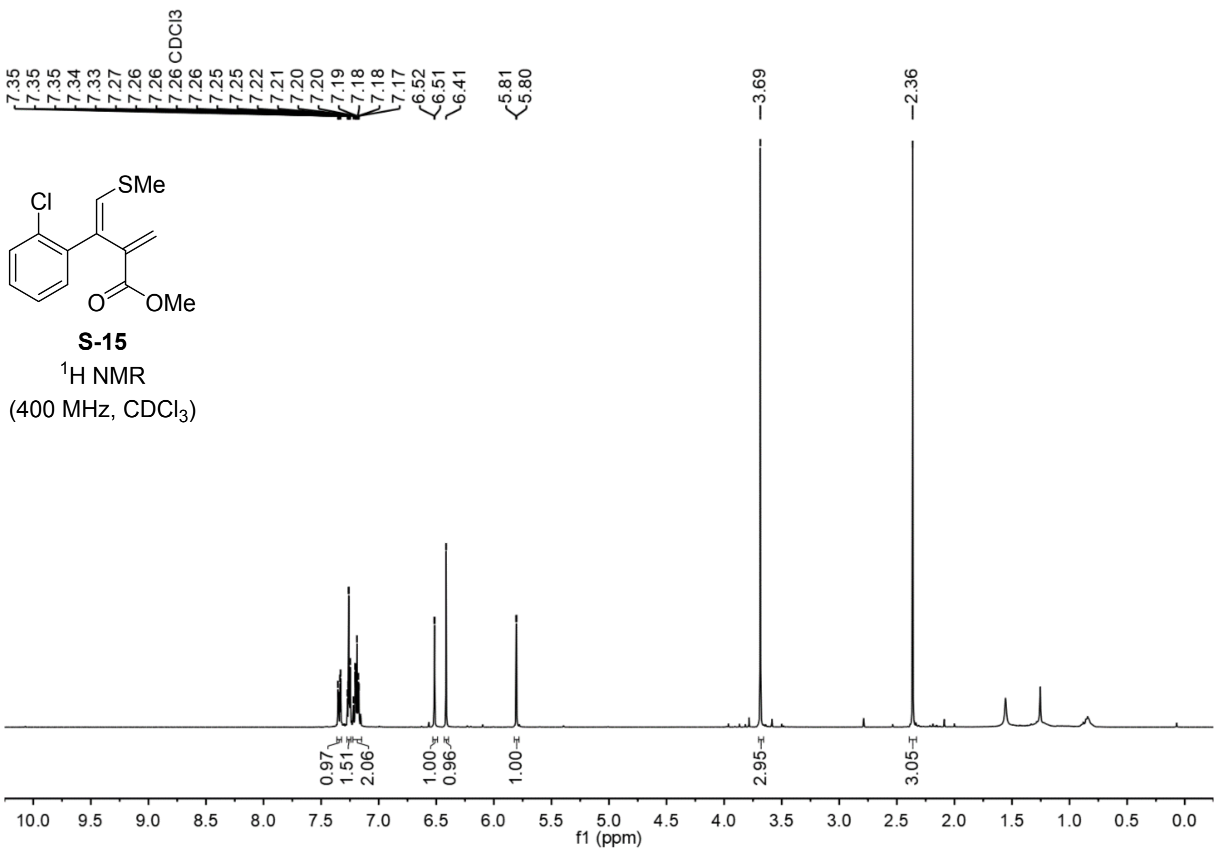
7.35 7.35 7.35 7.34 7.33 7.27 7.26 7.26 CDCl3 7.26 7.25 7.25 7.22 7.21 7.20 7.20 7.19 7.18 7.18 7.17 6.52 6.51 6.41 5.81 5.80
3.69
2.36
SMe
Cl
O
OMe
S-15
1H NMR
(400 MHz, CDCl3)
0.97 1.51 2.06 1.00 0.96 1.00 2.95 3.05
10.0 9.5 9.0 8.5 8.0 7.5 7.0 6.5 6.0 5.5 5.0 4.5 4.0 3.5 3.0 2.5 2.0 1.5 1.0 0.5 0.0
f1 (ppm)

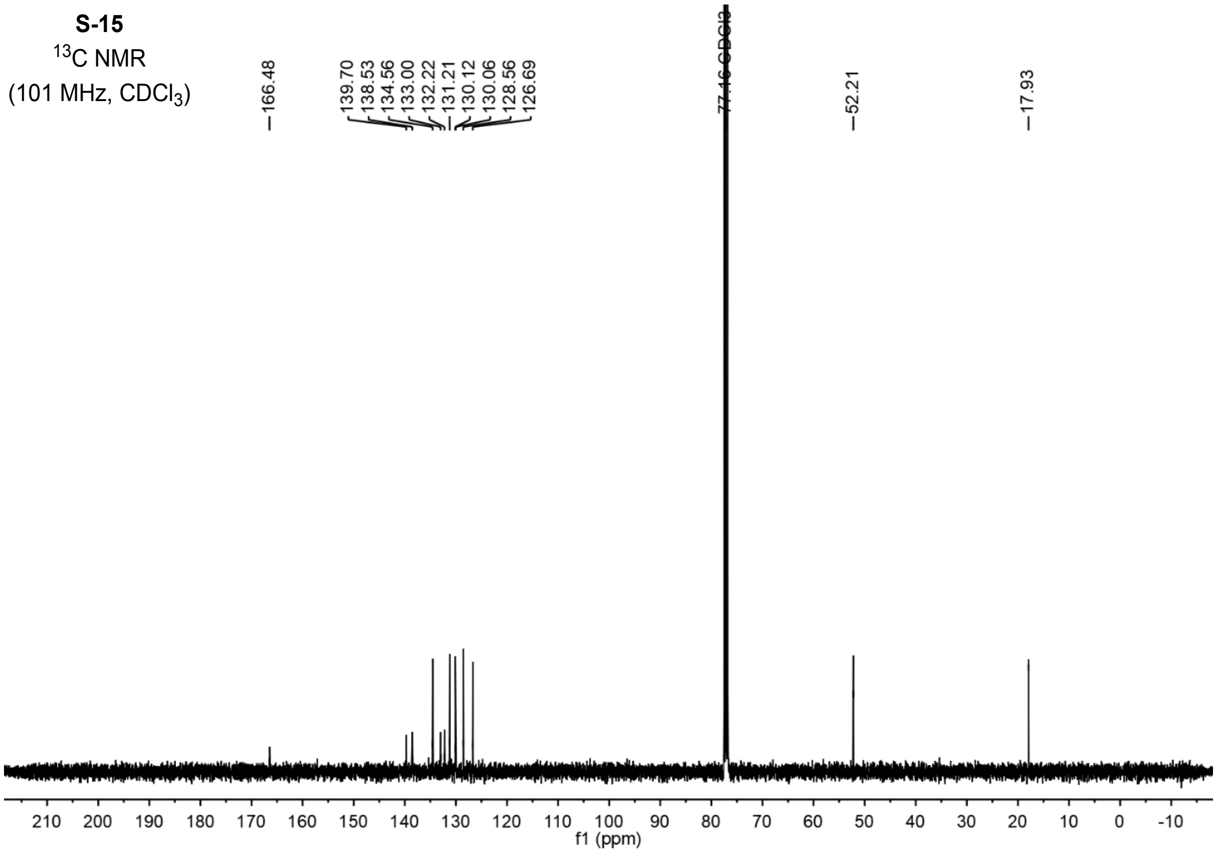
S-15
13C NMR
(101 MHz, CDCl3)
166.48
139.70 138.53 134.56 133.00 132.22 131.21 130.12 130.06 128.56 126.69
77.16 CDCl3
52.21
17.93
210 200 190 180 170 160 150 140 130 120 110 100 90 80 70 60 50 40 30 20 10 0 -10
f1 (ppm)

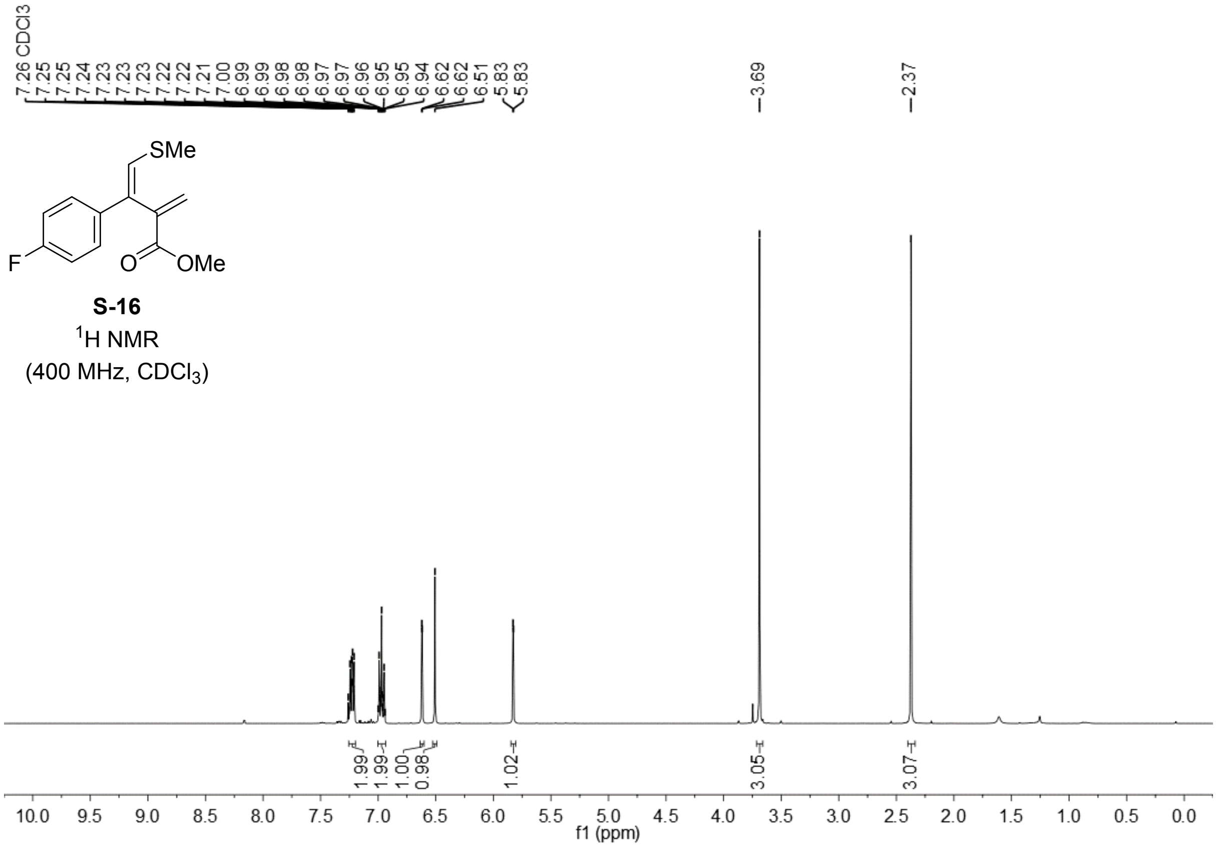
7.26 CDCl3
7.25
7.25
7.24
7.23
7.23
7.23
7.22
7.22
7.21
7.00
6.99
6.99
6.98
6.98
6.97
6.97
6.96
6.95
6.95
6.94
6.62
6.62
6.51
5.83
5.83
3.69
2.37
SMe
F
O
OMe
S-16
1H NMR
(400 MHz, CDCl3)
1.99
1.99
1.00
0.98
1.02
3.05
3.07
10.0 9.5 9.0 8.5 8.0 7.5 7.0 6.5 6.0 5.5 5.0 4.5 4.0 3.5 3.0 2.5 2.0 1.5 1.0 0.5 0.0
f1 (ppm)

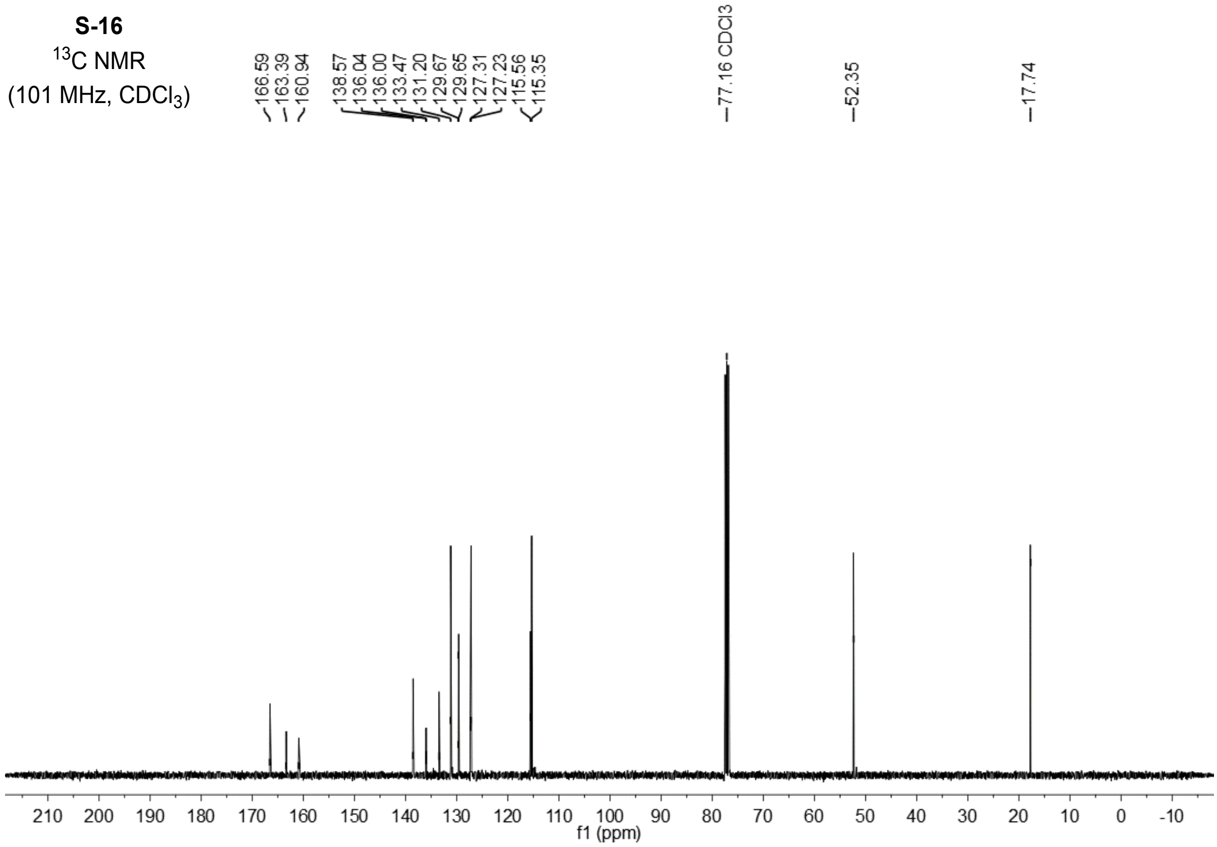
S-16
13C NMR
(101 MHz, CDCl3)
166.59
163.39
160.94
138.57
136.04
136.00
133.47
131.20
129.67
129.65
127.31
127.23
115.56
115.35
77.16 CDCl3
52.35
17.74
210 200 190 180 170 160 150 140 130 120 110 100 90 80 70 60 50 40 30 20 10 0 -10
f1 (ppm)

S-16
^{19}F NMR
(376 MHz, $CDCl_3$)

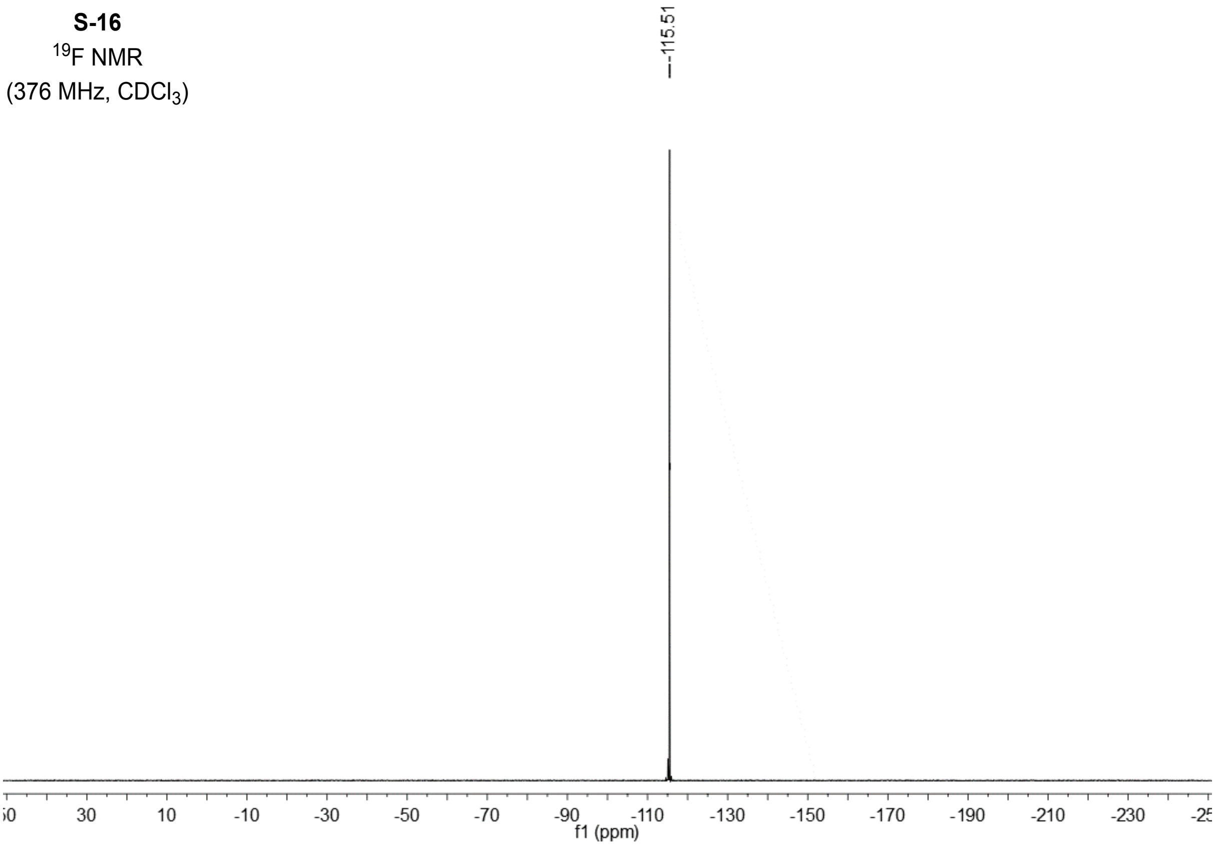

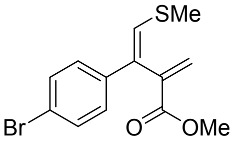

S-17
^{1}H NMR
(400 MHz, $CDCl_3$)

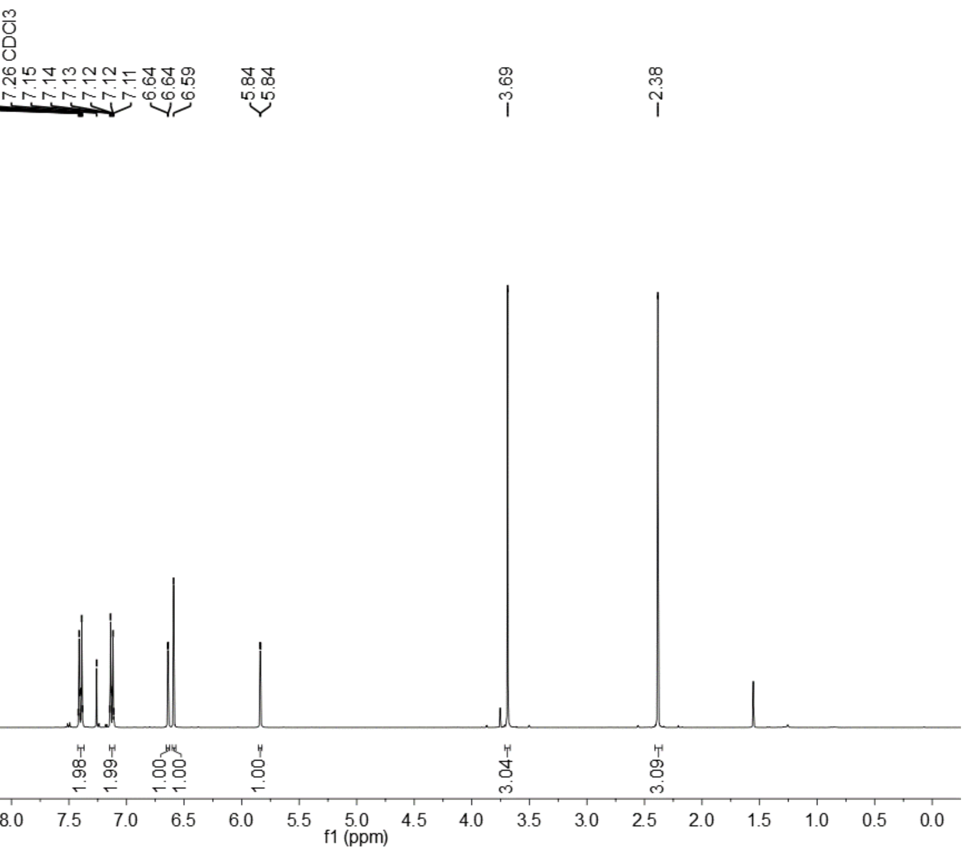

S-17
^{13}C NMR
(101 MHz, $CDCl_3$)

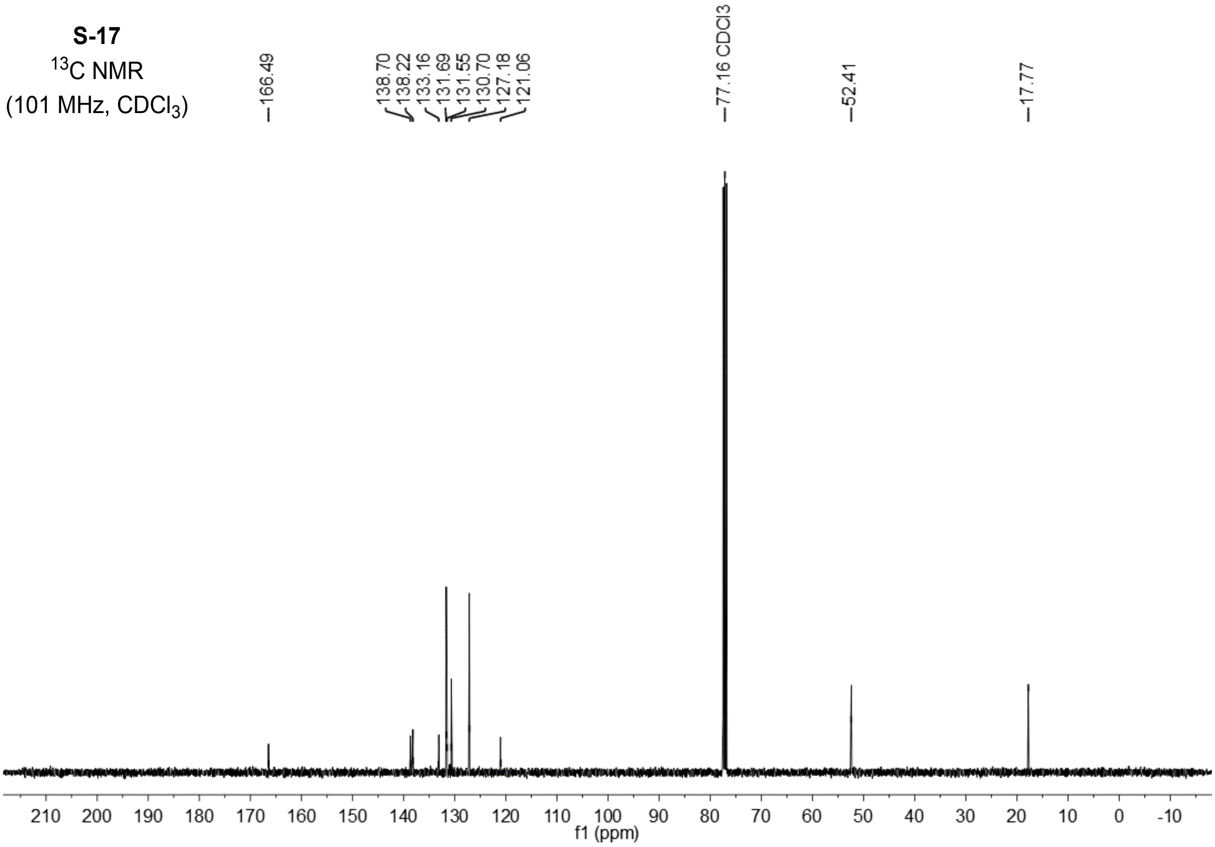

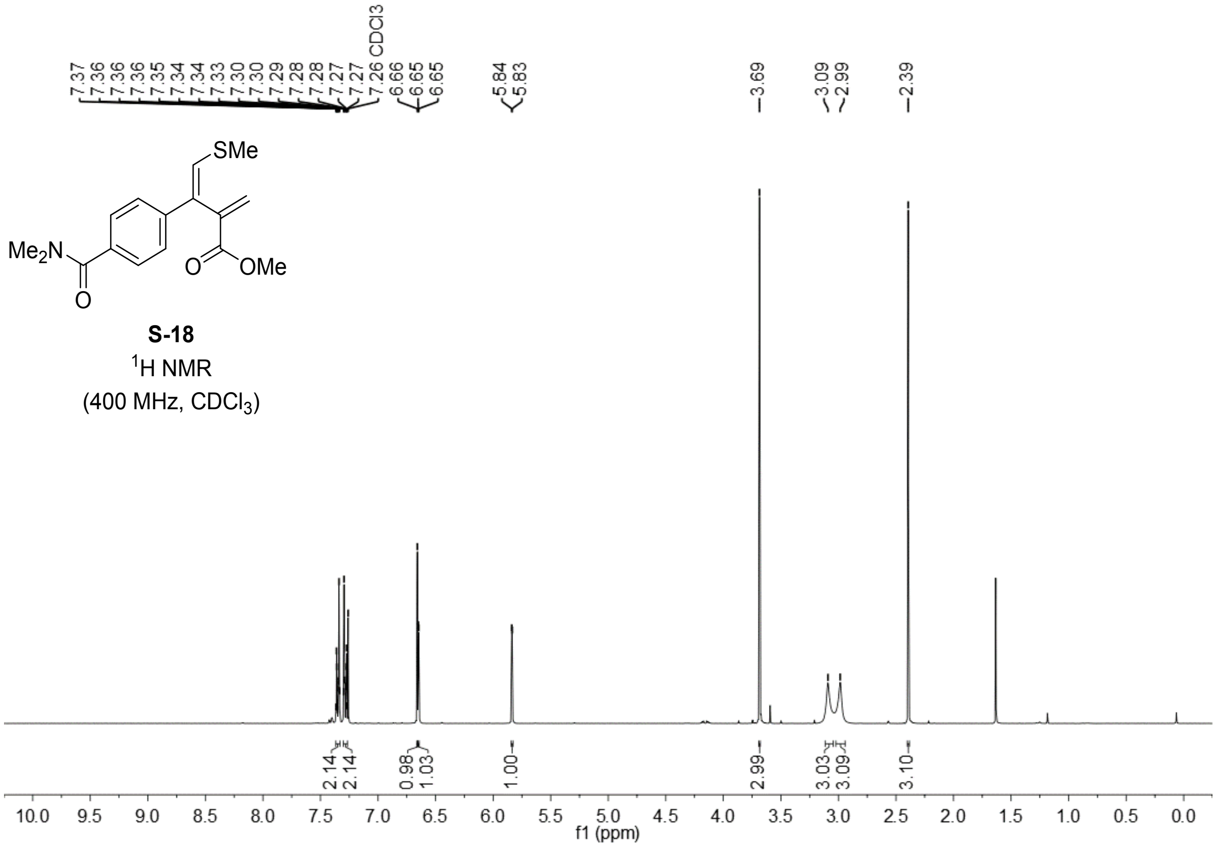
7.37
7.36
7.36
7.35
7.34
7.34
7.33
7.30
7.30
7.29
7.28
7.28
7.27
7.27
7.26 CDCl3
6.66
6.65
6.65
5.84
5.83
3.69
3.09
2.99
2.39
SMe
Me2N
O
O
OMe
S-18
1H NMR
(400 MHz, CDCl3)
2.14
2.14
0.98
1.03
1.00
2.99
3.03
3.09
3.10
f1 (ppm)

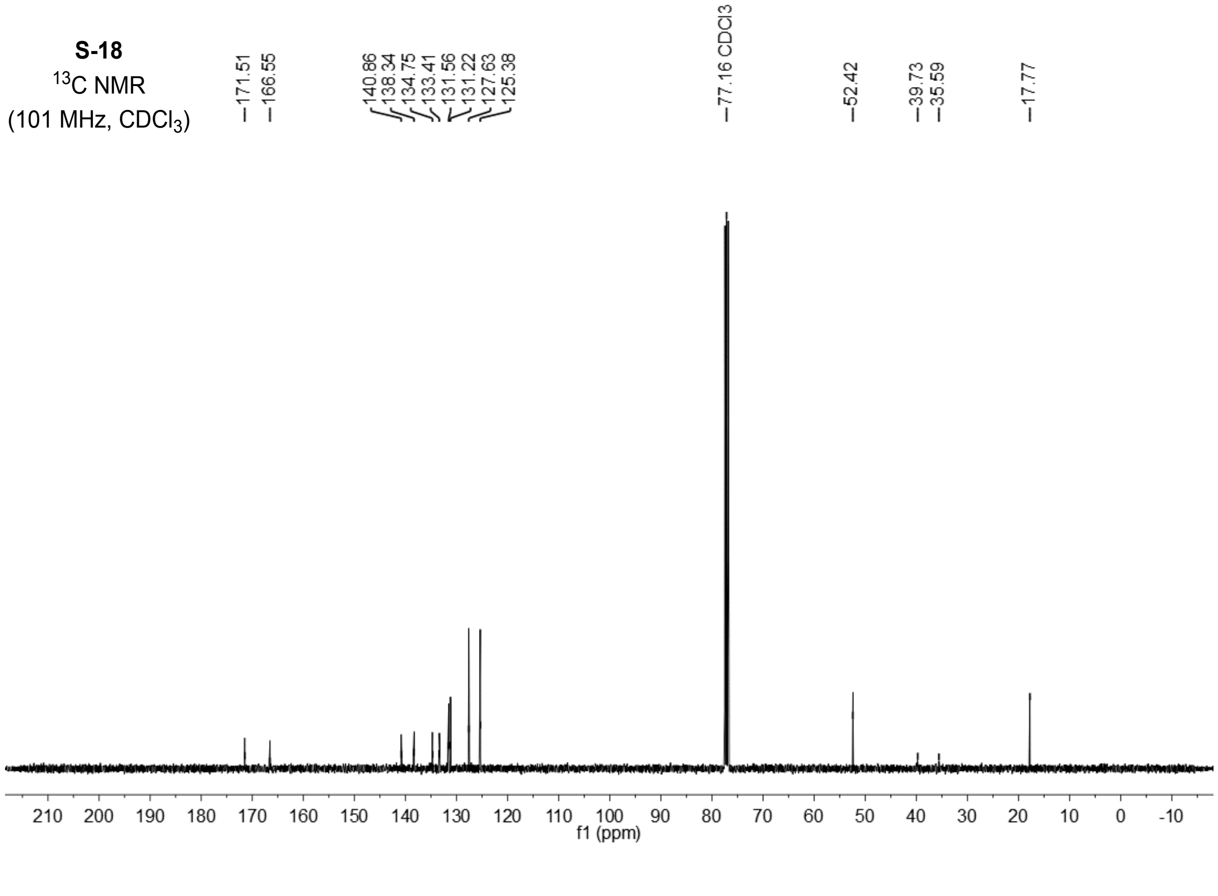
S-18
13C NMR
(101 MHz, CDCl3)
171.51
166.55
140.86
138.34
134.75
133.41
131.56
131.22
127.63
125.38
77.16 CDCl3
52.42
39.73
35.59
17.77
f1 (ppm)

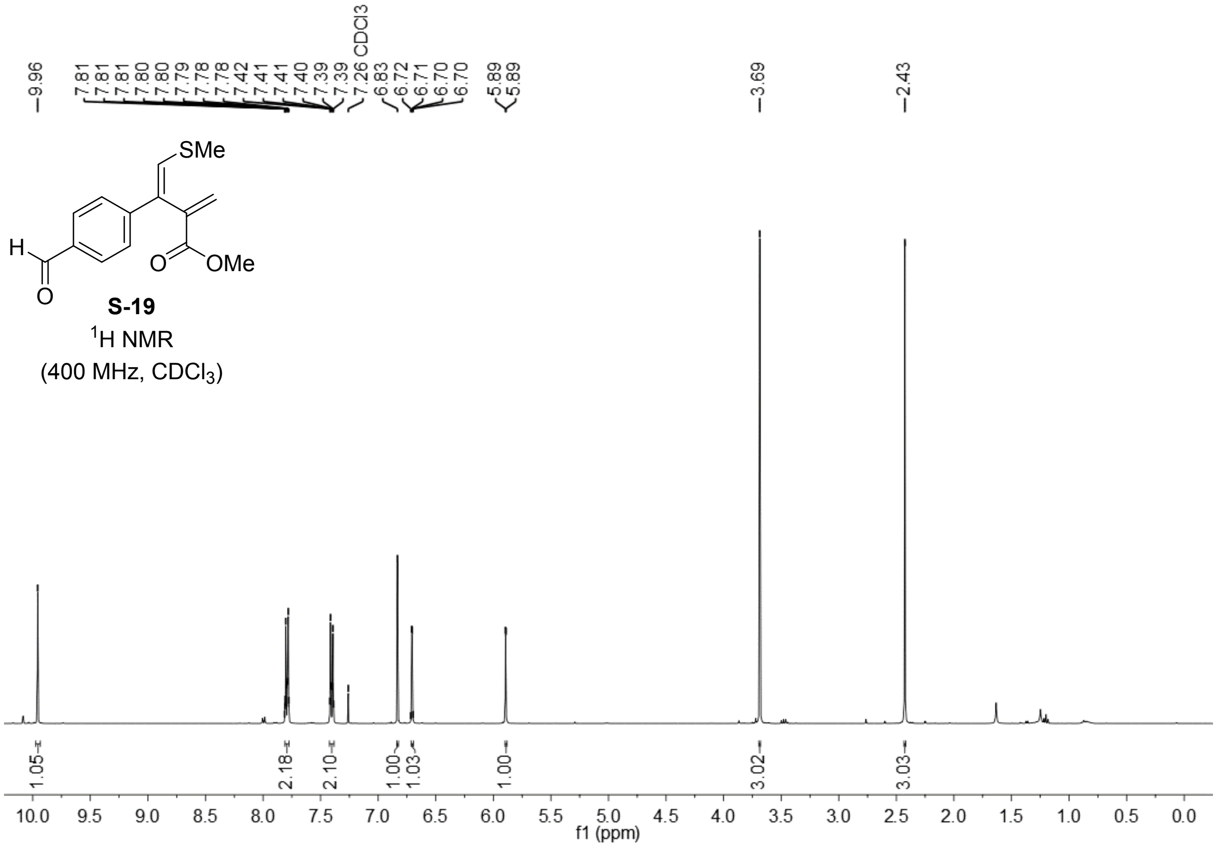

S-19

^{1}H NMR

(400 MHz, $CDCl_3$)

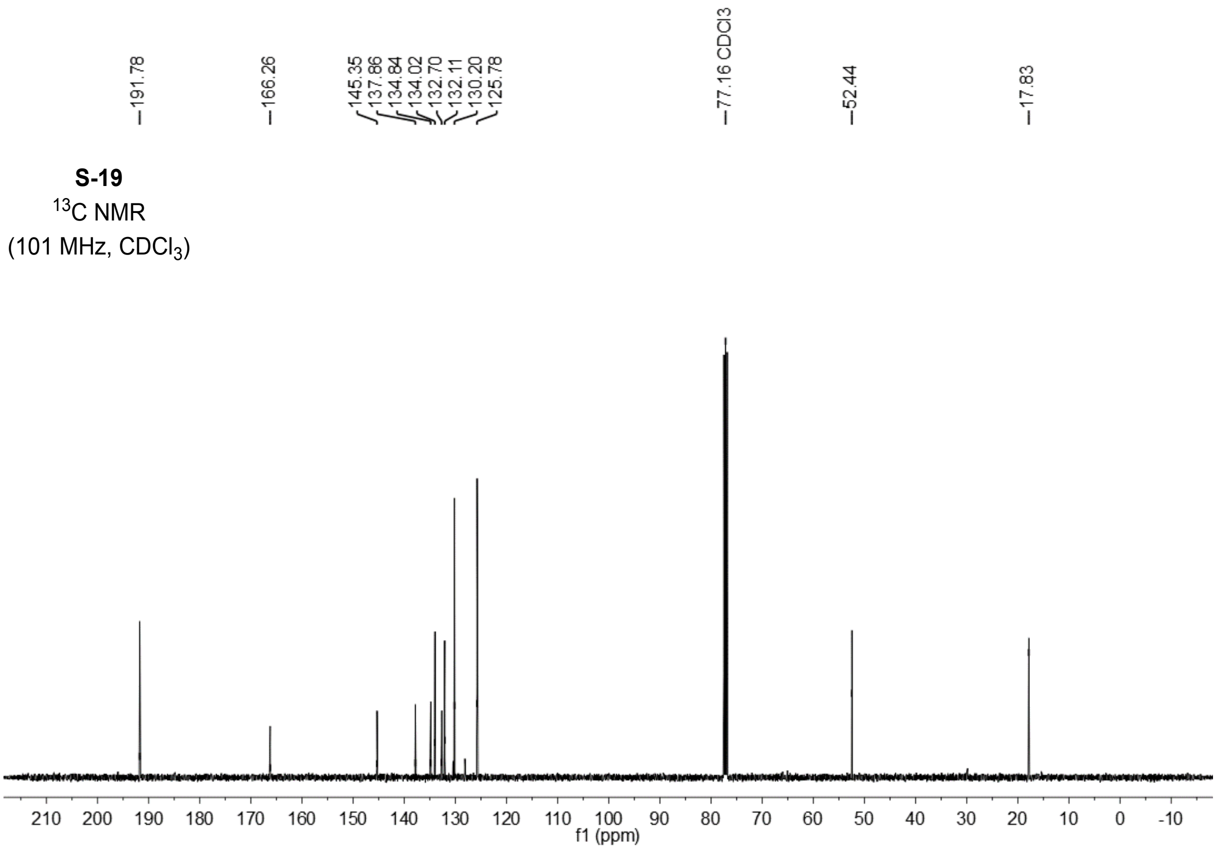

S-19

^{13}C NMR

(101 MHz, $CDCl_3$)

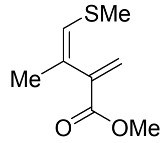

S-20
^{1}H NMR
(400 MHz, $CDCl_3$)

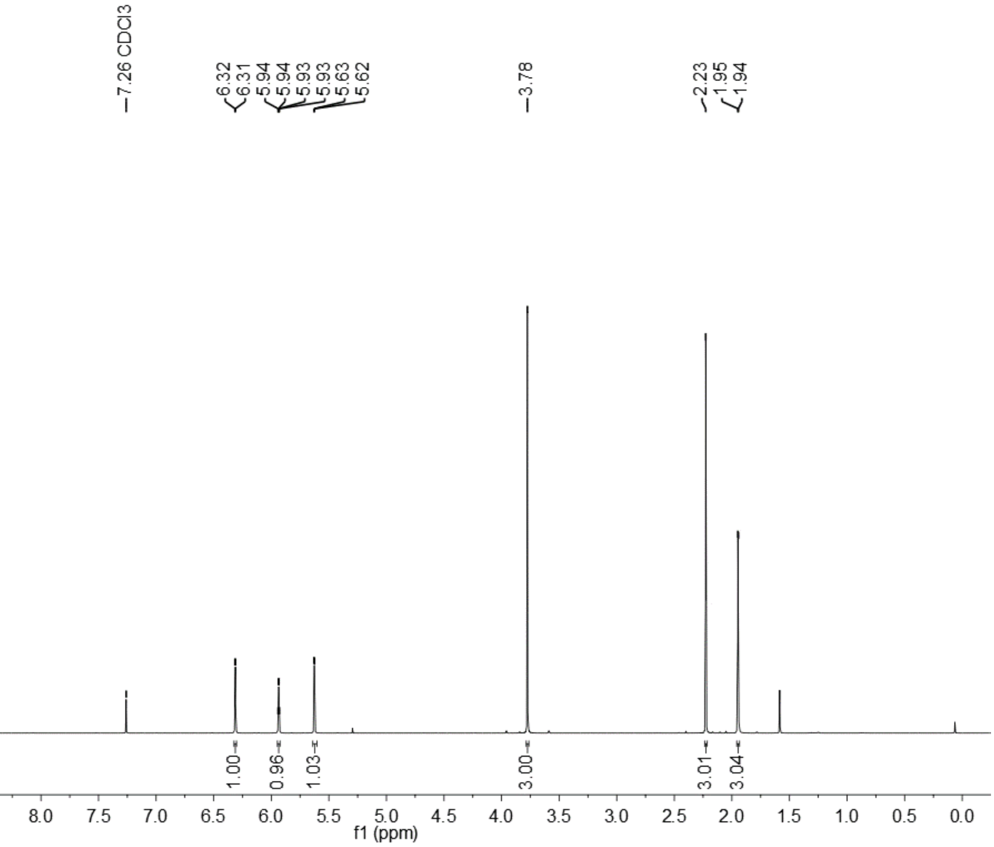

S-20
^{13}C NMR
(101 MHz, $CDCl_3$)

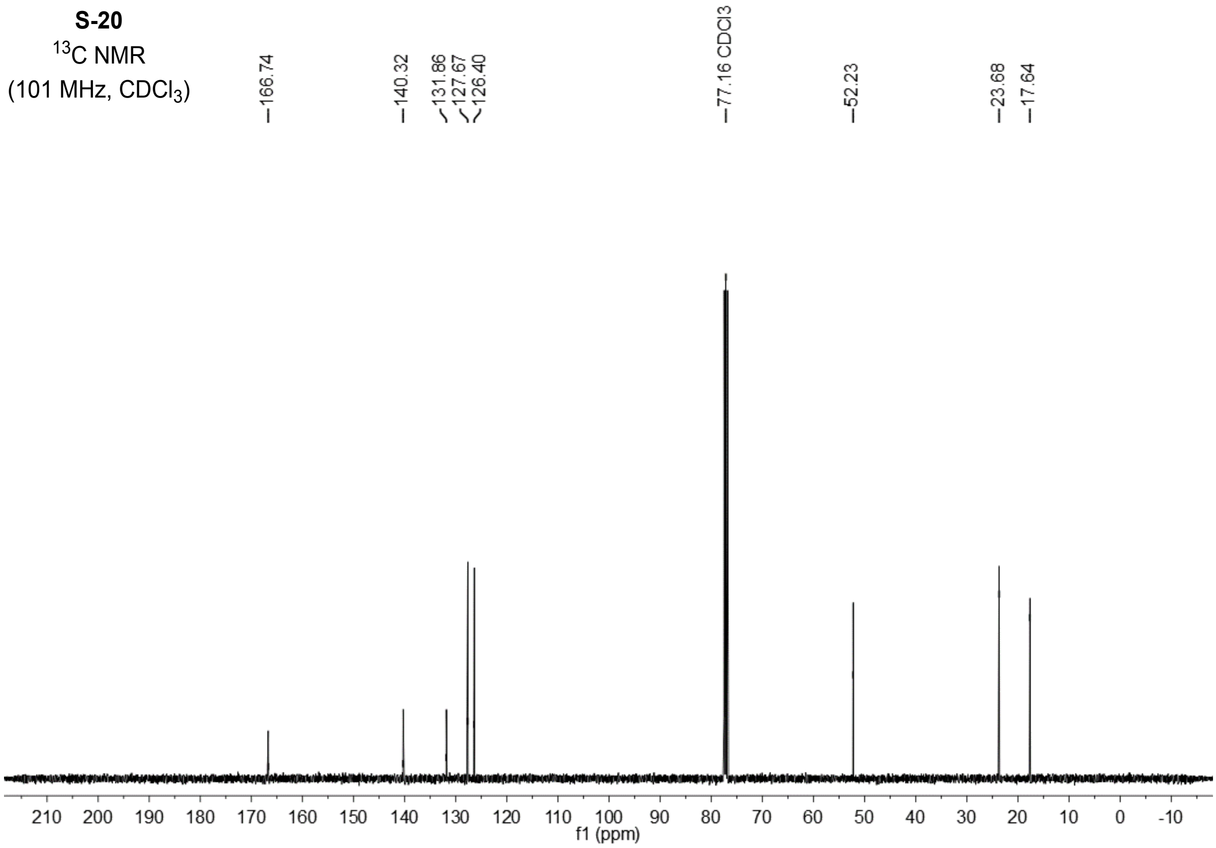

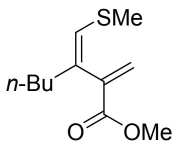

S-21
^{1}H NMR
(400 MHz, $CDCl_3$)

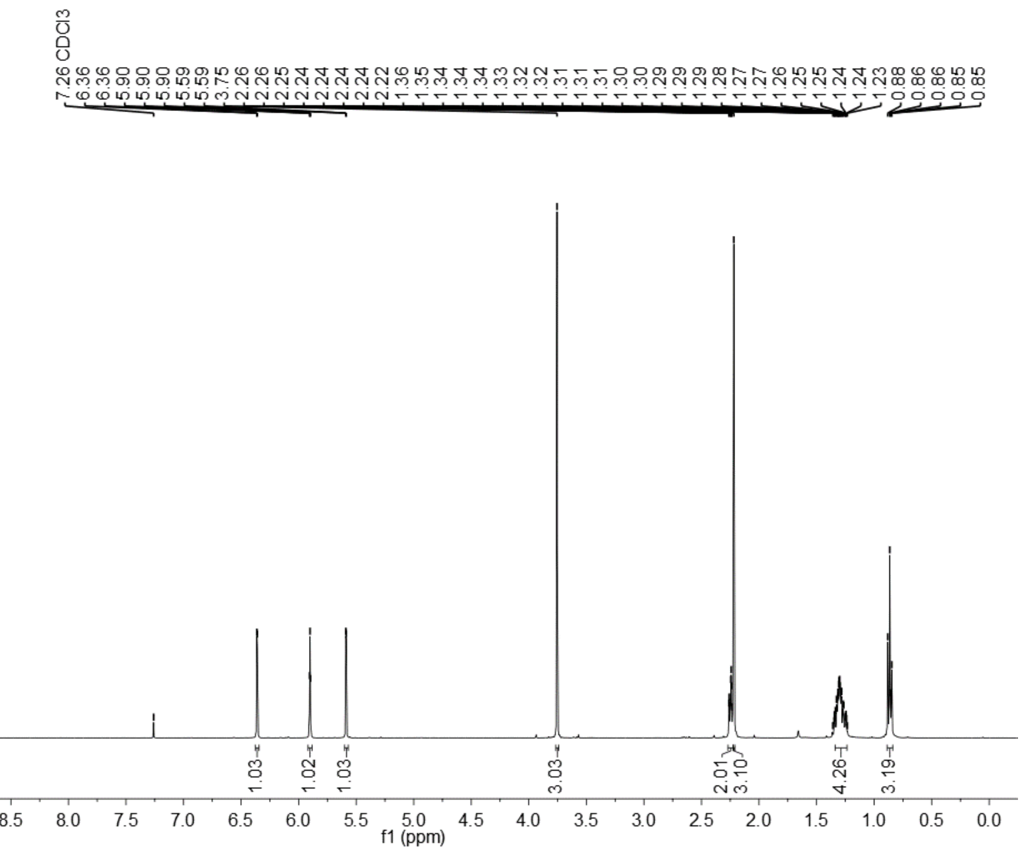

S-21
^{13}C NMR
(101 MHz, $CDCl_3$)

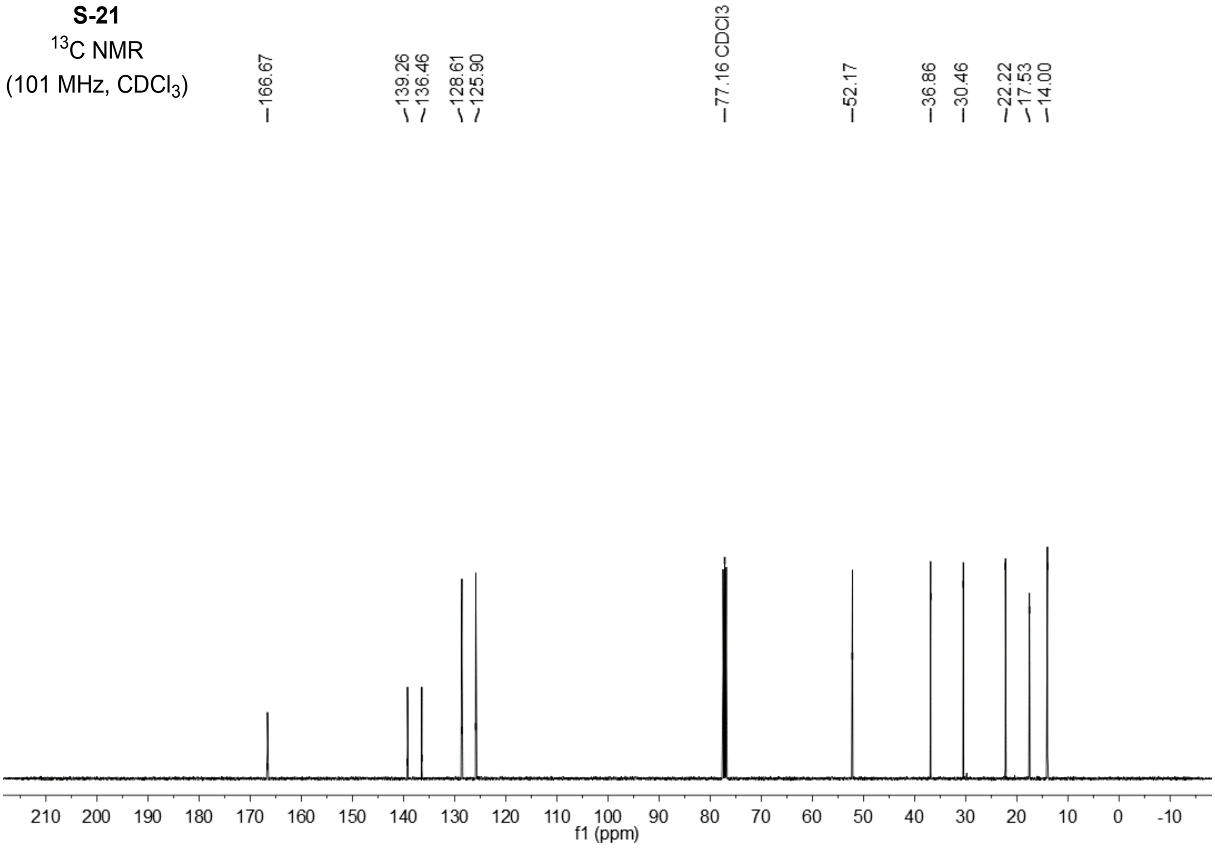

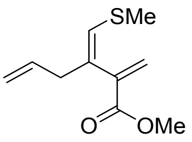

S-22
^{1}H NMR
(400 MHz, $CDCl_3$)

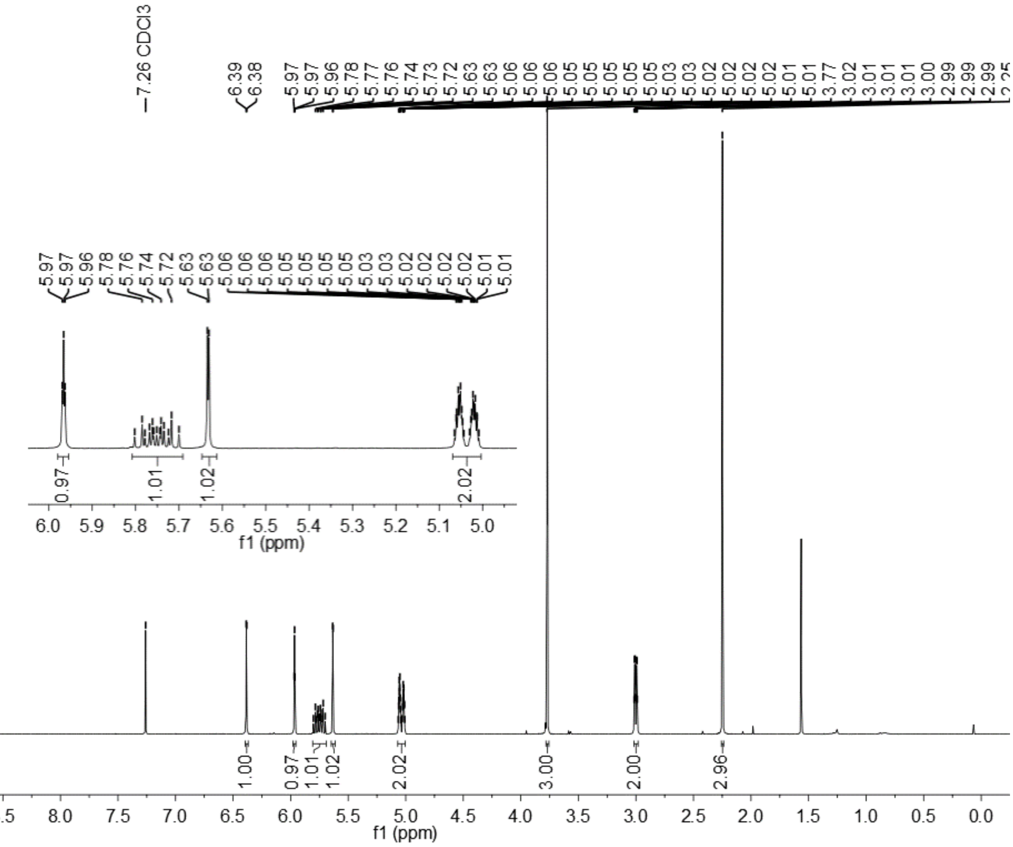

S-22
^{13}C NMR
(101 MHz, $CDCl_3$)

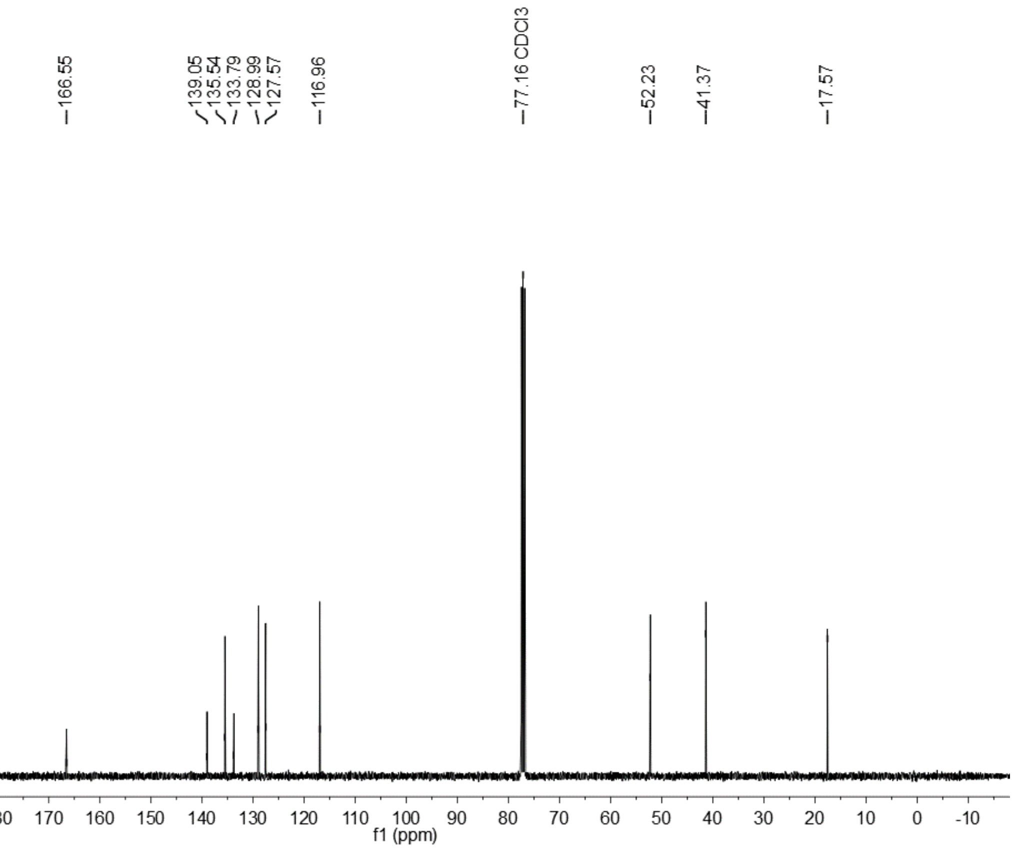

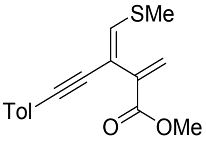

S-23
^{1}H NMR
(400 MHz, $CDCl_3$)

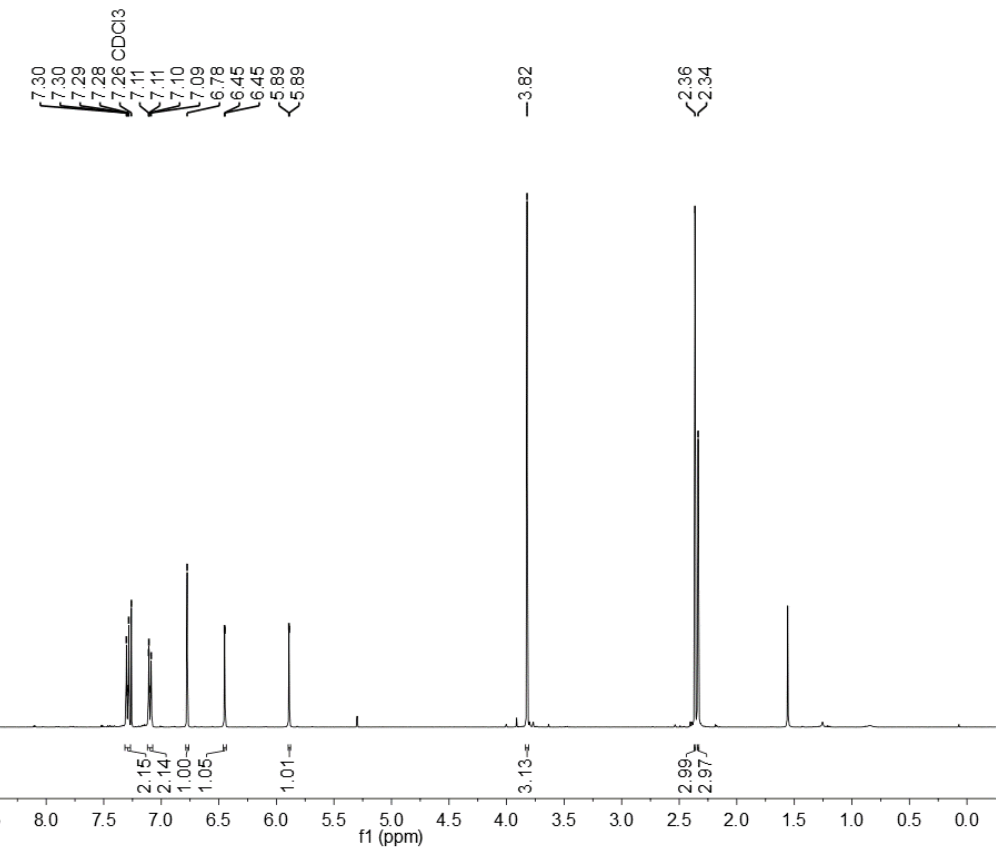

S-23
^{13}C NMR
(101 MHz, $CDCl_3$)

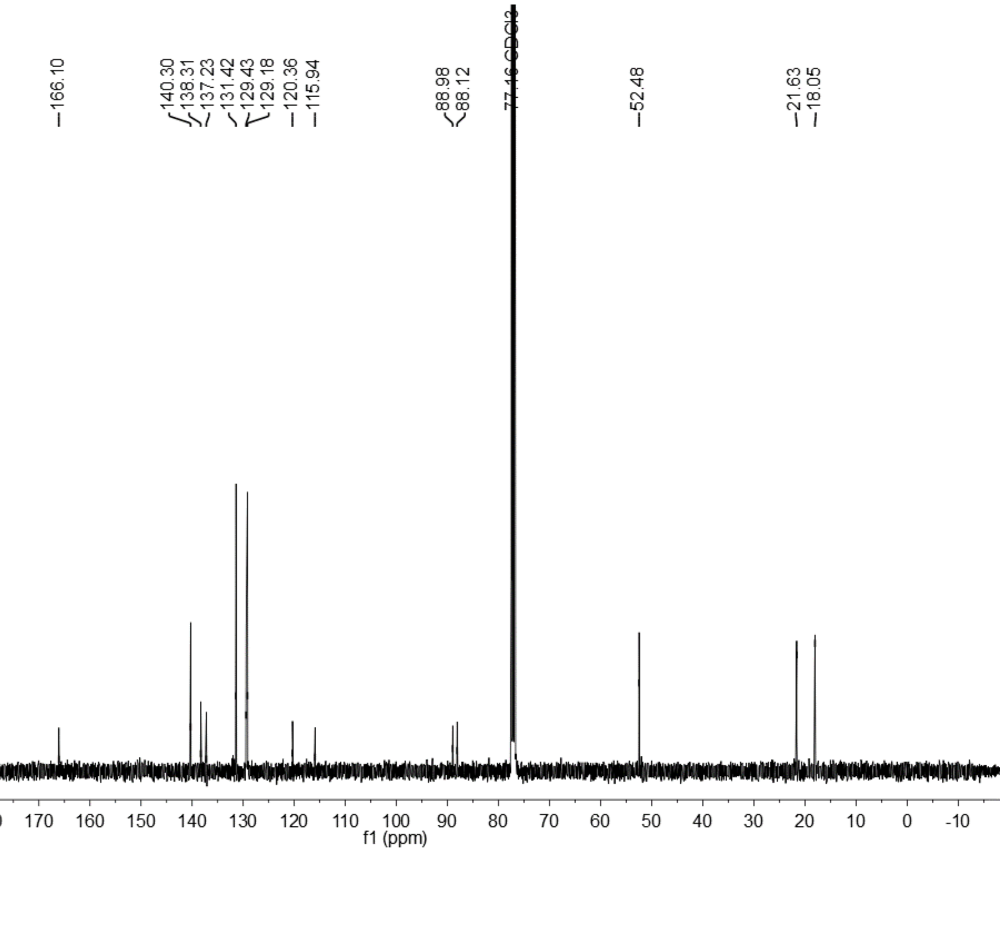

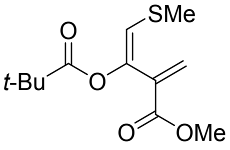

S-24
^{1}H NMR
(400 MHz, $CDCl_3$)

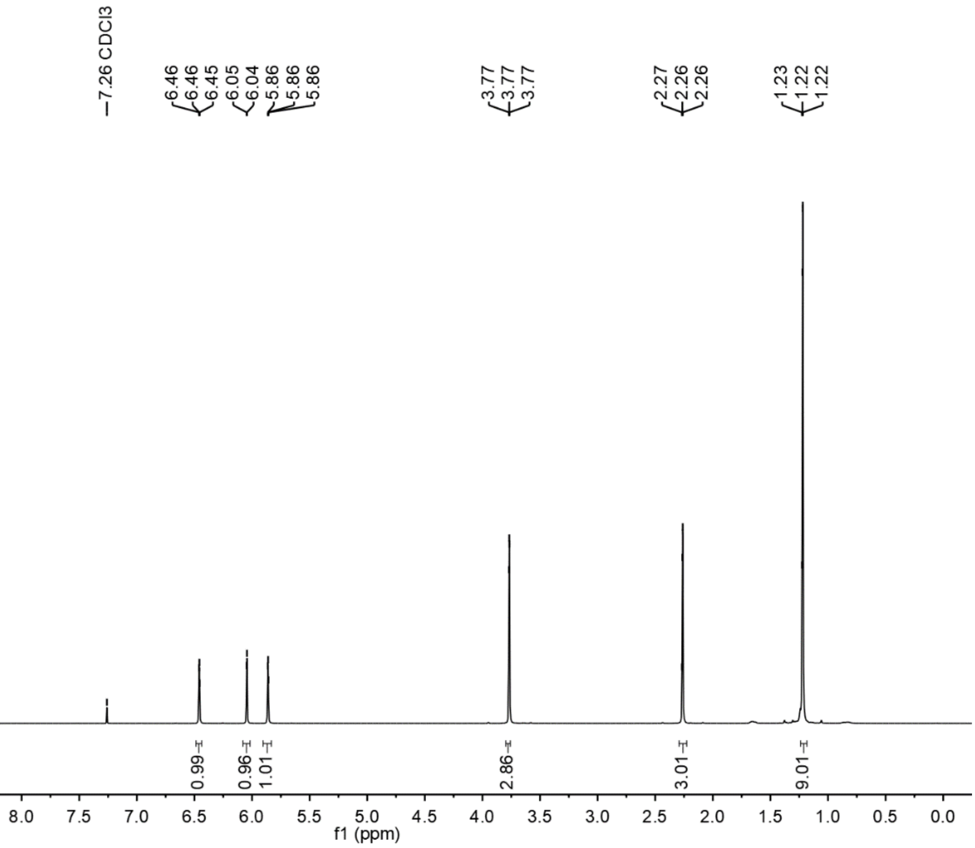

S-24
^{13}C NMR
(101 MHz, $CDCl_3$)

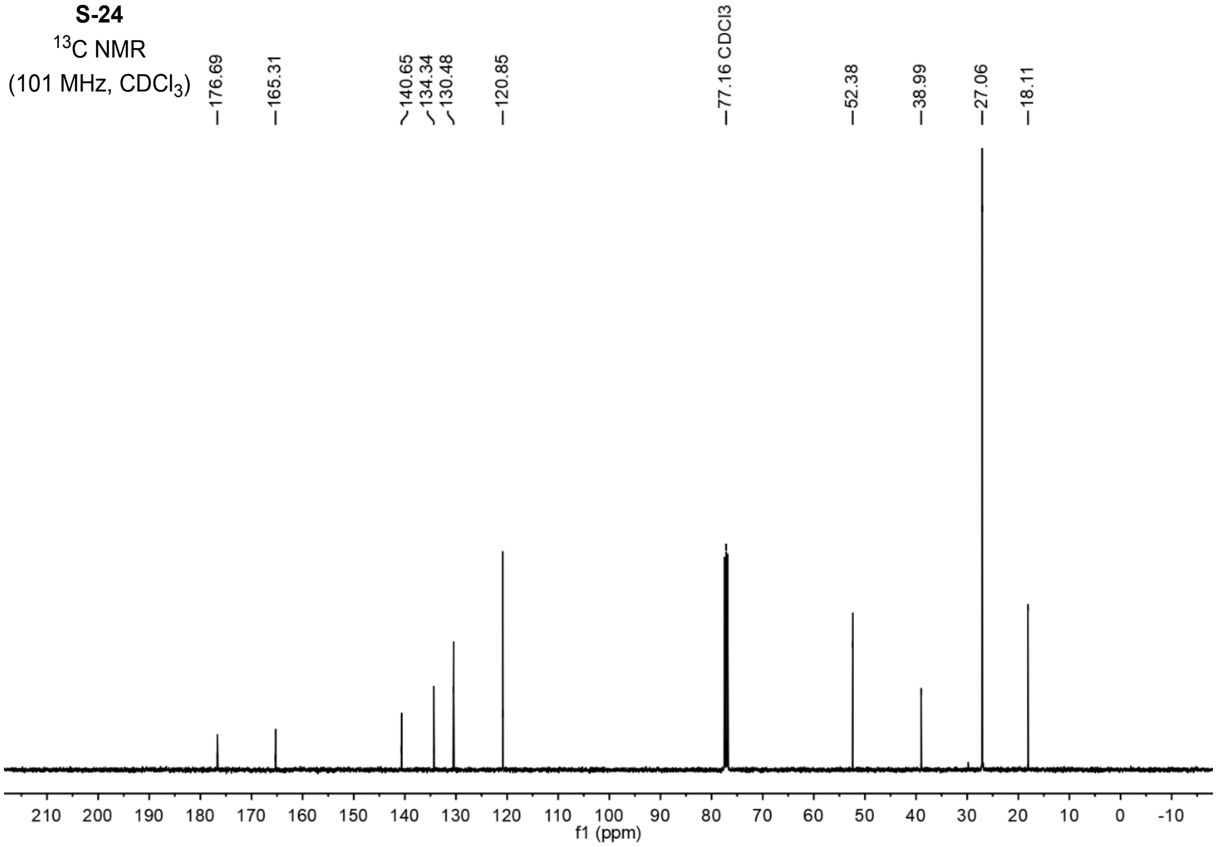

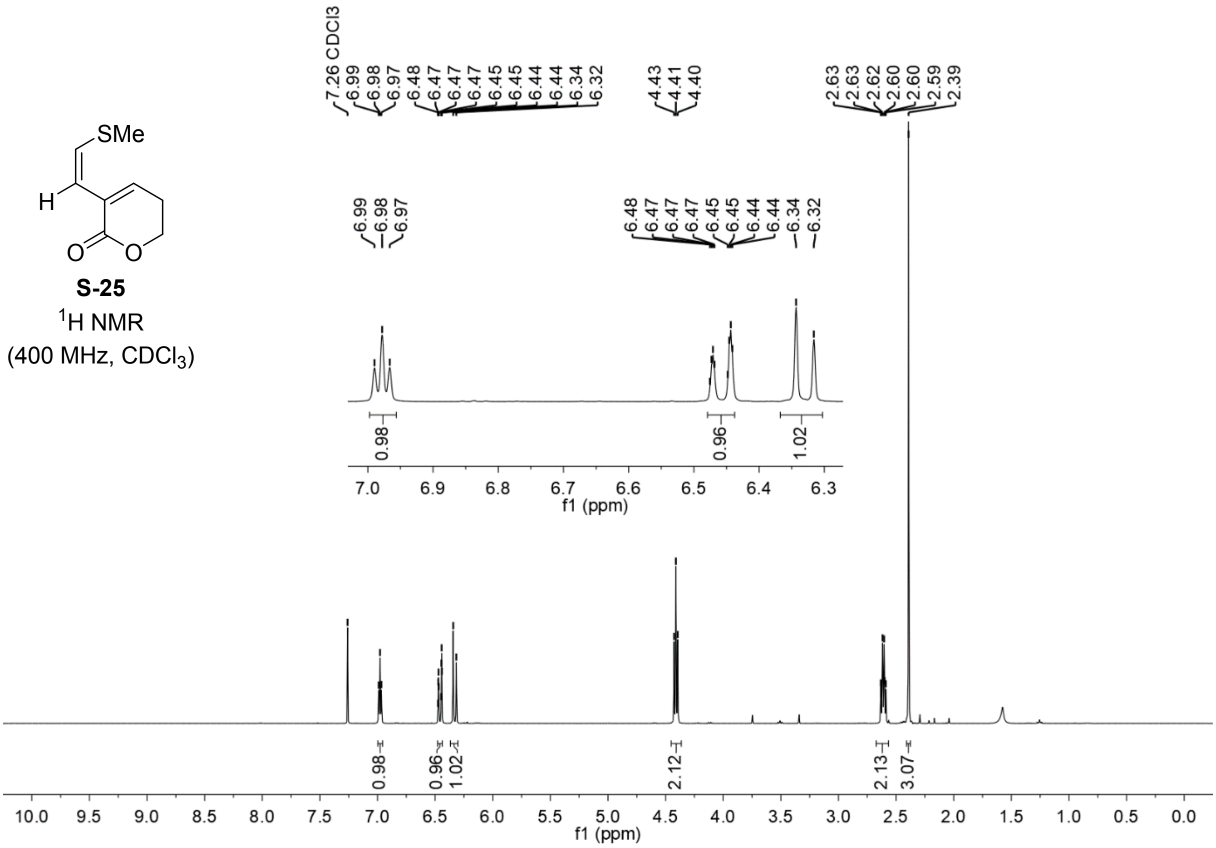

S-25
^{1}H NMR
(400 MHz, $CDCl_3$)

S-25
^{13}C NMR
(101 MHz, $CDCl_3$)

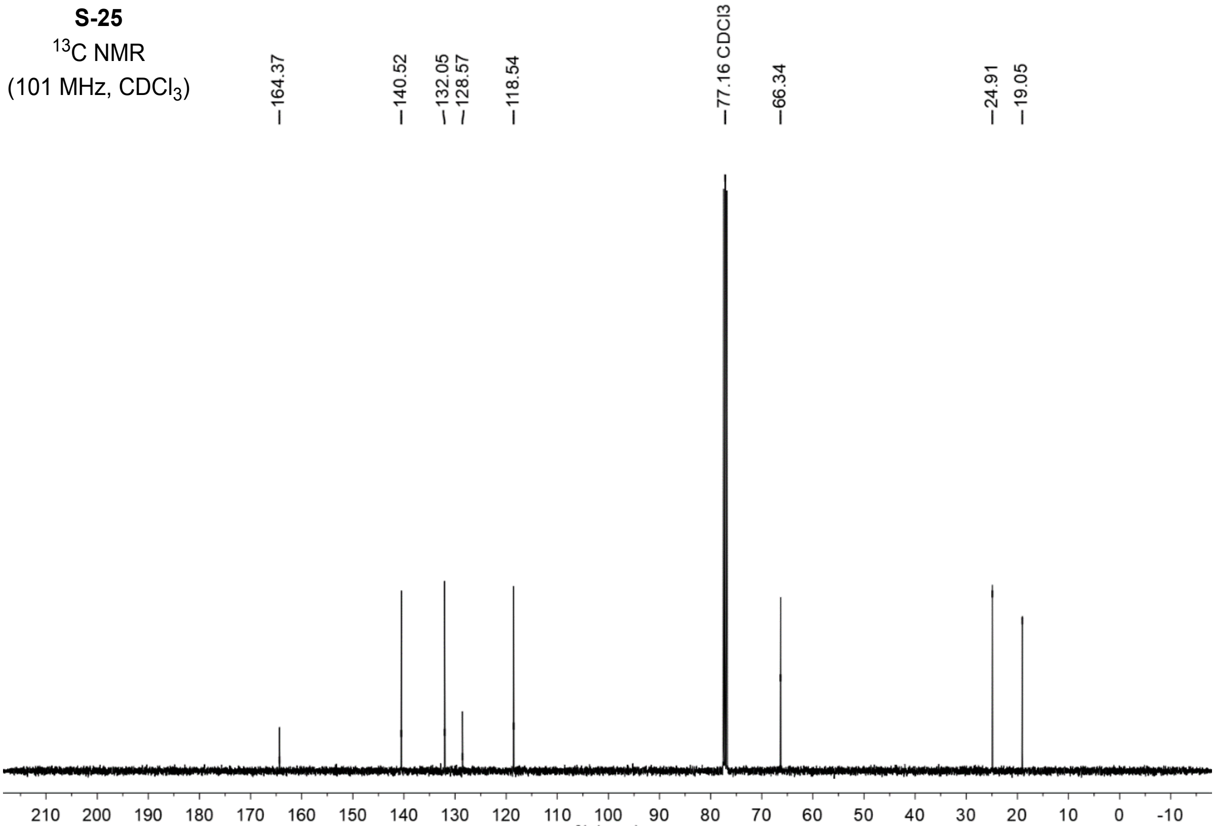

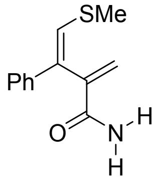

S-26
^{1}H NMR
(400 MHz, $CDCl_3$)

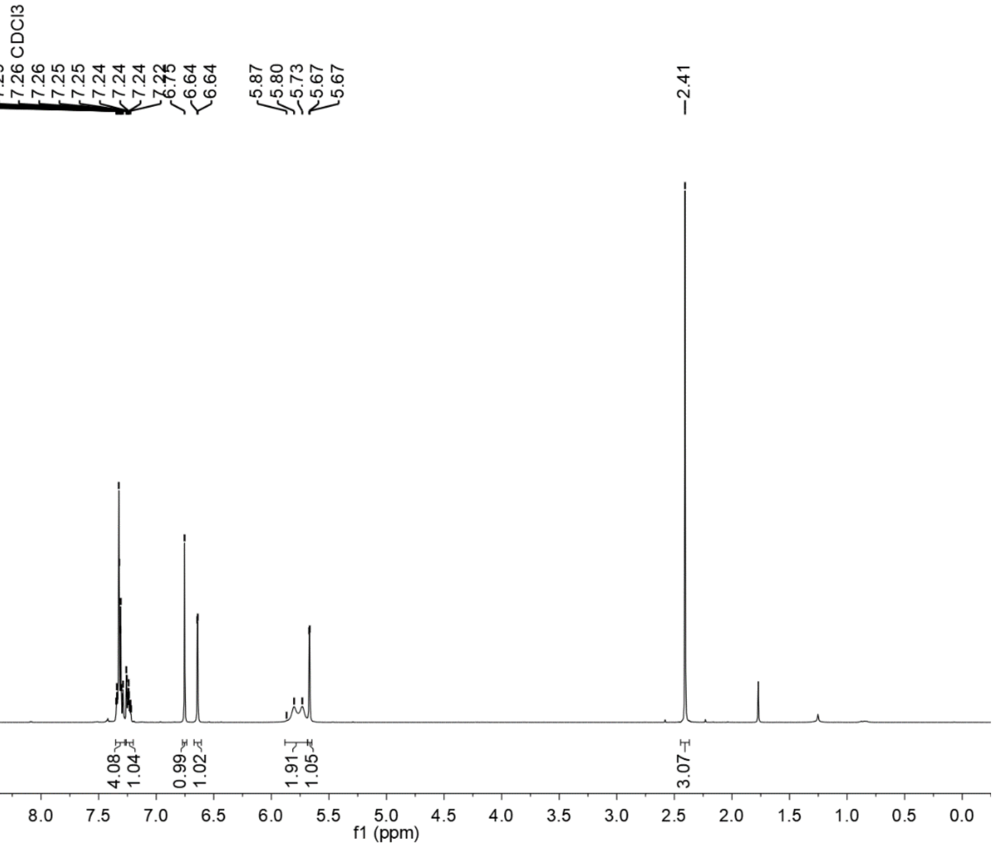

S-26
^{13}C NMR
(101 MHz, $CDCl_3$)

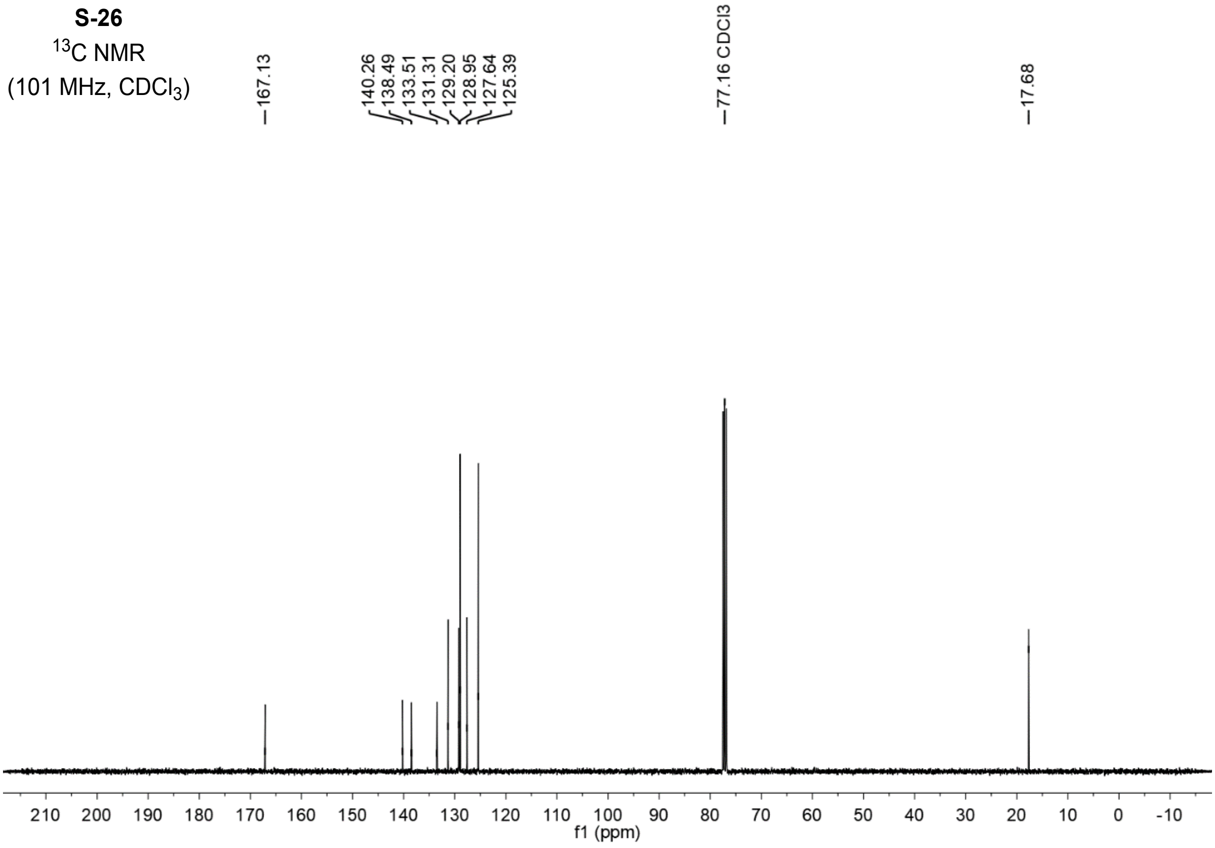

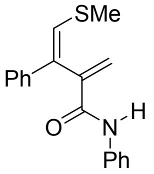

S-27
^{1}H NMR
(400 MHz, $CDCl_3$)

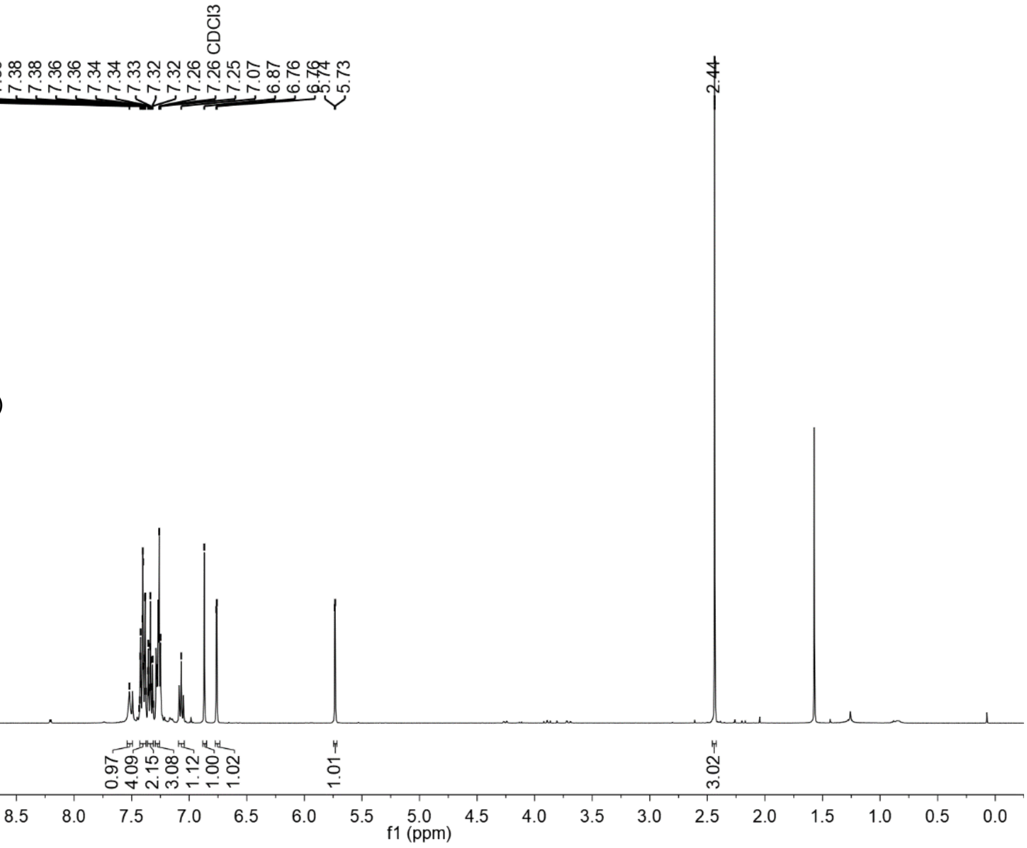

S-27
^{13}C NMR
(101 MHz, $CDCl_3$)

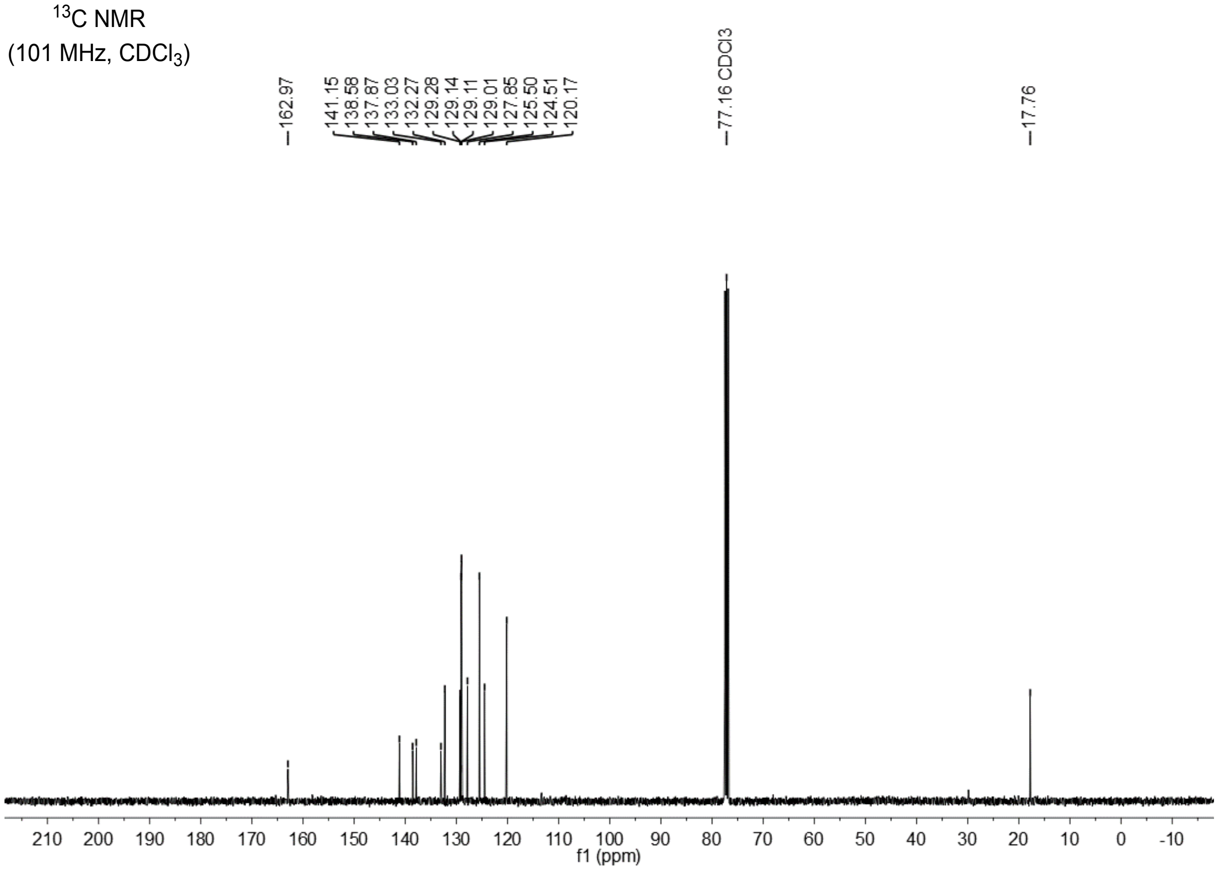

S-32
^{1}H NMR
(400 MHz, $CDCl_3$)

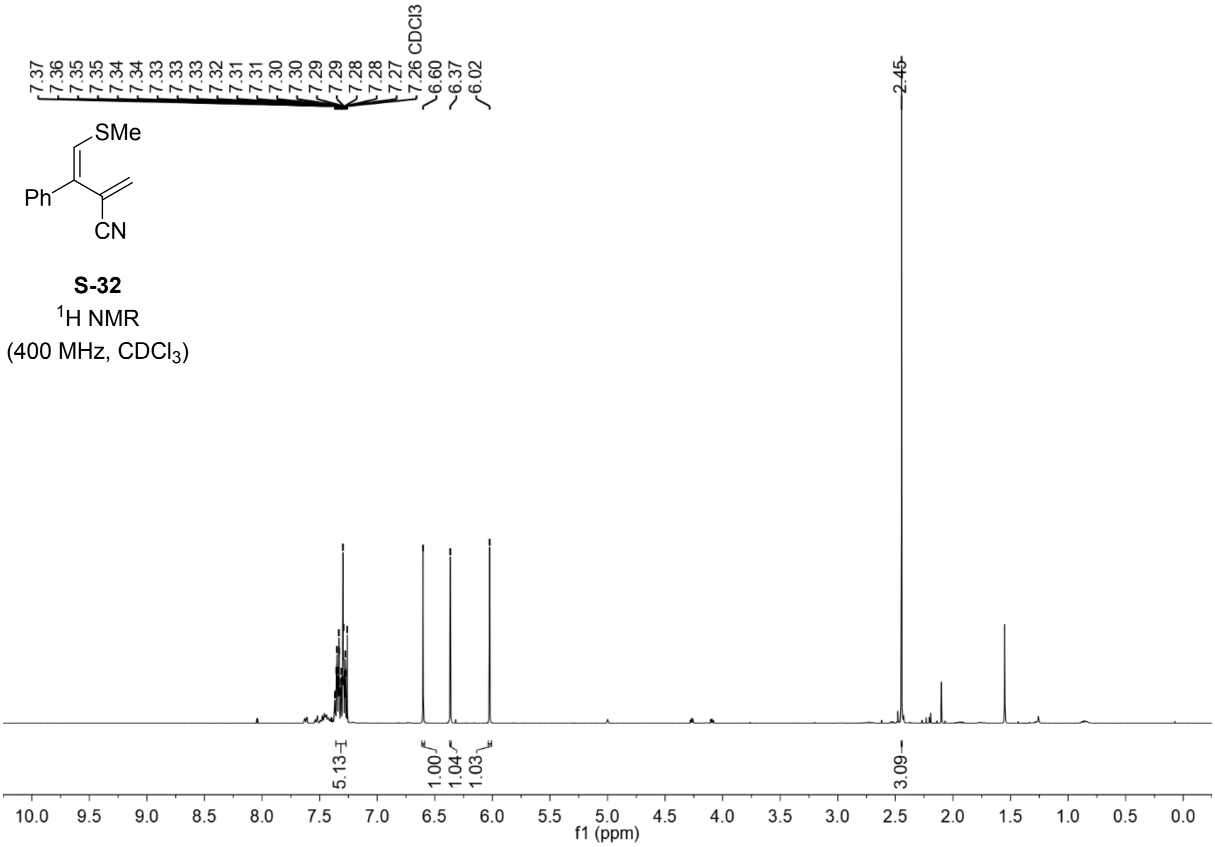

S-32
^{13}C NMR
(101 MHz, $CDCl_3$)

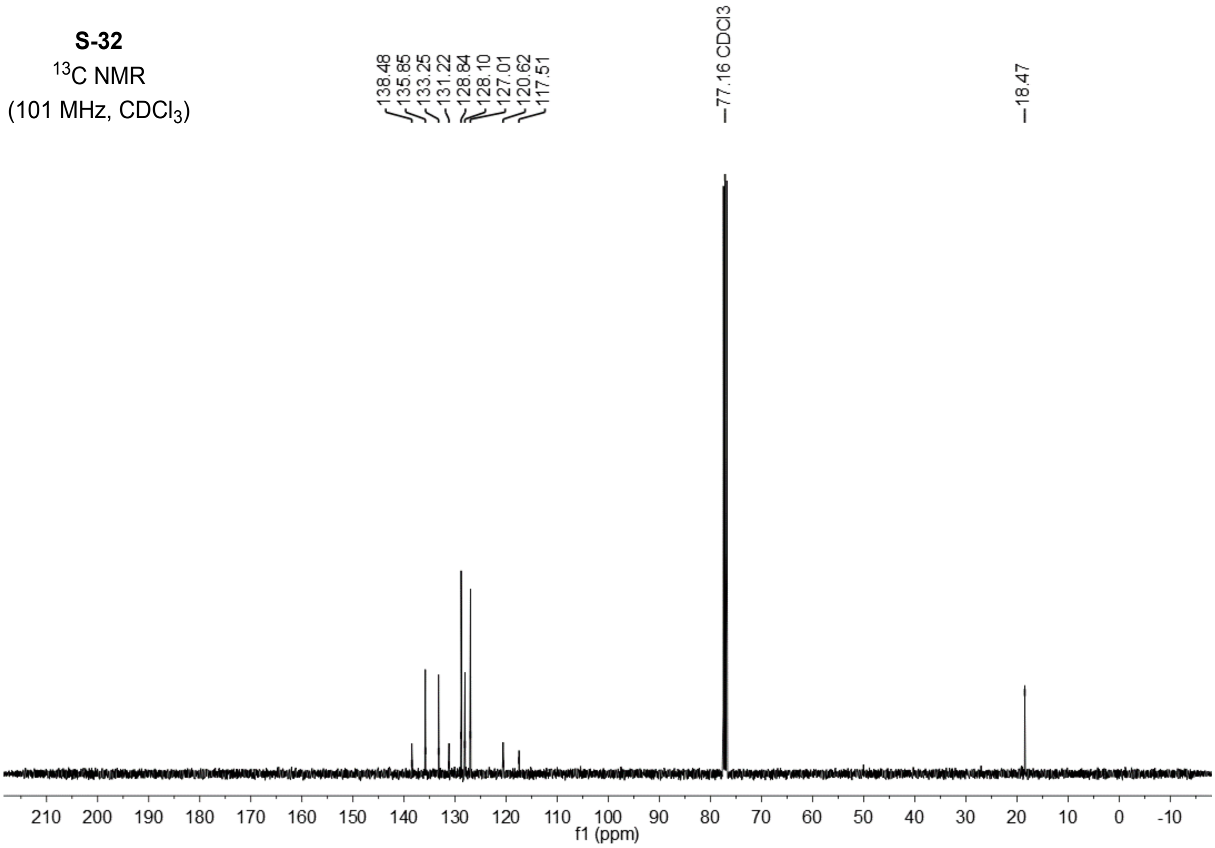

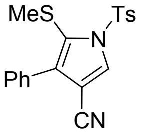

S-33
^{1}H NMR
(400 MHz, $CDCl_3$)

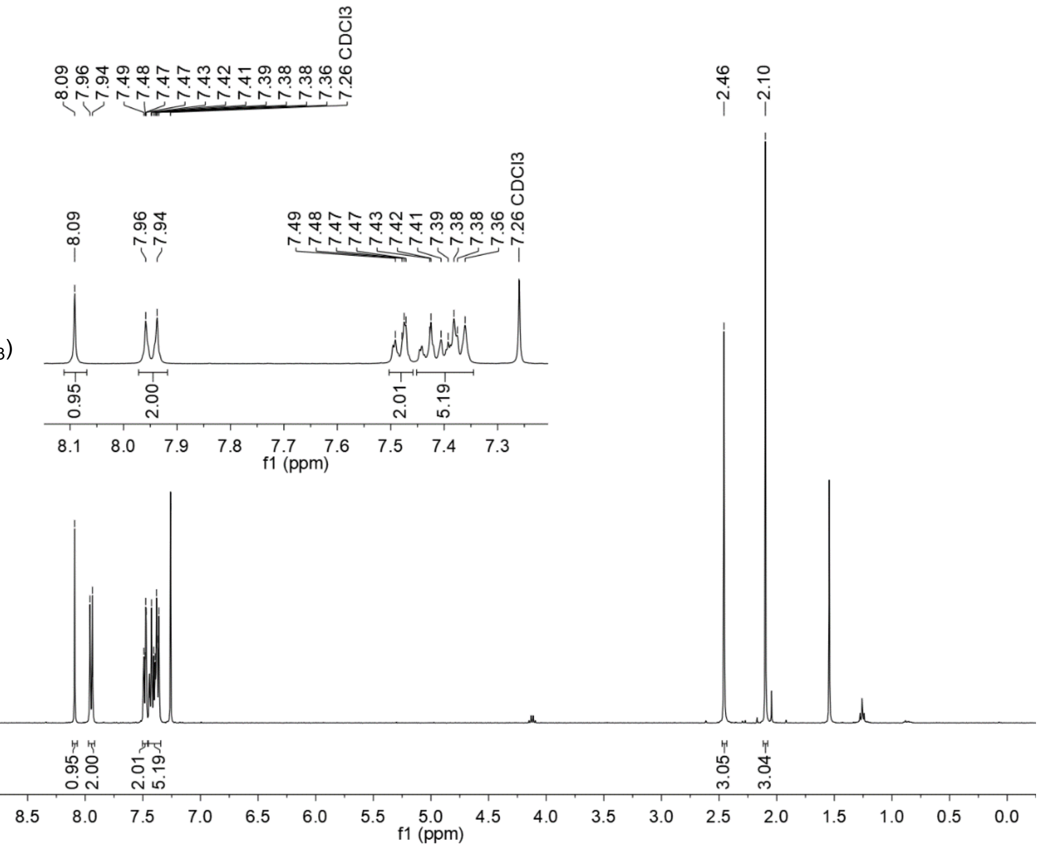

S-33
^{13}C NMR
(101 MHz, $CDCl_3$)

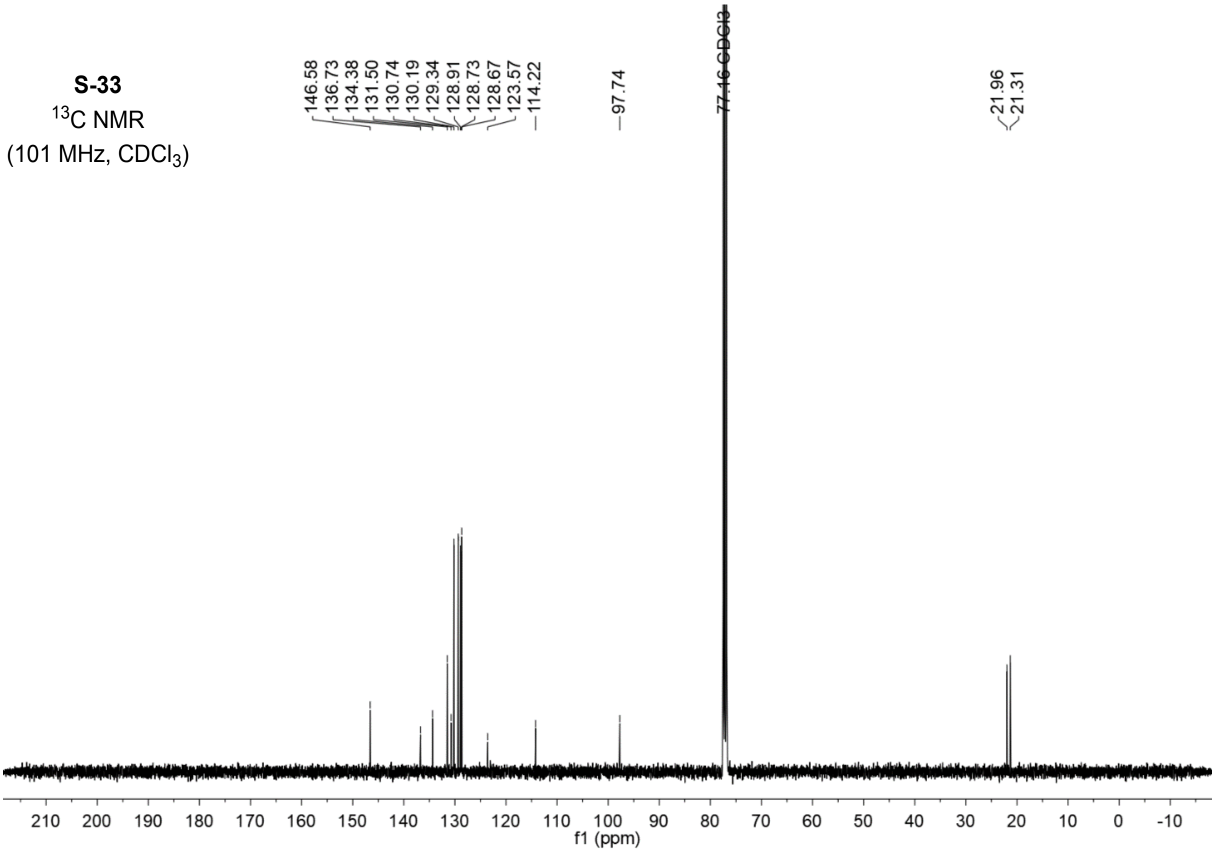

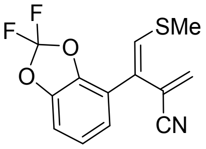

S-34
^{1}H NMR
(400 MHz, $CDCl_3$)

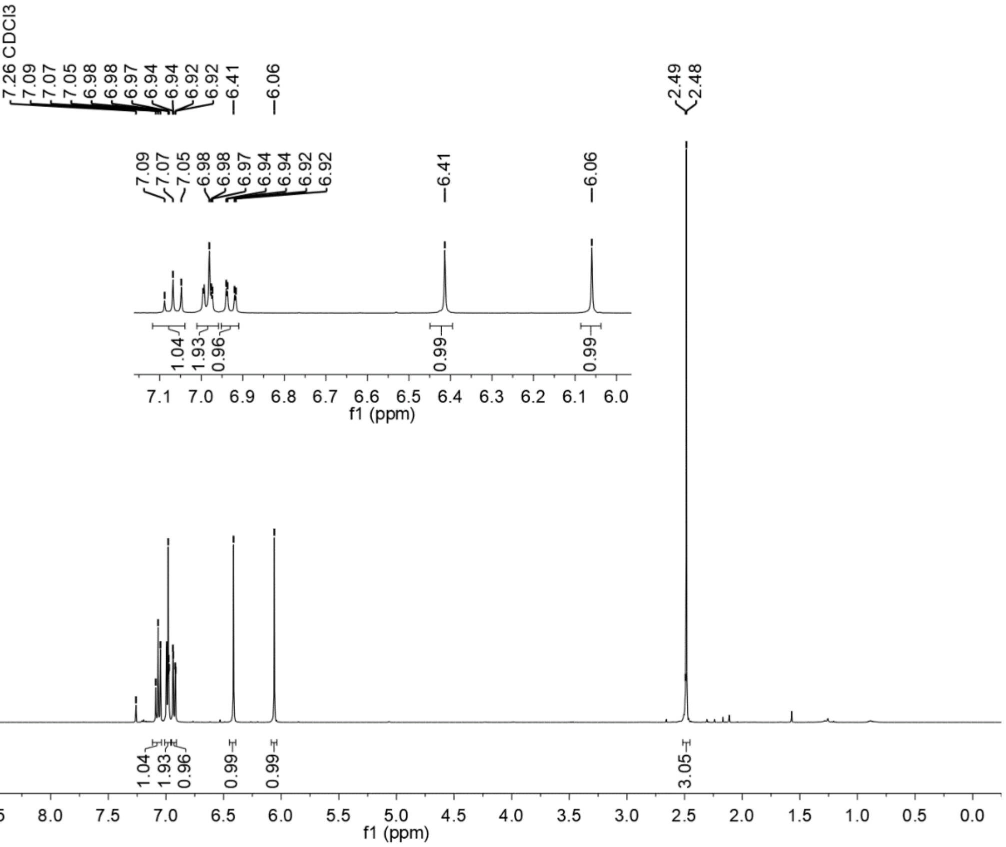

S-34
^{13}C NMR
(101 MHz, $CDCl_3$)

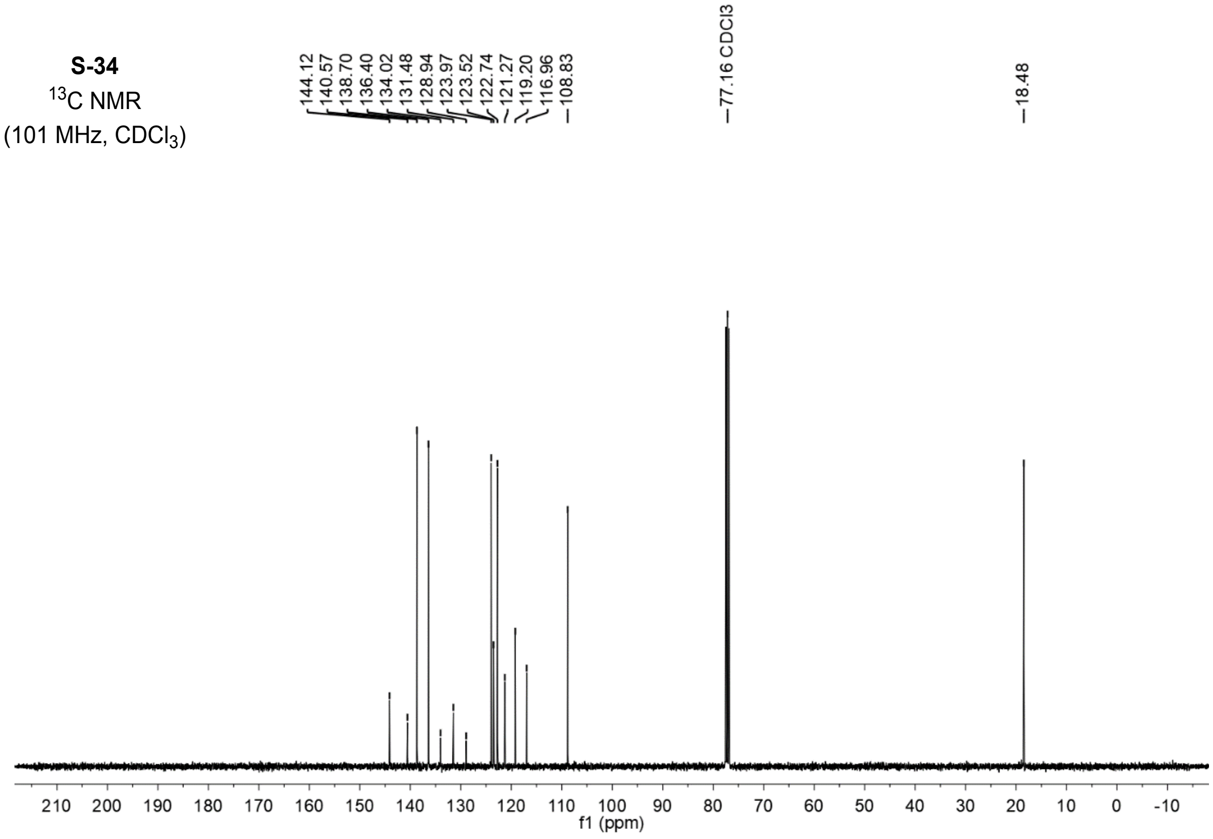

S-34
^{19}F NMR
(376 MHz, $CDCl_3$)

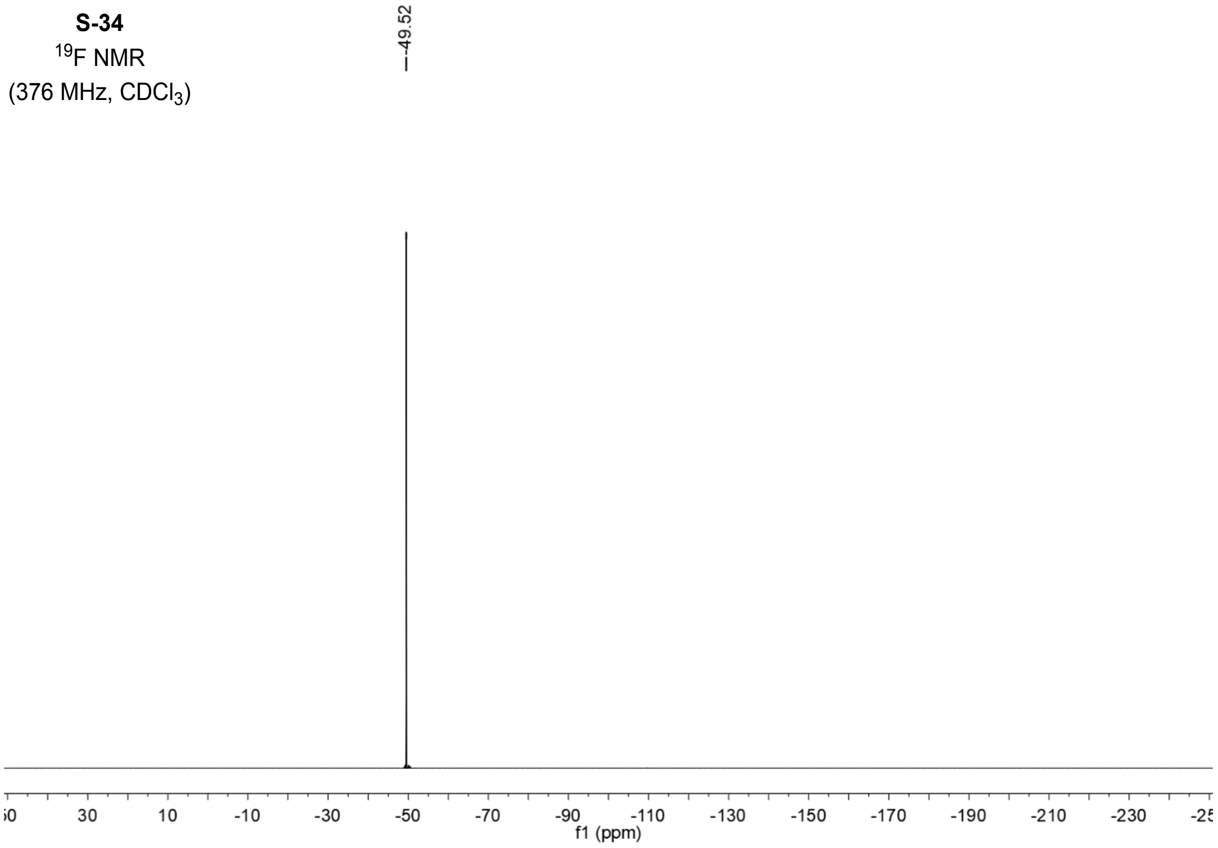

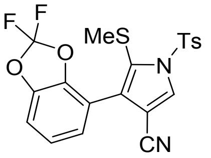

S-35
^{1}H NMR
(400 MHz, $CDCl_3$)

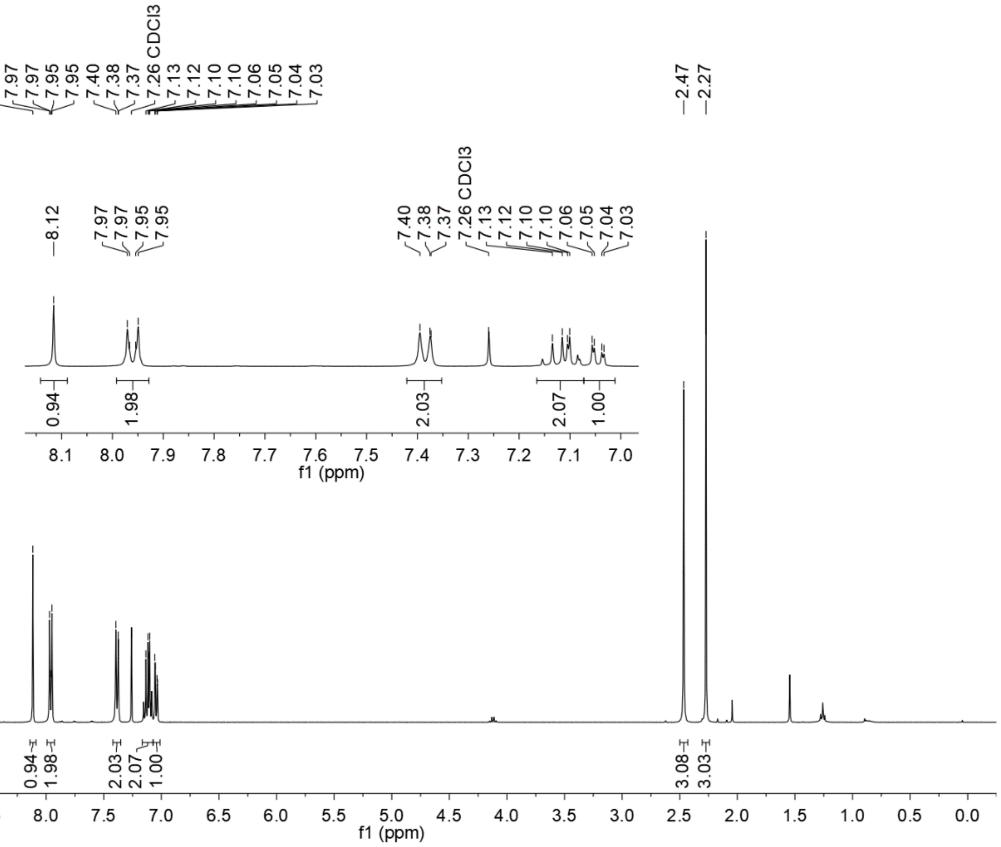

S-35
^{13}C NMR
(101 MHz, $CDCl_3$)

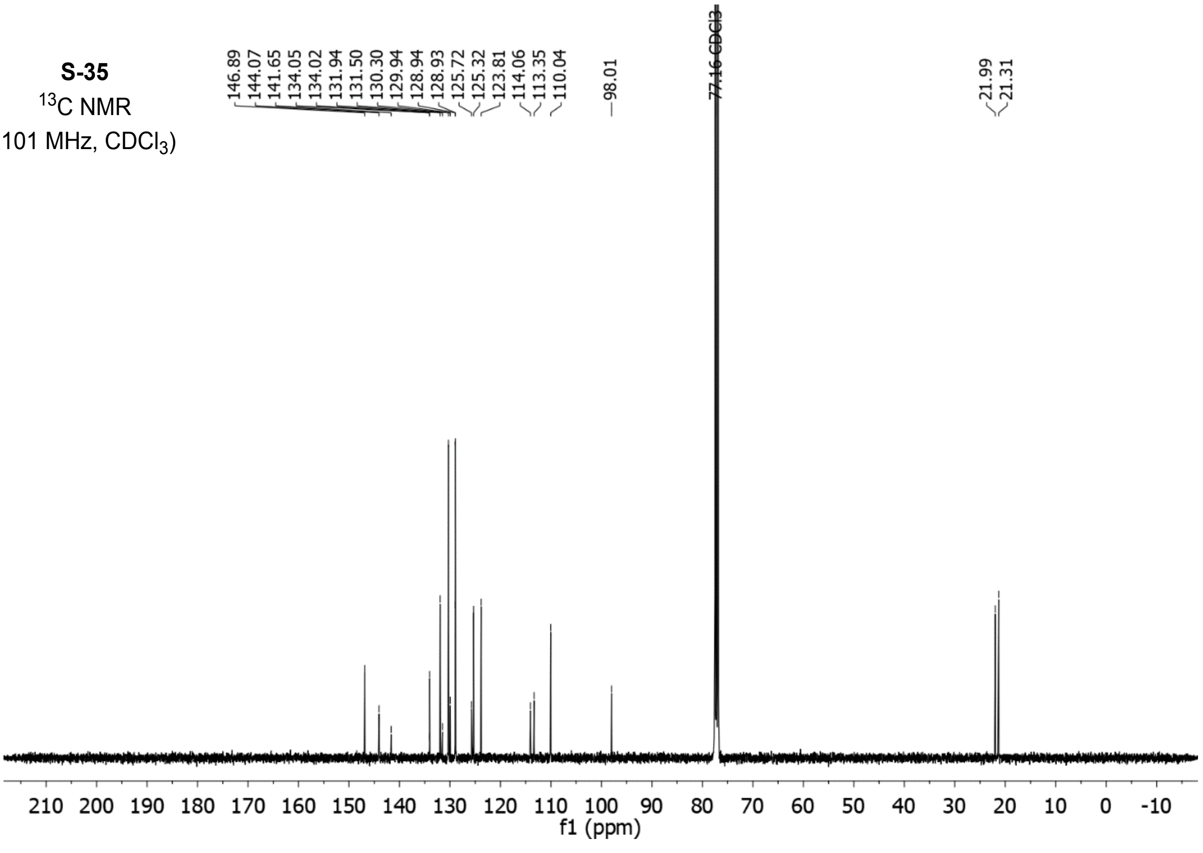

S-35
^{19}F NMR
(376 MHz, $CDCl_3$)

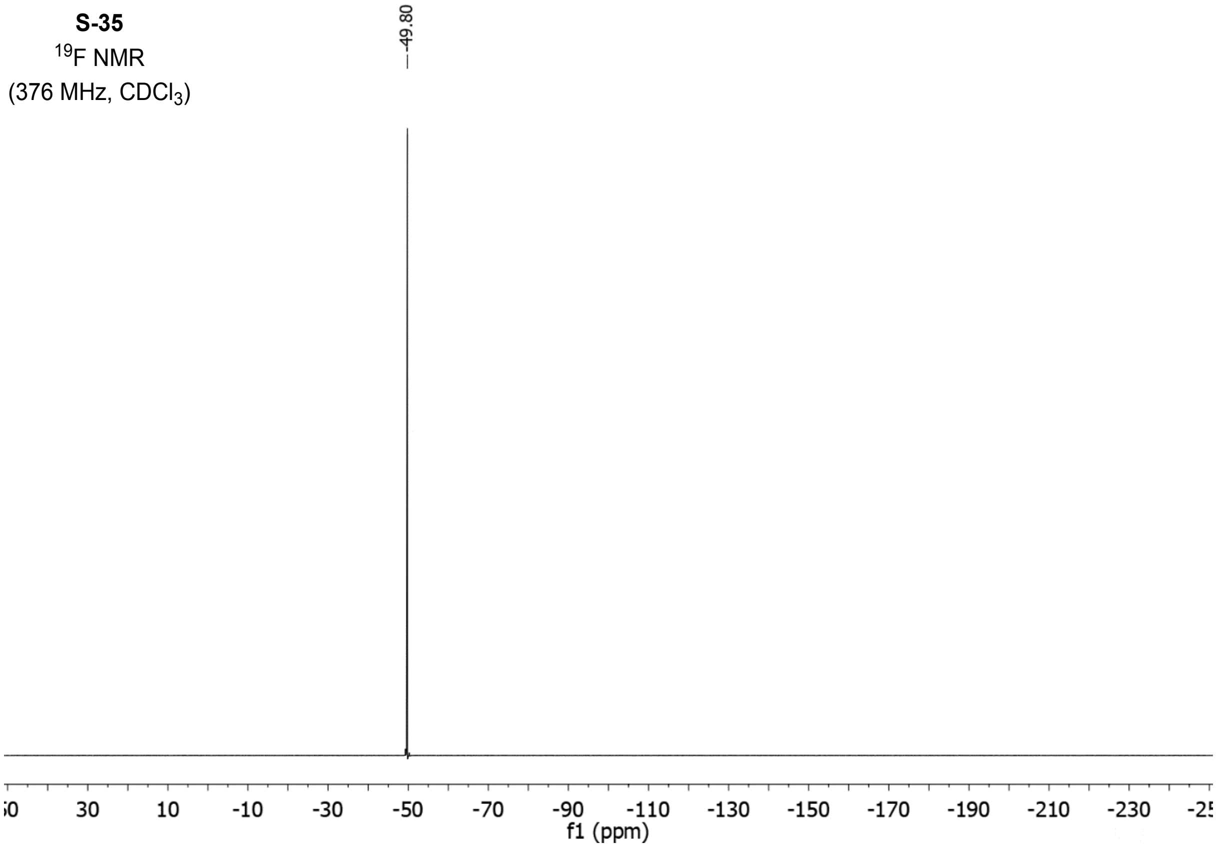

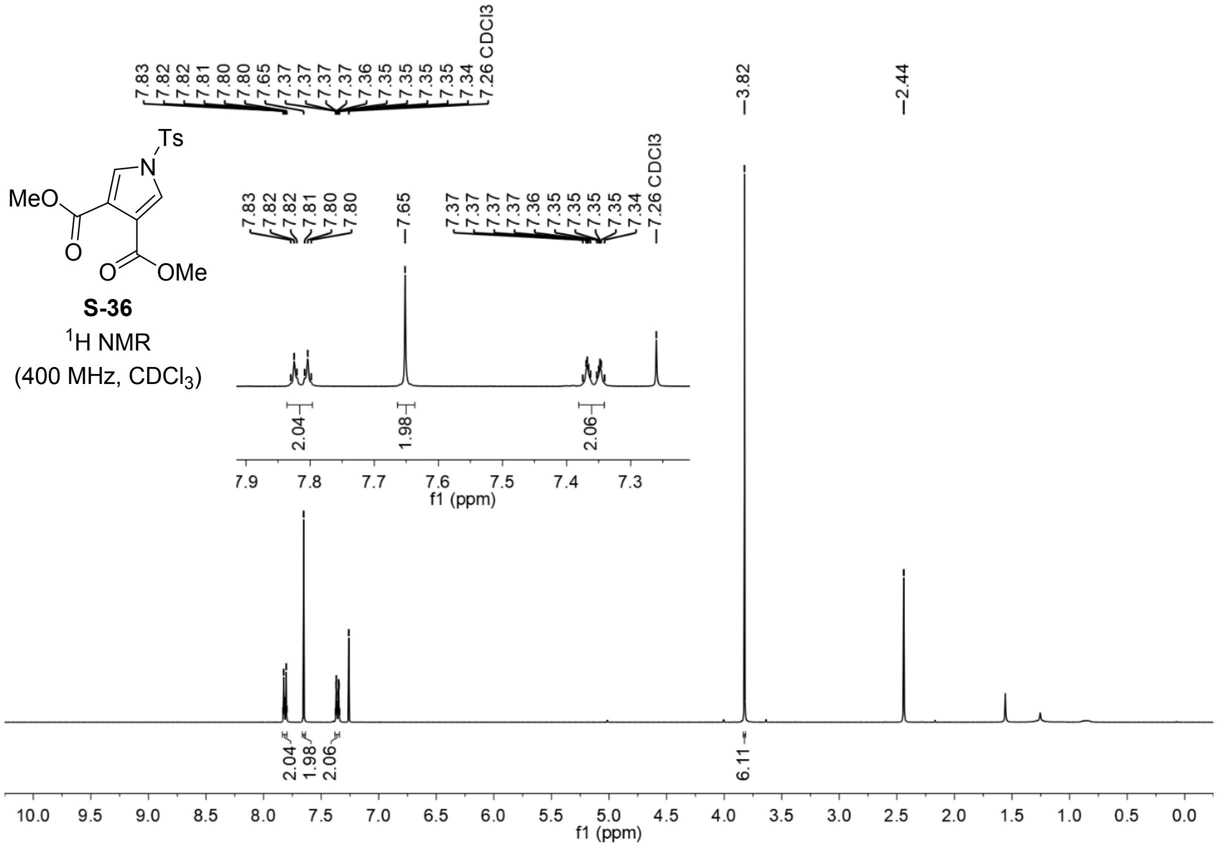
MeO
O
O
OMe
N
Ts
S-36
^{1}H NMR
(400 MHz, CDCl$_3$)
7.83 7.82 7.82 7.81 7.80 7.80 7.65 7.37 7.37 7.37 7.37 7.36 7.35 7.35 7.35 7.35 7.34 7.26 CDCl3
3.82
2.44
2.04
1.98
2.06
6.11
f1 (ppm)

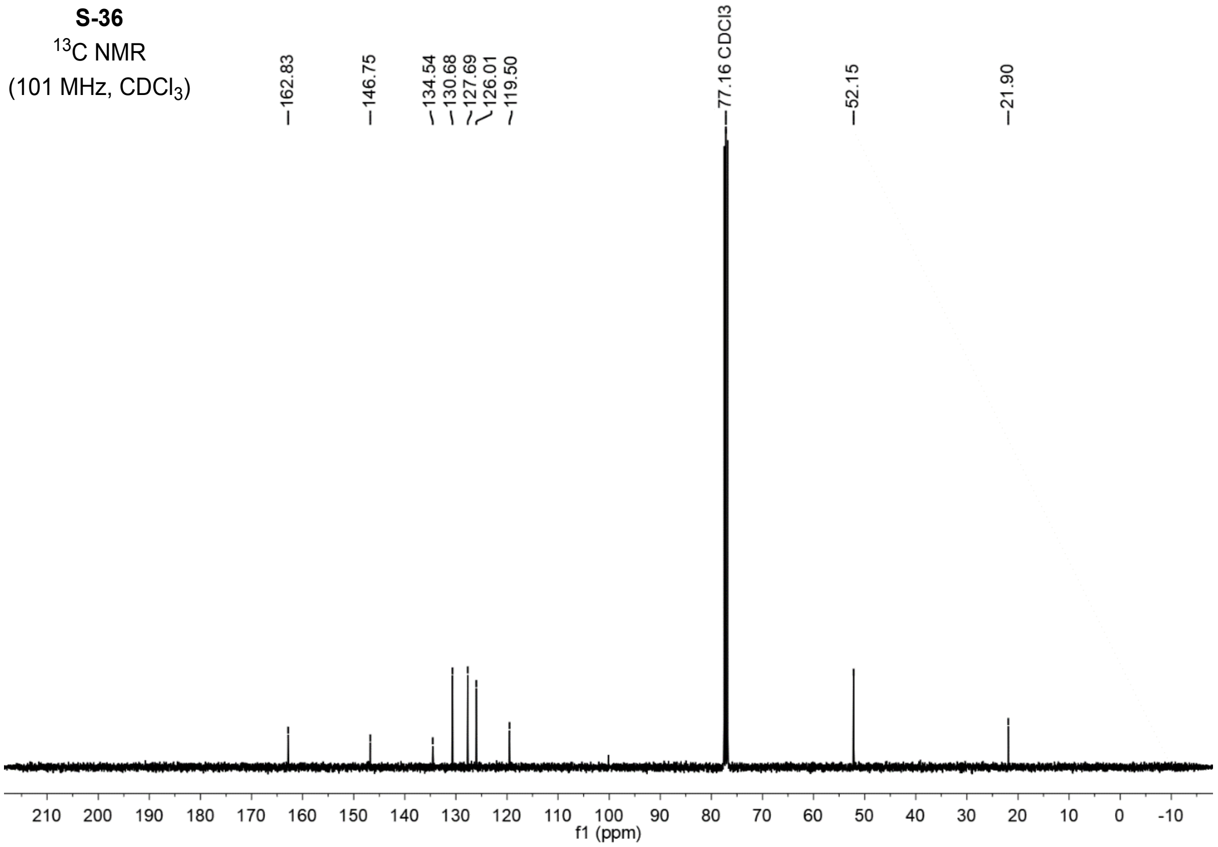
S-36
^{13}C NMR
(101 MHz, CDCl$_3$)
162.83
146.75
134.54
130.68
127.69
126.01
119.50
77.16 CDCl3
52.15
21.90
f1 (ppm)

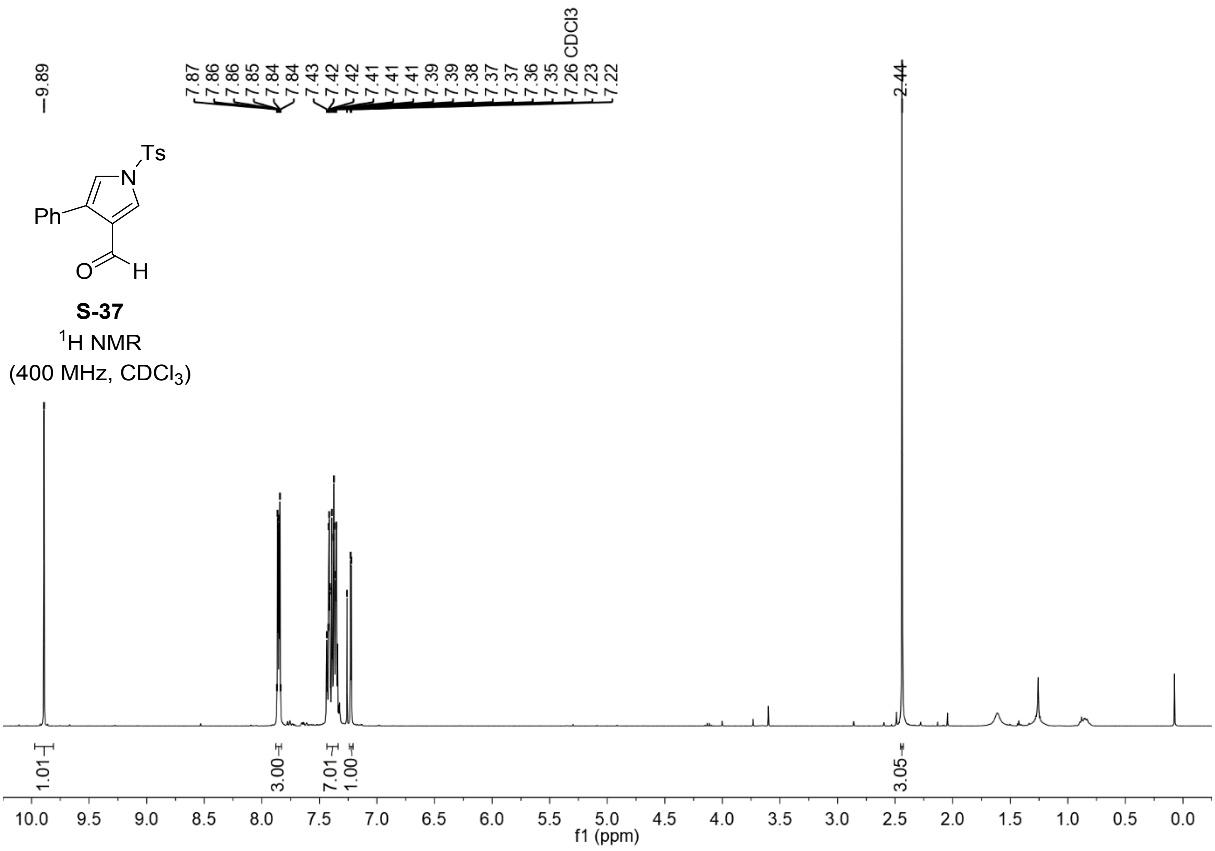

S-37
^{1}H NMR
(400 MHz, $CDCl_3$)

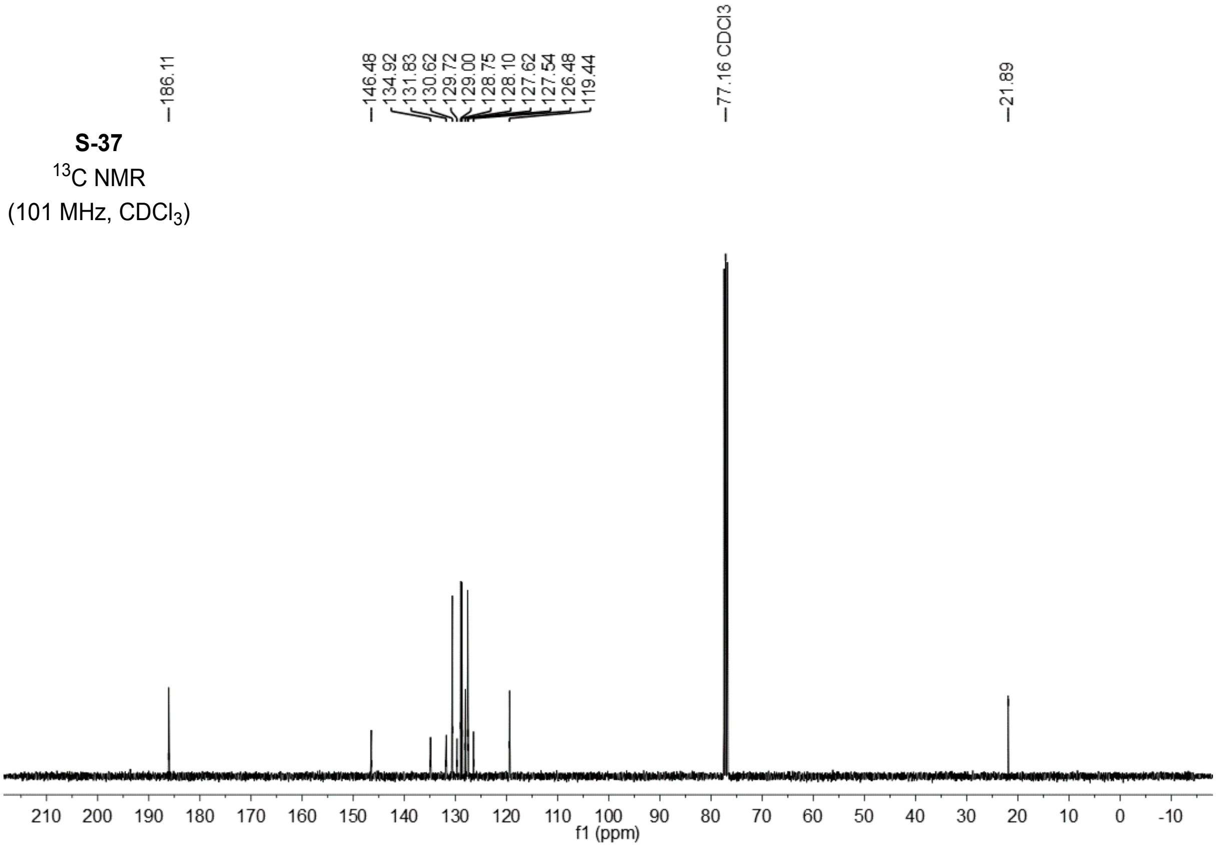

S-37
^{13}C NMR
(101 MHz, $CDCl_3$)

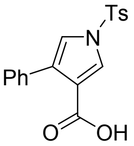

S-38
^{1}H NMR
(400 MHz, CD_2Cl_2)

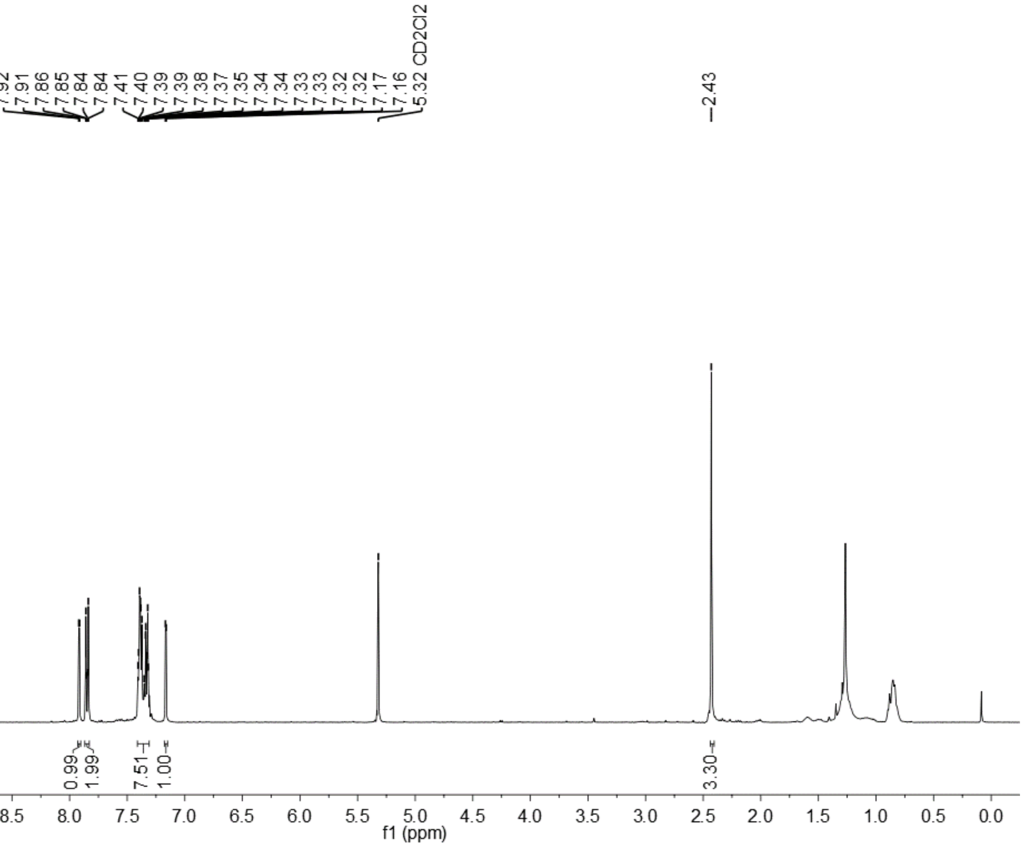

S-38
^{13}C NMR
(101 MHz, CD_2Cl_2)

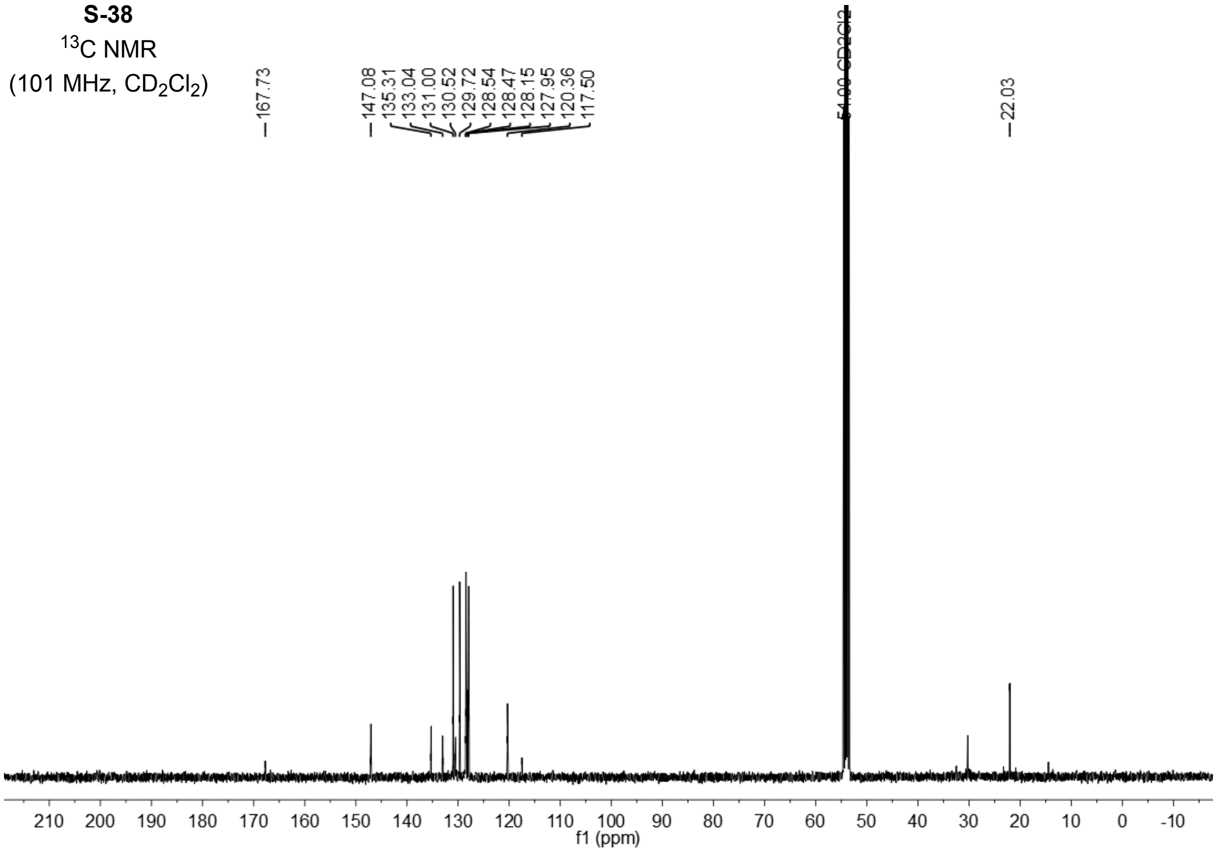

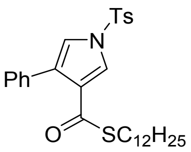

S-39
^{1}H NMR
(400 MHz, $CDCl_3$)

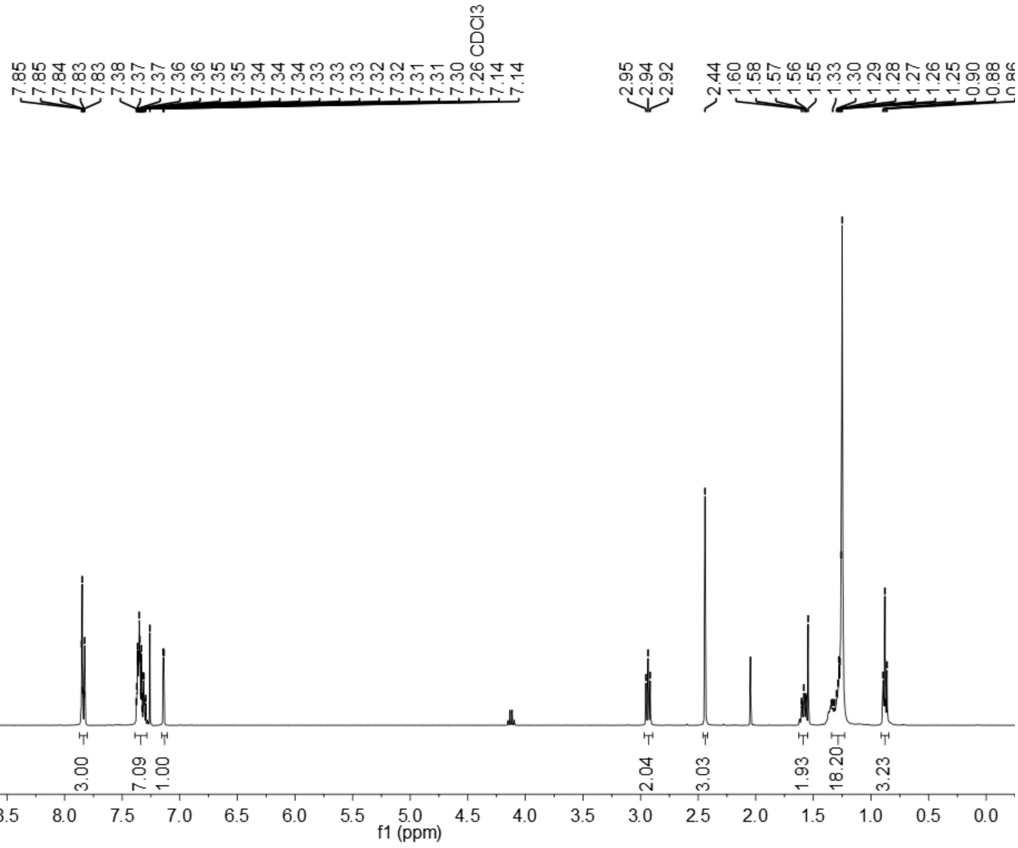

S-39
^{13}C NMR
(101 MHz, $CDCl_3$)

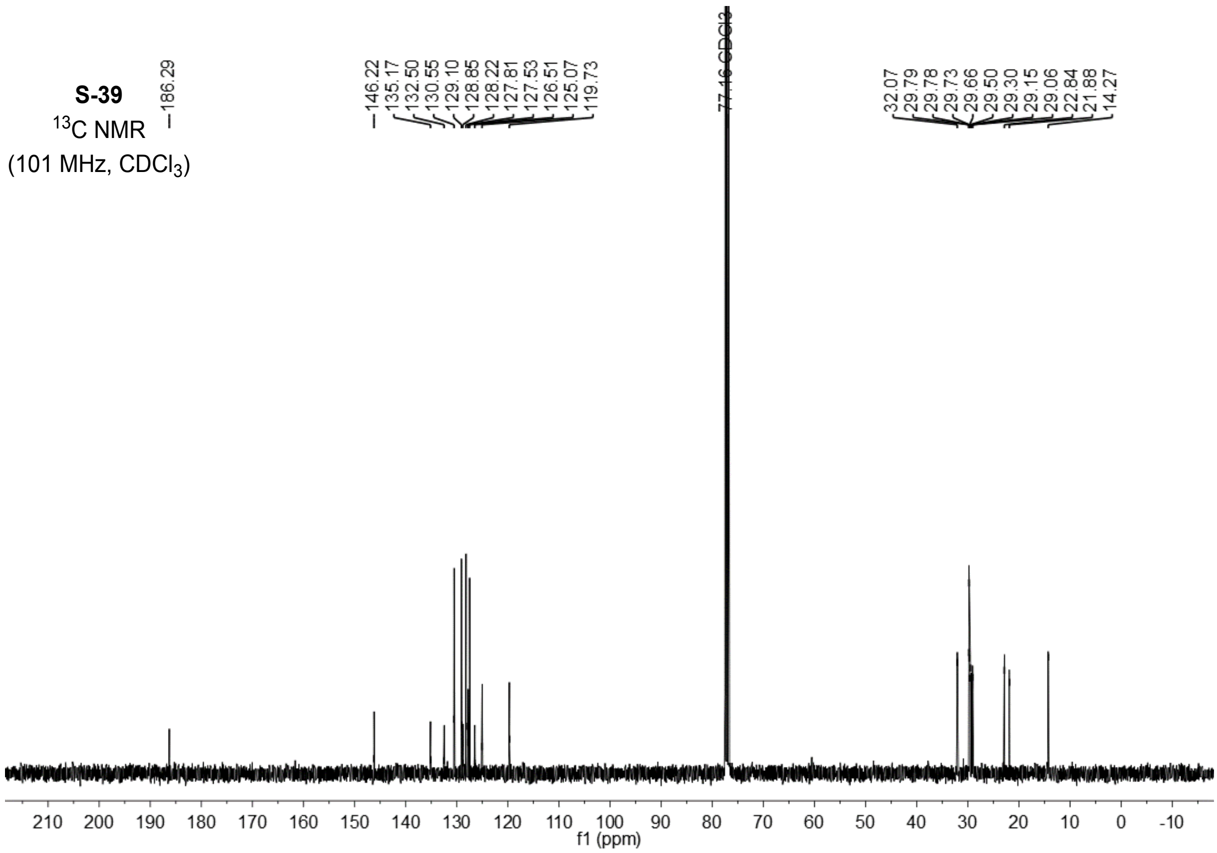

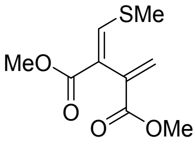

S-44
^{1}H NMR
(400 MHz, $CDCl_3$)

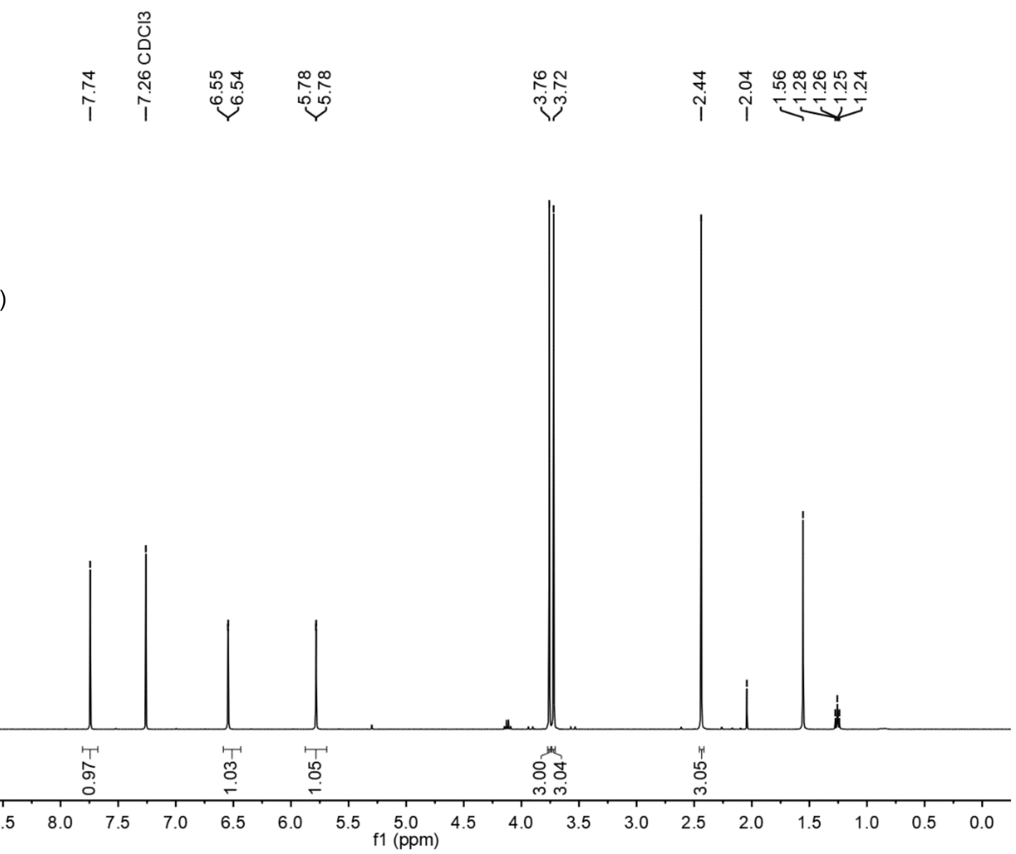

S-44
^{13}C NMR
(101 MHz, $CDCl_3$)

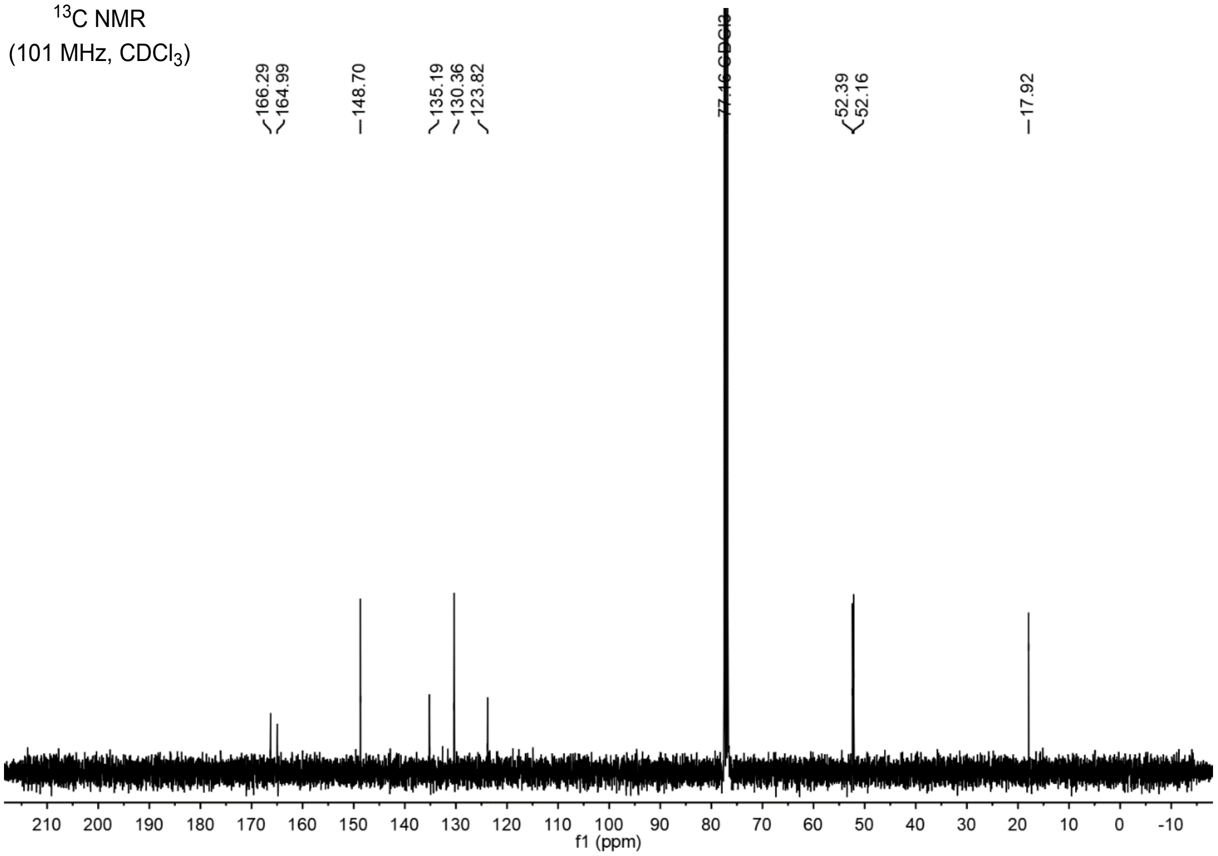

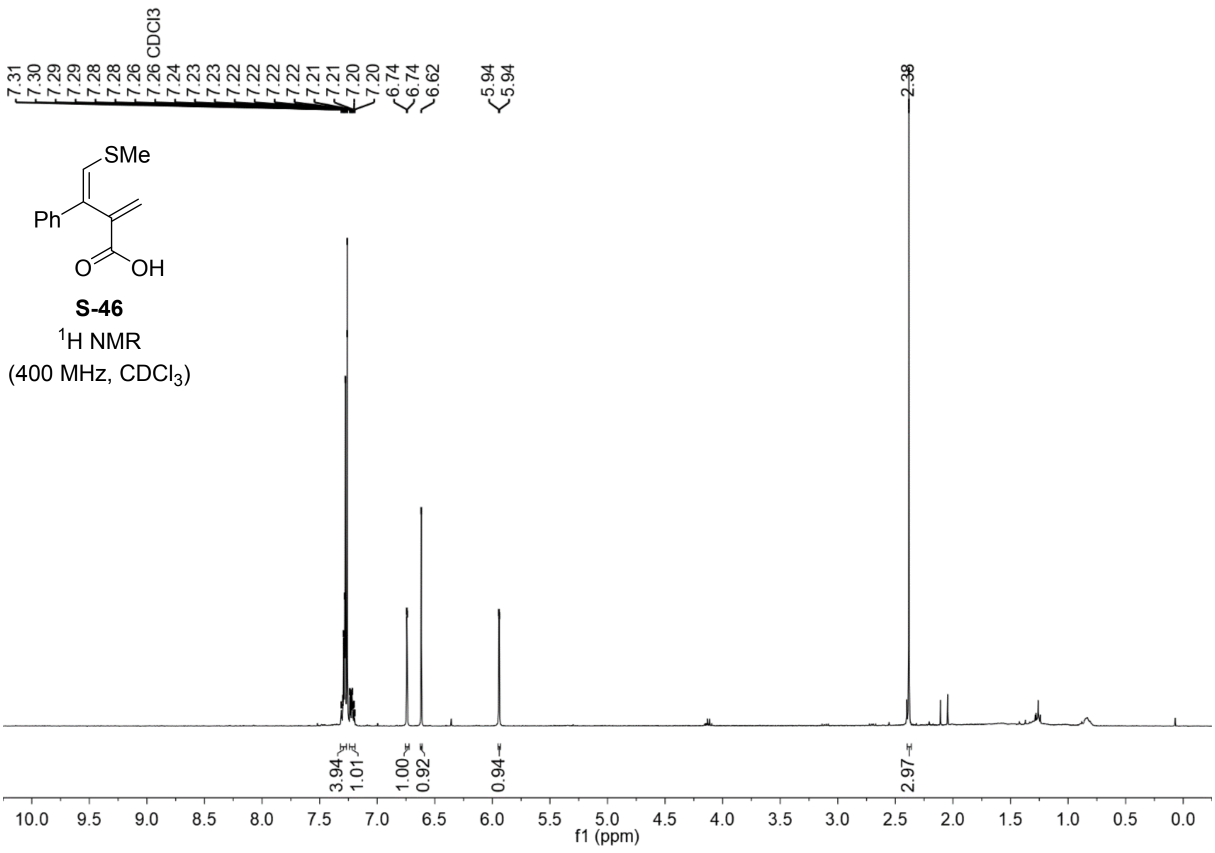

S-46
^{1}H NMR
(400 MHz, $CDCl_3$)

S-46
^{13}C NMR
(101 MHz, $CDCl_3$)

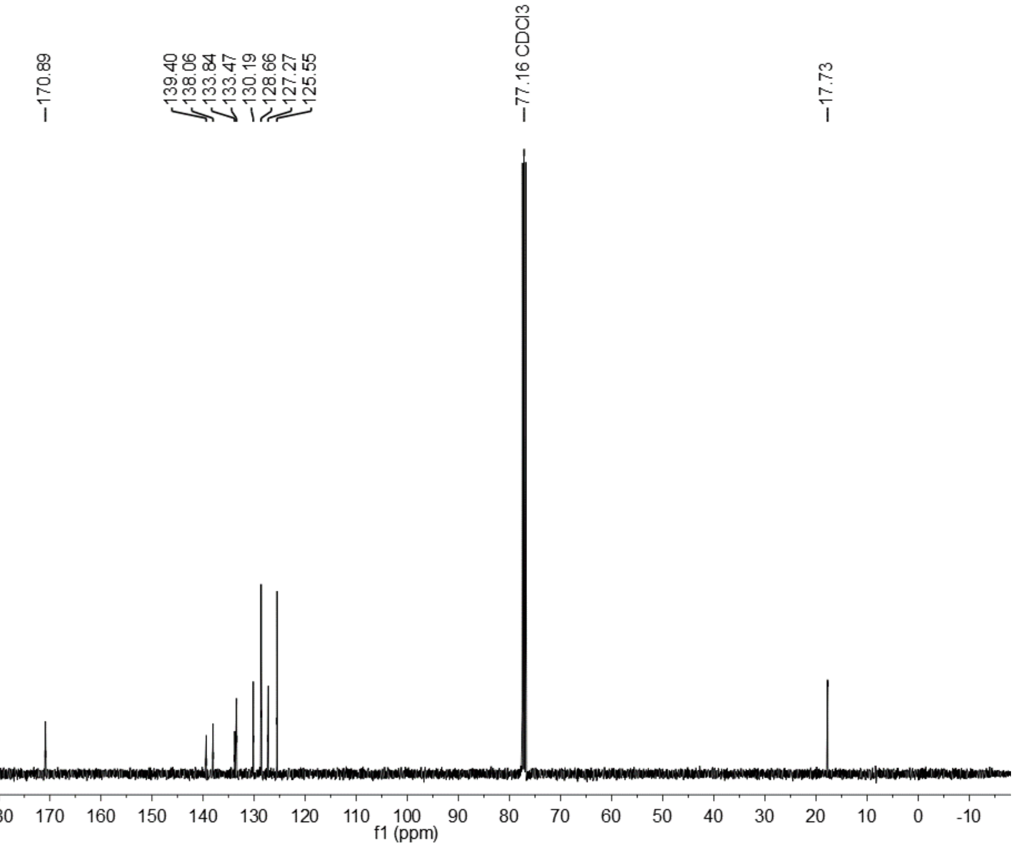

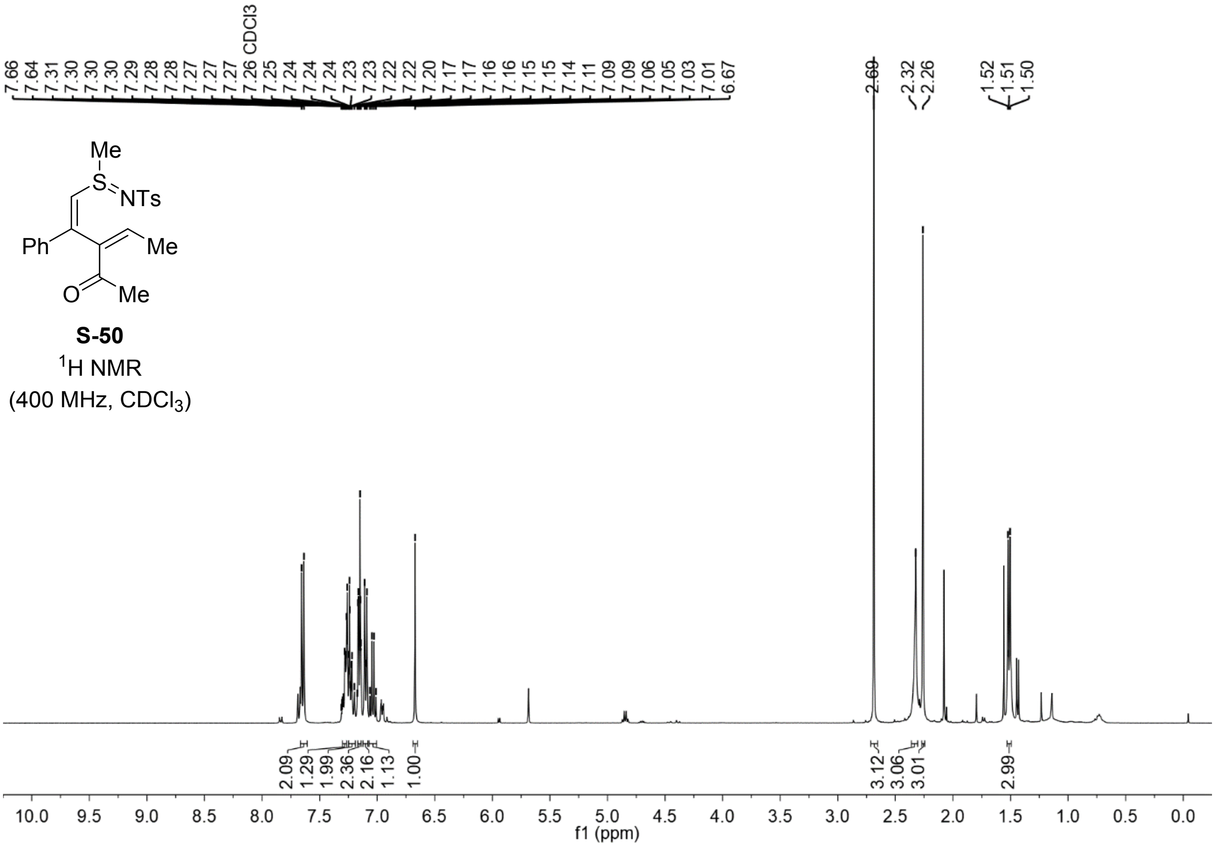

S-50
^{1}H NMR
(400 MHz, $CDCl_3$)

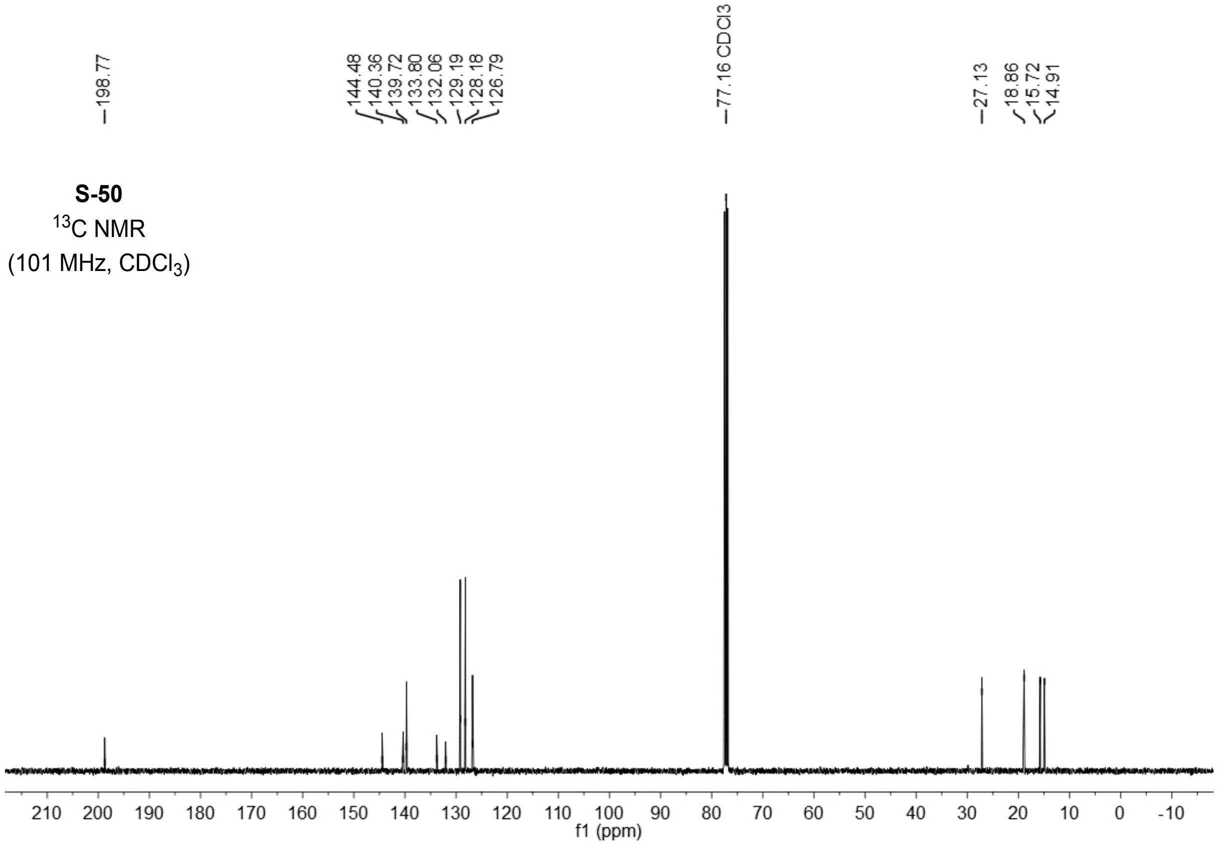

S-50
^{13}C NMR
(101 MHz, $CDCl_3$)

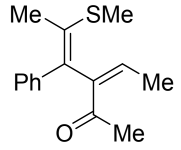

S-51
^{1}H NMR
(400 MHz, $CDCl_3$)

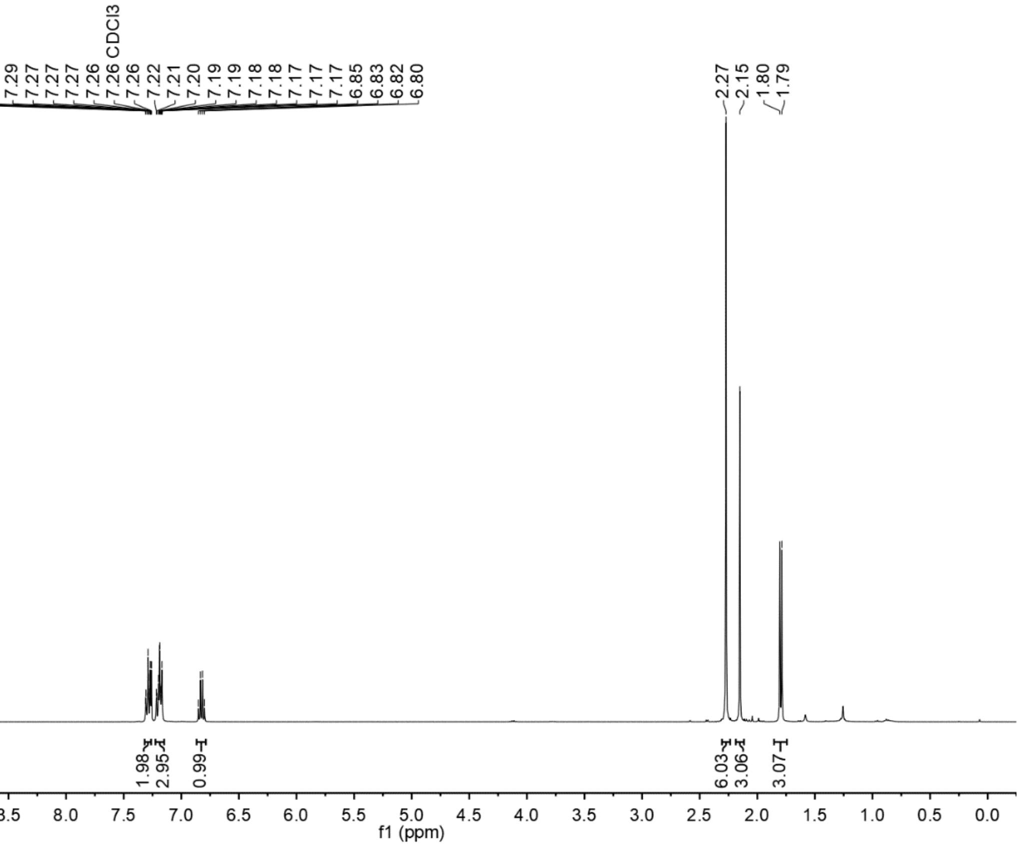

S-51
^{13}C NMR
(101 MHz, $CDCl_3$)

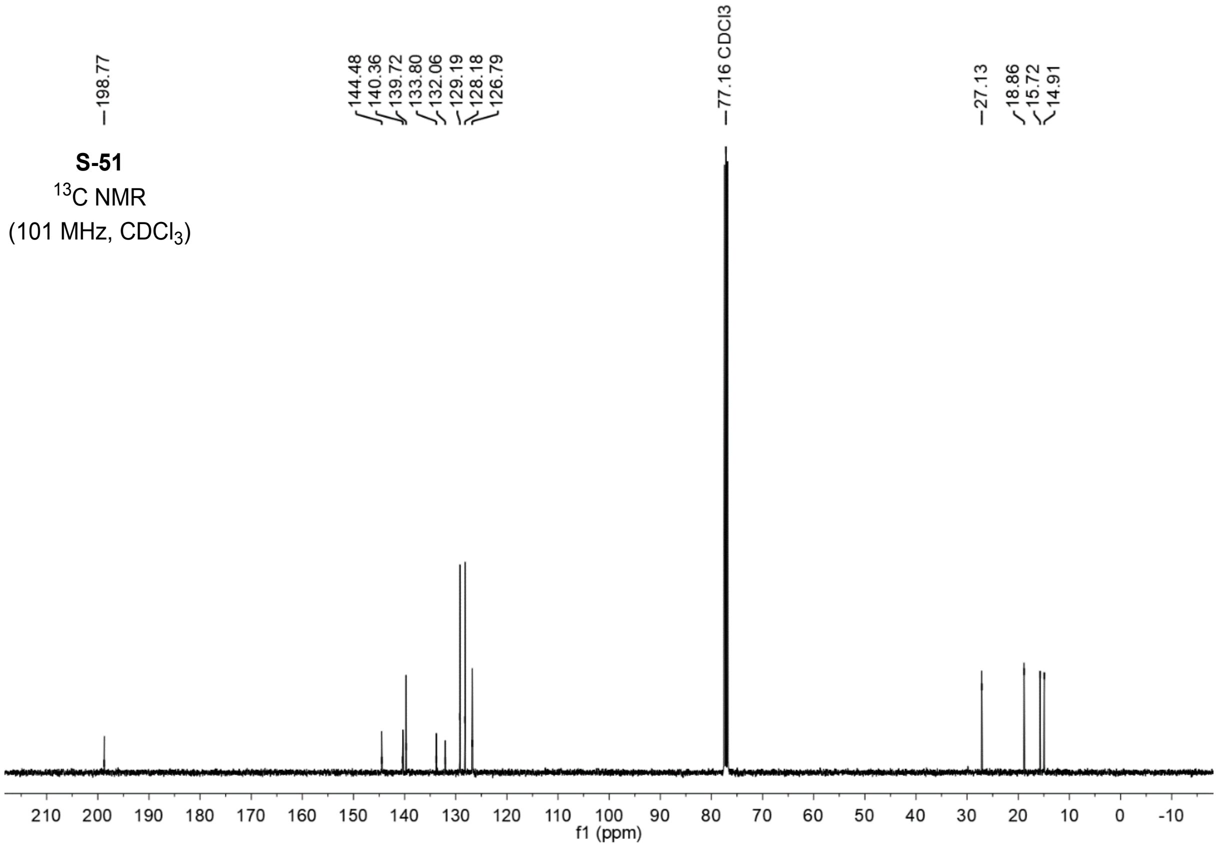

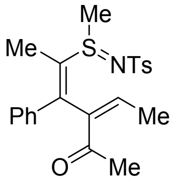

S-52
^{1}H NMR
(400 MHz, $CDCl_3$)

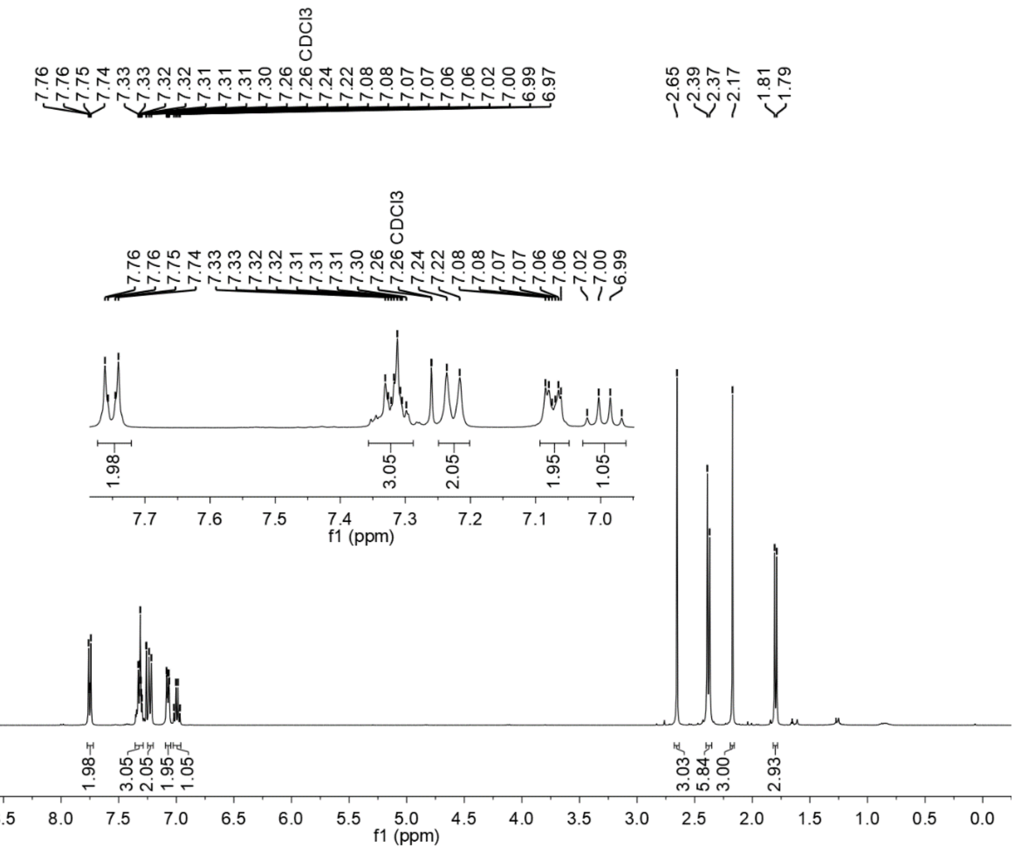

S-52
^{13}C NMR
(101 MHz, $CDCl_3$)

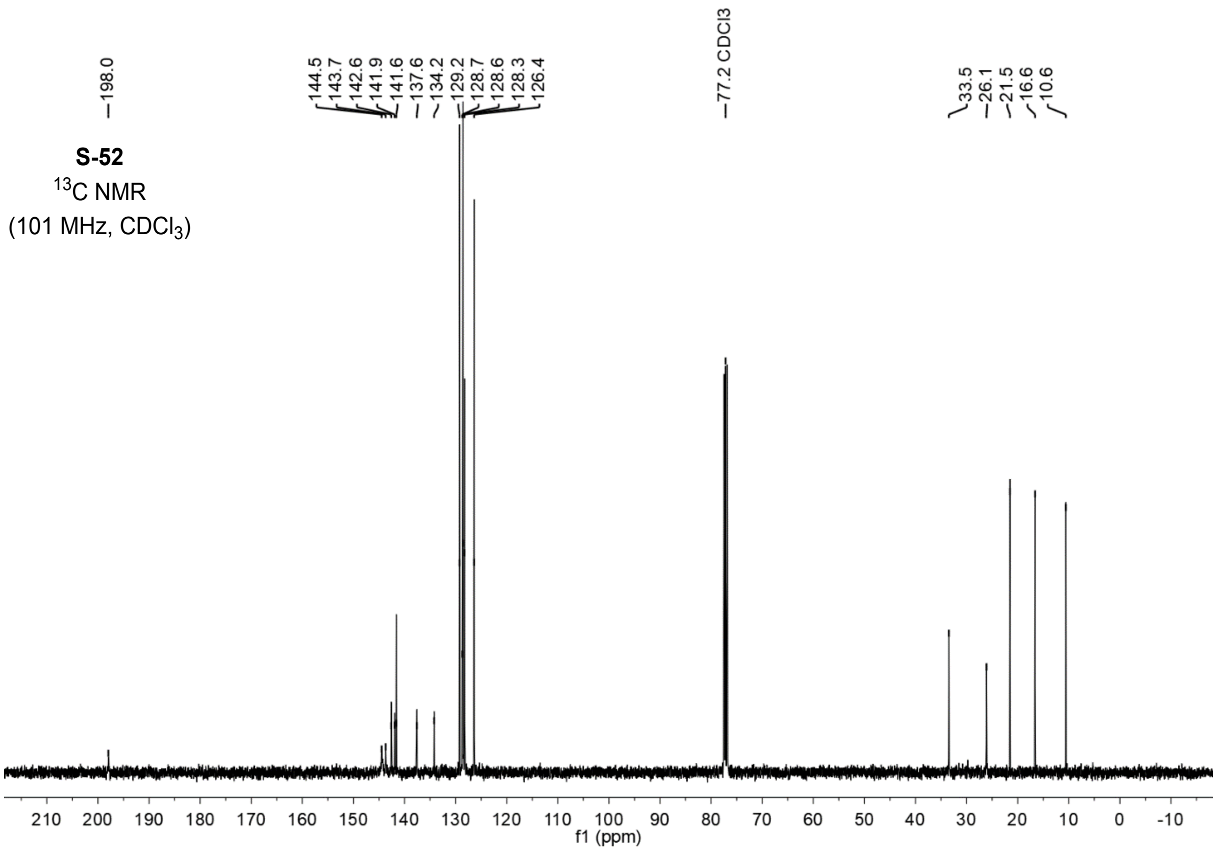

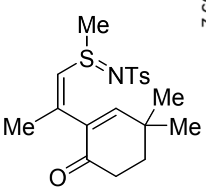

S-54
^{1}H NMR
(400 MHz, $CDCl_3$)

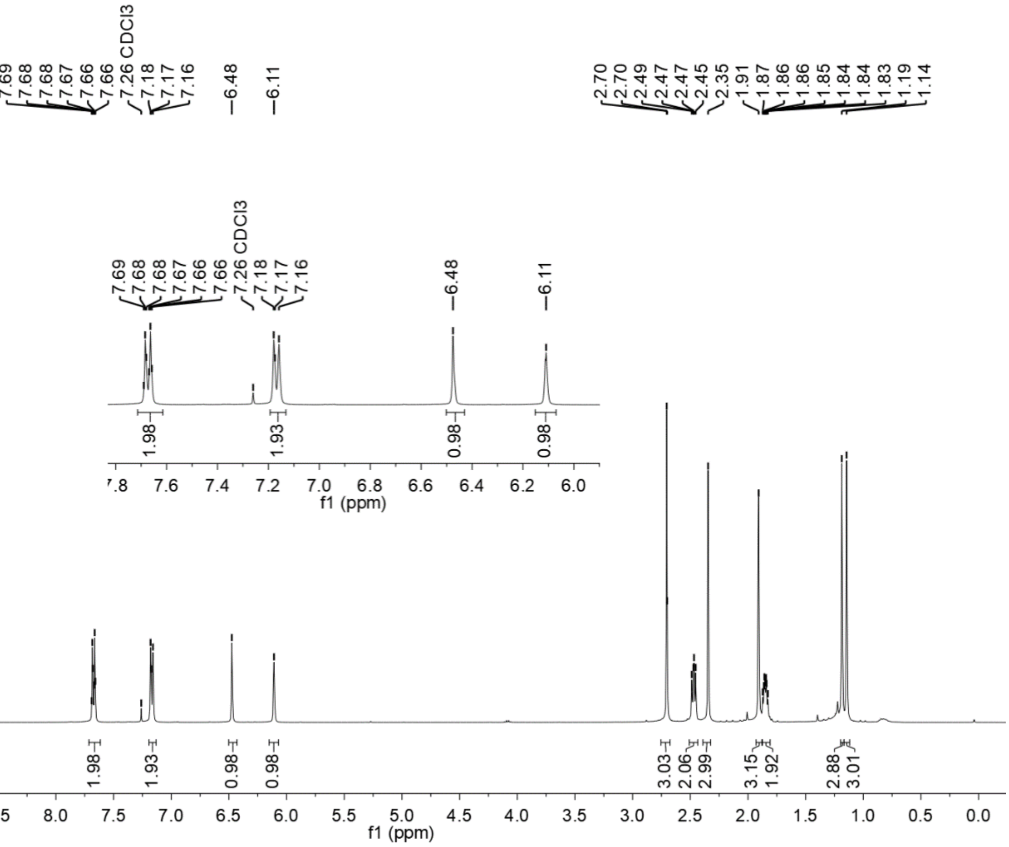

S-54
^{13}C NMR
(101 MHz, $CDCl_3$)

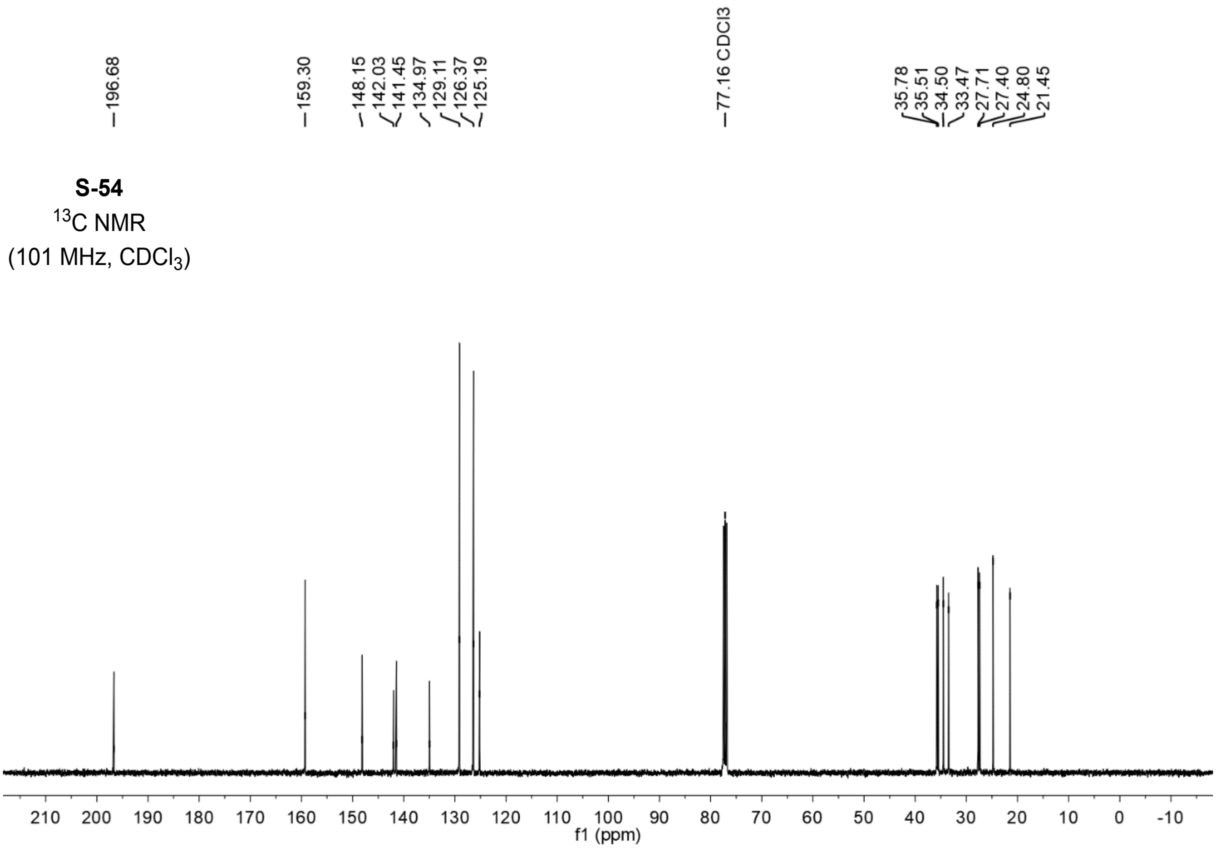

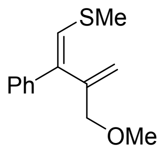

S-55
^{1}H NMR
(400 MHz, $CDCl_3$)

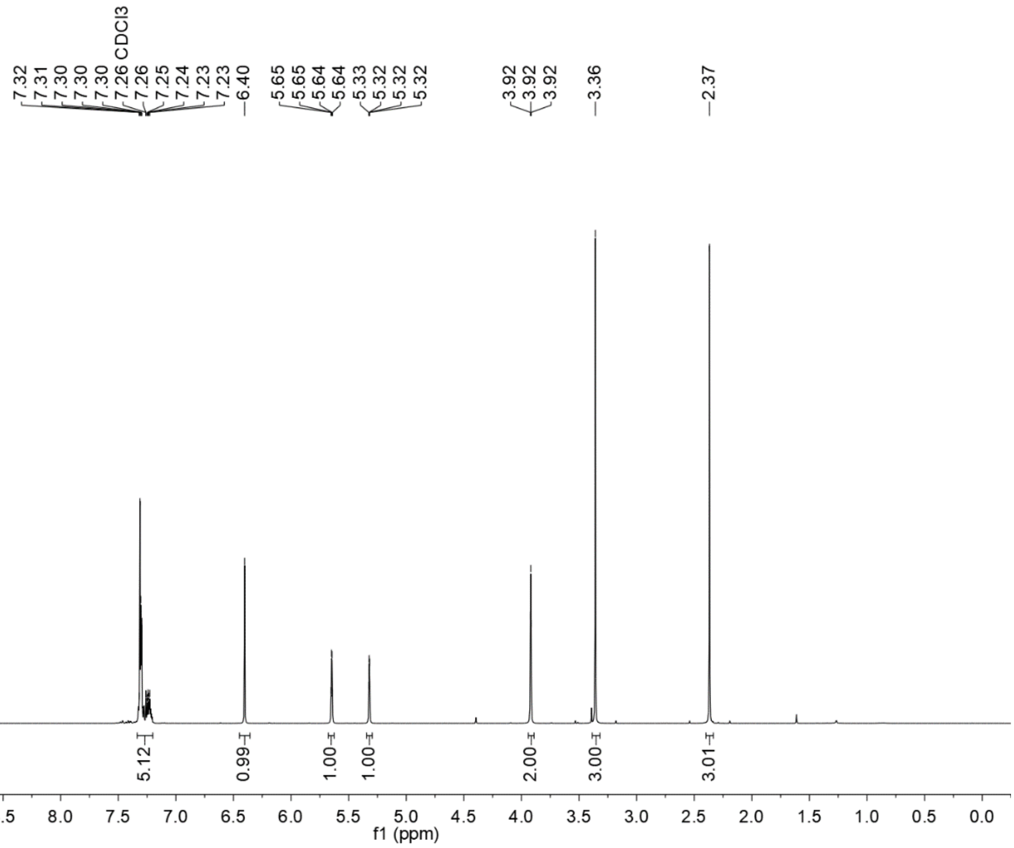

S-55
^{13}C NMR
(101 MHz, $CDCl_3$)

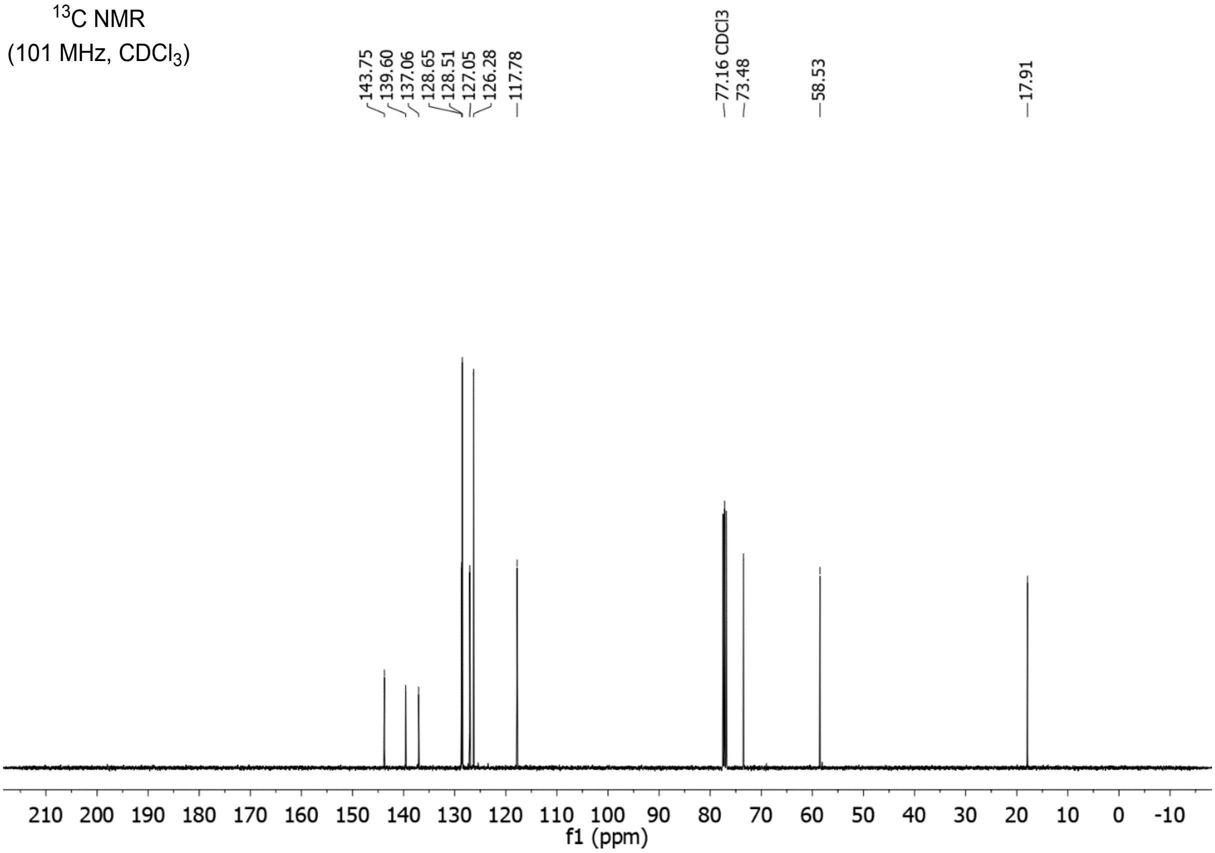

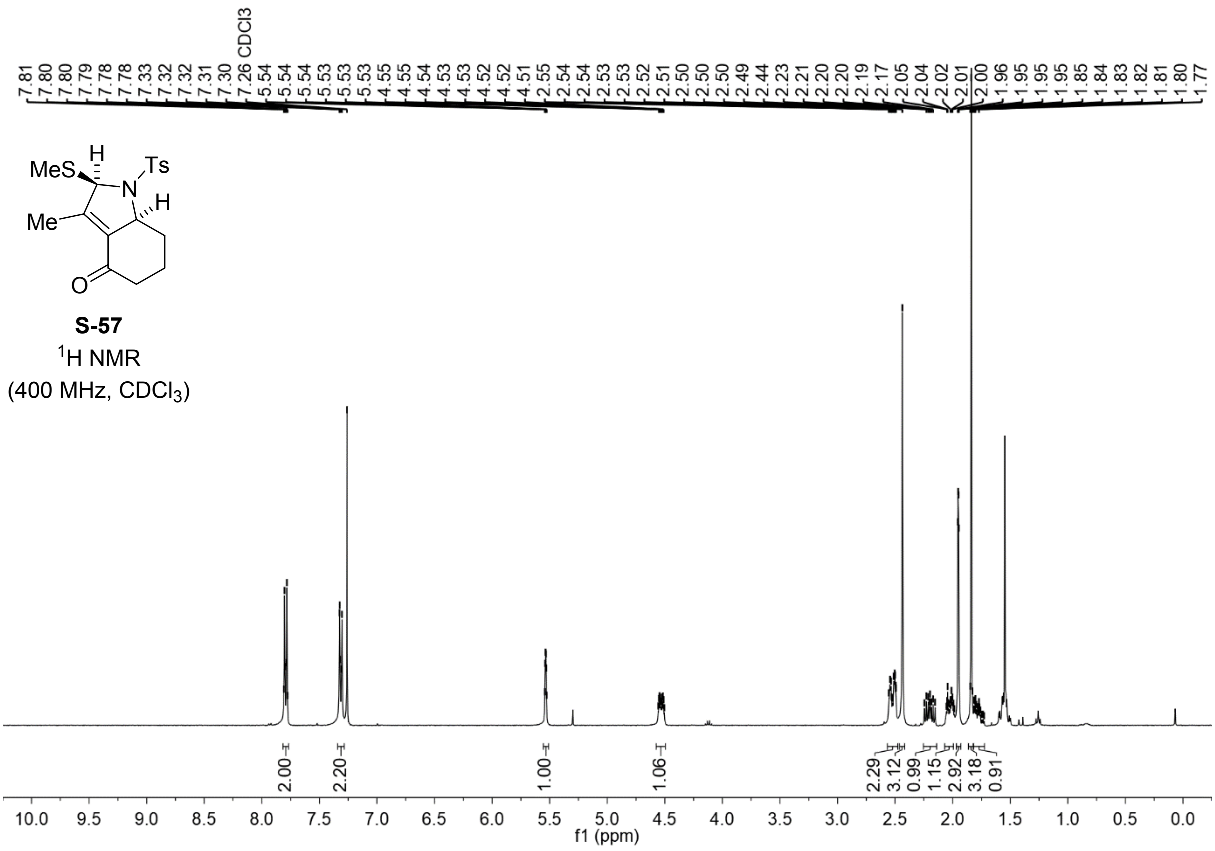

S-57
^{1}H NMR
(400 MHz, $CDCl_3$)

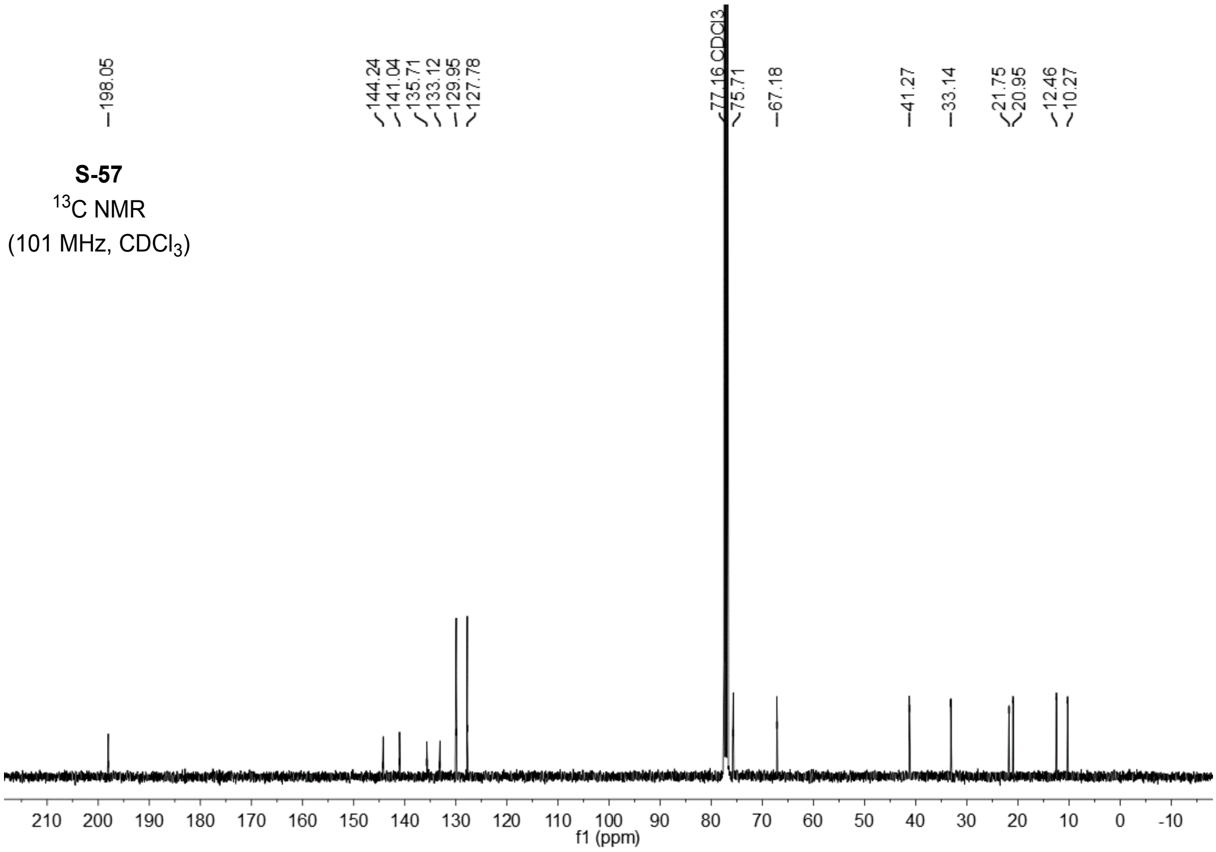

S-57
^{13}C NMR
(101 MHz, $CDCl_3$)

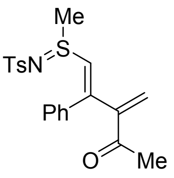

S-59
^{1}H NMR
(400 MHz, $CDCl_3$)

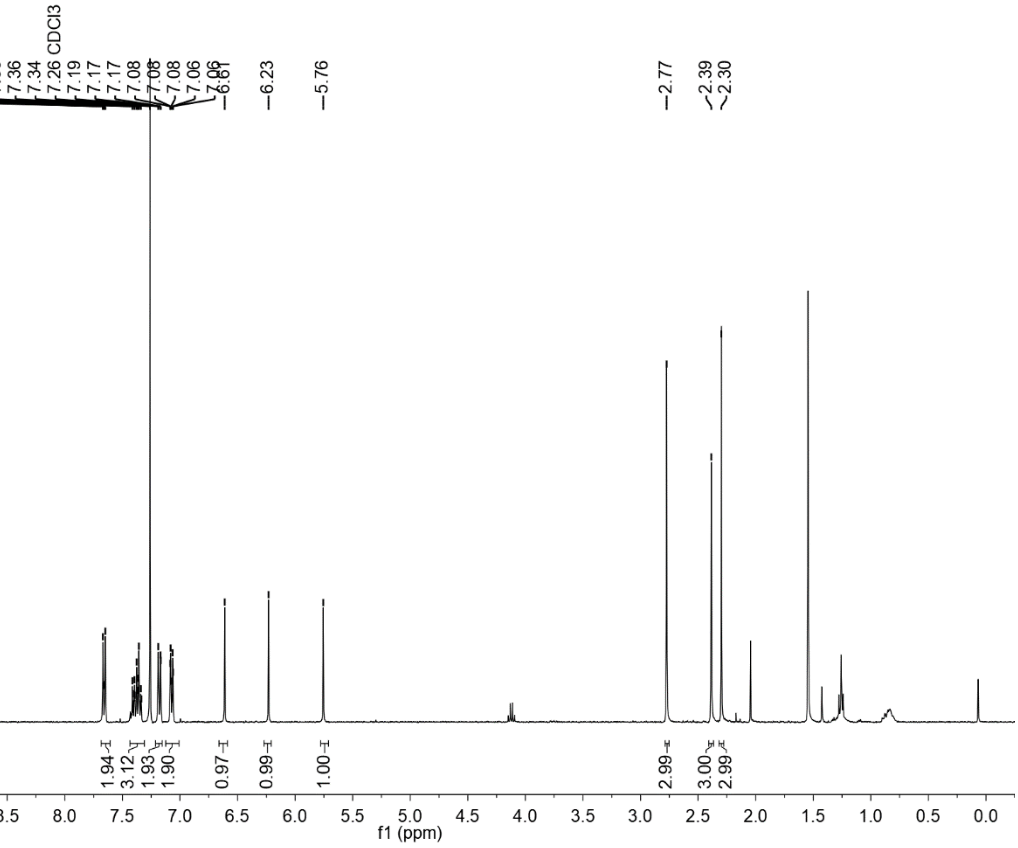

S-59
^{13}C NMR
(101 MHz, $CDCl_3$)

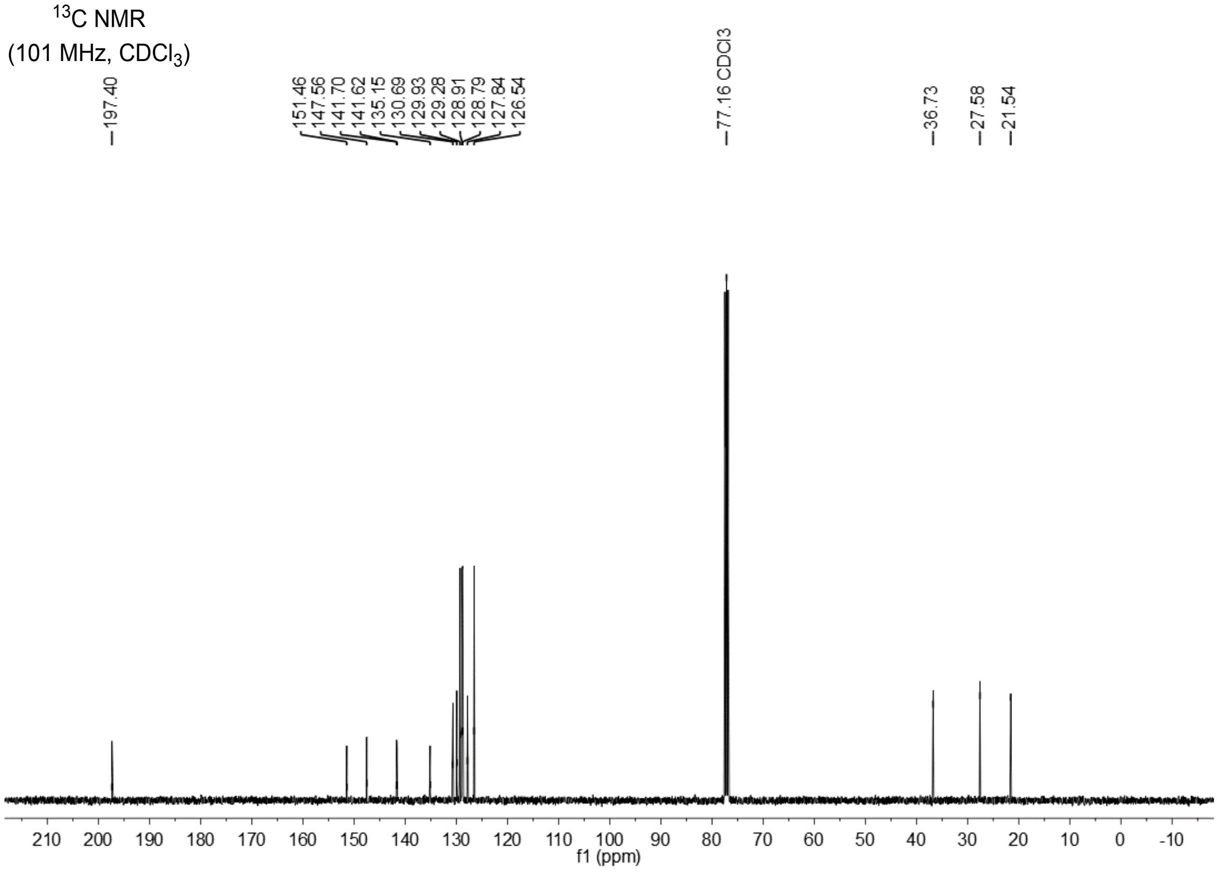

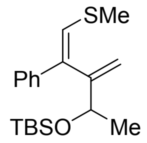

S-60
^{1}H NMR
(400 MHz, $CDCl_3$)

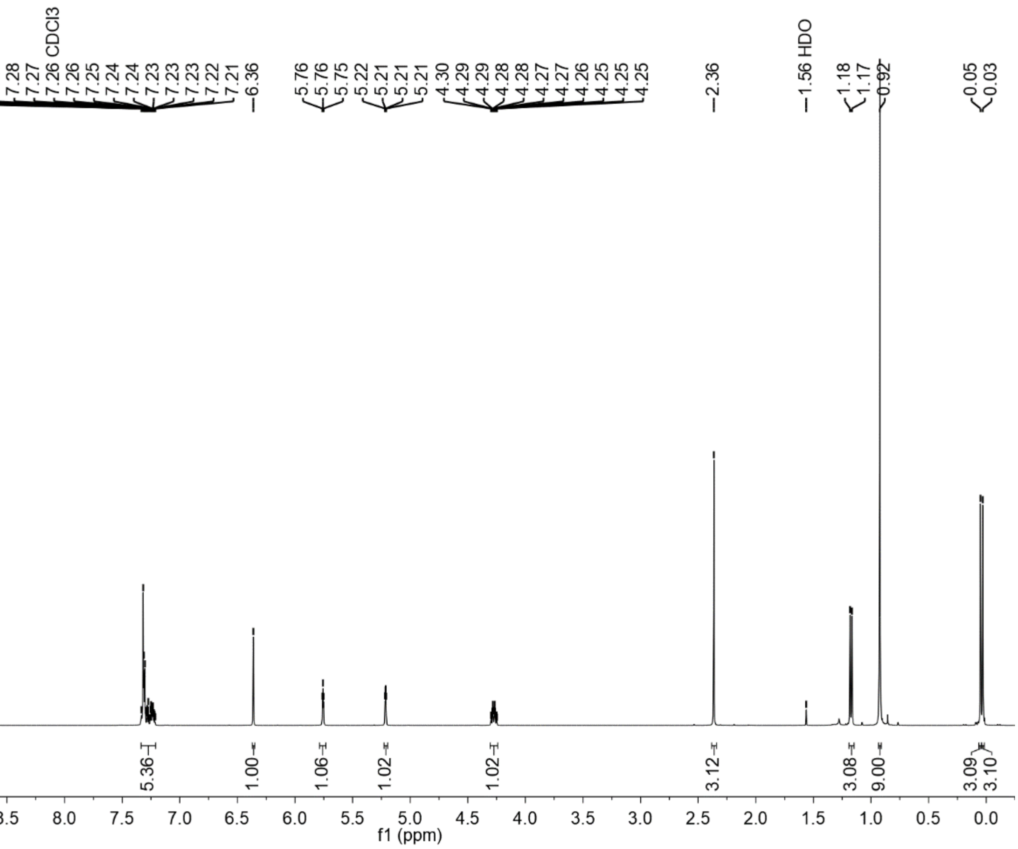

S-60
^{13}C NMR
(101 MHz, $CDCl_3$)

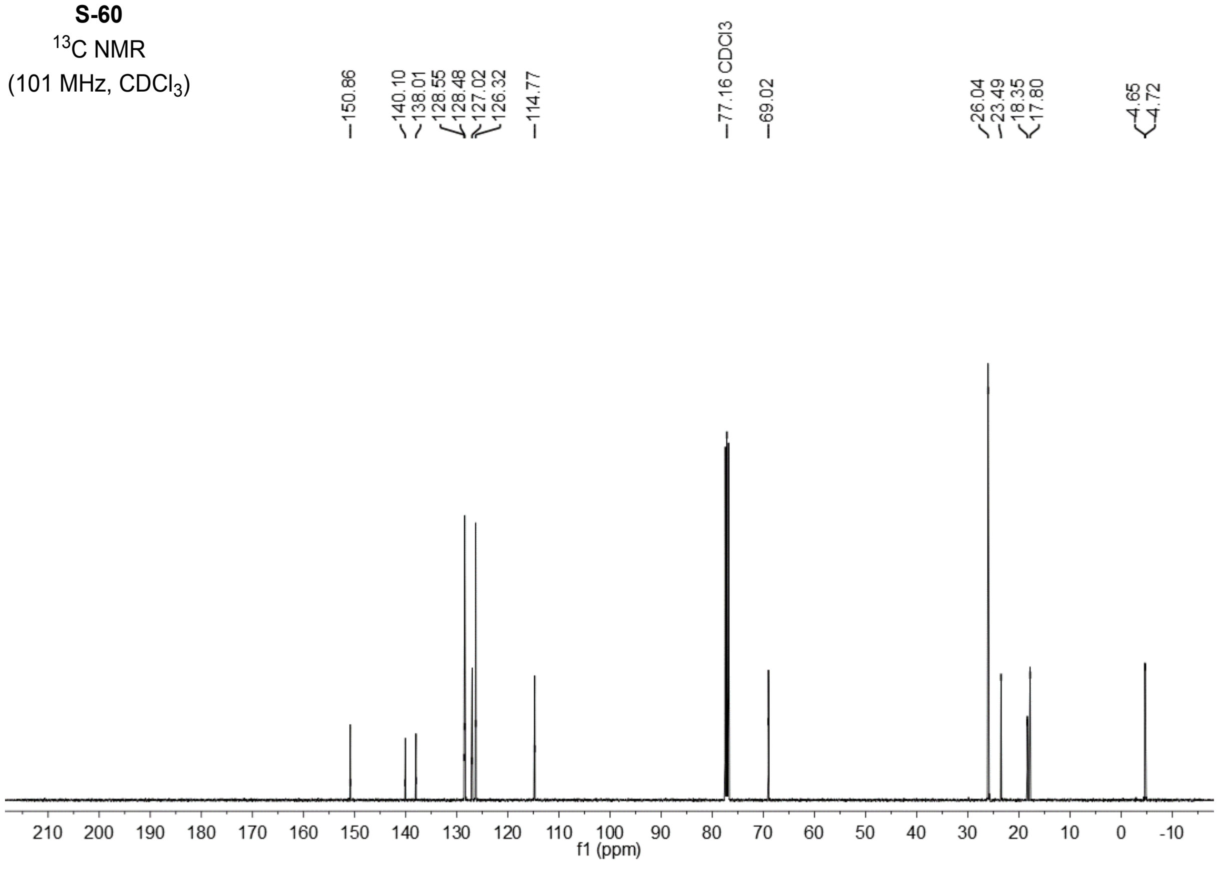

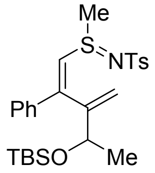

S-61
^1H NMR
(400 MHz, $CDCl_3$)

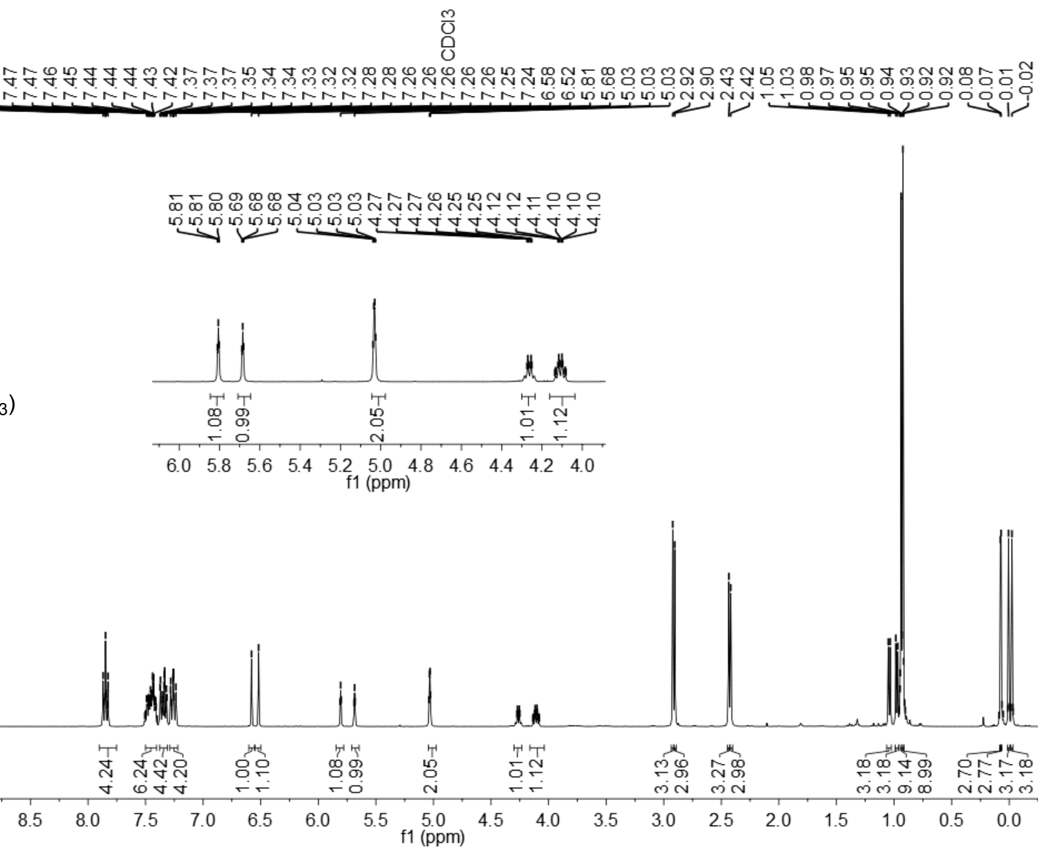

S-61
^{13}C NMR
(101 MHz, $CDCl_3$)

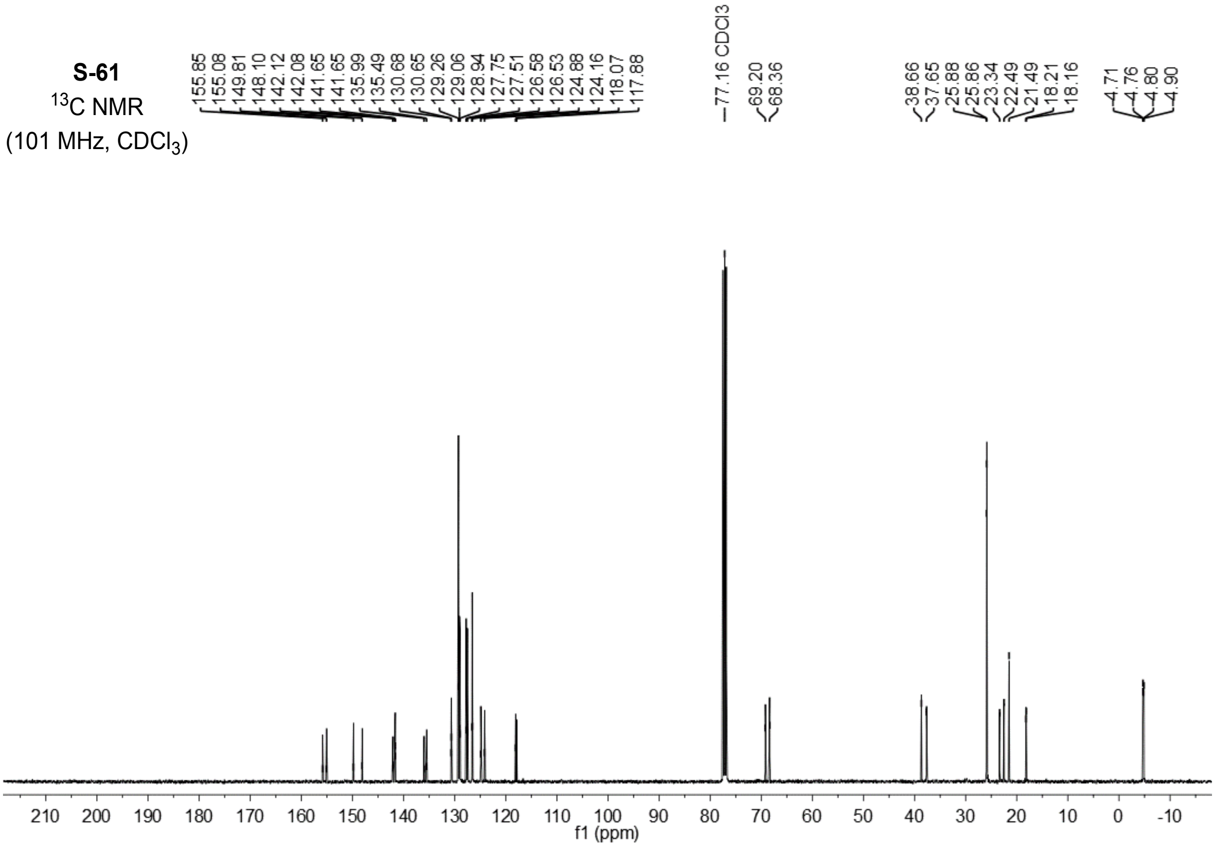

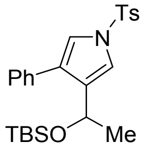

S-62
^{1}H NMR
(400 MHz, $CDCl_3$)

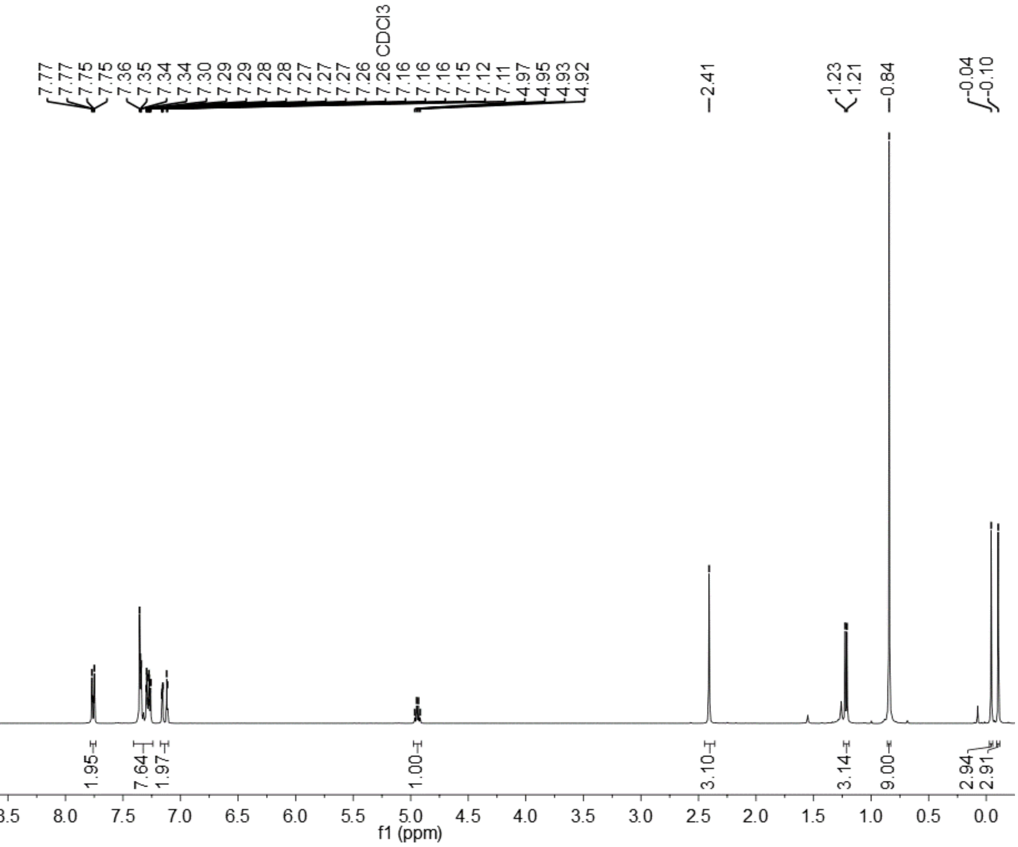

S-62
^{13}C NMR
(101 MHz, $CDCl_3$)

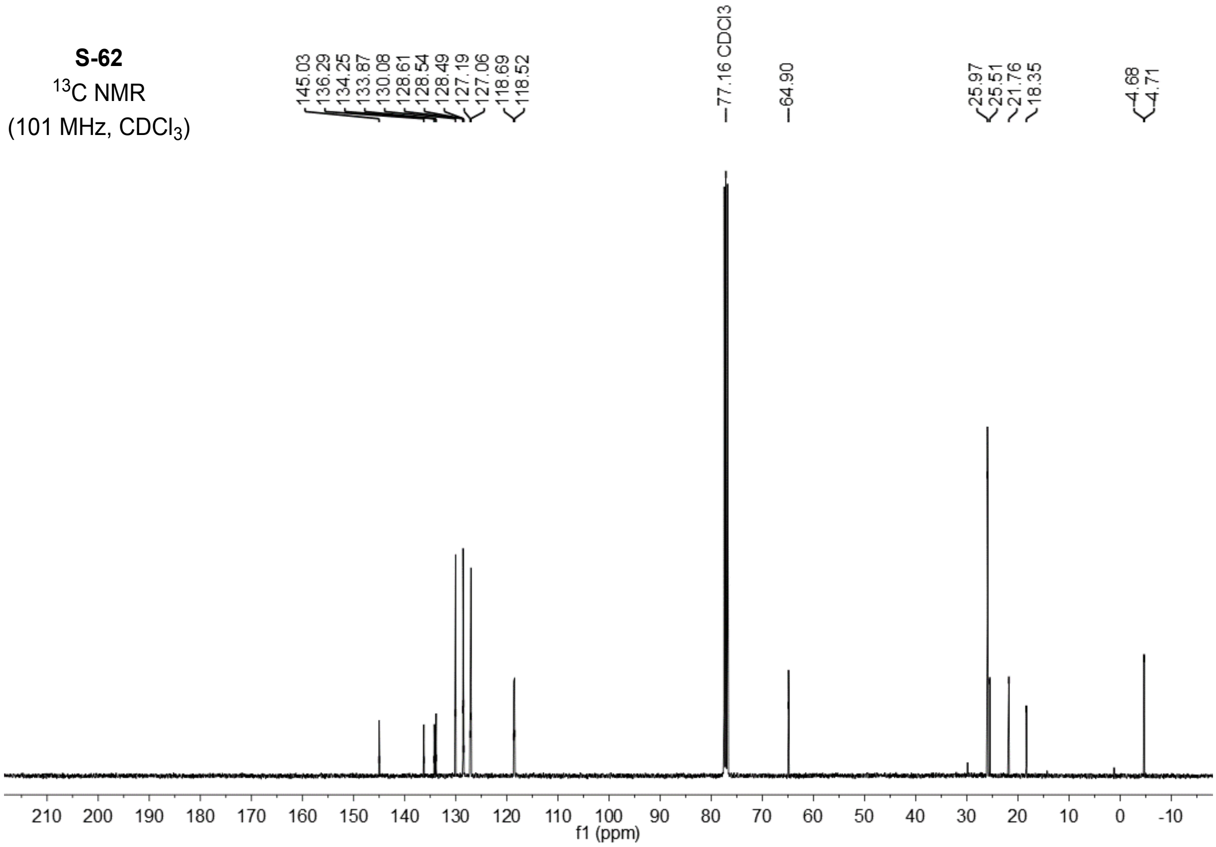

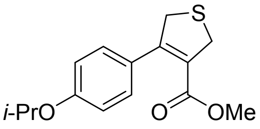

S-75
^{1}H NMR
(400 MHz, $CDCl_3$)

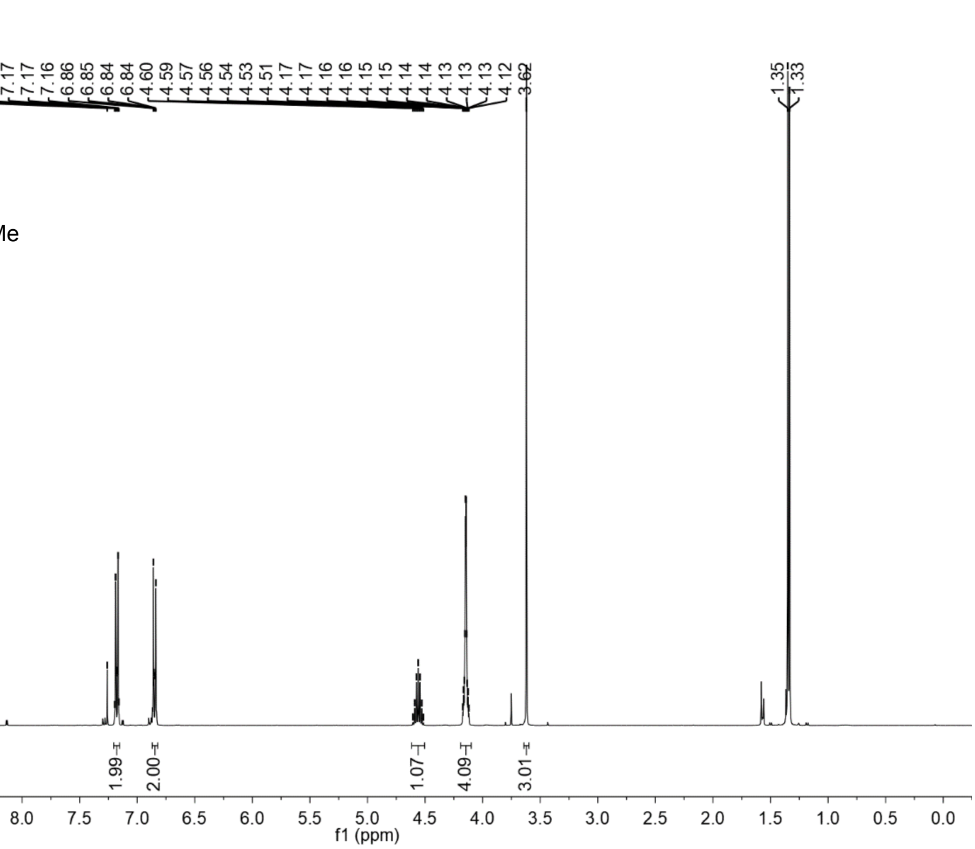

S-75
^{13}C NMR
(101 MHz, $CDCl_3$)

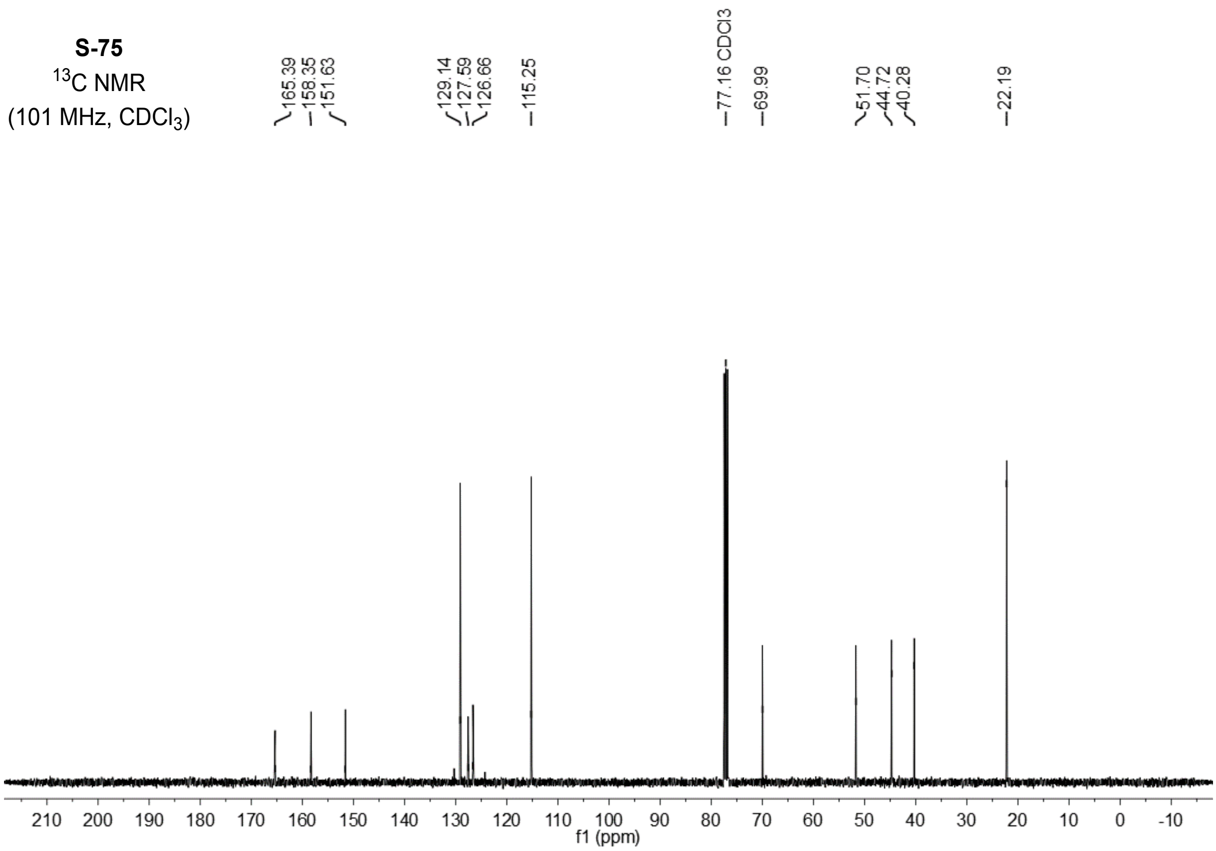

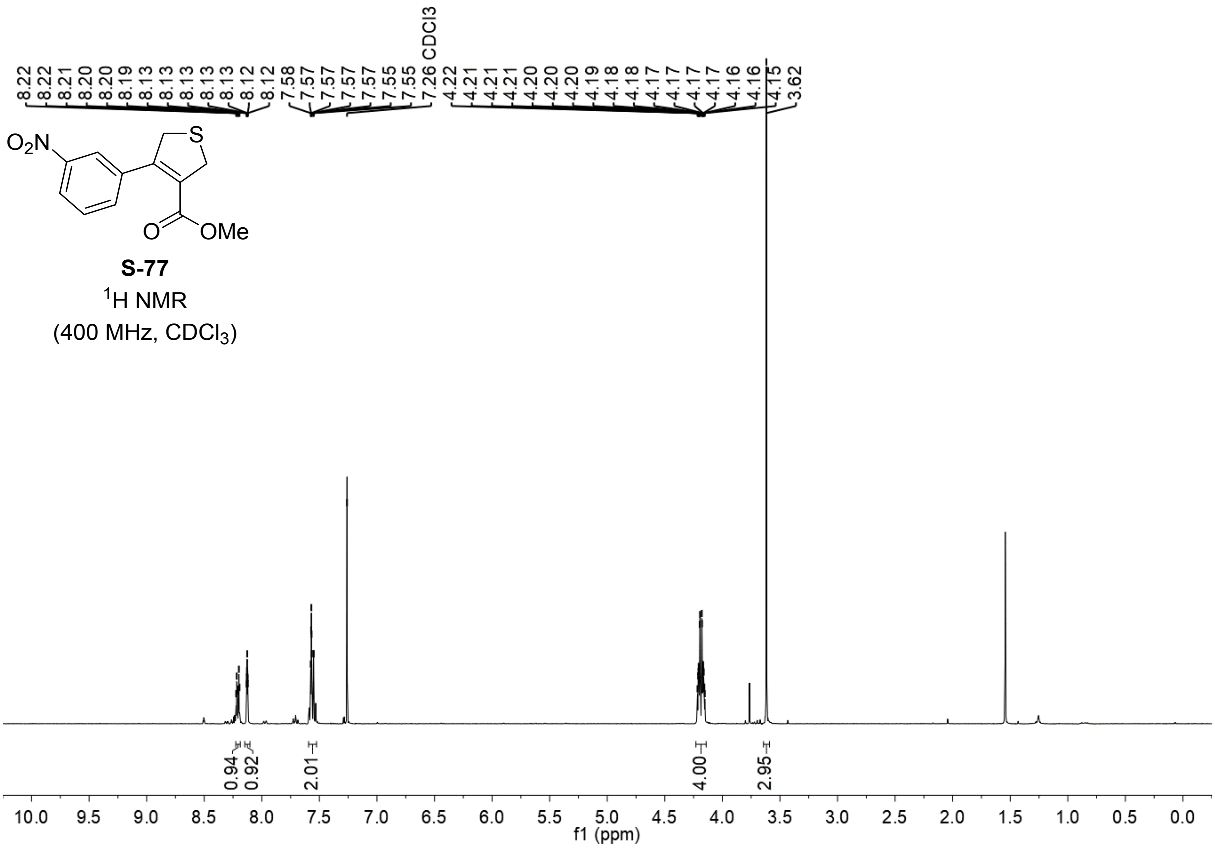

S-77
^{1}H NMR
(400 MHz, $CDCl_3$)

S-77
^{13}C NMR
(101 MHz, $CDCl_3$)

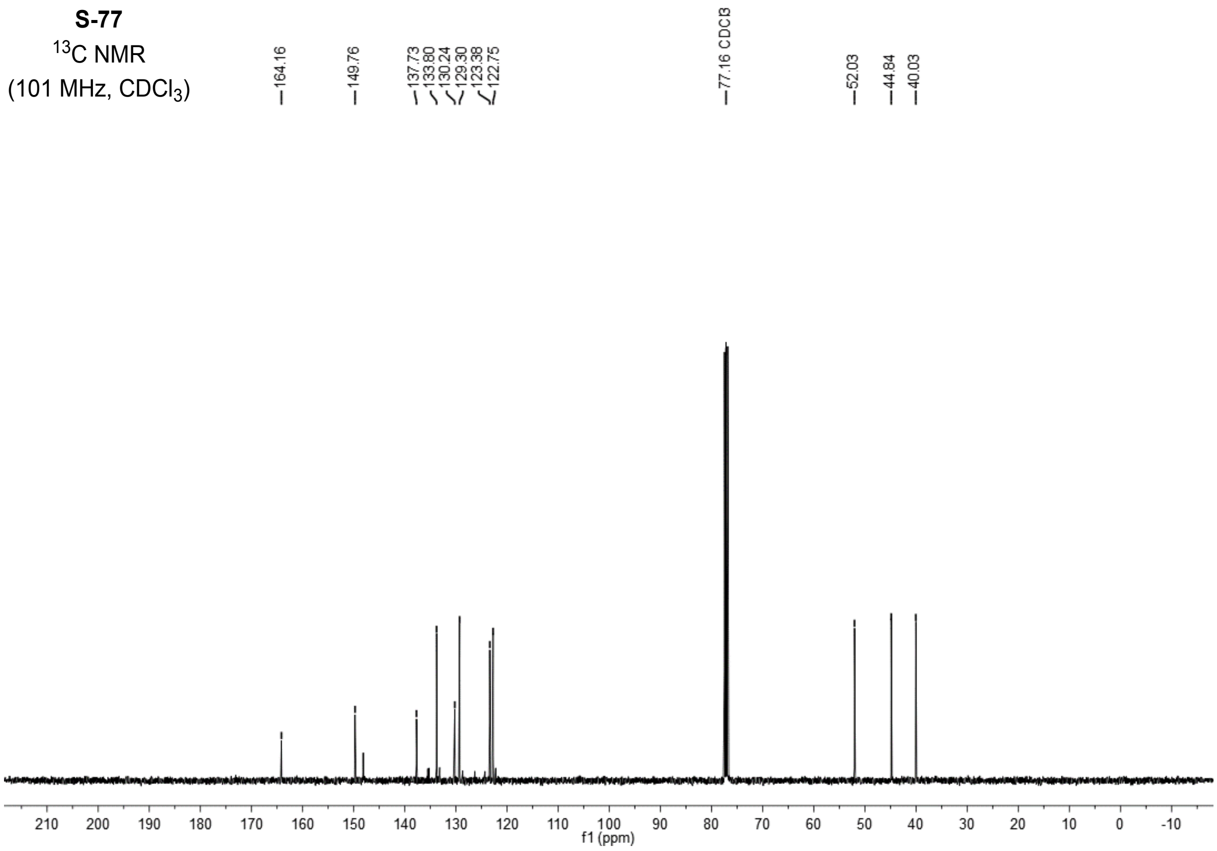

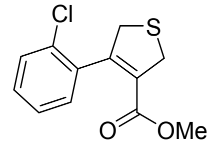

S-79

^{1}H NMR

(400 MHz, $CDCl_3$)

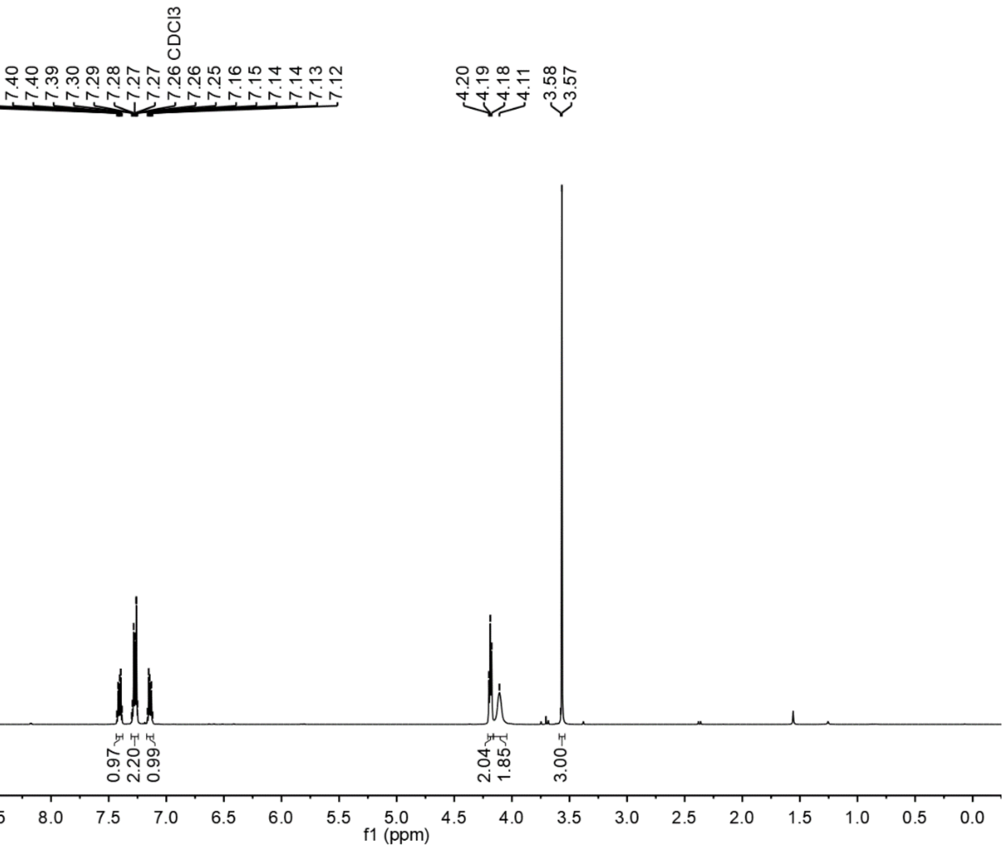

S-79

^{13}C NMR

(101 MHz, $CDCl_3$)

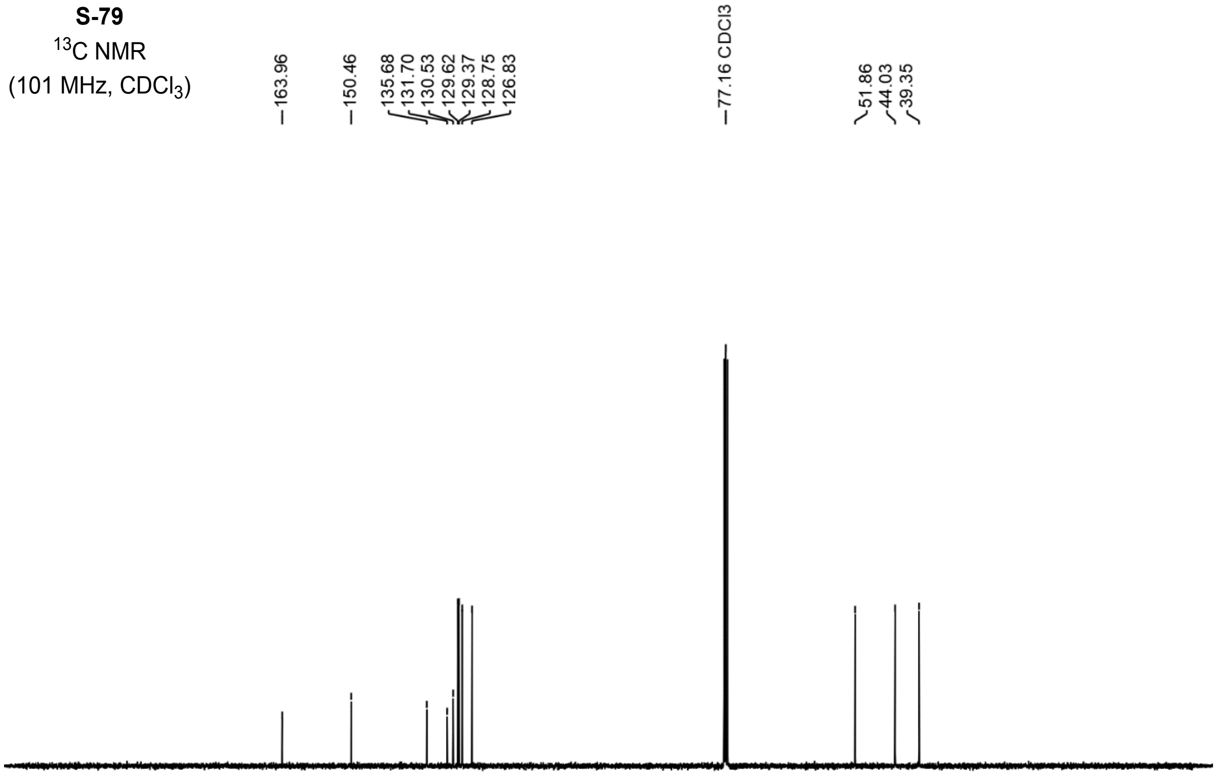

S-81
^{1}H NMR
(400 MHz, $CDCl_3$)

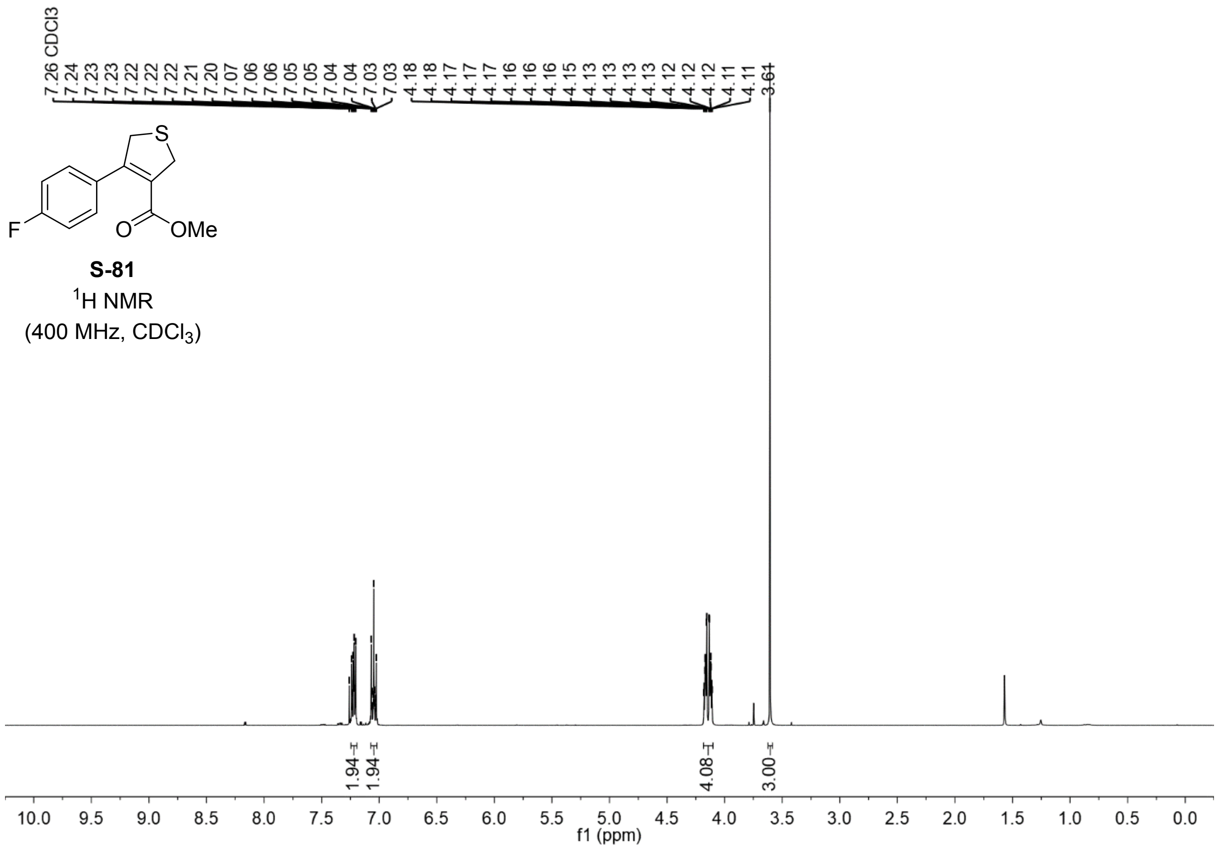

S-81
^{13}C NMR
(101 MHz, $CDCl_3$)

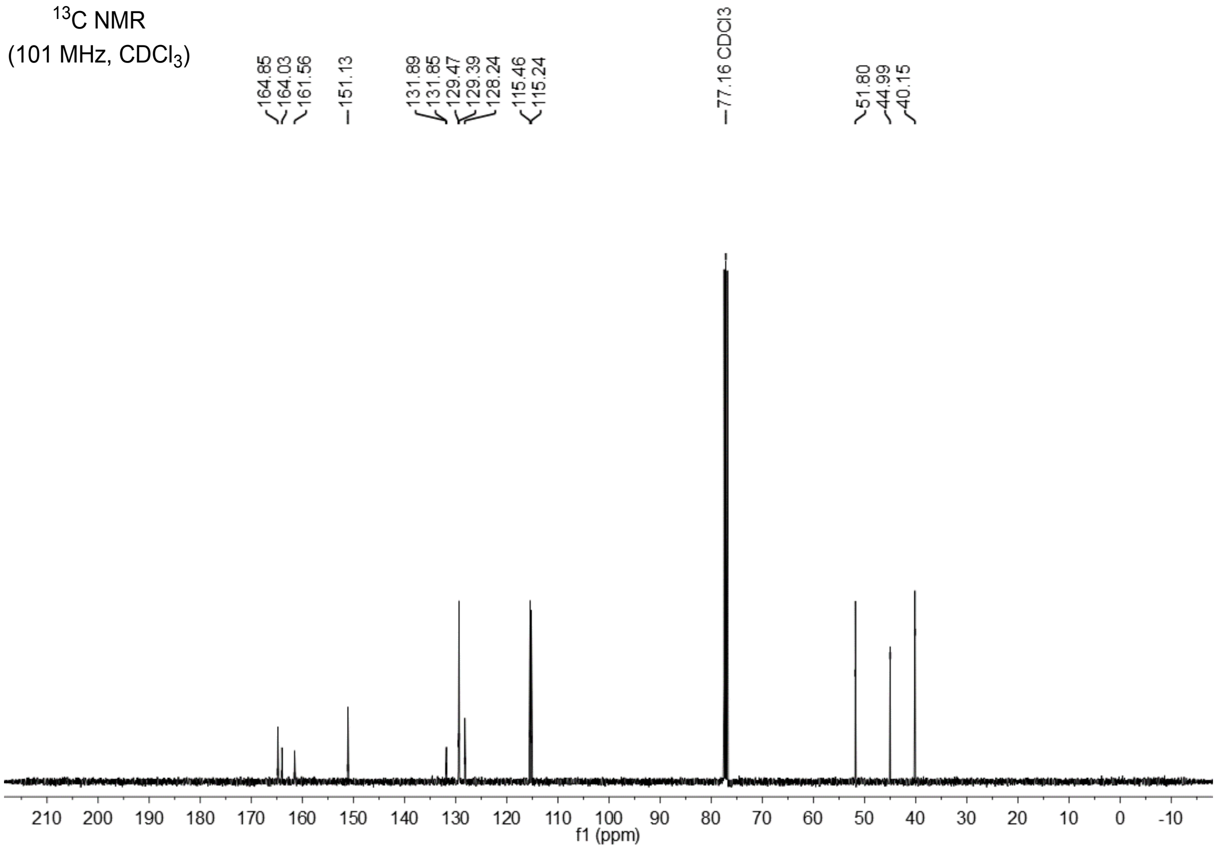

S-81
^{19}F NMR
(376 MHz, $CDCl_3$)

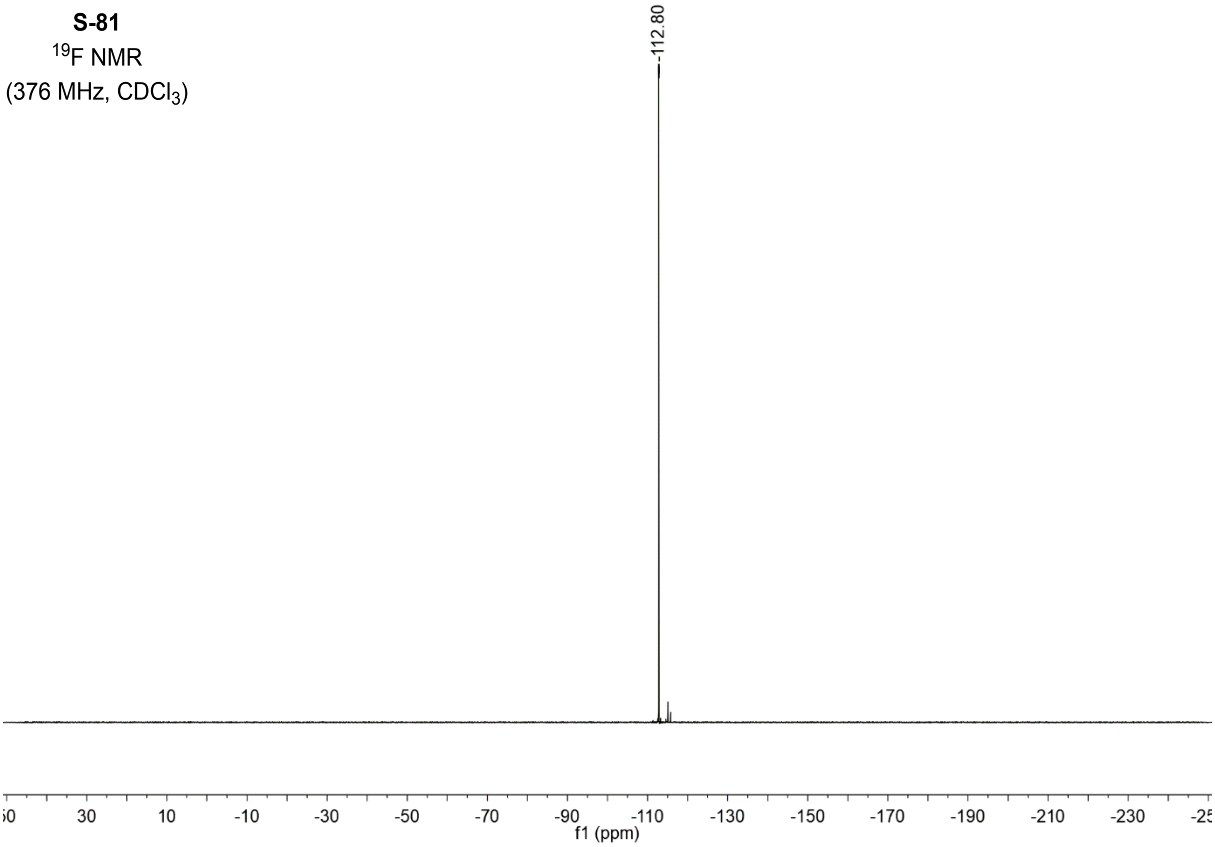

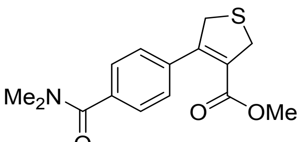

S-83
^{1}H NMR
(400 MHz, $CDCl_3$)

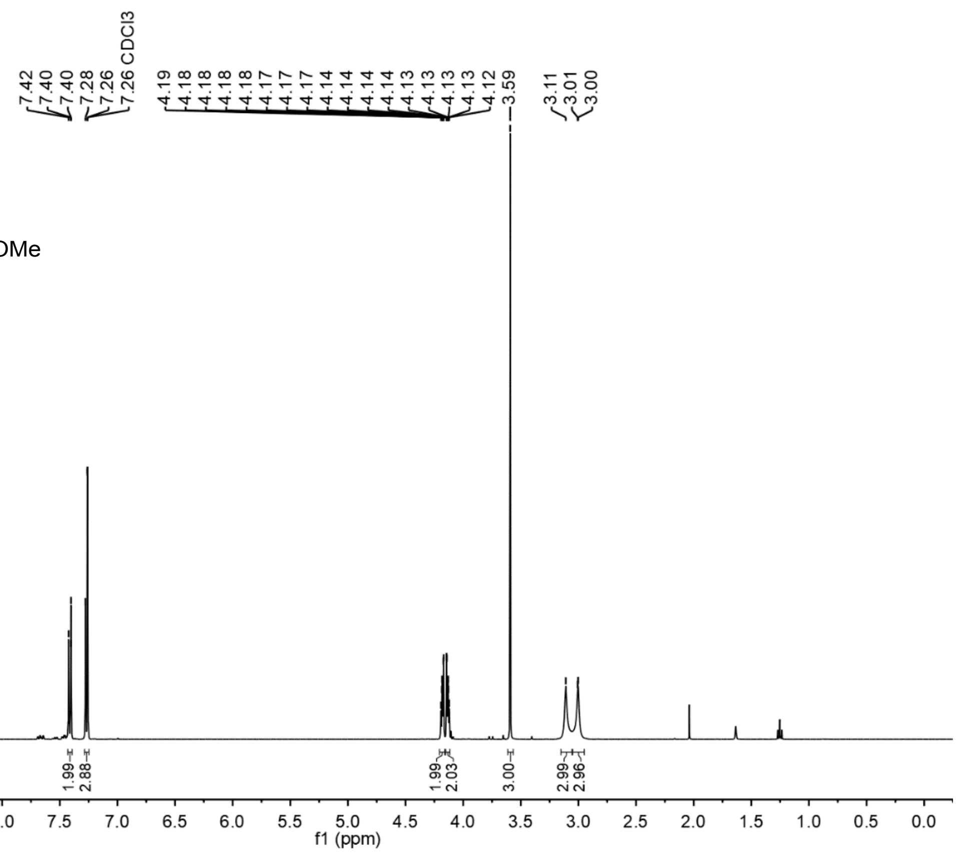

S-83
^{13}C NMR
(101 MHz, $CDCl_3$)

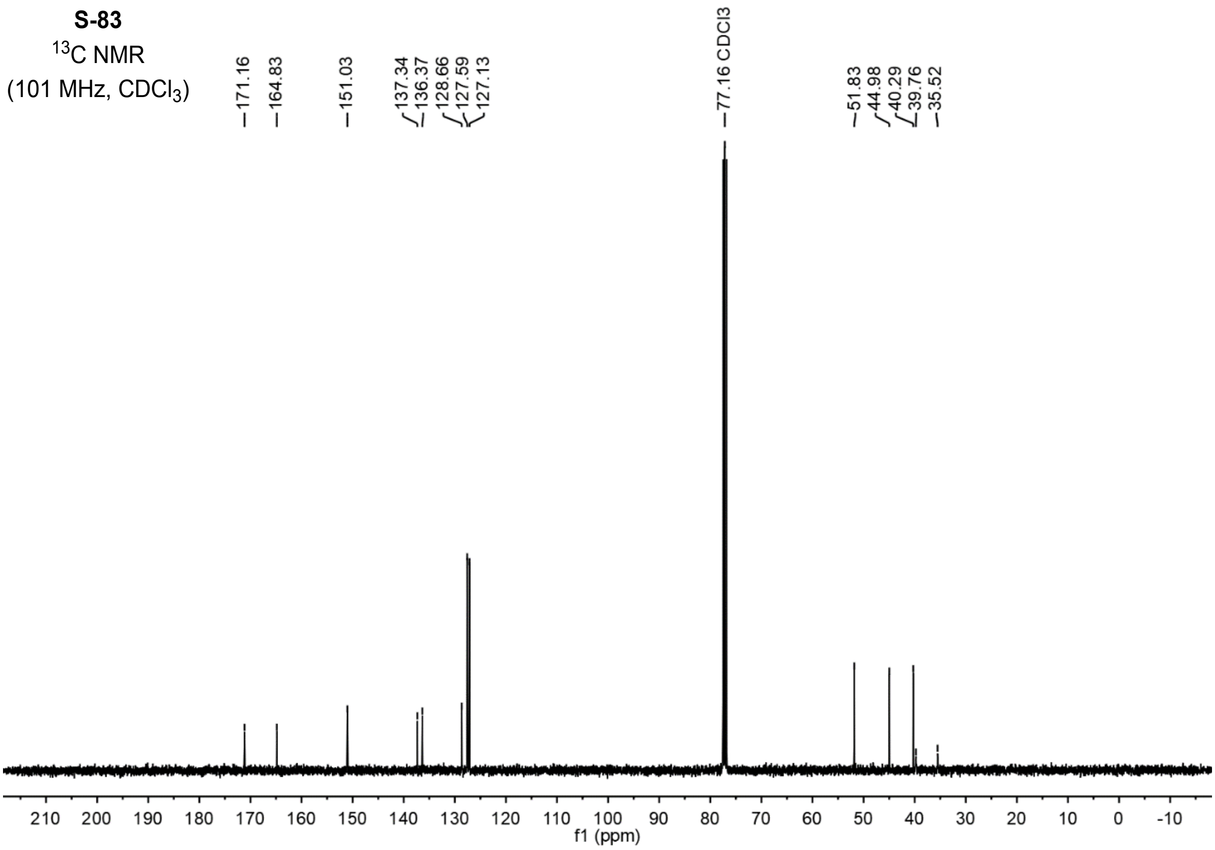

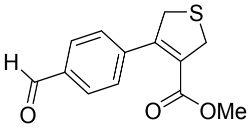

S-85
^{1}H NMR
(400 MHz, $CDCl_3$)

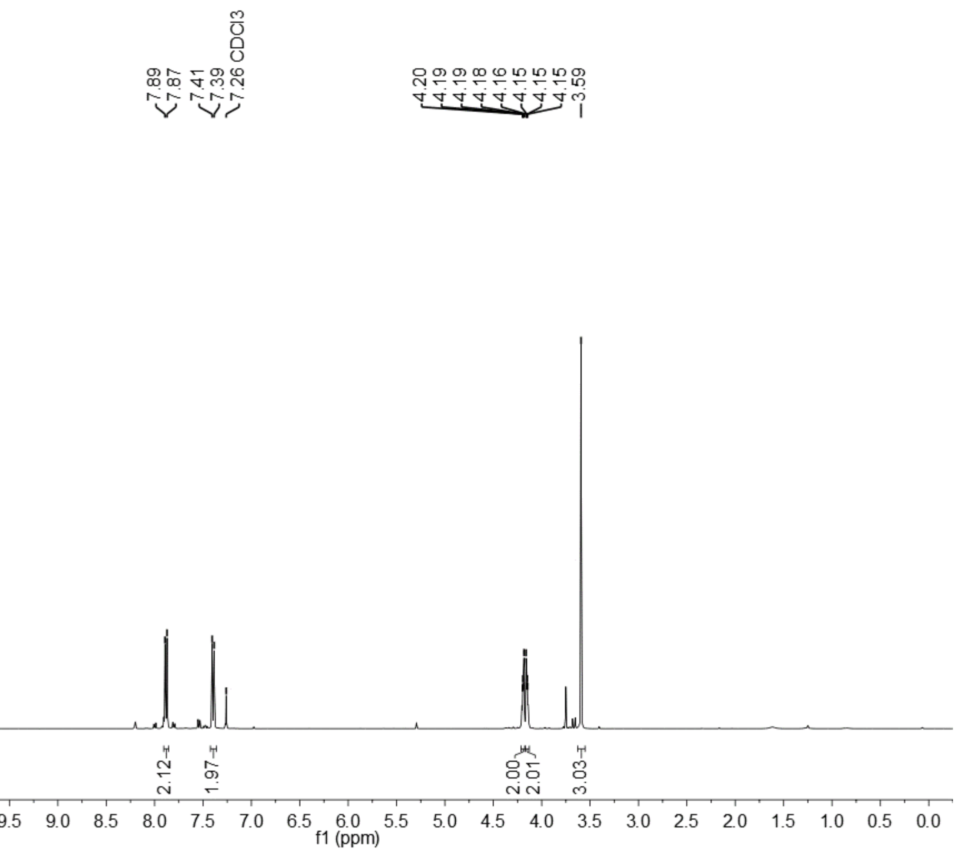

S-85
^{13}C NMR
(101 MHz, $CDCl_3$)

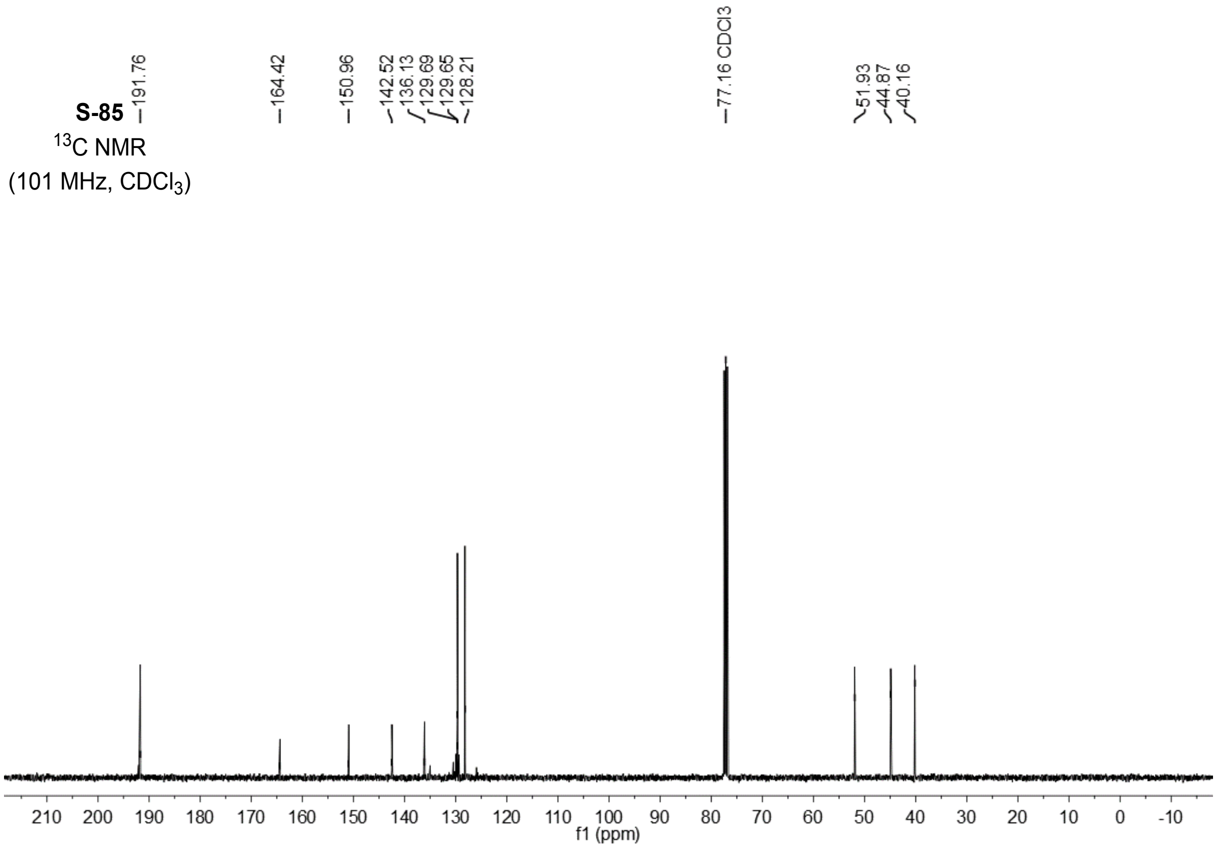

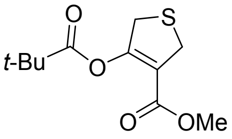

S-86
^{1}H NMR
(400 MHz, $CDCl_3$)

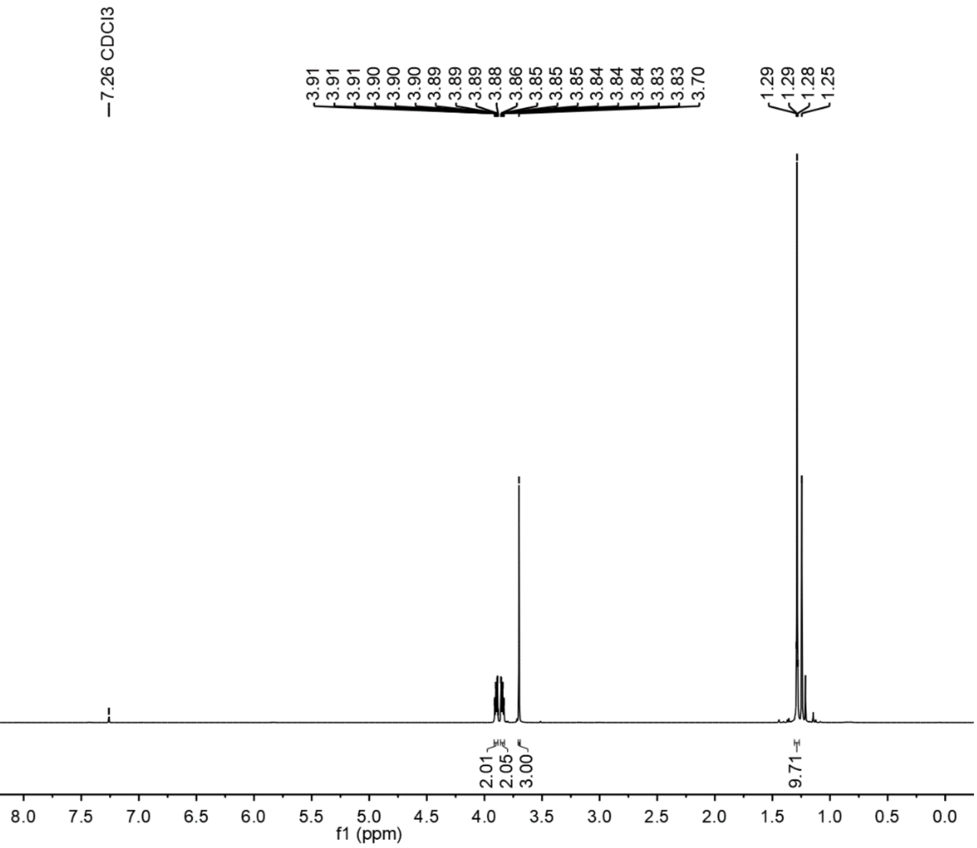

S-86
^{13}C NMR
(101 MHz, $CDCl_3$)

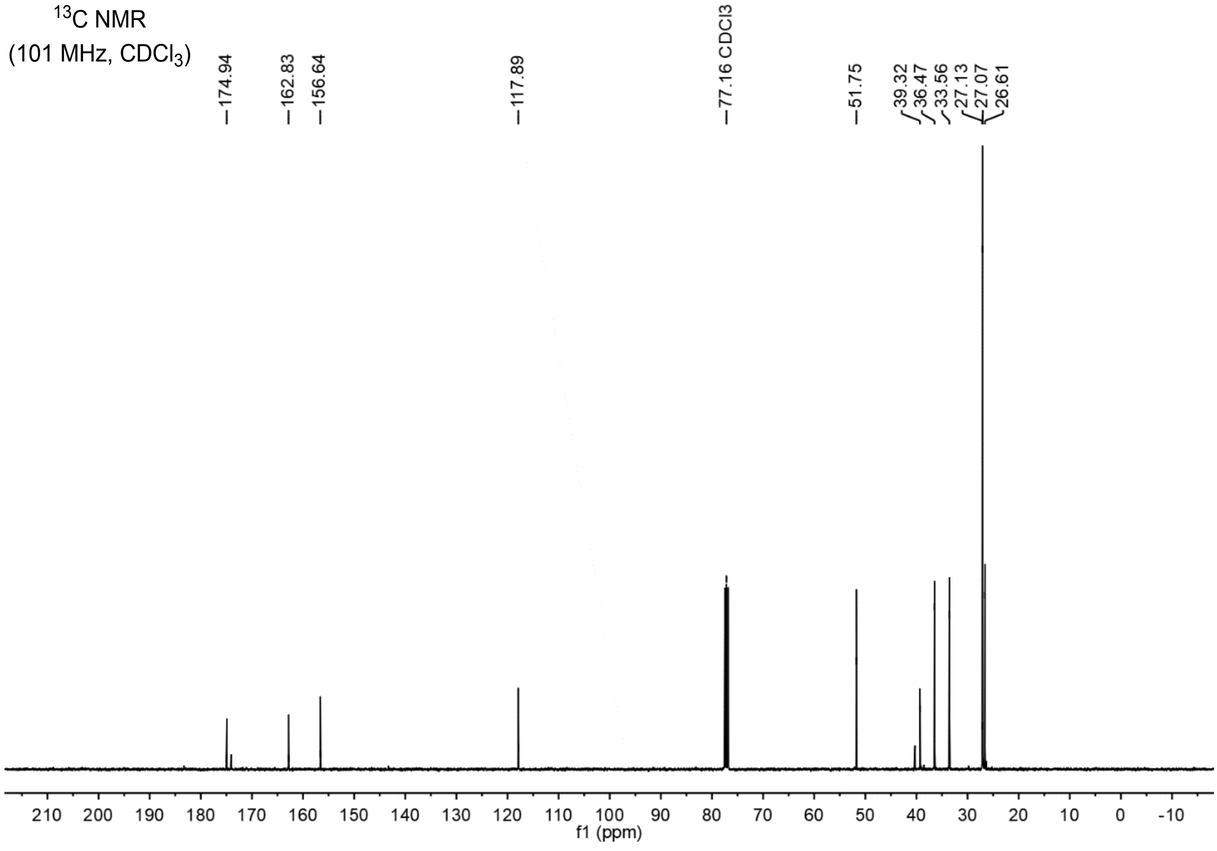

S-90

^{1}H NMR

(400 MHz, $CDCl_3$)

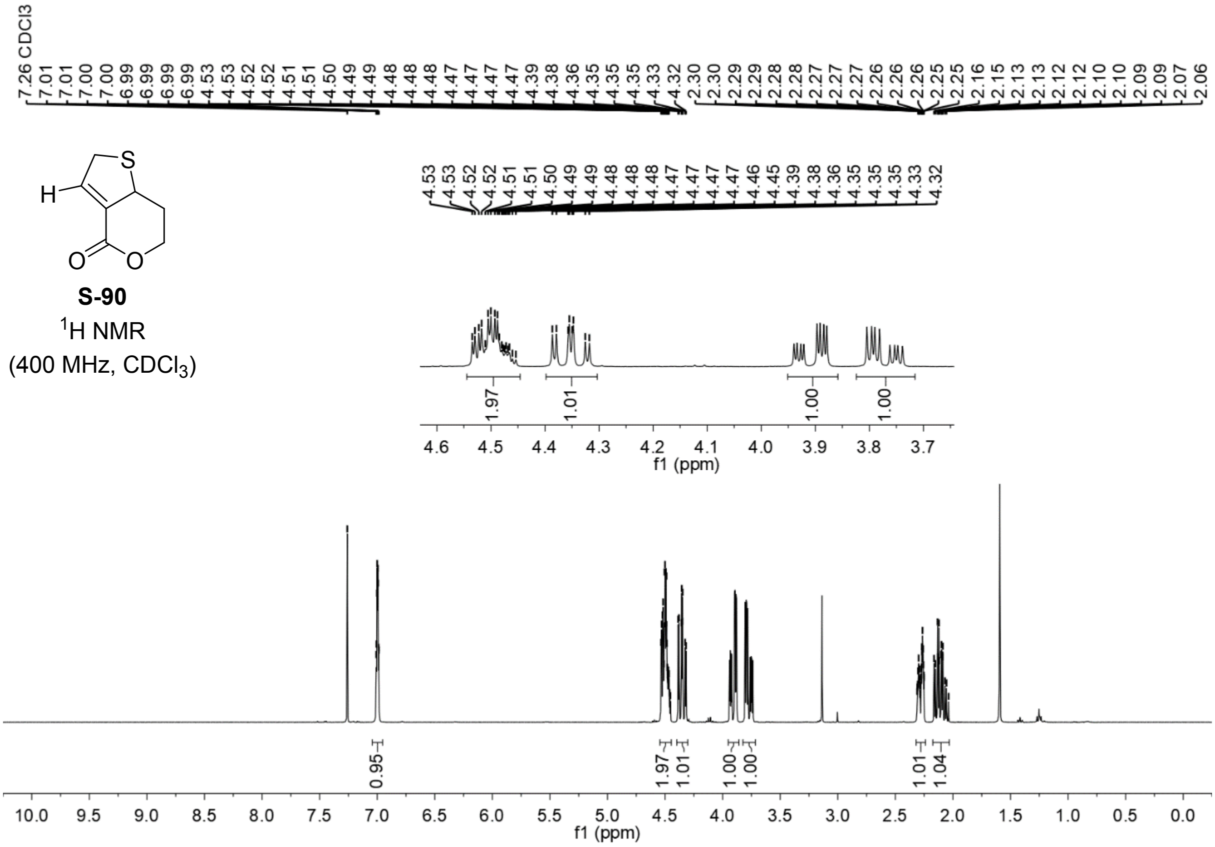

S-90

^{13}C NMR

(101 MHz, $CDCl_3$)

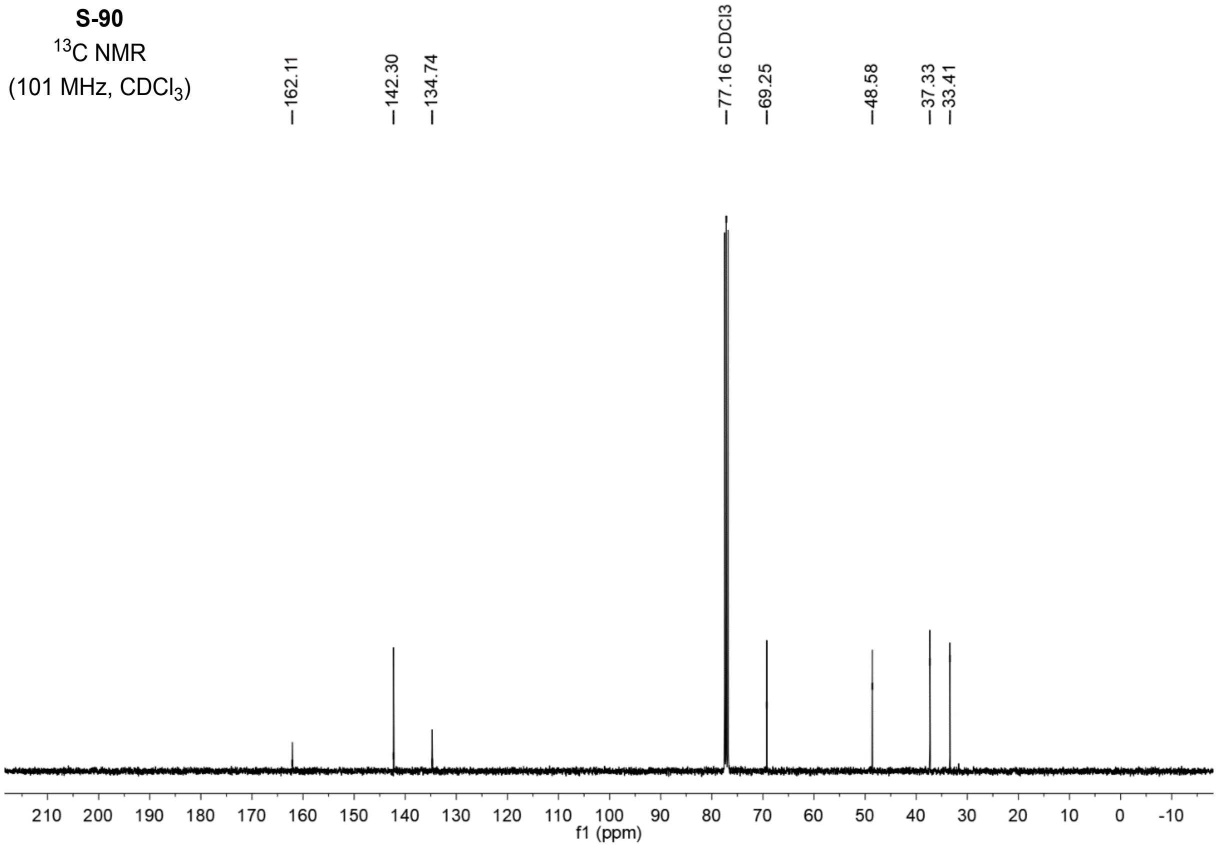

S-93
^{1}H NMR
(400 MHz, $CDCl_3$)

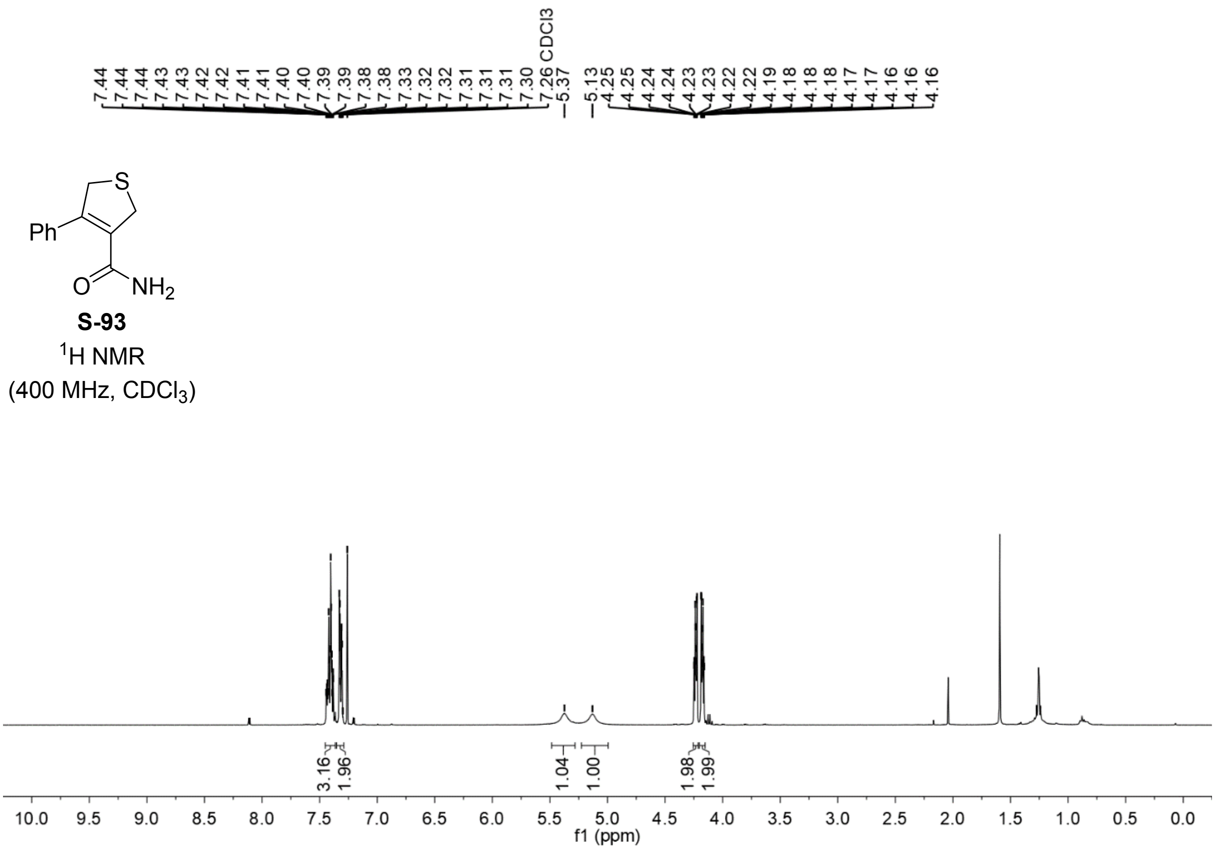

S-93
^{13}C NMR
(101 MHz, $CDCl_3$)

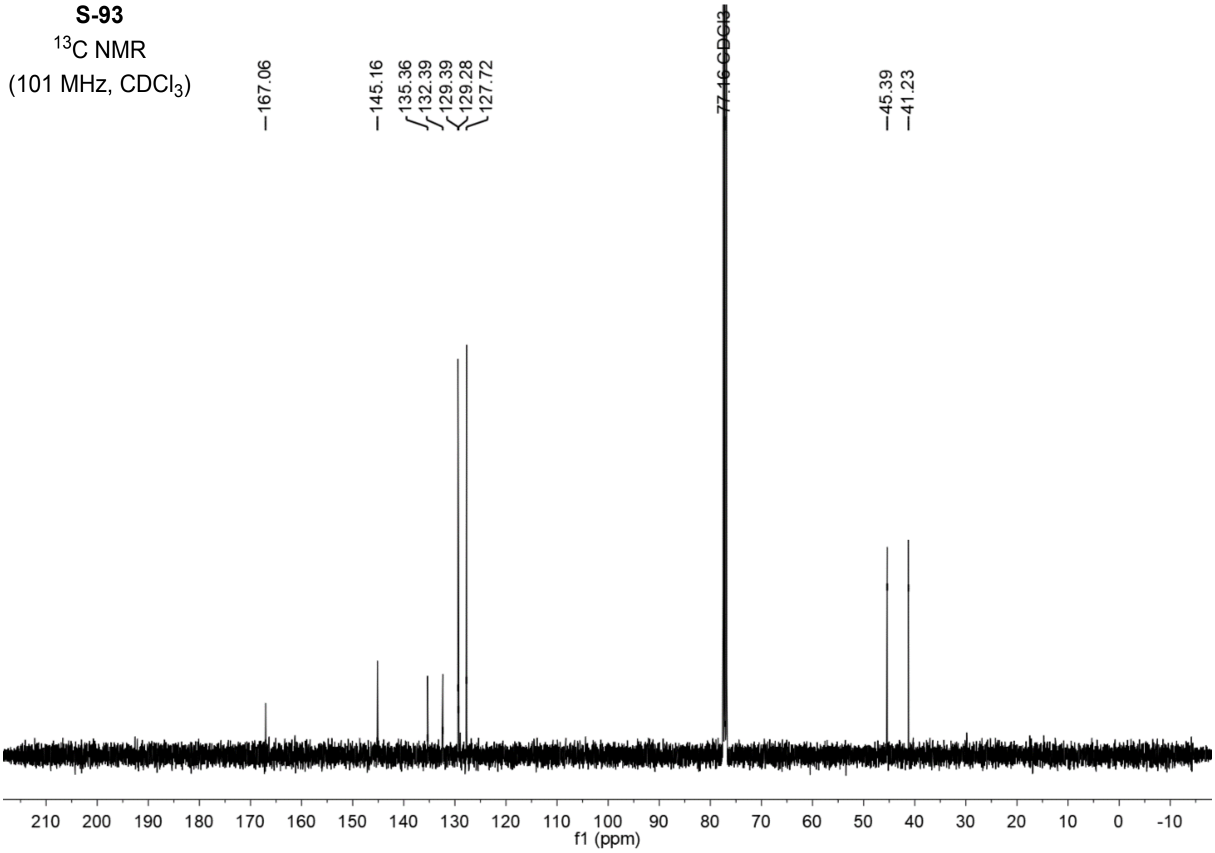

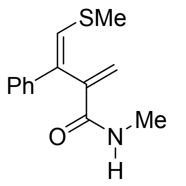

S-94
^{1}H NMR
(400 MHz, $CDCl_3$)

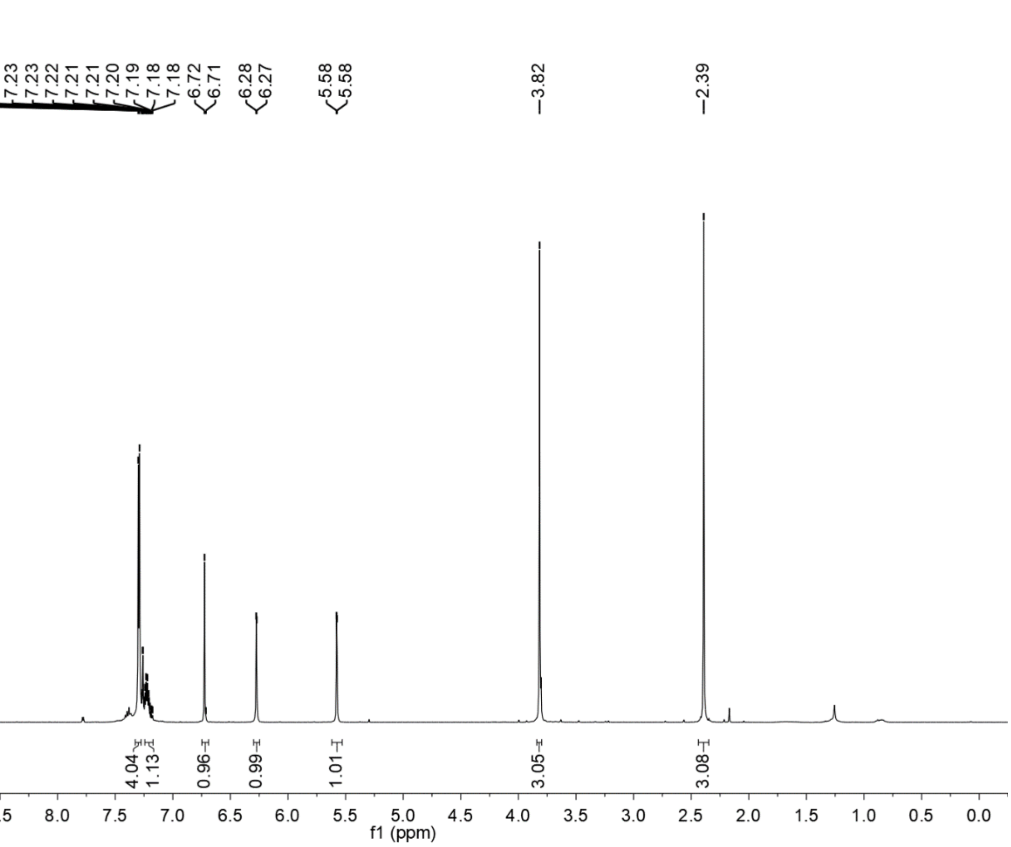

S-94
^{13}C NMR
(101 MHz, $CDCl_3$)

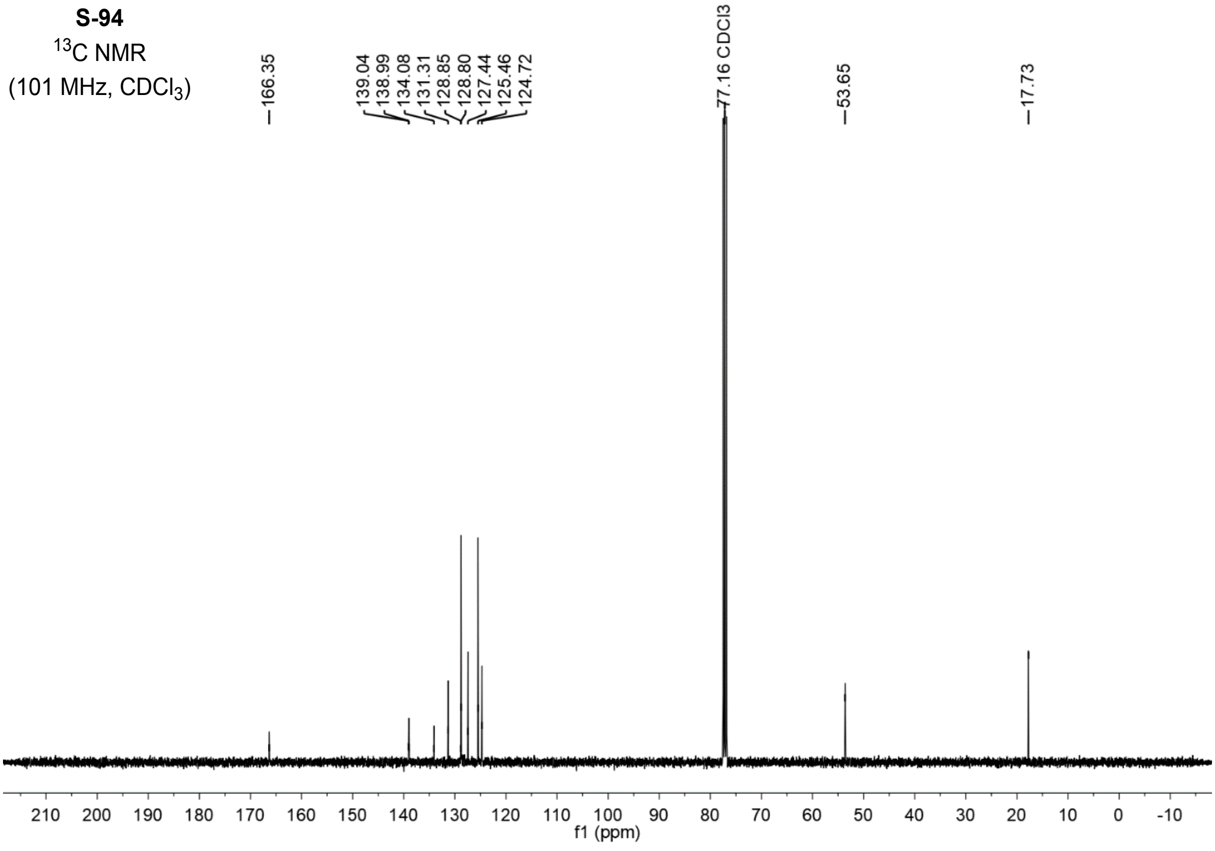

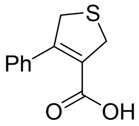

S-95
^{1}H NMR
(400 MHz, $CDCl_3$)

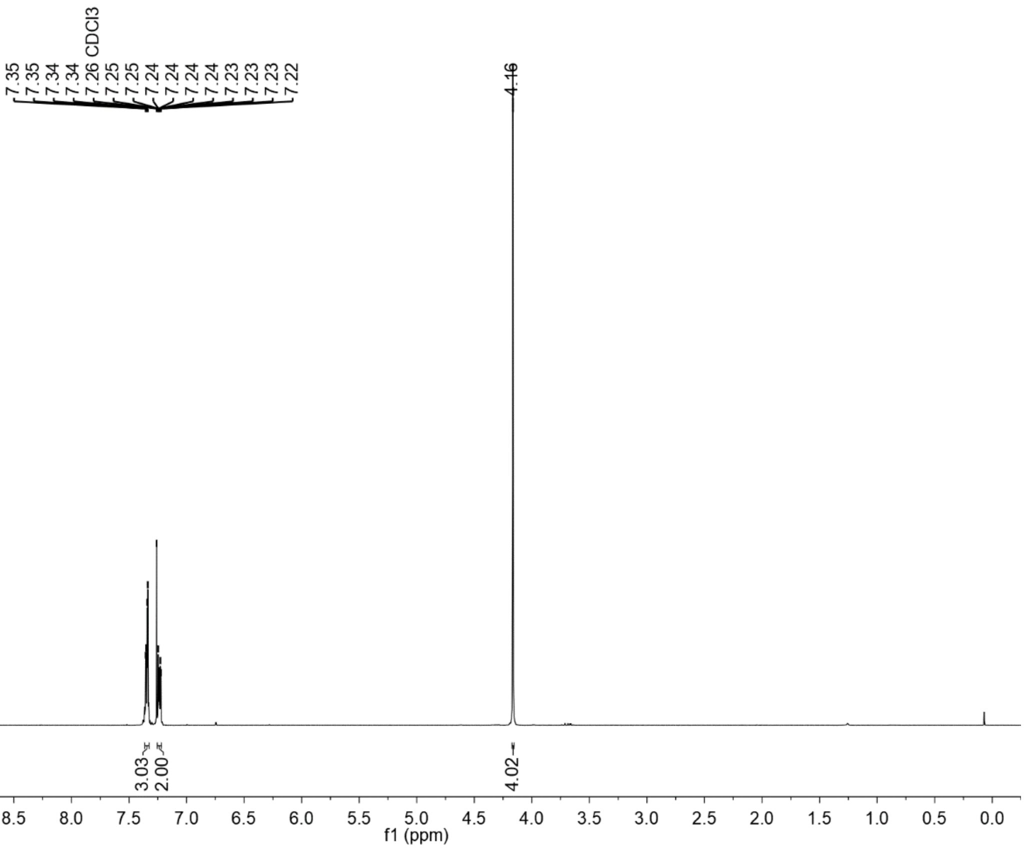

S-95
^{13}C NMR
(101 MHz, $CDCl_3$)

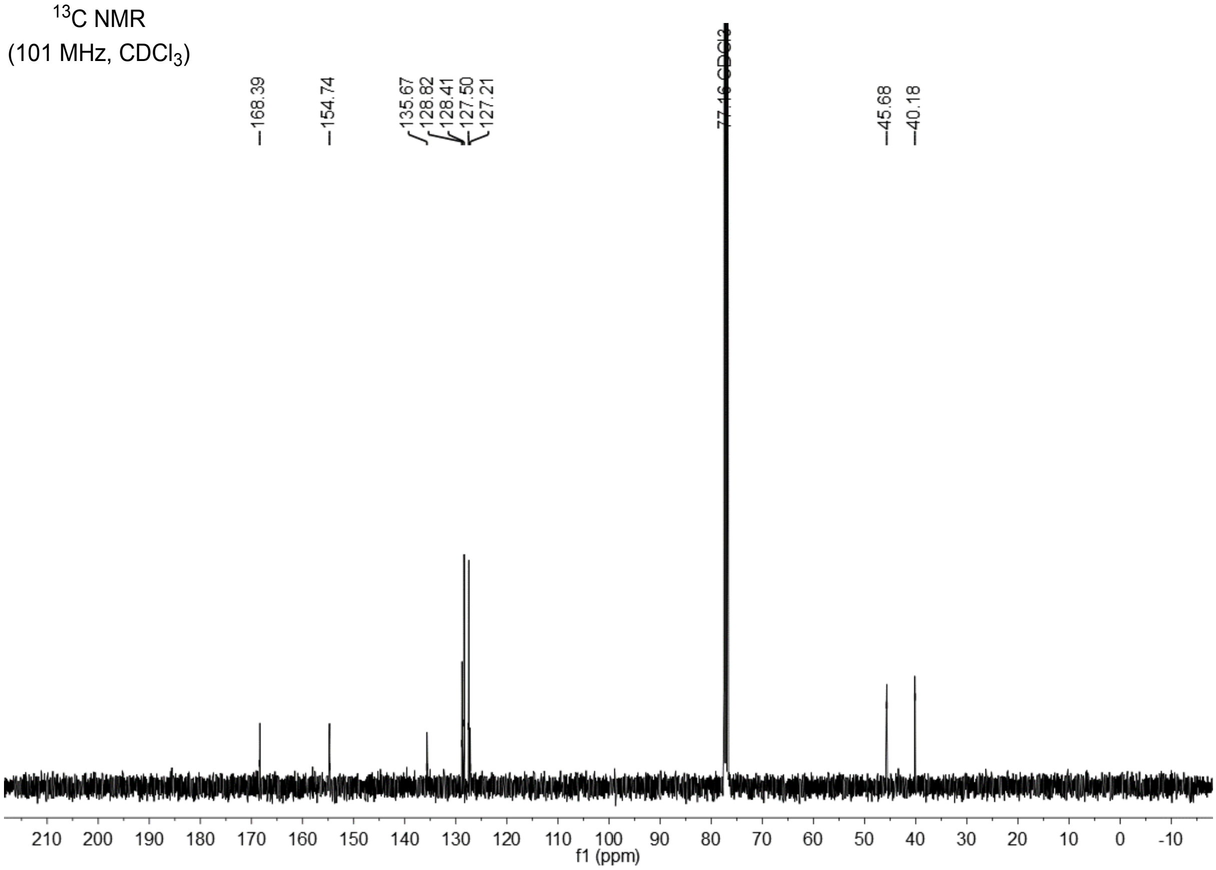

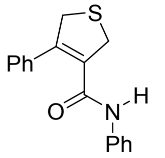

S-96
^{1}H NMR
(400 MHz, $CDCl_3$)

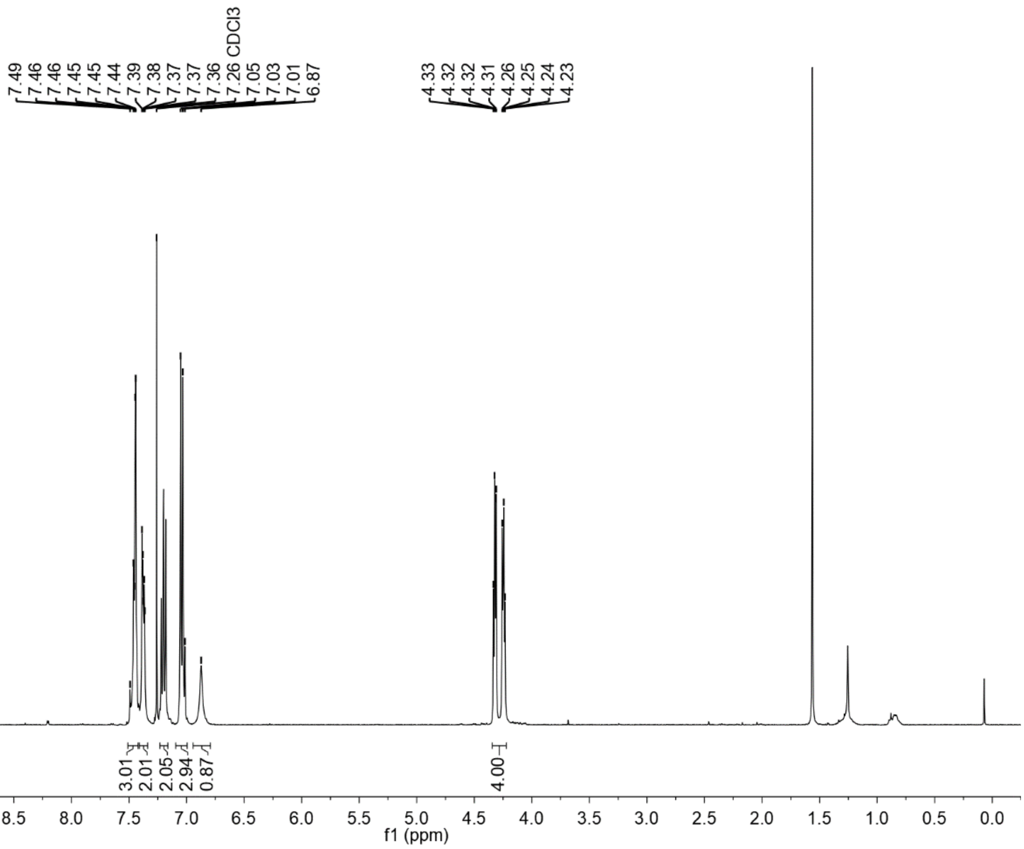

S-96
^{13}C NMR
(101 MHz, $CDCl_3$)

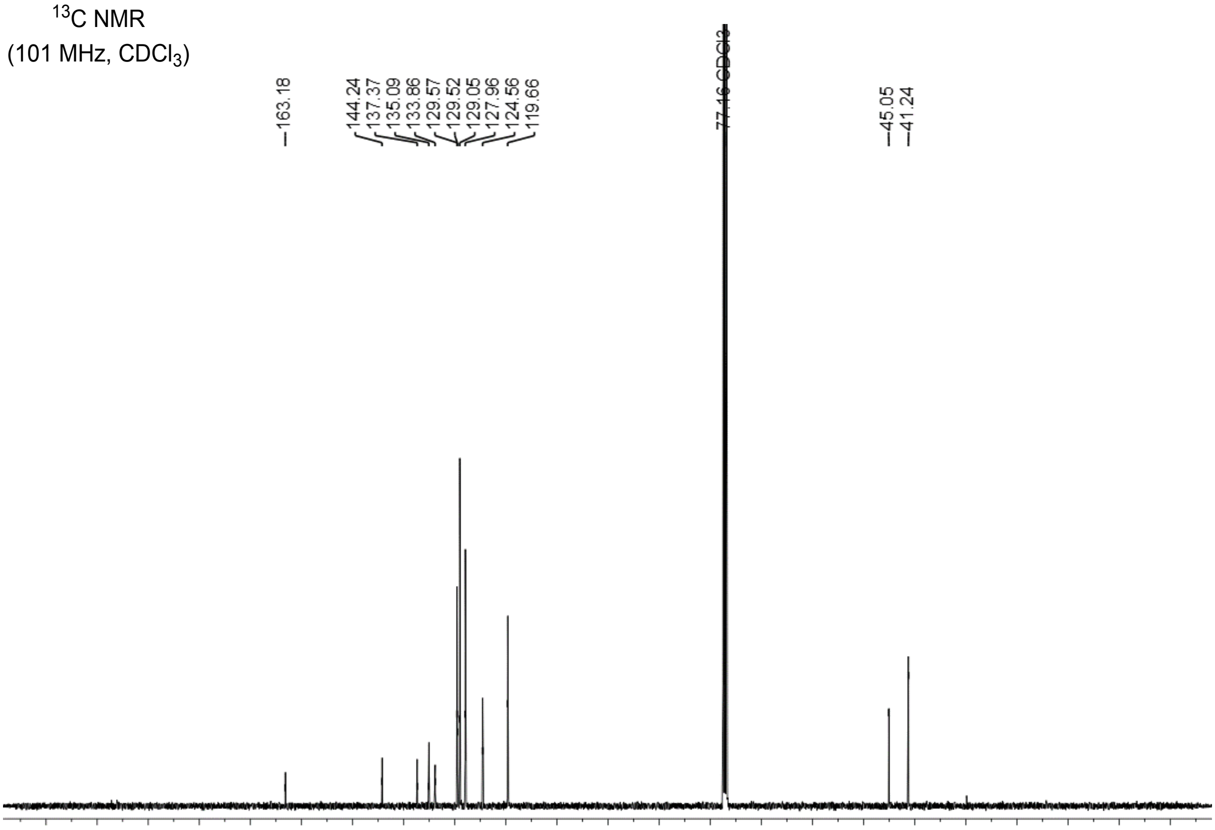

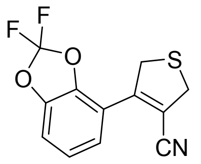

S-98
^{1}H NMR
(400 MHz, $CDCl_3$)

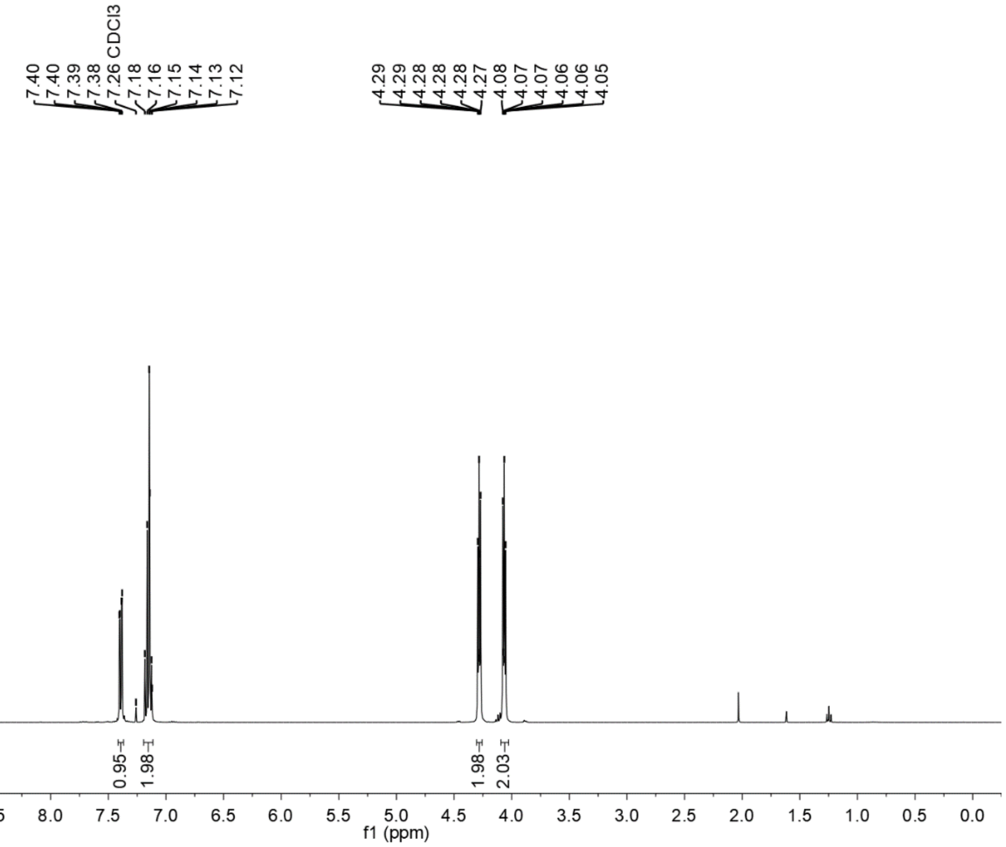

S-98
^{13}C NMR
(101 MHz, $CDCl_3$)

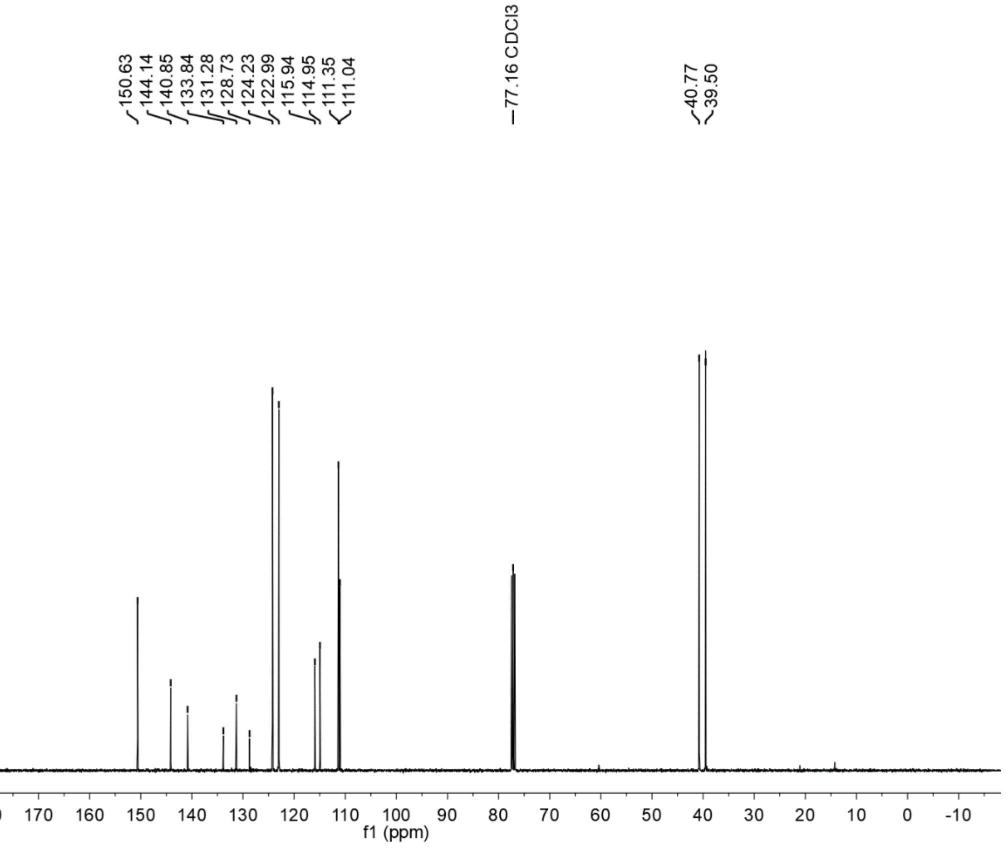

S-98
^{19}F NMR
(376 MHz, $CDCl_3$)

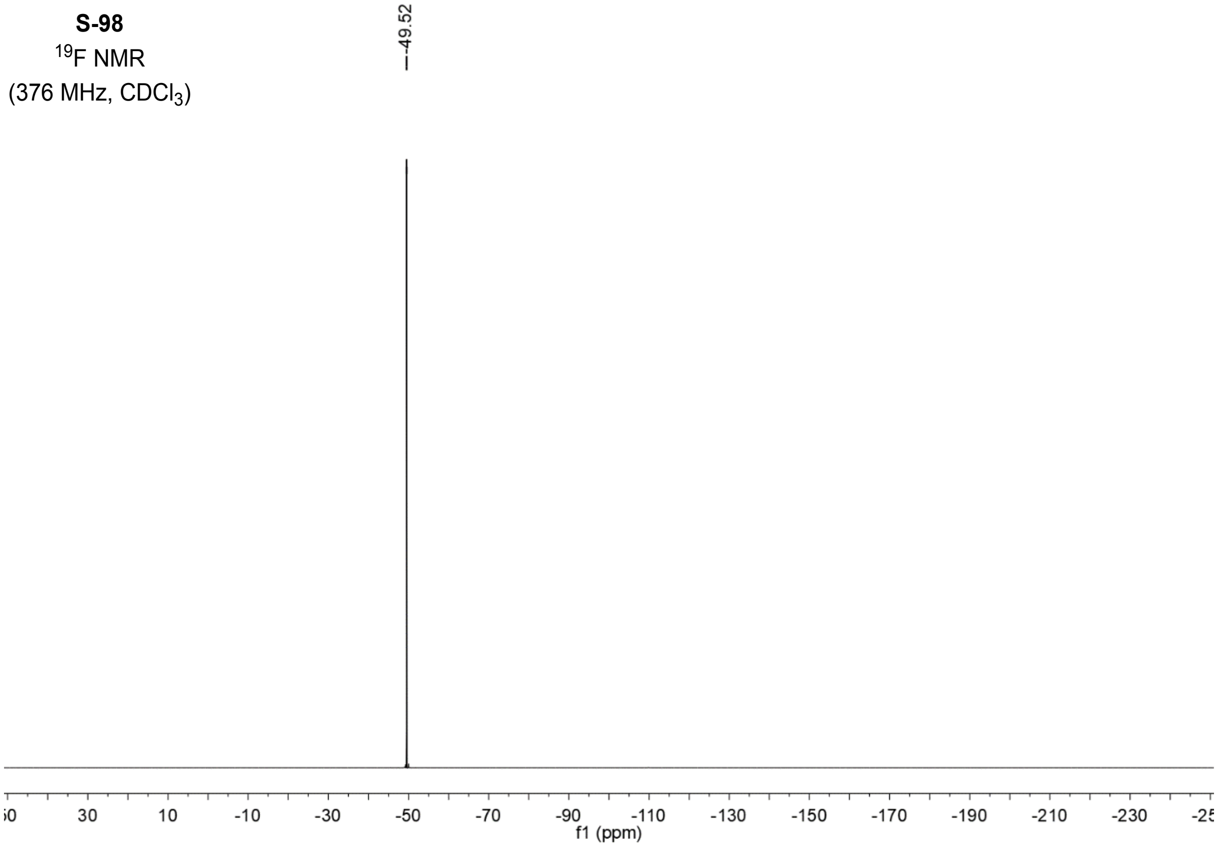

S-101
^{1}H NMR
(400 MHz, $CDCl_3$)

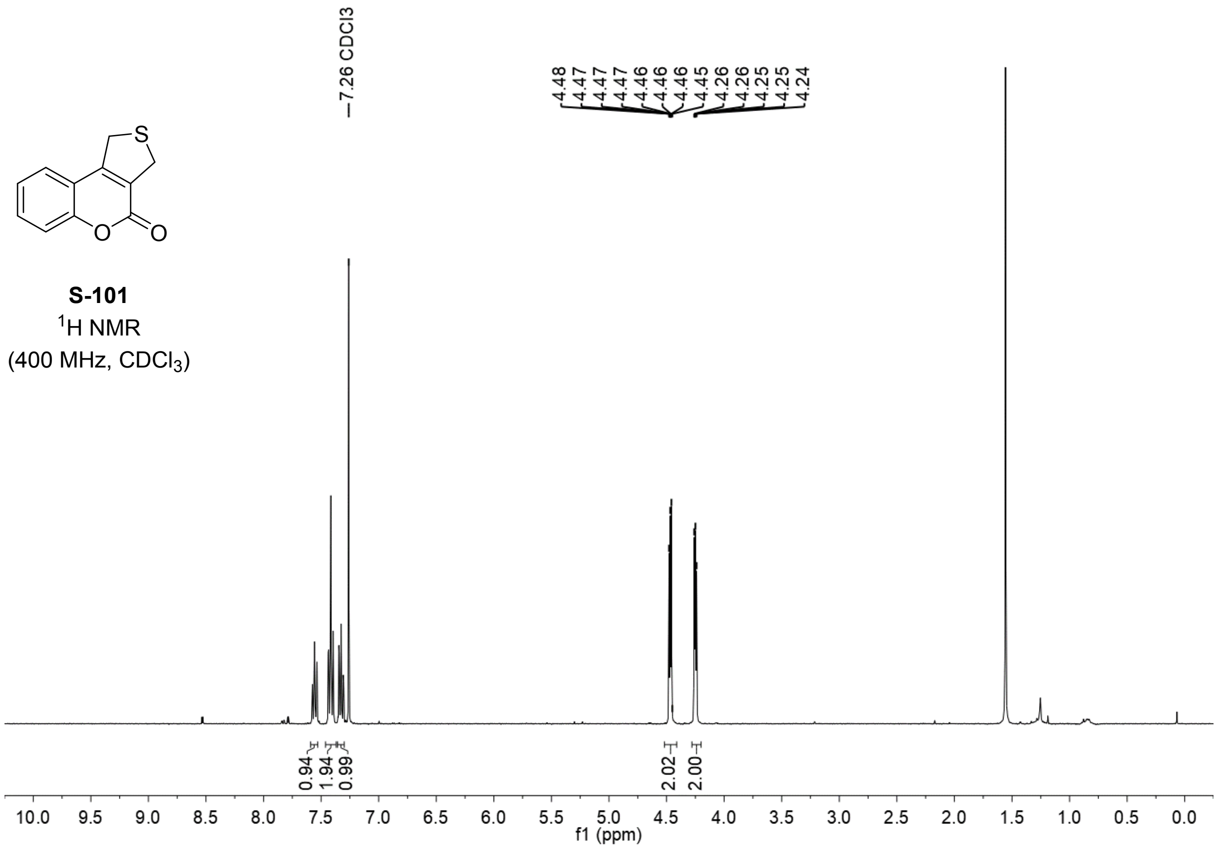

S-101
^{13}C NMR
(101 MHz, $CDCl_3$)

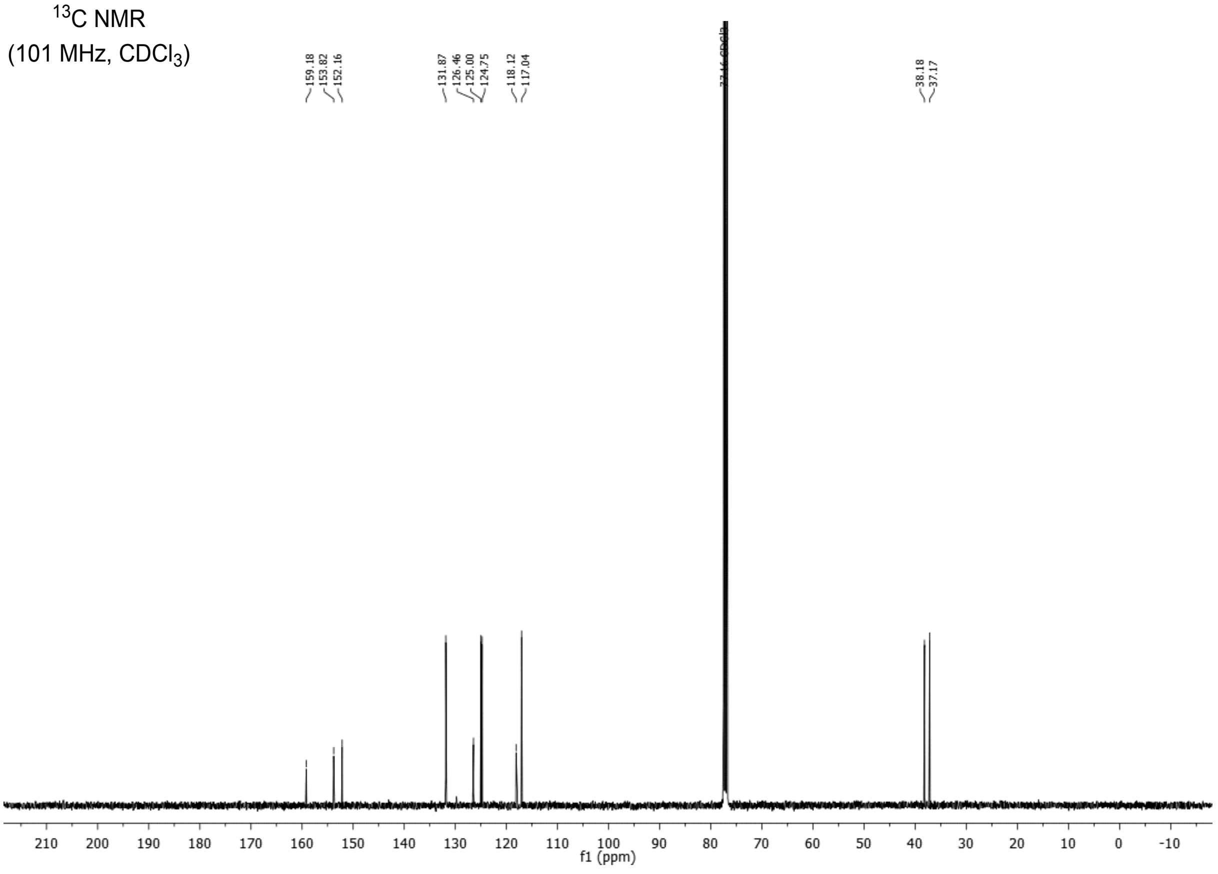

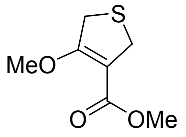

S-102
^{1}H NMR
(400 MHz, $CDCl_3$)

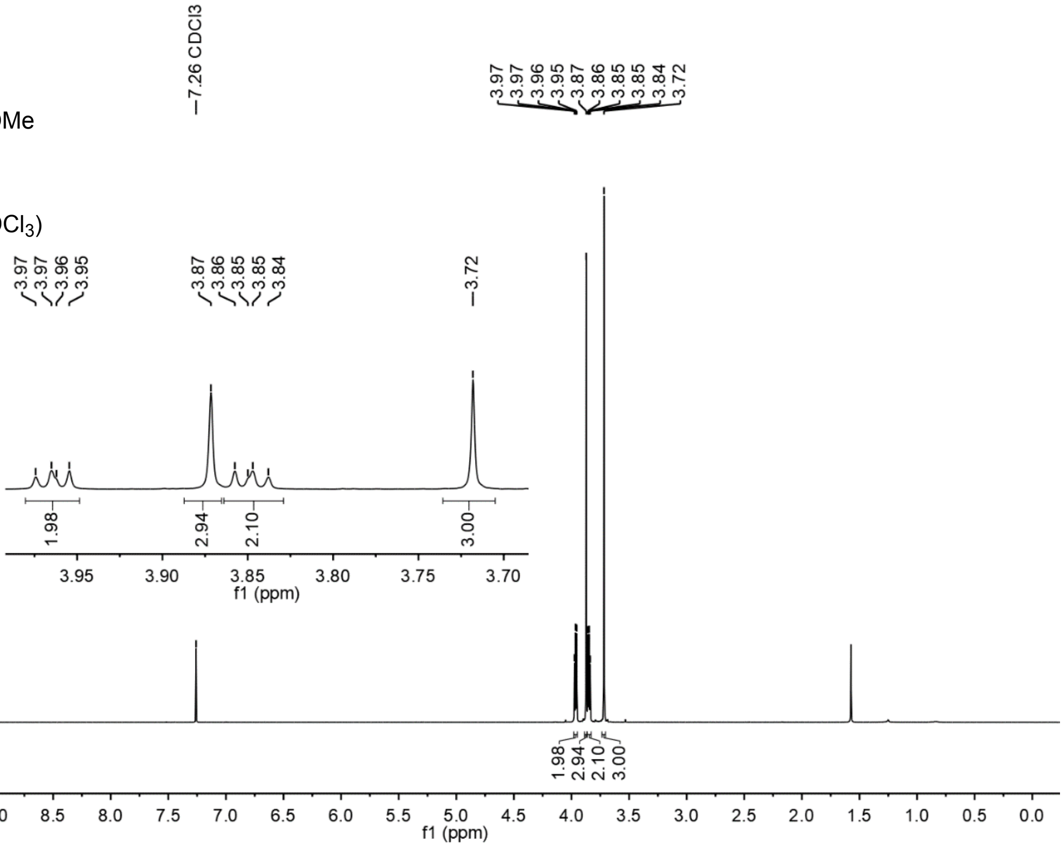

S-102
^{13}C NMR
(101 MHz, $CDCl_3$)

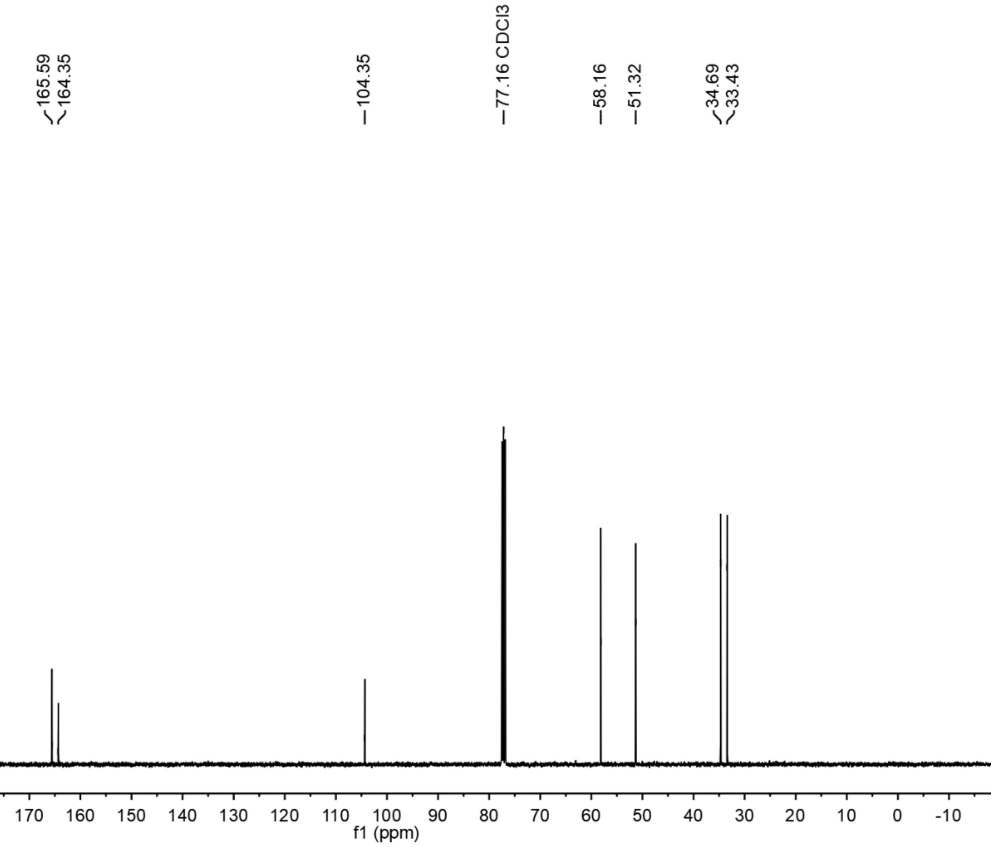

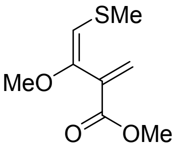

S-103
^{1}H NMR
(400 MHz, $CDCl_3$)

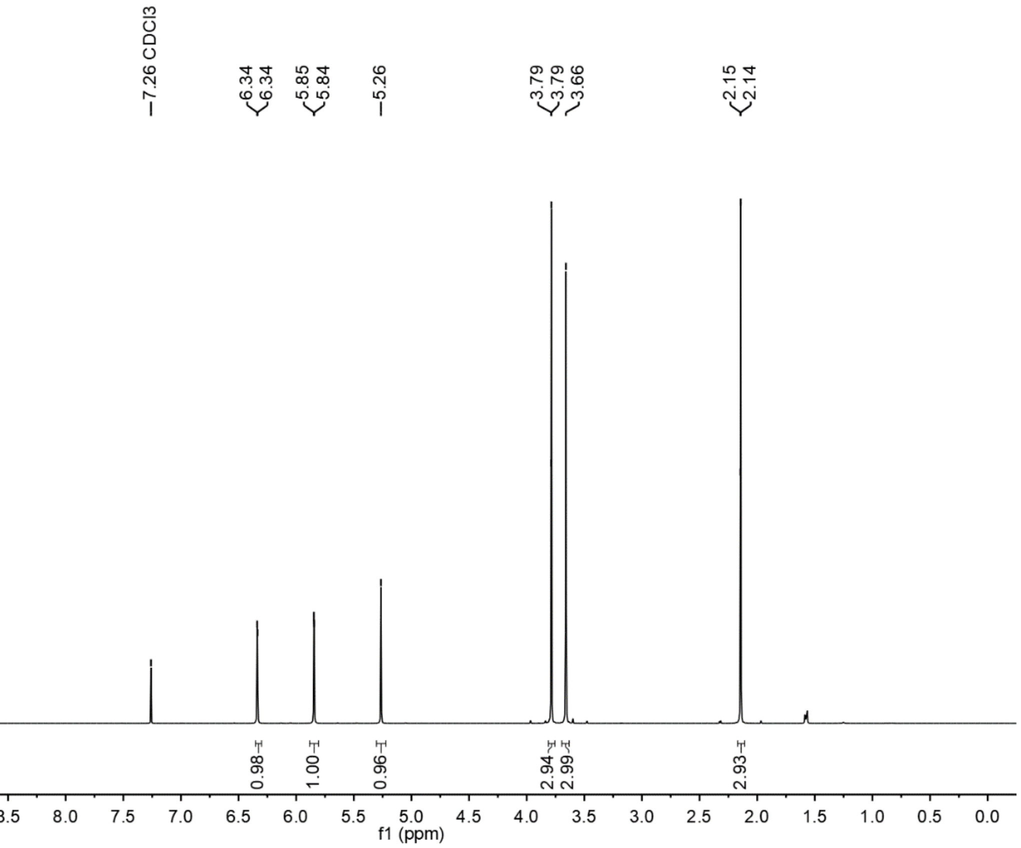

S-103
^{13}C NMR
(101 MHz, $CDCl_3$)

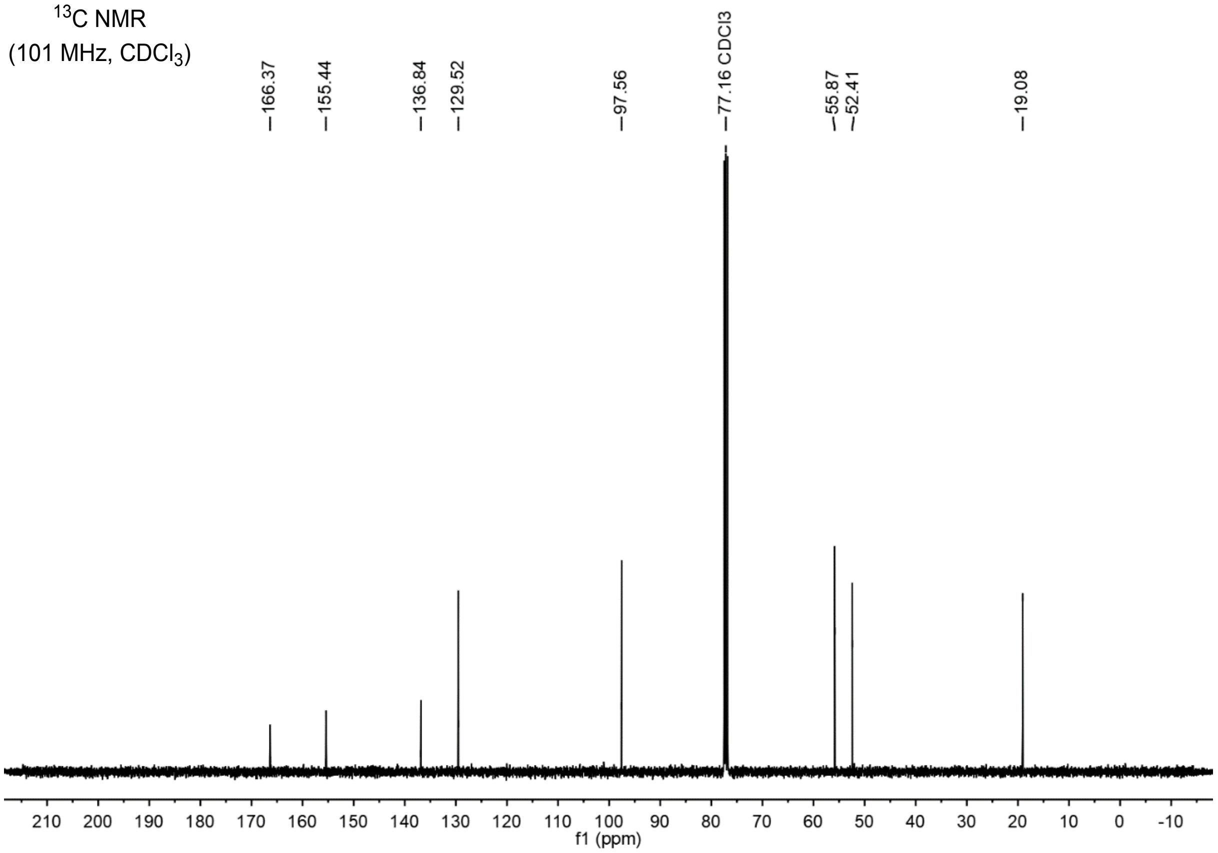

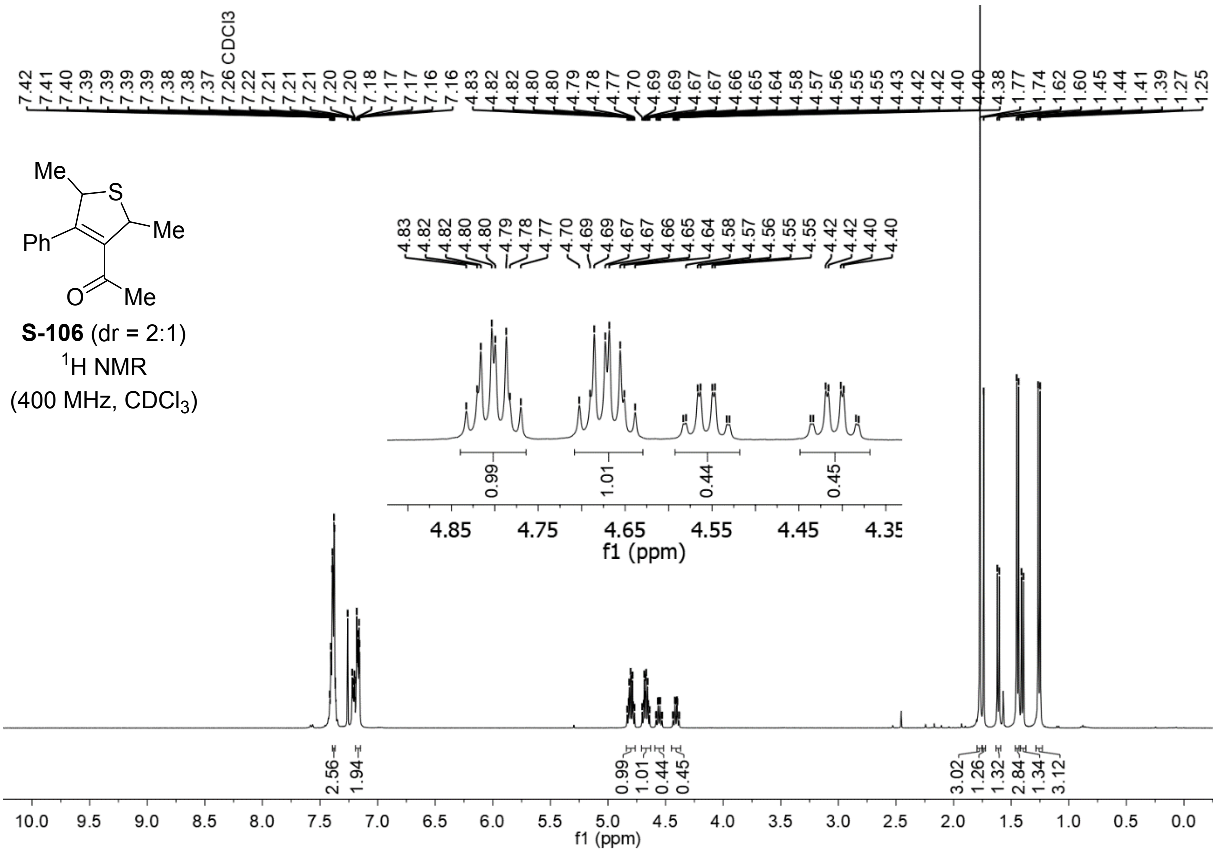

S-106 (dr = 2:1)
^{1}H NMR
(400 MHz, $CDCl_3$)

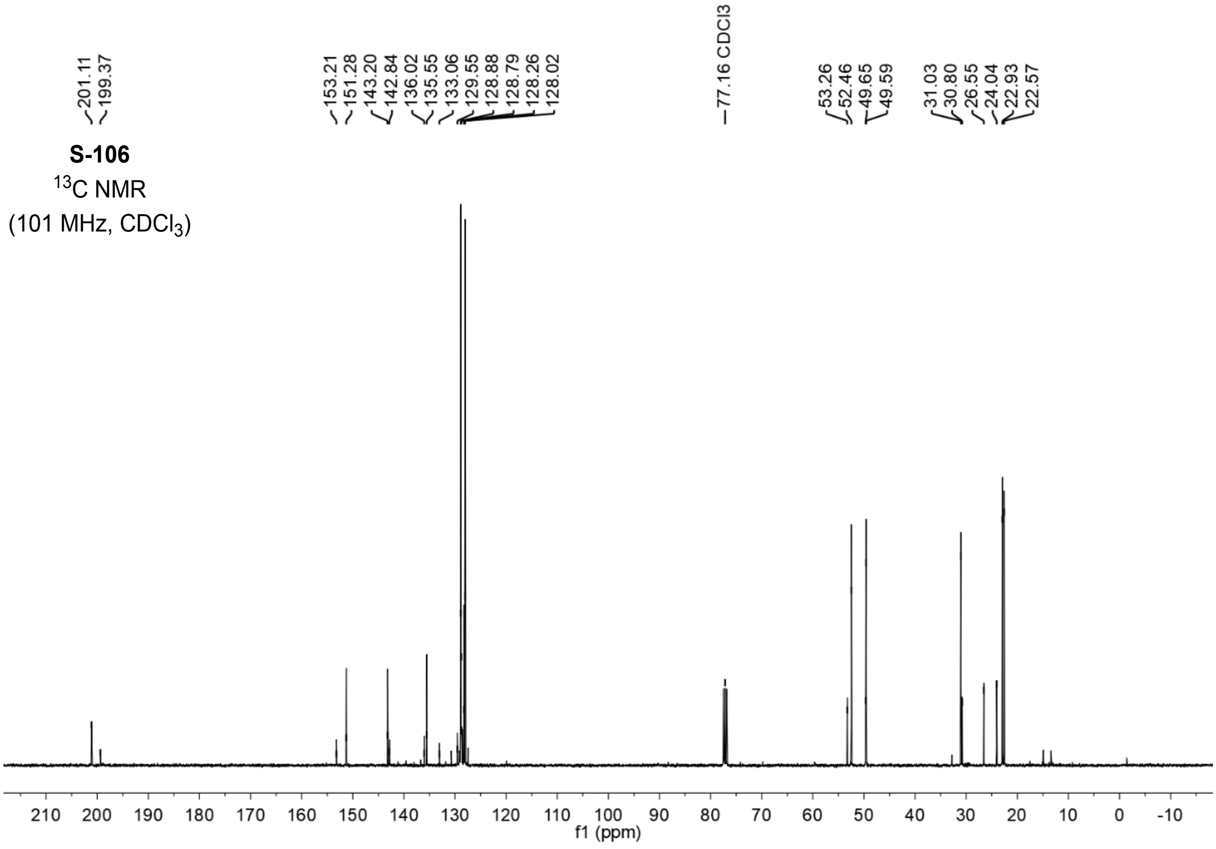

S-106
^{13}C NMR
(101 MHz, $CDCl_3$)

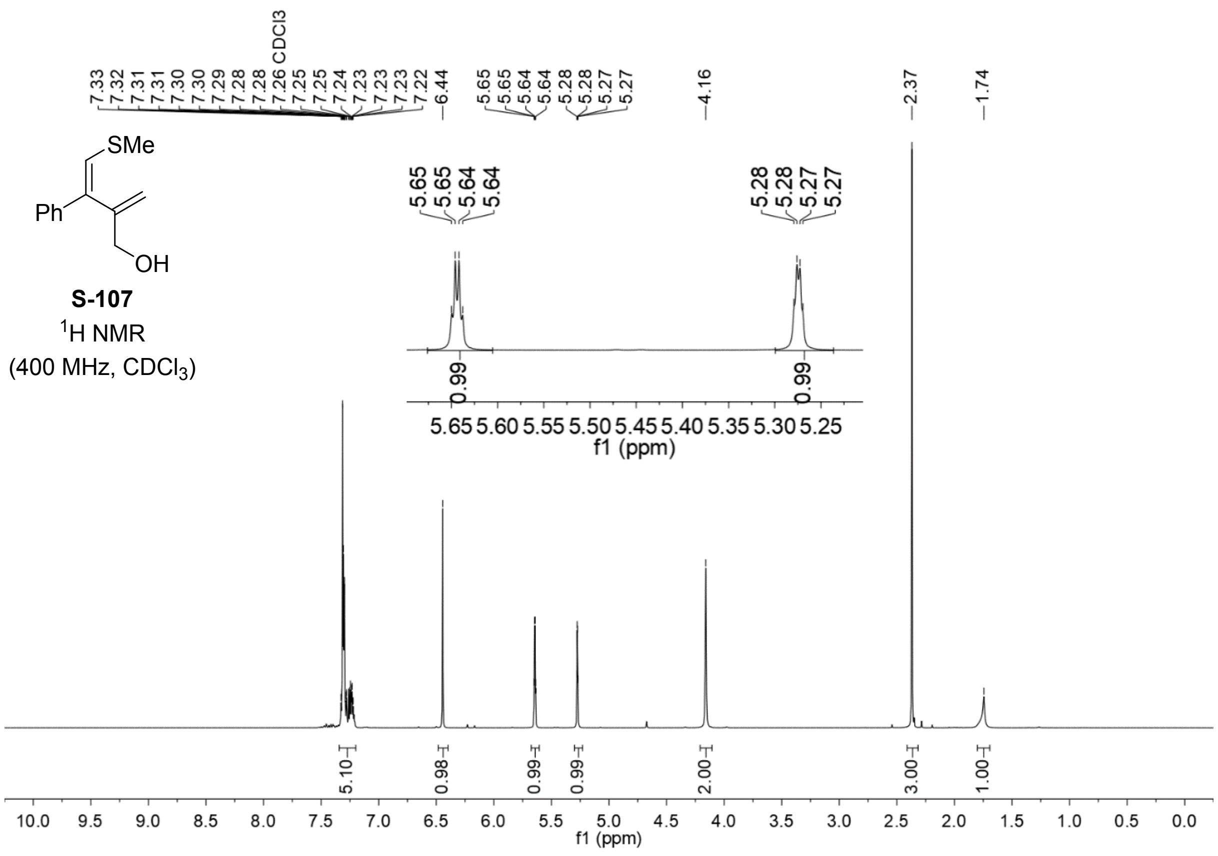
SMe
Ph
OH
S-107
1H NMR
(400 MHz, CDCl3)
7.33 7.32 7.31 7.31 7.30 7.30 7.29 7.28 7.28 7.26 CDCl3 7.25 7.25 7.24 7.23 7.23 7.23 7.23 7.22
6.44
5.65 5.65 5.64 5.64
5.28 5.28 5.27 5.27
4.16
2.37
1.74
5.10 0.98 0.99 0.99 2.00 3.00 1.00
f1 (ppm)

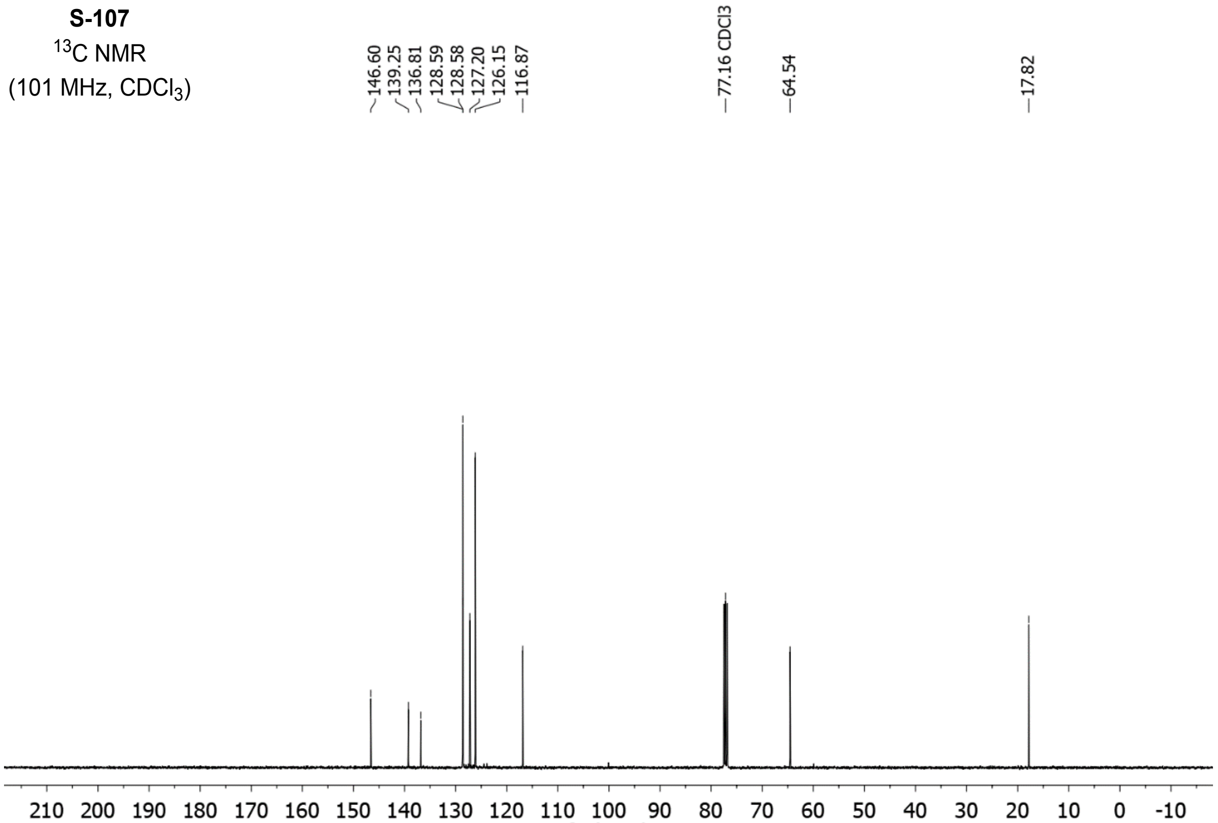
S-107
13C NMR
(101 MHz, CDCl3)
146.60 139.25 136.81 128.59 128.58 127.20 126.15 116.87
77.16 CDCl3
64.54
17.82
f1 (ppm)

S-108

^{1}H NMR

(400 MHz, $CDCl_3$)

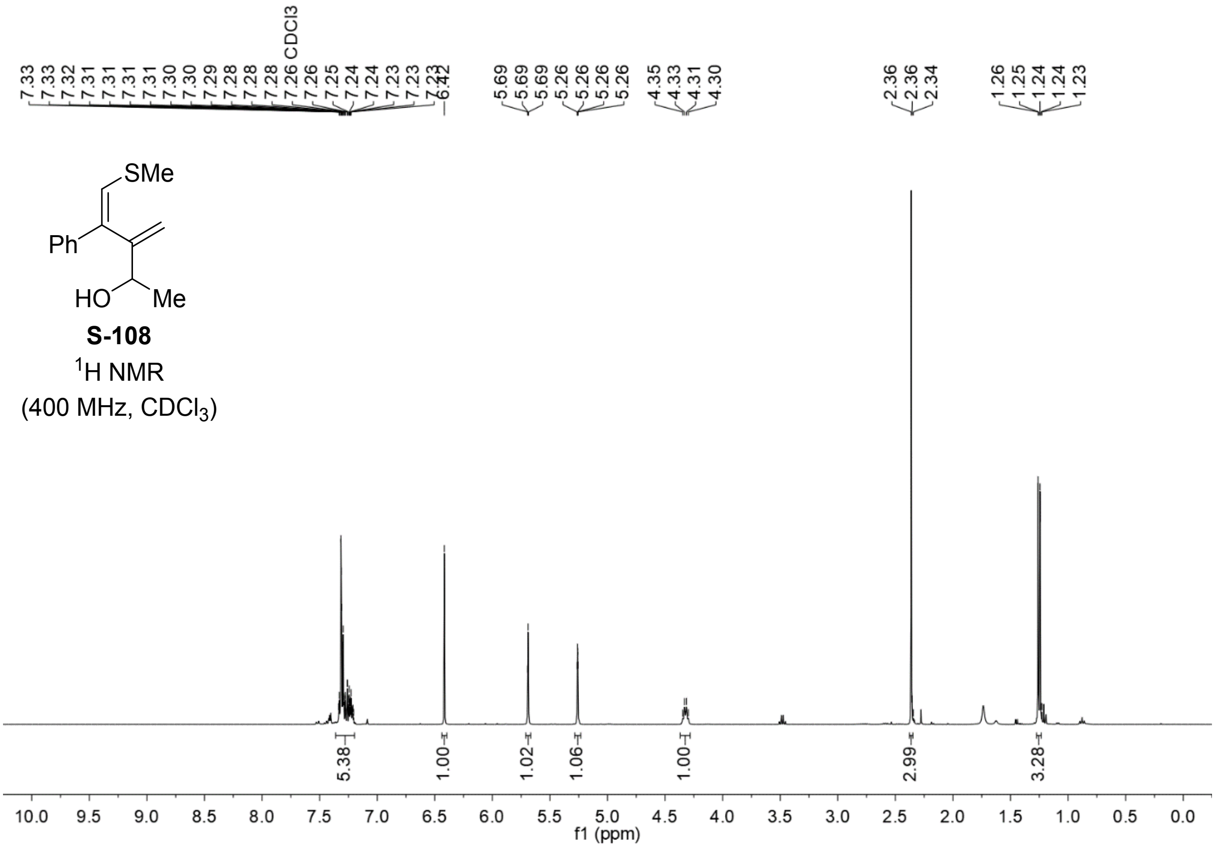

S-108

^{13}C NMR

(101 MHz, $CDCl_3$)

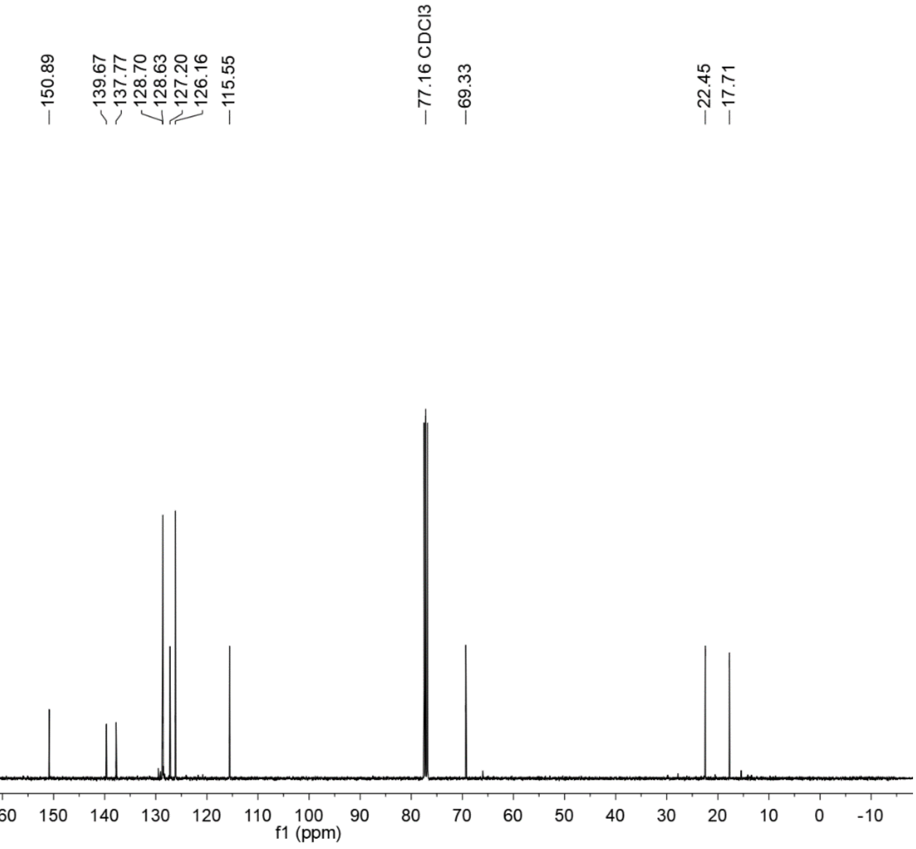

9 Crystallographic Data

Pyrrole 12b

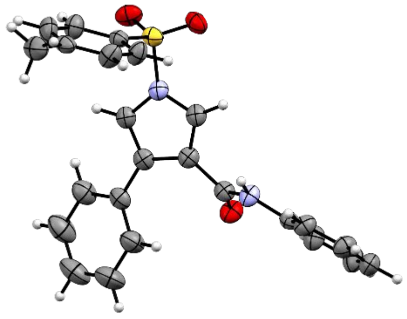

CCDC 2081881 contains the supplementary crystallographic data for **12b**.

Identification code	mar21-4	
Empirical formula	$C_{20}H_{20}N_2O_3S$	
Formula weight	416.48	
Temperature	183(2) K	
Wavelength	0.71073 Å	
Crystal system	Monoclinic	
Space group	$P2_1/c$ (no. 14)	
Unit cell dimensions	a = 9.7088(5) Å	α = 90°
	b = 23.8303(12) Å	β = 116.6810(10)°
	c = 10.0726(4) Å	γ = 90°
Volume	2082.29(17) Å^3	
Z	4	
Density (calculated)	1.328 Mg/m^3	
Absorption coefficient	0.184 mm^{-1}	
F(000)	872	
Crystal size	0.180 × 0.120 × 0.030 mm^3	
Theta range for data collection	2.419 to 24.998°	
Index ranges	–11<=h<=10, –28<=k<=28, –11<=l<=11	
Reflections collected	29657	
Independent reflections	3664 [R(int) = 0.0396]	

Completeness to theta = 24.998°	99.9 %
Absorption correction	Semi-empirical from equivalents
Max. and min. transmission	0.888 and 0.859
Refinement method	Full-matrix least-squares on F^2
Data / restraints / parameters	3664 / 1 / 277
Goodness-of-fit on F^2	1.033
Final R indices [I>2sigma(I)]	R1 = 0.0441, wR2 = 0.1118
R indices (all data)	R1 = 0.0558, wR2 = 0.1178
Largest diff. peak and hole	0.908 and –0.337 e.$Å^{-3}$

Pyrrole 14b

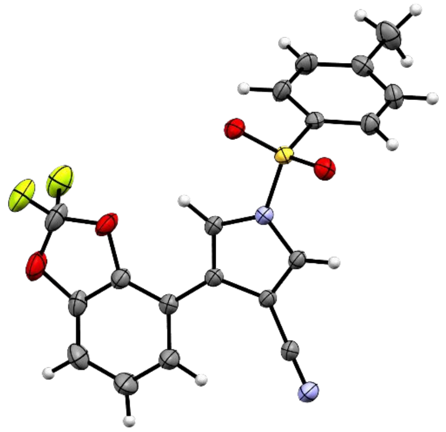

CCDC 2081882 contains the supplementary crystallographic data for **14b**.

Identification code	mar21-2	
Empirical formula	$C_{19}H_{12}F_2N_2O_4$ × 0.5 CH_2Cl_2	
Formula weight	444.83	
Temperature	183(2) K	
Wavelength	0.71073 Å	
Crystal system	Triclinic	
Space group	P-1 (no. 2)	
Unit cell dimensions	a = 4.8072(2) Å	α = 67.1666(17)°
	b = 13.4605(7) Å	β = 88.5473(17)°

	c = 16.1951(9) Å γ = 82.0749(17)°
Volume	956.14(8) $Å^3$
Z	2
Density (calculated)	1.545 Mg/m^3
Absorption coefficient	0.359 mm^{-1}
F(000)	454
Crystal size	0.180 × 0.110 × 0.050 mm^3
Theta range for data collection	2.524 to 25.500°
Index ranges	–5<=h<=5, –16<=k<=16, –19<=l<=19
Reflections collected	33177
Independent reflections	3552 [R(int) = 0.1282]
Completeness to theta = 24.992°	99.8 %
Absorption correction	Semi-empirical from equivalents
Max. and min. transmission	0.977 and 0.944
Refinement method	Full-matrix least-squares on F^2
Data / restraints / parameters	3552 / 0 / 299
Goodness-of-fit on F^2	1.056
Final R indices [I>2sigma(I)]	R1 = 0.0364, wR2 = 0.0976
R indices (all data)	R1 = 0.0399, wR2 = 0.1088
Extinction coefficient	0.081(5)
Largest diff. peak and hole	0.270 and –0.455 $e.Å^{-3}$

Sulfilimine 19

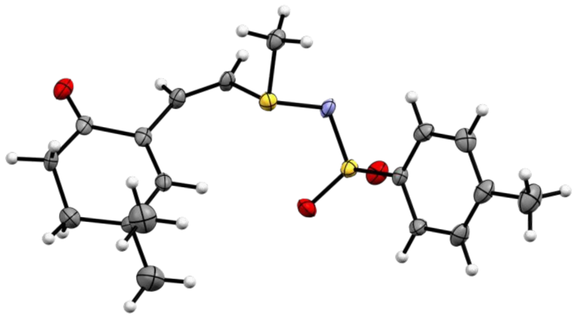

CCDC 2081883 contains the supplementary crystallographic data for **19**.

Identification code	mar20-25	
Empirical formula	$C_{18}H_{23}NO_3S_2$	
Formula weight	365.49	
Temperature	183(2) K	
Wavelength	0.71073 Å	
Crystal system	Triclinic	
Space group	P-1 (no. 2)	
Unit cell dimensions	a = 6.1963(8) Å	α = 94.130(4)°
	b = 11.2965(14) Å	β = 110.244(4)°
	c = 13.9843(16) Å	γ = 96.890(4)°
Volume	904.96(19) $Å^3$	
Z	2	
Density (calculated)	1.341 Mg/m^3	
Absorption coefficient	0.310 mm^{-1}	
F(000)	388	
Crystal size	0.160 × 0.080 × 0.020 mm^3	
Theta range for data collection	2.548 to 23.832°	
Index ranges	–7<=h<=6, –12<=k<=12, 0<=l<=15	
Reflections collected	2683	
Independent reflections	2683 [R(int) = ?]	

Completeness to theta = 24.996°	98.6 %
Absorption correction	Semi-empirical from equivalents
Max. and min. transmission	0.957 and 0.809
Refinement method	Full-matrix least-squares on F^2
Data / restraints / parameters	2683 / 0 / 219
Goodness-of-fit on F^2	1.136
Final R indices [I>2sigma(I)]	R1 = 0.0725, wR2 = 0.1344
R indices (all data)	R1 = 0.1044, wR2 = 0.1464
Extinction coefficient	n/a
Largest diff. peak and hole	0.372 and –0.528 e.Å^{-3}

2,5-Dihydropyrrole 26

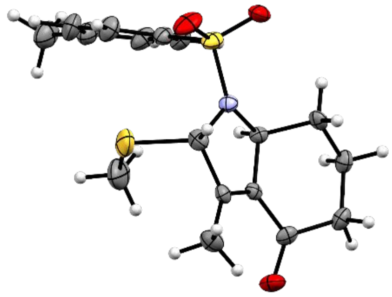

CCDC 2081884 contains the supplementary crystallographic data for **26**.

Identification code	mar21-3	
Empirical formula	$C_{17}H_{21}NO_3S_2$	
Formula weight	351.47	
Temperature	173(2) K	
Wavelength	0.71073 Å	
Crystal system	Monoclinic	
Space group	$P2_1/c$ (no. 14)	
Unit cell dimensions	a = 18.236(2) Å	α = 90°
	b = 8.2875(10) Å	β = 96.168(3)°
	c = 11.3392(13) Å	γ = 90°

Volume	1703.8(4) Å^3
Z	4
Density (calculated)	1.370 Mg/m^3
Absorption coefficient	0.326 mm^{-1}
F(000)	744
Crystal size	0.210 × 0.180 × 0.120 mm^3
Theta range for data collection	2.702 to 24.996°
Index ranges	–21<=h<=21, 0<=k<=9, 0<=l<=13
Reflections collected	3002
Independent reflections	3002 [R(int) = ?]
Completeness to theta = 24.996°	99.8 %
Absorption correction	Semi-empirical from equivalents
Max. and min. transmission	0.990 and 0.916
Refinement method	Full-matrix least-squares on F^2
Data / restraints / parameters	3002 / 0 / 213
Goodness-of-fit on F^2	1.180
Final R indices [I>2sigma(I)]	R1 = 0.0456, wR2 = 0.0949
R indices (all data)	R1 = 0.0594, wR2 = 0.0998
Extinction coefficient	0.0150(14)
Largest diff. peak and hole	0.221 and –0.297 e.Å^{-3}
